# Quantitative Label-Free Proteomics for Discovery of Biomarkers in Cerebrospinal Fluid: Assessment of Technical and Inter-Individual Variation

**DOI:** 10.1371/journal.pone.0064314

**Published:** 2013-05-20

**Authors:** Richard J. Perrin, Jacqueline E. Payton, James P. Malone, Petra Gilmore, Alan E. Davis, Chengjie Xiong, Anne M. Fagan, R. Reid Townsend, David M. Holtzman

**Affiliations:** 1 Division of Neuropathology, Washington University School of Medicine, St. Louis, Missouri, United States of America; 2 Division of Laboratory and Genomic Medicine, Washington University School of Medicine, St. Louis, Missouri, United States of America; 3 Department of Pathology and Immunology, Washington University School of Medicine, St. Louis, Missouri, United States of America; 4 Department of Medicine, Washington University School of Medicine, St. Louis, Missouri, United States of America; 5 Department of Cell Biology and Physiology, Washington University School of Medicine, St. Louis, Missouri, United States of America; 6 Division of Biostatistics, Washington University School of Medicine, St. Louis, Missouri, United States of America; 7 Department of Neurology, Washington University School of Medicine, St. Louis, Missouri, United States of America; 8 Knight Alzheimer Disease Research Center, Washington University School of Medicine, St. Louis, Missouri, United States of America; 9 Hope Center for Neurological Disorders, Washington University School of Medicine, St. Louis, Missouri, United States of America; Federal University of São Paulo (UNIFESP), Escola Paulista de Medicina, Brazil

## Abstract

**Background:**

Biomarkers are required for pre-symptomatic diagnosis, treatment, and monitoring of neurodegenerative diseases such as Alzheimer's disease. Cerebrospinal fluid (CSF) is a favored source because its proteome reflects the composition of the brain. Ideal biomarkers have low technical and inter-individual variability (subject variance) among control subjects to minimize overlaps between clinical groups. This study evaluates a process of multi-affinity fractionation (MAF) and quantitative label-free liquid chromatography tandem mass spectrometry (LC-MS/MS) for CSF biomarker discovery by (1) identifying reparable sources of technical variability, (2) assessing subject variance and residual technical variability for numerous CSF proteins, and (3) testing its ability to segregate samples on the basis of desired biomarker characteristics.

**Methods/Results:**

Fourteen aliquots of pooled CSF and two aliquots from six cognitively normal individuals were randomized, enriched for low-abundance proteins by MAF, digested endoproteolytically, randomized again, and analyzed by nano-LC-MS. Nano-LC-MS data were time and m/z aligned across samples for relative peptide quantification. Among 11,433 aligned charge groups, 1360 relatively abundant ones were annotated by MS2, yielding 823 unique peptides. Analyses, including Pearson correlations of annotated LC-MS ion chromatograms, performed for all pairwise sample comparisons, identified several sources of technical variability: i) incomplete MAF and keratins; ii) globally- or segmentally-decreased ion current in isolated LC-MS analyses; and iii) oxidized methionine-containing peptides. Exclusion of these sources yielded 609 peptides representing 81 proteins. Most of these proteins showed very low coefficients of variation (CV<5%) whether they were quantified from the mean of all or only the 2 most-abundant peptides. Unsupervised clustering, using only 24 proteins selected for high subject variance, yielded perfect segregation of pooled and individual samples.

**Conclusions:**

Quantitative label-free LC-MS/MS can measure scores of CSF proteins with low technical variability and can segregate samples according to desired criteria. Thus, this technique shows potential for biomarker discovery for neurological diseases.

## Introduction

Dementia of the Alzheimer type (DAT) currently affects an estimated 30 million people worldwide. This number is expected to grow three-fold over the next 40 years as the population ages [1]. In addition to those affected by DAT, many more are afflicted by Alzheimer's disease (AD, the pathological process responsible for DAT) but have not yet begun to experience symptoms. Individuals in this 10- to 15-year pre-symptomatic or ‘pre-clinical’ phase of AD are at increased risk to develop dementia [2]–[5] but have not yet experienced significant neuronal damage [6], [7]. For this reason, they are likely to receive relatively greater benefit from disease modifying treatments that are on the horizon. Indeed, the failure of many recent clinical trials aimed at AD is commonly attributed to the exclusive enrollment of participants who already have mild or moderate dementia and concomitant neuron loss. Therefore, tools and strategies (biomarkers) must be developed to diagnose and enroll individuals in the pre-clinical phase of AD, when brain pathology is present but cognition remains intact. By definition, this phase is not reliably detected by clinical examination, so biomarkers (for example, those measured by radiographic imaging and laboratory tests) will be required for diagnosis. Ideally, biomarkers should also estimate an individual's risk of impending cognitive decline (prognosis) and even allow monitoring of pathological progression and response to treatment. Once such biomarkers are developed, clinical trials should become more efficient and effective treatments will be identified more quickly. Subsequently, once successful treatments are identified, these biomarkers are likely to remain useful in a clinical setting.

Some progress has already been made in this direction. To date, leading modalities for such biomarkers include radiological imaging and cerebrospinal fluid (CSF) analysis (reviewed in references [1], [8], [9]). Both techniques can detect amyloid deposits (Alzheimer plaques) in the brain either directly, using amyloid-binding tracer compounds (e.g. Pittsburgh compound B, or PIB) and positron emission tomography, or indirectly, by measuring low CSF beta-amyloid42 (Aβ42) concentrations that correlate with amyloid deposition [3], [10]–[14]. Imaging and fluid biomarker studies have also shown potential to predict cognitive decline by measuring amyloid deposition [5], regional volumetric and metabolic changes in the brain [15]–[17], or specific changes in the CSF proteome (including concentrations of tau, YKL-40, VILIP-1, and calbindin, each in association with Aβ42) [2], [3], [18], [19]. CSF analysis may even allow classification of disease stage [20] and monitoring of acute changes in response to disease modifying therapies, as illustrated recently with gamma-secretase inhibitors [21]. In spite of these advances, however, these techniques must still be improved. Additional biomarkers will be required to improve the sensitivity and specificity of pre-clinical AD diagnosis, increase the accuracy of prognosis, and expand the breadth of pathophysiological changes that can be monitored.

CSF proteome analysis provides a favorable arena for such efforts. Indeed, many increasingly more powerful yet complementary proteomics technologies have been leveled at CSF biomarker discovery in the past decade, including: variations of 2D gel electrophoresis [2], [20], [22]–[30]; SELDI-TOF-MS [31]–[34]; offline LC – MALDI-TOF [35]; and LC-MS/MS with either isotope-coded affinity tags (ICAT) [36], Tandem Mass Tags [37] or iTRAQ (isobaric tag for relative and absolute quantification) [38], [39]. These techniques have all been used successfully to identify candidate biomarkers because they provide accurate relative quantitative information between or among samples. In order to provide this information, they share a common requirement: proteins or peptides must be stained or labeled for precise and accurate quantification. This requirement necessarily increases procedural costs and also may introduce additional sources of error. These techniques also share a major limitation in clinical proteomics (for review see [40]): they cannot readily be used to compare directly large numbers of samples, even with advances in multiplexing technologies and strategies [41]. This second shortcoming is quite important because most CSF biomarkers, at least in the AD field, show relatively modest disease-associated quantitative changes, on the order of 30%; detecting such differences with statistical rigor in a cross-sectional study requires precise and accurate measurements with potentially hundreds of CSF samples.

Label-free, quantitative proteomic methods have emerged that obviate the requirement for protein staining or peptide labeling [41]. Many of these ‘label-free’ approaches take advantage of the correlation between high-resolution LC/MS extracted ion currents (XIC's) and peptide abundances [42], [43]. Bioinformatics software tools have been developed that align LC elution times and accurate m/z values of the XIC's across numerous samples (n∼10–100) [44]. Thus, the signals of XICs with identical retention/elution times and m/z values can be directly compared (mathematically and visually) to measure statistically significant differences between sample groups. The actual sequences and genes of origin of the peptides responsible for XICs of interest can be determined by LC-MS/MS. Given sufficient sample and analytical time, extensive annotated libraries that match XICs (defined by elution time and m/z value) to their unique peptide sequences can be accumulated for a given sample type (e.g. CSF). This accurate mass and time tag (AMT) approach can be applied retrospectively or prospectively, reducing or eliminating the need for tandem mass spectrometry in subsequent studies of that biofluid within a given laboratory [45]. Alternatively, MS2 can be performed in series with LC-MS during primary quantitative data acquisition.

In this work, we apply quantitative label-free LC-MS/MS to the analysis of replicate CSF samples: to identify sources of technical variability that can be mitigated; to assess the inter-individual and residual technical variance with which this technique measures numerous proteins in cognitively normal (healthy control) subject samples; to compare alternative strategies for protein quantification from quantitative peptide data; and to test the ability of its output to segregate biological samples according to desired biomarker characteristics. In this way, we demonstrate the suitability of quantitative label-free LC-MS/MS as a tool for CSF biomarker discovery.

## Materials and Methods

### Ethics Statement

The study protocols were approved by the Human Research Protection Office at Washington University. Written and verbal informed consent were obtained from participants at enrollment and annually, thereafter. Capacity to consent was assessed in the following manner. Each participant was recruited with a ‘collateral source’ (spouse, next-of-kin, or close friend) to accompany them at research interviews, to provide information about the participant's level of memory impairment and to assess the participant's willingness to participate in the research. Cognitive status (dementia level) was determined in a 2 hour semi-structured interview conducted by dementia experts. Whereupon a participant was determined to have mild, moderate or severe dementia, the Durable Power of Attorney for Health Care or next-of-kin was asked to provide or renew consent. All aspects of this study were conducted according to the principles expressed in the Declaration of Helsinki.

### Participant Selection and Sample Preparation

**Figure 1:**
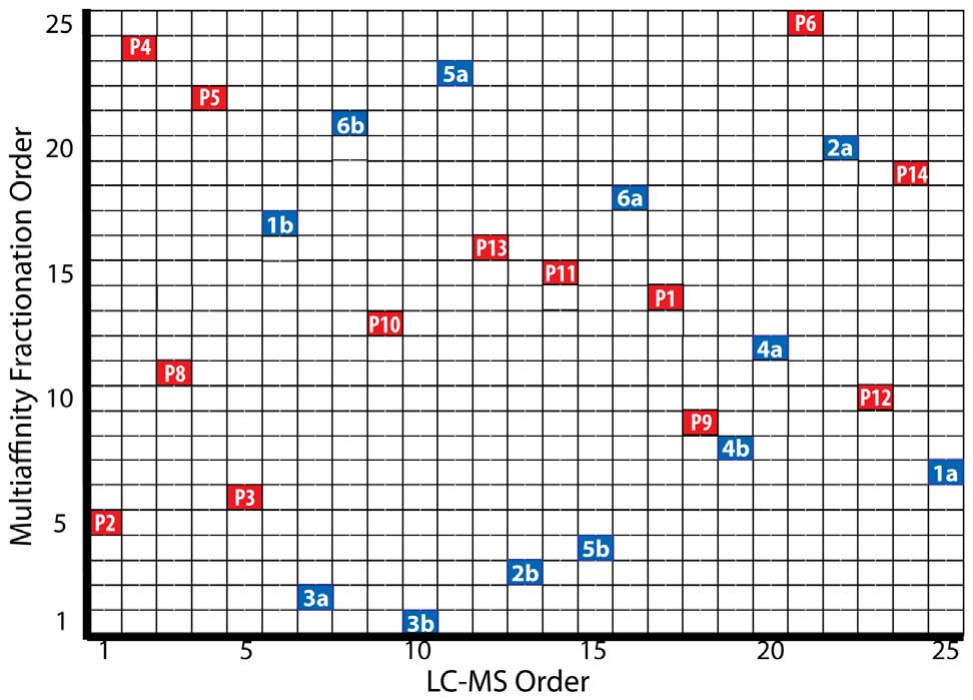
Chromatographic run order for MAF and LC-MS analysis. Duplicate aliquots of CSF (denoted by ‘a’ and ‘b’) from each of six cognitively normal individuals (numbered 1 through 6) and fourteen aliquots of CSF (labeled P1 through P14) pooled from 100 individuals with mild [n = 9], very mild [n = 33], and no [n = 58] dementia of the Alzheimer type were selected for single block proteomic analysis. The order that samples underwent MAF (Y-axis) and then LC-MS analysis (X-axis) were independently randomized. A single sample (P7) that did not pass the 1D SDS-PAGE quality assessment (Fig. S1) and was not analyzed by LC-MS is not represented.

14 replicates of a pooled sample of CSF were evaluated for assessment of coefficients of variation (CV); two aliquots of CSF samples from 6 cognitively normal individuals were evaluated for subject variance (Fig. 1). Participants, community-dwelling volunteers enrolled at the Knight Alzheimer Disease Research Center at Washington University, were ≥60 years of age and in good general health, having no other neurological, psychiatric, or major medical diagnoses that could contribute to dementia, nor use of exclusionary medications (e.g. anticoagulants) within 1–3 months of lumbar puncture (LP). Individuals were not excluded on the basis of gender, race, ethnicity, or APOE genotype. Cognitive status was evaluated based on criteria from the National Institute of Neurological and Communicative Diseases and Stroke-Alzheimer's Disease and Related Disorders Association [46]. Samples were de-identified and coded immediately after initial collection. Samples processed individually were selected from among many stored frozen samples donated by cognitively normal individuals (with a Clinical Dementia Rating of zero [CDR 0]) on the basis of high aliquot abundance and generally low CSF tau and high CSF Aβ42 levels (a biomarker profile consistent with the absence of AD pathology [10], [47], [48]); ranges of actual ‘individual’ samples chosen were: age of participant at LP, 62–80 years; Tau, 176–393 pg/mL; p-tau181, 37.2–108 pg/mL; Aβ42, 283–703 pg/mL. For creation of a pooled sample, stored frozen samples collected from participants who were cognitively normal (CDR 0, n = 58), very mildly demented (CDR 0.5, n = 33) or mildly demented (CDR 1, n = 9) at the time of LP were selected without regard to CSF tau and CSF Aβ42 measurements. Some CDR 0.5 participants met criteria for mild cognitive impairment (MCI); others showed even milder impairment, and could be considered “pre-MCI” [49]. All CDR 1 individuals had received a diagnosis of DAT. For each individual LP, fasted CSF (20–30 mL) was collected, gently mixed, centrifuged, aliquoted (0.5 mL) and frozen at −80°C in polypropylene tubes (2.0 mL tubes for storage) as described [3]. For preparation of pooled sample aliquots, all selected 0.5 mL samples were simultaneously thawed in an ice slurry within a 4°C room, combined and gently but thoroughly mixed in larger polypropylene tubes, re-aliquoted (0.5 mL) into fresh pre-chilled 2.0 mL polypropylene tubes, and frozen and stored at −80°C until use. Replicate samples from individuals were also processed in this manner; for each sample, two 0.5 mL aliquots of each sample were thawed, combined, mixed, re-aliquoted and re-frozen as described above.

### Enzyme Linked Immunosorbent Assays (ELISAs)

CSF samples were analyzed by ELISA in duplicate for Aβ42, total tau, and phospho-tau181 (INNOTEST, Innogenetics, Ghent, Belgium) after one freeze-thaw cycle.

### Multi-affinity Fractionation (MAF) of CSF

To enrich for proteins of low-abundance, each sample was depleted of six proteins that are highly abundant in CSF (albumin, IgG, IgA, haptoglobin, transferrin, and α-1-antitrypsin) by immunoaffinity chromatography (Agilent Technologies, Palo Alto, CA) using an automated adaptation of the method described in [29]. Multi-affinity fractionation was performed on a BioCad Vision Workstation, using a Cavro AFC 2000 autosampler/fraction collector. The affinity runs were monitored with a UV detector at 280 nm. The fluid path configuration of the automated chromatograph is shown in Fig. S1A. For automated MAF, an equal volume (0.5 mL) of 2X TBS (20 mM Tris-HCl, 300 mM NaCl, pH = 7.4) was added to each of the frozen CSF samples. After gentle inversion, each sample was filtered through a 0.45 µm filter unit (Millipore, Billerica MA) and an 800 µL aliquot of the filtrate was diluted to 2100 µL with 1X TBS. The diluted samples were injected (applied to the affinity column) in randomized order (Fig. 1) from an autosampler at 4°C. Bound proteins were eluted from the column with 25 mL of 100 mM glycine buffer, pH = 2.5, and discarded. The affinity column was then neutralized with 100 mM Tris-Cl, pH = 8 and re-equilibrated with TBS pH = 7.4. The flow-through fraction was transferred to a concentrating device (Amicon Ultra-15, nominal molecular weight cut off = 3 kDa) and centrifuged according to manufacturer's guidelines (4000×g, 4°C), reducing the volume to ∼300 µL for subsequent analyses.

### Analytical 1D SDS-PAGE

The reproducibility of automated MAF of the CSF samples was initially evaluated using analytical SDS-PAGE. Protein concentrations of the concentrated CSF samples were determined using the Advanced Protein Assay reagent (Cytoskeleton, Denver, CO) against a curve made with BSA standard solution (Pierce, Rockford, IL), measured at 590 nm. Aliquots of the concentrated samples, each containing 5 µg of protein (∼10 µL), were diluted with 5 µL of 4X sample buffer (Bio-Rad Laboratories, Hercules, CA) and 1 µl of 20X reductant (Bio-Rad), heated to 95°C for 5 min, cooled to room temperature, centrifuged at 13000 rpm for 30–60 s and loaded with molecular weight markers (Bio-Rad Precision Plus Protein standards, cat # 161-0363) onto 4–12% Criterion XT Bis-Tris gels. Gels were run in MES buffer, monitored using the blue dye front, placed in fixative solution (10% methanol, 5% acetic acid) for 1 hour, stained with SyproRuby (Invitrogen, Carlsbad, CA) for 2 h, destained (10% methanol, 5% acetic acid) for 30 min and scanned on a Typhoon 9400 scanner (GE Healthcare, United Kingdom) using the following settings: 457 nm excitation, 610BP30 emission filter, photomultiplier tube voltage adjusted to stay below saturation for the darkest band (Fig. S1B,C).

### Preparation of peptides from MAF CSF

The concentrated, unbound eluates from the multi-affinity columns were precipitated using the vendor protocol for the 2D clean-up kit (GE Healthcare, Pittsburgh, PA, Cat. No. 80-6484-51). Protein pellets were solubilized in 20 µL of Tris buffer (100 mM, pH 8.5) containing 8 M urea. Disulfide bonds were reduced with 1 mM tris(2-carboxyethyl)phosphine (TCEP bond breaker, 0.5 M solution, Thermo Fisher, Waltham, MA, Cat No. 77720) at room temperature for 30 min. Cysteine alkylation was performed using 2.2 µL of 100 mM iodoacetamide, 30 minutes at room temperature protected from light, quenched with 10 mM dithiothreitol at room temperature for 15 min. The reduced and alkylated protein samples (∼30 µL) were digested overnight at 37°C in 8 M urea with 1 µg of endoproteinase Lys-C (2 µl of a 0.5 µg/µL stock; Roche, Basel, Switzerland), then diluted 1∶4 with 100 mM Tris, pH 8.5, incubated with trypsin (Sigma Chemical, St. Louis, MO; Cat No. T6567) (∼1∶4 enzyme ratio) for 24 h at 37°C, and acidified with aqueous 5% formic acid (3.3 µL) (Fluka, St. Louis, MO; Cat No. 56302). Peptides were extracted with Nutip carbon tips (Glygen, Columbia, MD; Cat No. NT3CAR) that were preconditioned by repetitive pipetting with 25 µL (x 3) of the peptide elution solvent (60% acetonitrile in 1% formic acid) followed by equilibration with 10 washes (25 µL) of extraction solvent (1% formic acid). Samples were loaded with 50 pipetting cycles. The tips were then washed four times with extraction solution. The peptides were recovered by 20 pipetting cycles with 25 µL of elution solution, followed by four washes (20 µL each) of elution solution. The extraction and wash solutions were combined in an autosampler vial (SunSri, Rockwood, TN; Cat No. 200 046) and dried in a SpeedVac (Thermo ScientificSavant). AS2 autosampler vial caps were from National Scientific (Rockwood, TN; Cat. No. 03-396AA).

### Comparative Nano-LC-MS

The complex mixtures of peptides from the endoprotease digests of the affinity-depleted CSF samples were reconstituted in 1% acetonitrile, 1% formic acid (35 µL) and analyzed using high-resolution nano-LC-MS on a linear quadrupole ion trap Fourier transform ion cyclotron mass spectrometer (LTQ-FTMS, Thermo Fisher). Liquid chromatography was performed on a nanoflow HPLC system (NanoLC-2Dplus™) interfaced to the mass spectrometer with a nanospray source (PicoView PV550; New Objective, Woburn, MA). The in-house packed LC column (Jupiter C12 Proteo, 4 µm particle size, 90 Å pore size [Phenomenex, Torrance, CA]) was equilibrated in 98% solvent A (aqueous 0.1% formic acid) and 2% of solvent B (acetonitrile containing 0.1% formic acid). The samples (10 µL) were injected using the autosampler at a flow rate of 1.0 µL/min followed by segmented linear gradient elution at 250 nL/min as follows: solvent B: isocratic, 0–2 min; 2% B to 40% B, 2–65 min; 40% to 80%, 65–70 min; isocratic at 80%, 70–72 min; 80% to 2%, 72–77; and isocratic at 2% B, 77–82 min. All samples were run in a continuous block, and their injections were randomly ordered to minimize the contribution of instrument bias (Fig. 1).

The mass spectrometer was operated in the data-dependent mode, in which only abundant ions are targeted for MS2. The survey scans (mass/charge ratio [*m/z] = *350–2000) (MS1) were acquired at high resolution (∼100,000 at *m/z* = 421.75). The 8 most abundant ions were isolated in the ion trap and fragmented after reaching a target value of ∼40,000. The MS2 isolation width was 2.5 Da, and the normalized collision energy was 35%. The following ion source parameters were used: capillary temperature 200°C, source voltage 3.5 kV, source current 100 µA, and the tube lens at 79 V. The data were acquired using *X*calibur, version 2.0.7 (Thermo Fisher, San Jose, CA).

### MS Data Processing and Protein Quantification

The LC-MS data processing pipeline is detailed in Fig. S3. Briefly, for relative peptide quantification, the LC-MS unprocessed files were imported into Rosetta Elucidator™ (Rosetta Biosoftware, ver 3.3) for *m/z* and retention time alignment of the peptide ion currents across the samples (pooled replicates and samples from individuals) using previously-described parameters [50] that are detailed in the legend of Fig. S3. The aligned, normalized peptide ion currents were annotated at the feature level within the alignment software by generating database search files (*.dta). The ion current signals from all charge states for each peptide were concatenated unique using a visual script within the software. The table of peptides and peptide intensities was exported in Excel *.csv format.

For protein identification, the LC-MS/MS files that were acquired using Xcalibur were processed using Mascot Distiller software (ver. 2.0.3) for the preparation of files for database searching. A UNIPROT human protein database (downloaded April 21, 2011, with 105,706 sequences) was searched using Mascot software (ver. 2.2.04) with the parameters given in the legend of Fig. S3. The protein database searches were further processed using Scaffold software (ver. 3.00.07) and the proteins were qualified using the Protein Prophet algorithm [51] with protein and peptide probabilities of 95% and 50%, respectively, as implemented in Scaffold [52]. All proteins were identified with a minimum of two peptides and at least one peptide with a probability score of>95%. The identified peptide sequences and mass spectrometric data that were used for protein identifications are given in Table S2.

The peptides were grouped as products from individual genes (Table S2). The gene-grouped and peptide intensity data were imported into DAnTE-R for statistical analysis [53], [54]. Only proteins represented by 2 or more annotated peptides were considered for subsequent data analyses (all annotated peptides are reported in Table S2). Annotated peptides with missing data (any intensity value = 0) from any sample (Table S3, fourth [‘ART-MET-EEP’] tab) were excluded from protein quantification analysis; no imputation algorithm was applied. For quantification of each protein (gene product), a mean value was calculated from all contributing annotated peptides. For the purpose of comparing two different strategies for quantifying proteins from peptide data, protein abundances and most other downstream statistical analyses were calculated twice: first, using all contributing annotated peptides, and second, using only the two most abundant peptides from each protein. Results from this second strategy are represented in supporting figures S5, S6, S7, S8, S9, S10 (numbered to correspond with ‘non-supporting’ figures representing the first strategy) and also in figures S12 and S13.

### Assessment of Variability/Reproducibility

**Figure 2:**
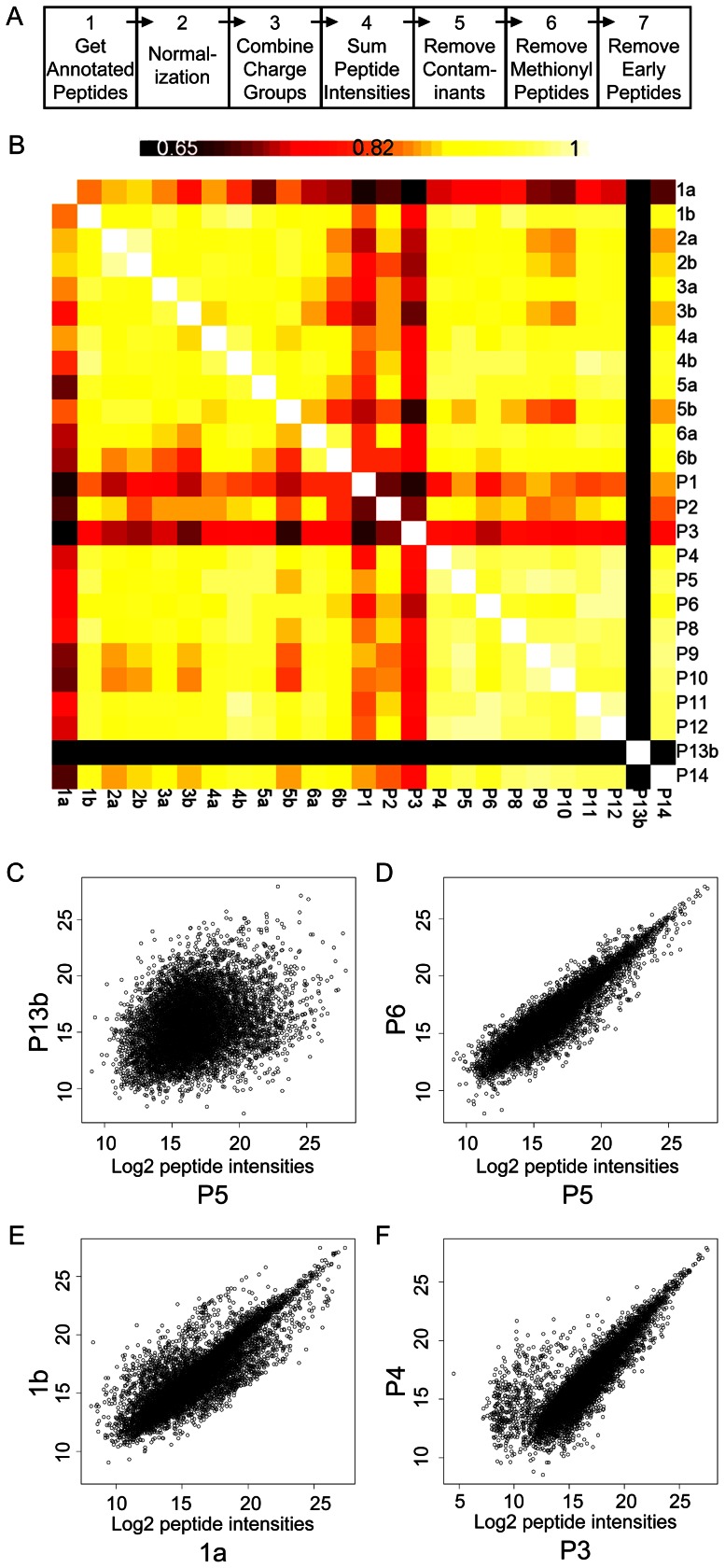
Data processing and symmetrical matrix for all sample pairwise comparisons of log_2_ annotated peptide intensities. **A.** Data processing steps in the visual script used within Rosetta Elucidator™ software. The intensities from the aligned peptide chromatograms were normalized and concatenated to sum signals from all charge states, isotope groups (Steps 1 through 3). Peak intensities of the isotope groups that were assigned to unique peptide sequences within each sample were summed (Step 4) for Pearson correlation coefficients (PCC). Common laboratory contaminants (e.g. keratin) and residual proteins from the MAF procedure (summarized in Table S4) were removed in Step 5. Methionine-containing peptides were removed in Step 6. ‘Early-eluting’ peptides were removed in Step 7. At each step the data were exported from the software and imported into DAnTE-R for further analysis using pair-wise correlations and scatter plots of the log_2_ transformed intensity data. **B**. Symmetrical matrix/non-clustering heatmap of PCC values from all pairwise comparisons from the annotated peptide intensity data (center), using a colorimetric scale ranging from black (low correlation, 0.65 and below), to red, orange, yellow, and white (high correlation, maximum 1). Self-pairwise comparisons, which yield a PCC equal to 1.0, appear as the diagonal of white squares. **C, D, E, F**. Representative scatter plots of all aligned charge group intensities from paired samples: P13b vs P5; P5 vs P6; 1a vs 1b; and P3 vs P4. Units of X- and Y-axes both represent log_2_ transformed charge group intensities.

**Figure 3:**
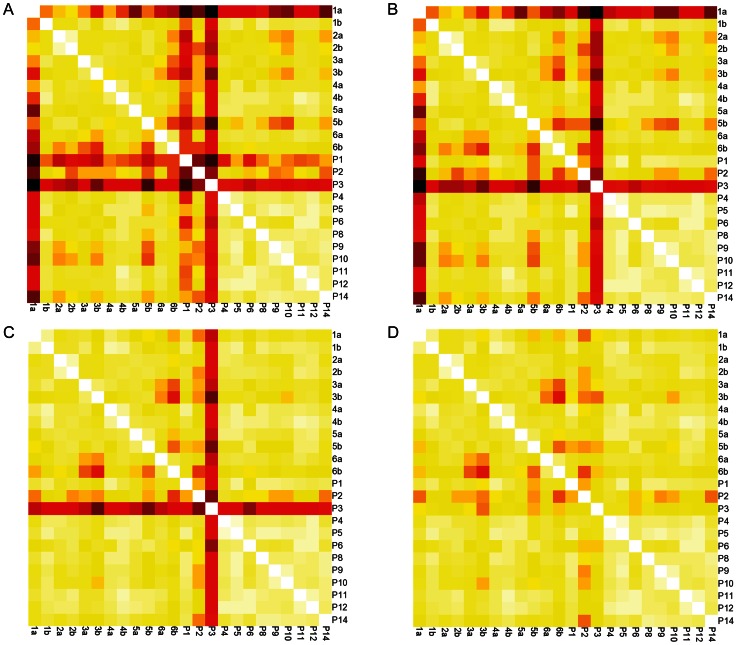
Correlations of pairwise sample comparisons after ‘removal’ of process contaminant, methionine-containing and early-eluting low-intensity peptides. **A.** Non clustering heat map of Pearson correlation values, after removal of pooled sample 13, calculated from all annotated peptides; **B.** Heat map after removal of contaminant and residual MAF-related peptides; **C.** Heat map after removal of Met-containing peptides from the contaminant-minus and MAF-minus set; **D**. Heat map after removal of low intensity, early eluting peptides (retention time = 20–42 min) from the contaminant/MAF/methionyl-peptide minus set. Colorimetric scale as depicted and described in Figure 2B. The ‘removed’ peptides and intensities are summarized in Table S4. Pearson correlation matrices are represented numerically in Table S5.

**Figure 4:**
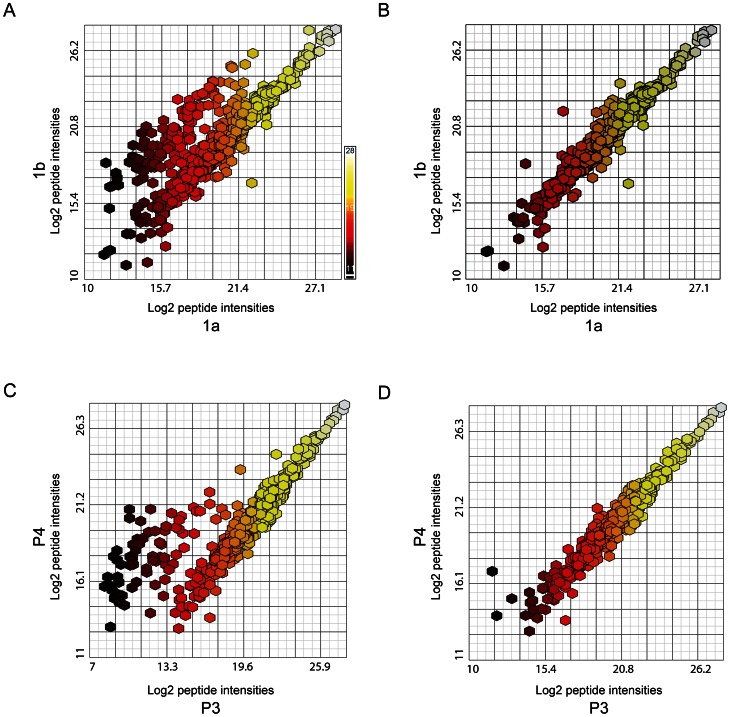
Annotated peptide intensity scatter-plots from select pairwise comparisons with removal of methionine-containing, early-eluting peptides. **A**. Scatter plot of annotated peptide intensities from individual sample aliquots 1a and 1b (process contaminant peptides already removed); **B.** Scatter plot of annotated peptide intensities from 1a and 1b after removal of Met-containing peptides from the contaminant-minus set; **C.** Scatter plot of annotated peptide intensities from pooled sample aliquots P3 and P4 (process contaminant and methionyl peptides already removed); **D**. Scatter plot of annotated peptide intensities from P3 and P4 after removal of early-eluting peptides. Colorimetric scale (in A) represents range of peptide intensities along x-axis, ranging from black (log_2_ intensity = 11), through red, orange and yellow to white (log_2_ intensity = 28). The ‘removed’ peptides and intensities are summarized in Table S4.

To estimate the overall correlation between pairs of CSF aliquots on a peptide level, Pearson correlation coefficients (PCC) were calculated for all aliquot pairs using the central tendency normalized data after a log_2_ transformation; several iterations of this procedure were performed, including all aligned charge groups (Fig. S11), or including only annotated peptides (Fig. 2B), with subsequent sequential selective exclusions of subsets of annotated peptides that were found to exhibit excessive variance (Table S5, Fig. 3, Fig. 4).

**Figure 5:**
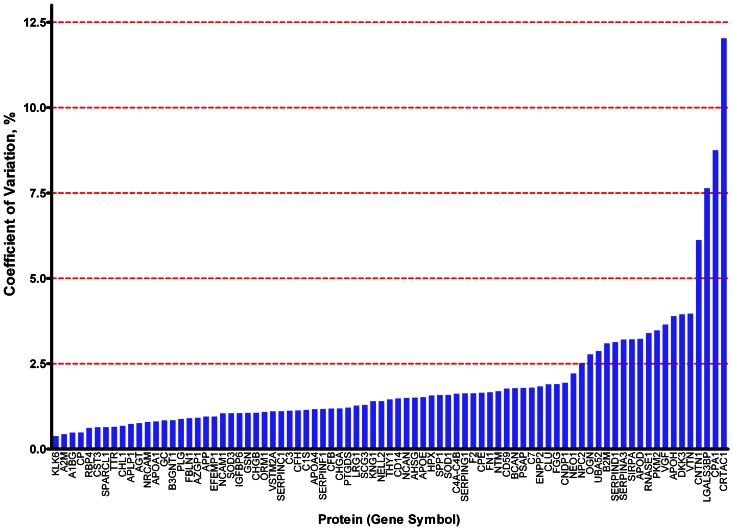
Coefficient of variation for each of 81 proteins. Coefficients of variation were calculated using values from all contributing peptides from pooled sample replicates. Numerical values in Table S6.

**Figure 6:**
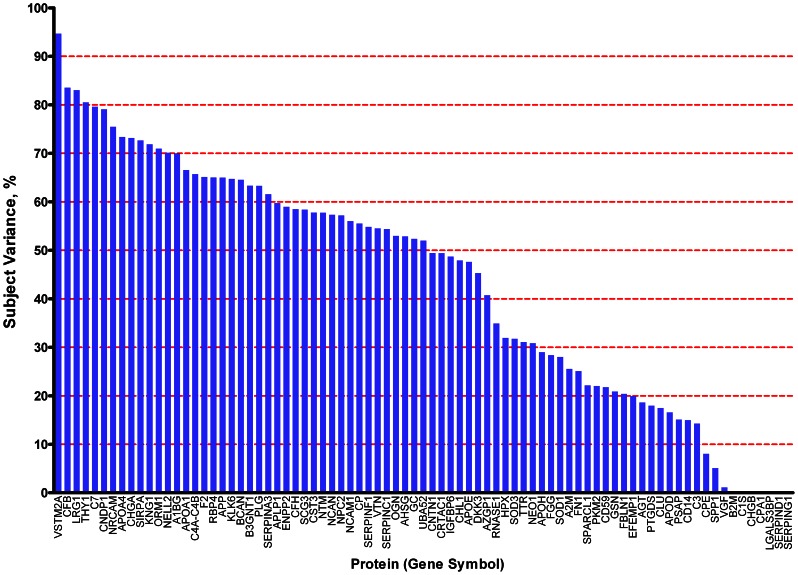
Subject variance for each of 81 proteins. Subject variance was calculated using values from all contributing peptides from paired replicates of individual samples. Numerical values in Table S6.

**Figure 7:**
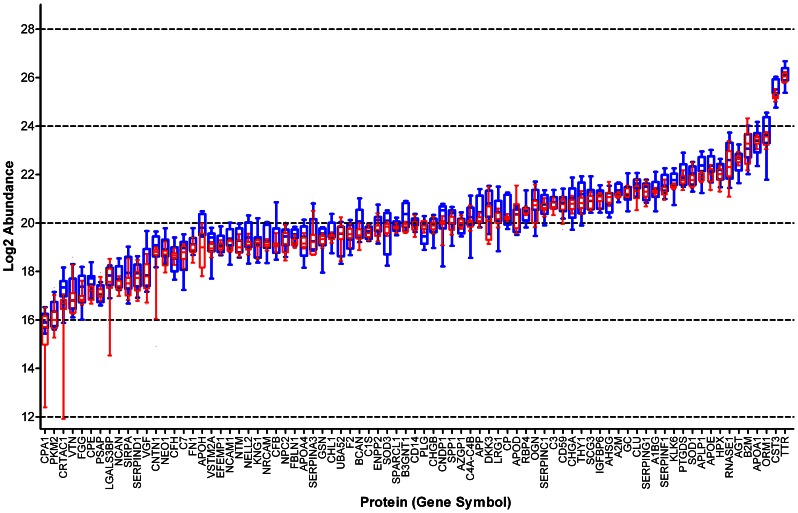
Biological variability and technical variability of 81 proteins, represented by all contributing peptides. Box and whiskers plot. All proteins ranked by mean of values from pooled sample replicates, left to right. Bar indicates median, box indicates 25^th^ to 75^th^ percentile, and whiskers indicate 10^th^ to 90^th^ percentile. Values derived from individual samples (n = 6; n = average of each pair of aliquots) are indicated in blue; those from pooled sample aliquots (n = 12), in red. Numerical values in Table S6.

Technical variability of each peptide/protein was estimated among replicates from the pooled sample by calculating the coefficient of variation (CV = standard deviation/absolute value of the mean). Inter-individual variability (subject variance) was estimated for each peptide/protein by comparing paired replicates from individuals using a random effects mixed model (SAS statistical analysis program, v9.2) (Table S6). The magnitude of each source of variability for each protein is depicted or reflected in figures 5, 6, 7.

Processing gradient (‘drift’) effects from MAF and LC-MS were evaluated at the protein level, with abundance values determined from the average of the two most abundant peptides. For each protein, abundance values from pooled sample replicates were plotted against run order during MAF (Fig. S12) or during LC/MS (Fig. S13). The effects of MAF run order and LC-MS run order (run orders depicted in Fig. 1) were each assessed independently by Pearson's correlation coefficient and Fisher's z transformation using PROC CORR in SAS v 9.1.3.

**Figure 8:**
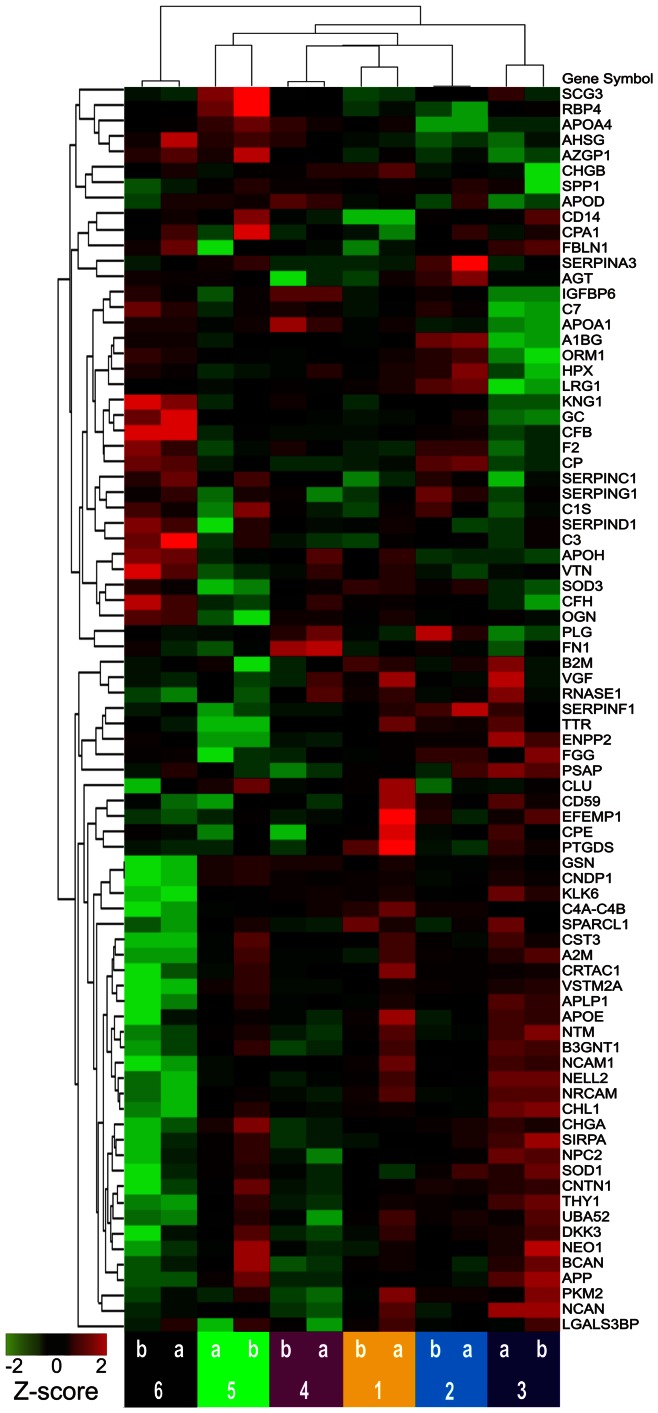
Unsupervised hierarchical clustering of duplicate samples from 6 cognitively normal individuals and all 81 proteins. All proteins were represented by two or more peptides. Data from all contributing peptides were used to calculate protein abundance. Samples are represented by columns as indicated by lettered and numbered colored blocks, below; proteins are represented by rows, as indicated by gene symbols on the right. Normalized protein abundance values (Z-scores) are indicated colorimetrically for each protein in each sample; red = high, black = mean value, green = low.

**Figure 9:**
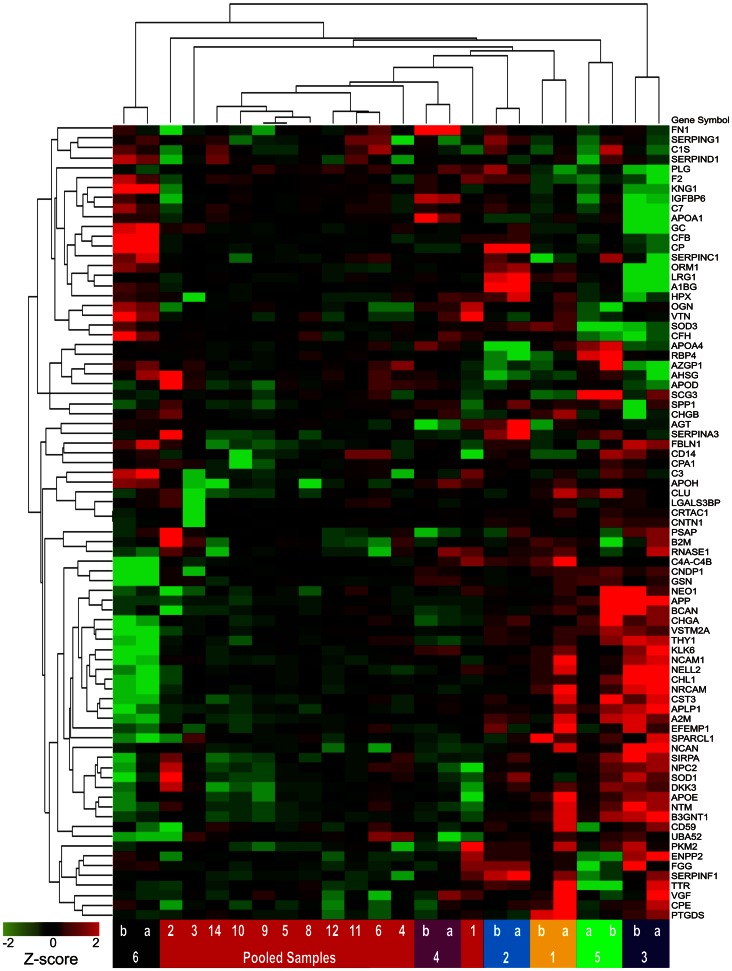
Unsupervised clustering of duplicate samples from 6 individuals, replicates of pooled CSF, and 81 proteins. Formatted as described for Fig. 8.

**Figure 10:**
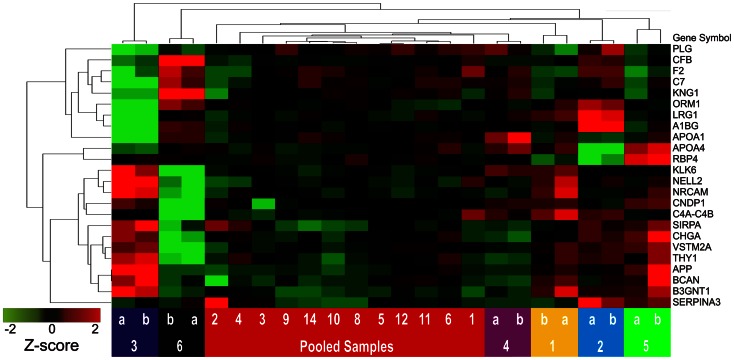
Unsupervised clustering of pooled and individual CSF replicates, limited to proteins with subject variance>60%. Formatted as described for Fig. 8.

To illustrate the potential of identified proteins as ensembles to segregate samples, unsupervised hierarchical clustering using Euclidean dissimilarity and average linkage analysis (Partek Genomics Suite v6.6 software) was applied: first, to values of all proteins for individual paired replicates (Figs.8, S8); second, to values of all proteins for all CSF aliquots (Figs.9, S9); and third, to all CSF aliquots, using only values of a subset of proteins that were selected on the basis of high inter-individual variability (high subject variance) (Fig. 10).

For the purpose of comparison, calculations of protein abundance, CV, subject variance, and unsupervised clustering were performed in two ways, using protein abundance values calculated from all contributing peptides for each protein (Figs. 5, 6, 7, 8, 9, 10, S10), or from only the two most abundant peptides from each protein (Figs. S5, S6, S7, S8, S9, S10, S12, S13).

## Results

The major goals of this study were: first, to identify the major reparable sources of technical variability within this complex proteomic workflow; second, to quantify the effect sizes of inter-individual and residual technical variability on measurements of protein abundances; third, to compare two strategies for protein quantification from peptide data generated using label free proteomics (mean of two most abundant peptides versus mean of all contributing peptides); and fourth, to evaluate the potential of the data generated by this proteomic workflow to segregate biological samples on the basis of desired biomarker characteristics.

### Technical Variance from Sample Processing

The CSF samples analyzed in this study included (1) fourteen aliquots of a pooled CSF sample derived from 100 individuals and (2) two aliquots from each of six cognitively normal individuals. The first group was selected to allow evaluation of technical variability associated with replicate processing of the same sample; the second, to allow assessment of inter-individual variability in a control group relevant to neurodegenerative biomarker discovery. To minimize variability associated with different reagents and instrument performance, all samples were processed continuously as a single block within each sequential experimental step: MAF (total processing time, 16.5 hours), endoprotease digestion/peptide preparation (∼3 days), and LC-MS (∼115 hours). The experimental design also included the randomization of sample order for MAF and again for LC-MS, to minimize the impact of any processing gradient (‘drift’) effects. Fig. 1 shows the two dimensional matrix of the run orders for MAF and LC-MS, showing, for example, that the pooled sample aliquot #2 (P2) was the fifth sample processed using MAF and the first to be analyzed in the LC-MS queue. As a quality assessment of MAF, the flow-through fractions were analyzed using 1D-gel electrophoresis (Fig. S1B, C). All 1D-gel image patterns were similar, except for pooled sample aliquot number 7 (P7), which had markedly less protein staining; P7 was not processed further.

After preparation of peptides, the remaining 25 samples were analyzed by LC-MS. All 25 samples showed similar total ion current profiles (Fig. S2) and intensities (within 20% of the mean), with the exception of pooled sample 13, which showed no MS signal from the initial data acquisition (P13a). A repeat LC-MS analysis of P13 (P13b) showed a total ion current (TIC) intensity of ∼50% of the mean TIC calculated from the other samples (Fig. S2, Table S1). The cause of the lower intensity in the peptide sample from P13 was not apparent. The high-resolution LC-MS analyses were time and *m/z* aligned, resulting in 37,629 aligned ion chromatograms that corresponded to 11,433 charge groups (Table S3 - fifth [‘ALL-PEP-INT’] tab). The frequency of missing data (charge groups with an intensity value of ‘0’) was very low for most samples; in contrast, sample P13b lacked measurable intensities for 24% of the charge groups (Table S3 - sixth [’MISSING DATA’] tab).

### Sources of Technical and Subject Variance at the Peptide Level

During the performance of each of these LC-MS analyses, the mass spectrometer was operating in data dependent mode and automatically isolated the most abundant ions for MS2. After processing, the MS2 data were then searched against the UNIPROT database for annotation (protein identification) as described in Fig. S3. The annotated features (n = 5630) were processed using a visual script that was executed with Rosetta Elucidator™ software (Fig. 2A). The annotated peak intensities were normalized (Fig. 2A, Step 2) as described in Fig. S3. Because some peptides were detected in more than one charge state, the individual charge states for each peptide were combined, yielding 1360 annotated isotope groups (Fig. 2A, Step 3). Additionally, when multiple isotope groups within an LC-MS analysis were found to be associated with a common peptide sequence, their peak intensities were summed, yielding 926 annotated, aligned peptide ion chromatograms across the 25 samples (Fig. 2A, Step 4) [55]. The data from each step of the sequential processing diagrammed in Fig. 2A were exported into a spread sheet (Table S3), grouped as gene names (HUGO convention) and imported into DAnTE-R software [52] for further analysis.

To evaluate variability in the proteomics workflow at the level of annotated peptides, non-clustering heat maps of Pearson correlation coefficients (PCC) were used. PCC values were generated using the log_2_ transformed peptide intensity data for all pair-wise sample comparisons (Table S5). Fig. 2B shows the symmetrical matrix of PCC values from all pairwise correlations among the pooled (P1-P14) and individual (1a–6b) sample aliquots, calculated from all *annotated* charge groups; for the purpose of comparison, Fig. S11 shows a similar matrix, calculated from all *aligned* charge groups. The diagonal white squares represent self-comparisons that yield a perfect correlation of 1.0 on a scale of 0.65 to 1.0; as shown by the color bar, imperfect correlations are represented by increased shading that ranges from yellow to orange to red, with the lowest values appearing as black squares.

These Pearson correlation heat maps corroborate the ion current results; pairwise comparisons of the pooled sample with the lowest ion current (P13) (Table S1) yielded uniformly low PCC's, represented by intense black bars in Fig. 2B and Fig. S11, with samples P13b and P5 showing the poorest correlation (PCC ∼0.3) (Table S5). The corresponding P13 vs. P5 scatter plot (Fig. 2C) appears as a wide, homogeneous cloud of log_2_ transformed aligned charge group intensities. In contrast, the scatter plot of two pooled samples with a high PCC (P5 vs. P6, PCC = 0.928), represented in Fig. 2D, appears predominantly as a tight linear cluster. We concluded from these data that TIC values below ∼25% of the mean value of the data set are not normalized by the algorithm described in Materials and Methods. Therefore, sample P13 was removed from the set in subsequent analyses.

The heat maps in Fig. 2B and Fig. S11 also revealed that, among the paired aliquots from individual samples, 1a vs. 1b gave a relatively poor pairwise correlation (PCC = 0.8377). Inspection of the corresponding scatter plot (Fig. 2E) showed many data points above and below the primary linear cluster. Similarly, the heat map in Fig. 2B showed uniformly poor correlations for P3, and the scatter plot comparing pooled sample aliquots P3 vs. P4 (PCC = 0.804) displayed a group of points with higher values in the P4 sample than in P3 (Fig. 2F). Many of the other aliquots from the pooled sample showed much higher correlation values; all P4–P12 pairwise comparisons showed PCC's = ∼0.9–0.95. However, P1–P3 displayed lower correlations, with PCC's = ∼0.75–0.85 (Table S4). The overall variability observed among these individual and pooled sample aliquots did not correlate with the order in which samples were processed by MAF or LC-MS analysis; for example, samples P2 and P3 (poor correlation) were processed 5^th^ and 6^th^ in the MAF order and in relatively short succession (1^st^ and 5^th^) for LC-MS, whereas samples P4 and P9 (high correlation) were widely separated in the MAF order (9^th^ and 24^th^) and in the LC-MS order (2^nd^ and 18^th^) (Fig. 1).

To investigate the source(s) of this variability, we examined the sequences of peptides that were poorly correlated in pairwise comparisons and considered the proteins from which they were derived. Examination of the identified proteins (Table S3) revealed that some samples contained “process contaminant” proteins (keratins [e.g. KRT1, KRT2, and KRT10] and residual proteins from the MAF [e.g. albumin and transferrin]). The peptides (103 total; Table S4, first [‘ART’] tab) from these proteins were removed from subsequent analyses of all samples (Table S3) and a new correlation heat map was generated (Fig. 3B). Although this exclusion of keratins and MAF proteins improved the correlations between selected pairwise comparisons (particularly those involving P1), it did not improve all poor correlations (for example, those involving aliquot 1a) (Fig. 3B). Further examination of the scatter plot of annotated peptide intensities for one of these unchanged pairwise comparisons (sample 1a vs. 1b) showed a distinct cloud of points with lower values in sample 1a (Fig. 4A). All the sequences of these discordant peptides contained at least one methionine residue (145 total; Table S4, second [‘MET’] tab). Removal of these methionyl peptides from subsequent PCC calculations (compare Fig. 4B to 4A) resulted in an increased correlation coefficient for 1a vs. 1b (0.967 vs. 0.835) (Table S5; also, compare Fig. 3C to Fig. 3B), and a modest increase in the correlation coefficient for 5a and 5b. However, the removal of methionyl peptides did not significantly alter the poorer correlation between P3 and the other pooled samples (Fig. 3C). A scatter plot of the peptide intensities of P3 vs. P4 showed a discrete group of peptide intensities that were lower in aliquot P3 than in P4 (Fig. 4C). Upon inspection, these discordant peptides (69 total; Table S4, third [‘P3’] tab) were found to have early elution times (20–42 min) during liquid chromatography. The markedly decreased ion current for P3 compared to P4 during this early elution window is shown in Fig. S4, panels A and B. As an example of the marked reduction in signal for P3 during this elution time window, all sample intensities for an early-eluting NCAM-1 peptide (DGEQIEQEEDDEK, elution time = 40.2 min) are shown in Fig. S4, panels C and D. Scatter plots of the peptide intensities of P3 vs. P4 before and after removal of the early eluting peptides (Fig. 4C and 4D, respectively) show improved correlation after exclusion. In kind, a new non-clustering heat map of PCC values, created after excluding these early-eluting peptides from all PCC calculations, showed improved correlations and a narrower range of PCC values between P3 and all other pooled samples (compare Fig. 3D to Fig. 3C). Although the reason for the low intensities of early-eluting peptides found in sample P3 remains unclear and does not appear to affect other samples substantially, these early-eluting peptides were excluded from subsequent analyses of all samples.

### Quantification, Technical Variance and Subject Variance at the Protein Level

To evaluate the reproducibility of this proteomics method and the overall differences between individual samples on a *protein* level, annotated peptides were grouped according to their gene product (protein) of origin. After ‘process contaminant’ peptides, methionyl peptides and ‘early-eluting’ peptides were excluded (Fig. 2A, steps 5,6,7), 81 proteins were represented by more than one of the remaining 609 peptides (Table S3, fourth [‘ART-MET-EEP’] tab). Each of these proteins was then quantified in each sample by calculating the mean of the values of its representative peptides. To compare two alternative strategies for protein quantification, these calculations were performed in two ways: using values from all contributing peptides (Figs. 5, 6, 7, 8, 9, 10); or using values from only the two most abundant peptides (Figs. S5, S6, S7, S8, S9, S10, S12, S13).

To estimate the residual technical variability with which this technique quantifies each of these proteins, coefficients of variation (CV) were calculated from replicates of the pooled CSF sample; to determine the inter-individual variability of each of these proteins within a group of cognitively normal individuals, subject variance was calculated from the paired aliquots from individual samples. These assessments of variability are important for biomarker studies because, hypothetical proteins with relatively higher CVs and/or relatively high subject variance among controls will be more likely than those with lower CVs and lower subject variances to show overlapping ranges between diseased and control cohorts, given equivalent fold-changes associated with disease. Greater overlaps mean lower sensitivity and specificity and, therefore, relatively less potential for a candidate to serve as an effective disease biomarker. Remarkably, almost all 81 proteins showed CVs<5% (Fig. 5; Figs. S5, S10; Table S6); this range of values is comparable or superior to those of other techniques (e.g., ELISA) that are commonly applied to quantify proteins in solution. Not unexpectedly, the range of subject variances was broad across the 81 proteins (Fig. 6, Table S6). Nevertheless, for the vast majority of these proteins, the technical variability and inter-individual variability were very modest in comparison to the median values of the proteins in question (Fig. 7, Fig. S7).

### Processing Gradient (‘Drift’) Effect at the Protein Level

To evaluate whether MAF or LC-MS processing order might introduce processing gradient (‘drift’) effects at the level of protein quantification, Pearson correlation coefficients were calculated for each of the 81 proteins, using values calculated from the two most abundant peptides from each of the pooled sample replicates. With a significance threshold of p = 0.05, only four such correlations in each category might be expected by chance, alone. However, these analyses identified six proteins (C1S, CHRB, FN1, NELL2, SPP1, UBA52) with significant positive or negative correlations with MAF (Fig. S12) and 17 proteins (APOA1, APP, B2M, C1s, C4A-C4B, CFH, CLU, FBLN1, GC, IGFBP6, NCAN, NEO-1, PLG, PTGDS, RNASEI, SERPINA3, VSTM2A [though VSTMA2 95% CI spans zero]) with significant positive or negative correlations with LC-MS (Fig. S13). Nevertheless, the magnitudes of these gradient effects were modest relative to median protein abundance values (depicted in Figs. S12 and S13 as individual graphs), as evidenced by uniformly low CVs, discussed above.

### Classification of Samples

To assess the capacity of these 81 proteins to segregate the different CSF samples, unsupervised hierarchical clustering was performed. Without any prior selection for proteins of interest, when all contributing peptides were used for protein quantification, this method accurately segregated duplicate aliquots of individual samples (Fig. 8) but did not completely segregate samples when pooled sample aliquots were included (Fig. 9). When a similar clustering analysis was performed, including only 24 candidate biomarkers selected for their high (>60%) subject variance (greater potential to distinguish individual samples), perfect segregation of pooled and individual samples was achieved (Fig. 10).

### Protein Quantification Using All or Two Most Abundant Peptides

A direct comparison of two strategies for quantifying proteins on the basis of peptide-level data showed the following: when only the two most abundant peptides, rather than all contributing peptides, were used, values of abundance were slightly higher, values of CV were marginally smaller (more so for some proteins of lower abundance), values of subject variance were slightly higher, and unsupervised clustering was less complete (Figs. S5, S6, S7, S8, S9, S10). Nevertheless, the two approaches yielded generally comparable results.

## Discussion

This study evaluates the technical merits and potential of a non-biased proteomics technique, label-free quantitative LC-MS/MS, for CSF biomarker discovery. In so doing, it describes analytical methods that can be applied to identify variability arising from technical sources across the workflow, from sample procurement through LC-MS. It also compares two approaches to quantify proteins (gene products) from peptide data (mean of all contributing peptides versus mean of two peptides with greatest intensities). Further, it presents baseline statistical data for 81 relatively abundant CSF proteins within a neurologically normal (‘control’) group of older individuals, calculates the technical variability and gradient effects observed in the measurement of these proteins within multiple replicates of a pooled CSF sample, and illustrates how selective subsets of these proteins might be used to classify samples that differ by biological phenotype. Thus, it provides a framework for future experiments that will evaluate CSF samples from individuals with neurological diseases, in search of relevant biomarkers.

### Alternative and Evolving Strategies for Peptide Identification

Recently, other reports have also described the use of this technique for CSF proteome characterization and biomarker discovery. Impressively, some have identified and quantified hundreds or thousands of proteins in a single CSF sample [56], [57], by referencing the unique LC retention times and m/z values of the extracted ion chromatograms of peptides to an annotated ‘library’ of retention times and m/z values compiled from previous MS/MS analyses of similar CSF preparations (accurate mass and time tag [AMT] strategy). For this current experiment, no such annotated AMT library was available. Instead, MS1 and MS2 scanning were performed simultaneously to enable the annotation of peptides in real time. This approach availed the identification of a comparatively smaller number of proteins, but was wholly adequate for the purpose of this experiment, which was not intended to discover novel rare CSF proteins or to maximize the number of proteins identified. As it happens, recent instrumentation advances during the short interval since this experiment was conducted now allow for the annotation of many more peptides in real time, effectively increasing the sensitivity of simultaneous scanning; these changes have reduced the popularity of the more laborious AMT approach. Regardless, even without such advances, either approach is likely to identify promising candidate biomarkers. Indeed, even in this limited experiment, in which tandem mass spectrometry was triggered solely on the basis of relatively high peptide abundance, a comparatively modest list of 81 proteins generated sufficient diagnostic potential to allow perfect segregation of ‘individual’ and ‘pooled’ sample replicates with a much smaller subset of 24 selected proteins (compare Fig. 9 and Fig. 10). It is also encouraging that many of the 81 proteins have already been reported as potential biomarkers for AD by multiple independent groups [2], [20], [28], [31], [36], [58]–[61]. Indeed, because none of these previously reported candidate biomarkers have been vetted sufficiently to be applied in clinical trials, they will have to be studied further: individually and in combination; in larger cohorts and in different diseases. Thus, particularly with recent advances, this technique is well suited for application in future AD research studies to facilitate the validation of promising biomarkers.

### Limitations of Quantitative Label-Free LC-MS/MS

In spite of its many advantages, quantitative label-free LC-MS/MS is not without limitations [41]. The use of endoproteolytic peptides as protein surrogates eliminates isoform information encoded in the CSF proteome; protein molecules that are modified by physiological or pathological proteolytic cleavage, differential mRNA splicing, or isolated post-translational modifications may be difficult or impossible to detect in the company of ‘full-length’ or ‘unmodified’ proteins encoded by the same gene; such information may be detectable using other techniques such as 2D gel analysis. Nevertheless, quantitative label-free LC-MS/MS is faster, less labor-intensive, more reproducible, more amenable to automation and peptide/protein identification, and more accommodating of larger sample sets than most complementary gel-based techniques.

A related challenge involves the reliability with which quantification at the peptide level can be “rolled up” to the protein level. Fortunately, statistical approaches have been developed to address this problem and are available in the open source proteomics platform DAnTE (http://omics.pnnl.gov/software/) [54]. As demonstrated by this study, when DAnTE is applied to multiple replicates of a pooled sample, quantification at the protein level is highly reproducible (CV<5% for most of the proteins analyzed). When all contributing peptides are used for protein abundance calculations, there appears to be a trend of increasing CV associated with decreasing abundance (Fig. S10), but this trend is diminished when only the top two peptides for each protein are considered. It remains to be seen in future studies whether comparably low CVs will be achieved for less abundant proteins. Nevertheless, these results suggest that this technique is highly quantitative, with technical reproducibility similar to that of ELISA and other, more ‘conventional’ methods that are commonly used to quantify protein concentrations in fluids. It is also conceivable that reference samples, containing known amounts of proteins of interest, could be processed and analyzed with patient CSF samples to provide for absolute, and not just relative, quantification of protein abundance.

### Subject Variance/Inter-individual Variability

Another issue regarding candidate biomarker discovery that is independent of this technique, but is addressed in this study and has strong implications for the potential of a protein to serve as a biomarker, is that of inter-individual variability. Hypothetically, if two biomarkers show identical fold-changes between a group of control samples and another group of samples representing a disease state, the biomarker with lower inter-individual variability (tighter clustering about the median) in each group will show less overlap between groups and will yield higher sensitivity and specificity for diagnosis than the biomarker with greater inter-individual variability. In this study, the distributions of values from cognitively normal control samples for each of these 81 proteins (Fig. 7) are generally quite narrow about the median. Of course, the amplitude of fold-change for the disease state in question is also a very important driving variable in determining the sensitivity and specificity of a biomarker. Indeed, even proteins with relatively broad ranges of concentration among cognitively normal individuals (e.g. chromogranin A, NrCAM) can show potential for diagnosing AD and control samples as part of a biomarker panel [20]. Nevertheless, most proteomic fold-changes reported to date for CSF biomarkers have been rather modest (<1.5 fold) and would show far greater biomarker utility in a background of far lower inter-individual variability.

### Process Gradient Effect – Randomization Required

A separate issue that confronts this technique involves processing gradient or ‘drift’ effects that may occur in the performance of MAF and LC-MS over the course of an experiment. In this experiment, processing gradient (‘drift’) effects appear to have influenced the values of multiple proteins, as demonstrated by statistically significant correlations of pooled sample replicate values with MAF or LC-MS run order (Figs. S12 and S13). However, these influences were small relative to mean protein abundances, as evidenced by low CV's among the 81 proteins (Fig. S5). Nevertheless, this observation of ‘drift’ does warrant sample randomization and the insertion/distribution of multiple technical control sample replicates throughout the processing order in future applications of this technique, as was practiced in this experiment.

### Classification of Samples

A final point of discussion addresses the purpose and the implications of the hierarchical clustering analyses performed in this study. Such analyses are employed here to illustrate the potential of this technique to measure ensembles of proteins that can classify samples according to desired characteristics. In most biomarker discovery studies, such clustering analyses would be preceded by a selection process in which candidate biomarkers are vetted on the basis of statistical association with a diagnosis of interest (as in [20]). In the current study, because the samples analyzed do not strictly represent two (or more) different disease states, the proteins were evaluated, instead, for their ability to segregate CSF from different sources (6 individuals and a pooled sample). In this context, each source of CSF, represented by multiple replicates, may be considered a surrogate for a different clinical state; subject variance may be considered analogous to fold-changes between different clinical conditions. The improved clustering of samples in Fig. 10 relative to Fig. 9 reflects a selection of biomarkers that can distinguish these individual samples. Thus, although this study does not directly illustrate the potential of these proteins as biomarkers for neurological diseases, it does suggest that this technique could perform such a task, when applied to CSF samples from appropriate cohorts.

### Summary

Though it is not free from some of the limitations of ‘bottom-up’ proteomics approaches, label-free LC-MS/MS is a powerful quantitative technique with a high capacity for multiplexing (simultaneously measuring multiple biomarkers), a modest per-analyte sample volume, and very low technical variability, provided that analytical procedures are used to identify variations in peptide intensities that can be ascribed to correctable technical sources. Therefore, quantitative label-free LC-MS/MS shows great promise as a tool for the discovery of CSF proteins that can serve as biomarkers for the diagnosis, staging, prognosis and monitoring of neurological diseases.

## Supporting Information

Figure S1 Instrument configuration for multi-affinity fractionation (A) and 1D-SDS-PAGE of individual (B) and pooled (C) samples. Molecular weight markers indicated by black bars to right of gel images represent (from top, in kD): 250, 150, 100, 75, 50, 37, 25, 20, 15, 10.
(TIF)

Figure S2 Total ion current chromatograms from LTQ-FTMS analysis of ‘flow-through’ from multi-affinity fractionation of CSF samples. Numerical values of total ion currents in Table S1.
(TIF)

Figure S3 Data processing for quantitative, label-free proteomics analysis of CSF. In step 1, the unprocessed LC-MS/MS files that were acquired using X-calibur (Thermo Fisher, ver. 2.0.7) were analyzed using Mascot Distiller software (ver 2.0.3) for preparation of files for database searching. After creating the *.mgf files, the MS2 data were searched using MASCOT (ver. 2.2.04) [62] against the UNIPROT human protein database (downloaded April 21, 2011, with 105,706 sequences), allowing for up to 4 missed cleavages (Step 2). The MS1 and MS2 mass tolerances were set at 20 ppm and 0.8 Da, respectively. Carbamidomethyl was set as a fixed modification for Cys residues and Met residue oxidation was allowed as a variable modification. The protein database searches were further analyzed using Scaffold software (ver. 3.00.07) (Step 3) and the proteins were identified using the Protein Prophet algorithm [51] with protein and peptide probabilities of 95% and 50%, respectively (Step 4), as implemented in Scaffold [52]. All proteins were identified with a minimum of two peptides and at least one peptide with a probability score of >95%. The identified proteins and supporting mass spectrometric data are given in Table S2. For relative protein quantification, the same set of unprocessed LC-MS files was imported into Rosetta Elucidator™ (Rosetta Biosoftware, ver 3.3) and the peptide ion chromatograms were aligned and mean normalized using the following modification of the previously described parameters [50]: Peak time score minimum = 0.5; peak m/z score minimum = 0.5; Scan width of m/z = 350–1400; LC time range of 30–140 min; intensity scaling based on the mean intensity of all features (Step 5). The aligned peptide ion currents (PIC's) were annotated within the software by generating *.dta files (Step 6) and searching the UNIPROT human database using MASCOT as described above (Step 7). The ion current signals from all charge states for each peptide were concatenated unique using a visual script within the software. The table of peptides and peptide intensities was exported in Excel *.csv format (Step 8). The peptides were grouped as individual genes (Table S2) (Step 9). The gene-grouped peptide intensity data were imported into DAnTE-R for statistical analysis [53], [54] (Step 10).
(TIF)

Figure S4 Total ion current of early-eluting peptides (samples P3 and P4); ion intensity of NCAM-1 peptide. The ion traces for the initial phase of the gradient elution of peptides from samples P4 (**A**) and P3 (**B**) are shown. The peak height intensities for an ‘early-eluting’ NCAM-1 peptide (DGEGIEQEEDDEK) for all samples are graphed (**C**) and listed (**D**) for all samples.
(TIF)

Figure S5 Coefficients of variation for 81 proteins, calculated using only the two most abundant peptides. Numerical values in Table S6.
(TIF)

Figure S6 Subject variance for each of 81 proteins, calculated using only the two most abundant peptides. Calculated using values from all paired individual sample replicates. Numerical values in Table S6.
(TIF)

Figure S7 Biological variability and technical variability of 81 proteins, represented by the two most abundant peptides. Box and whiskers plot, as described for Fig. 7. Numerical values in Table S6.
(TIF)

Figure S8 Unsupervised clustering of individual sample replicates, 81 proteins quantified using the two most abundant peptides. Formatted as in Fig. 8.
(TIF)

Figure S9 Unsupervised clustering of individual and pooled replicates; 81 proteins quantified using two most abundant peptides. Formatted as in Fig. 8.
(TIF)

Figure S10 Relationship of coefficient of variation and protein abundance, comparing two alternative strategies for protein quantification. Abundances (median values among pooled sample replicates) of 81 proteins were calculated from the mean of all peptide intensities (blue open circles) or from the mean of peptide intensities from the two most abundant peptides (red open squares). Abundance values are plotted against CVs that were calculated from pooled sample replicates, as described in Materials and Methods.
(TIF)

Figure S11 Symmetrical matrix of Pearson correlation analyses: all aligned charge groups (11,433), all pairwise sample comparisons. Formatted as in Fig. 2. Peptide intensity features were time and *m/z* aligned as described in Materials and Methods. The MS data were processed through Steps 1–3 (Fig. 2A). Sample P7, which did not pass 1D-gel-electrophoresis QC analysis (Fig. S1), was excluded.
(TIF)

Figure S12 Influence of multi-affinity fractionation (MAF) run order on protein abundance measurements. For each of 81 proteins (organized alphabetically by gene symbol of origin), log2 transformed abundance values (calculated from the mean of the two most abundant peptides) for each of the pooled sample replicates are plotted versus MAF run order. Pearson correlation coefficients and statistics (Fisher's z transformation) are listed for each protein.
(PDF)

Figure S13 Influence of LC-MS run order on protein abundance measurements. For each of 81 proteins (organized alphabetically by gene symbol of origin), log2 transformed abundance values (calculated from the mean of the two most abundant peptides) for each of the pooled sample replicates are plotted versus LC-MS run order. Pearson correlation coefficients and statistics (Fisher's z transformation) are listed for each protein.
(PDF)

Table S1 Total ion currents from LC-MS analysis of CSF. Duplicate aliquots from 6 individual samples and 14 aliquots from a pooled sample were assayed, as described under ‘Materials and methods’.
(XLSX)

Table S2 Mass spectrometry, peptide data, database parameters and search results. Scaffold Proteome Software (v.3.1.4.1) was used to display MASCOT search results. Protein probability filter was set at 95%, peptide probability at 50%, and a minimum of 1 peptide required. Protein and peptide false discovery rate were determined by Scaffold using the probabilistic method used by the Trans-proteomic pipeline (see http://proteome-software.wikispaces.com/FAQ-Statistics).
(XLSX)

Table S3 Amplitude-normalized peptide intensites from m/z and time alignment of ion chromatograms; contaminants, outliers sequentially removed. The gene grouped peptides are listed under the first (‘ALL-PEP’) tab. The intensities after removal of process contaminant, Met-containing, and early-eluting peptides are given under the second (‘ART’), third (‘ART-MET’), and fourth (‘ART-MET-EEP’) tabs, respectively. Intensity values for all 11,433 charge groups are given under the fifth (‘ALL-PEP-INT’) tab. Missing data values (number and percent of charge group values = 0, in ‘ALL-PEP-INT’ tab) for each sample are given under the sixth (‘MISSING DATA’) tab.
(XLSX)

Table S4 Contaminant and outlier peptides. Artifactual peptides from contamination or incomplete removal of targeted proteins during MAF are given under the first (‘ART’) tab. Methionine-containing peptides that were discordant in the scatter plot analysis of ‘duplicate’ samples (e.g. samples 1a and 1b, Fig. 4A, 4B) are shown under the second (‘MET’) tab. Early eluting peptides from pooled sample 3 (P3) (Fig. 4C, 4D and Fig. S4) are given under the third (‘P3’) tab.
(XLSX)

Table S5 Pearson correlation coefficients for all pairwise comparisons from the log_2_ transformed intensities of annotated peptides. The correlation coefficients from all pairwise comparisons of the log_2_ transformed peptide intensities are given under the first (‘ALL-PEPS’) tab. The matrices of correlation coefficients after removal of peptides from ‘artifactual’ proteins and after removal of ‘artifactual’ and methionine-containing peptides are given under the second (‘ART’) and third (‘ART-MET’) tabs, respectively.
(XLSX)

Table S6 Statistical analysis of protein data for CSF sample replicates. The first (‘SUBJ’) tab includes mean, median, standard deviation and subject variance for all 81 proteins among individual CSF sample aliquots. The second (‘POOLS’) tab includes mean, median, standard deviation and coefficient of variation for all 81 proteins among pooled sample aliquots. Values derived from protein abundances calculated using all contributing peptides are labeled ‘ALLPEP’ (columns B–E); those derived from abundances calculated using only the top two peptides are labeled ‘TOP2’ (columns F–I).
(XLSX)
