# Supplementary figures and images for: Quantitative Label-Free Proteomics for Discovery of Biomarkers in Cerebrospinal Fluid: Assessment of Technical and Inter-Individual Variation
Source: PLoS One. 2013 May 20;8(5):e64314. doi: 10.1371/journal.pone.0064314 (PMC3659127; doi:10.1371/journal.pone.0064314)

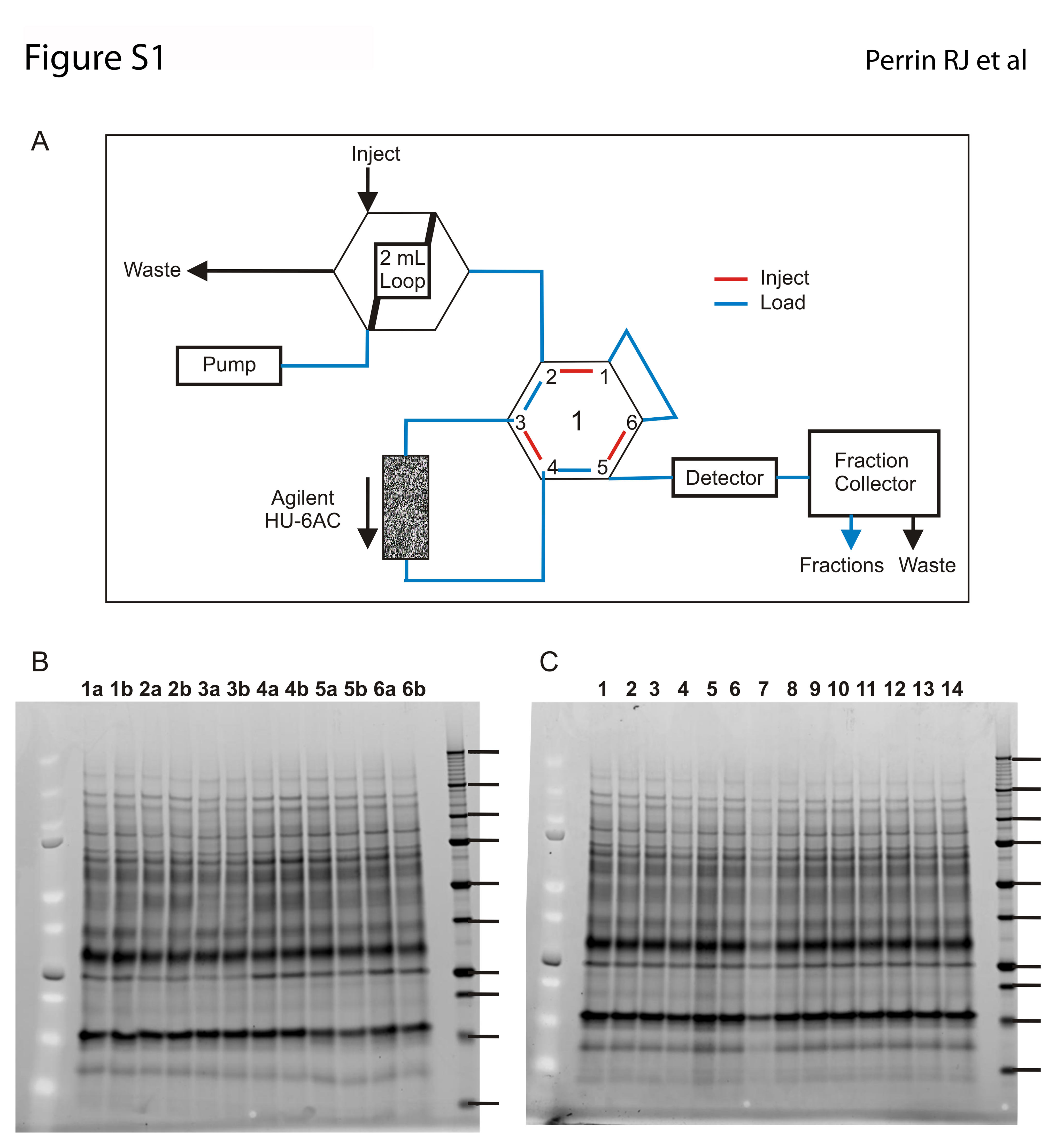

Supplement: Figure S1 — Instrument configuration for multi-affinity fractionation (A) and 1D-SDS-PAGE of individual (B) and pooled (C) samples. Molecular weight markers indicated by black bars to right of gel images represent (from top, in kD): 250, 150, 100, 75, 50, 37, 25, 20, 15, 10. (TIF) [file pone.0064314.s001.tif]

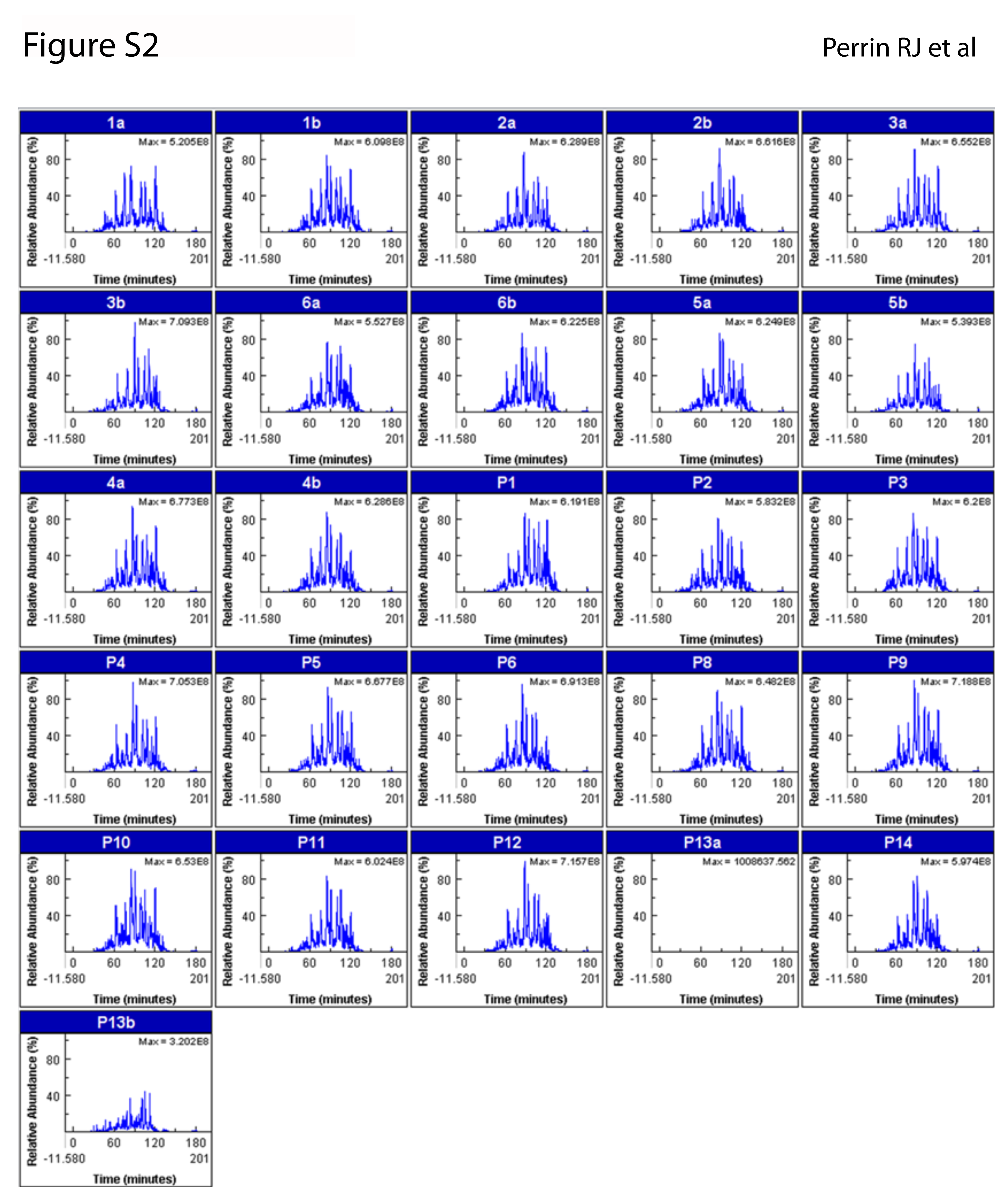

Supplement: Figure S2 — Total ion current chromatograms from LTQ-FTMS analysis of ‘flow-through’ from multi-affinity fractionation of CSF samples. Numerical values of total ion currents in Table S1. (TIF) [file pone.0064314.s002.tif]

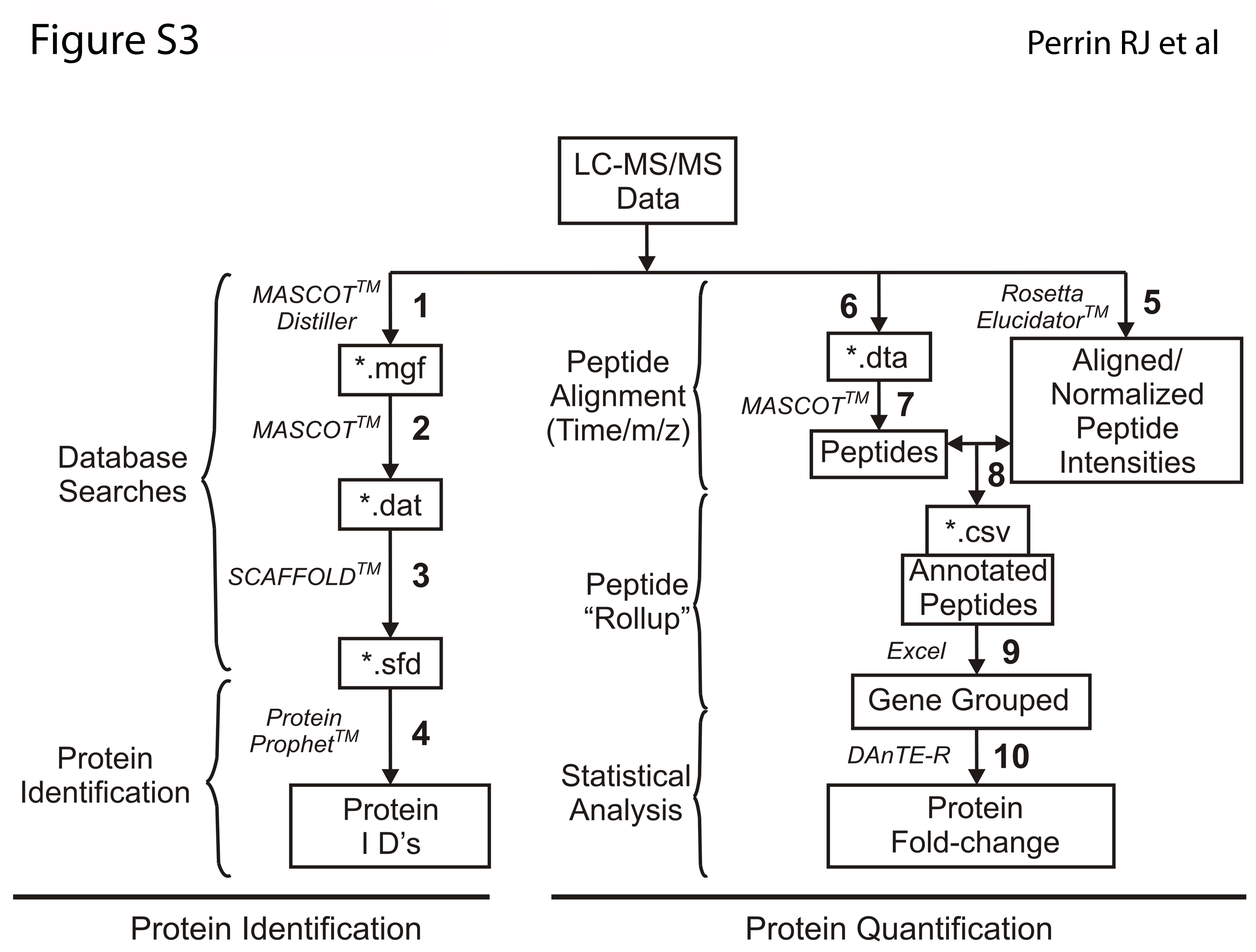

Supplement: Figure S3 — Data processing for quantitative, label-free proteomics analysis of CSF. In step 1, the unprocessed LC-MS/MS files that were acquired using X-calibur (Thermo Fisher, ver. 2.0.7) were analyzed using Mascot Distiller software (ver 2.0.3) for preparation of files for database searching. After creating the *.mgf files, the MS2 data were searched using MASCOT (ver. 2.2.04) [62] against the UNIPROT human protein database (downloaded April 21, 2011, with 105,706 sequences), allowing for up to 4 missed cleavages (Step 2). The MS1 and MS2 mass tolerances were set at 20 ppm and 0.8 Da, respectively. Carbamidomethyl was set as a fixed modification for Cys residues and Met residue oxidation was allowed as a variable modification. The protein database searches were further analyzed using Scaffold software (ver. 3.00.07) (Step 3) and the proteins were identified using the Protein Prophet algorithm [51] with protein and peptide probabilities of 95% and 50%, respectively (Step 4), as implemented in Scaffold [52]. All proteins were identified with a minimum of two peptides and at least one peptide with a probability score of >95%. The identified proteins and supporting mass spectrometric data are given in Table S2. For relative protein quantification, the same set of unprocessed LC-MS files was imported into Rosetta Elucidator™ (Rosetta Biosoftware, ver 3.3) and the peptide ion chromatograms were aligned and mean normalized using the following modification of the previously described parameters [50]: Peak time score minimum = 0.5; peak m/z score minimum = 0.5; Scan width of m/z = 350–1400; LC time range of 30–140 min; intensity scaling based on the mean intensity of all features (Step 5). The aligned peptide ion currents (PIC's) were annotated within the software by generating *.dta files (Step 6) and searching the UNIPROT human database using MASCOT as described above (Step 7). The ion current signals from all charge states for each peptide were concatenated unique using a visual s [file pone.0064314.s003.tif]

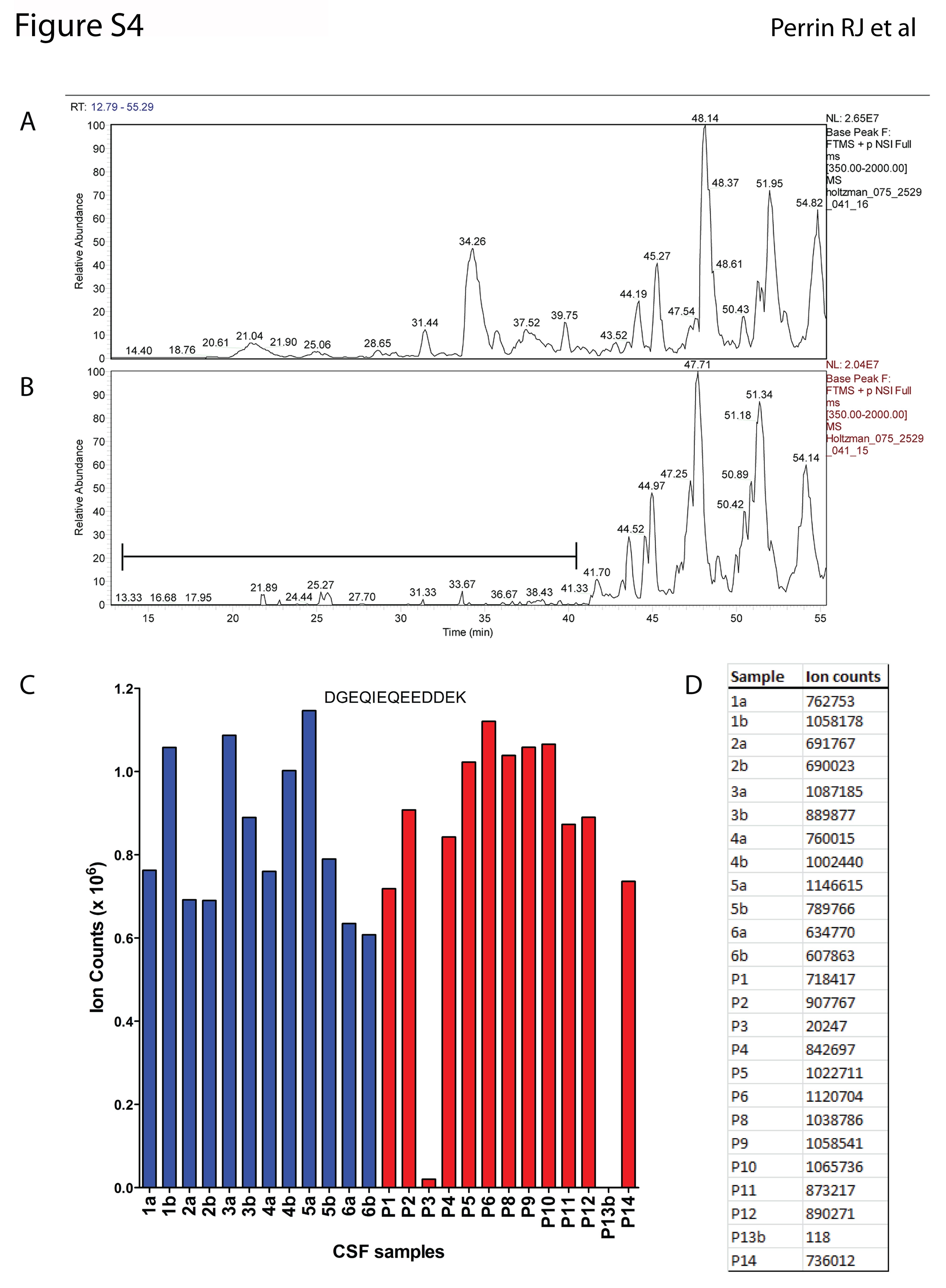

Supplement: Figure S4 — Total ion current of early-eluting peptides (samples P3 and P4); ion intensity of NCAM-1 peptide. The ion traces for the initial phase of the gradient elution of peptides from samples P4 (A) and P3 (B) are shown. The peak height intensities for an ‘early-eluting’ NCAM-1 peptide (DGEGIEQEEDDEK) for all samples are graphed (C) and listed (D) for all samples. (TIF) [file pone.0064314.s004.tif]

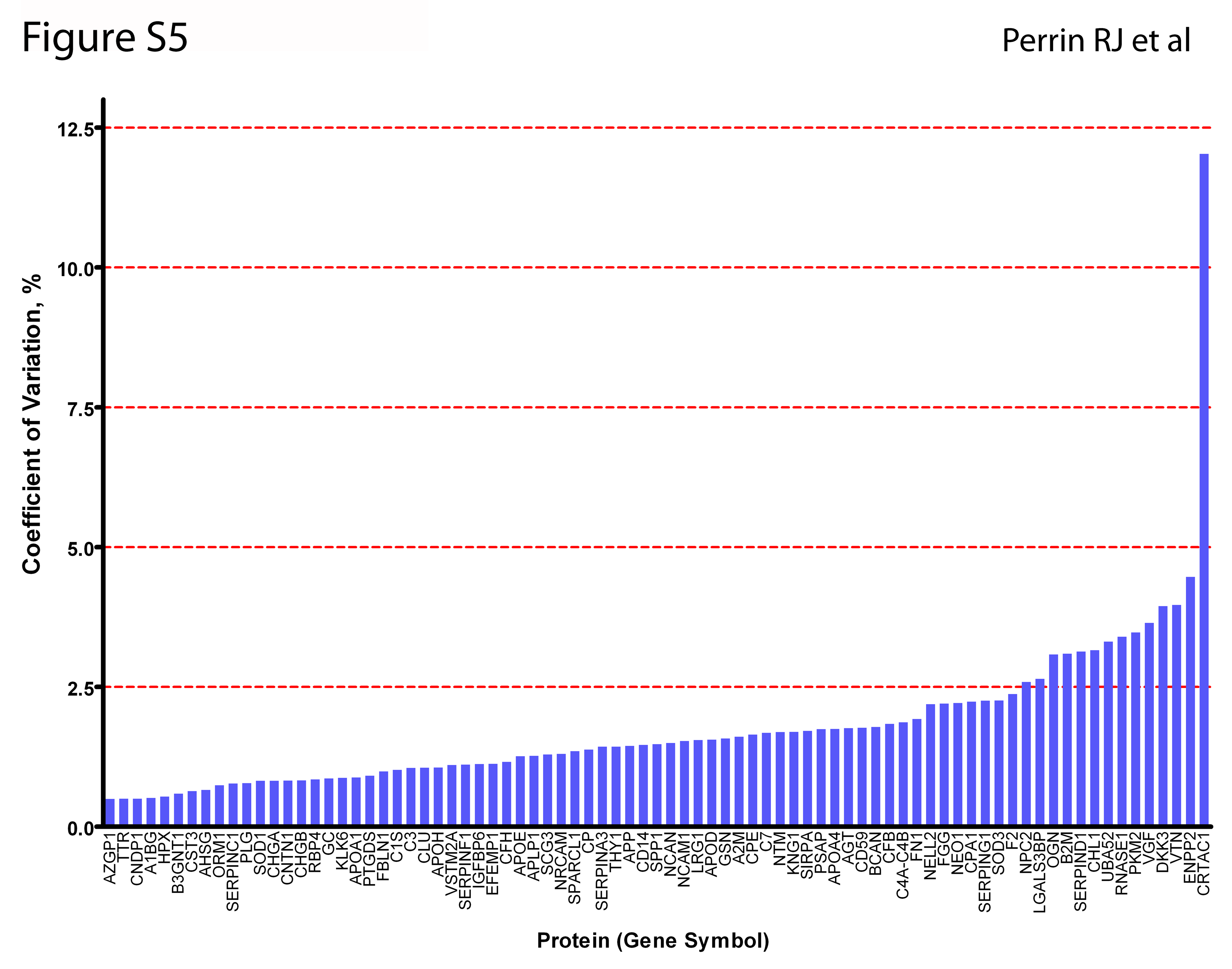

Supplement: Figure S5 — Coefficients of variation for 81 proteins, calculated using only the two most abundant peptides. Numerical values in Table S6. (TIF) [file pone.0064314.s005.tif]

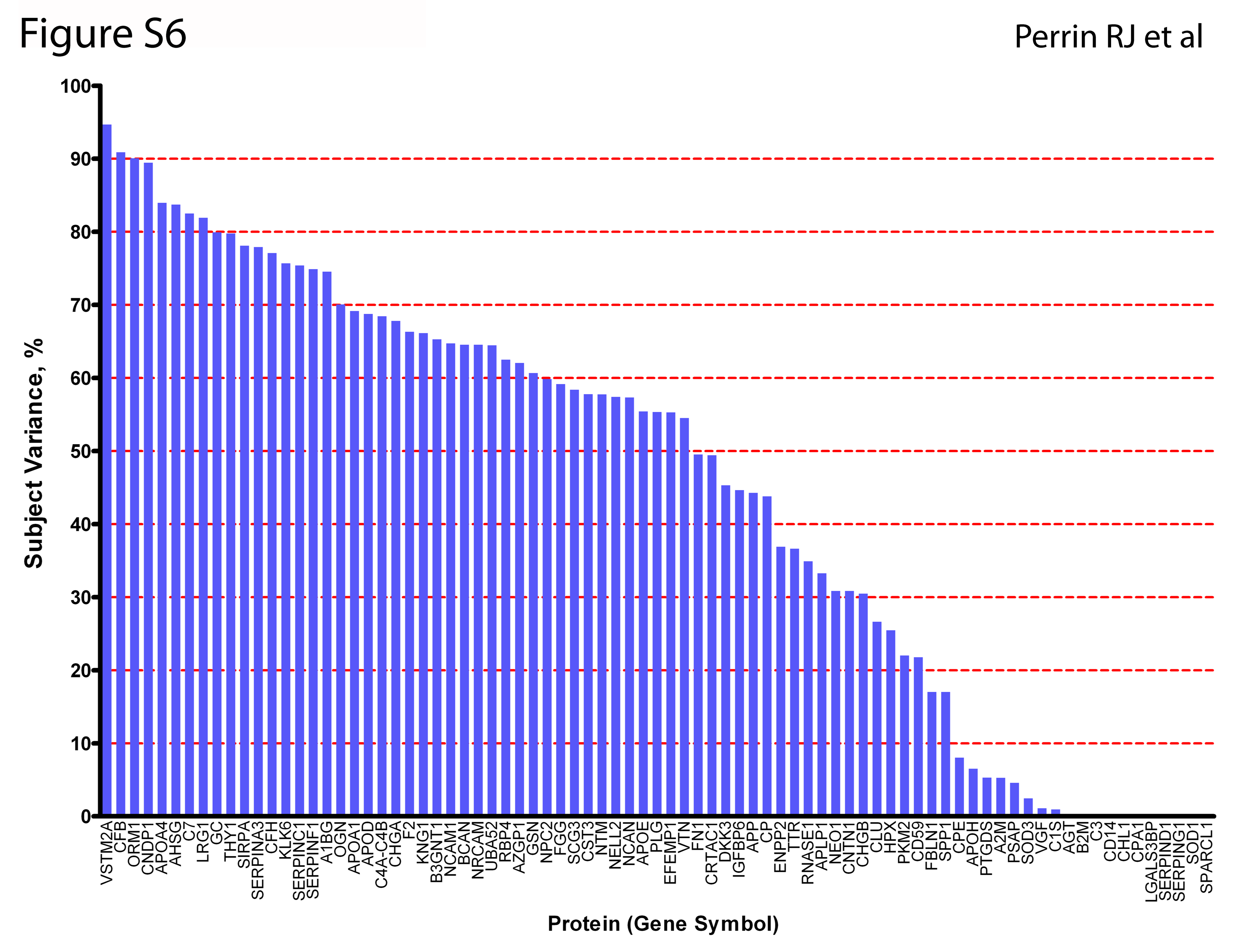

Supplement: Figure S6 — Subject variance for each of 81 proteins, calculated using only the two most abundant peptides. Calculated using values from all paired individual sample replicates. Numerical values in Table S6. (TIF) [file pone.0064314.s006.tif]

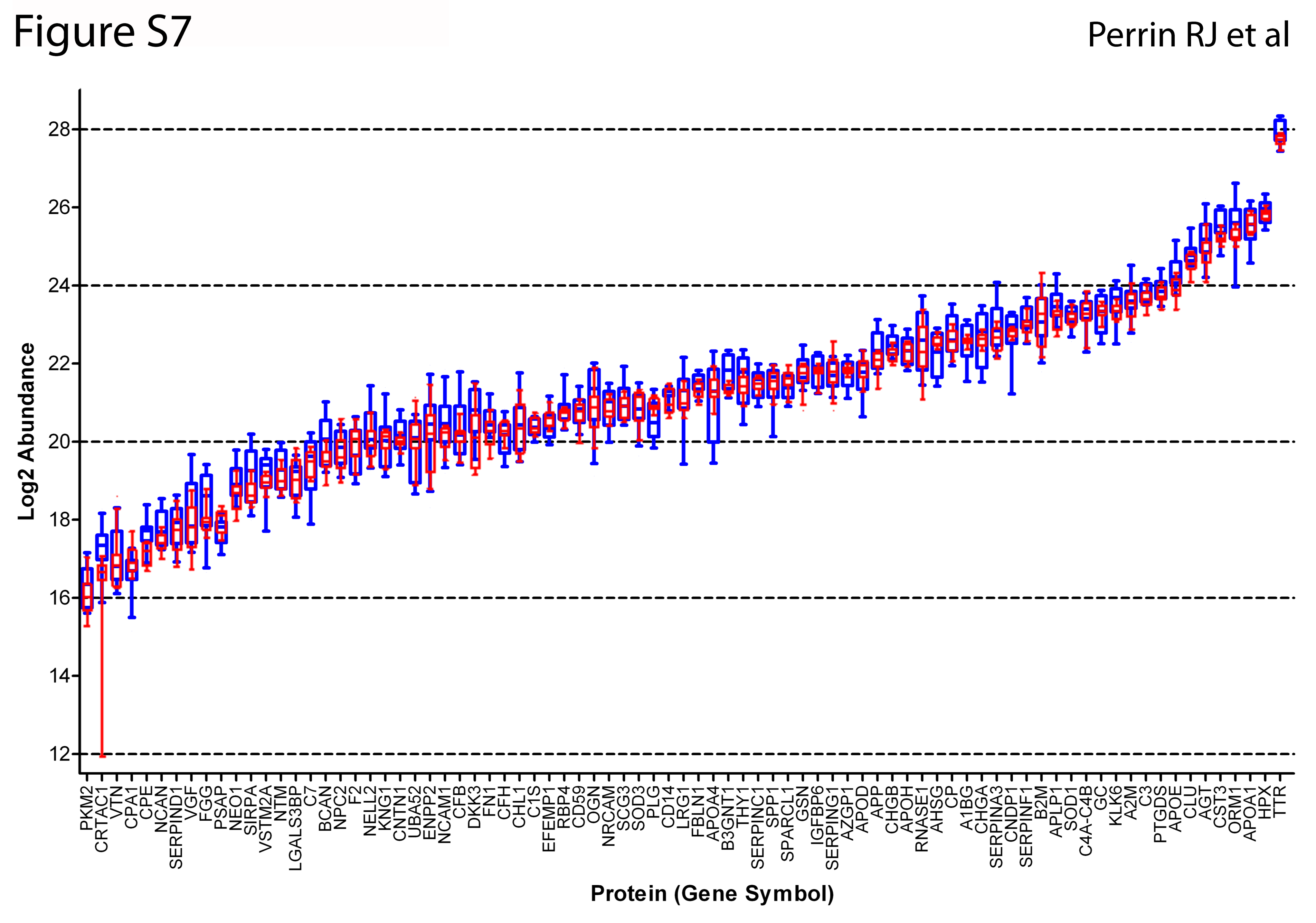

Supplement: Figure S7 — Biological variability and technical variability of 81 proteins, represented by the two most abundant peptides. Box and whiskers plot, as described for Fig. 7. Numerical values in Table S6. (TIF) [file pone.0064314.s007.tif]

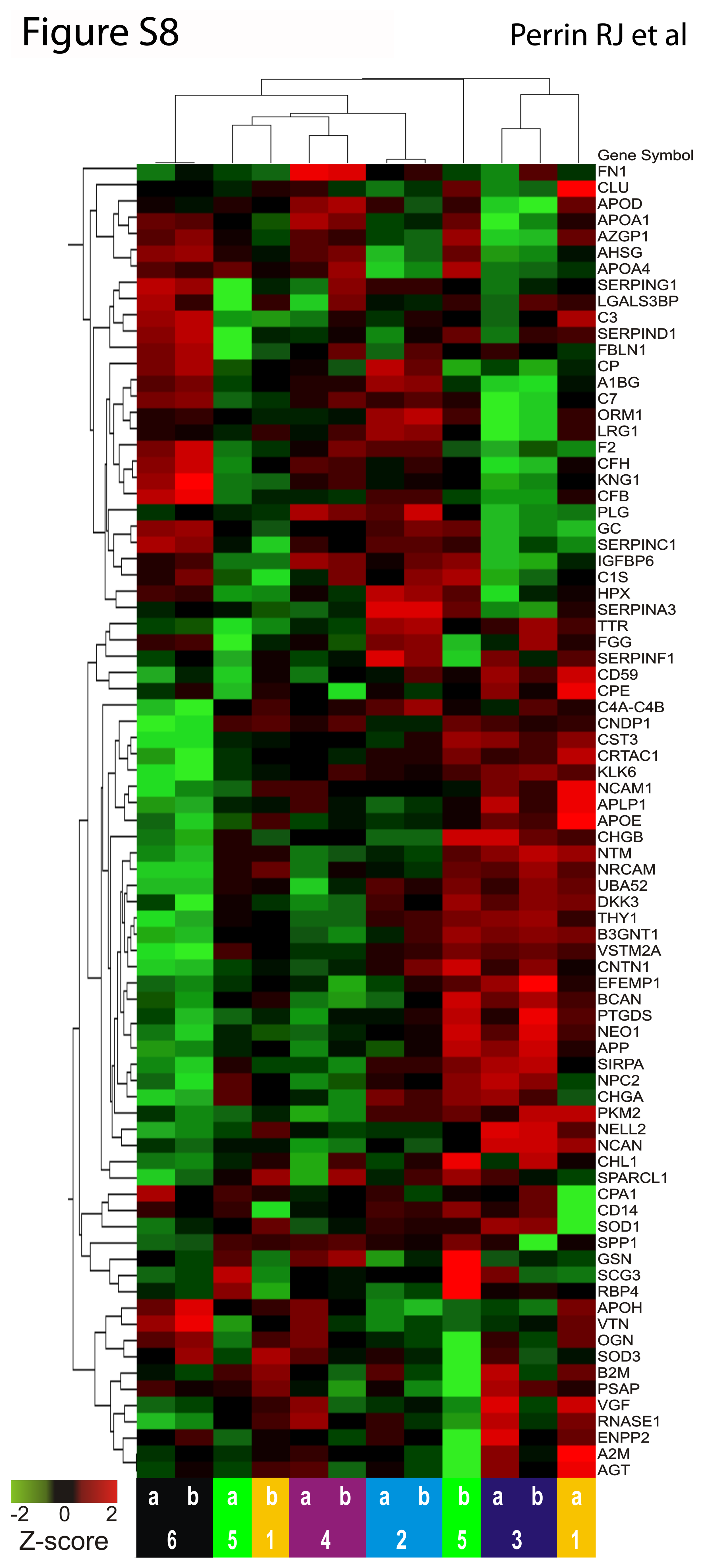

Supplement: Figure S8 — Unsupervised clustering of individual sample replicates, 81 proteins quantified using the two most abundant peptides. Formatted as in Fig. 8. (TIF) [file pone.0064314.s008.tif]

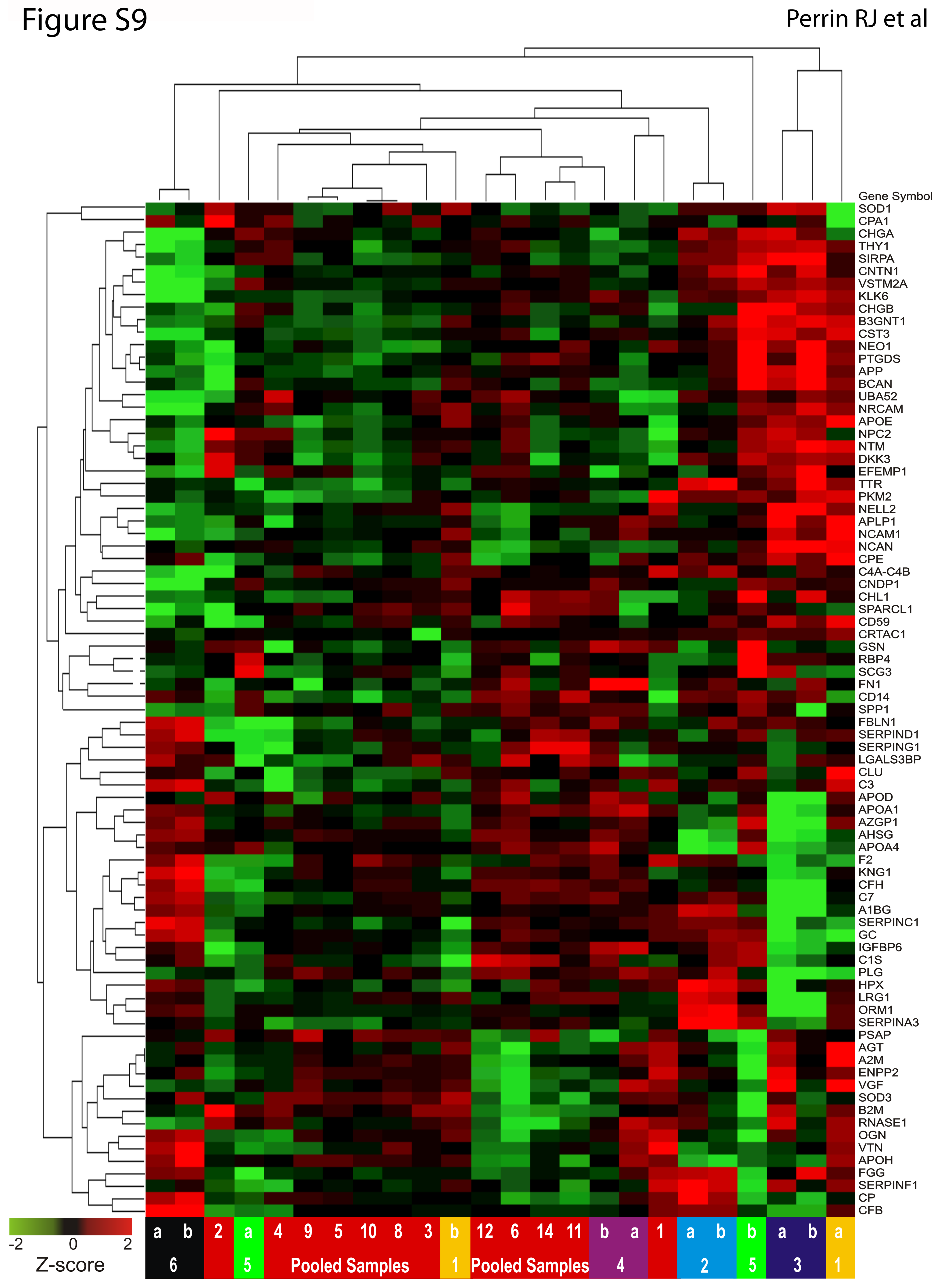

Supplement: Figure S9 — Unsupervised clustering of individual and pooled replicates; 81 proteins quantified using two most abundant peptides. Formatted as in Fig. 8. (TIF) [file pone.0064314.s009.tif]

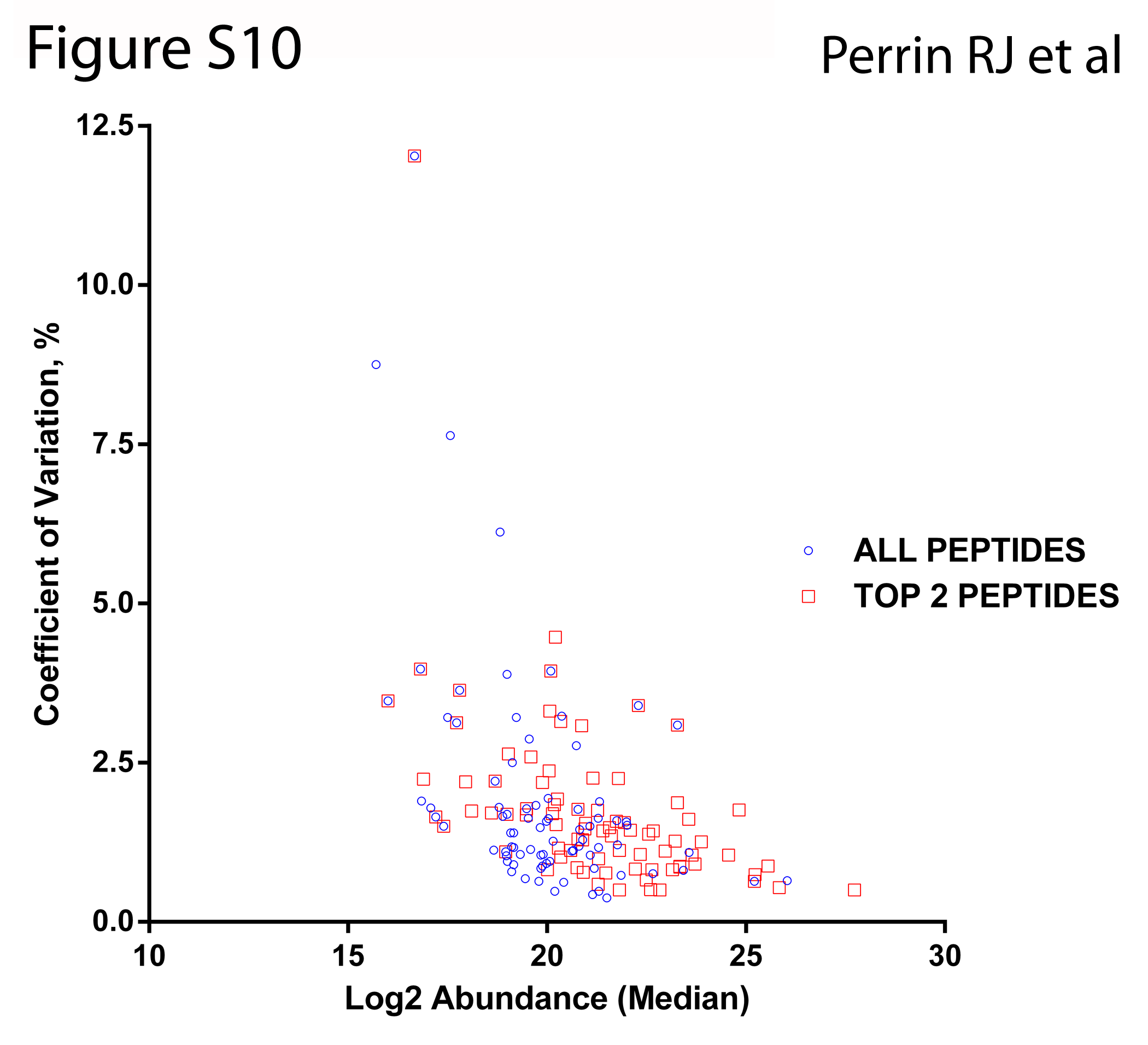

Supplement: Figure S10 — Relationship of coefficient of variation and protein abundance, comparing two alternative strategies for protein quantification. Abundances (median values among pooled sample replicates) of 81 proteins were calculated from the mean of all peptide intensities (blue open circles) or from the mean of peptide intensities from the two most abundant peptides (red open squares). Abundance values are plotted against CVs that were calculated from pooled sample replicates, as described in Materials and Methods. (TIF) [file pone.0064314.s010.tif]

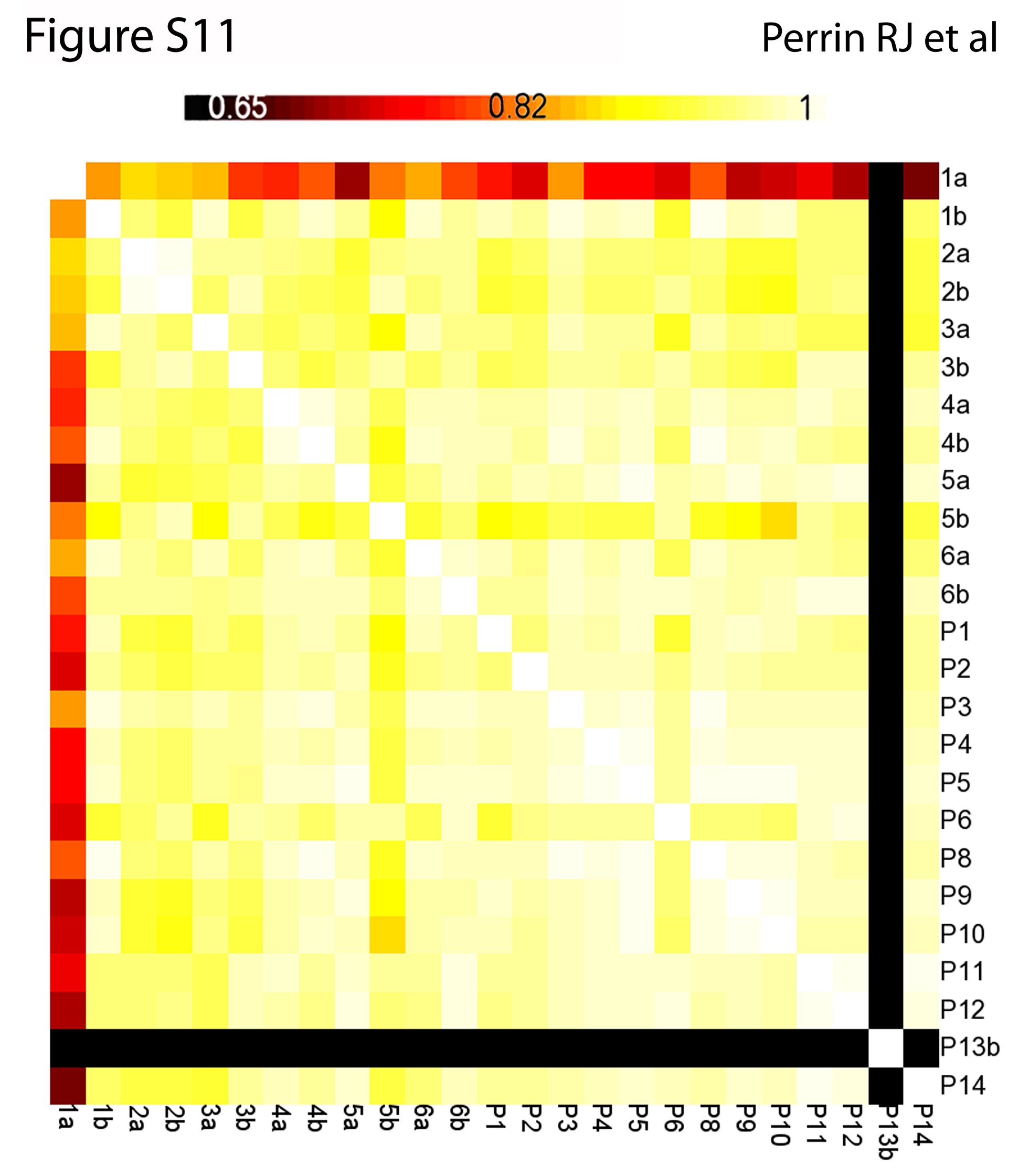

Supplement: Figure S11 — Symmetrical matrix of Pearson correlation analyses: all aligned charge groups (11,433), all pairwise sample comparisons. Formatted as in Fig. 2. Peptide intensity features were time and m/z aligned as described in Materials and Methods. The MS data were processed through Steps 1–3 (Fig. 2A). Sample P7, which did not pass 1D-gel-electrophoresis QC analysis (Fig. S1), was excluded. (TIF) [file pone.0064314.s011.tif]
