# Supplementary material for: Quantitative Label-Free Proteomics for Discovery of Biomarkers in Cerebrospinal Fluid: Assessment of Technical and Inter-Individual Variation
Source: PLoS One. 2013 May 20;8(5):e64314. doi: 10.1371/journal.pone.0064314 (PMC3659127; doi:10.1371/journal.pone.0064314)

Correlation C_pooltop2lg2mean_tp2 symbol MAF

The CORR Procedure

Gene_Symbol=A1BG

**2 Variables:**	top2lg2mean MAF

Simple Statistics						
**Variable**	**N**	**Mean**	**Std Dev**	**Sum**	**Minimum**	**Maximum**
**top2lg2mean**	12	22.57461	0.11608	270.89531	22.29520	22.78045
**MAF**	12	13.83333	7.10740	166.00000	5.00000	25.00000

Pearson Correlation Coefficients, N = 12<br>Prob > |r| under H0: Rho=0		
	**top2lg2mean**	**MAF**
**top2lg2mean**	1.00000	-0.01778<br>0.9563
**MAF**	-0.01778<br>0.9563	1.00000

Pearson Correlation Statistics (Fisher's z Transformation)									
**Variable**	**With Variable**	**N**	**Sample Correlation**	**Fisher's z**	**Bias Adjustment**	**Correlation Estimate**	**95% Confidence Limits**		**p Value for H0:Rho=0**
**top2lg2mean**	**MAF**	12	-0.01778	-0.01779	-0.0008084	-0.01698	-0.585176	0.562405	0.9574

Correlation C_pooltop2lg2mean_tp2 symbol MAF

The CORR Procedure

Gene_Symbol=A1BG

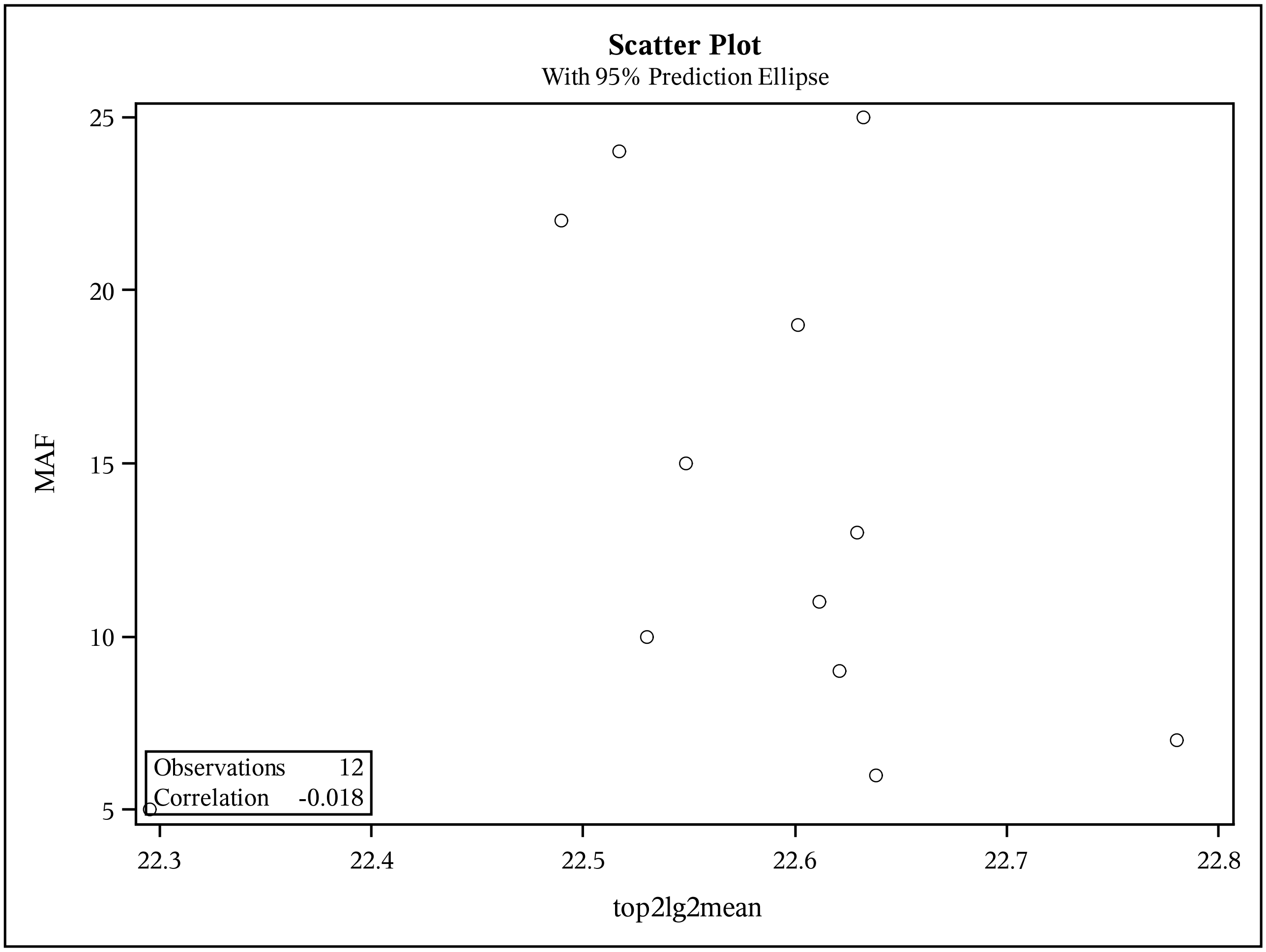

Correlation C_pooltop2lg2mean_tp2 symbol MAF

The CORR Procedure

Gene_Symbol=A2M

**2 Variables:**	top2lg2mean MAF

Simple Statistics						
**Variable**	**N**	**Mean**	**Std Dev**	**Sum**	**Minimum**	**Maximum**
**top2lg2mean**	12	23.51360	0.37790	282.16326	22.83730	24.15903
**MAF**	12	13.83333	7.10740	166.00000	5.00000	25.00000

Pearson Correlation Coefficients, N = 12<br>Prob > |r| under H0: Rho=0		
	**top2lg2mean**	**MAF**
**top2lg2mean**	1.00000	-0.35552<br>0.2567
**MAF**	-0.35552<br>0.2567	1.00000

Pearson Correlation Statistics (Fisher's z Transformation)									
**Variable**	**With Variable**	**N**	**Sample Correlation**	**Fisher's z**	**Bias Adjustment**	**Correlation Estimate**	**95% Confidence Limits**		**p Value for H0:Rho=0**
**top2lg2mean**	**MAF**	12	-0.35552	-0.37175	-0.01616	-0.34133	-0.765312	0.289233	0.2647

Correlation C_pooltop2lg2mean_tp2 symbol MAF

The CORR Procedure

Gene_Symbol=A2M

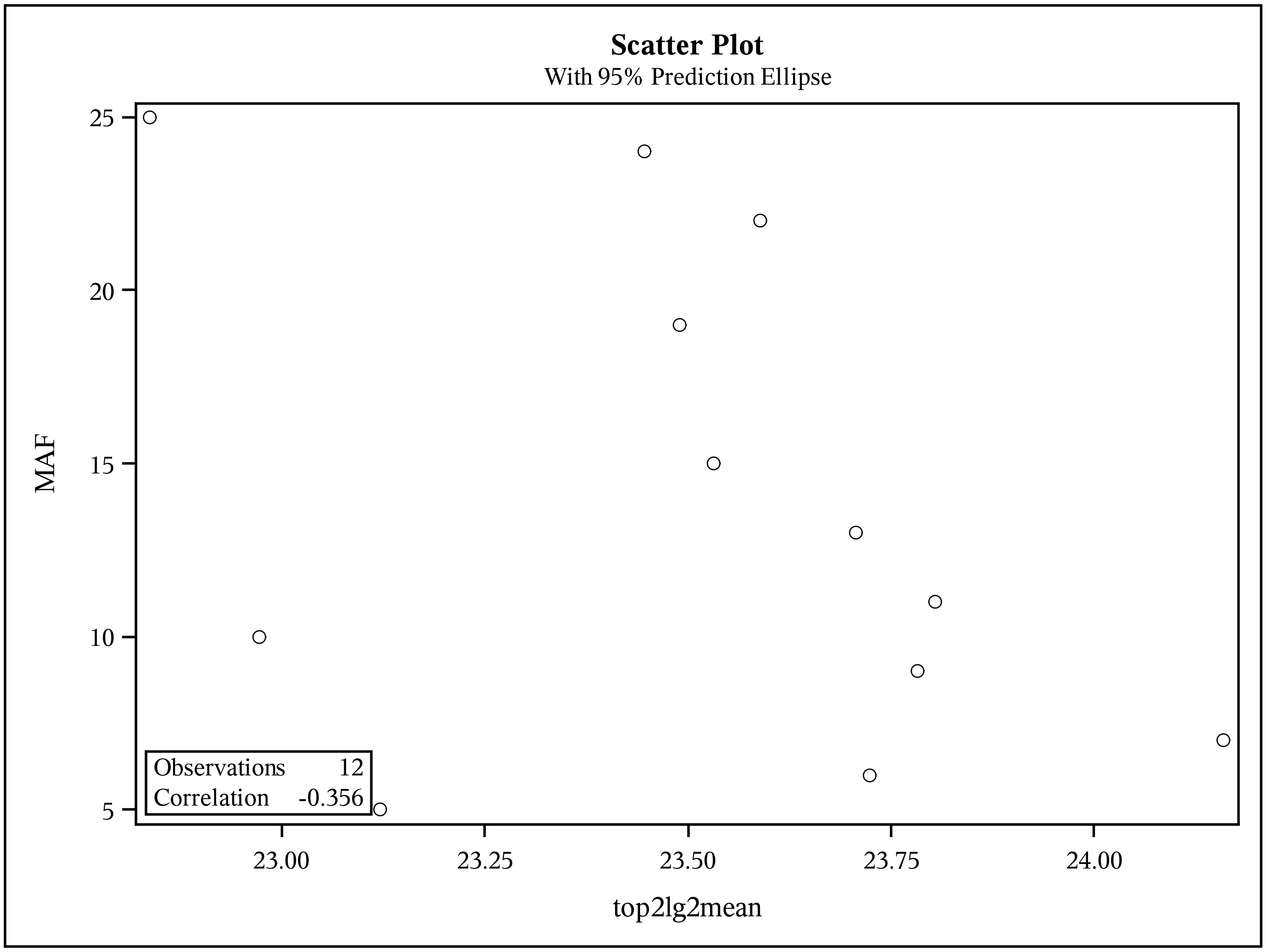

Correlation C_pooltop2lg2mean_tp2 symbol MAF

The CORR Procedure

Gene_Symbol=AGT

**2 Variables:**	top2lg2mean MAF

Simple Statistics						
**Variable**	**N**	**Mean**	**Std Dev**	**Sum**	**Minimum**	**Maximum**
**top2lg2mean**	12	24.82819	0.43733	297.93831	23.95626	25.72056
**MAF**	12	13.83333	7.10740	166.00000	5.00000	25.00000

Pearson Correlation Coefficients, N = 12<br>Prob > |r| under H0: Rho=0		
	**top2lg2mean**	**MAF**
**top2lg2mean**	1.00000	-0.41920<br>0.1750
**MAF**	-0.41920<br>0.1750	1.00000

Pearson Correlation Statistics (Fisher's z Transformation)									
**Variable**	**With Variable**	**N**	**Sample Correlation**	**Fisher's z**	**Bias Adjustment**	**Correlation Estimate**	**95% Confidence Limits**		**p Value for H0:Rho=0**
**top2lg2mean**	**MAF**	12	-0.41920	-0.44672	-0.01905	-0.40337	-0.793563	0.221906	0.1802

Correlation C_pooltop2lg2mean_tp2 symbol MAF

The CORR Procedure

Gene_Symbol=AGT

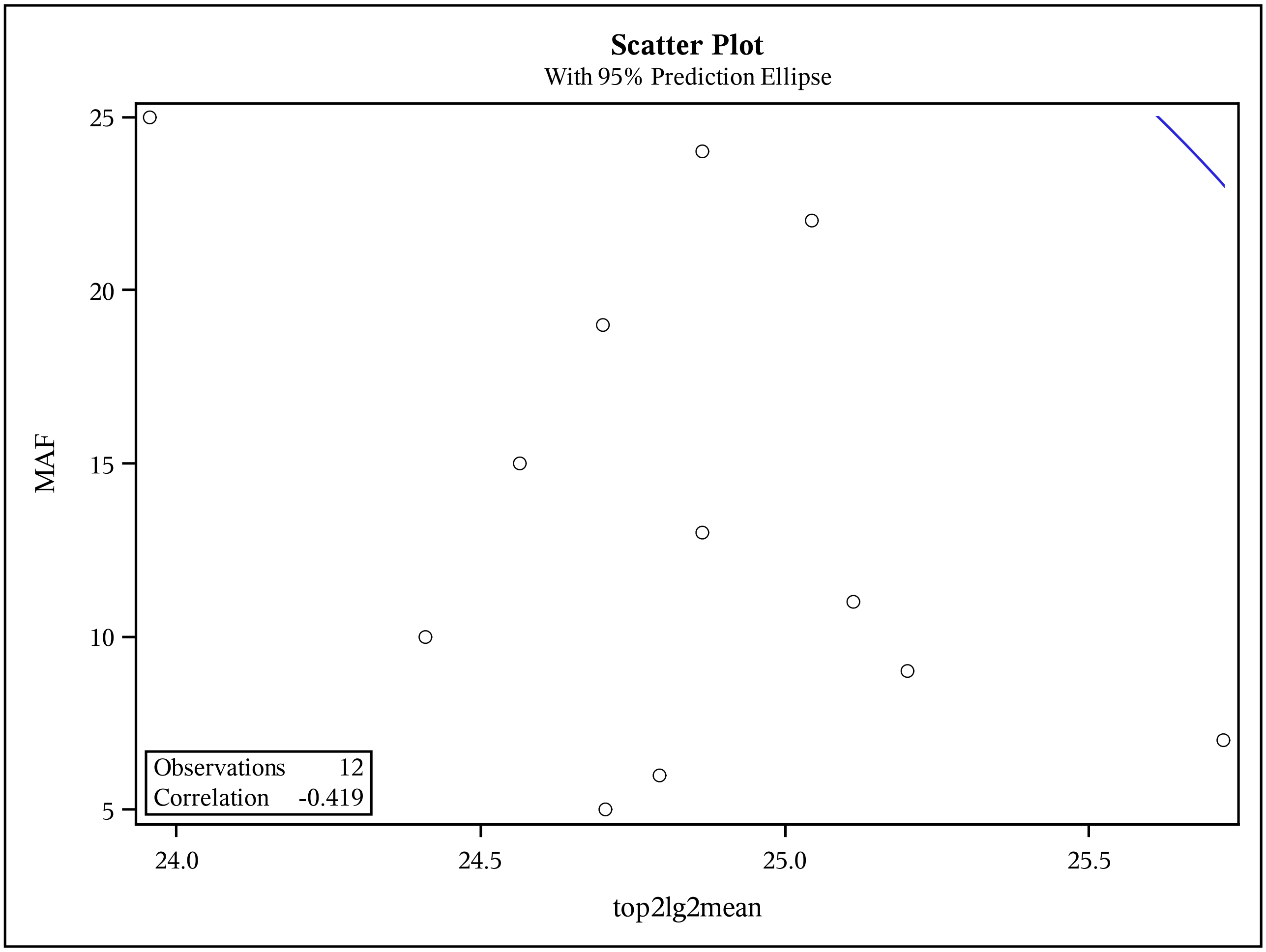

Correlation C_pooltop2lg2mean_tp2 symbol MAF

The CORR Procedure

Gene_Symbol=AHSG

2 Variables:	top2lg2mean MAF

Simple Statistics						
Variable	N	Mean	Std Dev	Sum	Minimum	Maximum
top2lg2mean	12	22.54882	0.14816	270.58587	22.32566	22.82825
MAF	12	13.83333	7.10740	166.00000	5.00000	25.00000

Pearson Correlation Coefficients, N = 12<br>Prob > |r| under H0: Rho=0		
	top2lg2mean	MAF
top2lg2mean	1.00000	0.07588<br>0.8147
MAF	0.07588<br>0.8147	1.00000

Pearson Correlation Statistics (Fisher's z Transformation)									
Variable	With Variable	N	Sample Correlation	Fisher's z	Bias Adjustment	Correlation Estimate	95% Confidence Limits		p Value for H0:Rho=0
top2lg2mean	MAF	12	0.07588	0.07603	0.00345	0.07245	-0.523203	0.620552	0.8196

Correlation C_pooltop2lg2mean_tp2 symbol MAF

The CORR Procedure

Gene_Symbol=AHSG

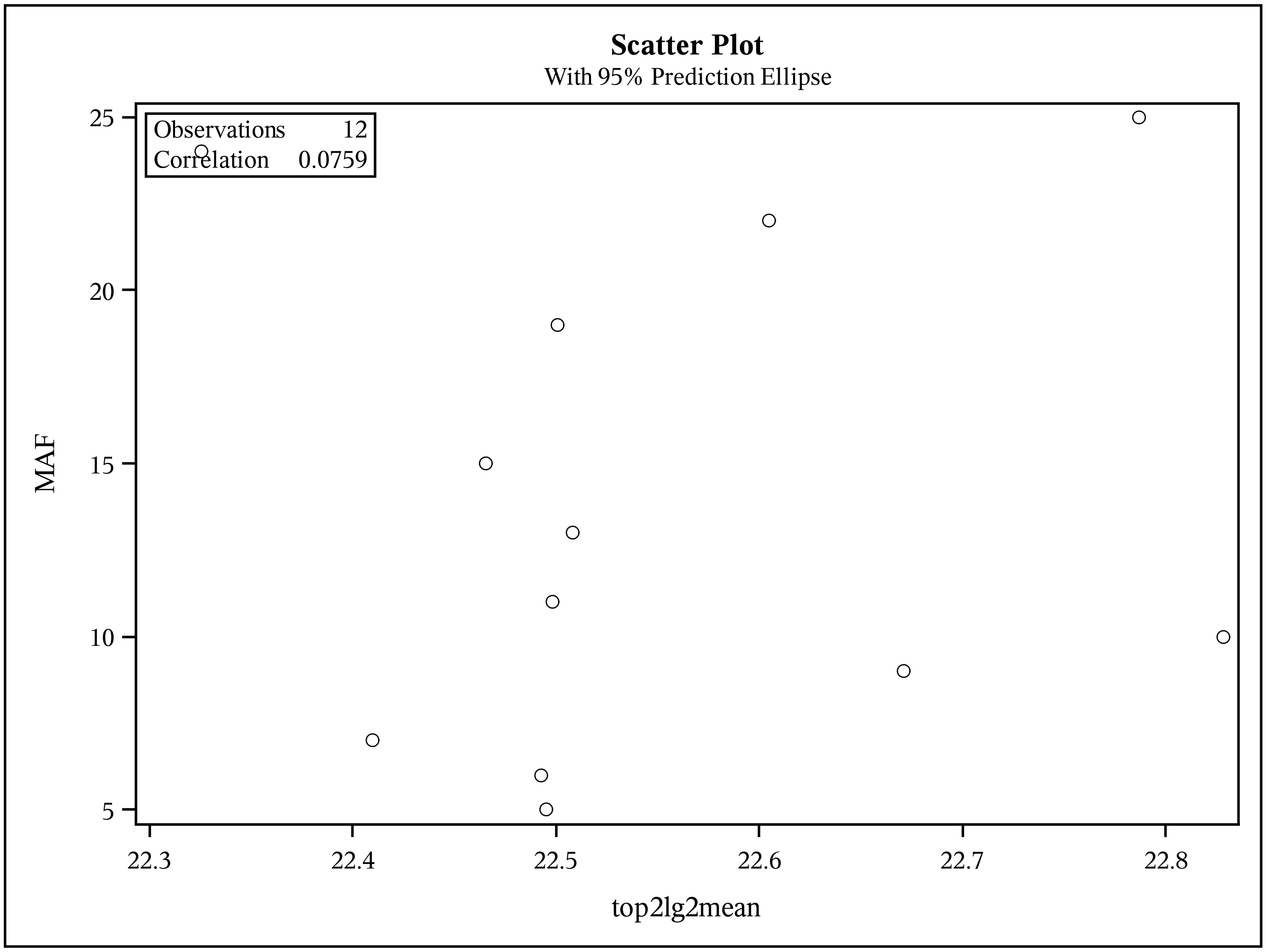

Correlation C_pooltop2lg2mean_tp2 symbol MAF

The CORR Procedure

Gene_Symbol=APLP1

**2 Variables:**	top2lg2mean MAF

Simple Statistics						
**Variable**	**N**	**Mean**	**Std Dev**	**Sum**	**Minimum**	**Maximum**
**top2lg2mean**	12	23.16222	0.29325	277.94665	22.65640	23.64982
**MAF**	12	13.83333	7.10740	166.00000	5.00000	25.00000

Pearson Correlation Coefficients, N = 12<br>Prob > |r| under H0: Rho=0		
	**top2lg2mean**	**MAF**
**top2lg2mean**	1.00000	-0.36811<br>0.2391
**MAF**	-0.36811<br>0.2391	1.00000

Pearson Correlation Statistics (Fisher's z Transformation)									
**Variable**	**With Variable**	**N**	**Sample Correlation**	**Fisher's z**	**Bias Adjustment**	**Correlation Estimate**	**95% Confidence Limits**		**p Value for H0:Rho=0**
**top2lg2mean**	**MAF**	12	-0.36811	-0.38623	-0.01673	-0.35356	-0.771014	0.276437	0.2466

Correlation C_pooltop2lg2mean_tp2 symbol MAF

The CORR Procedure

Gene_Symbol=APLP1

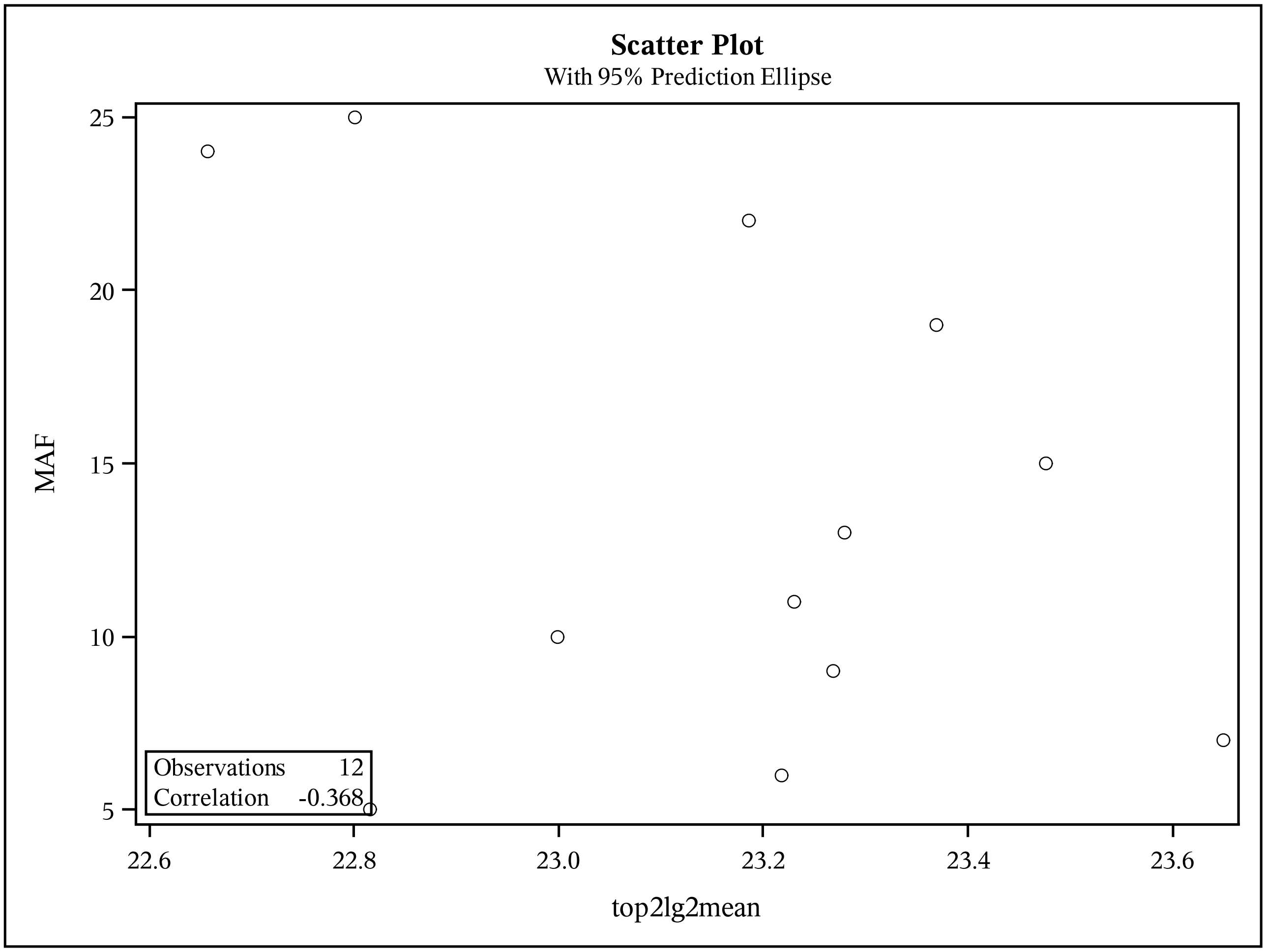

Correlation C_pooltop2lg2mean_tp2 symbol MAF

The CORR Procedure

Gene_Symbol=APOA1

**2 Variables:**	top2lg2mean MAF

Simple Statistics						
**Variable**	**N**	**Mean**	**Std Dev**	**Sum**	**Minimum**	**Maximum**
**top2lg2mean**	12	25.58229	0.22491	306.98745	25.28807	25.94263
**MAF**	12	13.83333	7.10740	166.00000	5.00000	25.00000

Pearson Correlation Coefficients, N = 12<br>Prob > |r| under H0: Rho=0		
	**top2lg2mean**	**MAF**
**top2lg2mean**	1.00000	0.15545<br>0.6295
**MAF**	0.15545<br>0.6295	1.00000

Pearson Correlation Statistics (Fisher's z Transformation)									
**Variable**	**With Variable**	**N**	**Sample Correlation**	**Fisher's z**	**Bias Adjustment**	**Correlation Estimate**	**95% Confidence Limits**		**p Value for H0:Rho=0**
**top2lg2mean**	**MAF**	12	0.15545	0.15672	0.00707	0.14854	-0.464998	0.665696	0.6382

Correlation C_pooltop2lg2mean_tp2 symbol MAF

The CORR Procedure

Gene_Symbol=APOA1

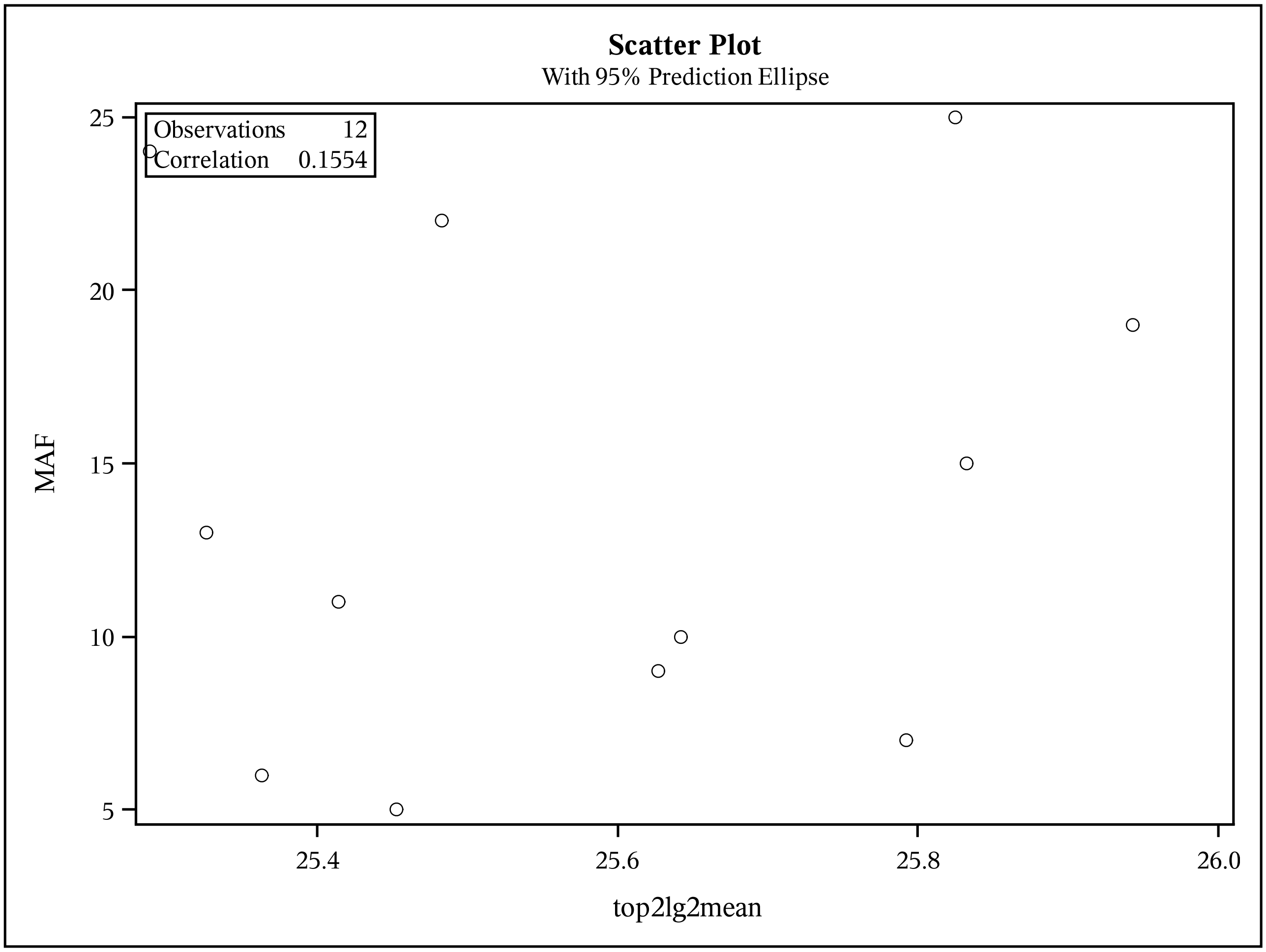

Correlation C_pooltop2lg2mean_tp2 symbol MAF

The CORR Procedure

Gene_Symbol=APOA4

2 Variables:	top2lg2mean MAF

Simple Statistics						
Variable	N	Mean	Std Dev	Sum	Minimum	Maximum
top2lg2mean	12	21.32405	0.37275	255.88859	20.64906	21.96869
MAF	12	13.83333	7.10740	166.00000	5.00000	25.00000

Pearson Correlation Coefficients, N = 12<br>Prob > |r| under H0: Rho=0		
	top2lg2mean	MAF
top2lg2mean	1.00000	0.16787<br>0.6020
MAF	0.16787<br>0.6020	1.00000

Pearson Correlation Statistics (Fisher's z Transformation)									
Variable	With Variable	N	Sample Correlation	Fisher's z	Bias Adjustment	Correlation Estimate	95% Confidence Limits		p Value for H0:Rho=0
top2lg2mean	MAF	12	0.16787	0.16948	0.00763	0.16045	-0.455387	0.672431	0.6112

Correlation C_pooltop2lg2mean_tp2 symbol MAF

The CORR Procedure

Gene_Symbol=APOA4

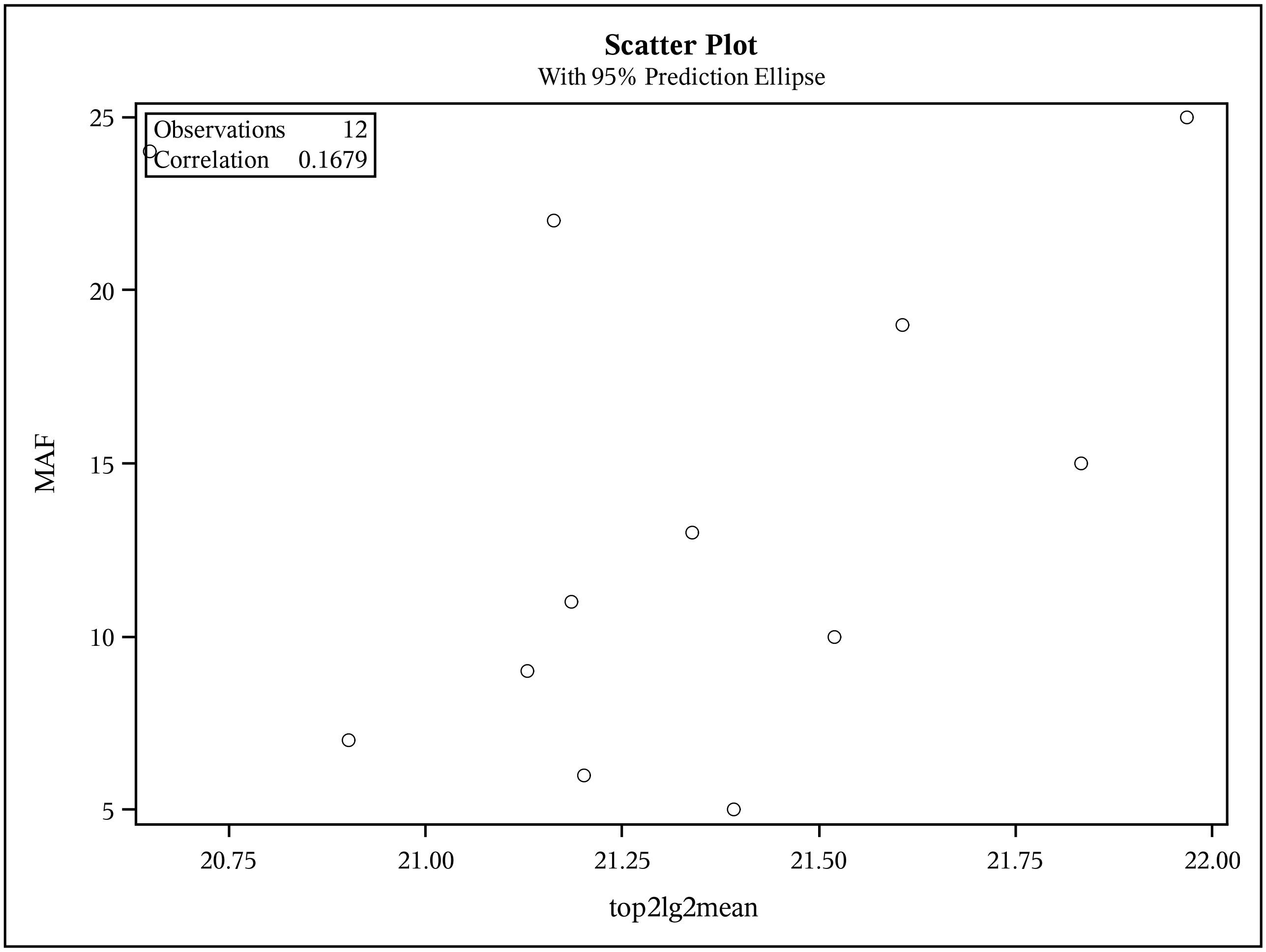

Correlation C_pooltop2lg2mean_tp2 symbol MAF

The CORR Procedure

Gene_Symbol=APOD

**2 Variables:**	top2lg2mean MAF

Simple Statistics						
**Variable**	**N**	**Mean**	**Std Dev**	**Sum**	**Minimum**	**Maximum**
**top2lg2mean**	12	21.87122	0.34024	262.45467	21.17123	22.36336
**MAF**	12	13.83333	7.10740	166.00000	5.00000	25.00000

Pearson Correlation Coefficients, N = 12<br>Prob > |r| under H0: Rho=0		
	**top2lg2mean**	**MAF**
**top2lg2mean**	1.00000	0.26564<br>0.4040
**MAF**	0.26564<br>0.4040	1.00000

Pearson Correlation Statistics (Fisher's z Transformation)									
**Variable**	**With Variable**	**N**	**Sample Correlation**	**Fisher's z**	**Bias Adjustment**	**Correlation Estimate**	**95% Confidence Limits**		**p Value for H0:Rho=0**
**top2lg2mean**	**MAF**	12	0.26564	0.27216	0.01207	0.25438	-0.374145	0.722764	0.4142

Correlation C_pooltop2lg2mean_tp2 symbol MAF

The CORR Procedure

Gene_Symbol=APOD

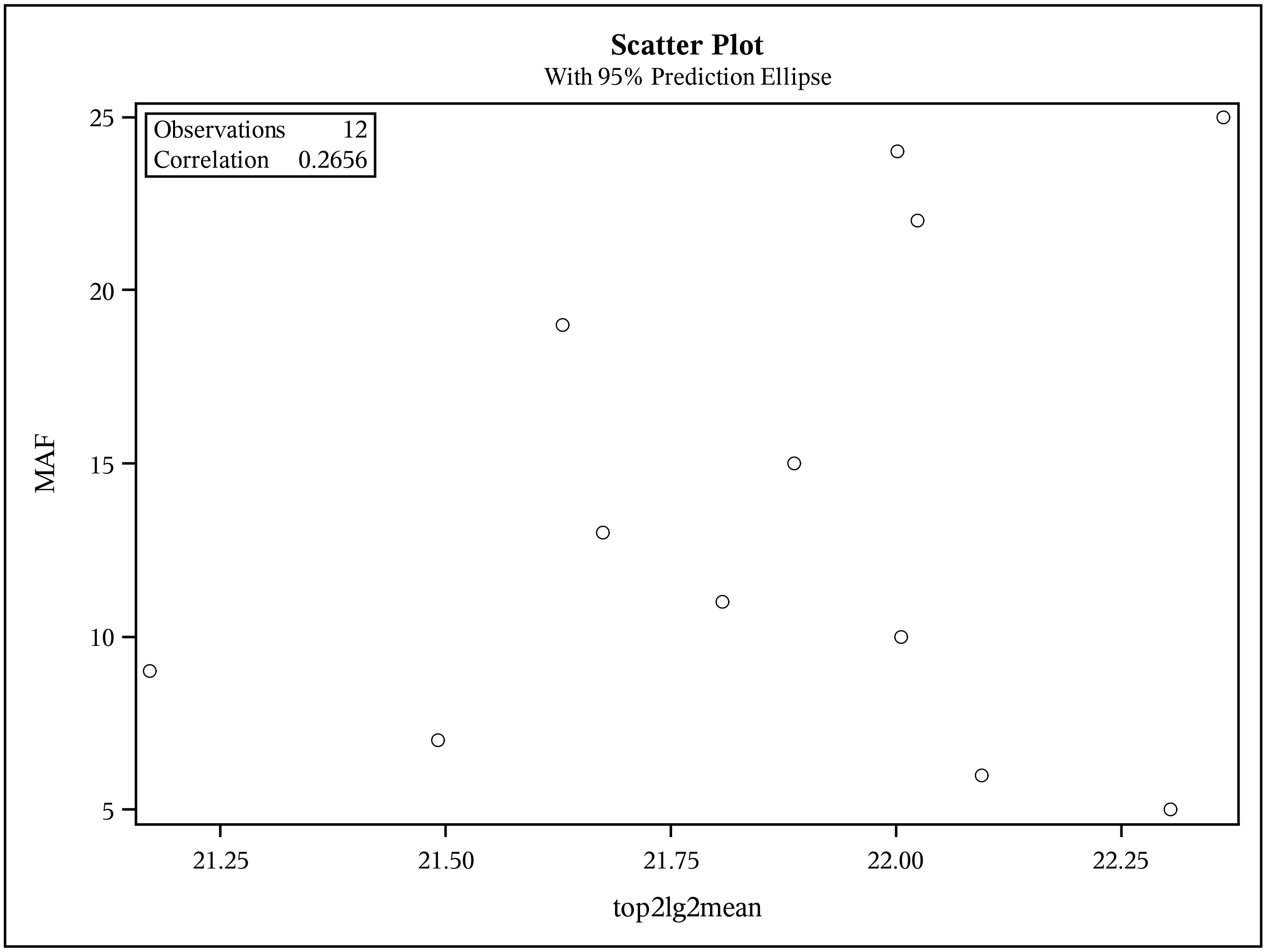

Correlation C_pooltop2lg2mean_tp2 symbol MAF

The CORR Procedure

Gene_Symbol=APOE

**2 Variables:**	top2lg2mean MAF

Simple Statistics						
**Variable**	**N**	**Mean**	**Std Dev**	**Sum**	**Minimum**	**Maximum**
**top2lg2mean**	12	23.90888	0.30086	286.90650	23.33682	24.38528
**MAF**	12	13.83333	7.10740	166.00000	5.00000	25.00000

Pearson Correlation Coefficients, N = 12<br>Prob > |r| under H0: Rho=0		
	**top2lg2mean**	**MAF**
**top2lg2mean**	1.00000	0.16097<br>0.6172
**MAF**	0.16097<br>0.6172	1.00000

Pearson Correlation Statistics (Fisher's z Transformation)									
**Variable**	**With Variable**	**N**	**Sample Correlation**	**Fisher's z**	**Bias Adjustment**	**Correlation Estimate**	**95% Confidence Limits**		**p Value for H0:Rho=0**
**top2lg2mean**	**MAF**	12	0.16097	0.16238	0.00732	0.15384	-0.460742	0.668701	0.6261

Correlation C_pooltop2lg2mean_tp2 symbol MAF

The CORR Procedure

Gene_Symbol=APOE

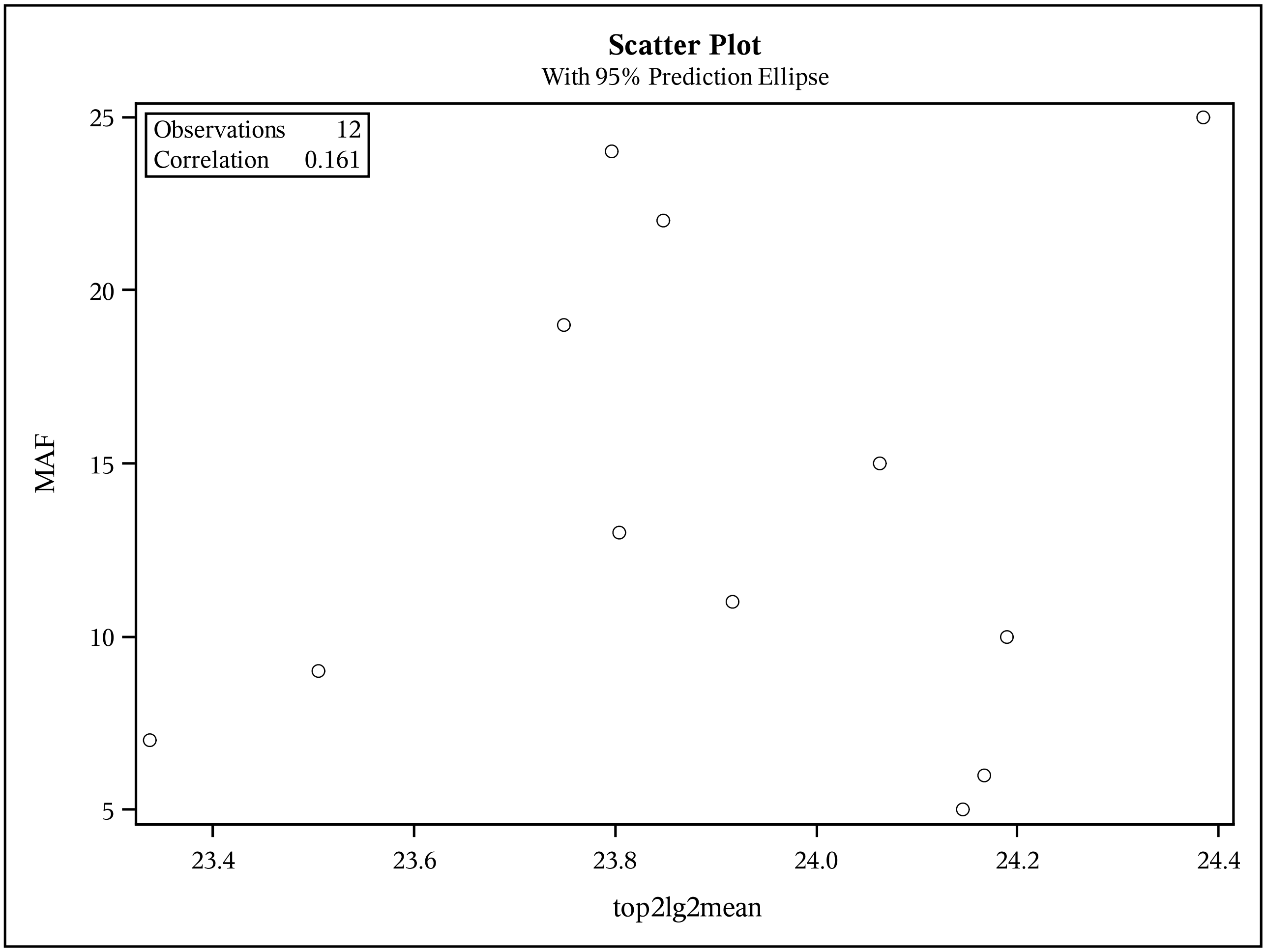

Correlation C_pooltop2lg2mean_tp2 symbol MAF

The CORR Procedure

Gene_Symbol=APOH

**2 Variables:**	top2lg2mean MAF

Simple Statistics						
**Variable**	**N**	**Mean**	**Std Dev**	**Sum**	**Minimum**	**Maximum**
**top2lg2mean**	12	22.29174	0.23615	267.50086	21.90547	22.59521
**MAF**	12	13.83333	7.10740	166.00000	5.00000	25.00000

Pearson Correlation Coefficients, N = 12<br>Prob > |r| under H0: Rho=0		
	**top2lg2mean**	**MAF**
**top2lg2mean**	1.00000	-0.27647<br>0.3844
**MAF**	-0.27647<br>0.3844	1.00000

Pearson Correlation Statistics (Fisher's z Transformation)									
**Variable**	**With Variable**	**N**	**Sample Correlation**	**Fisher's z**	**Bias Adjustment**	**Correlation Estimate**	**95% Confidence Limits**		**p Value for H0:Rho=0**
**top2lg2mean**	**MAF**	12	-0.27647	-0.28385	-0.01257	-0.26482	-0.728071	0.364472	0.3945

Correlation C_pooltop2lg2mean_tp2 symbol MAF

The CORR Procedure

Gene_Symbol=APOH

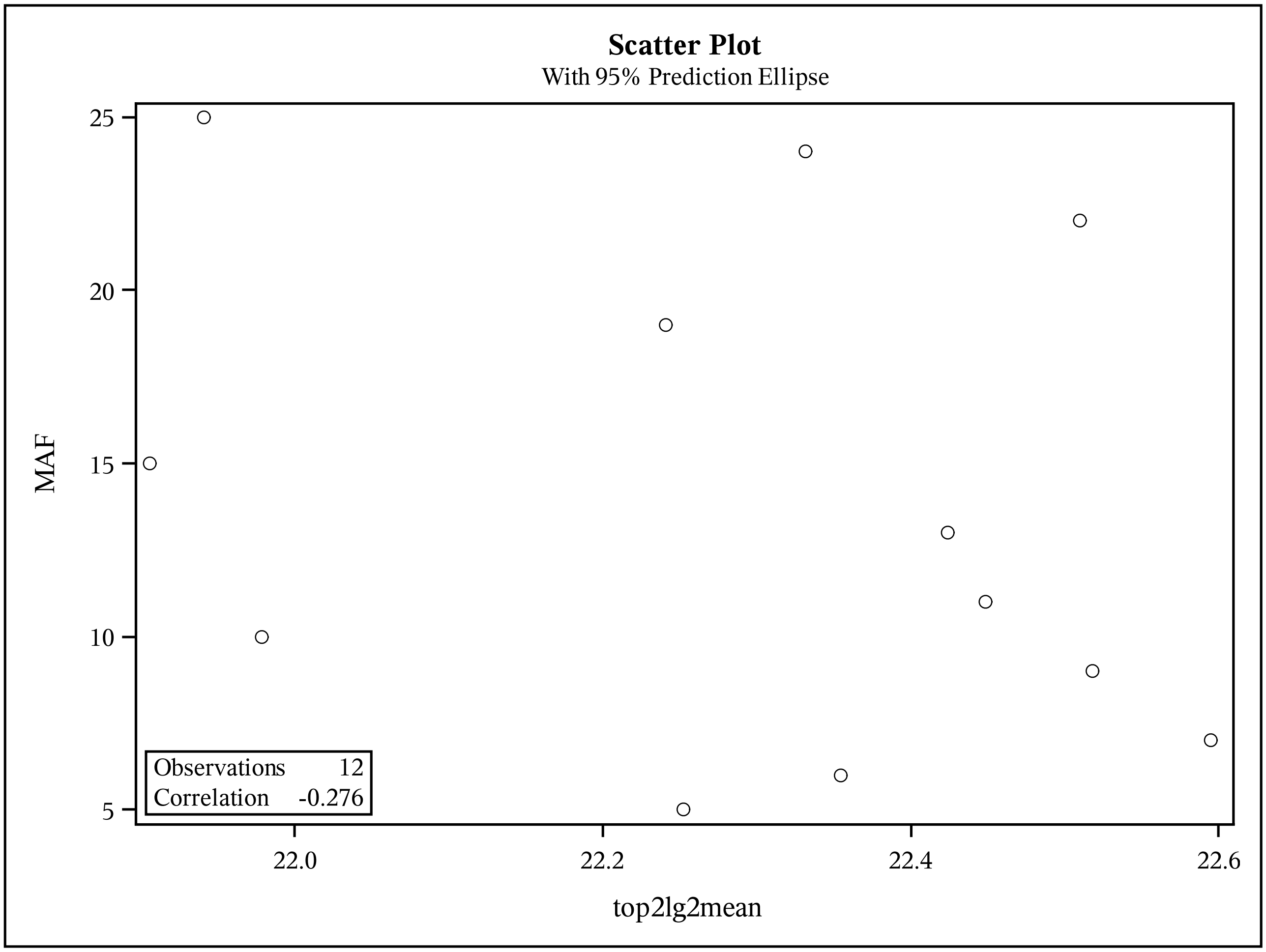

Correlation C_pooltop2lg2mean_tp2 symbol MAF

The CORR Procedure

Gene_Symbol=APP

2 Variables:	top2lg2mean MAF

Simple Statistics						
Variable	N	Mean	Std Dev	Sum	Minimum	Maximum
top2lg2mean	12	22.01715	0.31782	264.20575	21.17932	22.35052
MAF	12	13.83333	7.10740	166.00000	5.00000	25.00000

Pearson Correlation Coefficients, N = 12<br>Prob > |r| under H0: Rho=0		
	top2lg2mean	MAF
top2lg2mean	1.00000	0.31268<br>0.3224
MAF	0.31268<br>0.3224	1.00000

Pearson Correlation Statistics (Fisher's z Transformation)									
Variable	With Variable	N	Sample Correlation	Fisher's z	Bias Adjustment	Correlation Estimate	95% Confidence Limits		p Value for H0:Rho=0
top2lg2mean	MAF	12	0.31268	0.32351	0.01421	0.29980	-0.331064	0.745443	0.3318

Correlation C_pooltop2lg2mean_tp2 symbol MAF

The CORR Procedure

Gene_Symbol=APP

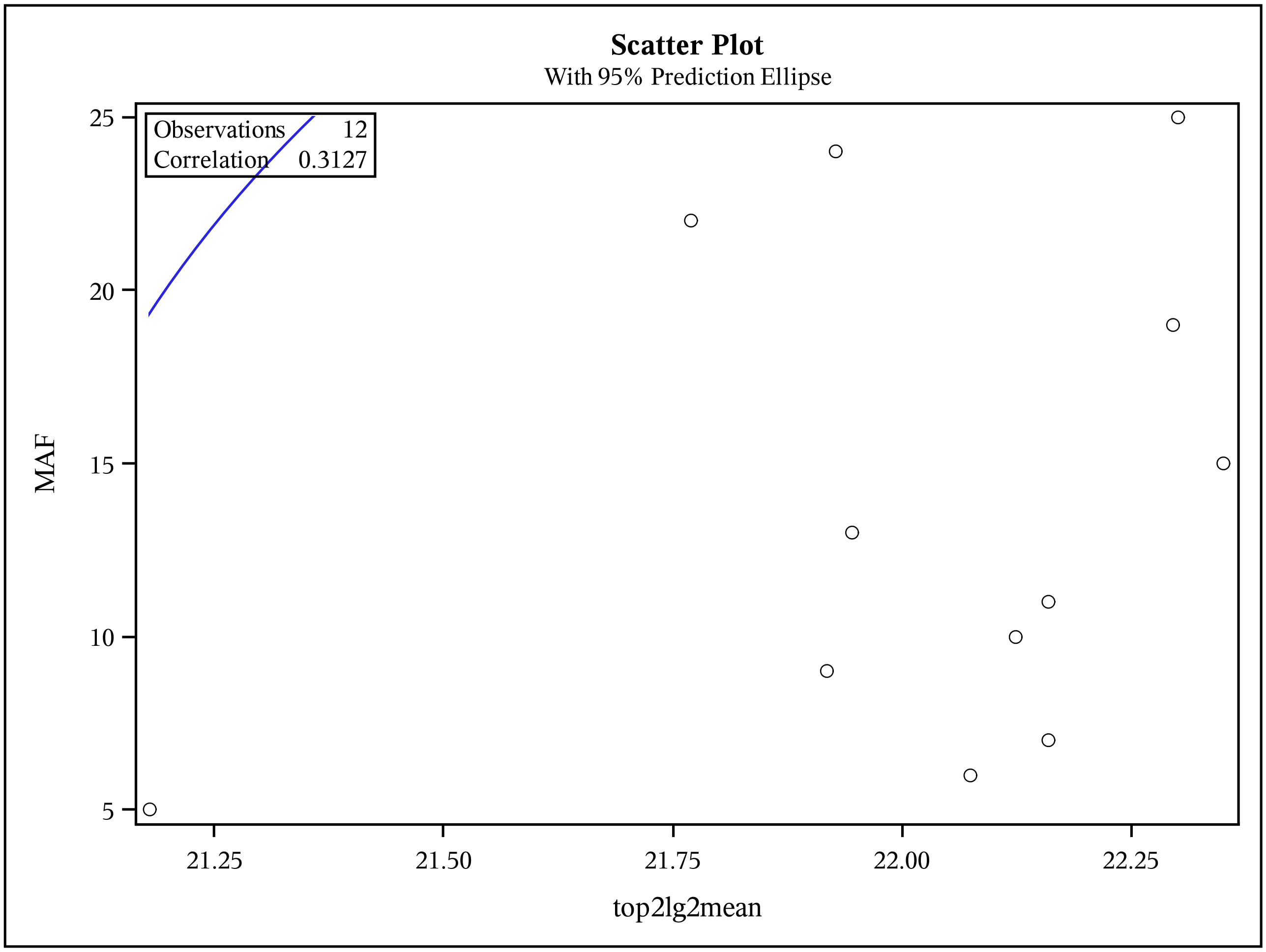

Correlation C_pooltop2lg2mean_tp2 symbol MAF

The CORR Procedure

Gene_Symbol=AZGP1

2 Variables:	top2lg2mean MAF

Simple Statistics						
Variable	N	Mean	Std Dev	Sum	Minimum	Maximum
top2lg2mean	12	21.82737	0.10820	261.92846	21.65513	22.02868
MAF	12	13.83333	7.10740	166.00000	5.00000	25.00000

Pearson Correlation Coefficients, N = 12<br>Prob > |r| under H0: Rho=0		
	top2lg2mean	MAF
top2lg2mean	1.00000	0.41986<br>0.1742
MAF	0.41986<br>0.1742	1.00000

Pearson Correlation Statistics (Fisher's z Transformation)									
Variable	With Variable	N	Sample Correlation	Fisher's z	Bias Adjustment	Correlation Estimate	95% Confidence Limits		p Value for H0:Rho=0
top2lg2mean	MAF	12	0.41986	0.44752	0.01908	0.40401	-0.221173	0.793849	0.1794

Correlation C_pooltop2lg2mean_tp2 symbol MAF

The CORR Procedure

Gene_Symbol=AZGP1

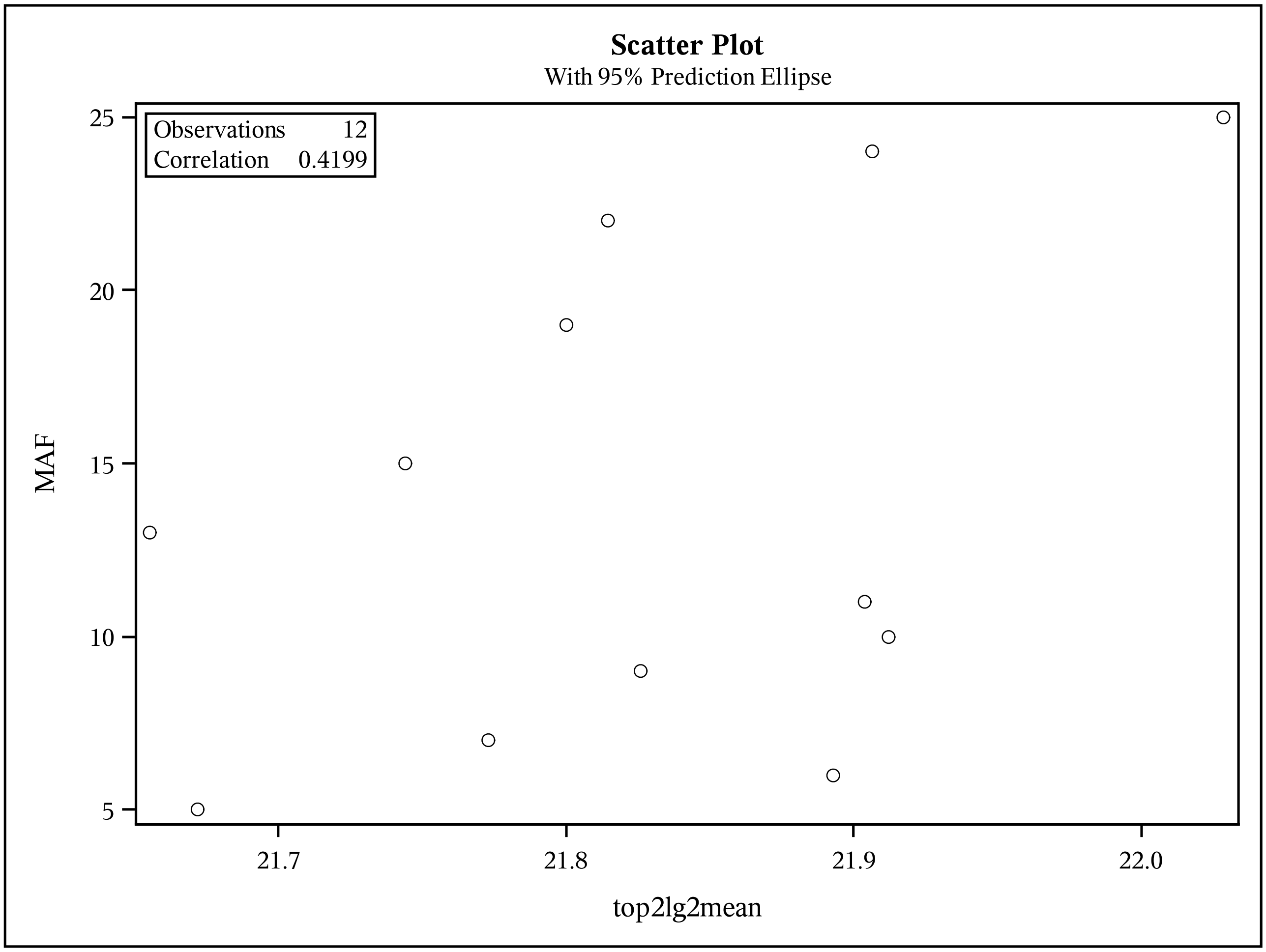

Correlation C_pooltop2lg2mean_tp2 symbol MAF

The CORR Procedure

Gene_Symbol=B2M

2 Variables:	top2lg2mean MAF

Simple Statistics						
Variable	N	Mean	Std Dev	Sum	Minimum	Maximum
top2lg2mean	12	23.15555	0.71637	277.86666	22.13271	24.50394
MAF	12	13.83333	7.10740	166.00000	5.00000	25.00000

Pearson Correlation Coefficients, N = 12<br>Prob > |r| under H0: Rho=0		
	top2lg2mean	MAF
top2lg2mean	1.00000	-0.45162<br>0.1405
MAF	-0.45162<br>0.1405	1.00000

Pearson Correlation Statistics (Fisher's z Transformation)									
Variable	With Variable	N	Sample Correlation	Fisher's z	Bias Adjustment	Correlation Estimate	95% Confidence Limits		p Value for H0:Rho=0
top2lg2mean	MAF	12	-0.45162	-0.48673	-0.02053	-0.43513	-0.807404	0.184963	0.1442

Correlation C_pooltop2lg2mean_tp2 symbol MAF

The CORR Procedure

Gene_Symbol=B2M

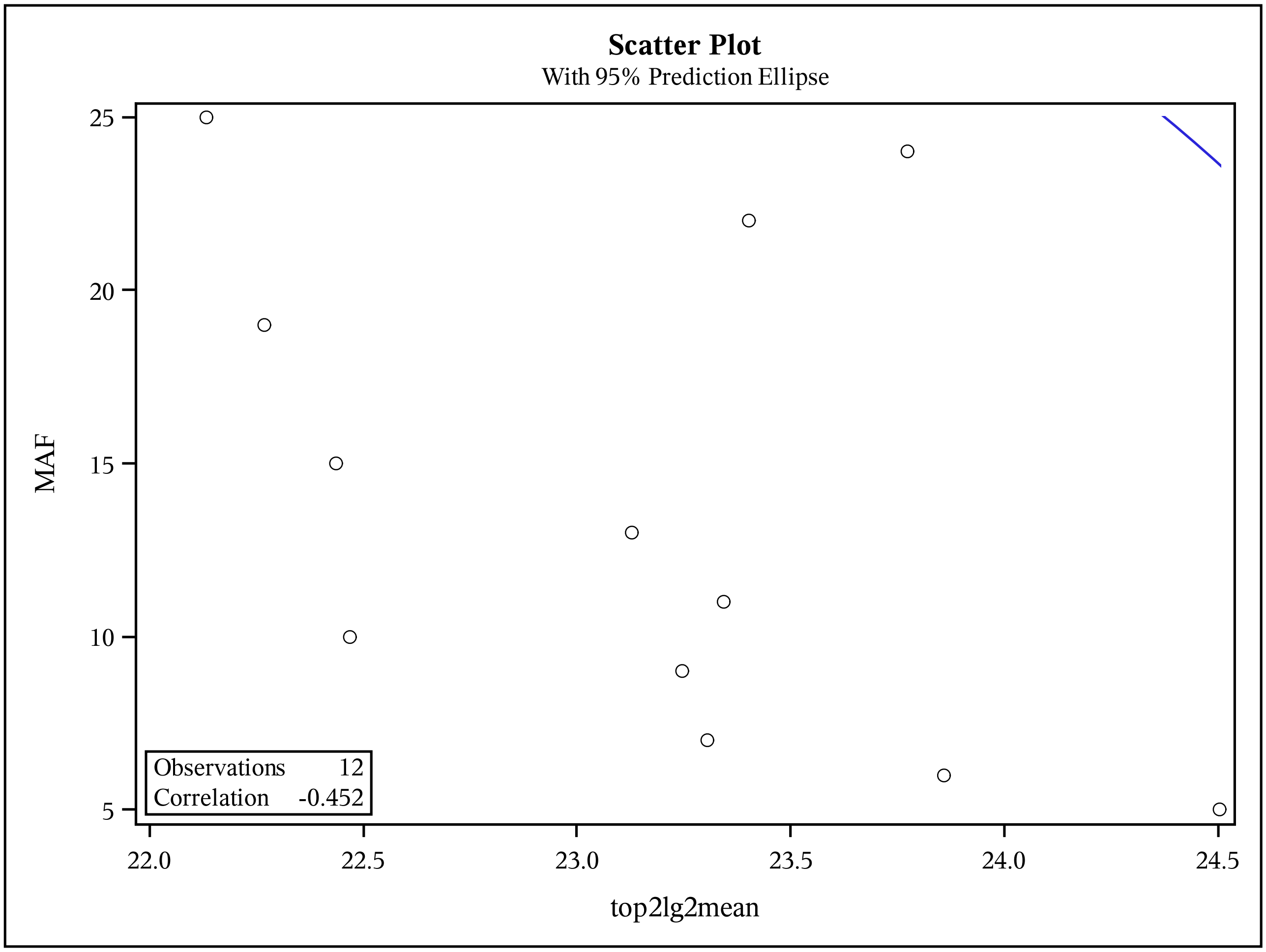

Correlation C_pooltop2lg2mean_tp2 symbol MAF

The CORR Procedure

Gene_Symbol=B3GNT1

**2 Variables:**	top2lg2mean MAF

Simple Statistics						
**Variable**	**N**	**Mean**	**Std Dev**	**Sum**	**Minimum**	**Maximum**
**top2lg2mean**	12	21.33297	0.12604	255.99567	21.16096	21.59144
**MAF**	12	13.83333	7.10740	166.00000	5.00000	25.00000

Pearson Correlation Coefficients, N = 12<br>Prob > |r| under H0: Rho=0		
	**top2lg2mean**	**MAF**
**top2lg2mean**	1.00000	0.23350<br>0.4651
**MAF**	0.23350<br>0.4651	1.00000

Pearson Correlation Statistics (Fisher's z Transformation)									
**Variable**	**With Variable**	**N**	**Sample Correlation**	**Fisher's z**	**Bias Adjustment**	**Correlation Estimate**	**95% Confidence Limits**		**p Value for H0:Rho=0**
**top2lg2mean**	**MAF**	12	0.23350	0.23789	0.01061	0.22344	-0.402013	0.706717	0.4754

Correlation C_pooltop2lg2mean_tp2 symbol MAF

The CORR Procedure

Gene_Symbol=B3GNT1

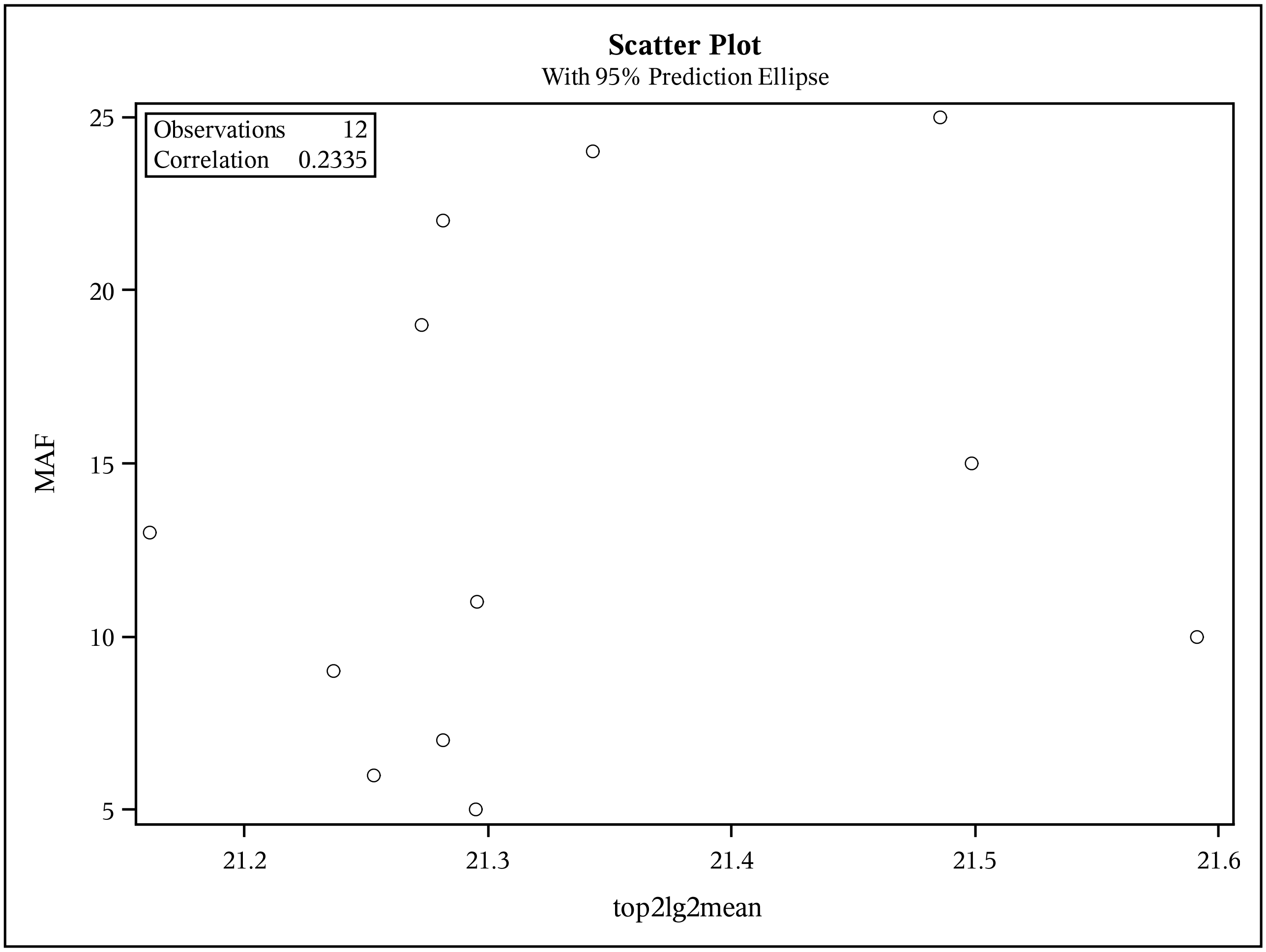

Correlation C_pooltop2lg2mean_tp2 symbol MAF

The CORR Procedure

Gene_Symbol=BCAN

2 Variables:	top2lg2mean MAF

Simple Statistics						
**Variable**	**N**	**Mean**	**Std Dev**	**Sum**	**Minimum**	**Maximum**
**top2lg2mean**	12	19.52838	0.34793	234.34050	18.70516	20.06111
**MAF**	12	13.83333	7.10740	166.00000	5.00000	25.00000

Pearson Correlation Coefficients, N = 12<br>Prob > |r| under H0: Rho=0		
	**top2lg2mean**	**MAF**
**top2lg2mean**	1.00000	0.38761<br>0.2132
**MAF**	0.38761<br>0.2132	1.00000

Pearson Correlation Statistics (Fisher's z Transformation)									
**Variable**	**With Variable**	**N**	**Sample Correlation**	**Fisher's z**	**Bias Adjustment**	**Correlation Estimate**	**95% Confidence Limits**		**p Value for H0:Rho=0**
**top2lg2mean**	**MAF**	12	0.38761	0.40899	0.01762	0.37254	-0.256123	0.779732	0.2198

Correlation C_pooltop2lg2mean_tp2 symbol MAF

The CORR Procedure

Gene_Symbol=BCAN

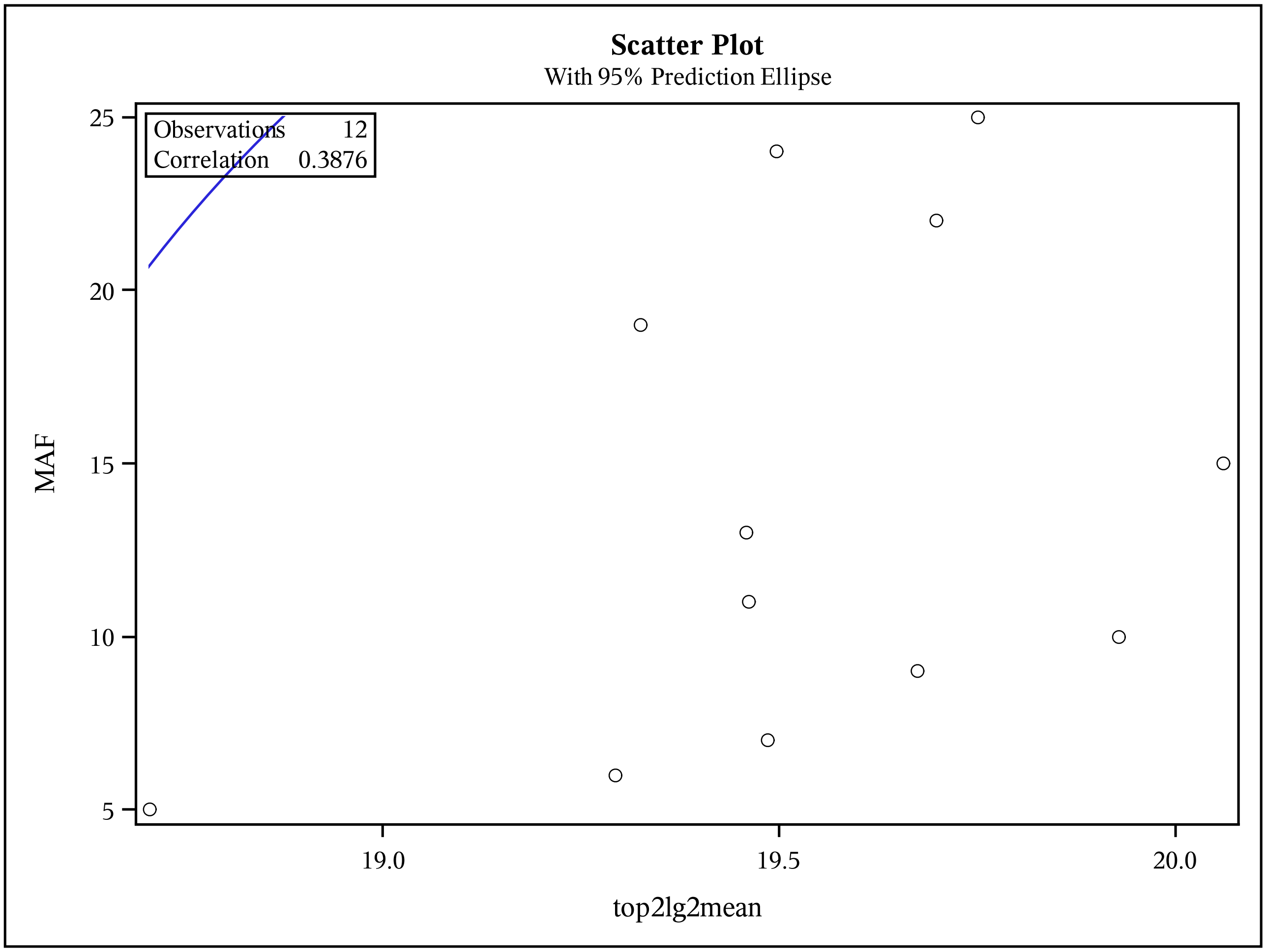

Correlation C_pooltop2lg2mean_tp2 symbol MAF

The CORR Procedure

Gene_Symbol=C1S

**2 Variables:**	top2lg2mean MAF

Simple Statistics						
**Variable**	**N**	**Mean**	**Std Dev**	**Sum**	**Minimum**	**Maximum**
**top2lg2mean**	12	20.39884	0.20738	244.78613	20.08916	20.75487
**MAF**	12	13.83333	7.10740	166.00000	5.00000	25.00000

Pearson Correlation Coefficients, N = 12<br>Prob > |r| under H0: Rho=0		
	**top2lg2mean**	**MAF**
**top2lg2mean**	1.00000	0.59823<br>0.0399
**MAF**	0.59823<br>0.0399	1.00000

Pearson Correlation Statistics (Fisher's z Transformation)									
**Variable**	**With Variable**	**N**	**Sample Correlation**	**Fisher's z**	**Bias Adjustment**	**Correlation Estimate**	**95% Confidence Limits**		**p Value for H0:Rho=0**
**top2lg2mean**	**MAF**	12	0.59823	0.69038	0.02719	0.58048	0.009869	0.865914	0.0383

Correlation C_pooltop2lg2mean_tp2 symbol MAF

The CORR Procedure

Gene_Symbol=C1S

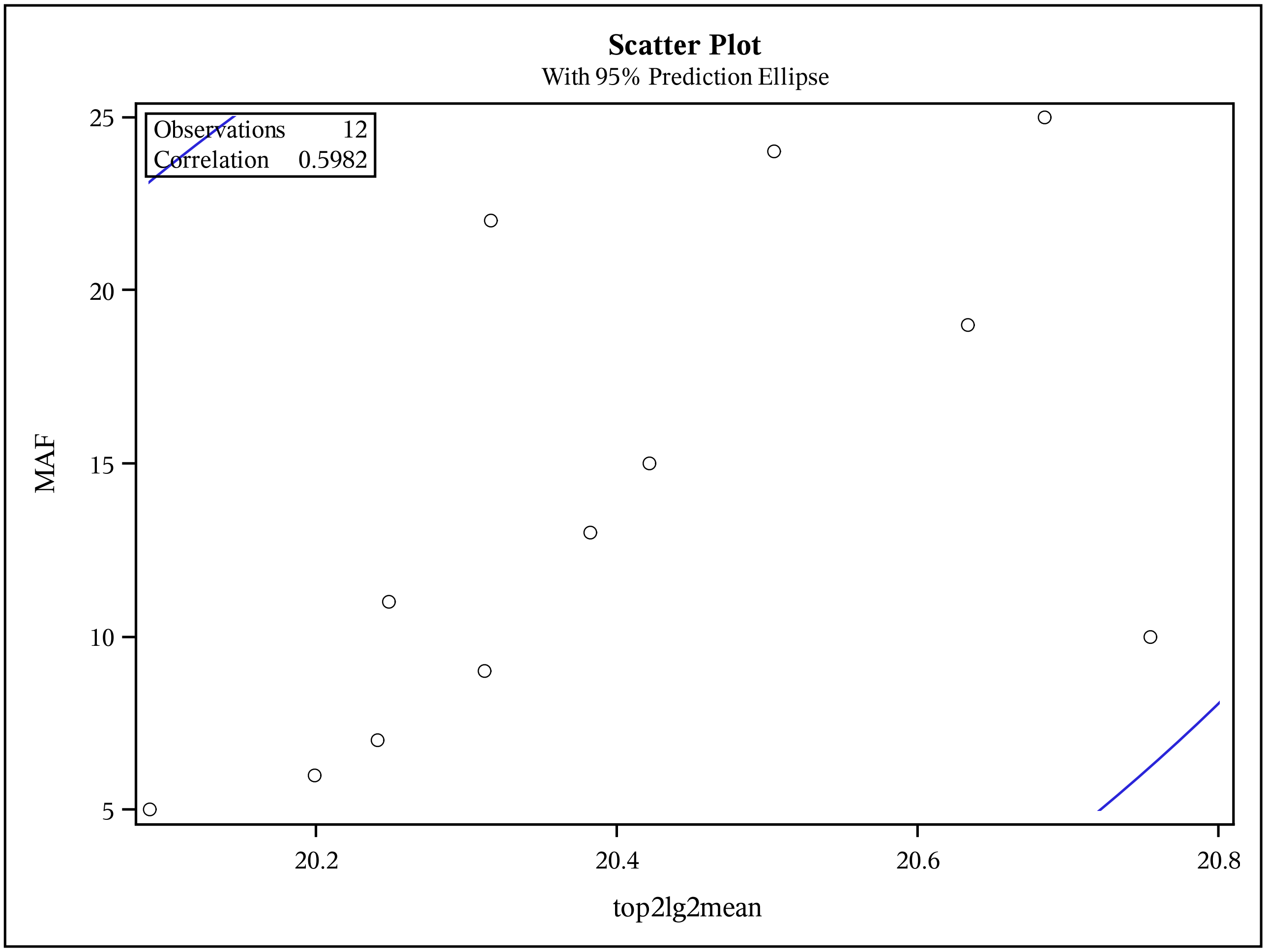

Correlation C_pooltop2lg2mean_tp2 symbol MAF

The CORR Procedure

Gene_Symbol=C3

**2 Variables:**	top2lg2mean MAF

Simple Statistics						
**Variable**	**N**	**Mean**	**Std Dev**	**Sum**	**Minimum**	**Maximum**
**top2lg2mean**	12	23.64879	0.24812	283.78550	23.13520	24.02660
**MAF**	12	13.83333	7.10740	166.00000	5.00000	25.00000

Pearson Correlation Coefficients, N = 12<br>Prob > |r| under H0: Rho=0		
	**top2lg2mean**	**MAF**
**top2lg2mean**	1.00000	-0.23343<br>0.4653
**MAF**	-0.23343<br>0.4653	1.00000

Pearson Correlation Statistics (Fisher's z Transformation)									
**Variable**	**With Variable**	**N**	**Sample Correlation**	**Fisher's z**	**Bias Adjustment**	**Correlation Estimate**	**95% Confidence Limits**		**p Value for H0:Rho=0**
**top2lg2mean**	**MAF**	12	-0.23343	-0.23781	-0.01061	-0.22337	-0.706681	0.402073	0.4756

Correlation C_pooltop2lg2mean_tp2 symbol MAF

The CORR Procedure

Gene_Symbol=C3

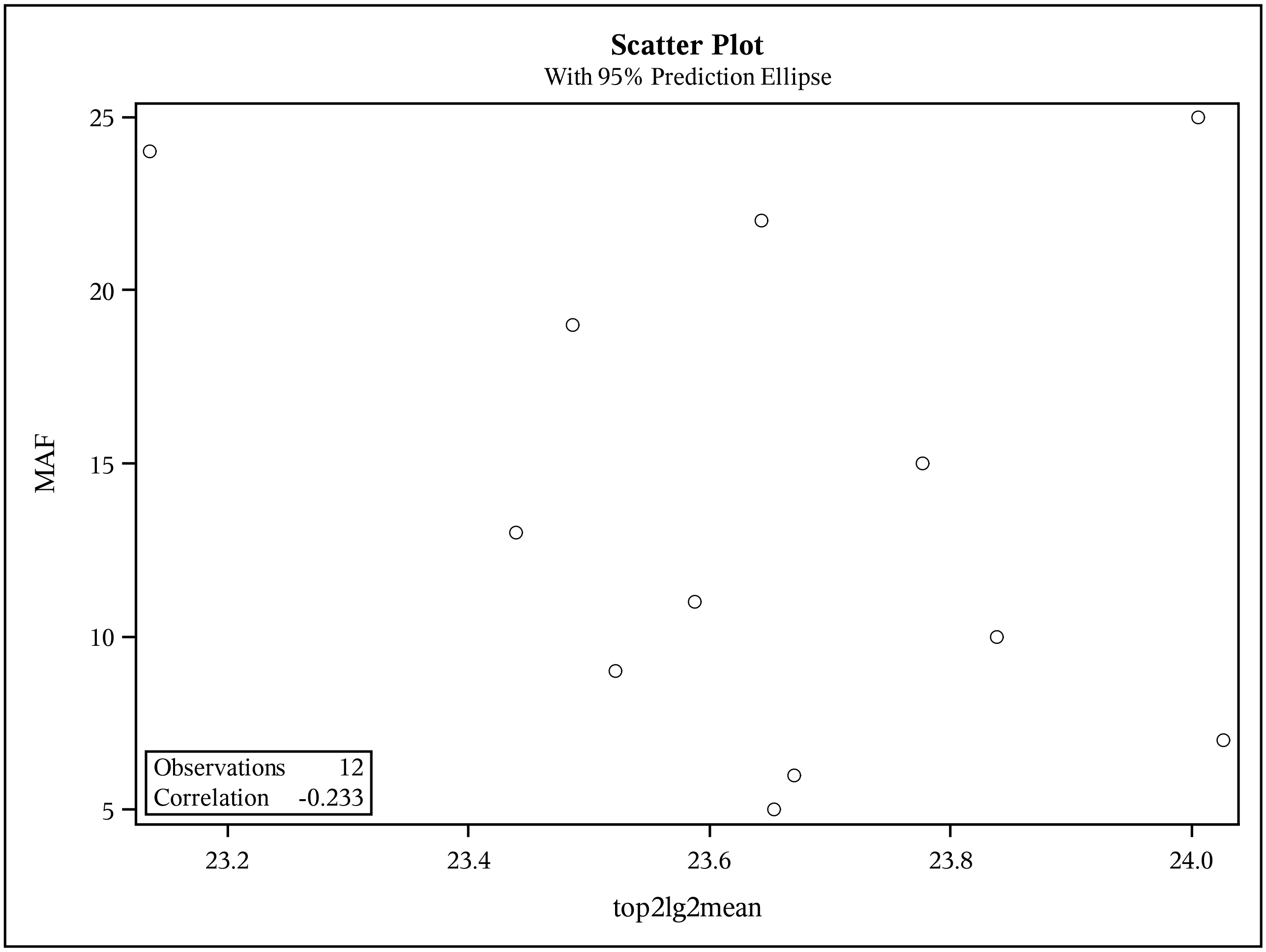

Correlation C_pooltop2lg2mean_tp2 symbol MAF

The CORR Procedure

Gene_Symbol=C4A-C4B

2 Variables:	top2lg2mean MAF

Simple Statistics						
**Variable**	**N**	**Mean**	**Std Dev**	**Sum**	**Minimum**	**Maximum**
**top2lg2mean**	12	23.25294	0.43429	279.03527	22.17556	23.98024
**MAF**	12	13.83333	7.10740	166.00000	5.00000	25.00000

Pearson Correlation Coefficients, N = 12<br>Prob > |r| under H0: Rho=0		
	**top2lg2mean**	**MAF**
**top2lg2mean**	1.00000	0.07725<br>0.8114
**MAF**	0.07725<br>0.8114	1.00000

Pearson Correlation Statistics (Fisher's z Transformation)									
**Variable**	**With Variable**	**N**	**Sample Correlation**	**Fisher's z**	**Bias Adjustment**	**Correlation Estimate**	**95% Confidence Limits**		**p Value for H0:Rho=0**
**top2lg2mean**	**MAF**	12	0.07725	0.07740	0.00351	0.07376	-0.522252	0.621356	0.8164

Correlation C_pooltop2lg2mean_tp2 symbol MAF

The CORR Procedure

Gene_Symbol=C4A-C4B

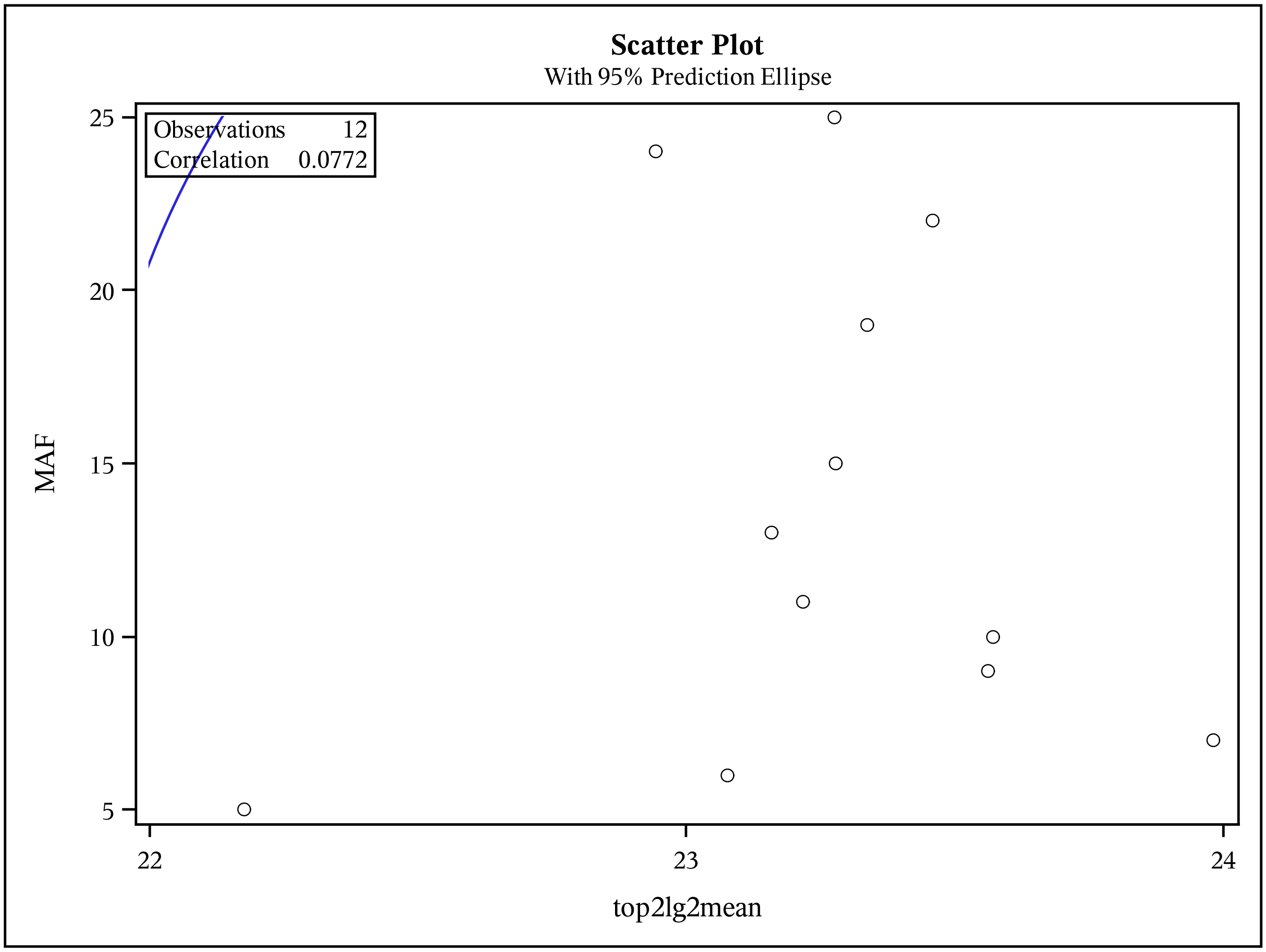

Correlation C_pooltop2lg2mean_tp2 symbol MAF

The CORR Procedure

Gene_Symbol=C7

**2 Variables:**	top2lg2mean MAF

Simple Statistics						
**Variable**	**N**	**Mean**	**Std Dev**	**Sum**	**Minimum**	**Maximum**
**top2lg2mean**	12	19.42969	0.32606	233.15628	18.96587	19.88187
**MAF**	12	13.83333	7.10740	166.00000	5.00000	25.00000

Pearson Correlation Coefficients, N = 12<br>Prob > |r| under H0: Rho=0		
	**top2lg2mean**	**MAF**
**top2lg2mean**	1.00000	0.14164<br>0.6606
**MAF**	0.14164<br>0.6606	1.00000

Pearson Correlation Statistics (Fisher's z Transformation)										
**Variable**	**With Variable**	**N**	**Sample Correlation**	**Fisher's z**	**Bias Adjustment**	**Correlation Estimate**	**95% Confidence Limits**		**p Value for H0:Rho=0**
**top2lg2mean**	**MAF**	12	0.14164	0.14259	0.00644	0.13532	-0.475509	0.658113	0.6688

Correlation C_pooltop2lg2mean_tp2 symbol MAF

The CORR Procedure

Gene_Symbol=C7

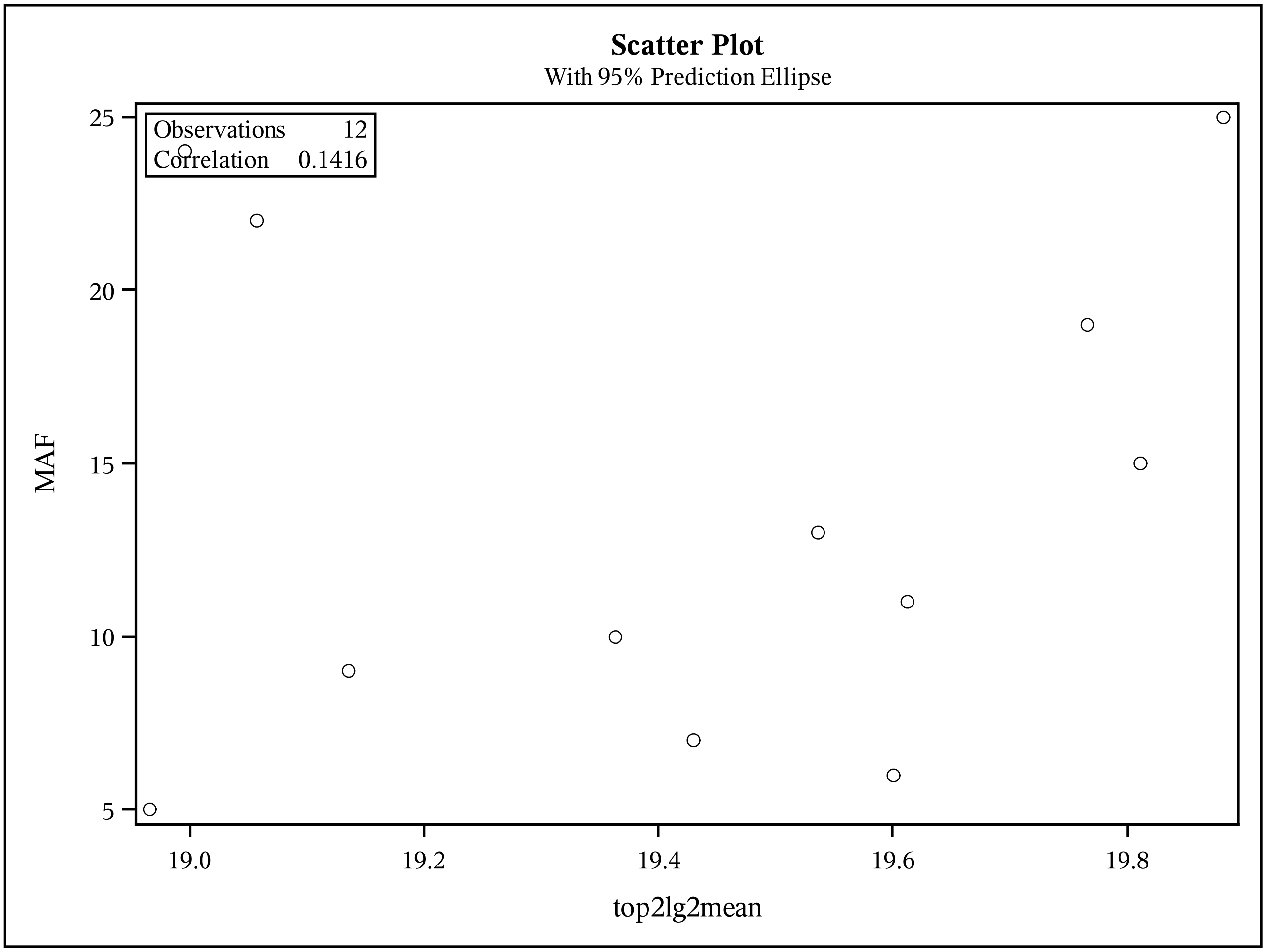

Correlation C_pooltop2lg2mean_tp2 symbol MAF

The CORR Procedure

Gene_Symbol=CD14

2 Variables:	top2lg2mean MAF

Simple Statistics						
Variable	N	Mean	Std Dev	Sum	Minimum	Maximum
top2lg2mean	12	21.00887	0.30714	252.10641	20.57639	21.50118
MAF	12	13.83333	7.10740	166.00000	5.00000	25.00000

Pearson Correlation Coefficients, N = 12<br>Prob > |r| under H0: Rho=0		
	top2lg2mean	MAF
top2lg2mean	1.00000	0.32389<br>0.3044
MAF	0.32389<br>0.3044	1.00000

Pearson Correlation Statistics (Fisher's z Transformation)									
Variable	With Variable	N	Sample Correlation	Fisher's z	Bias Adjustment	Correlation Estimate	95% Confidence Limits		p Value for H0:Rho=0
top2lg2mean	MAF	12	0.32389	0.33598	0.01472	0.31065	-0.320371	0.750711	0.3135

Correlation C_pooltop2lg2mean_tp2 symbol MAF

The CORR Procedure

Gene_Symbol=CD14

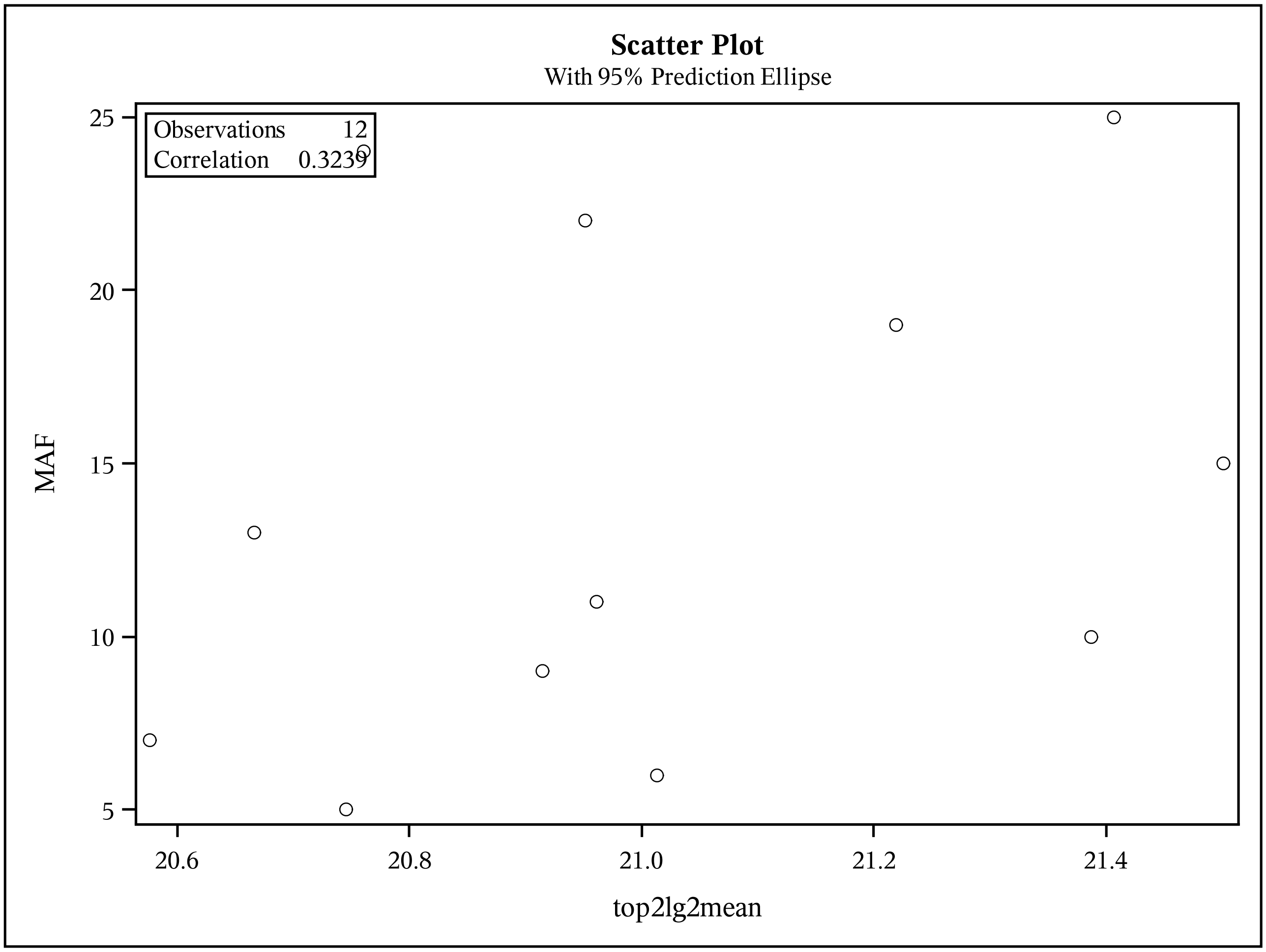

Correlation C_pooltop2lg2mean_tp2 symbol MAF

The CORR Procedure

Gene_Symbol=CD59

**2 Variables:**	top2lg2mean MAF

Simple Statistics						
**Variable**	**N**	**Mean**	**Std Dev**	**Sum**	**Minimum**	**Maximum**
**top2lg2mean**	12	20.70554	0.36595	248.46650	19.73407	21.12204
**MAF**	12	13.83333	7.10740	166.00000	5.00000	25.00000

Pearson Correlation Coefficients, N = 12<br>Prob > |r| under H0: Rho=0		
	**top2lg2mean**	**MAF**
**top2lg2mean**	1.00000	0.53446<br>0.0734
**MAF**	0.53446<br>0.0734	1.00000

Pearson Correlation Statistics (Fisher's z Transformation)									
**Variable**	**With Variable**	**N**	**Sample Correlation**	**Fisher's z**	**Bias Adjustment**	**Correlation Estimate**	**95% Confidence Limits**		**p Value for H0:Rho=0**
**top2lg2mean**	**MAF**	12	0.53446	0.59636	0.02429	0.51688	-0.081073	0.841237	0.0736

Correlation C_pooltop2lg2mean_tp2 symbol MAF

The CORR Procedure

Gene_Symbol=CD59

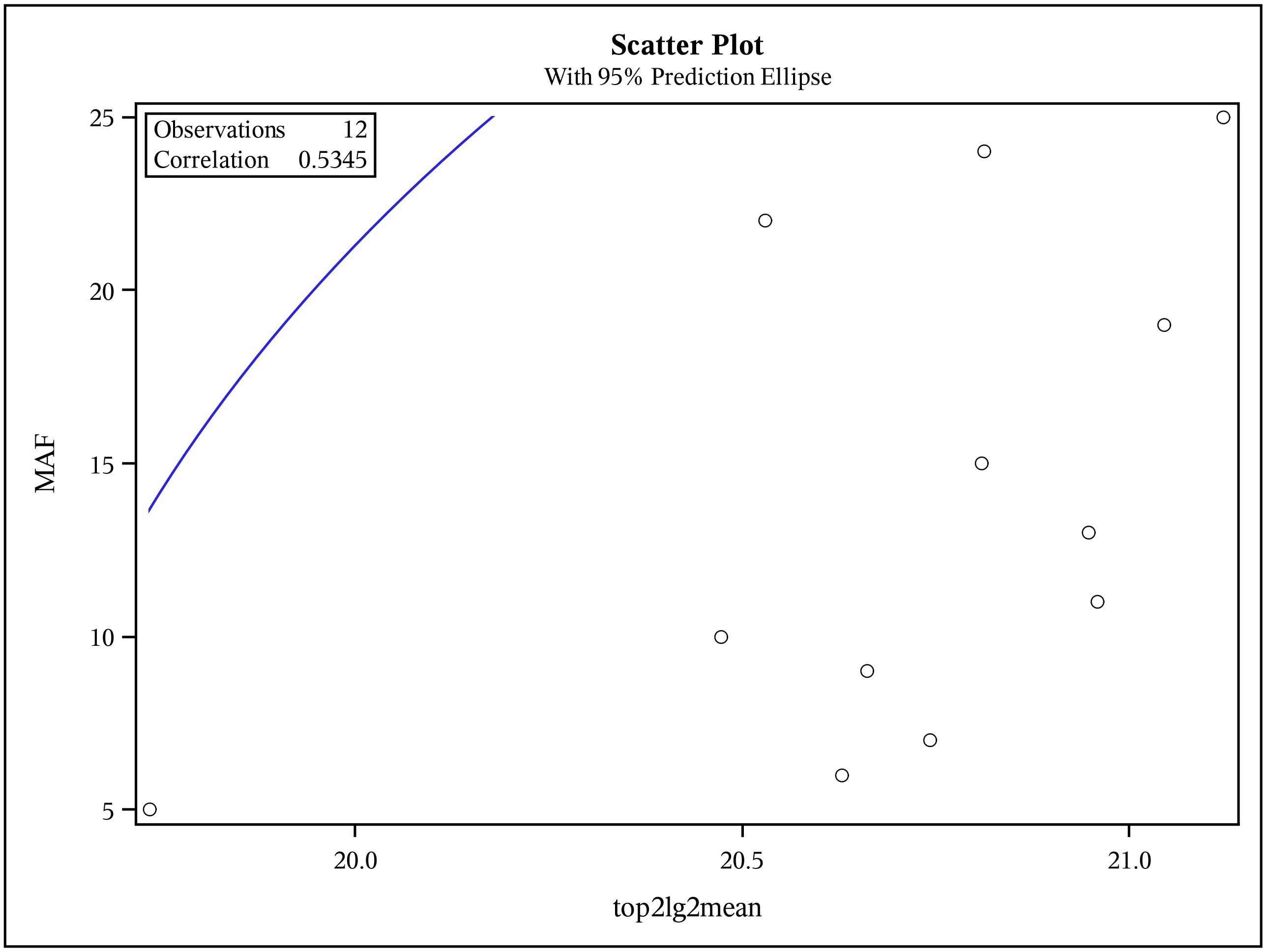

Correlation C_pooltop2lg2mean_tp2 symbol MAF

The CORR Procedure

Gene_Symbol=CFB

**2 Variables:**	top2lg2mean MAF

Simple Statistics						
**Variable**	**N**	**Mean**	**Std Dev**	**Sum**	**Minimum**	**Maximum**
**top2lg2mean**	12	20.10731	0.36953	241.28777	19.43929	20.88184
**MAF**	12	13.83333	7.10740	166.00000	5.00000	25.00000

Pearson Correlation Coefficients, N = 12<br>Prob > |r| under H0: Rho=0		
	**top2lg2mean**	**MAF**
**top2lg2mean**	1.00000	-0.18351<br>0.5681
**MAF**	-0.18351<br>0.5681	1.00000

Pearson Correlation Statistics (Fisher's z Transformation)									
**Variable**	**With Variable**	**N**	**Sample Correlation**	**Fisher's z**	**Bias Adjustment**	**Correlation Estimate**	**95% Confidence Limits**		**p Value for H0:Rho=0**
**top2lg2mean**	**MAF**	12	-0.18351	-0.18561	-0.00834	-0.17544	-0.680795	0.443073	0.5776

Correlation C_pooltop2lg2mean_tp2 symbol MAF

The CORR Procedure

Gene_Symbol=CFB

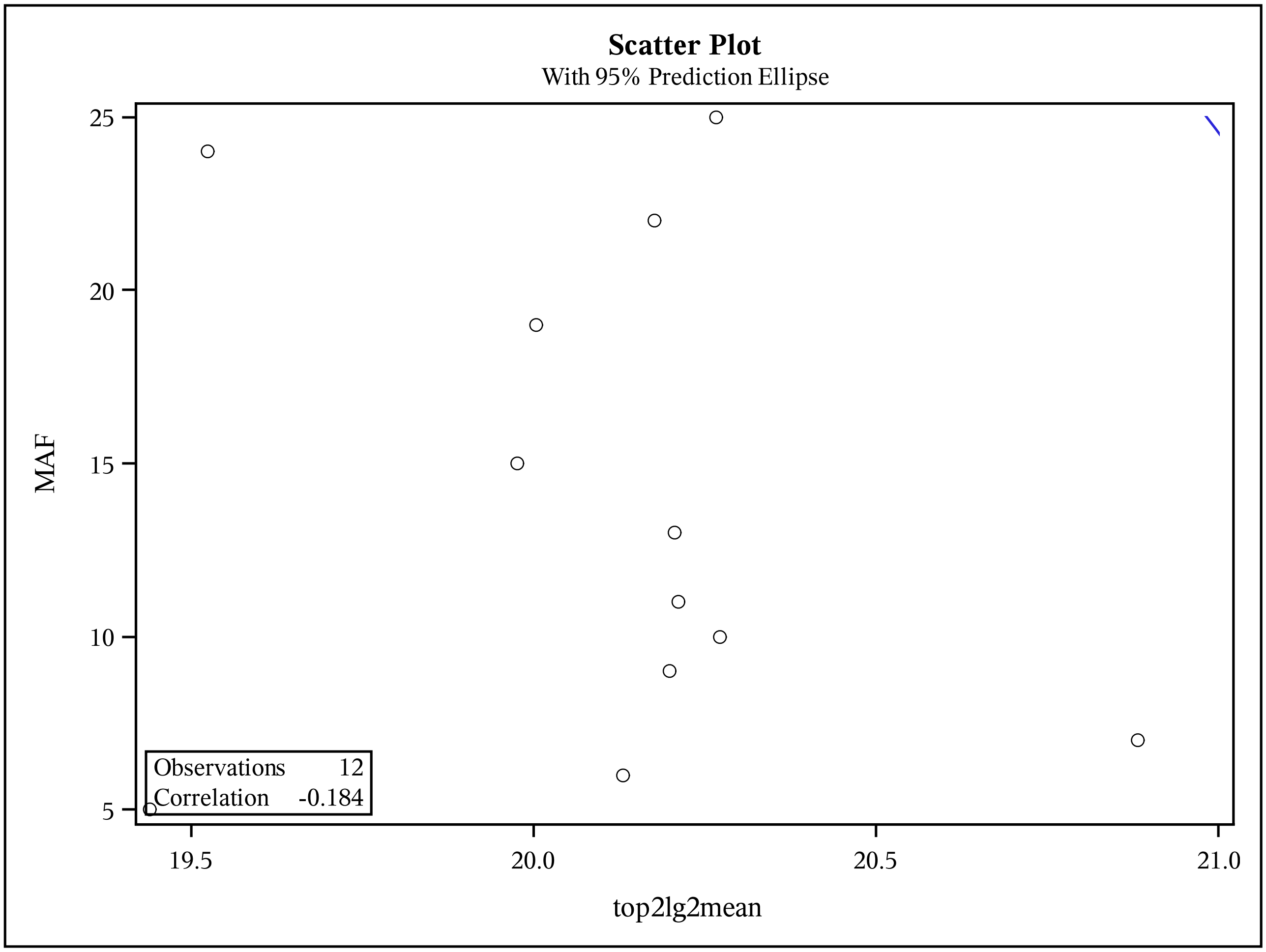

Correlation C_pooltop2lg2mean_tp2 symbol MAF

The CORR Procedure

Gene_Symbol=CFH

**2 Variables:**	top2lg2mean MAF

Simple Statistics						
**Variable**	**N**	**Mean**	**Std Dev**	**Sum**	**Minimum**	**Maximum**
**top2lg2mean**	12	20.28654	0.23510	243.43848	19.71553	20.55740
**MAF**	12	13.83333	7.10740	166.00000	5.00000	25.00000

Pearson Correlation Coefficients, N = 12<br>Prob > |r| under H0: Rho=0		
	**top2lg2mean**	**MAF**
**top2lg2mean**	1.00000	0.48744<br>0.1080
**MAF**	0.48744<br>0.1080	1.00000

Pearson Correlation Statistics (Fisher's z Transformation)									
**Variable**	**With Variable**	**N**	**Sample Correlation**	**Fisher's z**	**Bias Adjustment**	**Correlation Estimate**	**95% Confidence Limits**		**p Value for H0:Rho=0**
**top2lg2mean**	**MAF**	12	0.48744	0.53269	0.02216	0.47036	-0.141821	0.822294	0.1100

Correlation C_pooltop2lg2mean_tp2 symbol MAF

The CORR Procedure

Gene_Symbol=CFH

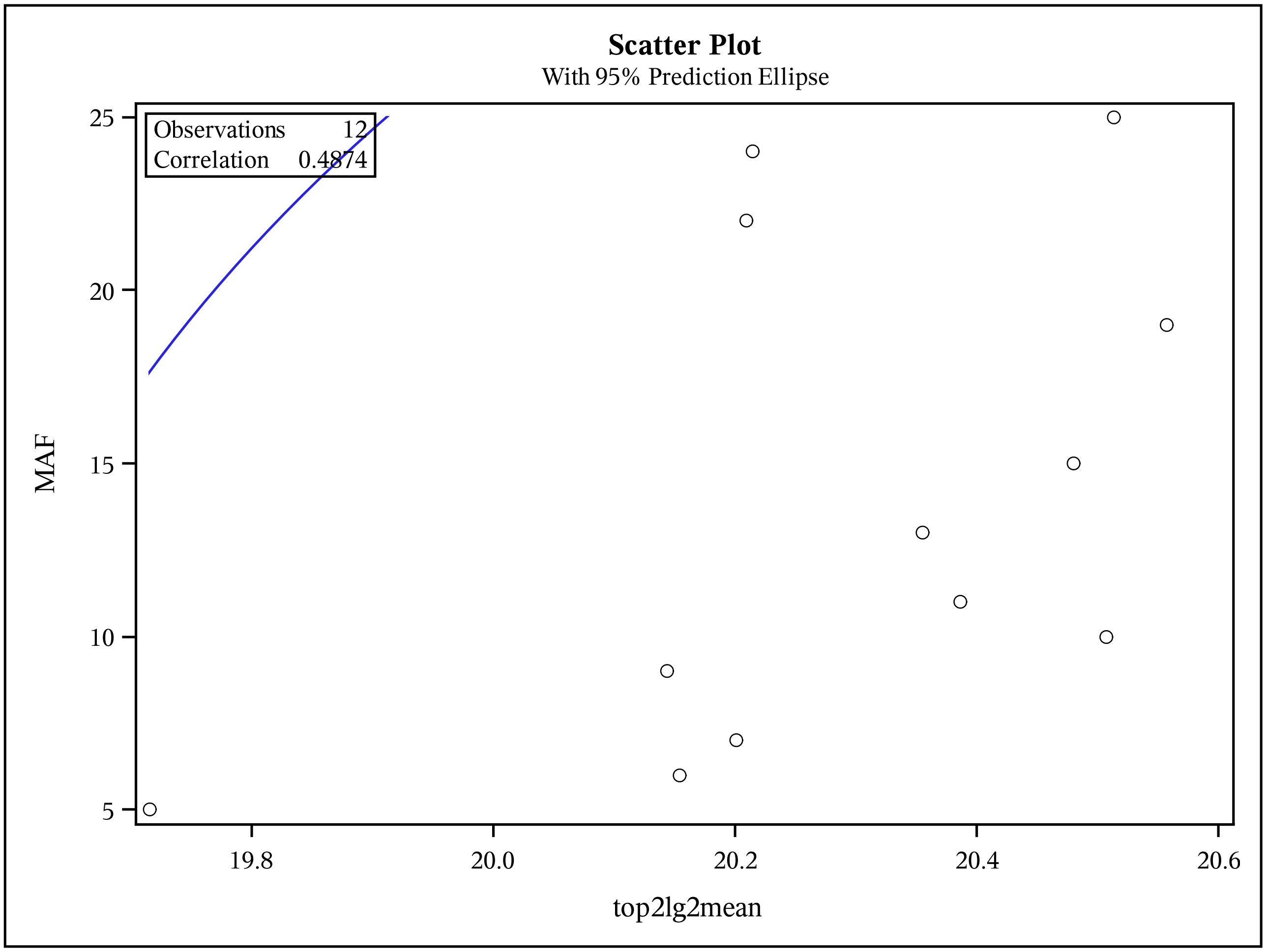

Correlation C_pooltop2lg2mean_tp2 symbol MAF

The CORR Procedure

Gene_Symbol=CHGA

2 Variables:	top2lg2mean MAF

Simple Statistics						
Variable	N	Mean	Std Dev	Sum	Minimum	Maximum
top2lg2mean	12	22.61559	0.18595	271.38709	22.31285	22.88093
MAF	12	13.83333	7.10740	166.00000	5.00000	25.00000

Pearson Correlation Coefficients, N = 12<br>Prob > |r| under H0: Rho=0		
	top2lg2mean	MAF
top2lg2mean	1.00000	0.26584<br>0.4036
MAF	0.26584<br>0.4036	1.00000

Pearson Correlation Statistics (Fisher's z Transformation)									
Variable	With Variable	N	Sample Correlation	Fisher's z	Bias Adjustment	Correlation Estimate	95% Confidence Limits		p Value for H0:Rho=0
top2lg2mean	MAF	12	0.26584	0.27238	0.01208	0.25457	-0.373967	0.722863	0.4139

Correlation C_pooltop2lg2mean_tp2 symbol MAF

The CORR Procedure

Gene_Symbol=CHGA

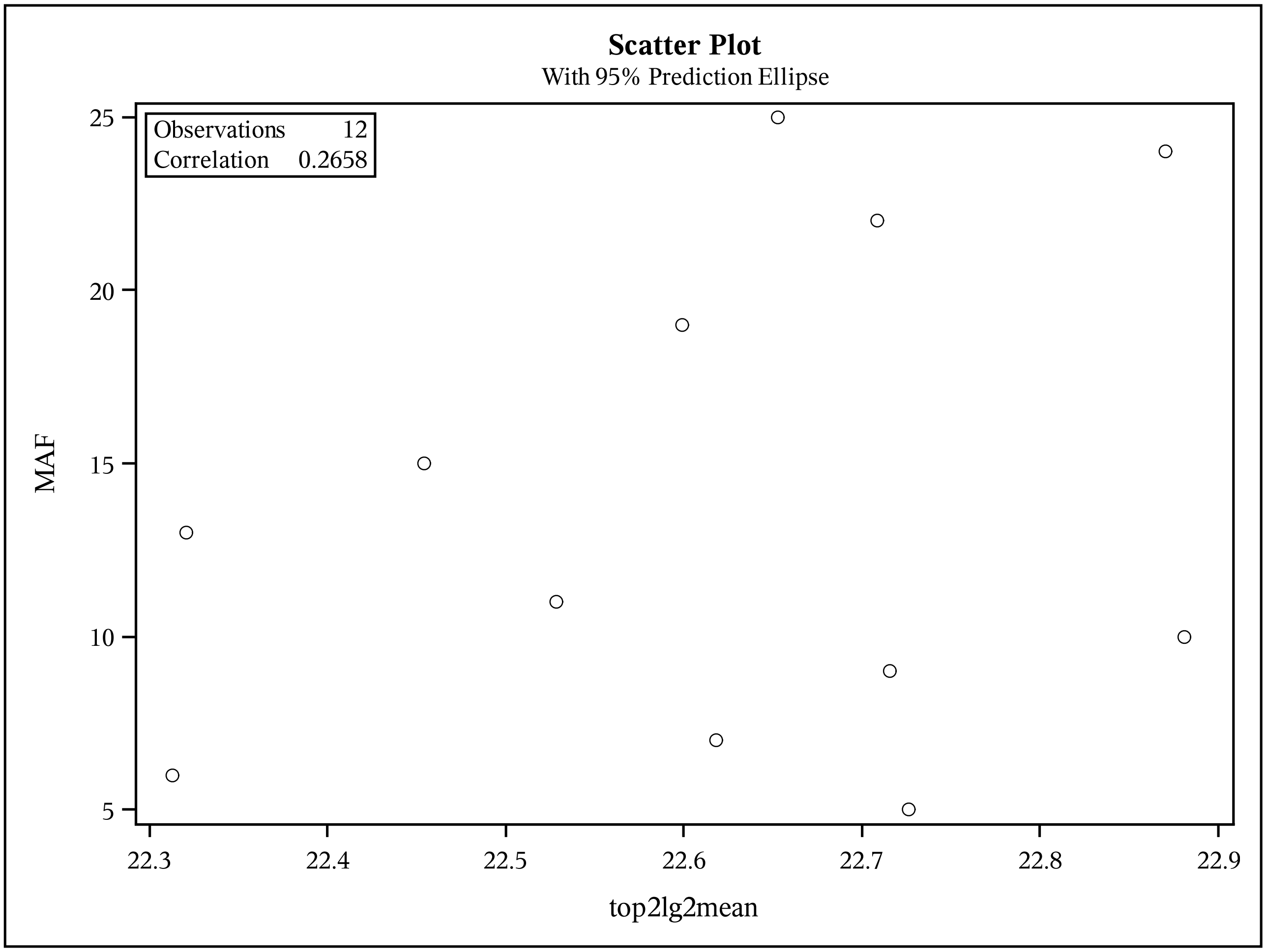

Correlation C_pooltop2lg2mean_tp2 symbol MAF

The CORR Procedure

Gene_Symbol=CHGB

**2 Variables:**	top2lg2mean MAF

Simple Statistics						
**Variable**	**N**	**Mean**	**Std Dev**	**Sum**	**Minimum**	**Maximum**
**top2lg2mean**	12	22.22482	0.18428	266.69786	21.95818	22.55311
**MAF**	12	13.83333	7.10740	166.00000	5.00000	25.00000

Pearson Correlation Coefficients, N = 12<br>Prob > |r| under H0: Rho=0		
	**top2lg2mean**	**MAF**
**top2lg2mean**	1.00000	0.70940<br>0.0098
**MAF**	0.70940<br>0.0098	1.00000

Pearson Correlation Statistics (Fisher's z Transformation)									
**Variable**	**With Variable**	**N**	**Sample Correlation**	**Fisher's z**	**Bias Adjustment**	**Correlation Estimate**	**95% Confidence Limits**		**p Value for H0:Rho=0**
**top2lg2mean**	**MAF**	12	0.70940	0.88597	0.03225	0.69301	0.197765	0.906414	0.0079

Correlation C_pooltop2lg2mean_tp2 symbol MAF

The CORR Procedure

Gene_Symbol=CHGB

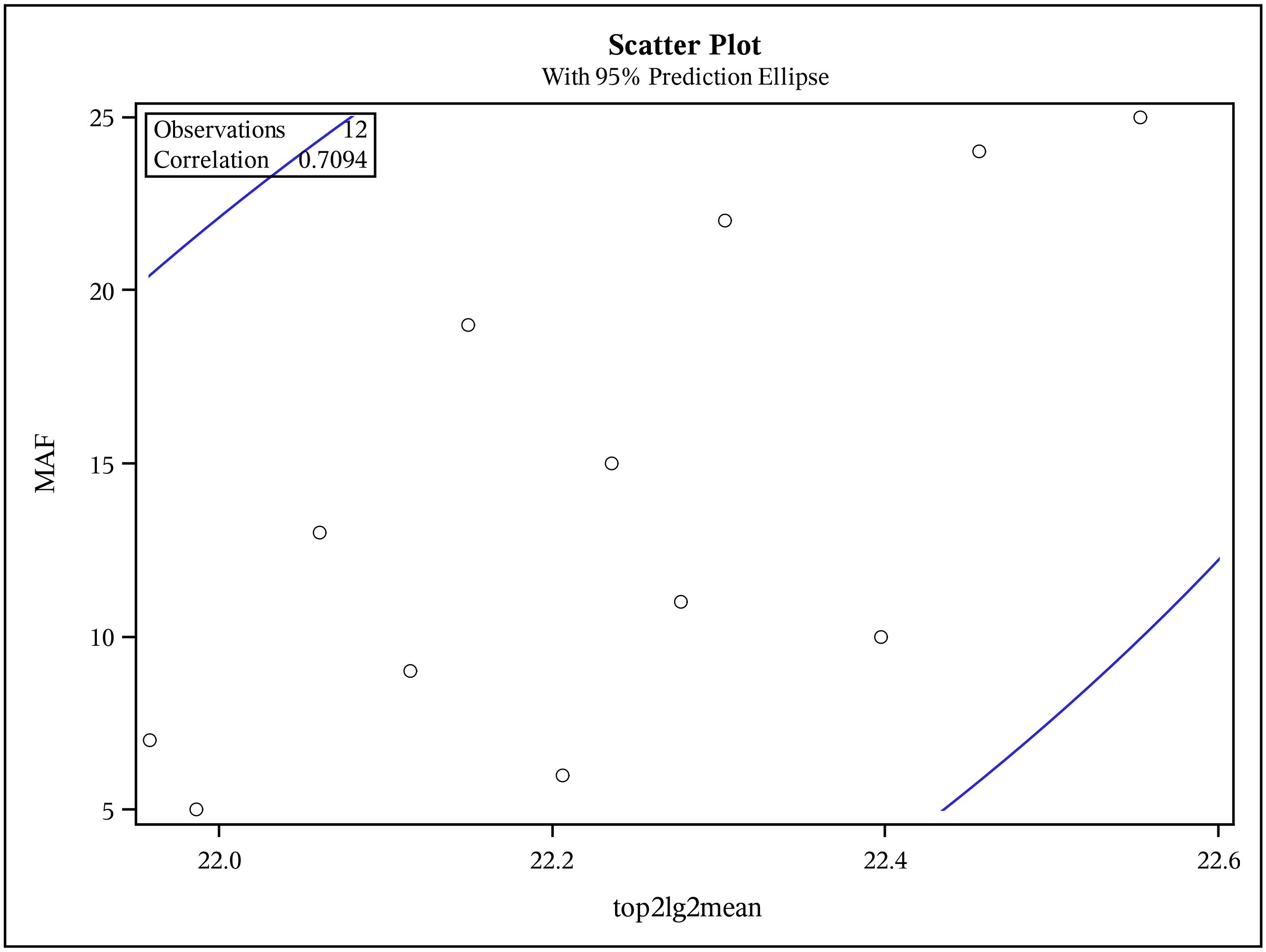

Correlation C_pooltop2lg2mean_tp2 symbol MAF

The CORR Procedure

Gene_Symbol=CHL1

2 Variables:	top2lg2mean MAF

Simple Statistics						
Variable	N	Mean	Std Dev	Sum	Minimum	Maximum
top2lg2mean	12	20.32975	0.64128	243.95697	19.43727	21.37758
MAF	12	13.83333	7.10740	166.00000	5.00000	25.00000

Pearson Correlation Coefficients, N = 12<br>Prob > |r| under H0: Rho=0		
	top2lg2mean	MAF
top2lg2mean	1.00000	0.26241<br>0.4100
MAF	0.26241<br>0.4100	1.00000

Pearson Correlation Statistics (Fisher's z Transformation)									
Variable	With Variable	N	Sample Correlation	Fisher's z	Bias Adjustment	Correlation Estimate	95% Confidence Limits		p Value for H0:Rho=0
top2lg2mean	MAF	12	0.26241	0.26870	0.01193	0.25127	-0.376993	0.721177	0.4202

Correlation C_pooltop2lg2mean_tp2 symbol MAF

The CORR Procedure

Gene_Symbol=CHL1

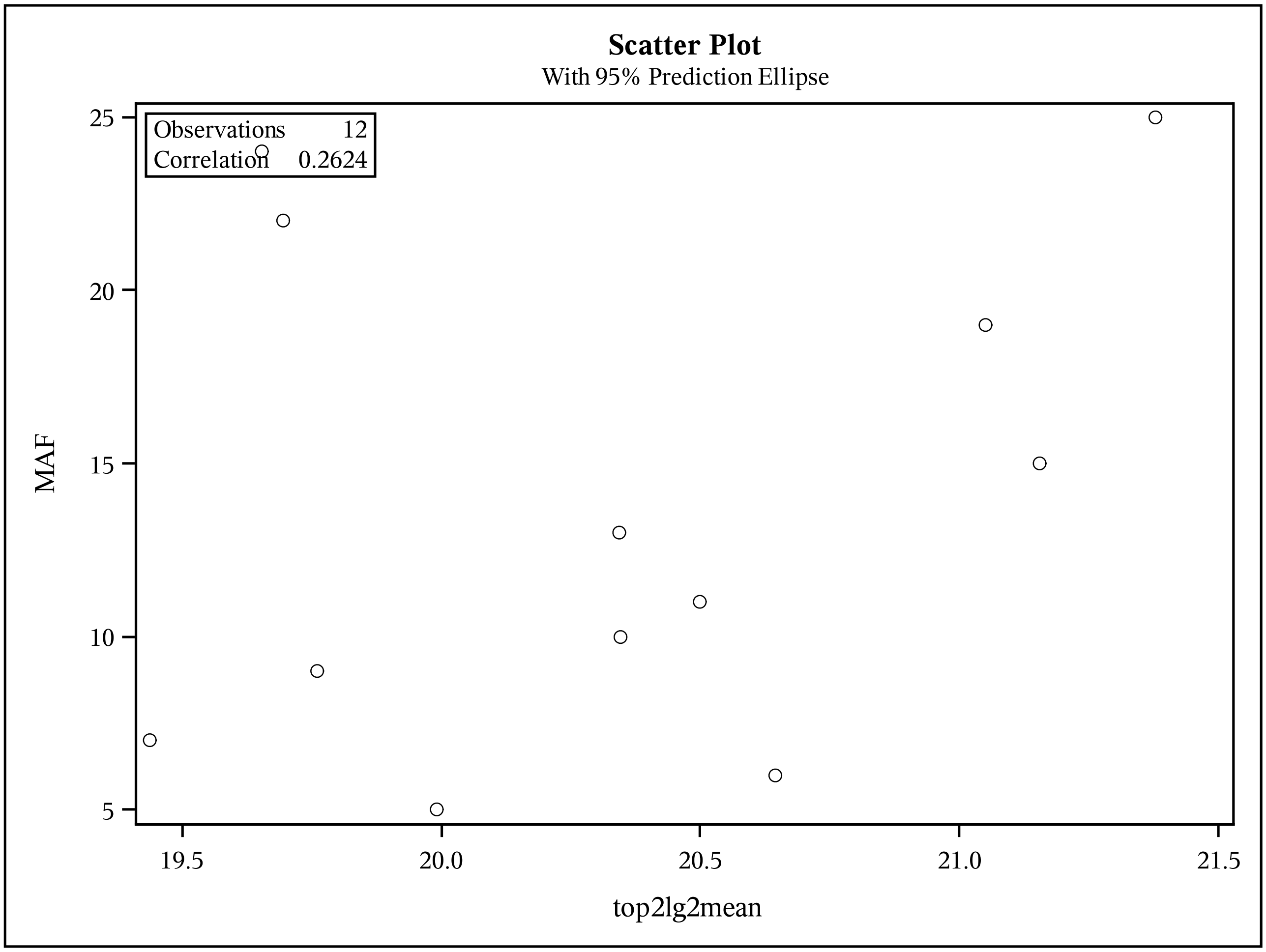

Correlation C_pooltop2lg2mean_tp2 symbol MAF

The CORR Procedure

Gene_Symbol=CLU

**2 Variables:**	top2lg2mean MAF

Simple Statistics						
**Variable**	**N**	**Mean**	**Std Dev**	**Sum**	**Minimum**	**Maximum**
**top2lg2mean**	12	24.56620	0.25894	294.79441	24.03016	24.87867
**MAF**	12	13.83333	7.10740	166.00000	5.00000	25.00000

Pearson Correlation Coefficients, N = 12<br>Prob > |r| under H0: Rho=0		
	**top2lg2mean**	**MAF**
**top2lg2mean**	1.00000	-0.03388<br>0.9168
**MAF**	-0.03388<br>0.9168	1.00000

Pearson Correlation Statistics (Fisher's z Transformation)									
**Variable**	**With Variable**	**N**	**Sample Correlation**	**Fisher's z**	**Bias Adjustment**	**Correlation Estimate**	**95% Confidence Limits**		**p Value for H0:Rho=0**
**top2lg2mean**	**MAF**	12	-0.03388	-0.03389	-0.00154	-0.03234	-0.595193	0.551805	0.9190

Correlation C_pooltop2lg2mean_tp2 symbol MAF

The CORR Procedure

Gene_Symbol=CLU

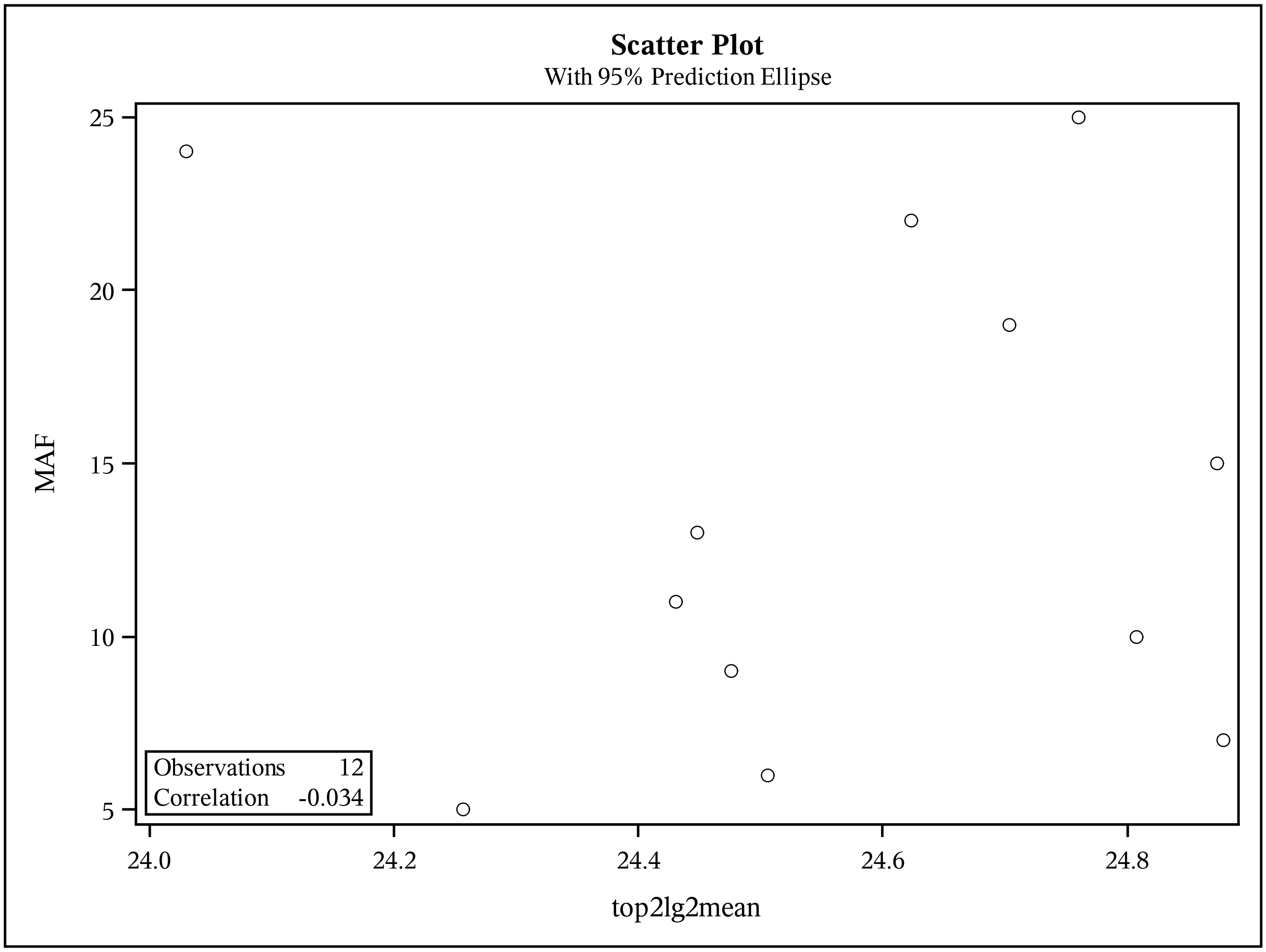

Correlation C_pooltop2lg2mean_tp2 symbol MAF

The CORR Procedure

Gene_Symbol=CNDP1

**2 Variables:**	top2lg2mean MAF

Simple Statistics						
**Variable**	**N**	**Mean**	**Std Dev**	**Sum**	**Minimum**	**Maximum**
**top2lg2mean**	12	22.80093	0.11438	273.61114	22.58143	22.93465
**MAF**	12	13.83333	7.10740	166.00000	5.00000	25.00000

Pearson Correlation Coefficients, N = 12<br>Prob > |r| under H0: Rho=0		
	**top2lg2mean**	**MAF**
**top2lg2mean**	1.00000	-0.05252<br>0.8712
**MAF**	-0.05252<br>0.8712	1.00000

Pearson Correlation Statistics (Fisher's z Transformation)									
**Variable**	**With Variable**	**N**	**Sample Correlation**	**Fisher's z**	**Bias Adjustment**	**Correlation Estimate**	**95% Confidence Limits**		**p Value for H0:Rho=0**
**top2lg2mean**	**MAF**	12	-0.05252	-0.05257	-0.00239	-0.05014	-0.606588	0.539279	0.8747

Correlation C_pooltop2lg2mean_tp2 symbol MAF

The CORR Procedure

Gene_Symbol=CNDP1

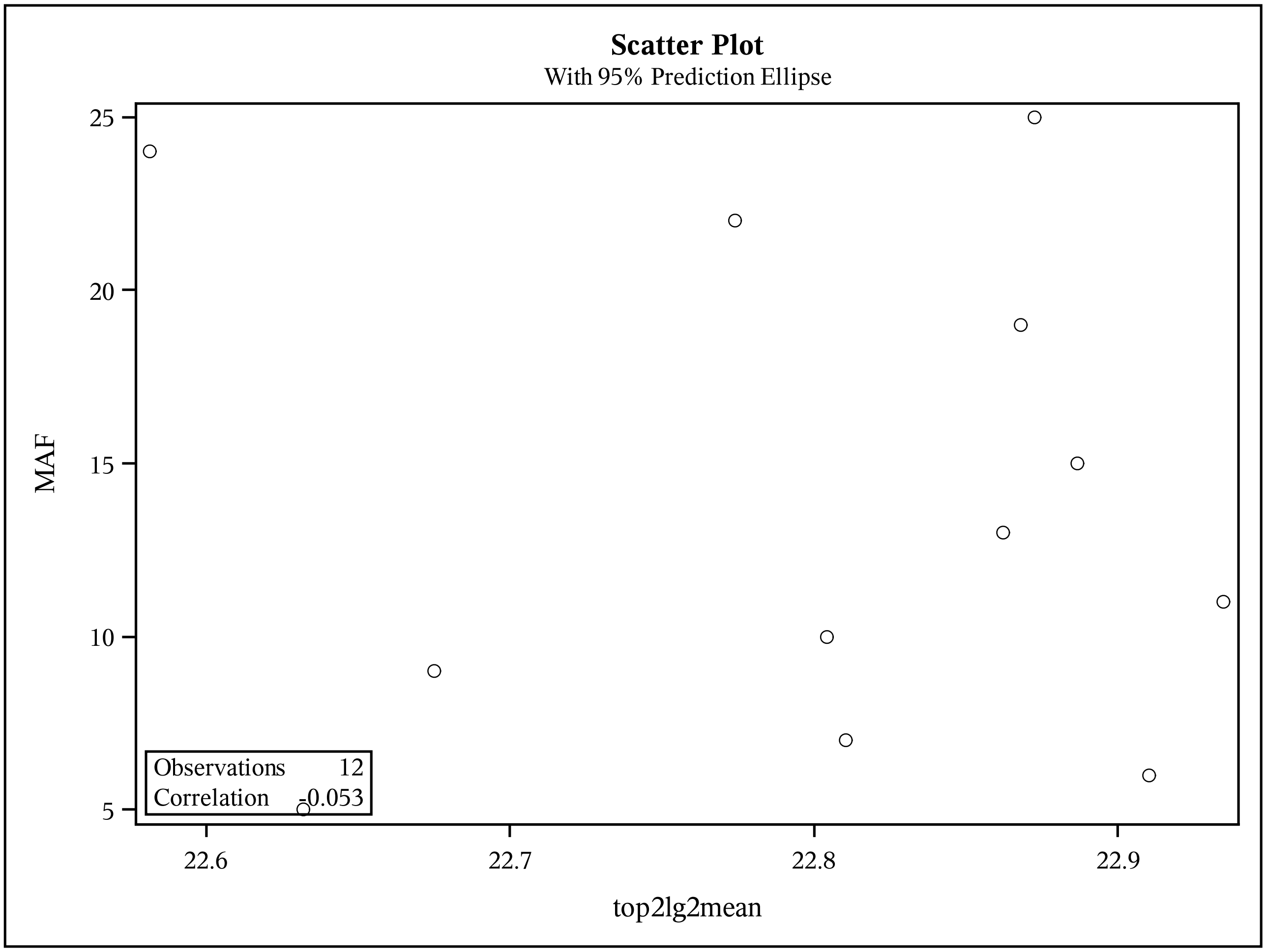

Correlation C_pooltop2lg2mean_tp2 symbol MAF

The CORR Procedure

Gene_Symbol=CNTN1

**2 Variables:**	top2lg2mean MAF

Simple Statistics						
**Variable**	**N**	**Mean**	**Std Dev**	**Sum**	**Minimum**	**Maximum**
**top2lg2mean**	12	20.00227	0.16488	240.02726	19.59967	20.23726
**MAF**	12	13.83333	7.10740	166.00000	5.00000	25.00000

Pearson Correlation Coefficients, N = 12<br>Prob > |r| under H0: Rho=0		
	**top2lg2mean**	**MAF**
**top2lg2mean**	1.00000	0.48987<br>0.1060
**MAF**	0.48987<br>0.1060	1.00000

Pearson Correlation Statistics (Fisher's z Transformation)									
**Variable**	**With Variable**	**N**	**Sample Correlation**	**Fisher's z**	**Bias Adjustment**	**Correlation Estimate**	**95% Confidence Limits**		**p Value for H0:Rho=0**
**top2lg2mean**	**MAF**	12	0.48987	0.53589	0.02227	0.47276	-0.138798	0.823290	0.1079

Correlation C_pooltop2lg2mean_tp2 symbol MAF

The CORR Procedure

Gene_Symbol=CNTN1

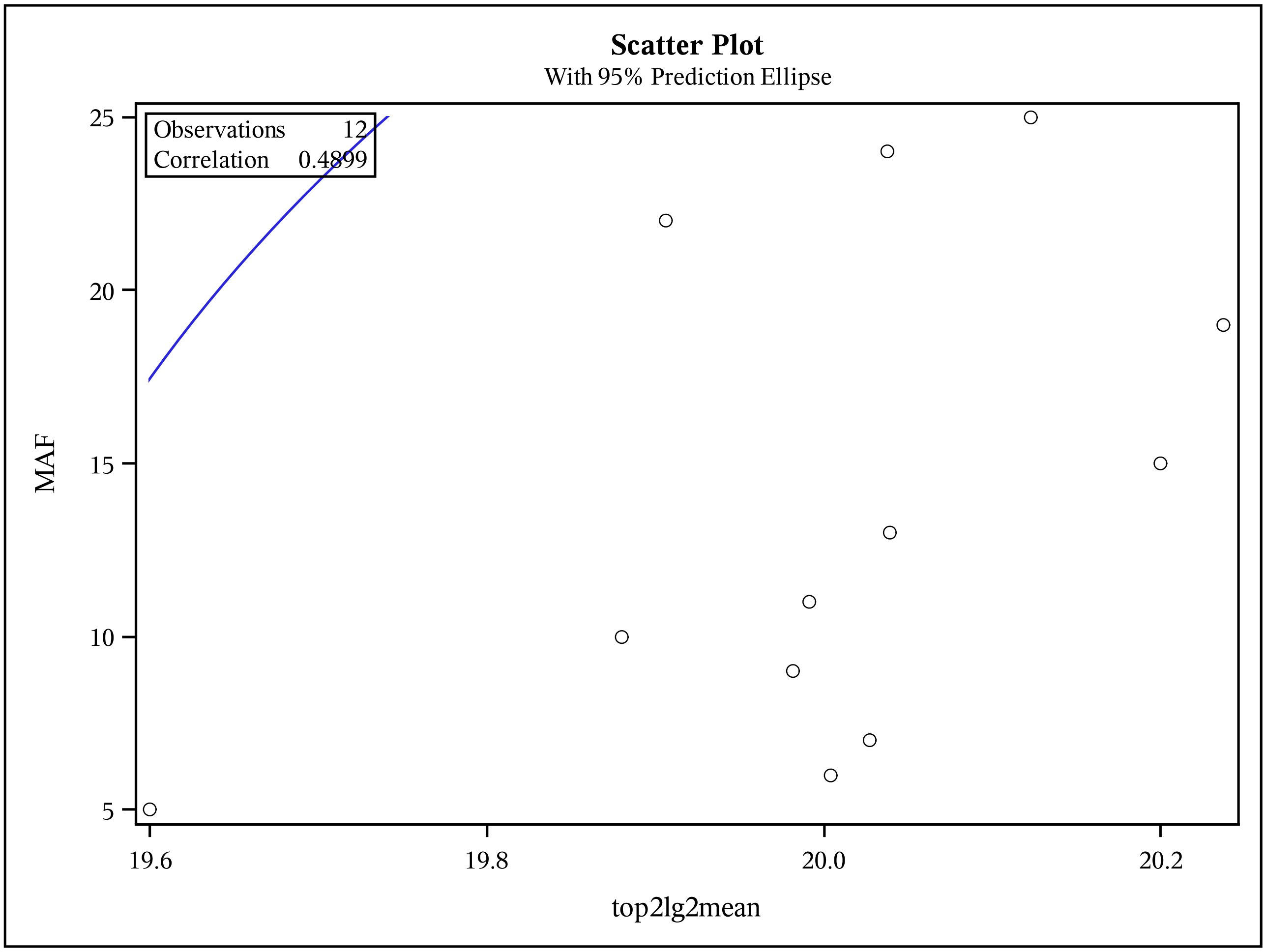

Correlation C_pooltop2lg2mean_tp2 symbol MAF

The CORR Procedure

Gene_Symbol=CP

2 Variables:	top2lg2mean MAF

Simple Statistics						
Variable	N	Mean	Std Dev	Sum	Minimum	Maximum
top2lg2mean	12	22.55661	0.31053	270.67936	22.02298	23.05525
MAF	12	13.83333	7.10740	166.00000	5.00000	25.00000

Pearson Correlation Coefficients, N = 12<br>Prob > |r| under H0: Rho=0		
	top2lg2mean	MAF
top2lg2mean	1.00000	-0.30867<br>0.3290
MAF	-0.30867<br>0.3290	1.00000

Pearson Correlation Statistics (Fisher's z Transformation)									
Variable	With Variable	N	Sample Correlation	Fisher's z	Bias Adjustment	Correlation Estimate	95% Confidence Limits		p Value for H0:Rho=0
top2lg2mean	MAF	12	-0.30867	-0.31907	-0.01403	-0.29592	-0.743545	0.334850	0.3385

Correlation C_pooltop2lg2mean_tp2 symbol MAF

The CORR Procedure

Gene_Symbol=CP

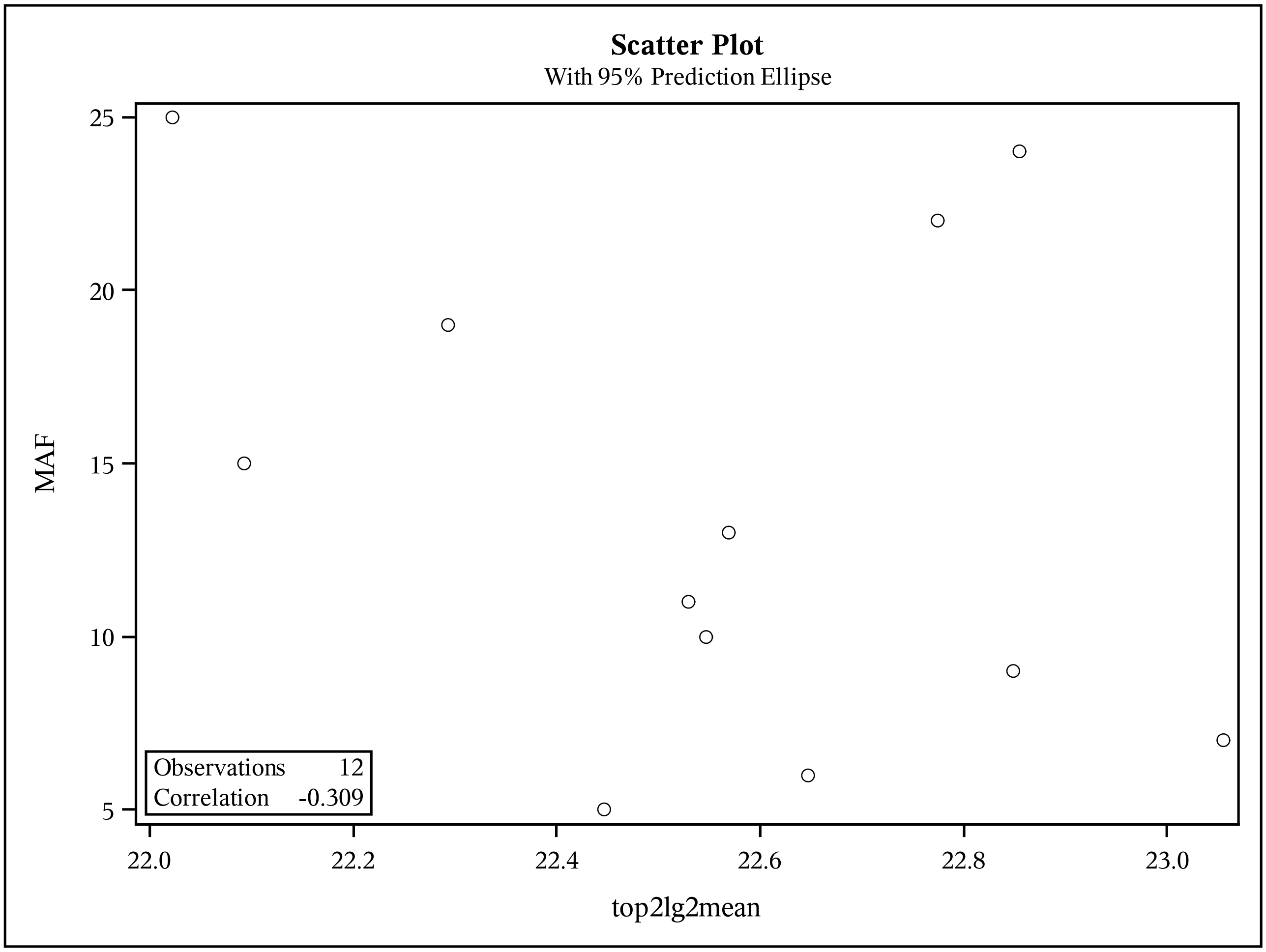

Correlation C_pooltop2lg2mean_tp2 symbol MAF

The CORR Procedure

Gene_Symbol=CPA1

2 Variables:	top2lg2mean MAF

Simple Statistics						
Variable	N	Mean	Std Dev	Sum	Minimum	Maximum
top2lg2mean	12	16.97277	0.37952	203.67329	16.44452	17.86625
MAF	12	13.83333	7.10740	166.00000	5.00000	25.00000

Pearson Correlation Coefficients, N = 12<br>Prob > |r| under H0: Rho=0		
	top2lg2mean	MAF
top2lg2mean	1.00000	-0.08206<br>0.7998
MAF	-0.08206<br>0.7998	1.00000

Pearson Correlation Statistics (Fisher's z Transformation)									
Variable	With Variable	N	Sample Correlation	Fisher's z	Bias Adjustment	Correlation Estimate	95% Confidence Limits		p Value for H0:Rho=0
top2lg2mean	MAF	12	-0.08206	-0.08225	-0.00373	-0.07836	-0.624190	0.518877	0.8051

Correlation C_pooltop2lg2mean_tp2 symbol MAF

The CORR Procedure

Gene_Symbol=CPA1

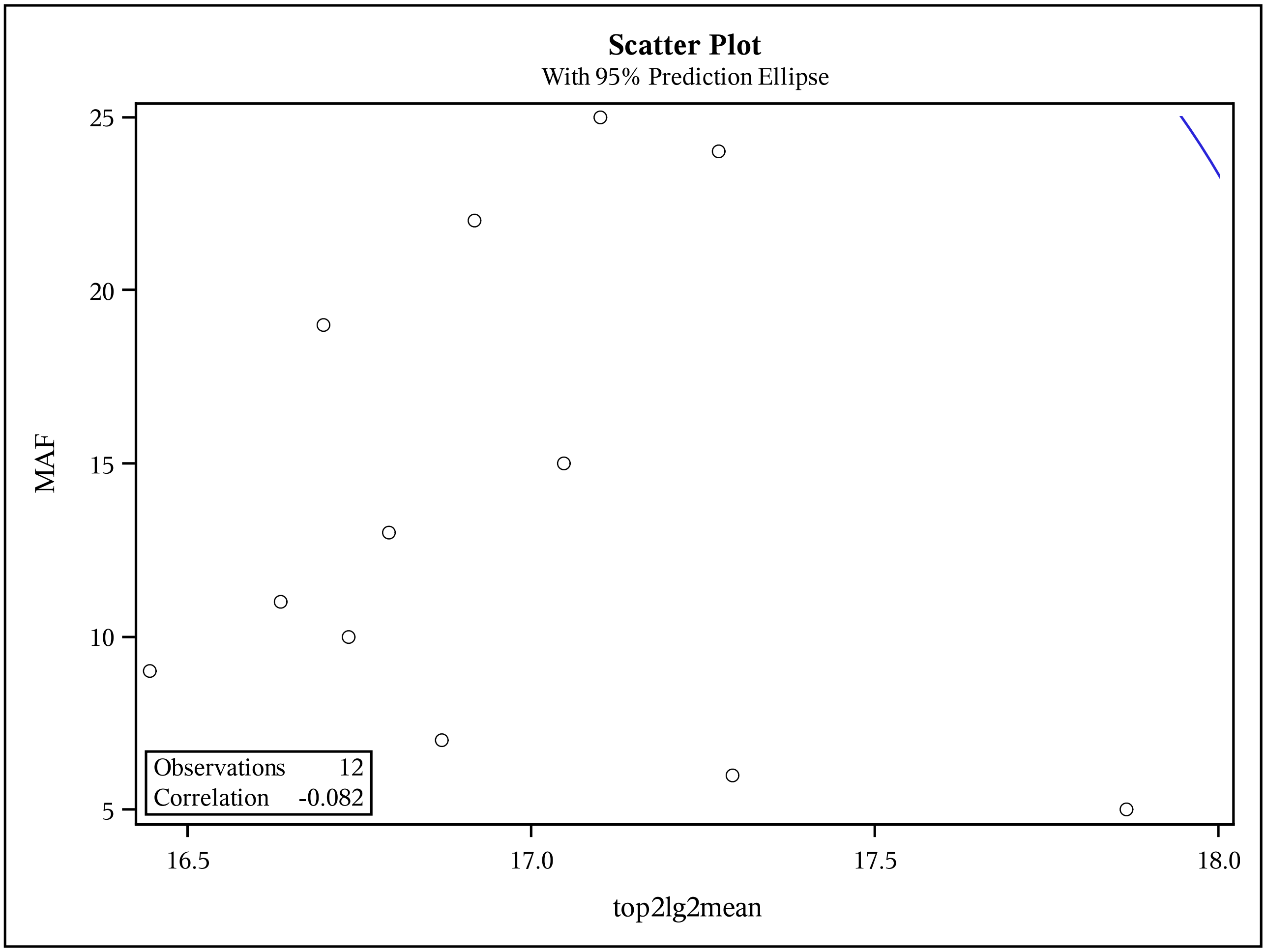

Correlation C_pooltop2lg2mean_tp2 symbol MAF

The CORR Procedure

Gene_Symbol=CPE

**2 Variables:**	top2lg2mean MAF

Simple Statistics						
**Variable**	**N**	**Mean**	**Std Dev**	**Sum**	**Minimum**	**Maximum**
**top2lg2mean**	12	17.11630	0.28200	205.39565	16.67208	17.43015
**MAF**	12	13.83333	7.10740	166.00000	5.00000	25.00000

Pearson Correlation Coefficients, N = 12<br>Prob > |r| under H0: Rho=0		
	**top2lg2mean**	**MAF**
**top2lg2mean**	1.00000	0.06398<br>0.8434
**MAF**	0.06398<br>0.8434	1.00000

Pearson Correlation Statistics (Fisher's z Transformation)									
**Variable**	**With Variable**	**N**	**Sample Correlation**	**Fisher's z**	**Bias Adjustment**	**Correlation Estimate**	**95% Confidence Limits**		**p Value for H0:Rho=0**
**top2lg2mean**	**MAF**	12	0.06398	0.06407	0.00291	0.06108	-0.531450	0.613478	0.8476

Correlation C_pooltop2lg2mean_tp2 symbol MAF

The CORR Procedure

Gene_Symbol=CPE

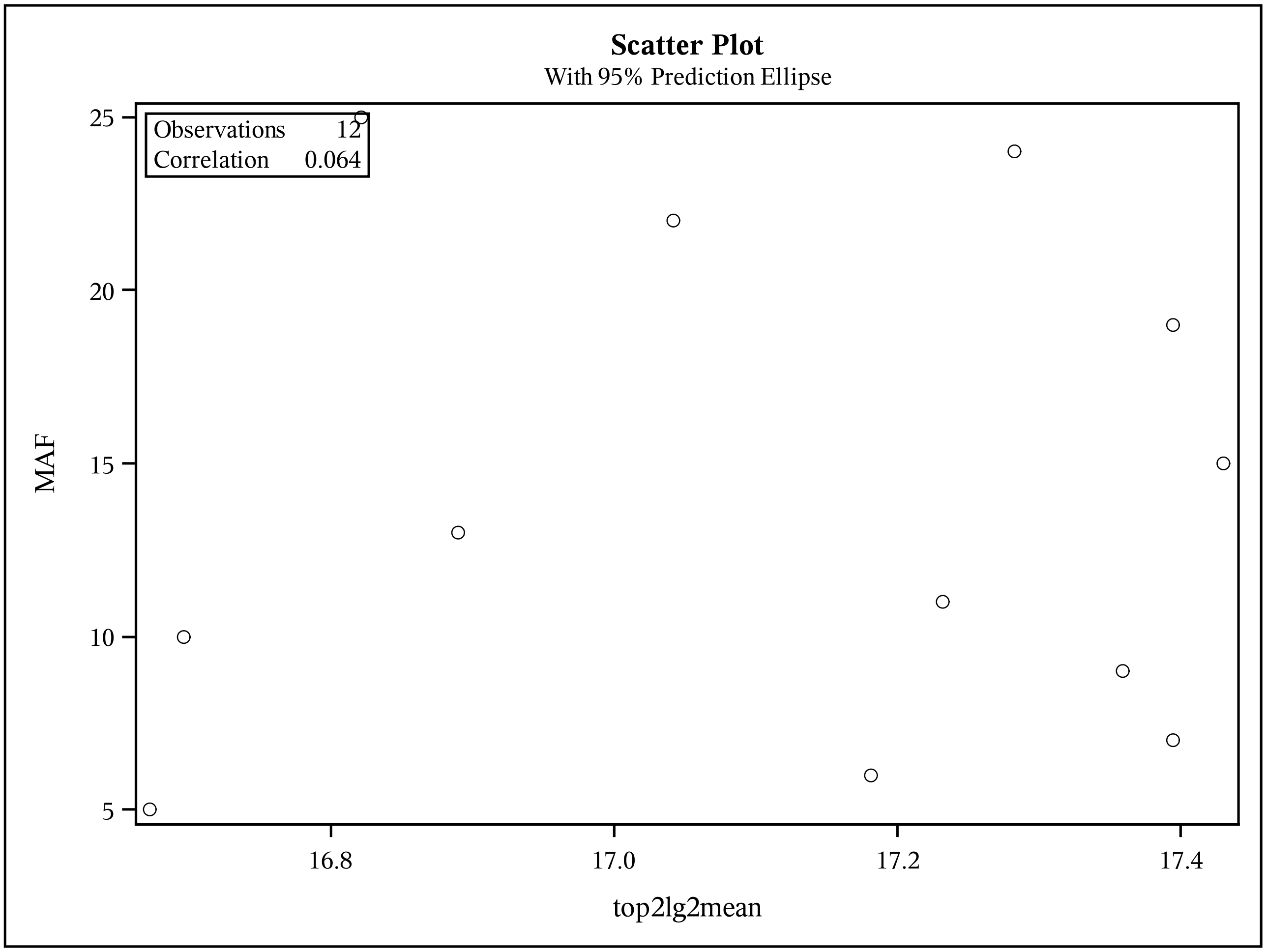

Correlation C_pooltop2lg2mean_tp2 symbol MAF

The CORR Procedure

Gene_Symbol=CRTAC1

2 Variables:	top2lg2mean MAF

Simple Statistics						
**Variable**	**N**	**Mean**	**Std Dev**	**Sum**	**Minimum**	**Maximum**
**top2lg2mean**	12	16.15360	1.94327	193.84315	10.01973	17.06571
**MAF**	12	13.83333	7.10740	166.00000	5.00000	25.00000

Pearson Correlation Coefficients, N = 12<br>Prob > |r| under H0: Rho=0		
	**top2lg2mean**	**MAF**
**top2lg2mean**	1.00000	0.33267<br>0.2907
**MAF**	0.33267<br>0.2907	1.00000

Pearson Correlation Statistics (Fisher's z Transformation)									
**Variable**	**With Variable**	**N**	**Sample Correlation**	**Fisher's z**	**Bias Adjustment**	**Correlation Estimate**	**95% Confidence Limits**		**p Value for H0:Rho=0**
**top2lg2mean**	**MAF**	12	0.33267	0.34582	0.01512	0.31915	-0.311872	0.754803	0.2995

Correlation C_pooltop2lg2mean_tp2 symbol MAF

The CORR Procedure

Gene_Symbol=CRTAC1

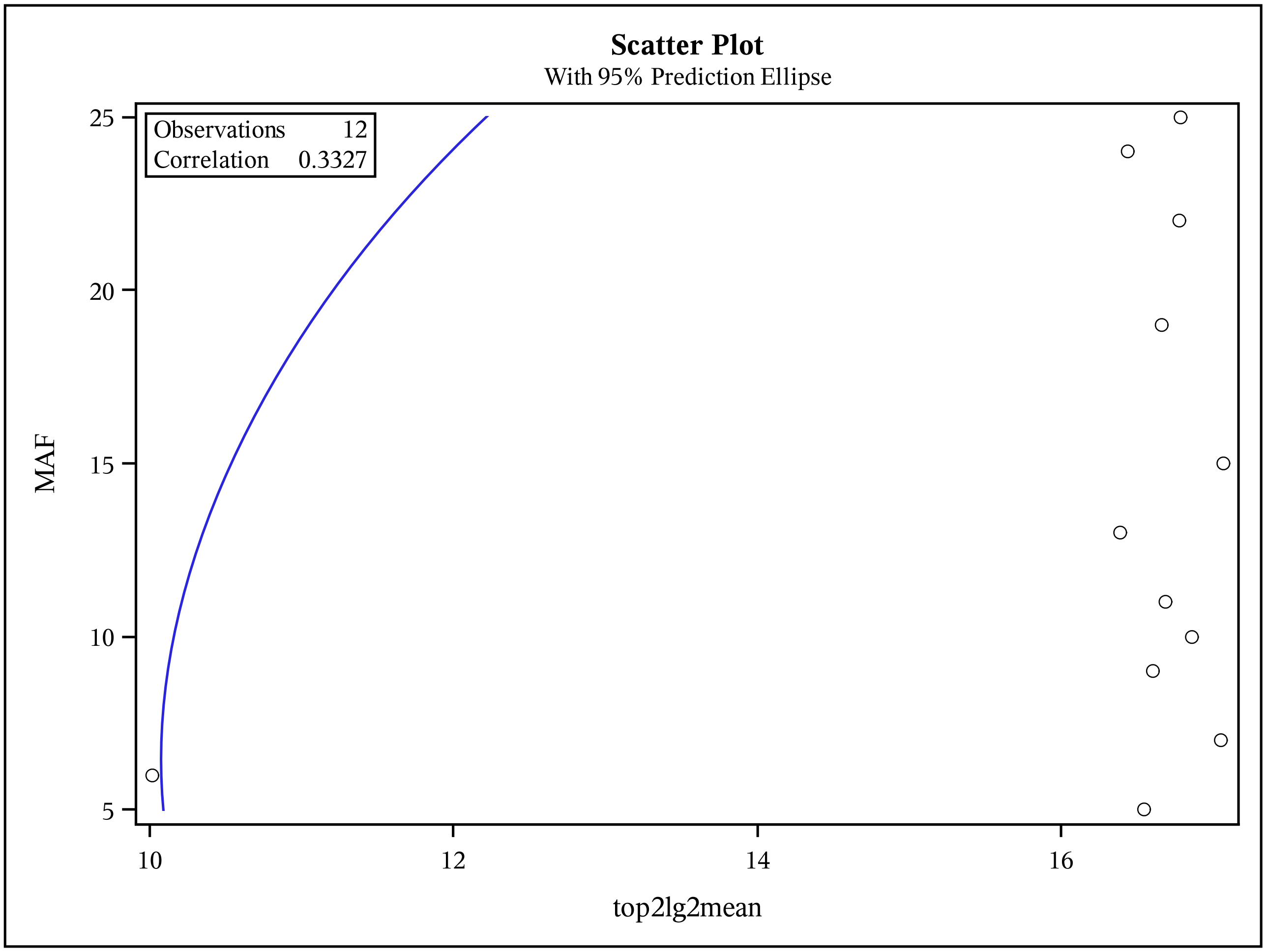

Correlation C_pooltop2lg2mean_tp2 symbol MAF

The CORR Procedure

Gene_Symbol=CST3

**2 Variables:**	top2lg2mean MAF

Simple Statistics						
**Variable**	**N**	**Mean**	**Std Dev**	**Sum**	**Minimum**	**Maximum**
**top2lg2mean**	12	25.23753	0.16033	302.85033	24.95270	25.56438
**MAF**	12	13.83333	7.10740	166.00000	5.00000	25.00000

Pearson Correlation Coefficients, N = 12<br>Prob > |r| under H0: Rho=0		
	**top2lg2mean**	**MAF**
**top2lg2mean**	1.00000	-0.01533<br>0.9623
**MAF**	-0.01533<br>0.9623	1.00000

Pearson Correlation Statistics (Fisher's z Transformation)									
**Variable**	**With Variable**	**N**	**Sample Correlation**	**Fisher's z**	**Bias Adjustment**	**Correlation Estimate**	**95% Confidence Limits**		**p Value for H0:Rho=0**
**top2lg2mean**	**MAF**	12	-0.01533	-0.01533	-0.0006966	-0.01463	-0.583631	0.564008	0.9633

Correlation C_pooltop2lg2mean_tp2 symbol MAF

The CORR Procedure

Gene_Symbol=CST3

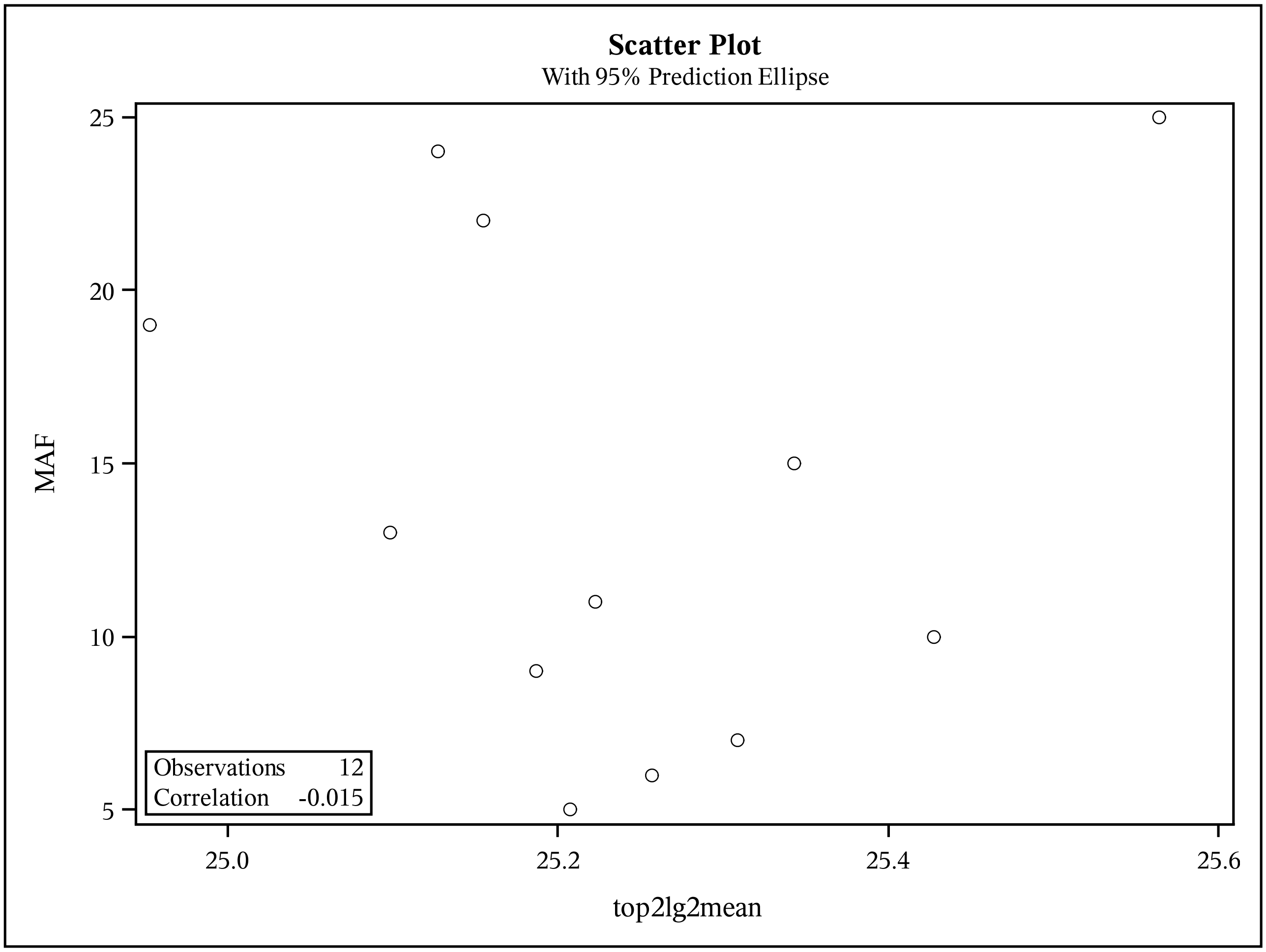

Correlation C_pooltop2lg2mean_tp2 symbol MAF

The CORR Procedure

Gene_Symbol=DKK3

**2 Variables:**	top2lg2mean MAF

Simple Statistics						
**Variable**	**N**	**Mean**	**Std Dev**	**Sum**	**Minimum**	**Maximum**
**top2lg2mean**	12	20.10947	0.79286	241.31363	19.13980	21.78206
**MAF**	12	13.83333	7.10740	166.00000	5.00000	25.00000

Pearson Correlation Coefficients, N = 12<br>Prob > |r| under H0: Rho=0		
	**top2lg2mean**	**MAF**
**top2lg2mean**	1.00000	-0.06304<br>0.8457
**MAF**	-0.06304<br>0.8457	1.00000

Pearson Correlation Statistics (Fisher's z Transformation)									
**Variable**	**With Variable**	**N**	**Sample Correlation**	**Fisher's z**	**Bias Adjustment**	**Correlation Estimate**	**95% Confidence Limits**		**p Value for H0:Rho=0**
**top2lg2mean**	**MAF**	12	-0.06304	-0.06312	-0.00287	-0.06018	-0.612915	0.532097	0.8498

Correlation C_pooltop2lg2mean_tp2 symbol MAF

The CORR Procedure

Gene_Symbol=DKK3

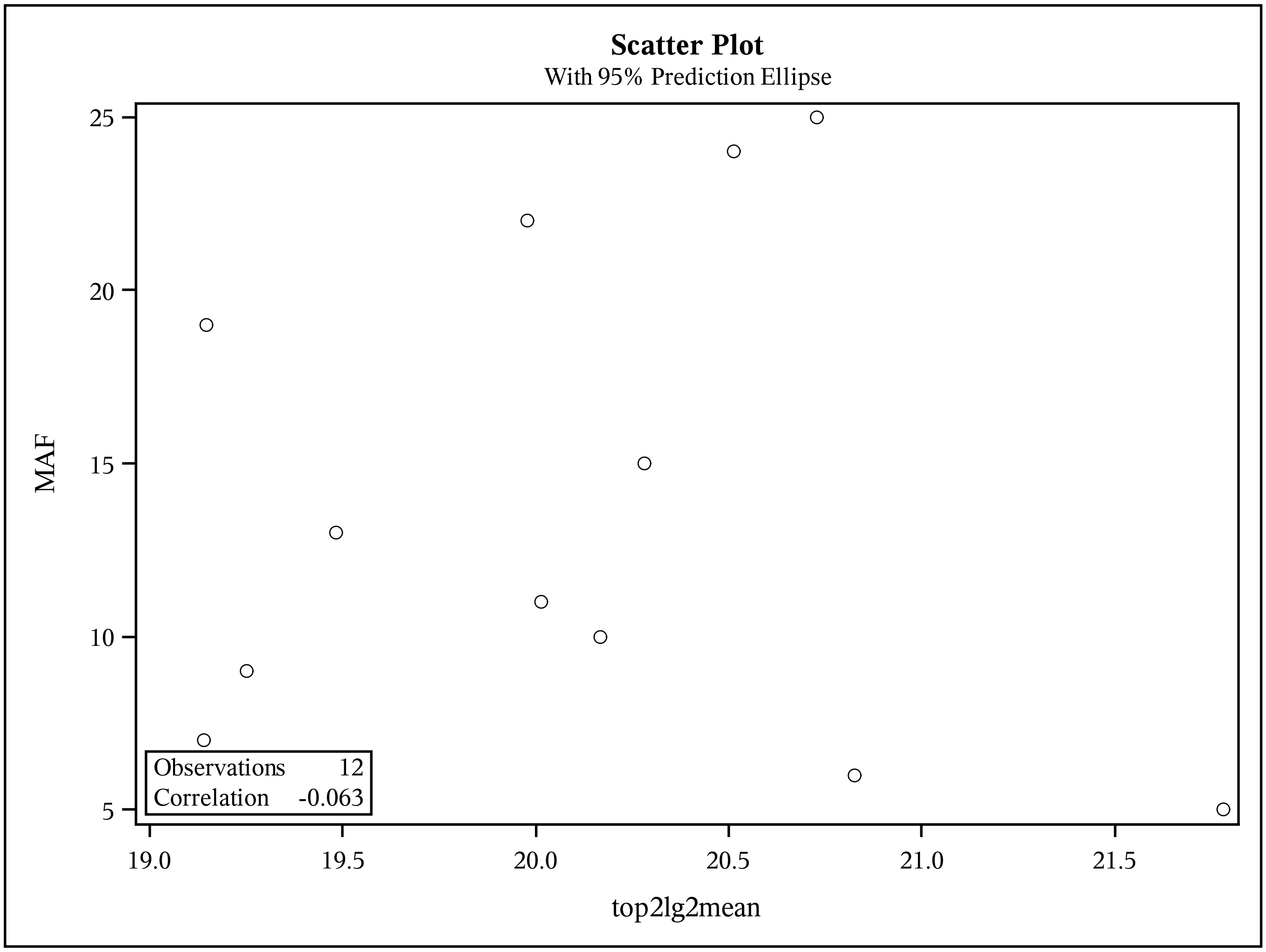

Correlation C_pooltop2lg2mean_tp2 symbol MAF

The CORR Procedure

Gene_Symbol=EFEMP1

**2 Variables:**	top2lg2mean MAF

Simple Statistics						
**Variable**	**N**	**Mean**	**Std Dev**	**Sum**	**Minimum**	**Maximum**
**top2lg2mean**	12	20.60120	0.23123	247.21445	20.22609	21.07743
**MAF**	12	13.83333	7.10740	166.00000	5.00000	25.00000

Pearson Correlation Coefficients, N = 12<br>Prob > |r| under H0: Rho=0		
	**top2lg2mean**	**MAF**
**top2lg2mean**	1.00000	0.06308<br>0.8456
**MAF**	0.06308<br>0.8456	1.00000

Pearson Correlation Statistics (Fisher's z Transformation)									
**Variable**	**With Variable**	**N**	**Sample Correlation**	**Fisher's z**	**Bias Adjustment**	**Correlation Estimate**	**95% Confidence Limits**		**p Value for H0:Rho=0**
**top2lg2mean**	**MAF**	12	0.06308	0.06316	0.00287	0.06022	-0.532069	0.612939	0.8497

Correlation C_pooltop2lg2mean_tp2 symbol MAF

The CORR Procedure

Gene_Symbol=EFEMP1

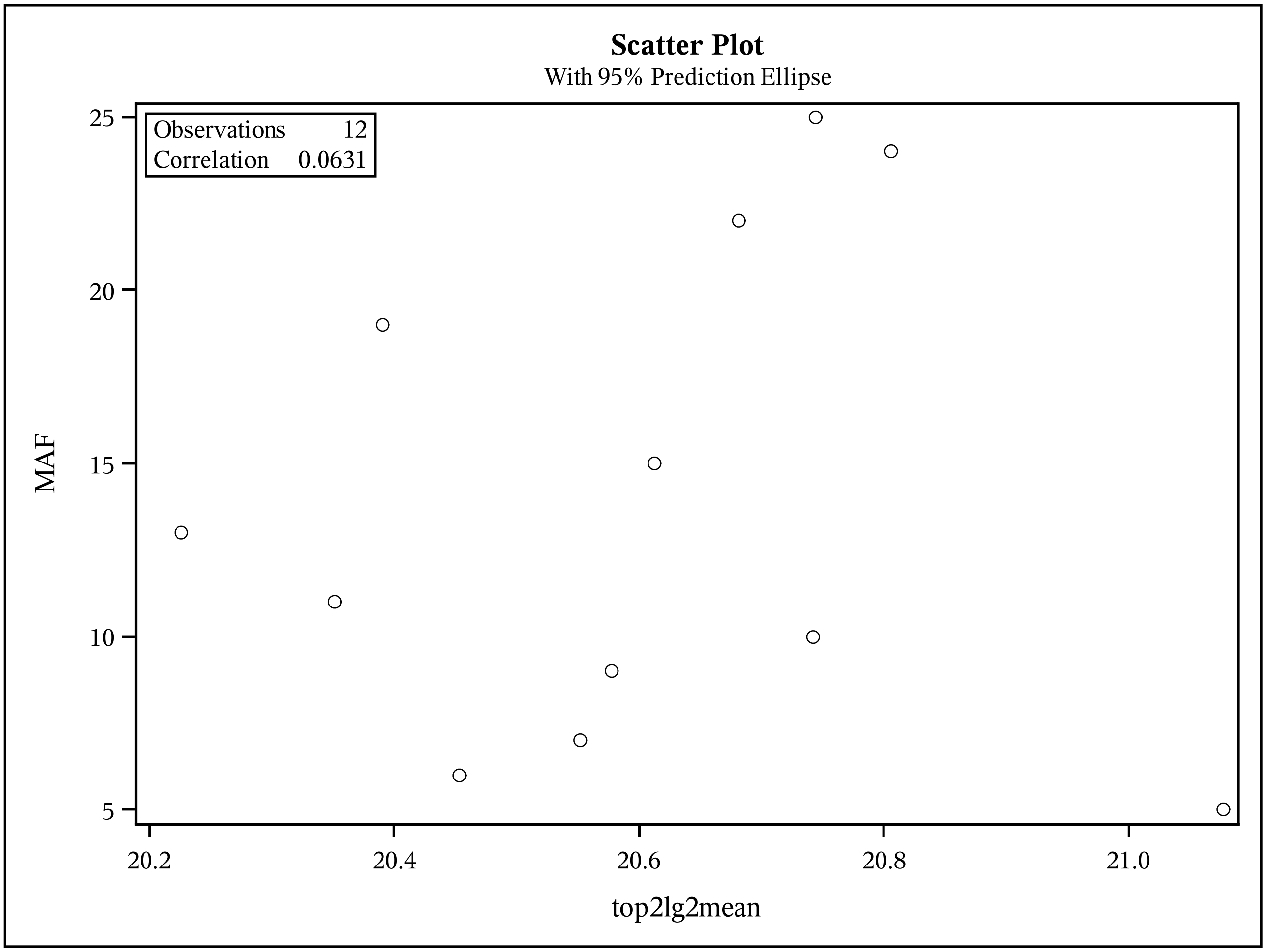

Correlation C_pooltop2lg2mean_tp2 symbol MAF

The CORR Procedure

Gene_Symbol=ENPP2

**2 Variables:**	top2lg2mean MAF

Simple Statistics						
**Variable**	**N**	**Mean**	**Std Dev**	**Sum**	**Minimum**	**Maximum**
**top2lg2mean**	12	20.07433	0.89659	240.89194	18.69225	21.72345
**MAF**	12	13.83333	7.10740	166.00000	5.00000	25.00000

Pearson Correlation Coefficients, N = 12<br>Prob > |r| under H0: Rho=0		
	**top2lg2mean**	**MAF**
**top2lg2mean**	1.00000	-0.32116<br>0.3087
**MAF**	-0.32116<br>0.3087	1.00000

Pearson Correlation Statistics (Fisher's z Transformation)									
**Variable**	**With Variable**	**N**	**Sample Correlation**	**Fisher's z**	**Bias Adjustment**	**Correlation Estimate**	**95% Confidence Limits**		**p Value for H0:Rho=0**
**top2lg2mean**	**MAF**	12	-0.32116	-0.33293	-0.01460	-0.30800	-0.749432	0.322992	0.3179

Correlation C_pooltop2lg2mean_tp2 symbol MAF

The CORR Procedure

Gene_Symbol=ENPP2

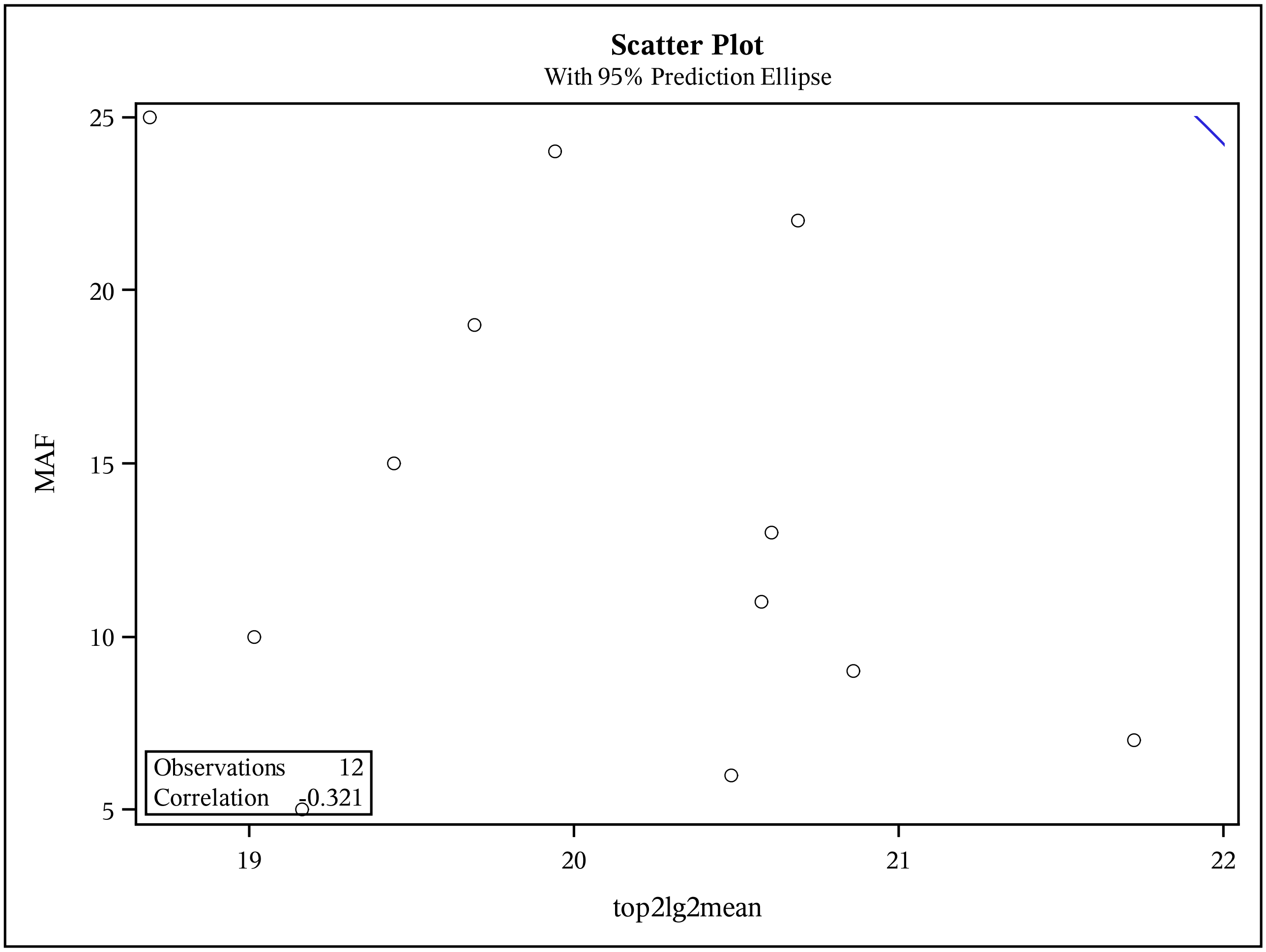

Correlation C_pooltop2lg2mean_tp2 symbol MAF

The CORR Procedure

Gene_Symbol=F2

**2 Variables:**	top2lg2mean MAF

Simple Statistics						
**Variable**	**N**	**Mean**	**Std Dev**	**Sum**	**Minimum**	**Maximum**
**top2lg2mean**	12	19.93872	0.47286	239.26468	19.14113	20.64381
**MAF**	12	13.83333	7.10740	166.00000	5.00000	25.00000

Pearson Correlation Coefficients, N = 12<br>Prob > |r| under H0: Rho=0		
	**top2lg2mean**	**MAF**
**top2lg2mean**	1.00000	-0.20913<br>0.5142
**MAF**	-0.20913<br>0.5142	1.00000

Pearson Correlation Statistics (Fisher's z Transformation)									
**Variable**	**With Variable**	**N**	**Sample Correlation**	**Fisher's z**	**Bias Adjustment**	**Correlation Estimate**	**95% Confidence Limits**		**p Value for H0:Rho=0**
**top2lg2mean**	**MAF**	12	-0.20913	-0.21226	-0.00951	-0.20003	-0.694232	0.422361	0.5243

Correlation C_pooltop2lg2mean_tp2 symbol MAF

The CORR Procedure

Gene_Symbol=F2

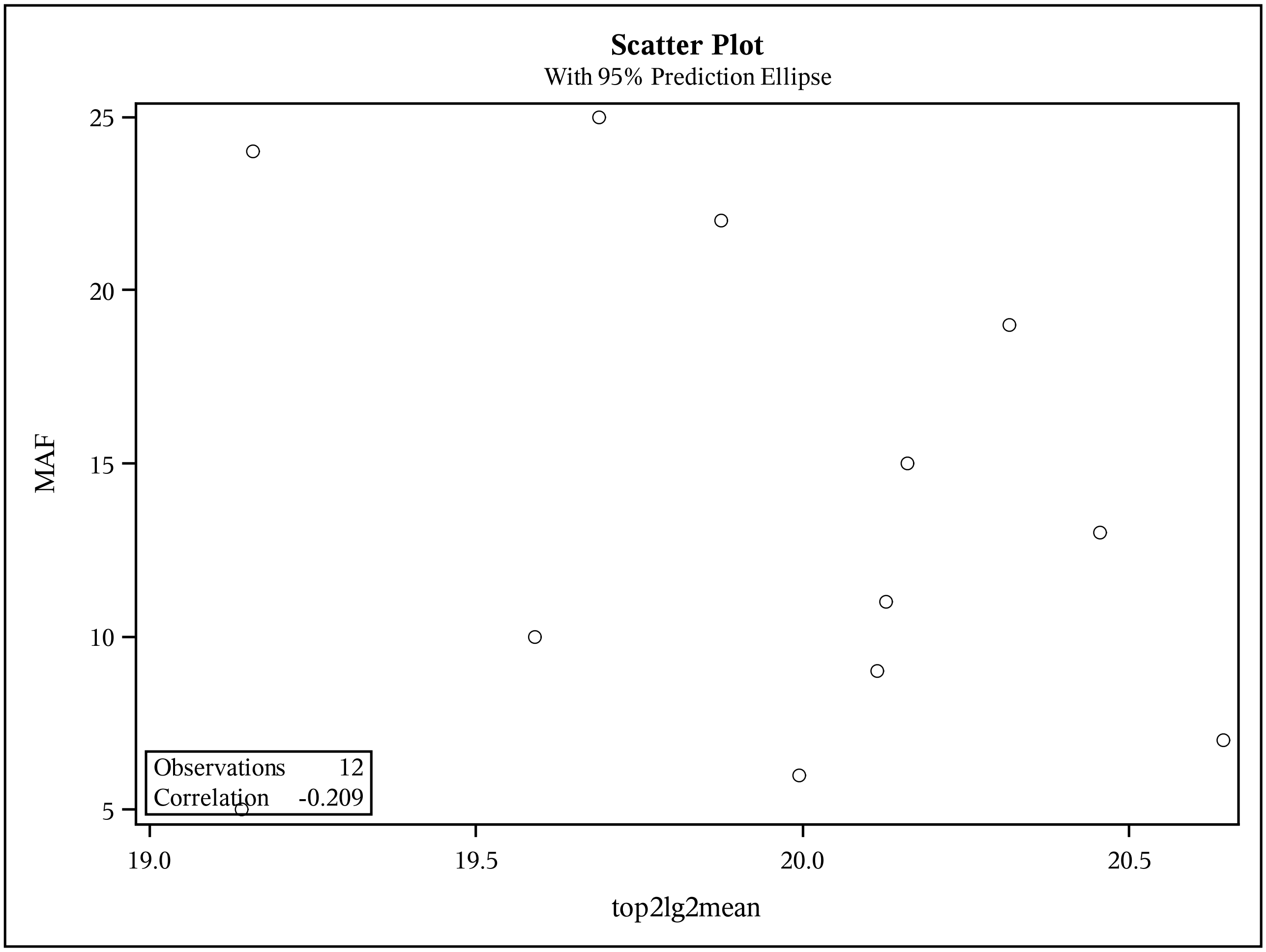

Correlation C_pooltop2lg2mean_tp2 symbol MAF

The CORR Procedure

Gene_Symbol=FBLN1

**2 Variables:**	top2lg2mean MAF

Simple Statistics						
**Variable**	**N**	**Mean**	**Std Dev**	**Sum**	**Minimum**	**Maximum**
**top2lg2mean**	12	21.28993	0.21031	255.47919	20.90053	21.60484
**MAF**	12	13.83333	7.10740	166.00000	5.00000	25.00000

Pearson Correlation Coefficients, N = 12<br>Prob > |r| under H0: Rho=0		
	**top2lg2mean**	**MAF**
**top2lg2mean**	1.00000	0.14107<br>0.6619
**MAF**	0.14107<br>0.6619	1.00000

Pearson Correlation Statistics (Fisher's z Transformation)									
**Variable**	**With Variable**	**N**	**Sample Correlation**	**Fisher's z**	**Bias Adjustment**	**Correlation Estimate**	**95% Confidence Limits**		**p Value for H0:Rho=0**
**top2lg2mean**	**MAF**	12	0.14107	0.14202	0.00641	0.13478	-0.475937	0.657799	0.6701

Correlation C_pooltop2lg2mean_tp2 symbol MAF

The CORR Procedure

Gene_Symbol=FBLN1

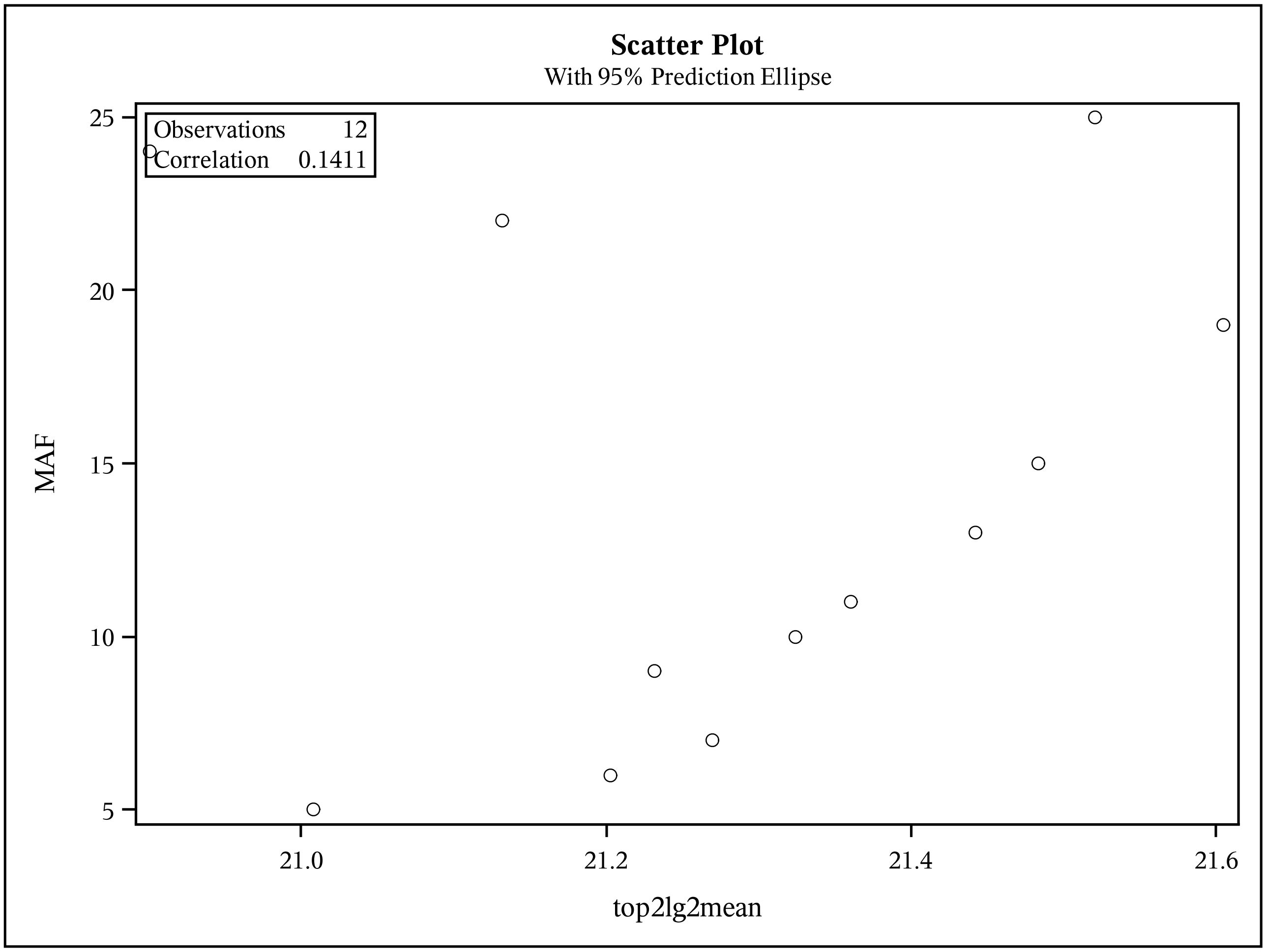

Correlation C_pooltop2lg2mean_tp2 symbol MAF

The CORR Procedure

Gene_Symbol=FGG

**2 Variables:**	top2lg2mean MAF

Simple Statistics						
**Variable**	**N**	**Mean**	**Std Dev**	**Sum**	**Minimum**	**Maximum**
**top2lg2mean**	12	17.97301	0.39551	215.67613	17.49951	19.09702
**MAF**	12	13.83333	7.10740	166.00000	5.00000	25.00000

Pearson Correlation Coefficients, N = 12<br>Prob > |r| under H0: Rho=0		
	**top2lg2mean**	**MAF**
**top2lg2mean**	1.00000	-0.24364<br>0.4454
**MAF**	-0.24364<br>0.4454	1.00000

Pearson Correlation Statistics (Fisher's z Transformation)									
**Variable**	**With Variable**	**N**	**Sample Correlation**	**Fisher's z**	**Bias Adjustment**	**Correlation Estimate**	**95% Confidence Limits**		**p Value for H0:Rho=0**
**top2lg2mean**	**MAF**	12	-0.24364	-0.24864	-0.01107	-0.23319	-0.711830	0.393352	0.4557

Correlation C_pooltop2lg2mean_tp2 symbol MAF

The CORR Procedure

Gene_Symbol=FGG

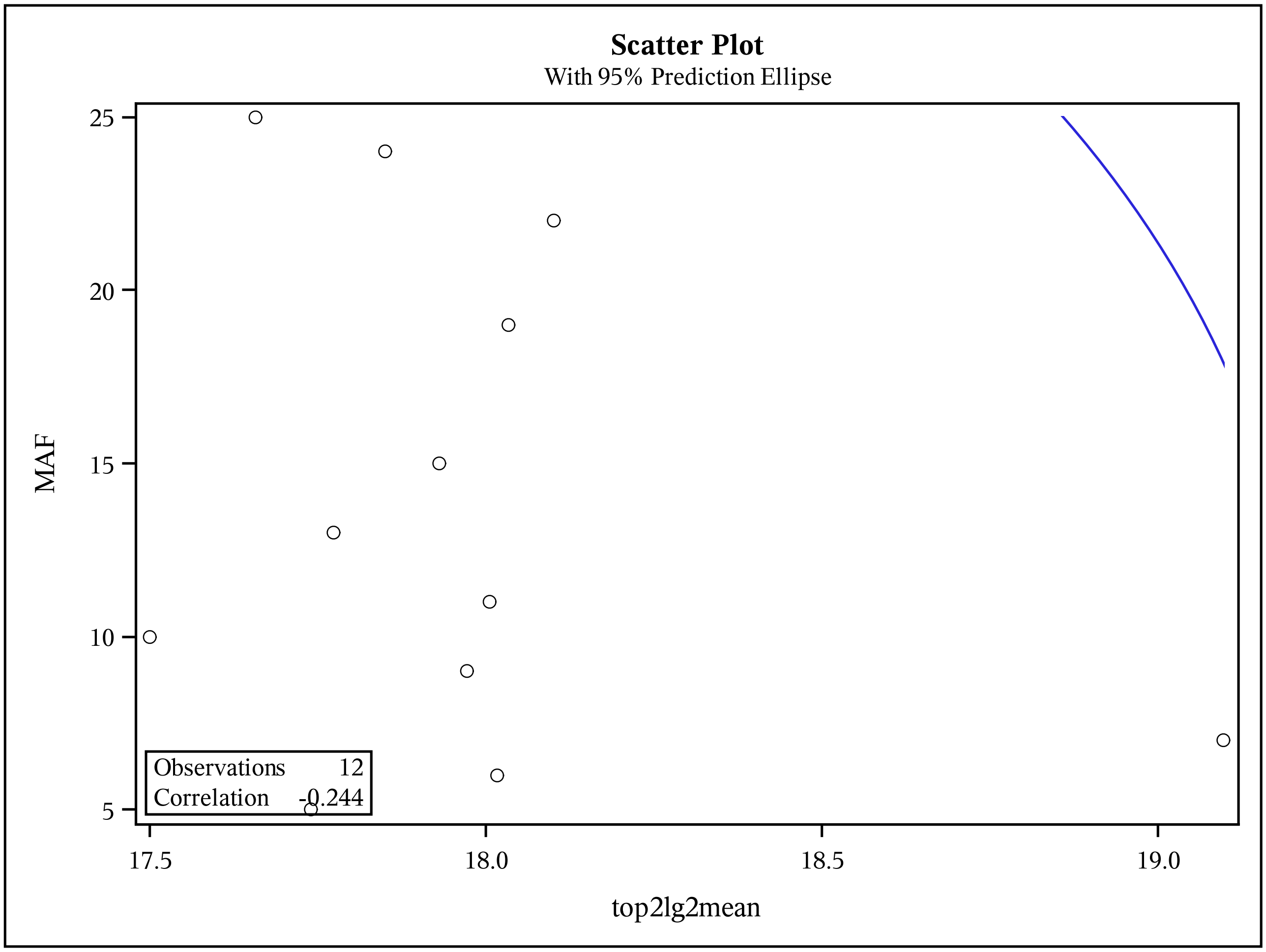

Correlation C_pooltop2lg2mean_tp2 symbol MAF

The CORR Procedure

Gene_Symbol=FN1

**2 Variables:**	top2lg2mean MAF

Simple Statistics						
**Variable**	**N**	**Mean**	**Std Dev**	**Sum**	**Minimum**	**Maximum**
**top2lg2mean**	12	20.22242	0.38957	242.66899	19.48613	20.90945
**MAF**	12	13.83333	7.10740	166.00000	5.00000	25.00000

Pearson Correlation Coefficients, N = 12<br>Prob > |r| under H0: Rho=0		
	**top2lg2mean**	**MAF**
**top2lg2mean**	1.00000	0.67285<br>0.0165
**MAF**	0.67285<br>0.0165	1.00000

Pearson Correlation Statistics (Fisher's z Transformation)									
**Variable**	**With Variable**	**N**	**Sample Correlation**	**Fisher's z**	**Bias Adjustment**	**Correlation Estimate**	**95% Confidence Limits**		**p Value for H0:Rho=0**
**top2lg2mean**	**MAF**	12	0.67285	0.81594	0.03058	0.65577	0.131272	0.893431	0.0144

Correlation C_pooltop2lg2mean_tp2 symbol MAF

The CORR Procedure

Gene_Symbol=FN1

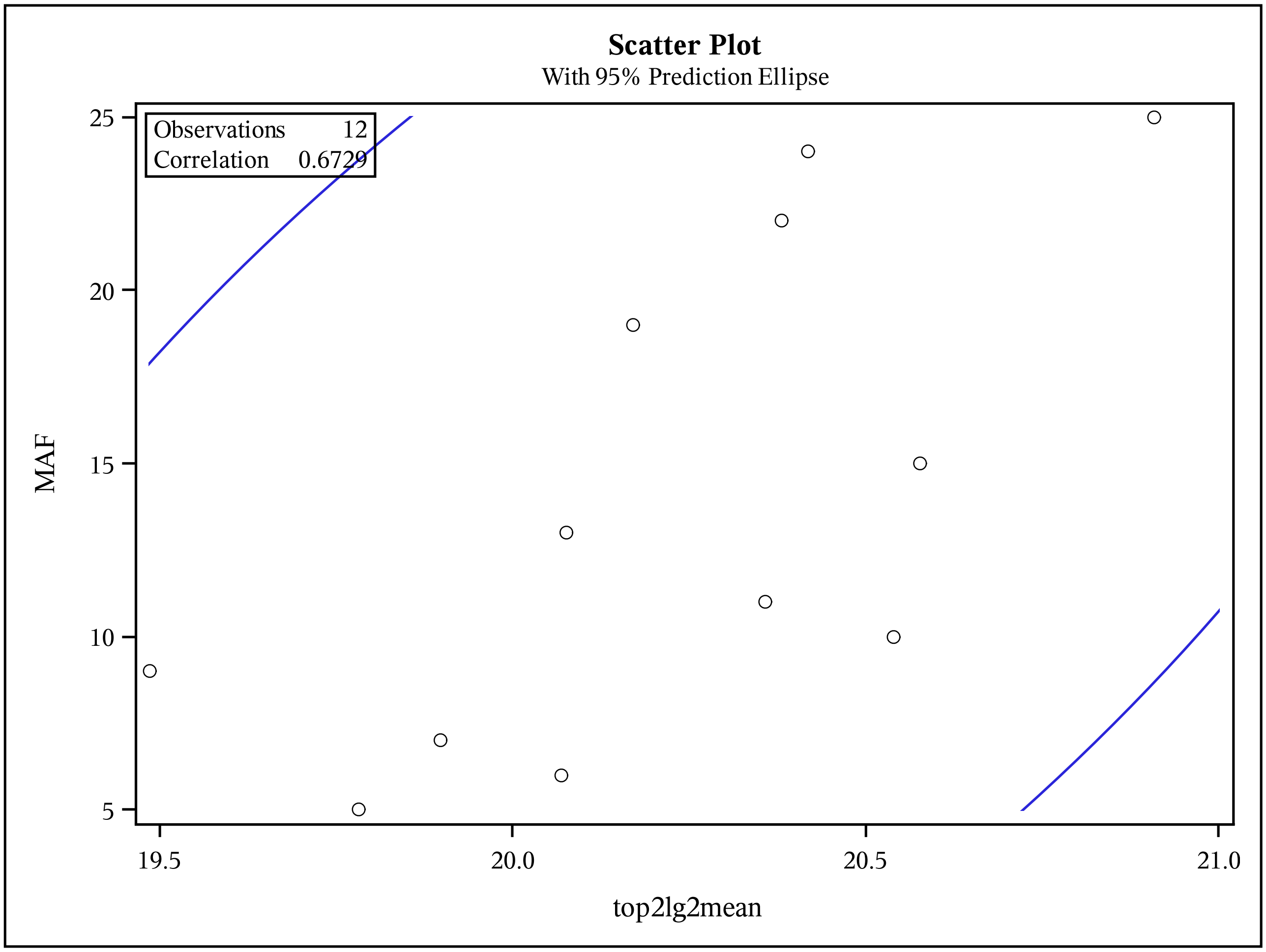

Correlation C_pooltop2lg2mean_tp2 symbol MAF

The CORR Procedure

Gene_Symbol=GC

2 Variables:	top2lg2mean MAF

Simple Statistics						
Variable	N	Mean	Std Dev	Sum	Minimum	Maximum
top2lg2mean	12	23.33209	0.20100	279.98510	22.82401	23.60913
MAF	12	13.83333	7.10740	166.00000	5.00000	25.00000

Pearson Correlation Coefficients, N = 12<br>Prob > |r| under H0: Rho=0		
	top2lg2mean	MAF
top2lg2mean	1.00000	0.49518<br>0.1017
MAF	0.49518<br>0.1017	1.00000

Pearson Correlation Statistics (Fisher's z Transformation)									
Variable	With Variable	N	Sample Correlation	Fisher's z	Bias Adjustment	Correlation Estimate	95% Confidence Limits		p Value for H0:Rho=0
top2lg2mean	MAF	12	0.49518	0.54290	0.02251	0.47800	-0.132150	0.825460	0.1034

Correlation C_pooltop2lg2mean_tp2 symbol MAF

The CORR Procedure

Gene_Symbol=GC

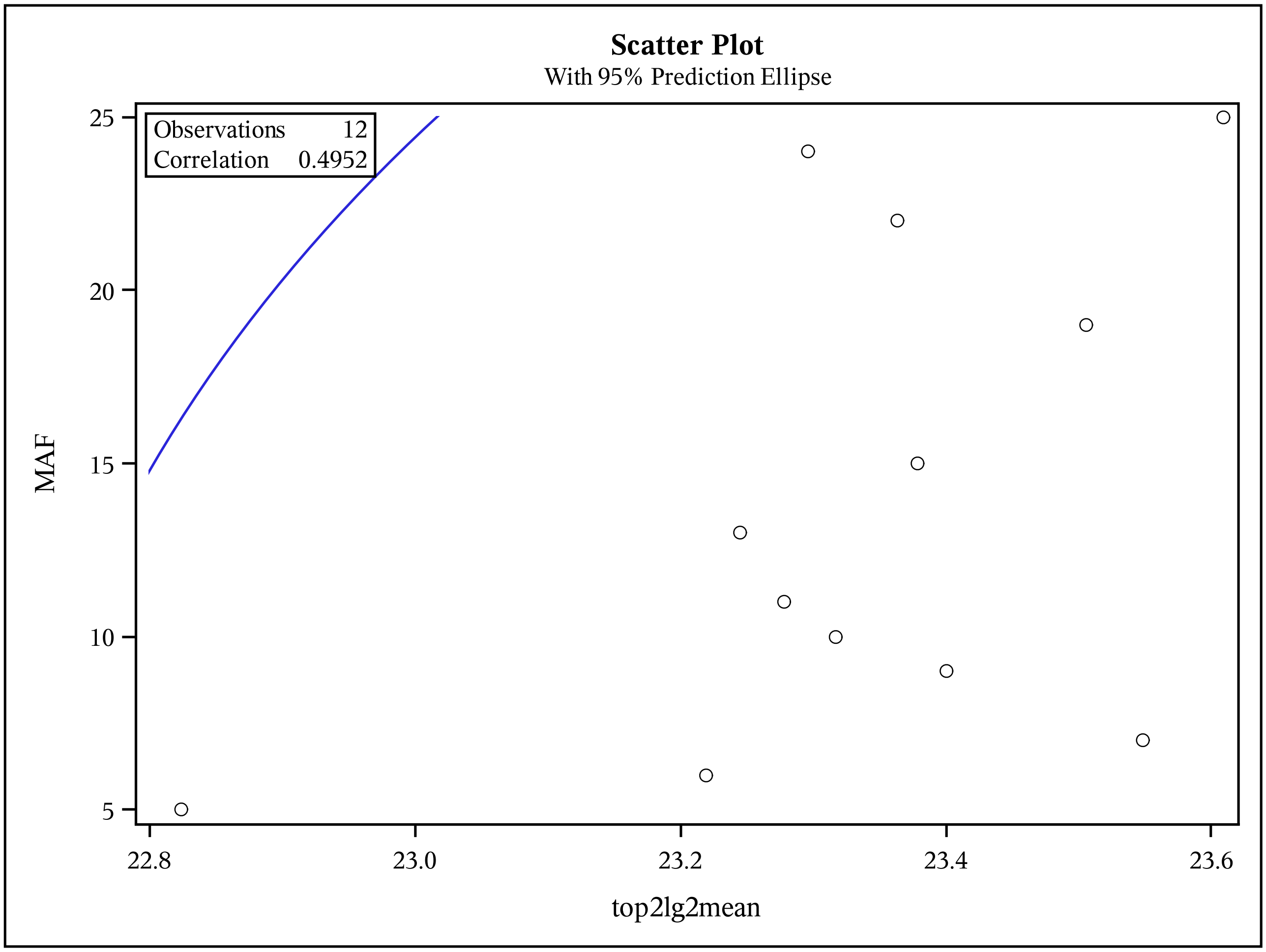

Correlation C_pooltop2lg2mean_tp2 symbol MAF

The CORR Procedure

Gene_Symbol=GSN

**2 Variables:**	top2lg2mean MAF

Simple Statistics						
**Variable**	**N**	**Mean**	**Std Dev**	**Sum**	**Minimum**	**Maximum**
**top2lg2mean**	12	21.67081	0.34197	260.04974	20.77562	21.98326
**MAF**	12	13.83333	7.10740	166.00000	5.00000	25.00000

Pearson Correlation Coefficients, N = 12<br>Prob > |r| under H0: Rho=0		
	**top2lg2mean**	**MAF**
**top2lg2mean**	1.00000	-0.50583<br>0.0934
**MAF**	-0.50583<br>0.0934	1.00000

Pearson Correlation Statistics (Fisher's z Transformation)									
**Variable**	**With Variable**	**N**	**Sample Correlation**	**Fisher's z**	**Bias Adjustment**	**Correlation Estimate**	**95% Confidence Limits**		**p Value for H0:Rho=0**
**top2lg2mean**	**MAF**	12	-0.50583	-0.55711	-0.02299	-0.48853	-0.829784	0.118638	0.0947

Correlation C_pooltop2lg2mean_tp2 symbol MAF

The CORR Procedure

Gene_Symbol=GSN

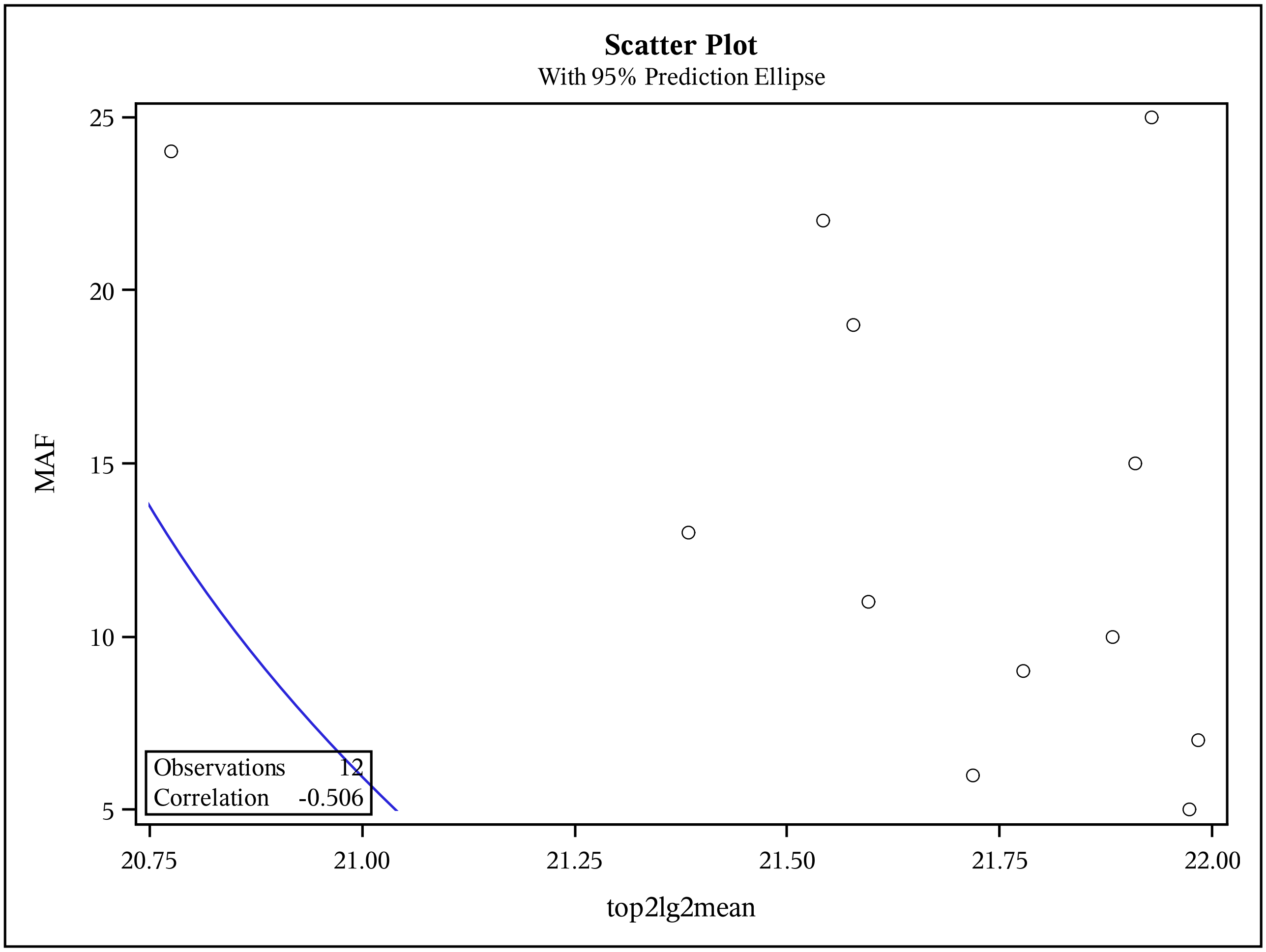

Correlation C_pooltop2lg2mean_tp2 symbol MAF

The CORR Procedure

Gene_Symbol=HPX

**2 Variables:**	top2lg2mean MAF

Simple Statistics						
**Variable**	**N**	**Mean**	**Std Dev**	**Sum**	**Minimum**	**Maximum**
**top2lg2mean**	12	25.81965	0.13873	309.83584	25.65299	26.07131
**MAF**	12	13.83333	7.10740	166.00000	5.00000	25.00000

Pearson Correlation Coefficients, N = 12<br>Prob > |r| under H0: Rho=0		
	**top2lg2mean**	**MAF**
**top2lg2mean**	1.00000	-0.01345<br>0.9669
**MAF**	-0.01345<br>0.9669	1.00000

Pearson Correlation Statistics (Fisher's z Transformation)									
**Variable**	**With Variable**	**N**	**Sample Correlation**	**Fisher's z**	**Bias Adjustment**	**Correlation Estimate**	**95% Confidence Limits**		**p Value for H0:Rho=0**
**top2lg2mean**	**MAF**	12	-0.01345	-0.01345	-0.0006112	-0.01284	-0.582447	0.565230	0.9678

Correlation C_pooltop2lg2mean_tp2 symbol MAF

The CORR Procedure

Gene_Symbol=HPX

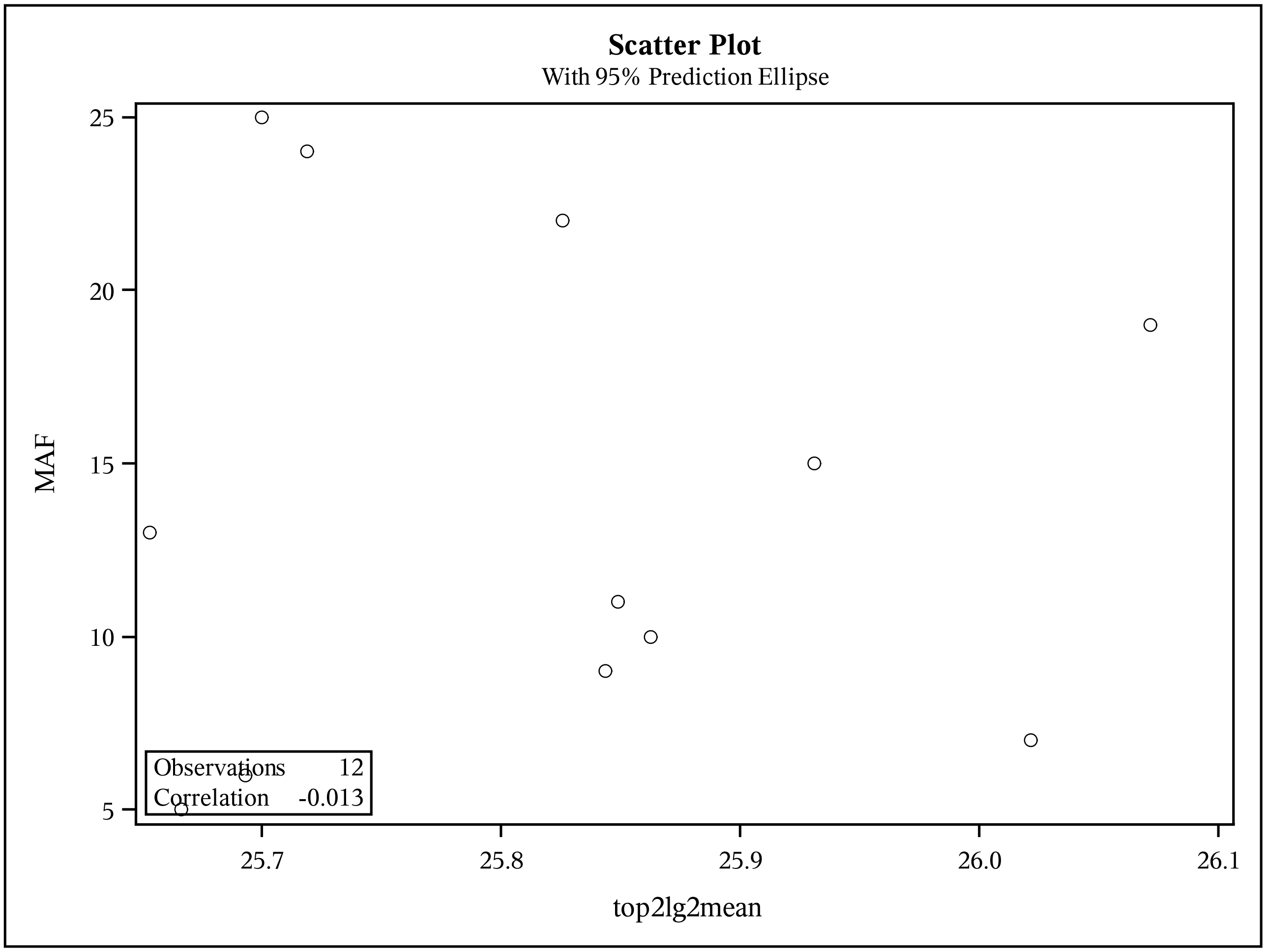

Correlation C_pooltop2lg2mean_tp2 symbol MAF

The CORR Procedure

Gene_Symbol=IGFBP6

**2 Variables:**	top2lg2mean MAF

Simple Statistics						
**Variable**	**N**	**Mean**	**Std Dev**	**Sum**	**Minimum**	**Maximum**
**top2lg2mean**	12	21.76537	0.24356	261.18448	21.06331	22.01586
**MAF**	12	13.83333	7.10740	166.00000	5.00000	25.00000

Pearson Correlation Coefficients, N = 12<br>Prob > |r| under H0: Rho=0		
	**top2lg2mean**	**MAF**
**top2lg2mean**	1.00000	0.39640<br>0.2021
**MAF**	0.39640<br>0.2021	1.00000

Pearson Correlation Statistics (Fisher's z Transformation)									
**Variable**	**With Variable**	**N**	**Sample Correlation**	**Fisher's z**	**Bias Adjustment**	**Correlation Estimate**	**95% Confidence Limits**		**p Value for H0:Rho=0**
**top2lg2mean**	**MAF**	12	0.39640	0.41937	0.01802	0.38110	-0.246773	0.783615	0.2084

Correlation C_pooltop2lg2mean_tp2 symbol MAF

The CORR Procedure

Gene_Symbol=IGFBP6

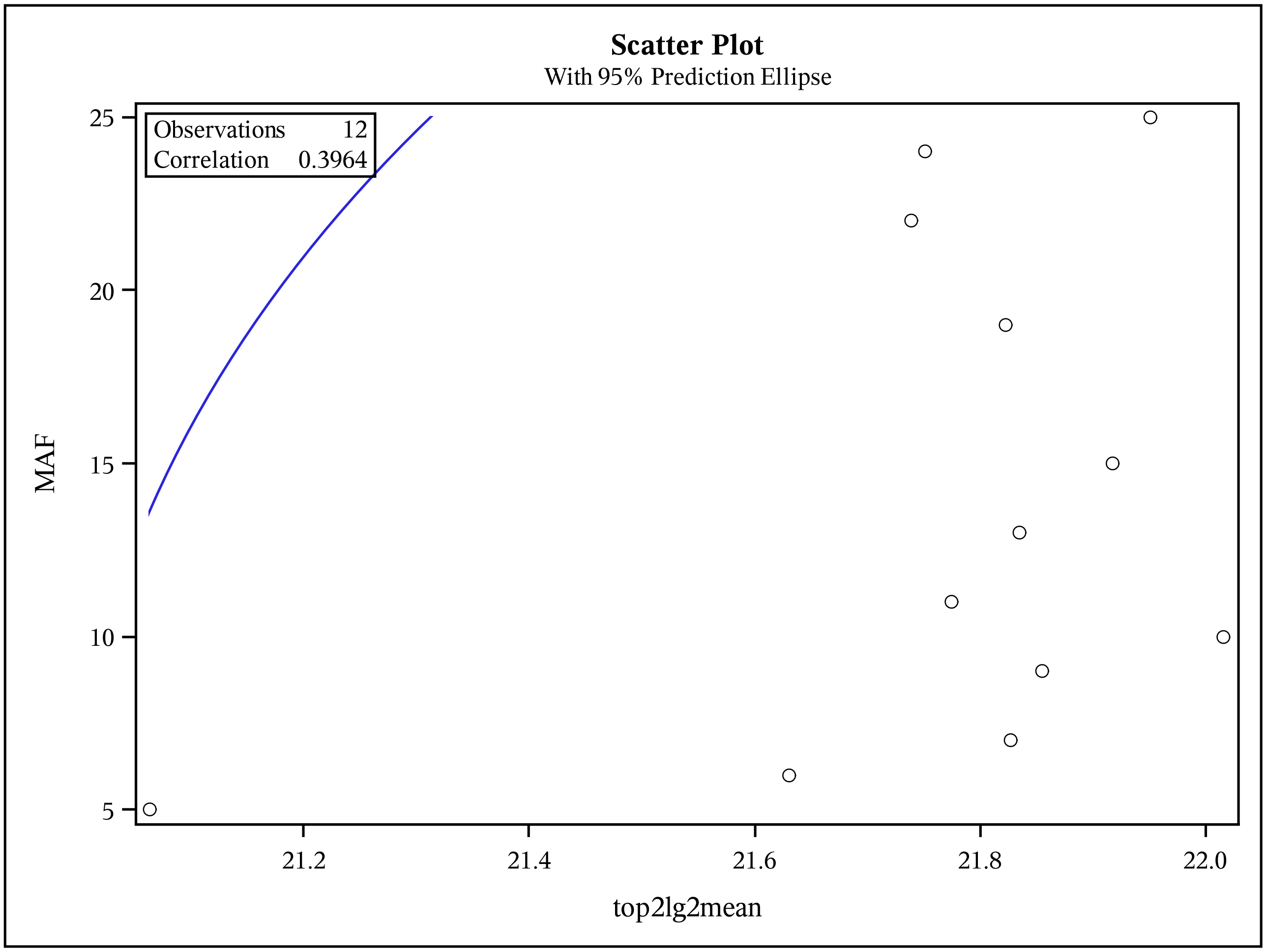

Correlation C_pooltop2lg2mean_tp2 symbol MAF

The CORR Procedure

Gene_Symbol=KLK6

**2 Variables:**	top2lg2mean MAF

Simple Statistics						
**Variable**	**N**	**Mean**	**Std Dev**	**Sum**	**Minimum**	**Maximum**
**top2lg2mean**	12	23.33664	0.20344	280.03972	23.06448	23.67569
**MAF**	12	13.83333	7.10740	166.00000	5.00000	25.00000

Pearson Correlation Coefficients, N = 12<br>Prob > |r| under H0: Rho=0		
	**top2lg2mean**	**MAF**
**top2lg2mean**	1.00000	0.27600<br>0.3852
**MAF**	0.27600<br>0.3852	1.00000

Pearson Correlation Statistics (Fisher's z Transformation)									
**Variable**	**With Variable**	**N**	**Sample Correlation**	**Fisher's z**	**Bias Adjustment**	**Correlation Estimate**	**95% Confidence Limits**		**p Value for H0:Rho=0**
**top2lg2mean**	**MAF**	12	0.27600	0.28334	0.01255	0.26437	-0.364896	0.727841	0.3953

Correlation C_pooltop2lg2mean_tp2 symbol MAF

The CORR Procedure

Gene_Symbol=KLK6

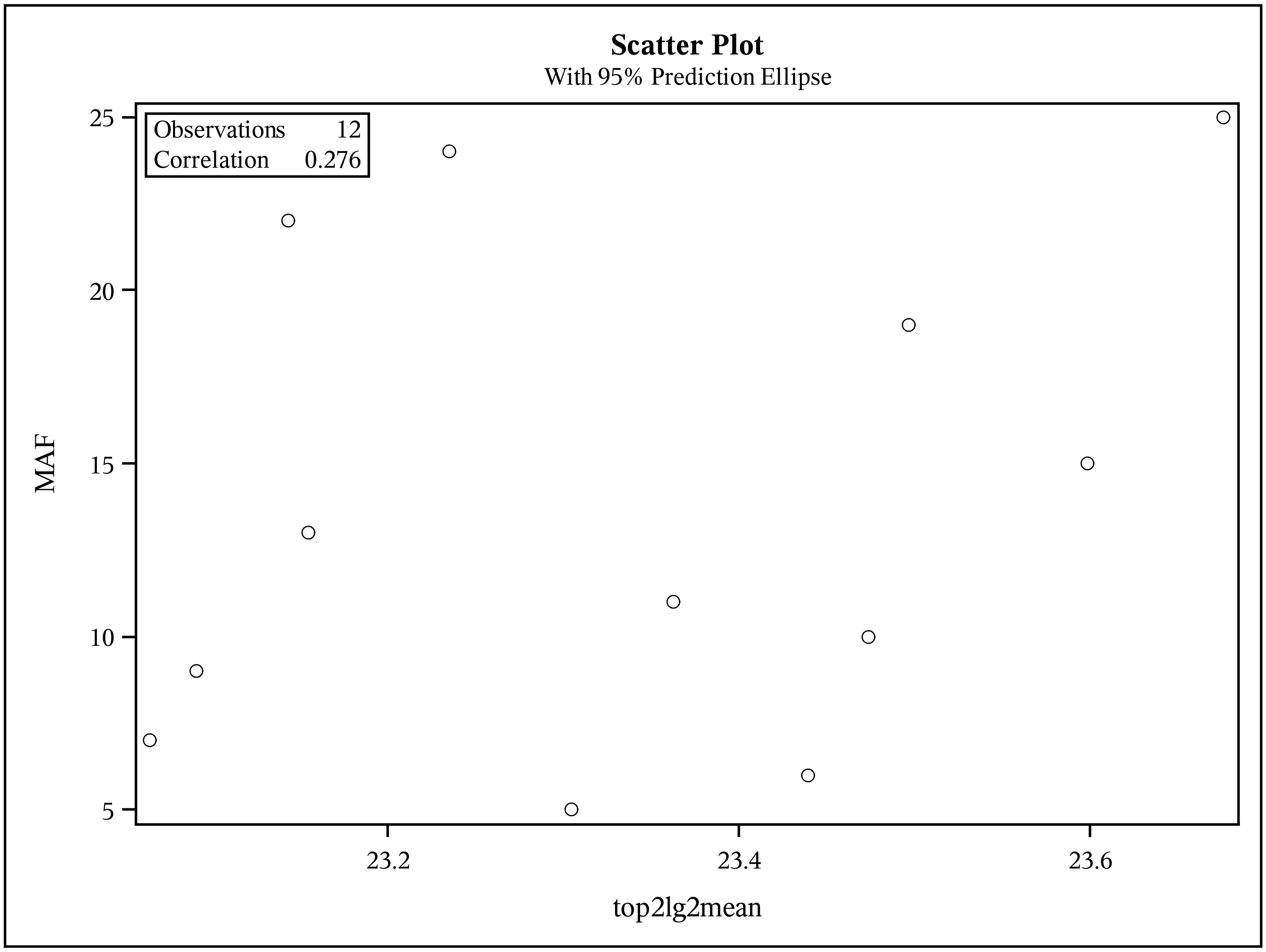

Correlation C_pooltop2lg2mean_tp2 symbol MAF

The CORR Procedure

Gene_Symbol=KNG1

**2 Variables:**	top2lg2mean MAF

Simple Statistics						
**Variable**	**N**	**Mean**	**Std Dev**	**Sum**	**Minimum**	**Maximum**
**top2lg2mean**	12	19.99606	0.33916	239.95268	19.18894	20.32536
**MAF**	12	13.83333	7.10740	166.00000	5.00000	25.00000

Pearson Correlation Coefficients, N = 12<br>Prob > |r| under H0: Rho=0		
	**top2lg2mean**	**MAF**
**top2lg2mean**	1.00000	0.39838<br>0.1996
**MAF**	0.39838<br>0.1996	1.00000

Pearson Correlation Statistics (Fisher's z Transformation)									
**Variable**	**With Variable**	**N**	**Sample Correlation**	**Fisher's z**	**Bias Adjustment**	**Correlation Estimate**	**95% Confidence Limits**		**p Value for H0:Rho=0**
**top2lg2mean**	**MAF**	12	0.39838	0.42172	0.01811	0.38303	-0.244647	0.784487	0.2058

Correlation C_pooltop2lg2mean_tp2 symbol MAF

The CORR Procedure

Gene_Symbol=KNG1

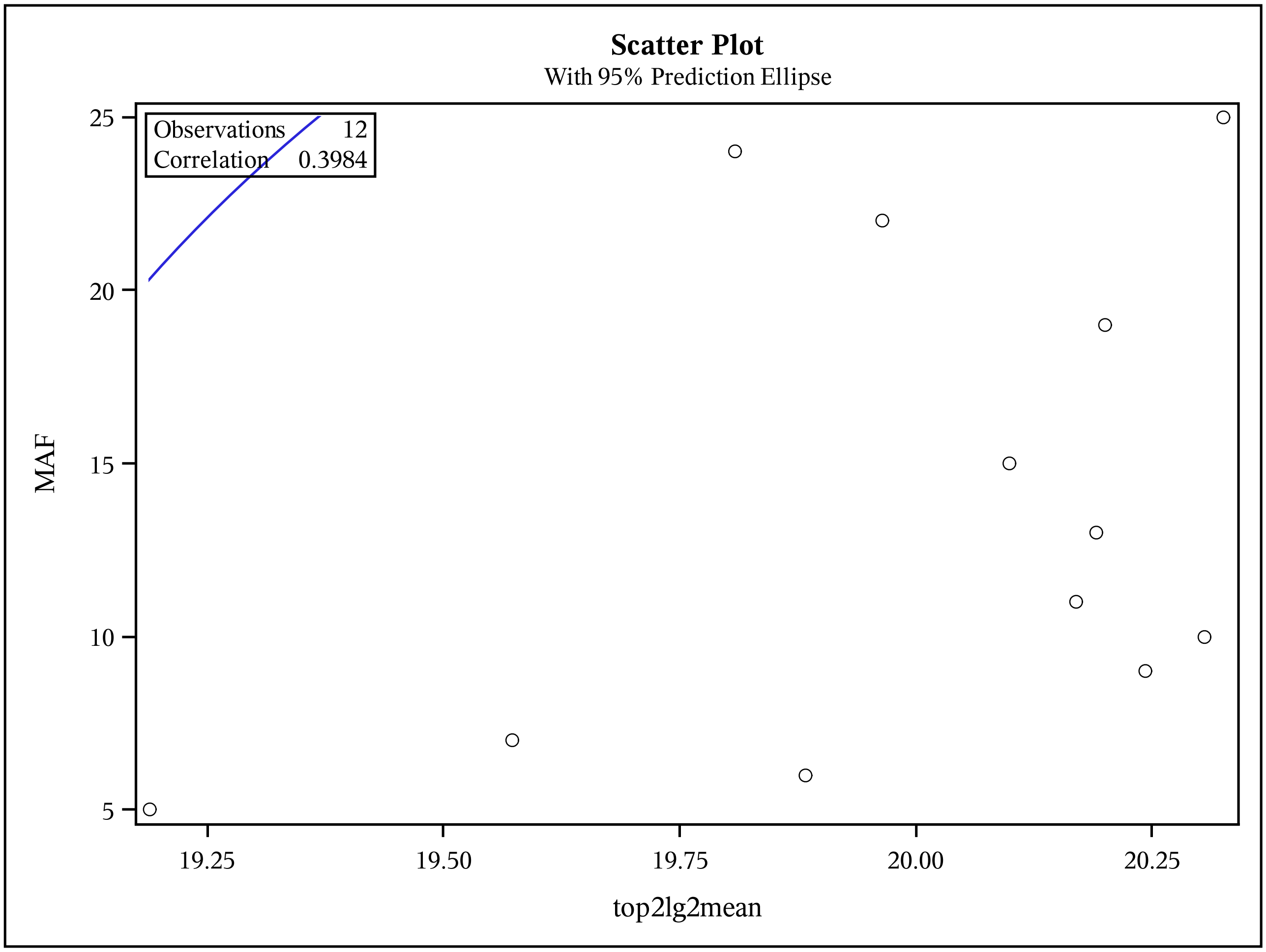

Correlation C_pooltop2lg2mean_tp2 symbol MAF

The CORR Procedure

Gene_Symbol=LGALS3BP

2 Variables:	top2lg2mean MAF

Simple Statistics						
**Variable**	**N**	**Mean**	**Std Dev**	**Sum**	**Minimum**	**Maximum**
**top2lg2mean**	12	19.05697	0.50398	228.68362	18.42866	19.85145
**MAF**	12	13.83333	7.10740	166.00000	5.00000	25.00000

Pearson Correlation Coefficients, N = 12<br>Prob > |r| under H0: Rho=0		
	**top2lg2mean**	**MAF**
**top2lg2mean**	1.00000	0.06263<br>0.8467
**MAF**	0.06263<br>0.8467	1.00000

Pearson Correlation Statistics (Fisher's z Transformation)									
**Variable**	**With Variable**	**N**	**Sample Correlation**	**Fisher's z**	**Bias Adjustment**	**Correlation Estimate**	**95% Confidence Limits**		**p Value for H0:Rho=0**
**top2lg2mean**	**MAF**	12	0.06263	0.06272	0.00285	0.05980	-0.532374	0.612674	0.8508

Correlation C_pooltop2lg2mean_tp2 symbol MAF

The CORR Procedure

Gene_Symbol=LGALS3BP

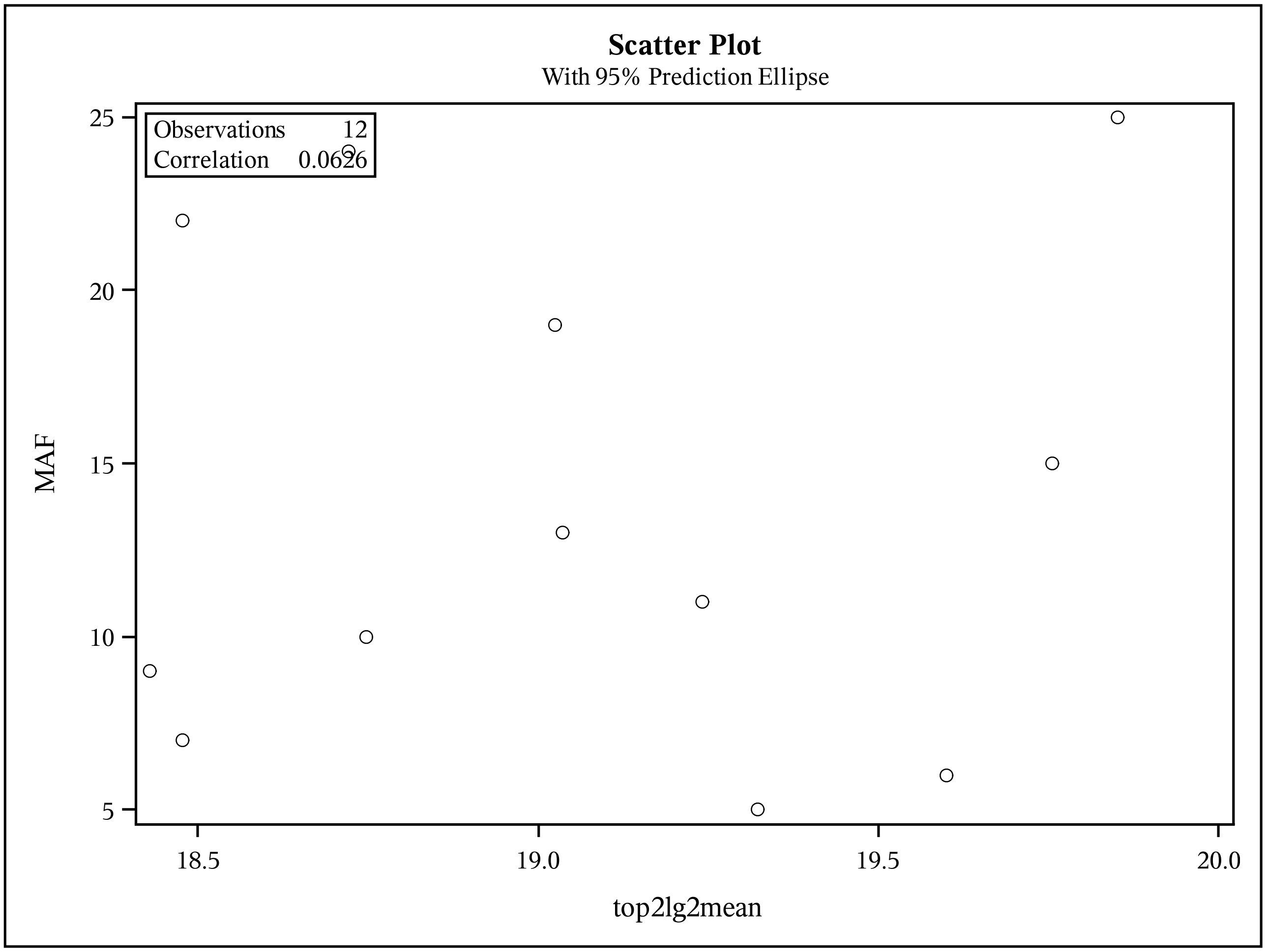

Correlation C_pooltop2lg2mean_tp2 symbol MAF

The CORR Procedure

Gene_Symbol=LRG1

**2 Variables:**	top2lg2mean MAF

Simple Statistics						
**Variable**	**N**	**Mean**	**Std Dev**	**Sum**	**Minimum**	**Maximum**
**top2lg2mean**	12	21.04480	0.32579	252.53765	20.54096	21.57320
**MAF**	12	13.83333	7.10740	166.00000	5.00000	25.00000

Pearson Correlation Coefficients, N = 12<br>Prob > |r| under H0: Rho=0		
	**top2lg2mean**	**MAF**
**top2lg2mean**	1.00000	0.17021<br>0.5969
**MAF**	0.17021<br>0.5969	1.00000

Pearson Correlation Statistics (Fisher's z Transformation)									
**Variable**	**With Variable**	**N**	**Sample Correlation**	**Fisher's z**	**Bias Adjustment**	**Correlation Estimate**	**95% Confidence Limits**		**p Value for H0:Rho=0**
**top2lg2mean**	**MAF**	12	0.17021	0.17188	0.00774	0.16268	-0.453564	0.673687	0.6061

Correlation C_pooltop2lg2mean_tp2 symbol MAF

The CORR Procedure

Gene_Symbol=LRG1

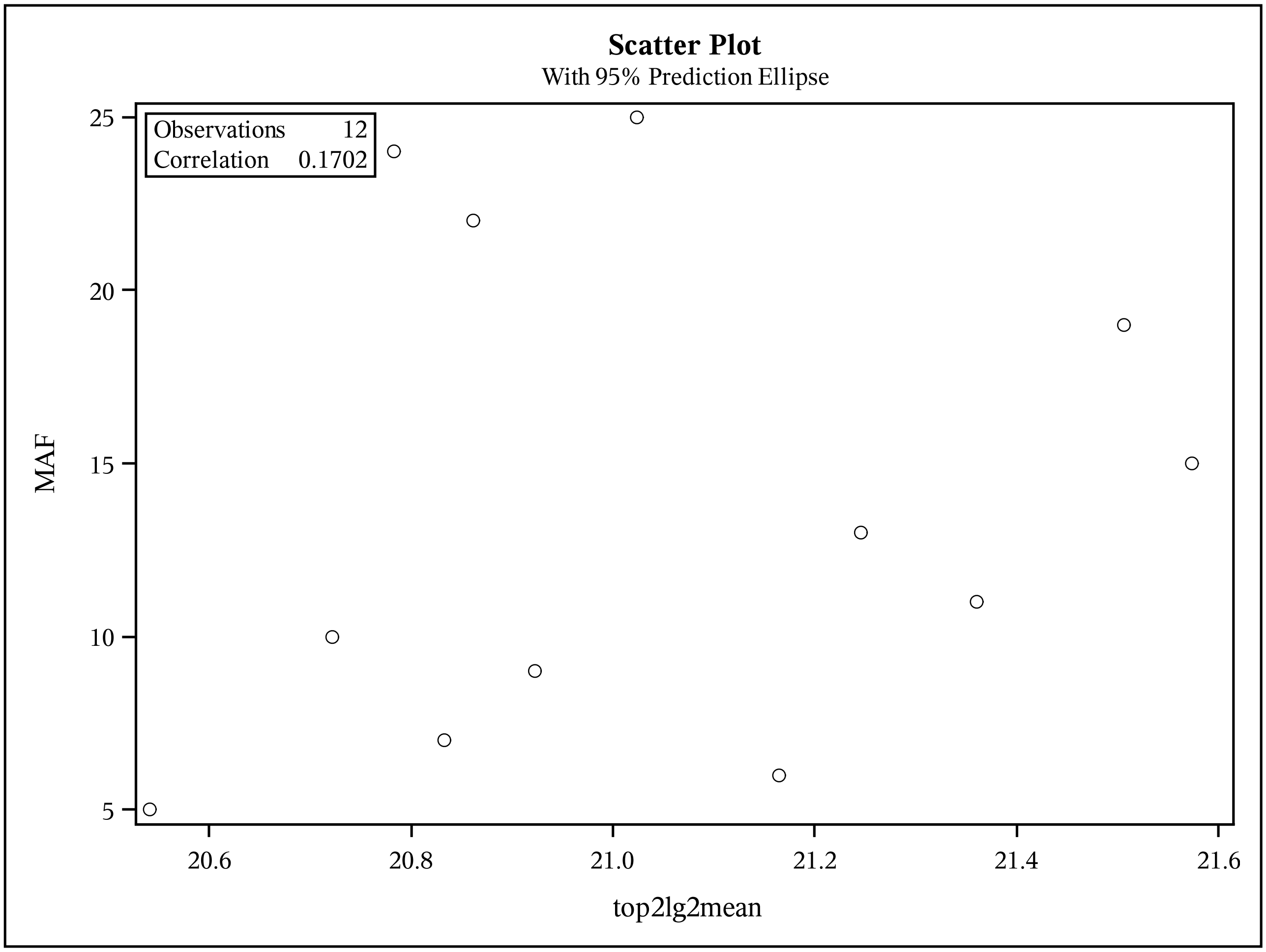

Correlation C_pooltop2lg2mean_tp2 symbol MAF

The CORR Procedure

Gene_Symbol=NCAM1

**2 Variables:**	top2lg2mean MAF

Simple Statistics						
**Variable**	**N**	**Mean**	**Std Dev**	**Sum**	**Minimum**	**Maximum**
**top2lg2mean**	12	20.08905	0.30789	241.06857	19.47076	20.43145
**MAF**	12	13.83333	7.10740	166.00000	5.00000	25.00000

Pearson Correlation Coefficients, N = 12<br>Prob > |r| under H0: Rho=0		
	**top2lg2mean**	**MAF**
**top2lg2mean**	1.00000	-0.09329<br>0.7731
**MAF**	-0.09329<br>0.7731	1.00000

Pearson Correlation Statistics (Fisher's z Transformation)									
**Variable**	**With Variable**	**N**	**Sample Correlation**	**Fisher's z**	**Bias Adjustment**	**Correlation Estimate**	**95% Confidence Limits**		**p Value for H0:Rho=0**
**top2lg2mean**	**MAF**	12	-0.09329	-0.09356	-0.00424	-0.08909	-0.630740	0.510939	0.7789

Correlation C_pooltop2lg2mean_tp2 symbol MAF

The CORR Procedure

Gene_Symbol=NCAM1

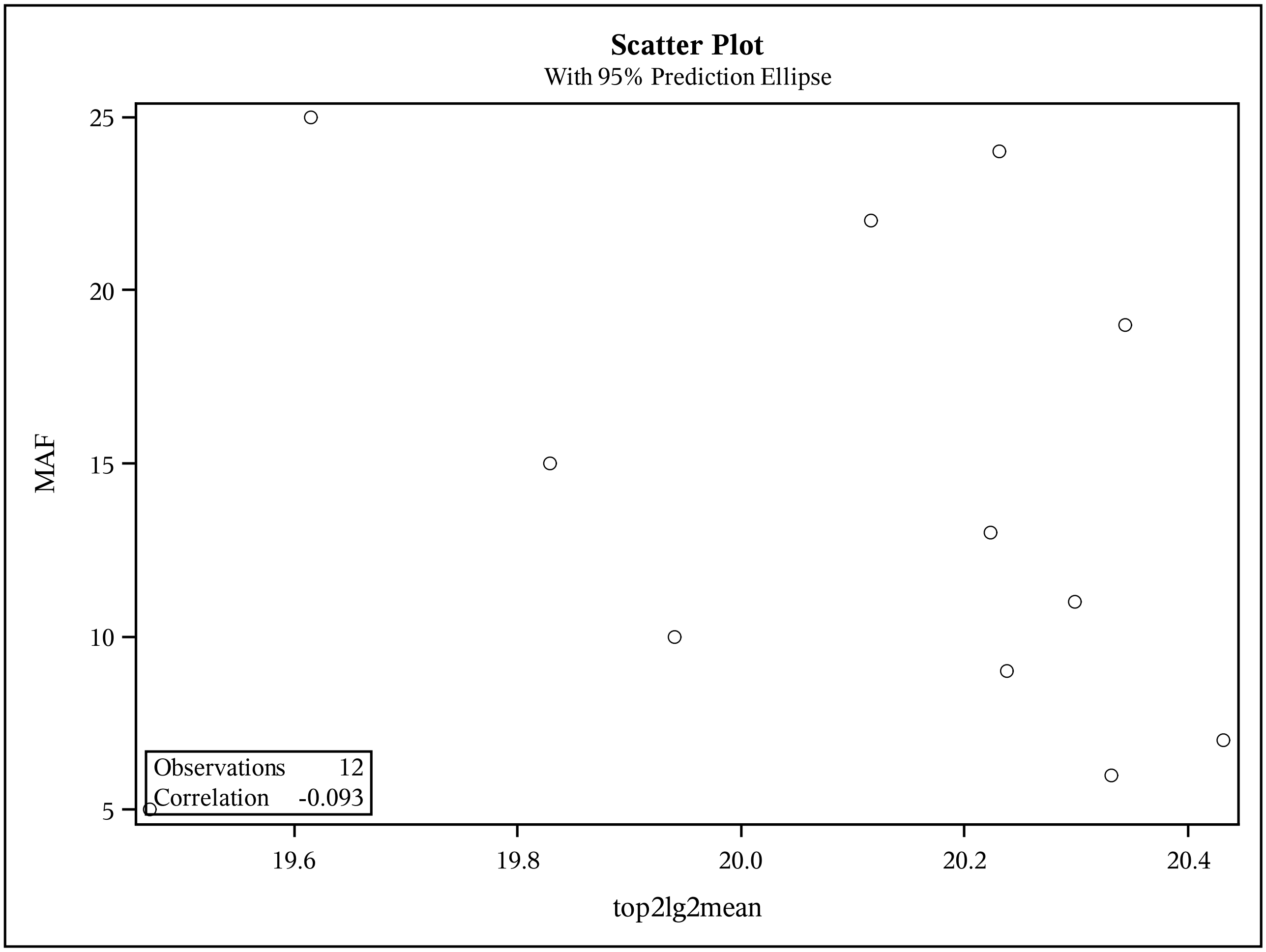

Correlation C_pooltop2lg2mean_tp2 symbol MAF

The CORR Procedure

Gene_Symbol=NCAN

**2 Variables:**	top2lg2mean MAF

Simple Statistics						
**Variable**	**N**	**Mean**	**Std Dev**	**Sum**	**Minimum**	**Maximum**
**top2lg2mean**	12	17.41564	0.26096	208.98771	16.95932	17.82961
**MAF**	12	13.83333	7.10740	166.00000	5.00000	25.00000

Pearson Correlation Coefficients, N = 12<br>Prob > |r| under H0: Rho=0		
	**top2lg2mean**	**MAF**
**top2lg2mean**	1.00000	-0.28205<br>0.3744
**MAF**	-0.28205<br>0.3744	1.00000

Pearson Correlation Statistics (Fisher's z Transformation)									
**Variable**	**With Variable**	**N**	**Sample Correlation**	**Fisher's z**	**Bias Adjustment**	**Correlation Estimate**	**95% Confidence Limits**		**p Value for H0:Rho=0**
**top2lg2mean**	**MAF**	12	-0.28205	-0.28991	-0.01282	-0.27021	-0.730785	0.359432	0.3845

Correlation C_pooltop2lg2mean_tp2 symbol MAF

The CORR Procedure

Gene_Symbol=NCAN

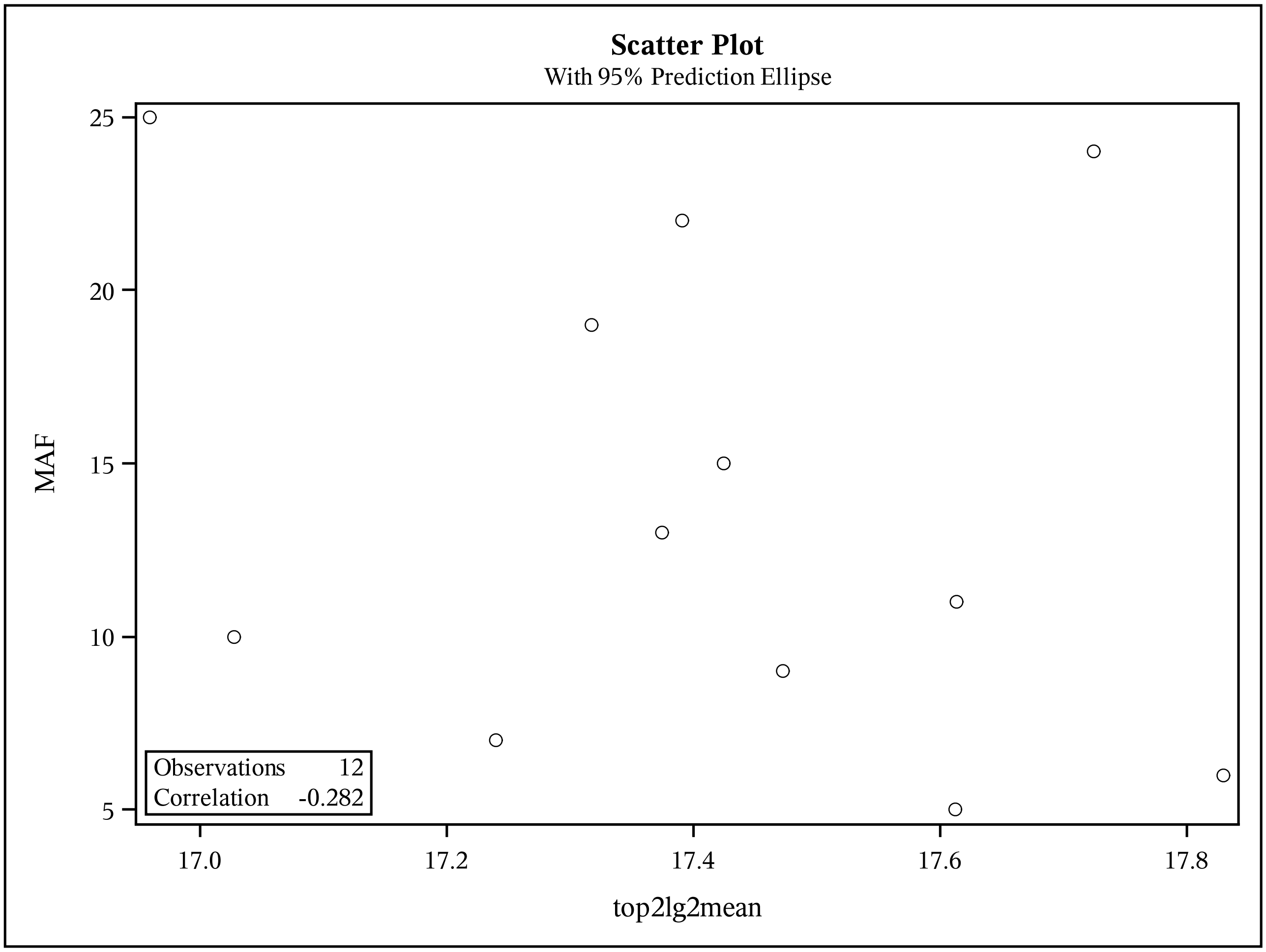

Correlation C_pooltop2lg2mean_tp2 symbol MAF

The CORR Procedure

Gene_Symbol=NELL2

**2 Variables:**	top2lg2mean MAF

Simple Statistics						
**Variable**	**N**	**Mean**	**Std Dev**	**Sum**	**Minimum**	**Maximum**
**top2lg2mean**	12	19.96714	0.43713	239.60562	19.30654	20.90915
**MAF**	12	13.83333	7.10740	166.00000	5.00000	25.00000

Pearson Correlation Coefficients, N = 12<br>Prob > |r| under H0: Rho=0		
	**top2lg2mean**	**MAF**
**top2lg2mean**	1.00000	-0.62021<br>0.0314
**MAF**	-0.62021<br>0.0314	1.00000

Pearson Correlation Statistics (Fisher's z Transformation)									
**Variable**	**With Variable**	**N**	**Sample Correlation**	**Fisher's z**	**Bias Adjustment**	**Correlation Estimate**	**95% Confidence Limits**		**p Value for H0:Rho=0**
**top2lg2mean**	**MAF**	12	-0.62021	-0.72534	-0.02819	-0.60256	-0.874165	-0.043801	0.0296

Correlation C_pooltop2lg2mean_tp2 symbol MAF

The CORR Procedure

Gene_Symbol=NELL2

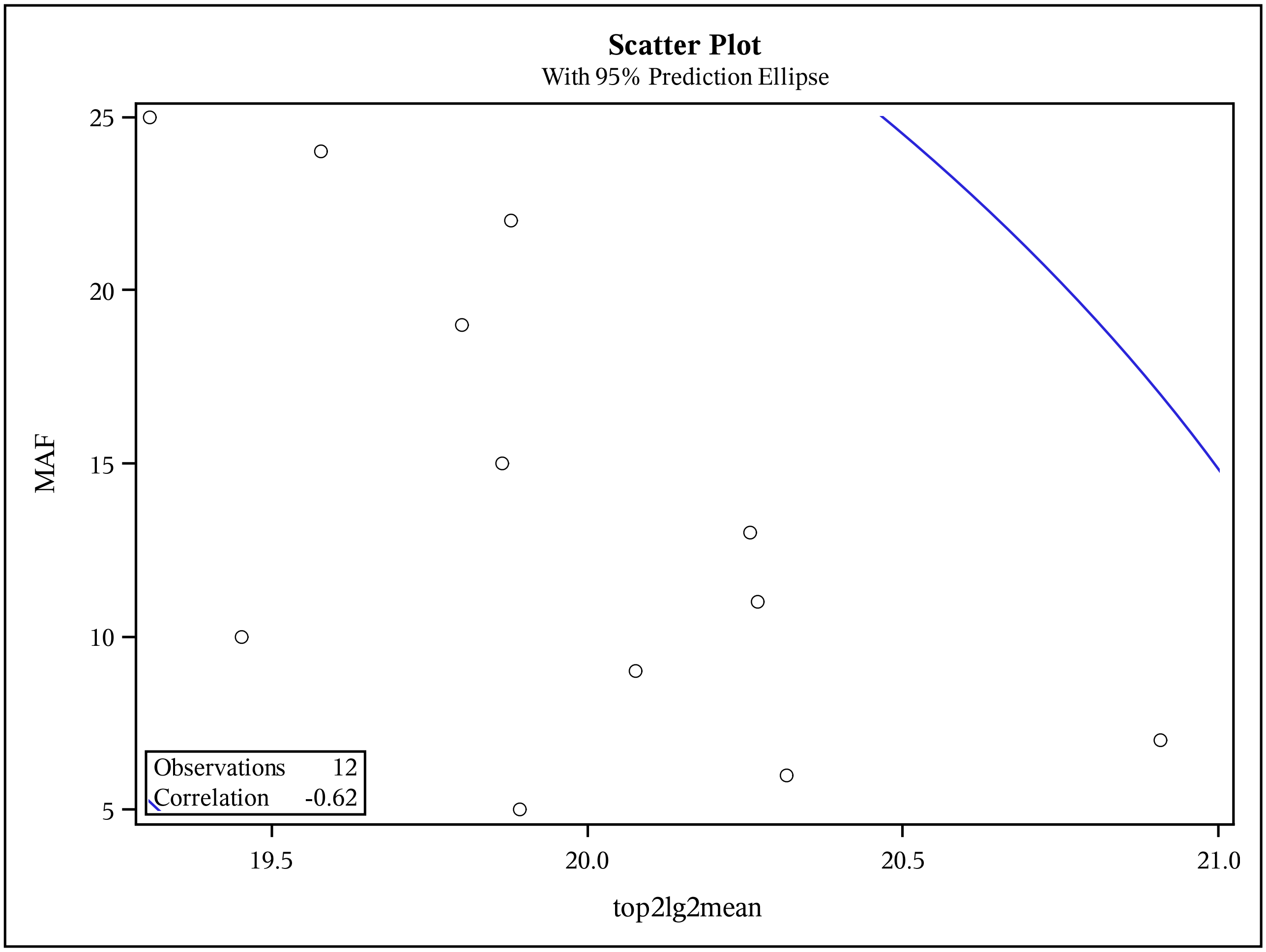

Correlation C_pooltop2lg2mean_tp2 symbol MAF

The CORR Procedure

Gene_Symbol=NEO1

**2 Variables:**	top2lg2mean MAF

Simple Statistics						
**Variable**	**N**	**Mean**	**Std Dev**	**Sum**	**Minimum**	**Maximum**
**top2lg2mean**	12	18.63564	0.41217	223.62765	17.85830	19.26165
**MAF**	12	13.83333	7.10740	166.00000	5.00000	25.00000

Pearson Correlation Coefficients, N = 12<br>Prob > |r| under H0: Rho=0		
	**top2lg2mean**	**MAF**
**top2lg2mean**	1.00000	0.23728<br>0.4577
**MAF**	0.23728<br>0.4577	1.00000

Pearson Correlation Statistics (Fisher's z Transformation)									
**Variable**	**With Variable**	**N**	**Sample Correlation**	**Fisher's z**	**Bias Adjustment**	**Correlation Estimate**	**95% Confidence Limits**		**p Value for H0:Rho=0**
**top2lg2mean**	**MAF**	12	0.23728	0.24189	0.01079	0.22708	-0.398794	0.708631	0.4680

Correlation C_pooltop2lg2mean_tp2 symbol MAF

The CORR Procedure

Gene_Symbol=NEO1

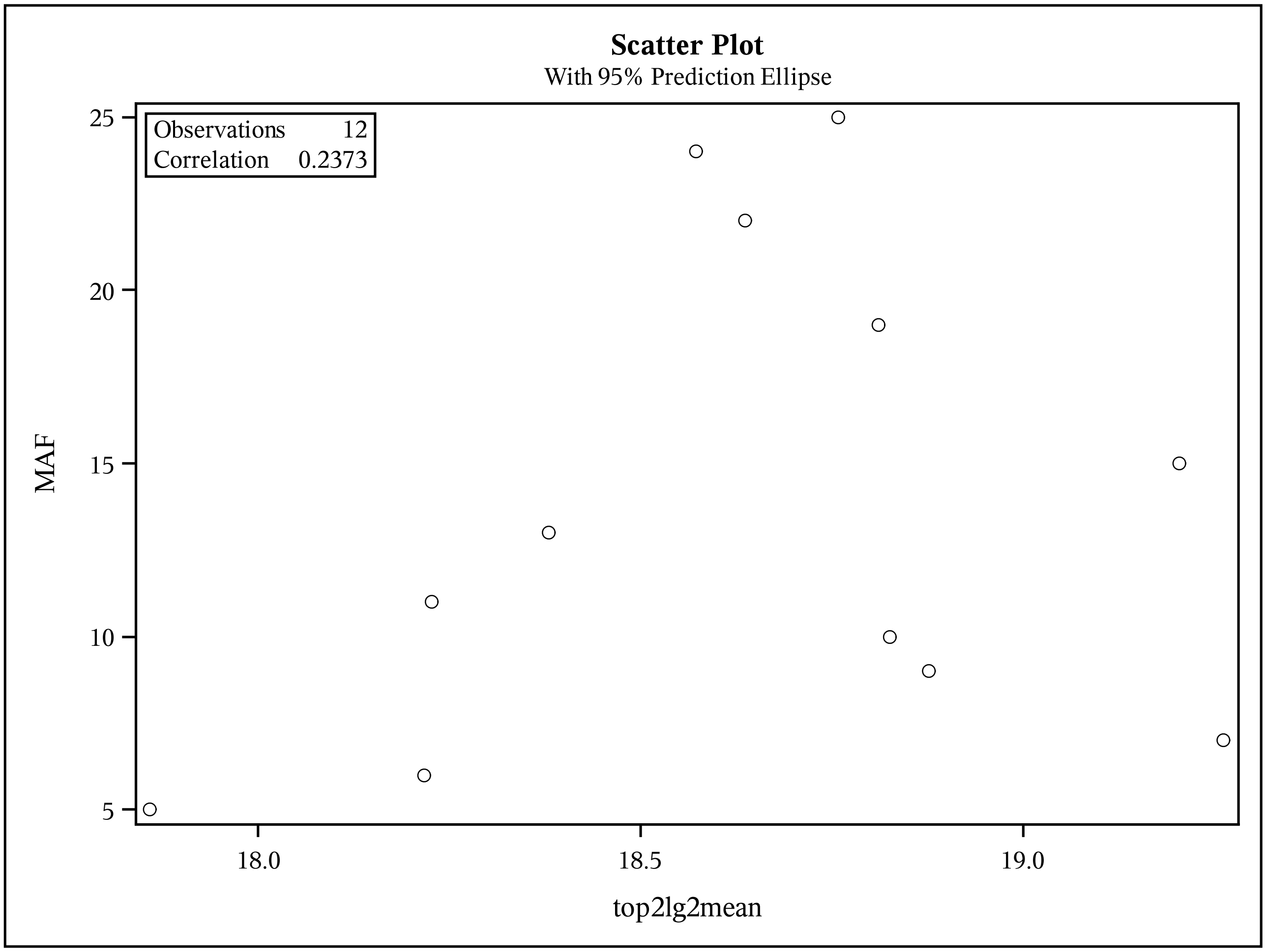

Correlation C_pooltop2lg2mean_tp2 symbol MAF

The CORR Procedure

Gene_Symbol=NPC2

**2 Variables:**	top2lg2mean MAF

Simple Statistics						
**Variable**	**N**	**Mean**	**Std Dev**	**Sum**	**Minimum**	**Maximum**
**top2lg2mean**	12	19.67006	0.50896	236.04070	18.82205	20.78194
**MAF**	12	13.83333	7.10740	166.00000	5.00000	25.00000

Pearson Correlation Coefficients, N = 12<br>Prob > |r| under H0: Rho=0		
	**top2lg2mean**	**MAF**
**top2lg2mean**	1.00000	0.08982<br>0.7813
**MAF**	0.08982<br>0.7813	1.00000

Pearson Correlation Statistics (Fisher's z Transformation)									
**Variable**	**With Variable**	**N**	**Sample Correlation**	**Fisher's z**	**Bias Adjustment**	**Correlation Estimate**	**95% Confidence Limits**		**p Value for H0:Rho=0**
**top2lg2mean**	**MAF**	12	0.08982	0.09006	0.00408	0.08577	-0.513405	0.628722	0.7870

Correlation C_pooltop2lg2mean_tp2 symbol MAF

The CORR Procedure

Gene_Symbol=NPC2

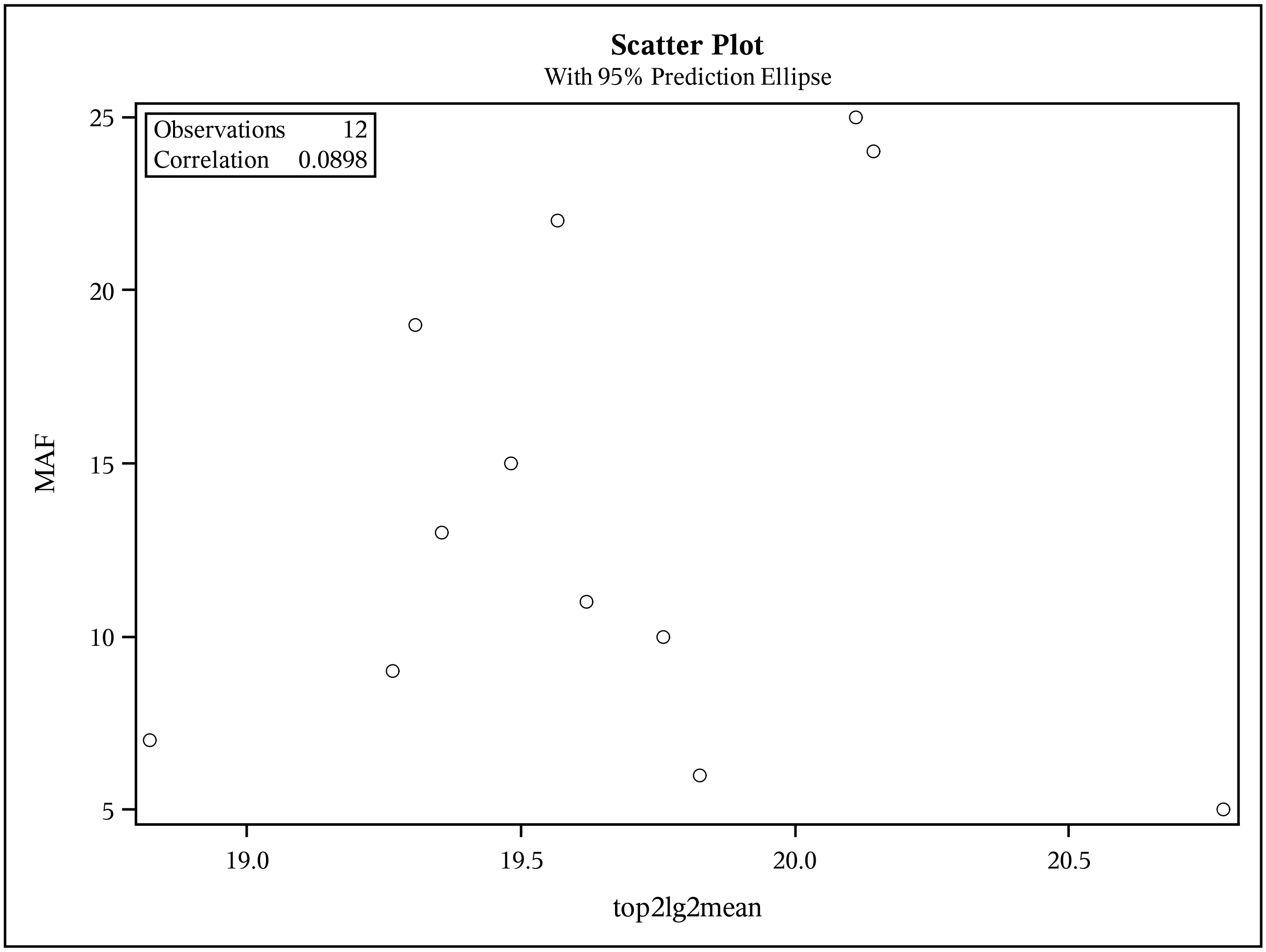

Correlation C_pooltop2lg2mean_tp2 symbol MAF

The CORR Procedure

Gene_Symbol=NRCAM

**2 Variables:**	top2lg2mean MAF

Simple Statistics						
**Variable**	**N**	**Mean**	**Std Dev**	**Sum**	**Minimum**	**Maximum**
**top2lg2mean**	12	20.82374	0.27138	249.88485	20.37181	21.20853
**MAF**	12	13.83333	7.10740	166.00000	5.00000	25.00000

Pearson Correlation Coefficients, N = 12<br>Prob > |r| under H0: Rho=0		
	**top2lg2mean**	**MAF**
**top2lg2mean**	1.00000	0.38890<br>0.2115
**MAF**	0.38890<br>0.2115	1.00000

Pearson Correlation Statistics (Fisher's z Transformation)									
**Variable**	**With Variable**	**N**	**Sample Correlation**	**Fisher's z**	**Bias Adjustment**	**Correlation Estimate**	**95% Confidence Limits**		**p Value for H0:Rho=0**
**top2lg2mean**	**MAF**	12	0.38890	0.41051	0.01768	0.37380	-0.254756	0.780305	0.2181

Correlation C_pooltop2lg2mean_tp2 symbol MAF

The CORR Procedure

Gene_Symbol=NRCAM

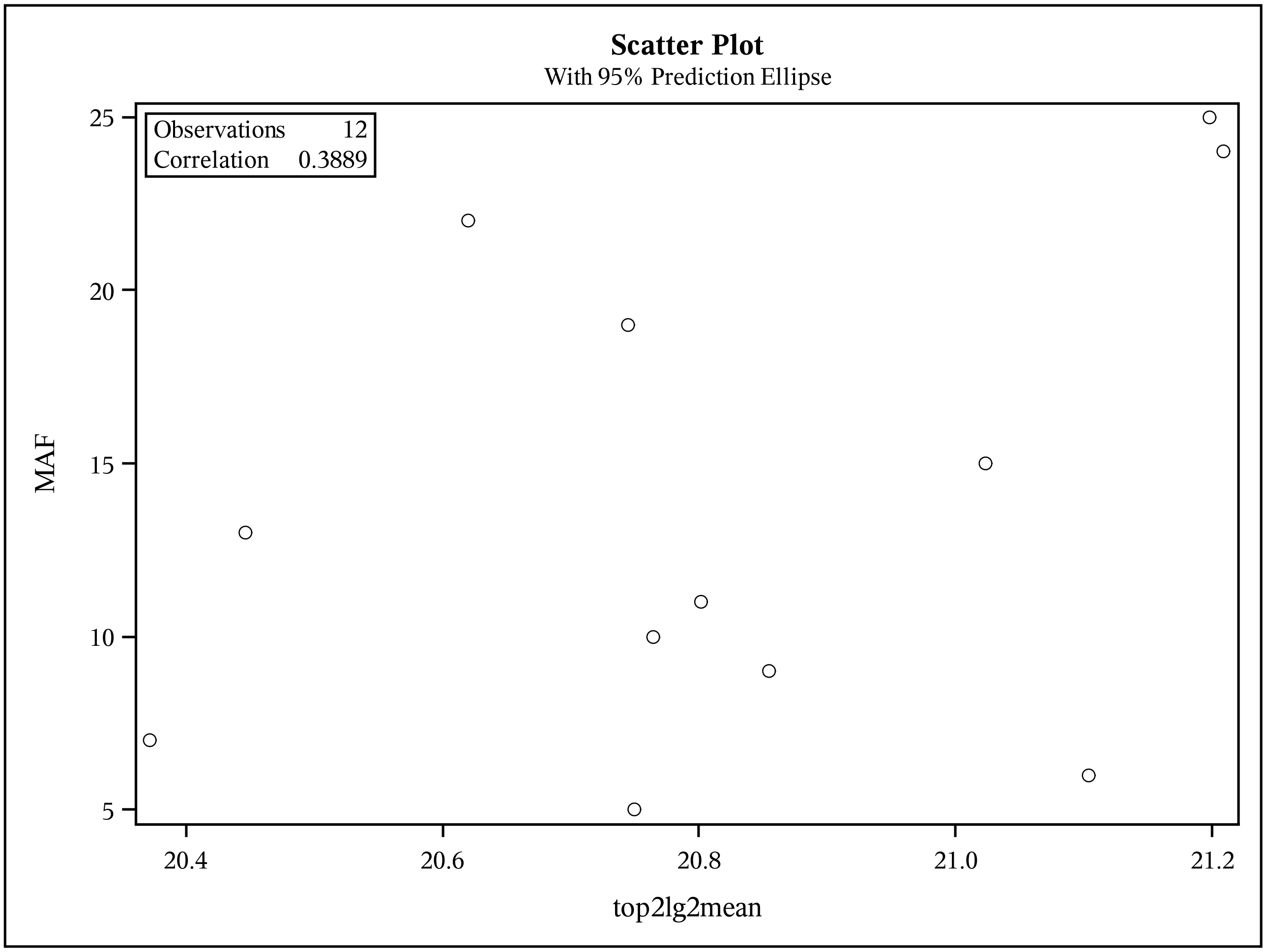

Correlation C_pooltop2lg2mean_tp2 symbol MAF

The CORR Procedure

Gene_Symbol=NTM

**2 Variables:**	top2lg2mean MAF

Simple Statistics						
**Variable**	**N**	**Mean**	**Std Dev**	**Sum**	**Minimum**	**Maximum**
**top2lg2mean**	12	19.04428	0.32215	228.53139	18.56987	19.55018
**MAF**	12	13.83333	7.10740	166.00000	5.00000	25.00000

Pearson Correlation Coefficients, N = 12<br>Prob > |r| under H0: Rho=0		
	**top2lg2mean**	**MAF**
**top2lg2mean**	1.00000	0.17001<br>0.5973
**MAF**	0.17001<br>0.5973	1.00000

Pearson Correlation Statistics (Fisher's z Transformation)									
**Variable**	**With Variable**	**N**	**Sample Correlation**	**Fisher's z**	**Bias Adjustment**	**Correlation Estimate**	**95% Confidence Limits**		**p Value for H0:Rho=0**
**top2lg2mean**	**MAF**	12	0.17001	0.17168	0.00773	0.16250	-0.453716	0.673583	0.6065

Correlation C_pooltop2lg2mean_tp2 symbol MAF

The CORR Procedure

Gene_Symbol=NTM

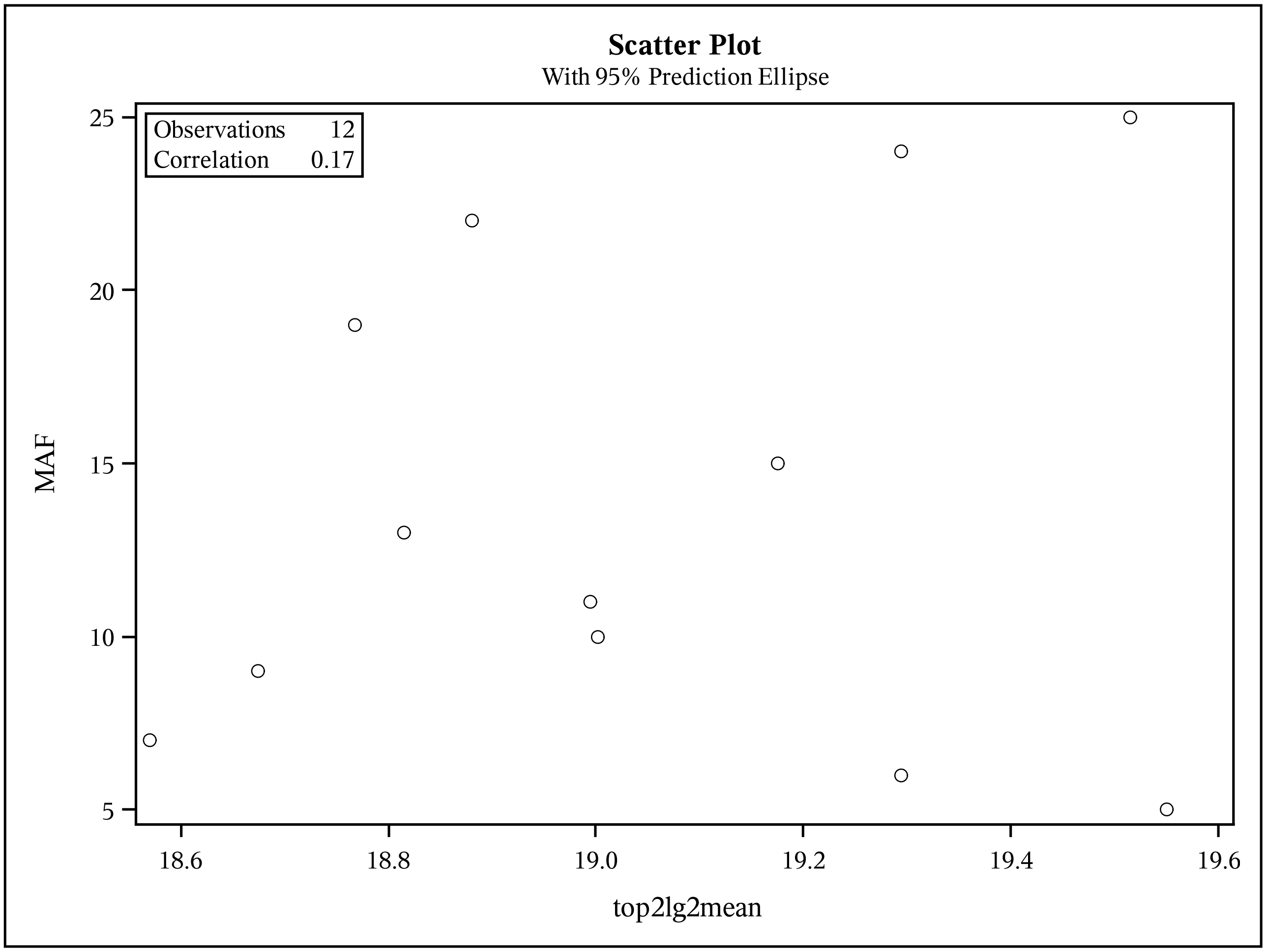

Correlation C_pooltop2lg2mean_tp2 symbol MAF

The CORR Procedure

Gene_Symbol=OGN

**2 Variables:**	top2lg2mean MAF

Simple Statistics						
**Variable**	**N**	**Mean**	**Std Dev**	**Sum**	**Minimum**	**Maximum**
**top2lg2mean**	12	20.80877	0.64074	249.70526	19.75513	22.16848
**MAF**	12	13.83333	7.10740	166.00000	5.00000	25.00000

Pearson Correlation Coefficients, N = 12<br>Prob > |r| under H0: Rho=0		
	**top2lg2mean**	**MAF**
**top2lg2mean**	1.00000	-0.56498<br>0.0556
**MAF**	-0.56498<br>0.0556	1.00000

Pearson Correlation Statistics (Fisher's z Transformation)									
**Variable**	**With Variable**	**N**	**Sample Correlation**	**Fisher's z**	**Bias Adjustment**	**Correlation Estimate**	**95% Confidence Limits**		**p Value for H0:Rho=0**
**top2lg2mean**	**MAF**	12	-0.56498	-0.64011	-0.02568	-0.54724	-0.853188	0.038869	0.0548

Correlation C_pooltop2lg2mean_tp2 symbol MAF

The CORR Procedure

Gene_Symbol=OGN

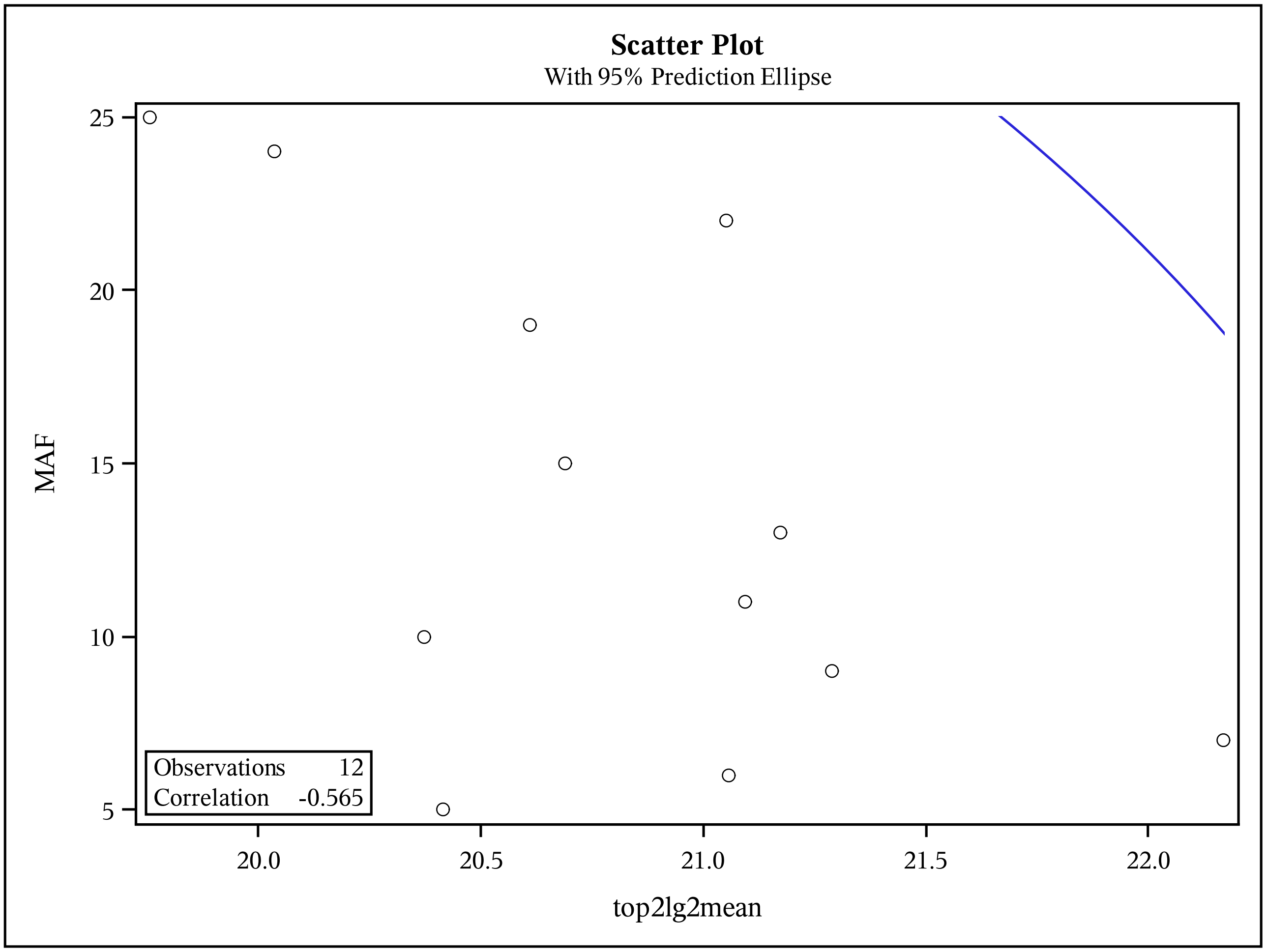

Correlation C_pooltop2lg2mean_tp2 symbol MAF

The CORR Procedure

Gene_Symbol=ORM1

**2 Variables:**	top2lg2mean MAF

Simple Statistics						
**Variable**	**N**	**Mean**	**Std Dev**	**Sum**	**Minimum**	**Maximum**
**top2lg2mean**	12	25.27837	0.18764	303.34041	24.93241	25.59154
**MAF**	12	13.83333	7.10740	166.00000	5.00000	25.00000

Pearson Correlation Coefficients, N = 12<br>Prob > |r| under H0: Rho=0		
	**top2lg2mean**	**MAF**
**top2lg2mean**	1.00000	0.54016<br>0.0698
**MAF**	0.54016<br>0.0698	1.00000

Pearson Correlation Statistics (Fisher's z Transformation)									
**Variable**	**With Variable**	**N**	**Sample Correlation**	**Fisher's z**	**Bias Adjustment**	**Correlation Estimate**	**95% Confidence Limits**		**p Value for H0:Rho=0**
**top2lg2mean**	**MAF**	12	0.54016	0.60438	0.02455	0.52254	-0.073361	0.843490	0.0698

Correlation C_pooltop2lg2mean_tp2 symbol MAF

The CORR Procedure

Gene_Symbol=ORM1

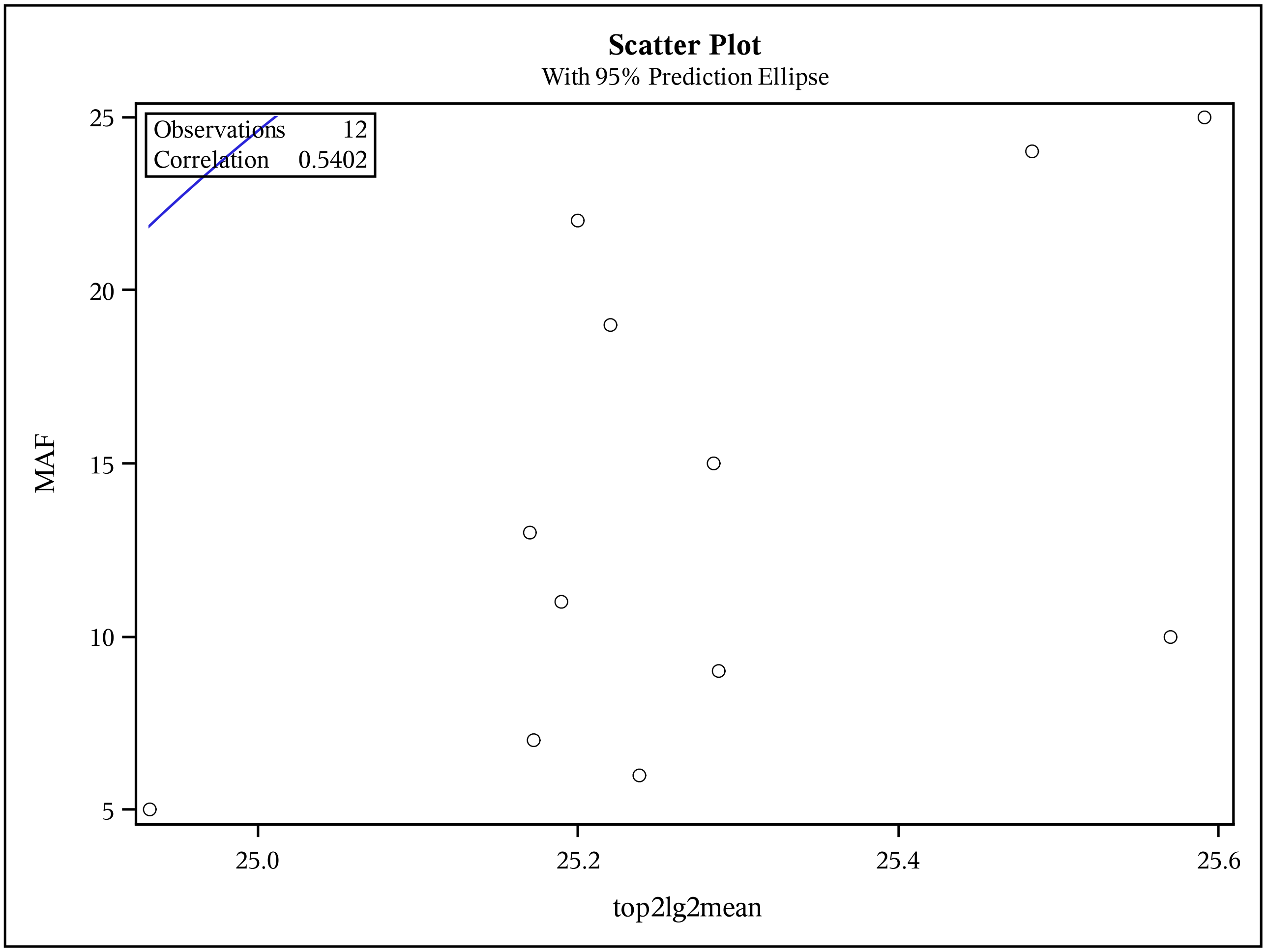

Correlation C_pooltop2lg2mean_tp2 symbol MAF

The CORR Procedure

Gene_Symbol=PKM2

**2 Variables:**	top2lg2mean MAF

Simple Statistics						
**Variable**	**N**	**Mean**	**Std Dev**	**Sum**	**Minimum**	**Maximum**
**top2lg2mean**	12	16.02777	0.55684	192.33324	15.22430	17.32496
**MAF**	12	13.83333	7.10740	166.00000	5.00000	25.00000

Pearson Correlation Coefficients, N = 12<br>Prob > |r| under H0: Rho=0		
	**top2lg2mean**	**MAF**
**top2lg2mean**	1.00000	-0.30041<br>0.3428
**MAF**	-0.30041<br>0.3428	1.00000

Pearson Correlation Statistics (Fisher's z Transformation)									
**Variable**	**With Variable**	**N**	**Sample Correlation**	**Fisher's z**	**Bias Adjustment**	**Correlation Estimate**	**95% Confidence Limits**		**p Value for H0:Rho=0**
**top2lg2mean**	**MAF**	12	-0.30041	-0.30997	-0.01365	-0.28793	-0.739616	0.342578	0.3524

Correlation C_pooltop2lg2mean_tp2 symbol MAF

The CORR Procedure

Gene_Symbol=PKM2

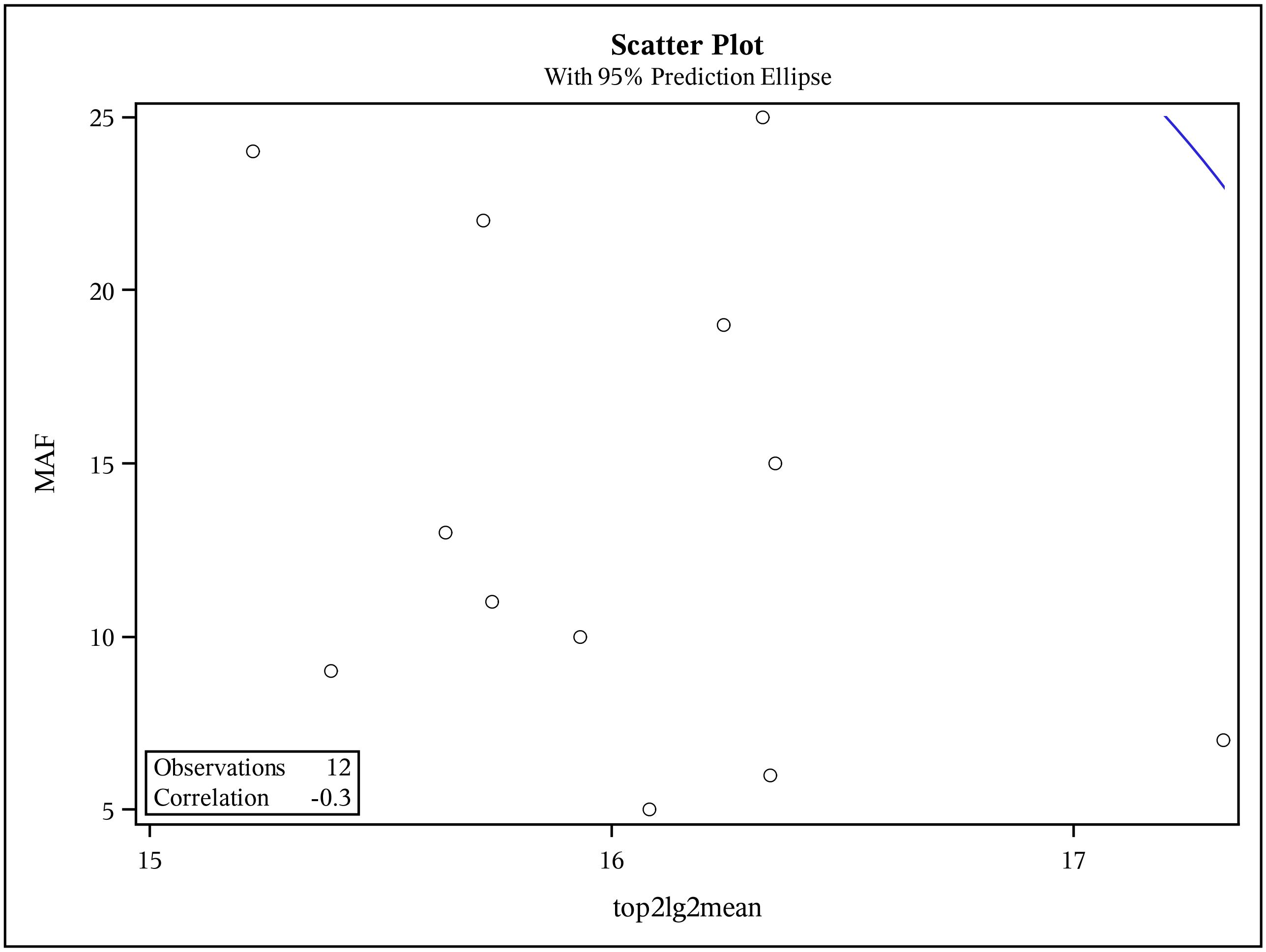

Correlation C_pooltop2lg2mean_tp2 symbol MAF

The CORR Procedure

Gene_Symbol=PLG

**2 Variables:**	top2lg2mean MAF

Simple Statistics						
**Variable**	**N**	**Mean**	**Std Dev**	**Sum**	**Minimum**	**Maximum**
**top2lg2mean**	12	20.94222	0.16342	251.30666	20.64623	21.19274
**MAF**	12	13.83333	7.10740	166.00000	5.00000	25.00000

Pearson Correlation Coefficients, N = 12<br>Prob > |r| under H0: Rho=0		
	**top2lg2mean**	**MAF**
**top2lg2mean**	1.00000	0.19839<br>0.5365
**MAF**	0.19839<br>0.5365	1.00000

Pearson Correlation Statistics (Fisher's z Transformation)									
**Variable**	**With Variable**	**N**	**Sample Correlation**	**Fisher's z**	**Bias Adjustment**	**Correlation Estimate**	**95% Confidence Limits**		**p Value for H0:Rho=0**
**top2lg2mean**	**MAF**	12	0.19839	0.20105	0.00902	0.18971	-0.431133	0.688635	0.5464

Correlation C_pooltop2lg2mean_tp2 symbol MAF

The CORR Procedure

Gene_Symbol=PLG

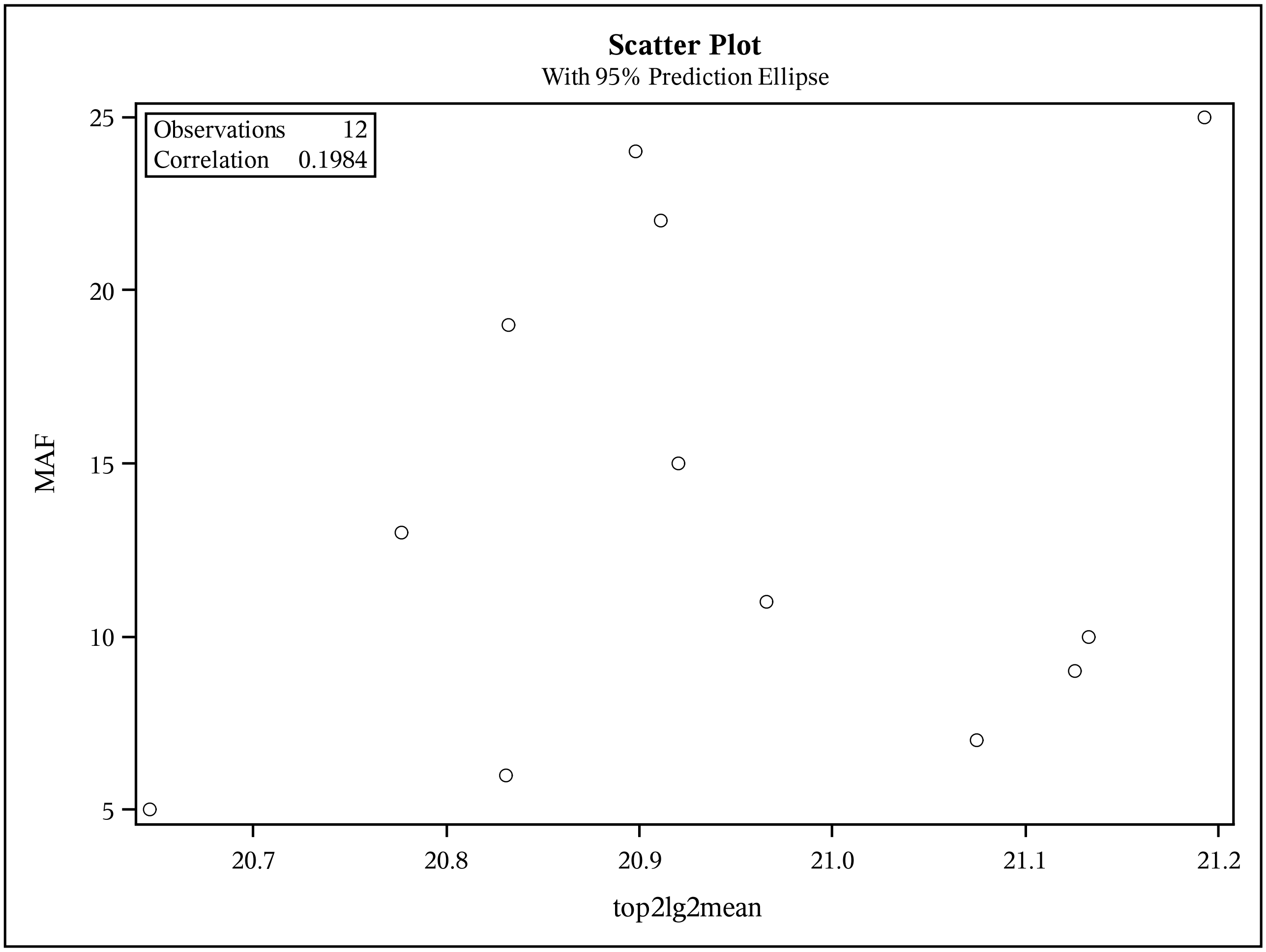

Correlation C_pooltop2lg2mean_tp2 symbol MAF

The CORR Procedure

Gene_Symbol=PSAP

**2 Variables:**	top2lg2mean MAF

Simple Statistics						
**Variable**	**N**	**Mean**	**Std Dev**	**Sum**	**Minimum**	**Maximum**
**top2lg2mean**	12	17.99037	0.31361	215.88439	17.41062	18.37198
**MAF**	12	13.83333	7.10740	166.00000	5.00000	25.00000

Pearson Correlation Coefficients, N = 12<br>Prob > |r| under H0: Rho=0		
	**top2lg2mean**	**MAF**
**top2lg2mean**	1.00000	-0.35563<br>0.2566
**MAF**	-0.35563<br>0.2566	1.00000

Pearson Correlation Statistics (Fisher's z Transformation)									
**Variable**	**With Variable**	**N**	**Sample Correlation**	**Fisher's z**	**Bias Adjustment**	**Correlation Estimate**	**95% Confidence Limits**		**p Value for H0:Rho=0**
**top2lg2mean**	**MAF**	12	-0.35563	-0.37187	-0.01616	-0.34143	-0.765360	0.289128	0.2646

Correlation C_pooltop2lg2mean_tp2 symbol MAF

The CORR Procedure

Gene_Symbol=PSAP

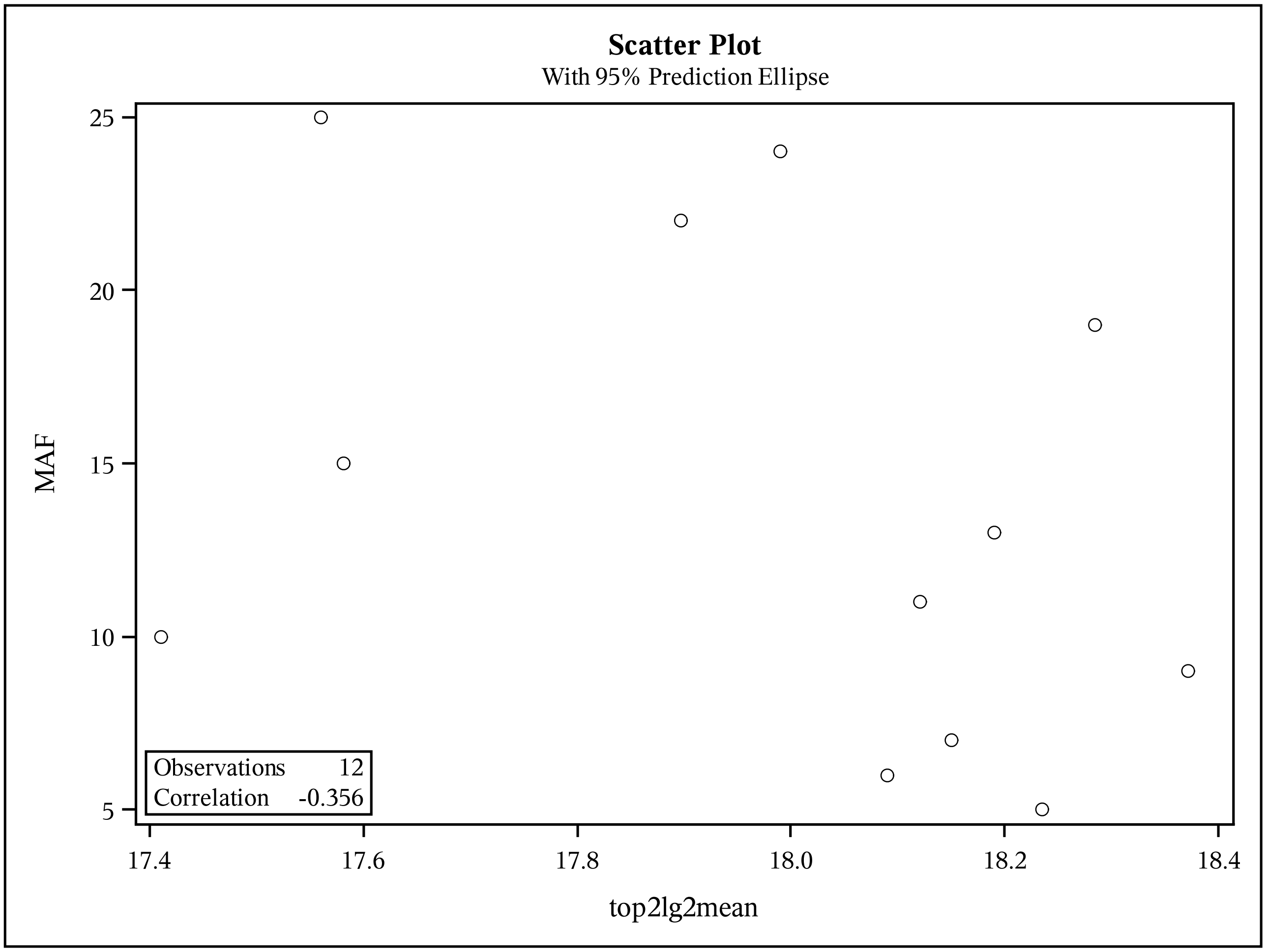

Correlation C_pooltop2lg2mean_tp2 symbol MAF

The CORR Procedure

Gene_Symbol=PTGDS

**2 Variables:**	top2lg2mean MAF

Simple Statistics						
**Variable**	**N**	**Mean**	**Std Dev**	**Sum**	**Minimum**	**Maximum**
**top2lg2mean**	12	23.74446	0.21616	284.93356	23.35282	24.11171
**MAF**	12	13.83333	7.10740	166.00000	5.00000	25.00000

Pearson Correlation Coefficients, N = 12<br>Prob > |r| under H0: Rho=0		
	**top2lg2mean**	**MAF**
**top2lg2mean**	1.00000	0.44208<br>0.1502
**MAF**	0.44208<br>0.1502	1.00000

Pearson Correlation Statistics (Fisher's z Transformation)									
**Variable**	**With Variable**	**N**	**Sample Correlation**	**Fisher's z**	**Bias Adjustment**	**Correlation Estimate**	**95% Confidence Limits**		**p Value for H0:Rho=0**
**top2lg2mean**	**MAF**	12	0.44208	0.47481	0.02009	0.42577	-0.196038	0.803366	0.1543

Correlation C_pooltop2lg2mean_tp2 symbol MAF

The CORR Procedure

Gene_Symbol=PTGDS

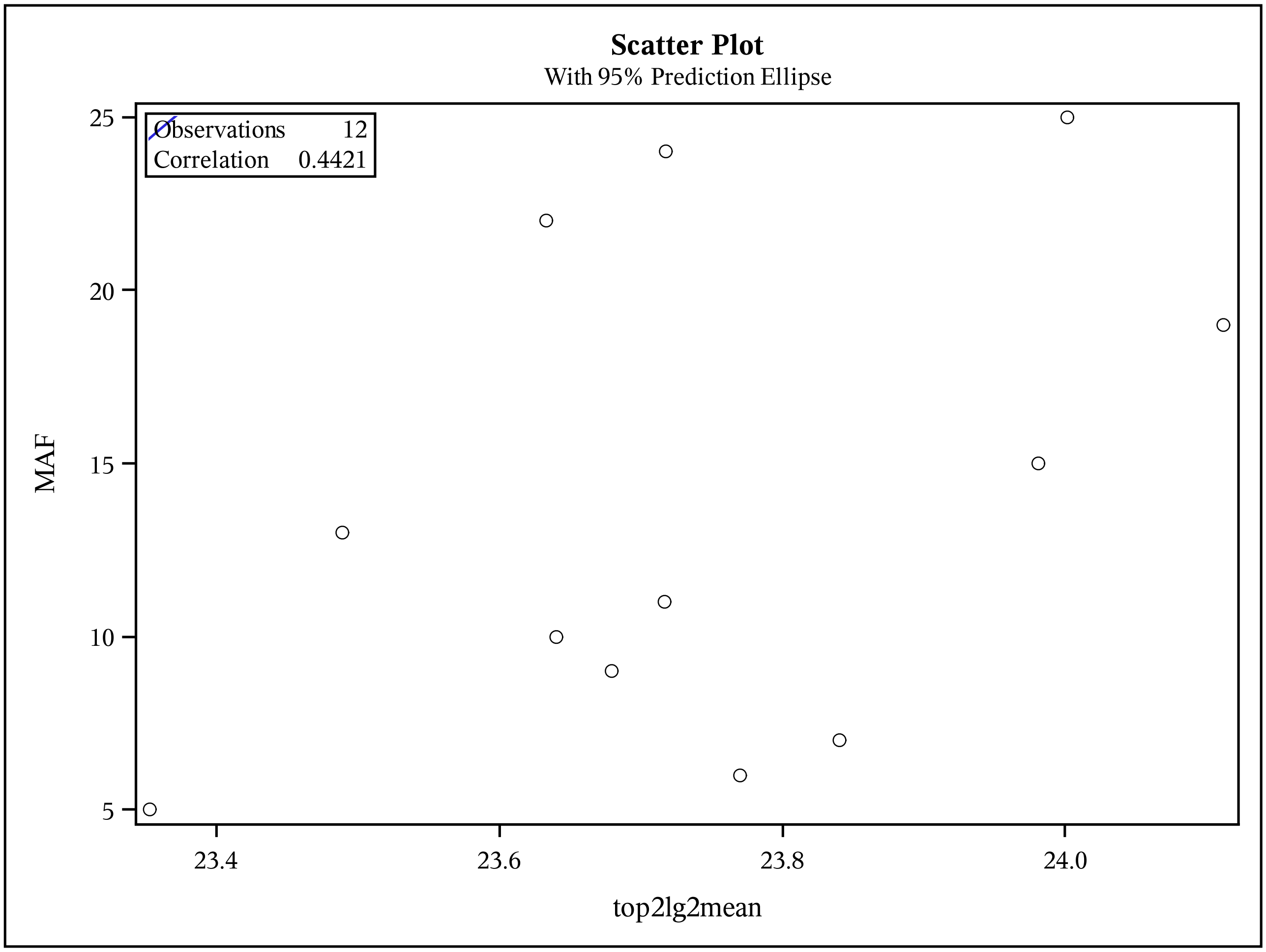

Correlation C_pooltop2lg2mean_tp2 symbol MAF

The CORR Procedure

Gene_Symbol=RBP4

2 Variables:	top2lg2mean MAF

Simple Statistics						
Variable	N	Mean	Std Dev	Sum	Minimum	Maximum
top2lg2mean	12	20.68553	0.17505	248.22639	20.30637	20.87414
MAF	12	13.83333	7.10740	166.00000	5.00000	25.00000

Pearson Correlation Coefficients, N = 12<br>Prob > |r| under H0: Rho=0		
	top2lg2mean	MAF
top2lg2mean	1.00000	0.13563<br>0.6743
MAF	0.13563<br>0.6743	1.00000

Pearson Correlation Statistics (Fisher's z Transformation)									
Variable	With Variable	N	Sample Correlation	Fisher's z	Bias Adjustment	Correlation Estimate	95% Confidence Limits		p Value for H0:Rho=0
top2lg2mean	MAF	12	0.13563	0.13647	0.00617	0.12958	-0.480023	0.654785	0.6822

Correlation C_pooltop2lg2mean_tp2 symbol MAF

The CORR Procedure

Gene_Symbol=RBP4

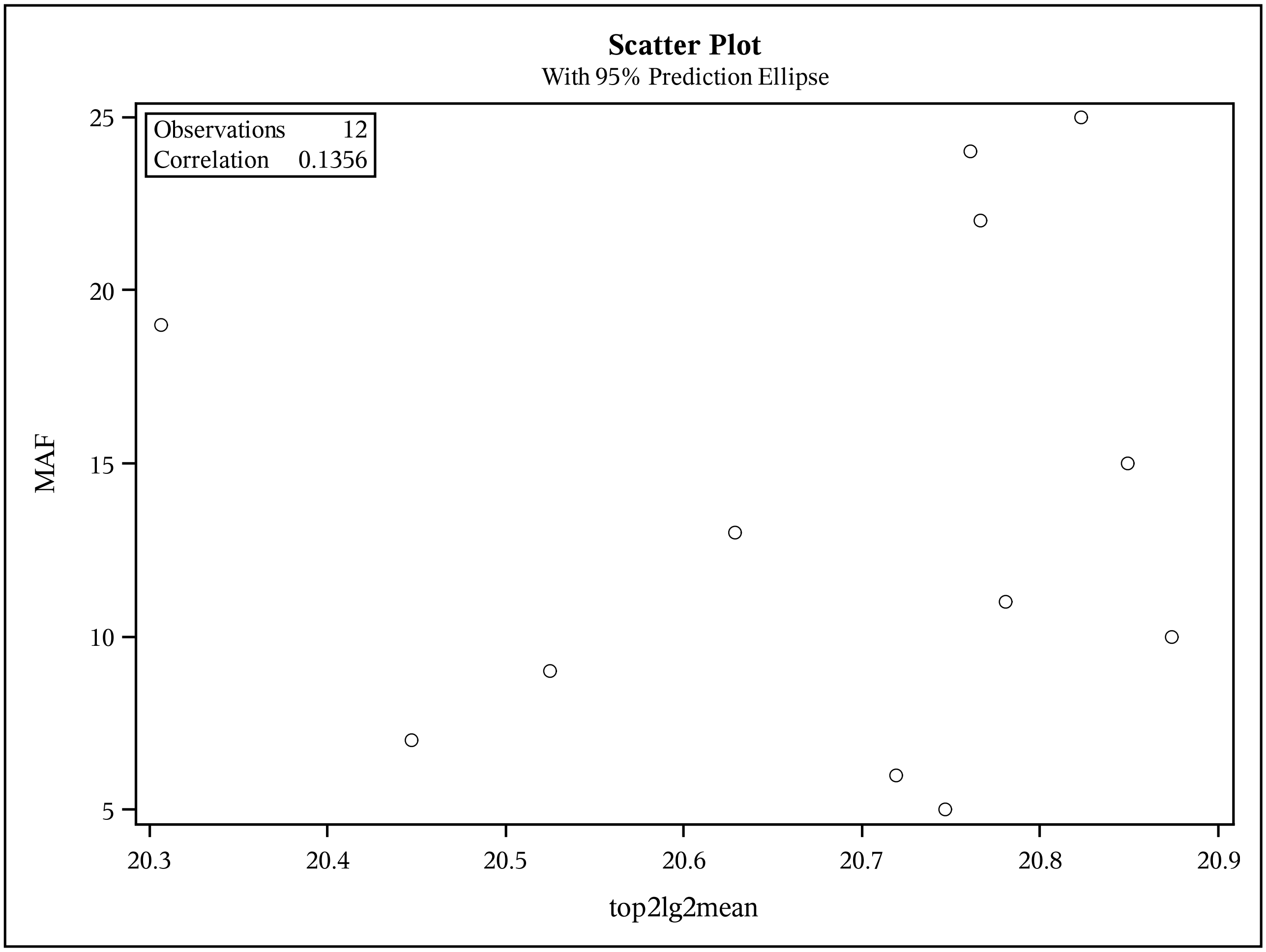

Correlation C_pooltop2lg2mean_tp2 symbol MAF

The CORR Procedure

Gene_Symbol=RNASE1

**2 Variables:**	top2lg2mean MAF

Simple Statistics						
**Variable**	**N**	**Mean**	**Std Dev**	**Sum**	**Minimum**	**Maximum**
**top2lg2mean**	12	22.32073	0.75803	267.84872	21.06036	23.40056
**MAF**	12	13.83333	7.10740	166.00000	5.00000	25.00000

Pearson Correlation Coefficients, N = 12<br>Prob > |r| under H0: Rho=0		
	**top2lg2mean**	**MAF**
**top2lg2mean**	1.00000	-0.52065<br>0.0826
**MAF**	-0.52065<br>0.0826	1.00000

Pearson Correlation Statistics (Fisher's z Transformation)									
**Variable**	**With Variable**	**N**	**Sample Correlation**	**Fisher's z**	**Bias Adjustment**	**Correlation Estimate**	**95% Confidence Limits**		**p Value for H0:Rho=0**
**top2lg2mean**	**MAF**	12	-0.52065	-0.57724	-0.02367	-0.50319	-0.835745	0.099421	0.0833

Correlation C_pooltop2lg2mean_tp2 symbol MAF

The CORR Procedure

Gene_Symbol=RNASE1

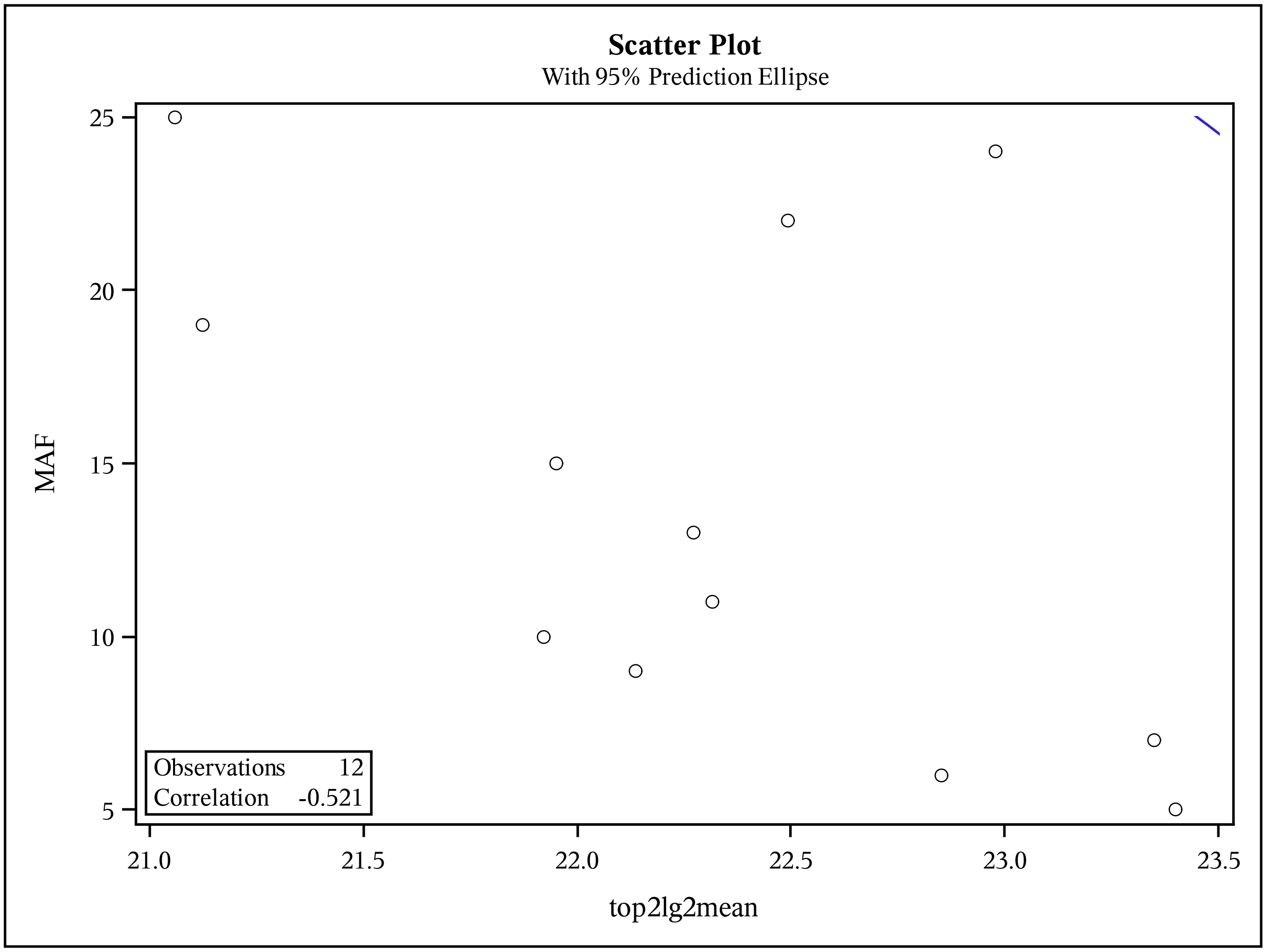

Correlation C_pooltop2lg2mean_tp2 symbol MAF

The CORR Procedure

Gene_Symbol=SCG3

**2 Variables:**	top2lg2mean MAF

Simple Statistics						
**Variable**	**N**	**Mean**	**Std Dev**	**Sum**	**Minimum**	**Maximum**
**top2lg2mean**	12	20.88501	0.26995	250.62014	20.48278	21.33450
**MAF**	12	13.83333	7.10740	166.00000	5.00000	25.00000

Pearson Correlation Coefficients, N = 12<br>Prob > |r| under H0: Rho=0		
	**top2lg2mean**	**MAF**
**top2lg2mean**	1.00000	0.19128<br>0.5515
**MAF**	0.19128<br>0.5515	1.00000

Pearson Correlation Statistics (Fisher's z Transformation)									
**Variable**	**With Variable**	**N**	**Sample Correlation**	**Fisher's z**	**Bias Adjustment**	**Correlation Estimate**	**95% Confidence Limits**		**p Value for H0:Rho=0**
**top2lg2mean**	**MAF**	12	0.19128	0.19367	0.00869	0.18289	-0.436865	0.684904	0.5612

Correlation C_pooltop2lg2mean_tp2 symbol MAF

The CORR Procedure

Gene_Symbol=SCG3

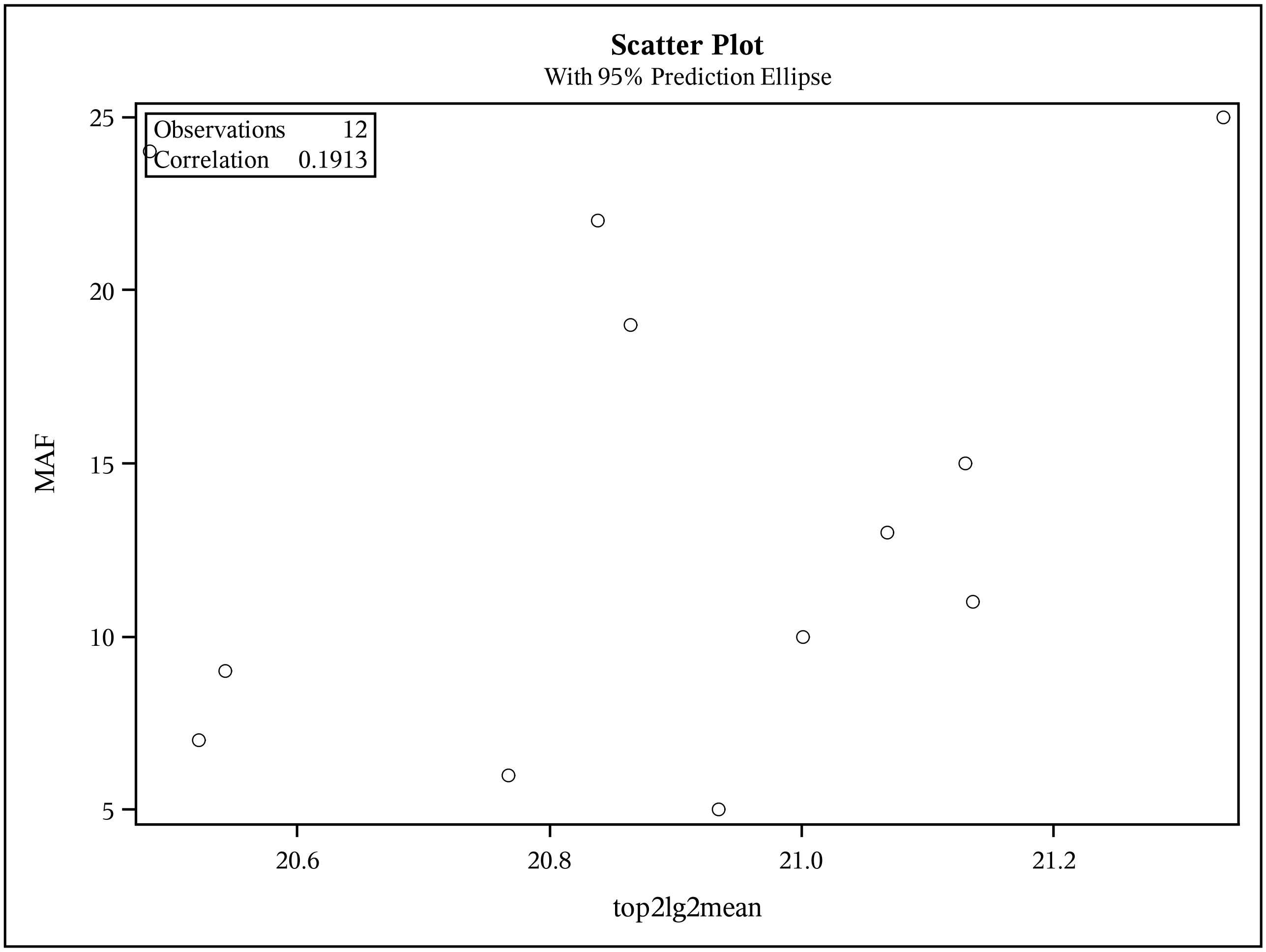

Correlation C_pooltop2lg2mean_tp2 symbol MAF

The CORR Procedure

Gene_Symbol=SERPINA3

2 Variables:	top2lg2mean MAF

Simple Statistics						
Variable	N	Mean	Std Dev	Sum	Minimum	Maximum
top2lg2mean	12	22.62516	0.32380	271.50187	22.10855	23.09064
MAF	12	13.83333	7.10740	166.00000	5.00000	25.00000

Pearson Correlation Coefficients, N = 12<br>Prob > |r| under H0: Rho=0		
	top2lg2mean	MAF
top2lg2mean	1.00000	0.00748<br>0.9816
MAF	0.00748<br>0.9816	1.00000

Pearson Correlation Statistics (Fisher's z Transformation)									
Variable	With Variable	N	Sample Correlation	Fisher's z	Bias Adjustment	Correlation Estimate	95% Confidence Limits		p Value for H0:Rho=0
top2lg2mean	MAF	12	0.00748	0.00748	0.0003398	0.00714	-0.569096	0.578668	0.9821

Correlation C_pooltop2lg2mean_tp2 symbol MAF

The CORR Procedure

Gene_Symbol=SERPINA3

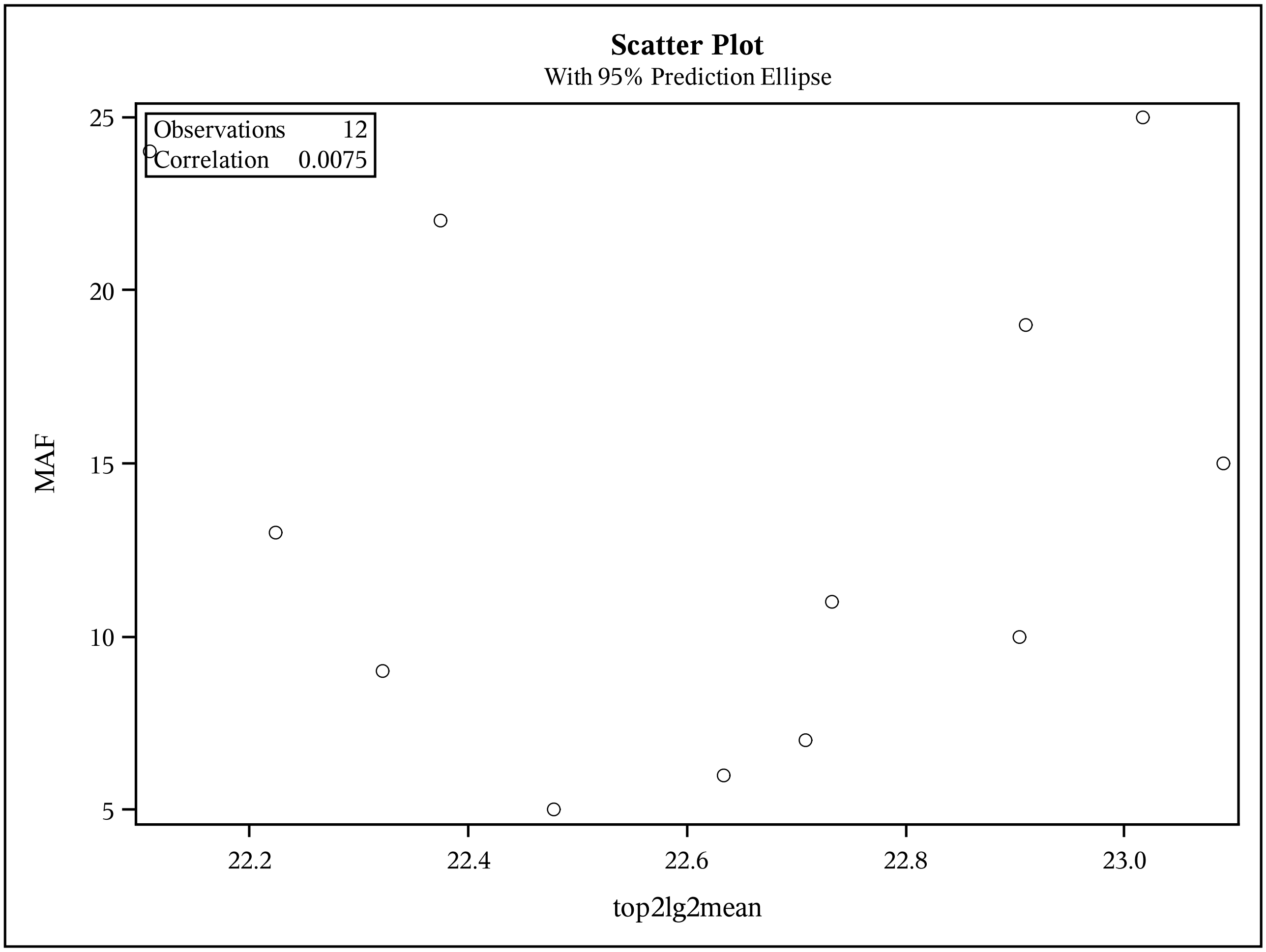

Correlation C_pooltop2lg2mean_tp2 symbol MAF

The CORR Procedure

Gene_Symbol=SERPINC1

**2 Variables:**	top2lg2mean MAF

Simple Statistics						
**Variable**	**N**	**Mean**	**Std Dev**	**Sum**	**Minimum**	**Maximum**
**top2lg2mean**	12	21.46794	0.16544	257.61532	21.17691	21.68697
**MAF**	12	13.83333	7.10740	166.00000	5.00000	25.00000

Pearson Correlation Coefficients, N = 12<br>Prob > |r| under H0: Rho=0		
	**top2lg2mean**	**MAF**
**top2lg2mean**	1.00000	0.27772<br>0.3821
**MAF**	0.27772<br>0.3821	1.00000

Pearson Correlation Statistics (Fisher's z Transformation)									
**Variable**	**With Variable**	**N**	**Sample Correlation**	**Fisher's z**	**Bias Adjustment**	**Correlation Estimate**	**95% Confidence Limits**		**p Value for H0:Rho=0**
**top2lg2mean**	**MAF**	12	0.27772	0.28521	0.01262	0.26603	-0.363349	0.728678	0.3922

Correlation C_pooltop2lg2mean_tp2 symbol MAF

The CORR Procedure

Gene_Symbol=SERPINC1

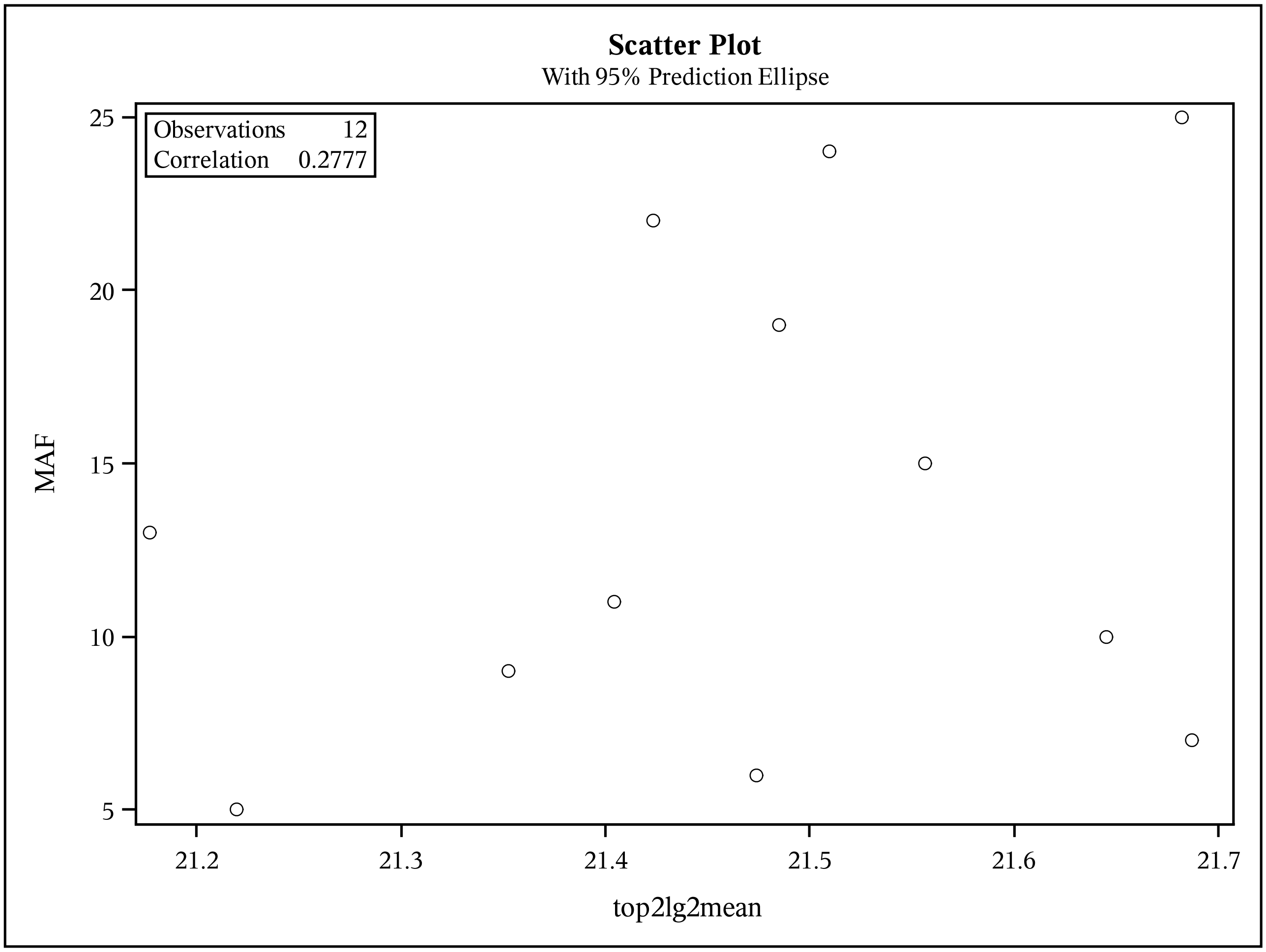

Correlation C_pooltop2lg2mean_tp2 symbol MAF

The CORR Procedure

Gene_Symbol=SERPIND1

**2 Variables:**	top2lg2mean MAF

Simple Statistics						
**Variable**	**N**	**Mean**	**Std Dev**	**Sum**	**Minimum**	**Maximum**
**top2lg2mean**	12	17.67289	0.55318	212.07465	16.75935	18.49608
**MAF**	12	13.83333	7.10740	166.00000	5.00000	25.00000

Pearson Correlation Coefficients, N = 12<br>Prob > |r| under H0: Rho=0		
	**top2lg2mean**	**MAF**
**top2lg2mean**	1.00000	0.16599<br>0.6061
**MAF**	0.16599<br>0.6061	1.00000

Pearson Correlation Statistics (Fisher's z Transformation)									
**Variable**	**With Variable**	**N**	**Sample Correlation**	**Fisher's z**	**Bias Adjustment**	**Correlation Estimate**	**95% Confidence Limits**		**p Value for H0:Rho=0**
**top2lg2mean**	**MAF**	12	0.16599	0.16754	0.00755	0.15865	-0.456849	0.671418	0.6152

Correlation C_pooltop2lg2mean_tp2 symbol MAF

The CORR Procedure

Gene_Symbol=SERPIND1

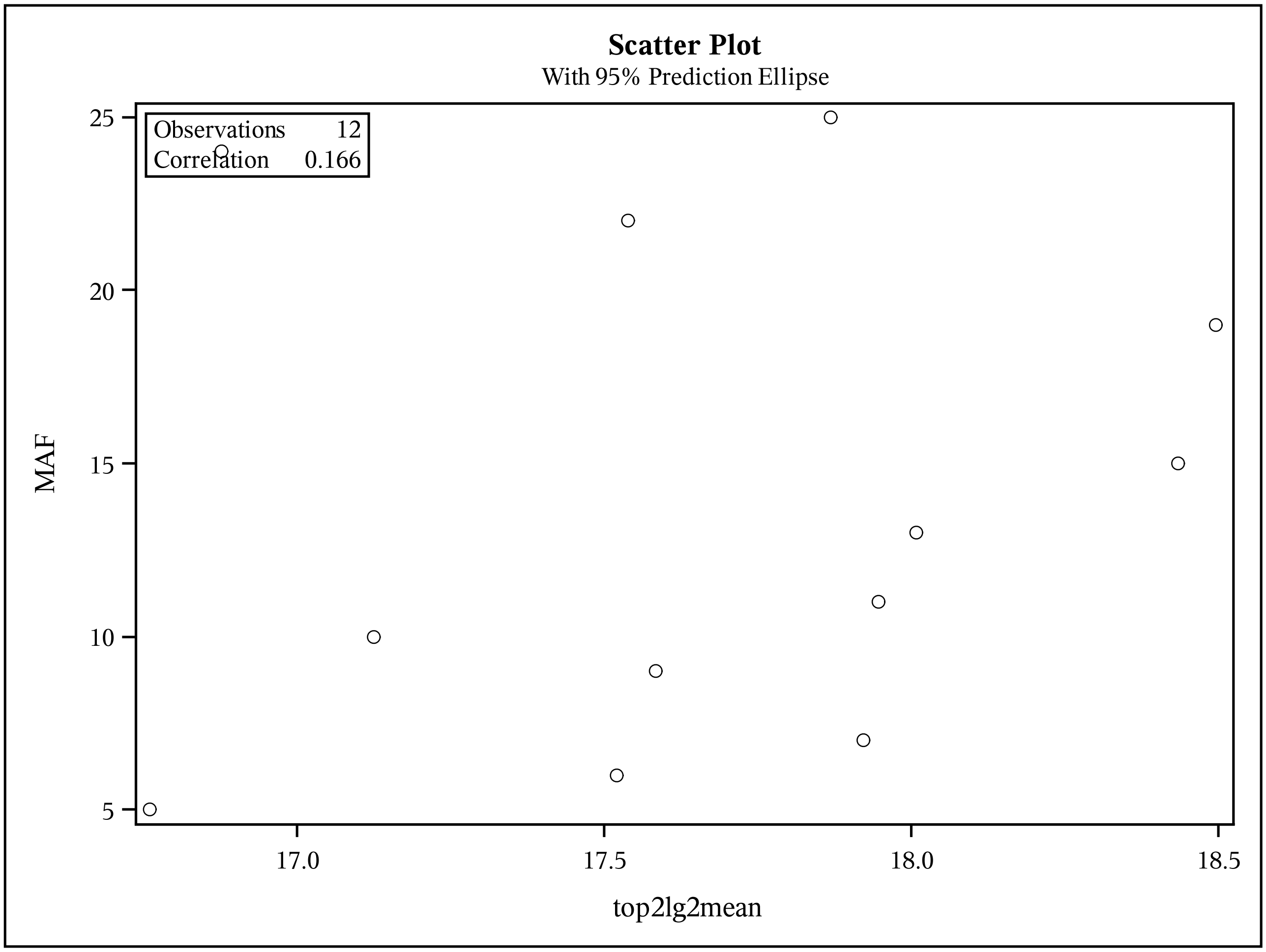

Correlation C_pooltop2lg2mean_tp2 symbol MAF

The CORR Procedure

Gene_Symbol=SERPINF1

2 Variables:	top2lg2mean MAF

Simple Statistics						
**Variable**	**N**	**Mean**	**Std Dev**	**Sum**	**Minimum**	**Maximum**
**top2lg2mean**	12	22.95120	0.25443	275.41434	22.55917	23.56111
**MAF**	12	13.83333	7.10740	166.00000	5.00000	25.00000

Pearson Correlation Coefficients, N = 12<br>Prob > |r| under H0: Rho=0		
	**top2lg2mean**	**MAF**
**top2lg2mean**	1.00000	-0.46203<br>0.1305
**MAF**	-0.46203<br>0.1305	1.00000

Pearson Correlation Statistics (Fisher's z Transformation)									
**Variable**	**With Variable**	**N**	**Sample Correlation**	**Fisher's z**	**Bias Adjustment**	**Correlation Estimate**	**95% Confidence Limits**		**p Value for H0:Rho=0**
**top2lg2mean**	**MAF**	12	-0.46203	-0.49988	-0.02100	-0.44535	-0.811772	0.172691	0.1337

Correlation C_pooltop2lg2mean_tp2 symbol MAF

The CORR Procedure

Gene_Symbol=SERPINF1

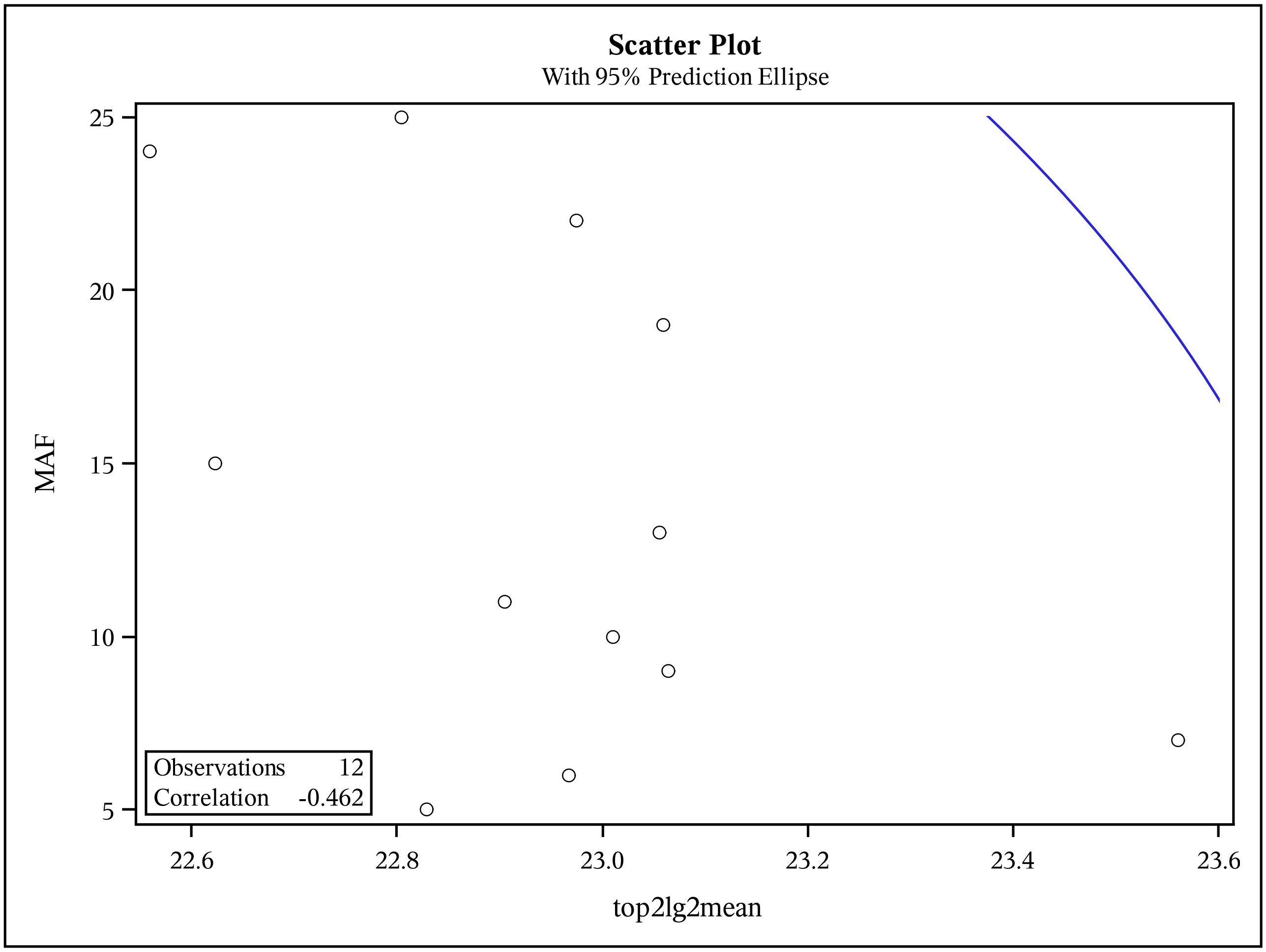

Correlation C_pooltop2lg2mean_tp2 symbol MAF

The CORR Procedure

Gene_Symbol=SERPING1

2 Variables:	top2lg2mean MAF

Simple Statistics						
Variable	N	Mean	Std Dev	Sum	Minimum	Maximum
top2lg2mean	12	21.81399	0.49183	261.76791	20.79293	22.57029
MAF	12	13.83333	7.10740	166.00000	5.00000	25.00000

Pearson Correlation Coefficients, N = 12<br>Prob > |r| under H0: Rho=0		
	top2lg2mean	MAF
top2lg2mean	1.00000	-0.07424<br>0.8186
MAF	-0.07424<br>0.8186	1.00000

Pearson Correlation Statistics (Fisher's z Transformation)										
Variable	With Variable	N	Sample Correlation	Fisher's z	Bias Adjustment	Correlation Estimate	95% Confidence Limits		p Value for H0:Rho=0
top2lg2mean	MAF	12	-0.07424	-0.07438	-0.00337	-0.07088	-0.619581	0.524348	0.8234

Correlation C_pooltop2lg2mean_tp2 symbol MAF

The CORR Procedure

Gene_Symbol=SERPING1

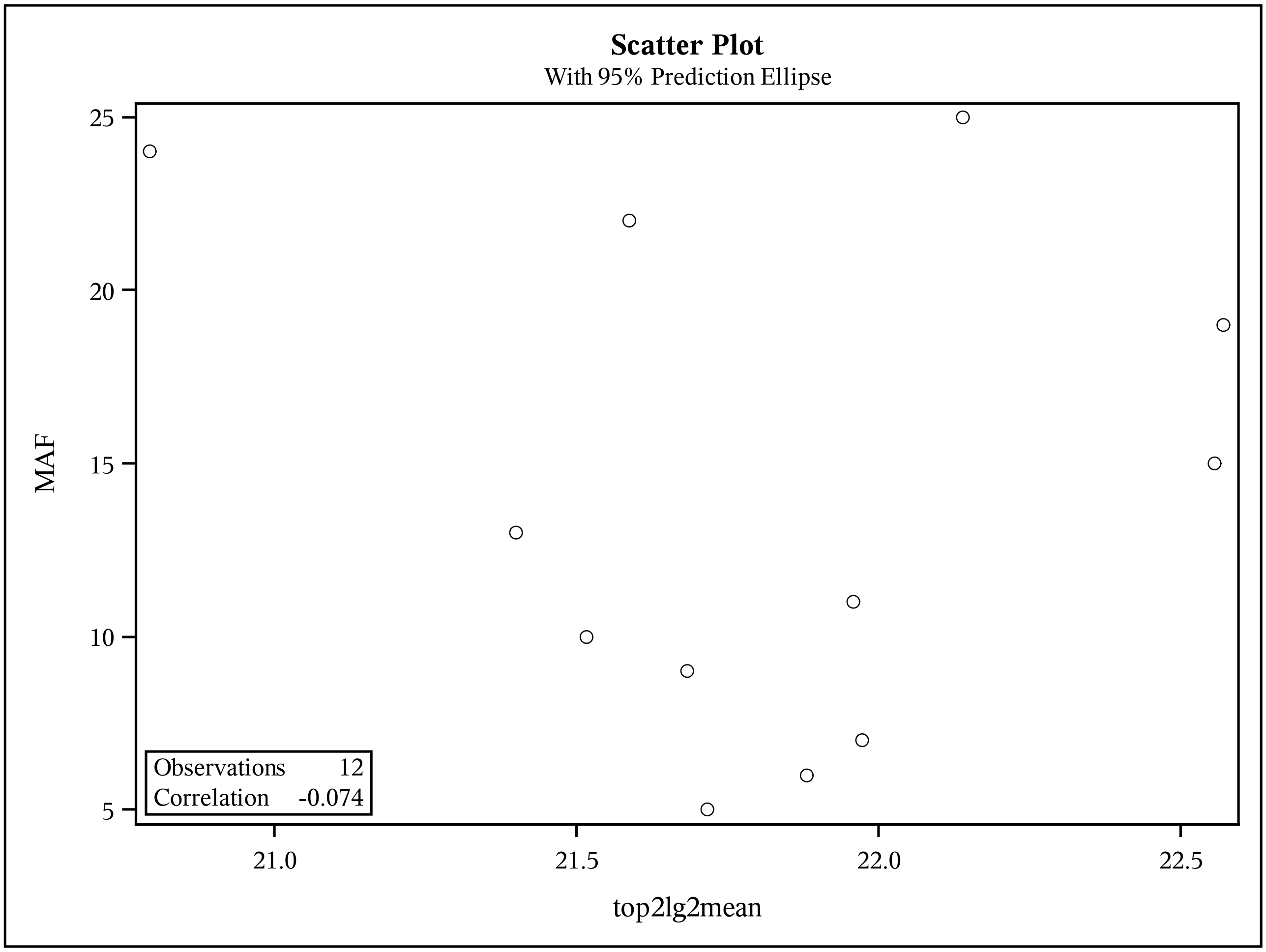

Correlation C_pooltop2lg2mean_tp2 symbol MAF

The CORR Procedure

Gene_Symbol=SIRPA

2 Variables:	top2lg2mean MAF

Simple Statistics						
Variable	N	Mean	Std Dev	Sum	Minimum	Maximum
top2lg2mean	12	18.70502	0.32043	224.46021	18.32002	19.31147
MAF	12	13.83333	7.10740	166.00000	5.00000	25.00000

Pearson Correlation Coefficients, N = 12<br>Prob > |r| under H0: Rho=0		
	top2lg2mean	MAF
top2lg2mean	1.00000	0.38309<br>0.2190
MAF	0.38309<br>0.2190	1.00000

Pearson Correlation Statistics (Fisher's z Transformation)									
Variable	With Variable	N	Sample Correlation	Fisher's z	Bias Adjustment	Correlation Estimate	95% Confidence Limits		p Value for H0:Rho=0
top2lg2mean	MAF	12	0.38309	0.40367	0.01741	0.36813	-0.260887	0.777723	0.2259

Correlation C_pooltop2lg2mean_tp2 symbol MAF

The CORR Procedure

Gene_Symbol=SIRPA

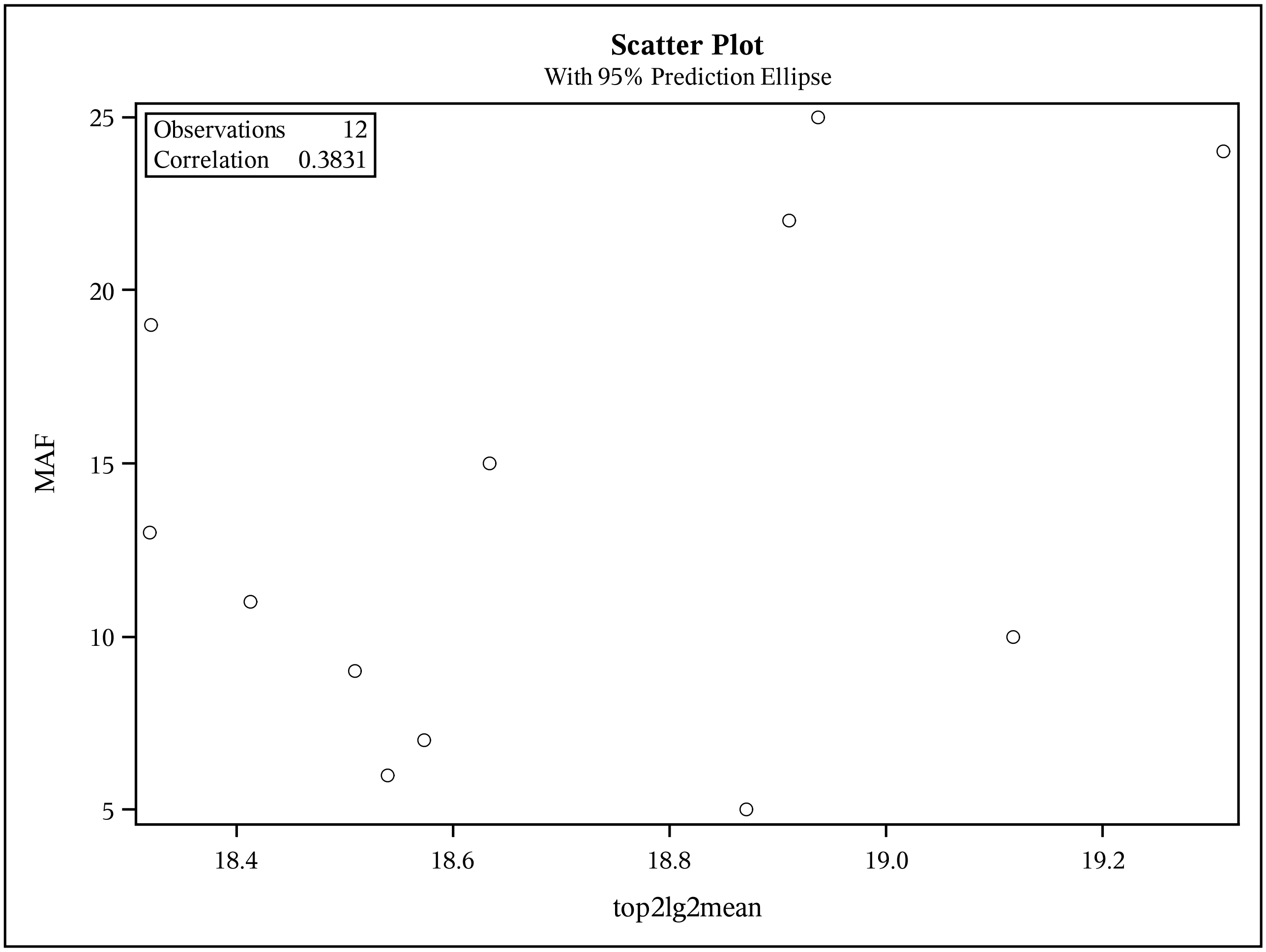

Correlation C_pooltop2lg2mean_tp2 symbol MAF

The CORR Procedure

Gene_Symbol=SOD1

**2 Variables:**	top2lg2mean MAF

Simple Statistics						
**Variable**	**N**	**Mean**	**Std Dev**	**Sum**	**Minimum**	**Maximum**
**top2lg2mean**	12	23.16349	0.19007	277.96185	22.94400	23.52580
**MAF**	12	13.83333	7.10740	166.00000	5.00000	25.00000

Pearson Correlation Coefficients, N = 12<br>Prob > |r| under H0: Rho=0		
	**top2lg2mean**	**MAF**
**top2lg2mean**	1.00000	-0.31532<br>0.3181
**MAF**	-0.31532<br>0.3181	1.00000

Pearson Correlation Statistics (Fisher's z Transformation)									
**Variable**	**With Variable**	**N**	**Sample Correlation**	**Fisher's z**	**Bias Adjustment**	**Correlation Estimate**	**95% Confidence Limits**		**p Value for H0:Rho=0**
**top2lg2mean**	**MAF**	12	-0.31532	-0.32644	-0.01433	-0.30236	-0.746690	0.328558	0.3274

Correlation C_pooltop2lg2mean_tp2 symbol MAF

The CORR Procedure

Gene_Symbol=SOD1

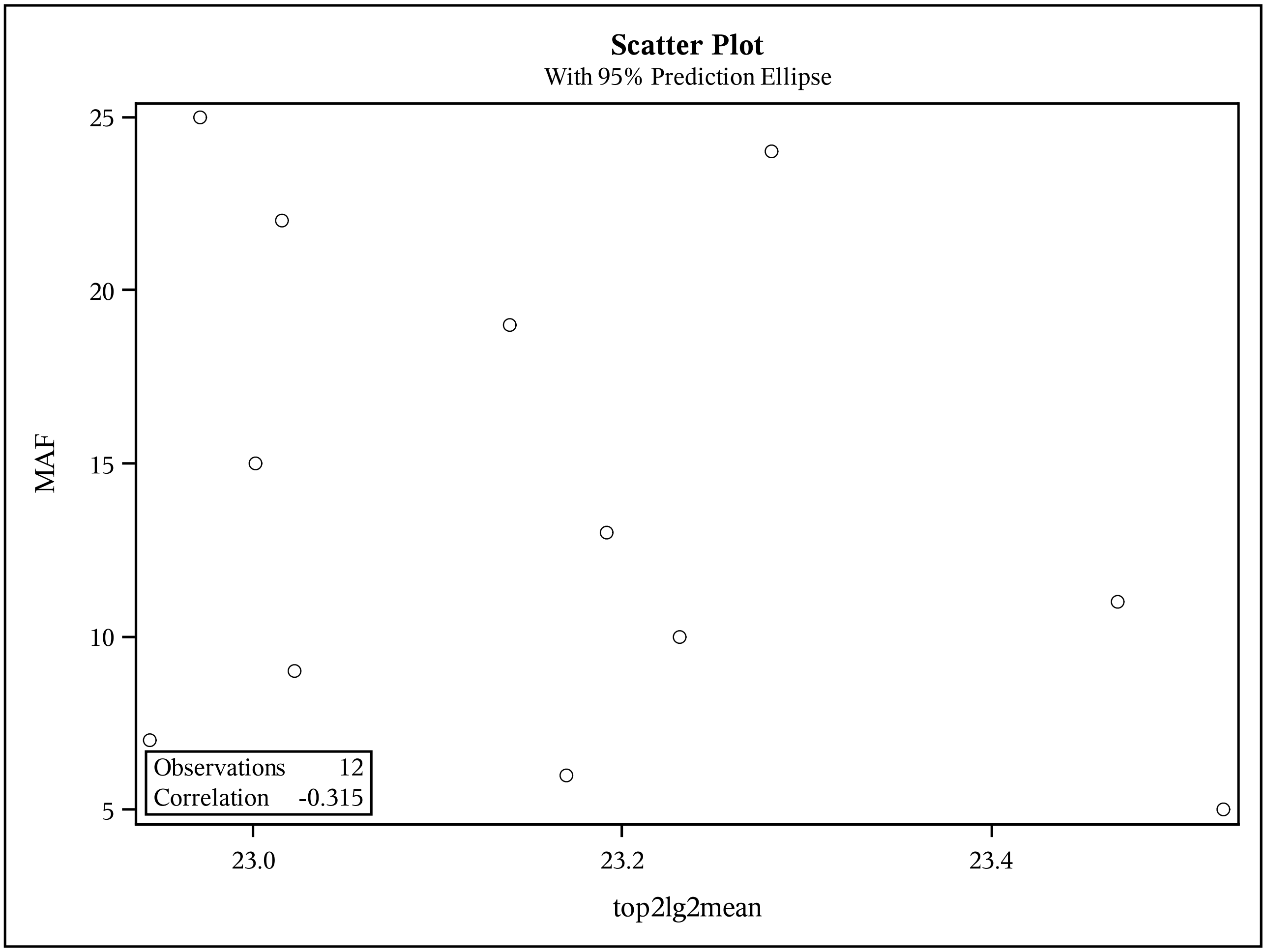

Correlation C_pooltop2lg2mean_tp2 symbol MAF

The CORR Procedure

Gene_Symbol=SOD3

2 Variables:	top2lg2mean MAF

Simple Statistics						
Variable	N	Mean	Std Dev	Sum	Minimum	Maximum
top2lg2mean	12	20.89297	0.47158	250.71558	19.97754	21.33323
MAF	12	13.83333	7.10740	166.00000	5.00000	25.00000

Pearson Correlation Coefficients, N = 12<br>Prob > |r| under H0: Rho=0		
	top2lg2mean	MAF
top2lg2mean	1.00000	-0.32876<br>0.2968
MAF	-0.32876<br>0.2968	1.00000

Pearson Correlation Statistics (Fisher's z Transformation)									
Variable	With Variable	N	Sample Correlation	Fisher's z	Bias Adjustment	Correlation Estimate	95% Confidence Limits		p Value for H0:Rho=0
top2lg2mean	MAF	12	-0.32876	-0.34144	-0.01494	-0.31537	-0.752987	0.315665	0.3057

Correlation C_pooltop2lg2mean_tp2 symbol MAF

The CORR Procedure

Gene_Symbol=SOD3

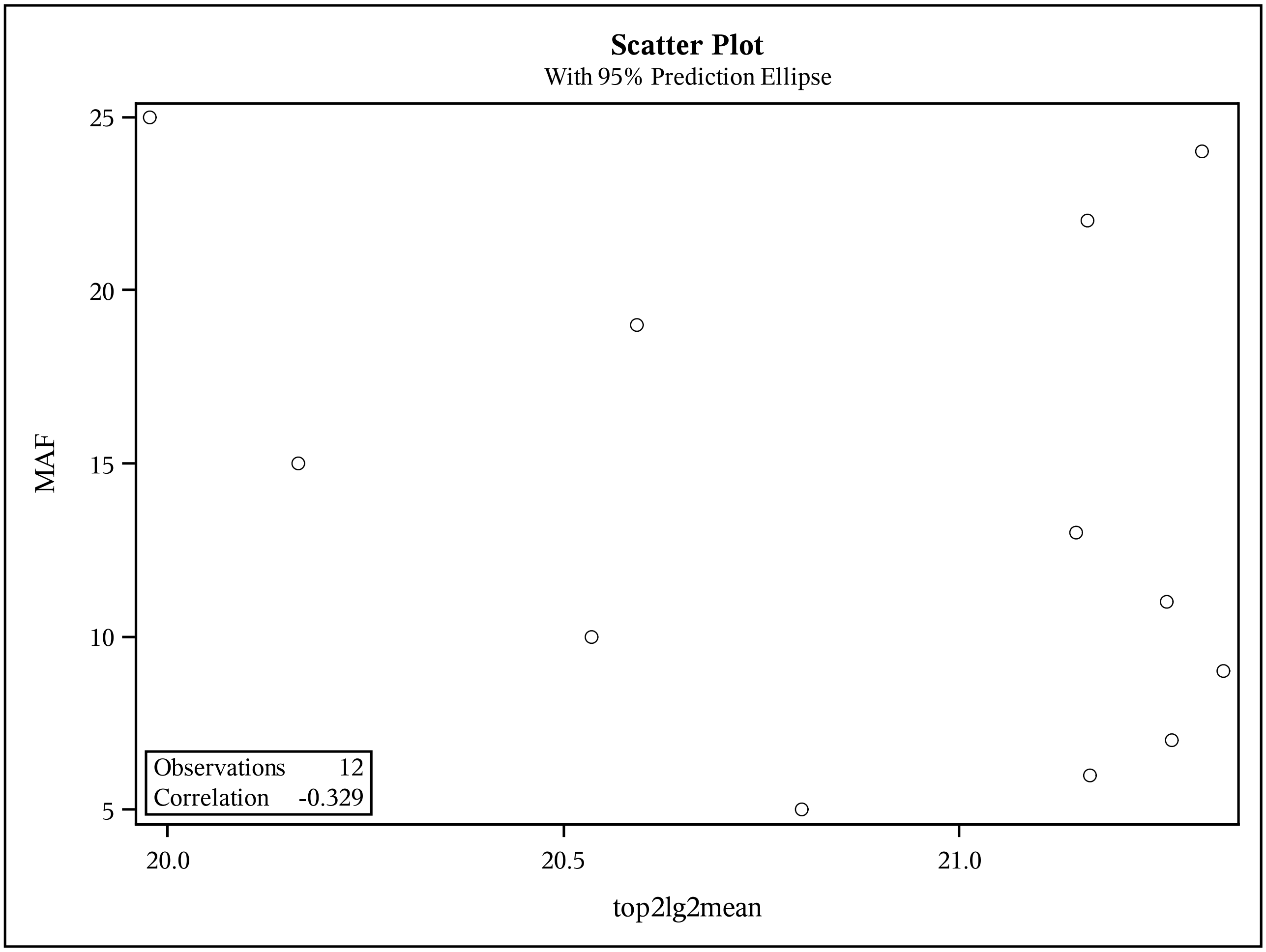

Correlation C_pooltop2lg2mean_tp2 symbol MAF

The CORR Procedure

Gene_Symbol=SPARCL1

**2 Variables:**	top2lg2mean MAF

Simple Statistics						
**Variable**	**N**	**Mean**	**Std Dev**	**Sum**	**Minimum**	**Maximum**
**top2lg2mean**	12	21.56743	0.29155	258.80916	20.82870	22.02751
**MAF**	12	13.83333	7.10740	166.00000	5.00000	25.00000

Pearson Correlation Coefficients, N = 12<br>Prob > |r| under H0: Rho=0		
	**top2lg2mean**	**MAF**
**top2lg2mean**	1.00000	0.47057<br>0.1226
**MAF**	0.47057<br>0.1226	1.00000

Pearson Correlation Statistics (Fisher's z Transformation)									
**Variable**	**With Variable**	**N**	**Sample Correlation**	**Fisher's z**	**Bias Adjustment**	**Correlation Estimate**	**95% Confidence Limits**		**p Value for H0:Rho=0**
**top2lg2mean**	**MAF**	12	0.47057	0.51080	0.02139	0.45375	-0.162458	0.815332	0.1254

Correlation C_pooltop2lg2mean_tp2 symbol MAF

The CORR Procedure

Gene_Symbol=SPARCL1

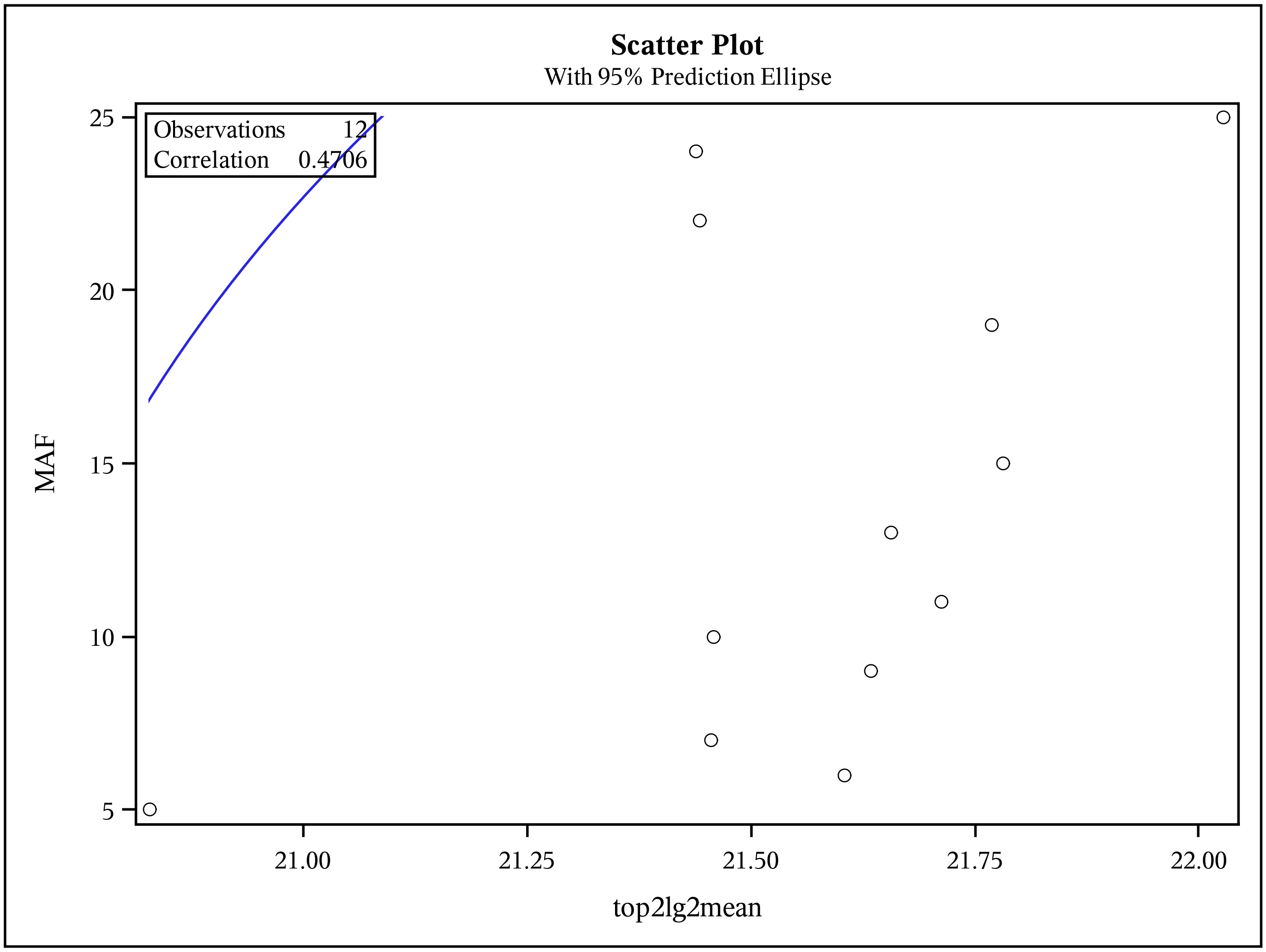

Correlation C_pooltop2lg2mean_tp2 symbol MAF

The CORR Procedure

Gene_Symbol=SPP1

**2 Variables:**	top2lg2mean MAF

Simple Statistics						
**Variable**	**N**	**Mean**	**Std Dev**	**Sum**	**Minimum**	**Maximum**
**top2lg2mean**	12	21.53026	0.31804	258.36306	20.94234	21.95977
**MAF**	12	13.83333	7.10740	166.00000	5.00000	25.00000

Pearson Correlation Coefficients, N = 12<br>Prob > |r| under H0: Rho=0		
	**top2lg2mean**	**MAF**
**top2lg2mean**	1.00000	0.62353<br>0.0303
**MAF**	0.62353<br>0.0303	1.00000

Pearson Correlation Statistics (Fisher's z Transformation)									
**Variable**	**With Variable**	**N**	**Sample Correlation**	**Fisher's z**	**Bias Adjustment**	**Correlation Estimate**	**95% Confidence Limits**		**p Value for H0:Rho=0**
**top2lg2mean**	**MAF**	12	0.62353	0.73076	0.02834	0.60590	0.049061	0.875402	0.0284

Correlation C_pooltop2lg2mean_tp2 symbol MAF

The CORR Procedure

Gene_Symbol=SPP1

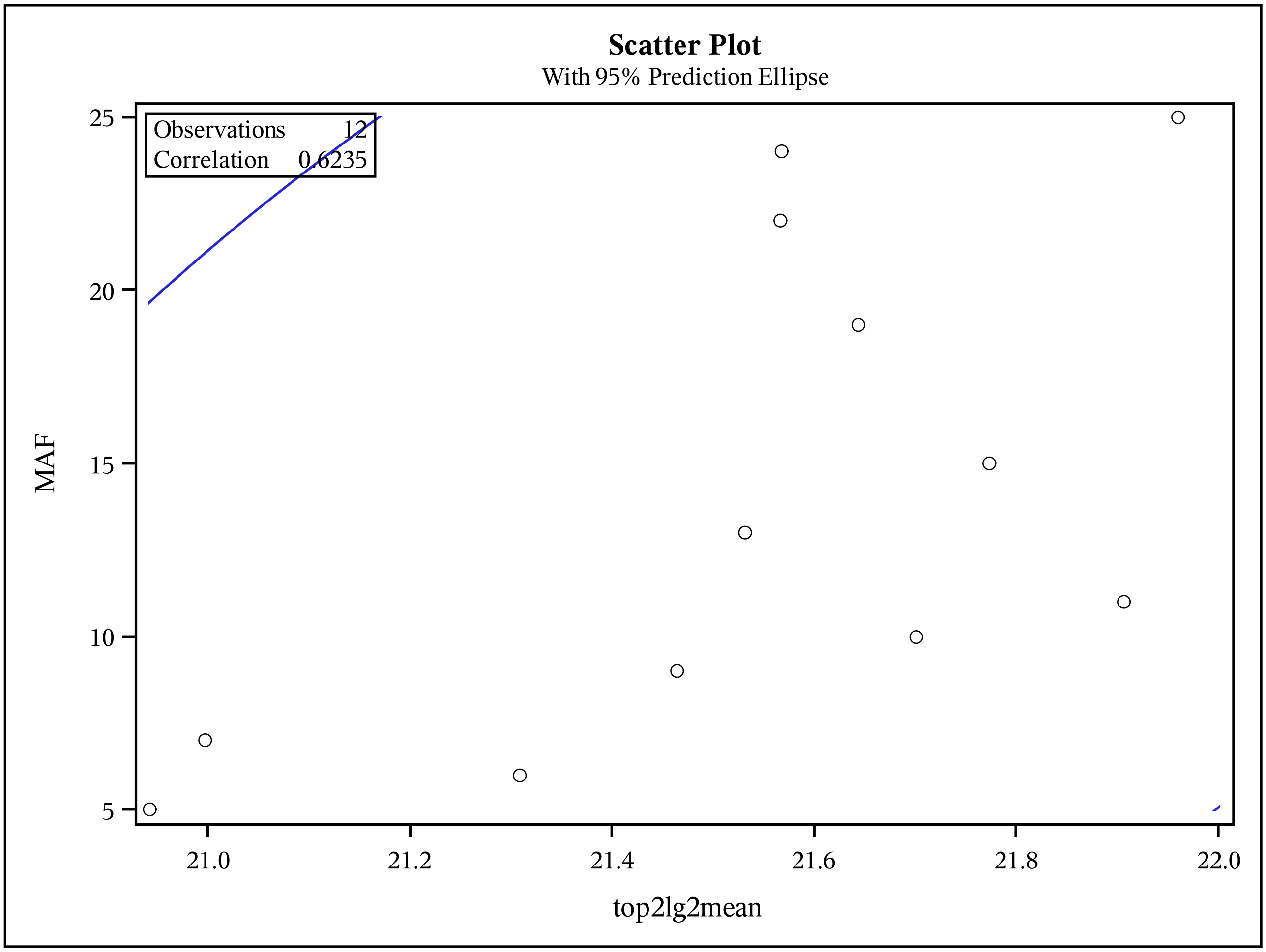

Correlation C_pooltop2lg2mean_tp2 symbol MAF

The CORR Procedure

Gene_Symbol=THY1

**2 Variables:**	top2lg2mean MAF

Simple Statistics						
**Variable**	**N**	**Mean**	**Std Dev**	**Sum**	**Minimum**	**Maximum**
**top2lg2mean**	12	21.42731	0.30687	257.12772	20.79614	21.87430
**MAF**	12	13.83333	7.10740	166.00000	5.00000	25.00000

Pearson Correlation Coefficients, N = 12<br>Prob > |r| under H0: Rho=0		
	**top2lg2mean**	**MAF**
**top2lg2mean**	1.00000	0.37242<br>0.2332
**MAF**	0.37242<br>0.2332	1.00000

Pearson Correlation Statistics (Fisher's z Transformation)									
**Variable**	**With Variable**	**N**	**Sample Correlation**	**Fisher's z**	**Bias Adjustment**	**Correlation Estimate**	**95% Confidence Limits**		**p Value for H0:Rho=0**
**top2lg2mean**	**MAF**	12	0.37242	0.39122	0.01693	0.35774	-0.272002	0.772951	0.2405

Correlation C_pooltop2lg2mean_tp2 symbol MAF

The CORR Procedure

Gene_Symbol=THY1

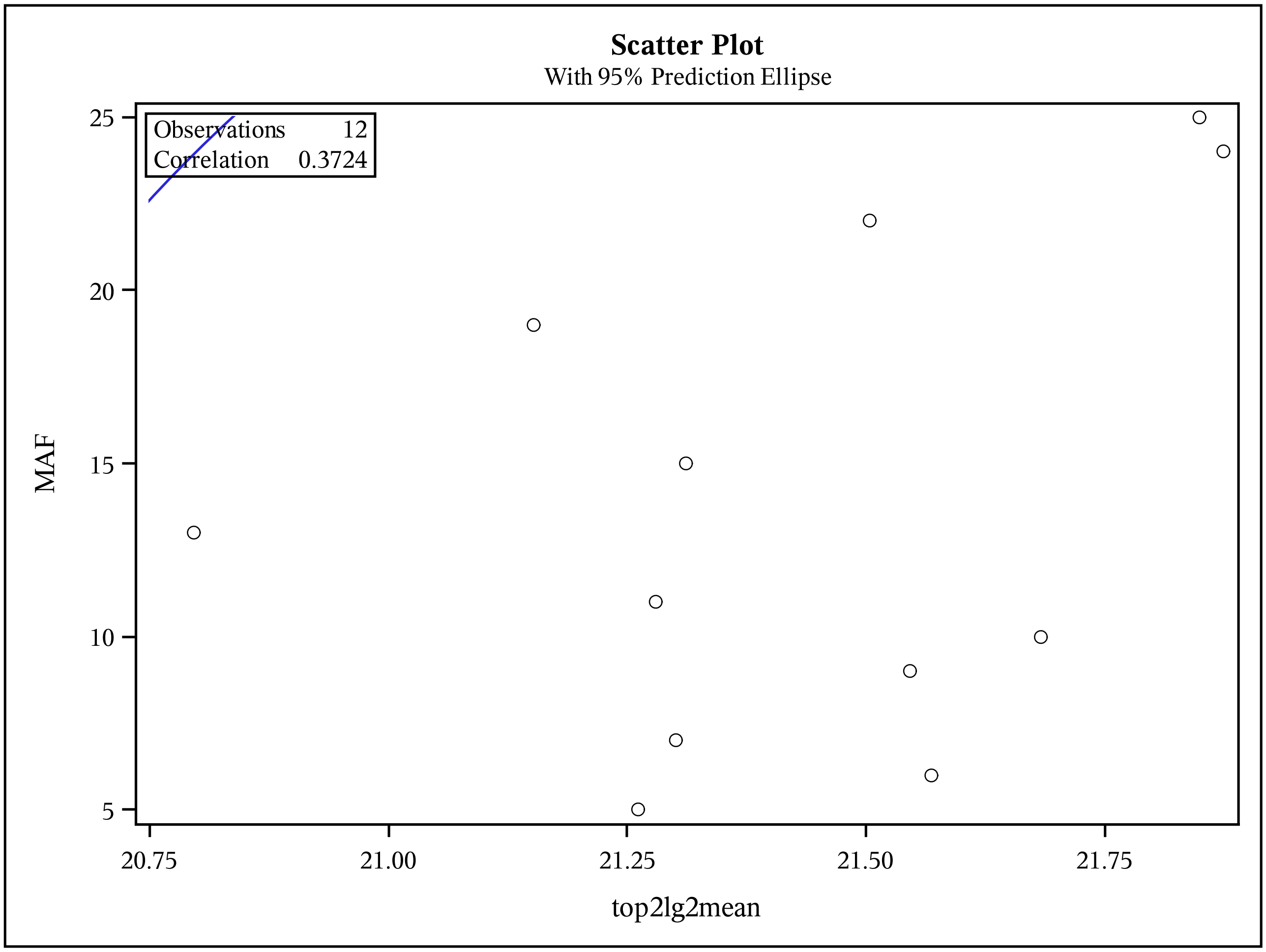

Correlation C_pooltop2lg2mean_tp2 symbol MAF

The CORR Procedure

Gene_Symbol=TTR

**2 Variables:**	top2lg2mean MAF

Simple Statistics						
**Variable**	**N**	**Mean**	**Std Dev**	**Sum**	**Minimum**	**Maximum**
**top2lg2mean**	12	27.71611	0.13881	332.59337	27.42233	27.91455
**MAF**	12	13.83333	7.10740	166.00000	5.00000	25.00000

Pearson Correlation Coefficients, N = 12<br>Prob > |r| under H0: Rho=0		
	**top2lg2mean**	**MAF**
**top2lg2mean**	1.00000	0.14512<br>0.6527
**MAF**	0.14512<br>0.6527	1.00000

Pearson Correlation Statistics (Fisher's z Transformation)									
**Variable**	**With Variable**	**N**	**Sample Correlation**	**Fisher's z**	**Bias Adjustment**	**Correlation Estimate**	**95% Confidence Limits**		**p Value for H0:Rho=0**
**top2lg2mean**	**MAF**	12	0.14512	0.14615	0.00660	0.13866	-0.472874	0.660036	0.6611

Correlation C_pooltop2lg2mean_tp2 symbol MAF

The CORR Procedure

Gene_Symbol=TTR

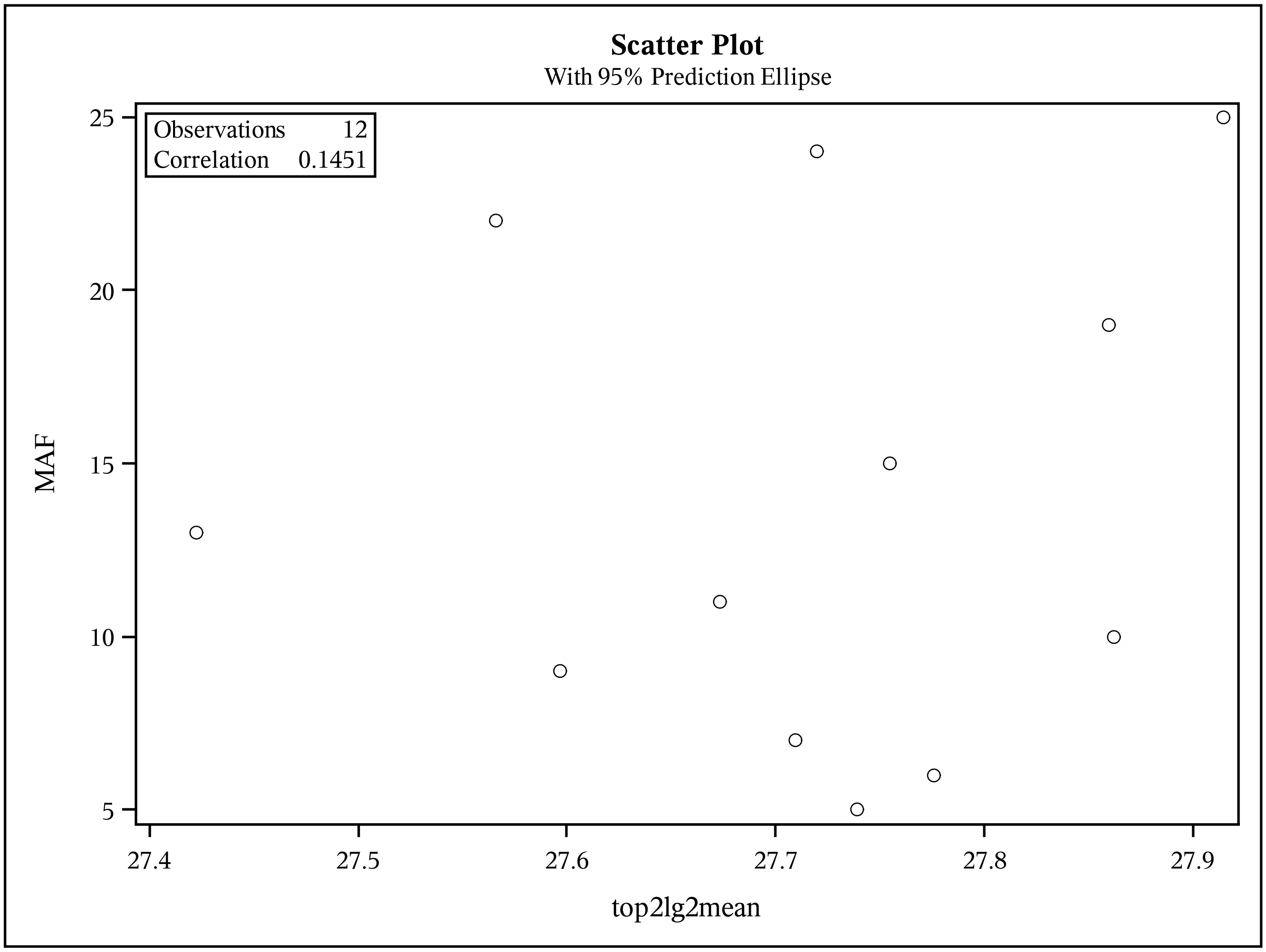

Correlation C_pooltop2lg2mean_tp2 symbol MAF

The CORR Procedure

Gene_Symbol=UBA52

**2 Variables:**	top2lg2mean MAF

Simple Statistics						
**Variable**	**N**	**Mean**	**Std Dev**	**Sum**	**Minimum**	**Maximum**
**top2lg2mean**	12	20.04327	0.66303	240.51925	18.84271	21.12546
**MAF**	12	13.83333	7.10740	166.00000	5.00000	25.00000

Pearson Correlation Coefficients, N = 12<br>Prob > |r| under H0: Rho=0		
	**top2lg2mean**	**MAF**
**top2lg2mean**	1.00000	0.66990<br>0.0172
**MAF**	0.66990<br>0.0172	1.00000

Pearson Correlation Statistics (Fisher's z Transformation)									
**Variable**	**With Variable**	**N**	**Sample Correlation**	**Fisher's z**	**Bias Adjustment**	**Correlation Estimate**	**95% Confidence Limits**		**p Value for H0:Rho=0**
**top2lg2mean**	**MAF**	12	0.66990	0.81056	0.03045	0.65277	0.126116	0.892368	0.0150

Correlation C_pooltop2lg2mean_tp2 symbol MAF

The CORR Procedure

Gene_Symbol=UBA52

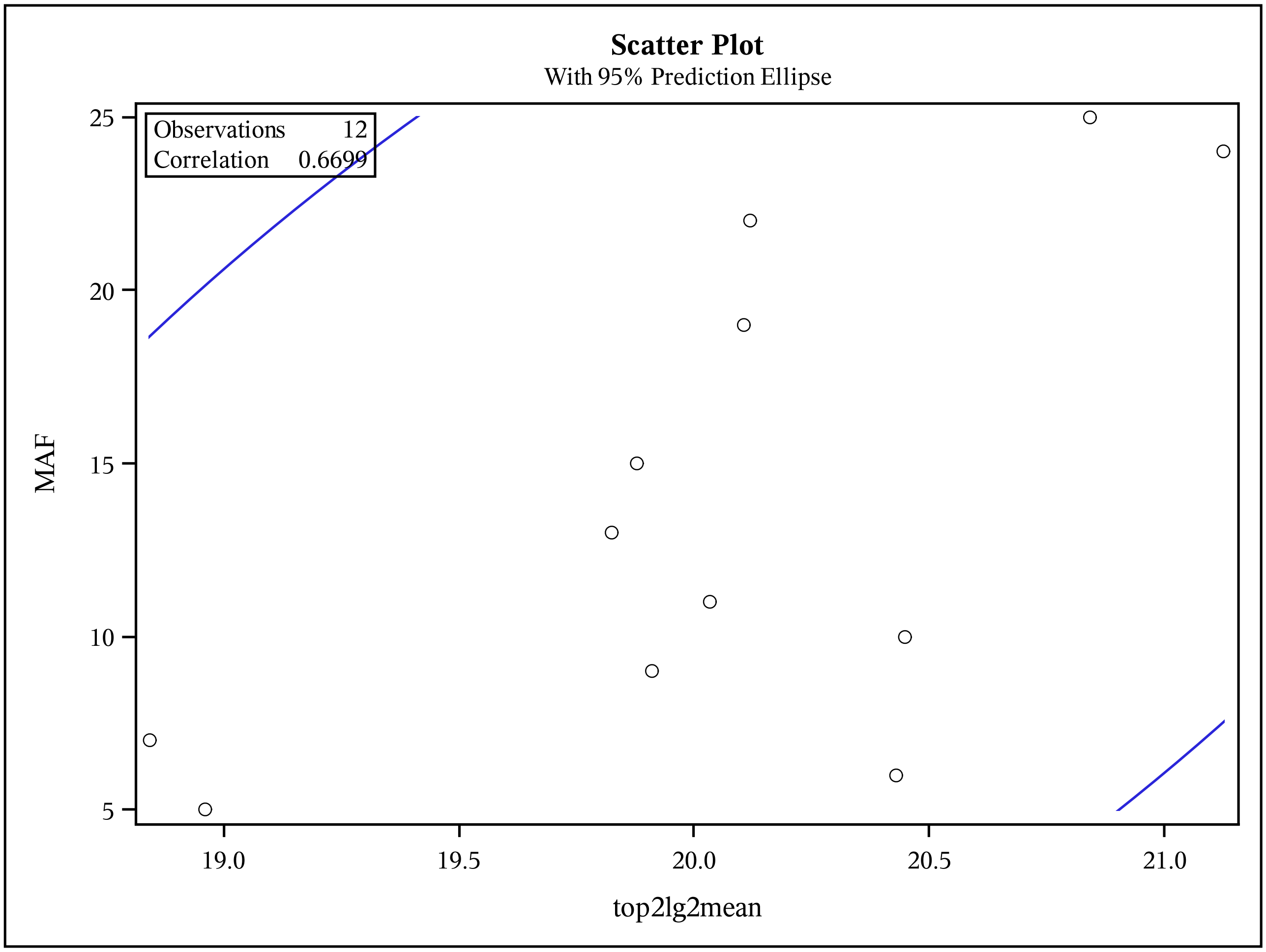

Correlation C_pooltop2lg2mean_tp2 symbol MAF

The CORR Procedure

Gene_Symbol=VGF

**2 Variables:**	top2lg2mean MAF

Simple Statistics						
**Variable**	**N**	**Mean**	**Std Dev**	**Sum**	**Minimum**	**Maximum**
**top2lg2mean**	12	17.78075	0.64762	213.36900	16.63359	18.78800
**MAF**	12	13.83333	7.10740	166.00000	5.00000	25.00000

Pearson Correlation Coefficients, N = 12<br>Prob > |r| under H0: Rho=0		
	**top2lg2mean**	**MAF**
**top2lg2mean**	1.00000	-0.45136<br>0.1408
**MAF**	-0.45136<br>0.1408	1.00000

Pearson Correlation Statistics (Fisher's z Transformation)									
**Variable**	**With Variable**	**N**	**Sample Correlation**	**Fisher's z**	**Bias Adjustment**	**Correlation Estimate**	**95% Confidence Limits**		**p Value for H0:Rho=0**
**top2lg2mean**	**MAF**	12	-0.45136	-0.48641	-0.02052	-0.43488	-0.807297	0.185261	0.1445

Correlation C_pooltop2lg2mean_tp2 symbol MAF

The CORR Procedure

Gene_Symbol=VGF

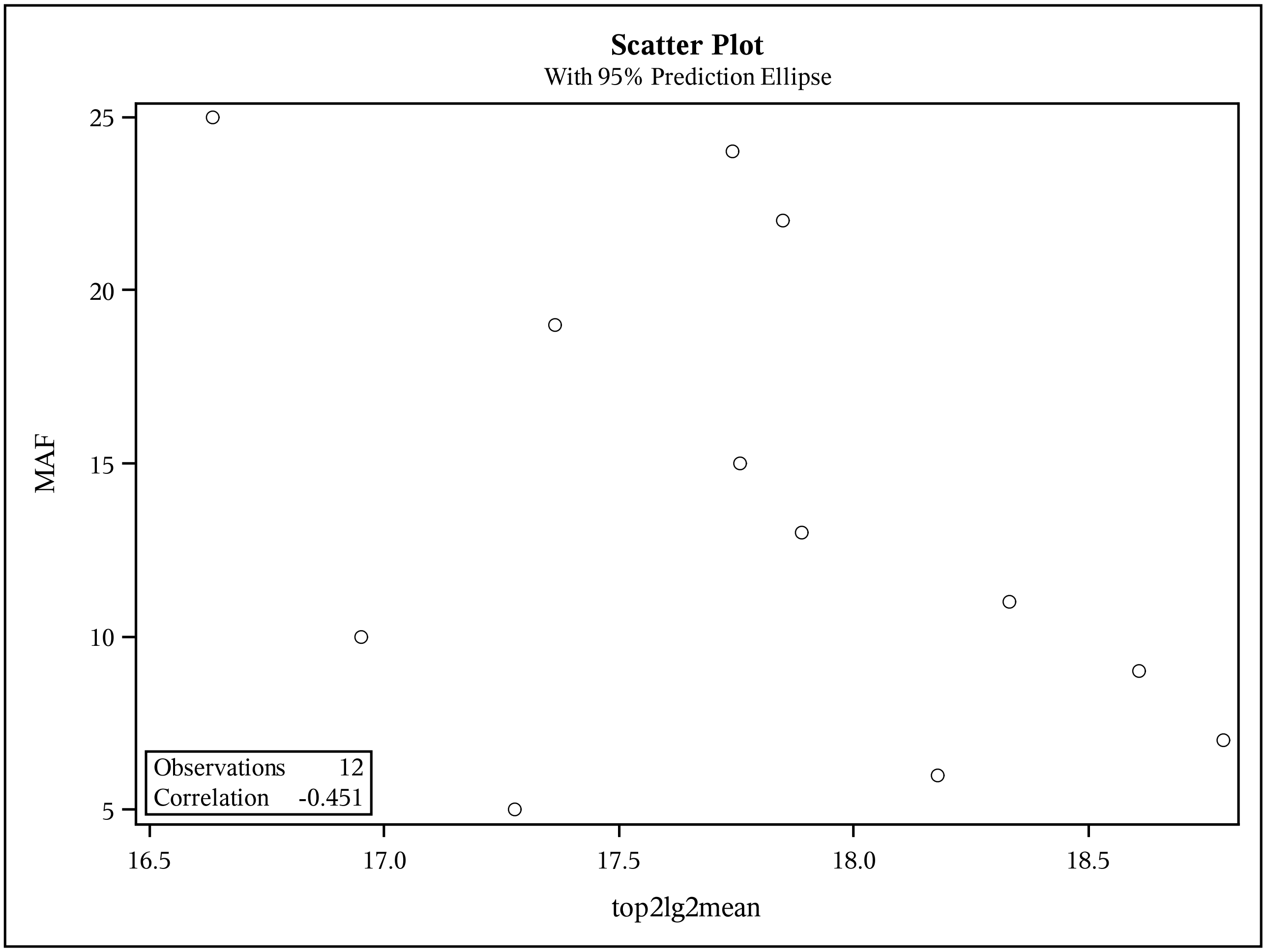

Correlation C_pooltop2lg2mean_tp2 symbol MAF

The CORR Procedure

Gene_Symbol=VSTM2A

**2 Variables:**	top2lg2mean MAF

Simple Statistics						
**Variable**	**N**	**Mean**	**Std Dev**	**Sum**	**Minimum**	**Maximum**
**top2lg2mean**	12	18.95094	0.20903	227.41134	18.48949	19.22659
**MAF**	12	13.83333	7.10740	166.00000	5.00000	25.00000

Pearson Correlation Coefficients, N = 12<br>Prob > |r| under H0: Rho=0		
	**top2lg2mean**	**MAF**
**top2lg2mean**	1.00000	0.50398<br>0.0948
**MAF**	0.50398<br>0.0948	1.00000

Pearson Correlation Statistics (Fisher's z Transformation)									
**Variable**	**With Variable**	**N**	**Sample Correlation**	**Fisher's z**	**Bias Adjustment**	**Correlation Estimate**	**95% Confidence Limits**		**p Value for H0:Rho=0**
**top2lg2mean**	**MAF**	12	0.50398	0.55463	0.02291	0.48670	-0.121003	0.829036	0.0961

Correlation C_pooltop2lg2mean_tp2 symbol MAF

The CORR Procedure

Gene_Symbol=VSTM2A

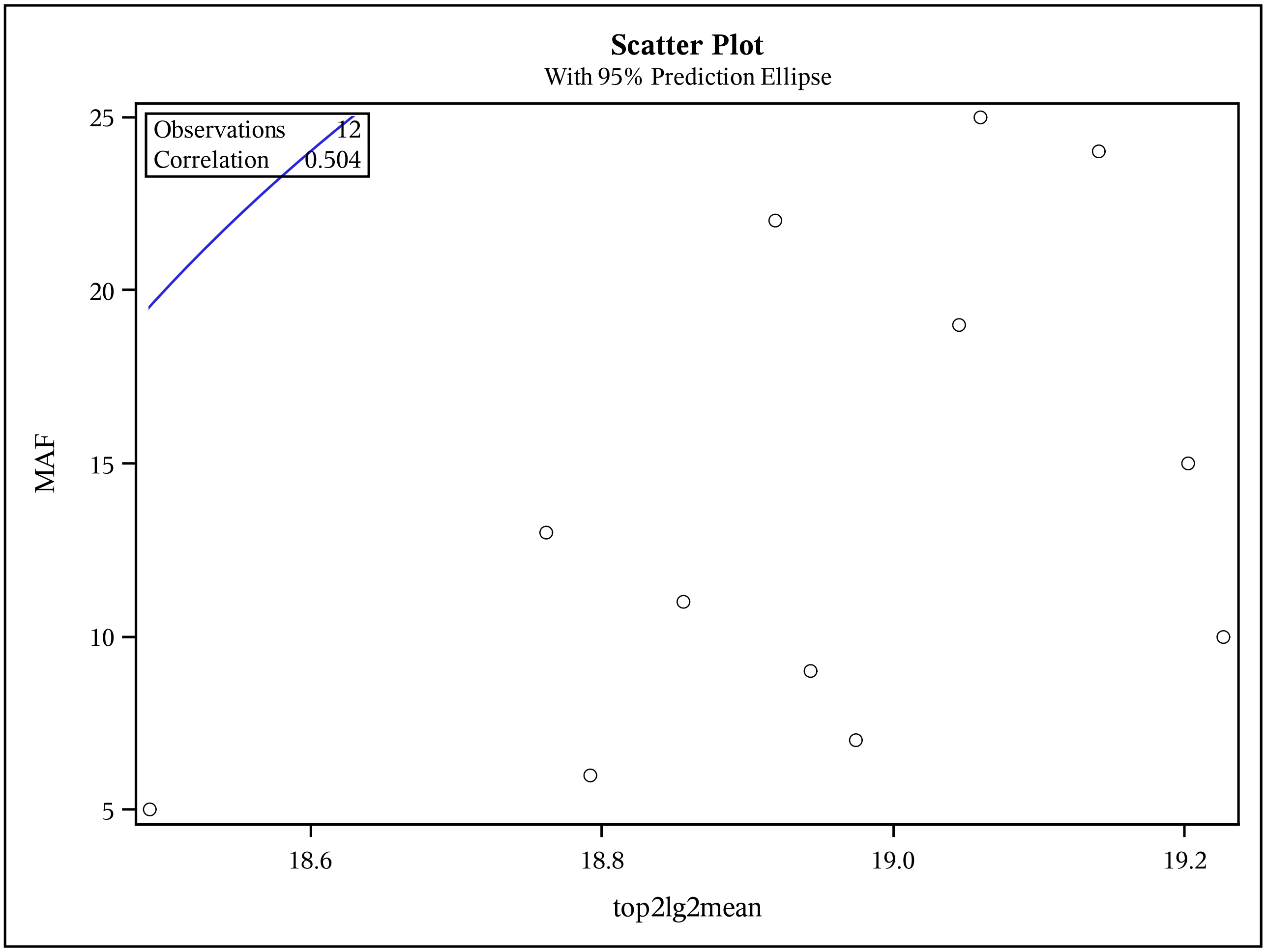

Correlation C_pooltop2lg2mean_tp2 symbol MAF

The CORR Procedure

Gene_Symbol=VTN

**2 Variables:**	top2lg2mean MAF

Simple Statistics						
**Variable**	**N**	**Mean**	**Std Dev**	**Sum**	**Minimum**	**Maximum**
**top2lg2mean**	12	16.88153	0.66936	202.57832	16.23902	18.58806
**MAF**	12	13.83333	7.10740	166.00000	5.00000	25.00000

Pearson Correlation Coefficients, N = 12<br>Prob > |r| under H0: Rho=0		
	**top2lg2mean**	**MAF**
**top2lg2mean**	1.00000	-0.41375<br>0.1812
**MAF**	-0.41375<br>0.1812	1.00000

Pearson Correlation Statistics (Fisher's z Transformation)										
**Variable**	**With Variable**	**N**	**Sample Correlation**	**Fisher's z**	**Bias Adjustment**	**Correlation Estimate**	**95% Confidence Limits**		**p Value for H0:Rho=0**
**top2lg2mean**	**MAF**	12	-0.41375	-0.44013	-0.01881	-0.39804	-0.791204	0.227924	0.1867

Correlation C_pooltop2lg2mean_tp2 symbol MAF

The CORR Procedure

Gene_Symbol=VTN

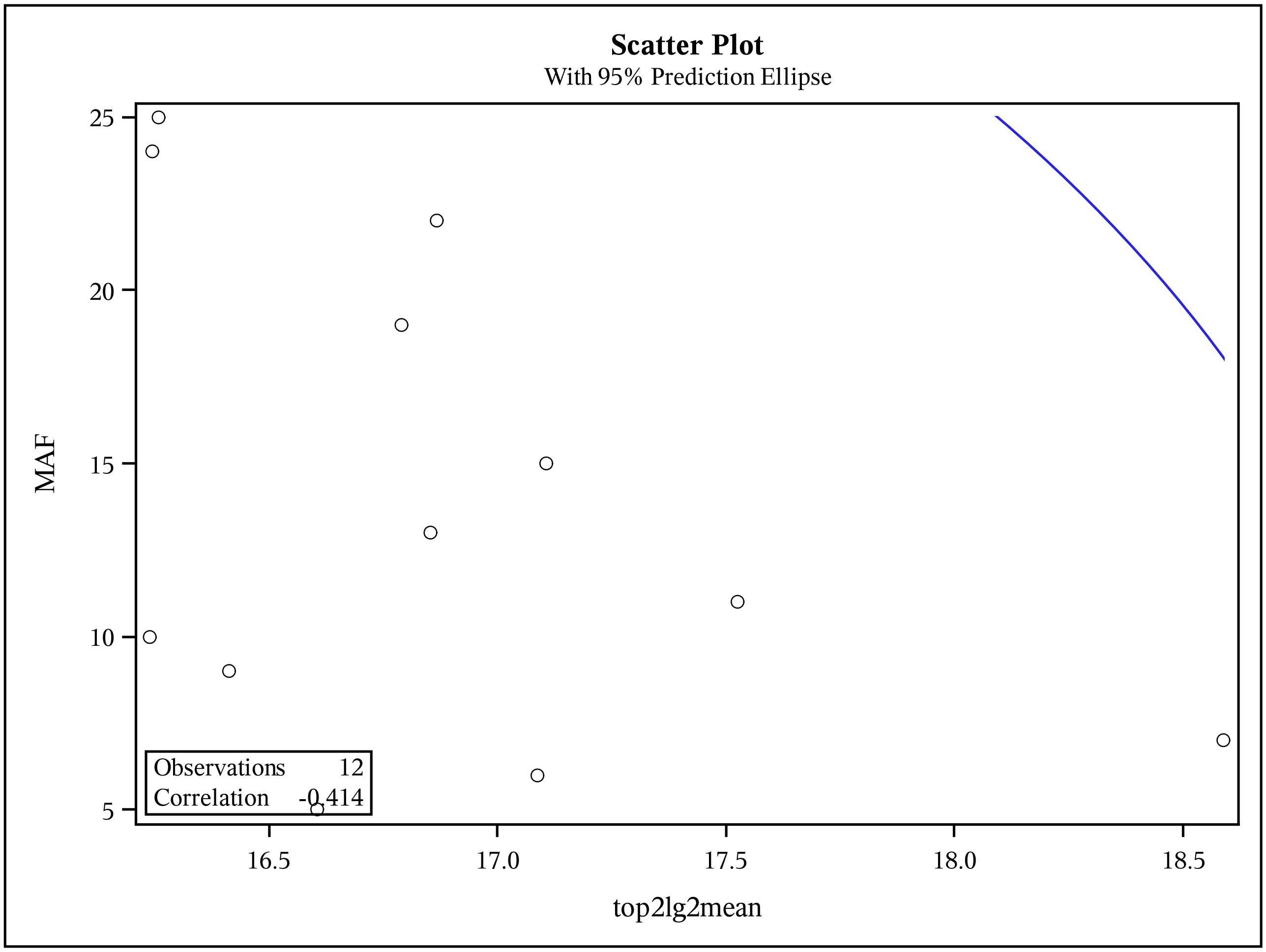

Supplement: Figure S12 — Influence of multi-affinity fractionation (MAF) run order on protein abundance measurements. For each of 81 proteins (organized alphabetically by gene symbol of origin), log2 transformed abundance values (calculated from the mean of the two most abundant peptides) for each of the pooled sample replicates are plotted versus MAF run order. Pearson correlation coefficients and statistics (Fisher's z transformation) are listed for each protein. (PDF) [file pone.0064314.s012.pdf]
