# Supplementary material for: Quantitative Label-Free Proteomics for Discovery of Biomarkers in Cerebrospinal Fluid: Assessment of Technical and Inter-Individual Variation
Source: PLoS One. 2013 May 20;8(5):e64314. doi: 10.1371/journal.pone.0064314 (PMC3659127; doi:10.1371/journal.pone.0064314)

Correlation C_pooltop2lg2mean_tp2 symbol LCMS

The CORR Procedure

Gene_Symbol=A1BG

2 Variables:	top2lg2mean LCMS

Simple Statistics						
Variable	N	Mean	Std Dev	Sum	Minimum	Maximum
top2lg2mean	12	22.57461	0.11608	270.89531	22.29520	22.78045
LCMS	12	11.75000	8.70867	141.00000	1.00000	24.00000

Pearson Correlation Coefficients, N = 12<br>Prob > |r| under H0: Rho=0		
	top2lg2mean	LCMS
top2lg2mean	1.00000	0.44971<br>0.1424
LCMS	0.44971<br>0.1424	1.00000

Pearson Correlation Statistics (Fisher's z Transformation)									
Variable	With Variable	N	Sample Correlation	Fisher's z	Bias Adjustment	Correlation Estimate	95% Confidence Limits		p Value for H0:Rho=0
top2lg2mean	LCMS	12	0.44971	0.48433	0.02044	0.43325	-0.187195	0.806598	0.1462

Correlation C_pooltop2lg2mean_tp2 symbol LCMS

The CORR Procedure

Gene_Symbol=A1BG

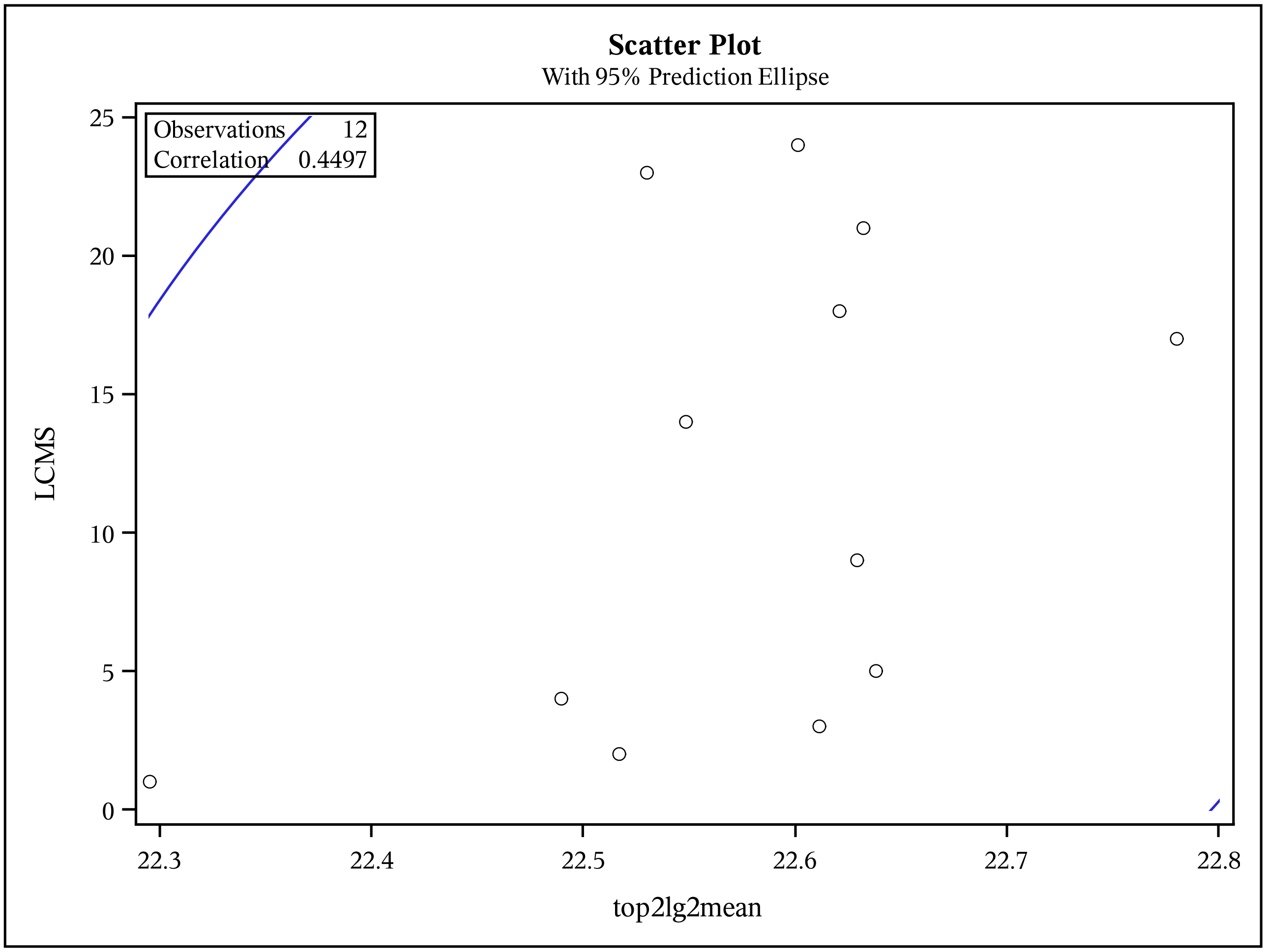

Correlation C_pooltop2lg2mean_tp2 symbol LCMS

The CORR Procedure

Gene_Symbol=A2M

2 Variables:	top2lg2mean LCMS

Simple Statistics						
Variable	N	Mean	Std Dev	Sum	Minimum	Maximum
top2lg2mean	12	23.51360	0.37790	282.16326	22.83730	24.15903
LCMS	12	11.75000	8.70867	141.00000	1.00000	24.00000

Pearson Correlation Coefficients, N = 12<br>Prob > |r| under H0: Rho=0		
	top2lg2mean	LCMS
top2lg2mean	1.00000	-0.21330<br>0.5056
LCMS	-0.21330<br>0.5056	1.00000

Pearson Correlation Statistics (Fisher's z Transformation)									
Variable	With Variable	N	Sample Correlation	Fisher's z	Bias Adjustment	Correlation Estimate	95% Confidence Limits		p Value for H0:Rho=0
top2lg2mean	LCMS	12	-0.21330	-0.21663	-0.00970	-0.20403	-0.696390	0.418924	0.5158

Correlation C_pooltop2lg2mean_tp2 symbol LCMS

The CORR Procedure

Gene_Symbol=A2M

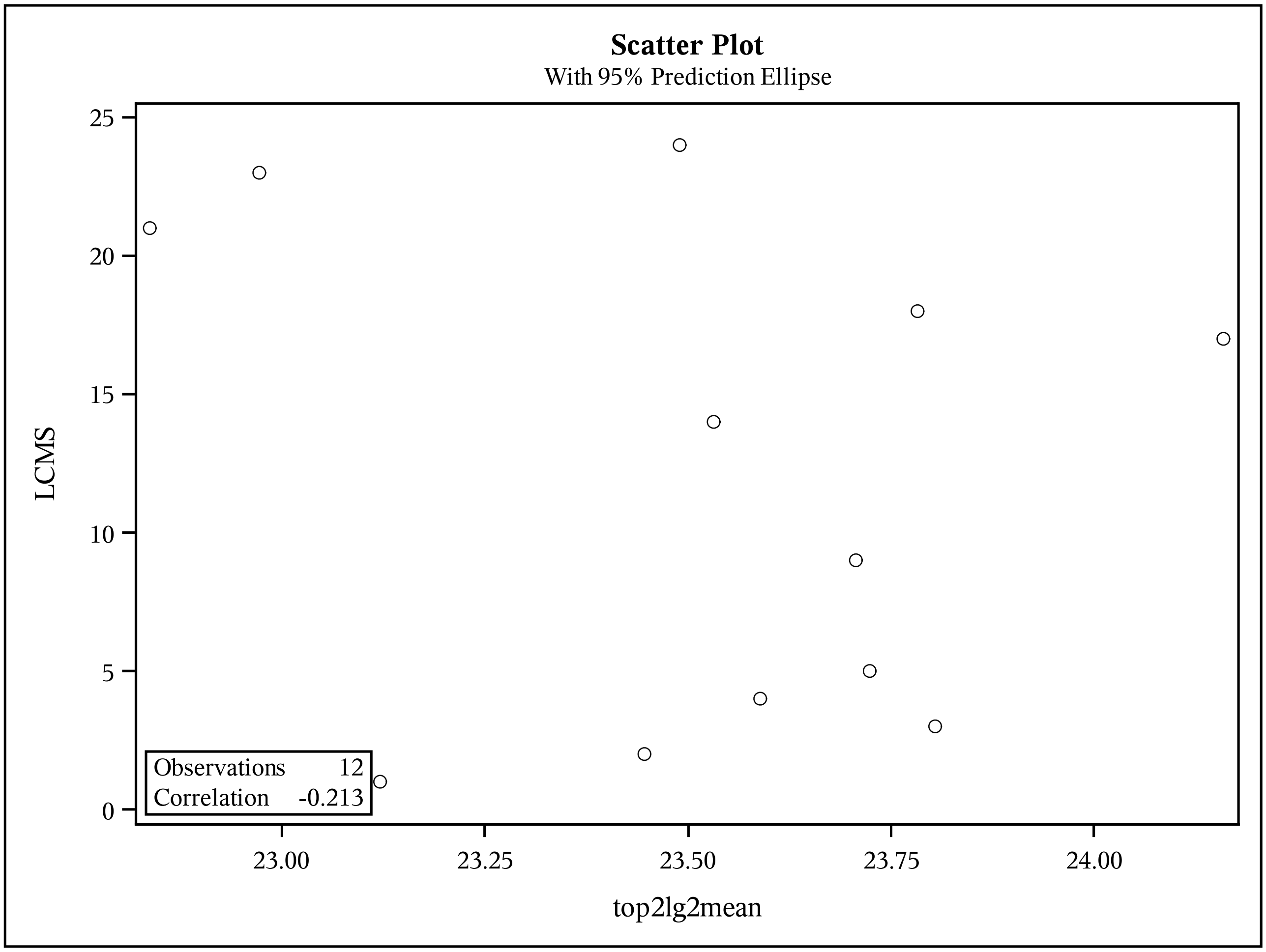

Correlation C_pooltop2lg2mean_tp2 symbol LCMS

The CORR Procedure

Gene_Symbol=AGT

2 Variables:	top2lg2mean LCMS

Simple Statistics						
**Variable**	**N**	**Mean**	**Std Dev**	**Sum**	**Minimum**	**Maximum**
**top2lg2mean**	12	24.82819	0.43733	297.93831	23.95626	25.72056
**LCMS**	12	11.75000	8.70867	141.00000	1.00000	24.00000

Pearson Correlation Coefficients, N = 12<br>Prob > |r| under H0: Rho=0		
	**top2lg2mean**	**LCMS**
**top2lg2mean**	1.00000	-0.26110<br>0.4124
**LCMS**	-0.26110<br>0.4124	1.00000

Pearson Correlation Statistics (Fisher's z Transformation)									
**Variable**	**With Variable**	**N**	**Sample Correlation**	**Fisher's z**	**Bias Adjustment**	**Correlation Estimate**	**95% Confidence Limits**		**p Value for H0:Rho=0**
**top2lg2mean**	**LCMS**	12	-0.26110	-0.26729	-0.01187	-0.25001	-0.720529	0.378149	0.4226

Correlation C_pooltop2lg2mean_tp2 symbol LCMS

The CORR Procedure

Gene_Symbol=AGT

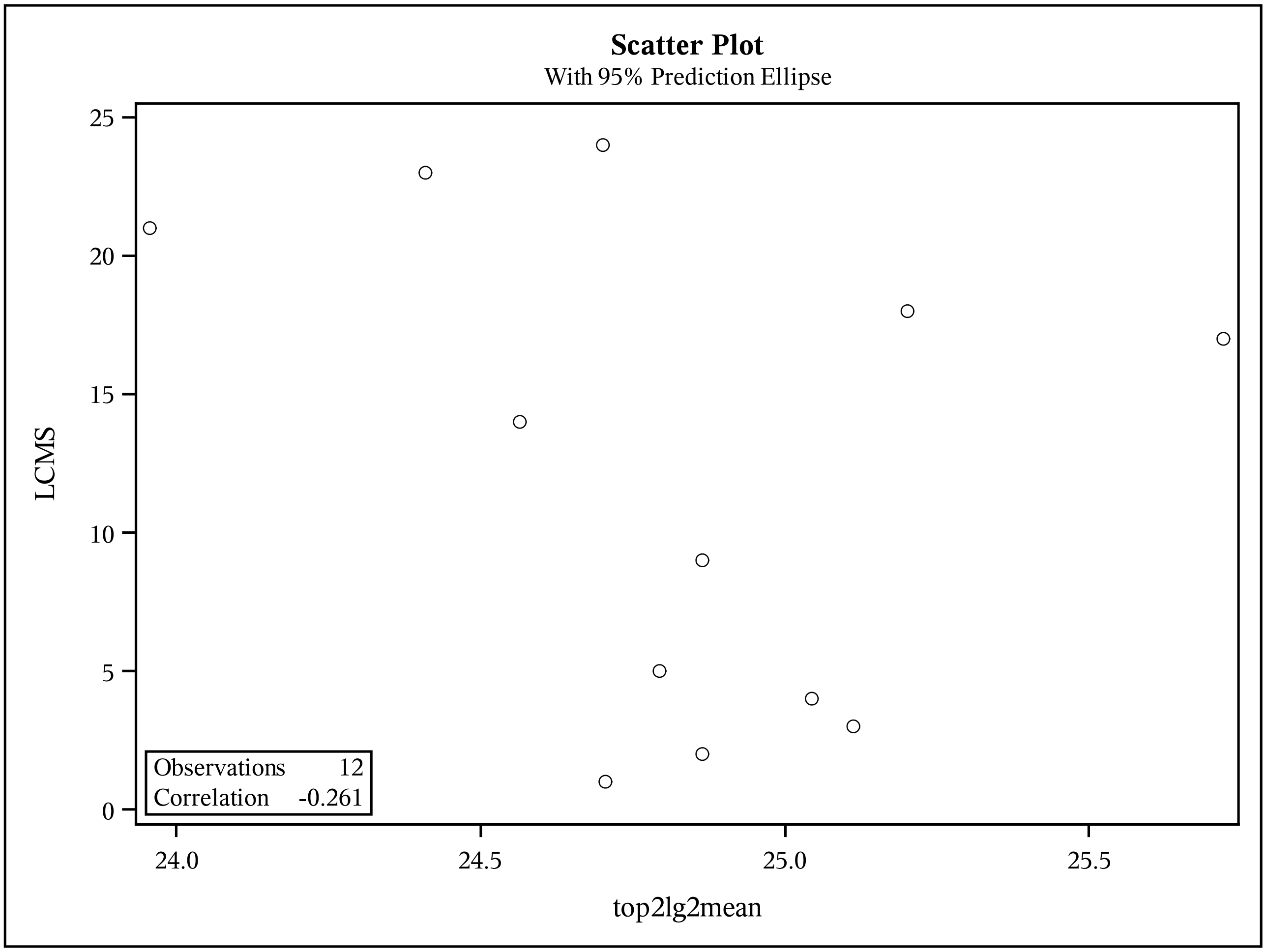

Correlation C_pooltop2lg2mean_tp2 symbol LCMS

The CORR Procedure

Gene_Symbol=AHSG

2 Variables:	top2lg2mean LCMS

Simple Statistics						
Variable	N	Mean	Std Dev	Sum	Minimum	Maximum
top2lg2mean	12	22.54882	0.14816	270.58587	22.32566	22.82825
LCMS	12	11.75000	8.70867	141.00000	1.00000	24.00000

Pearson Correlation Coefficients, N = 12<br>Prob > |r| under H0: Rho=0		
	top2lg2mean	LCMS
top2lg2mean	1.00000	0.55293<br>0.0622
LCMS	0.55293<br>0.0622	1.00000

Pearson Correlation Statistics (Fisher's z Transformation)									
Variable	With Variable	N	Sample Correlation	Fisher's z	Bias Adjustment	Correlation Estimate	95% Confidence Limits		p Value for H0:Rho=0
top2lg2mean	LCMS	12	0.55293	0.62260	0.02513	0.53524	-0.055800	0.848504	0.0618

Correlation C_pooltop2lg2mean_tp2 symbol LCMS

The CORR Procedure

Gene_Symbol=AHSG

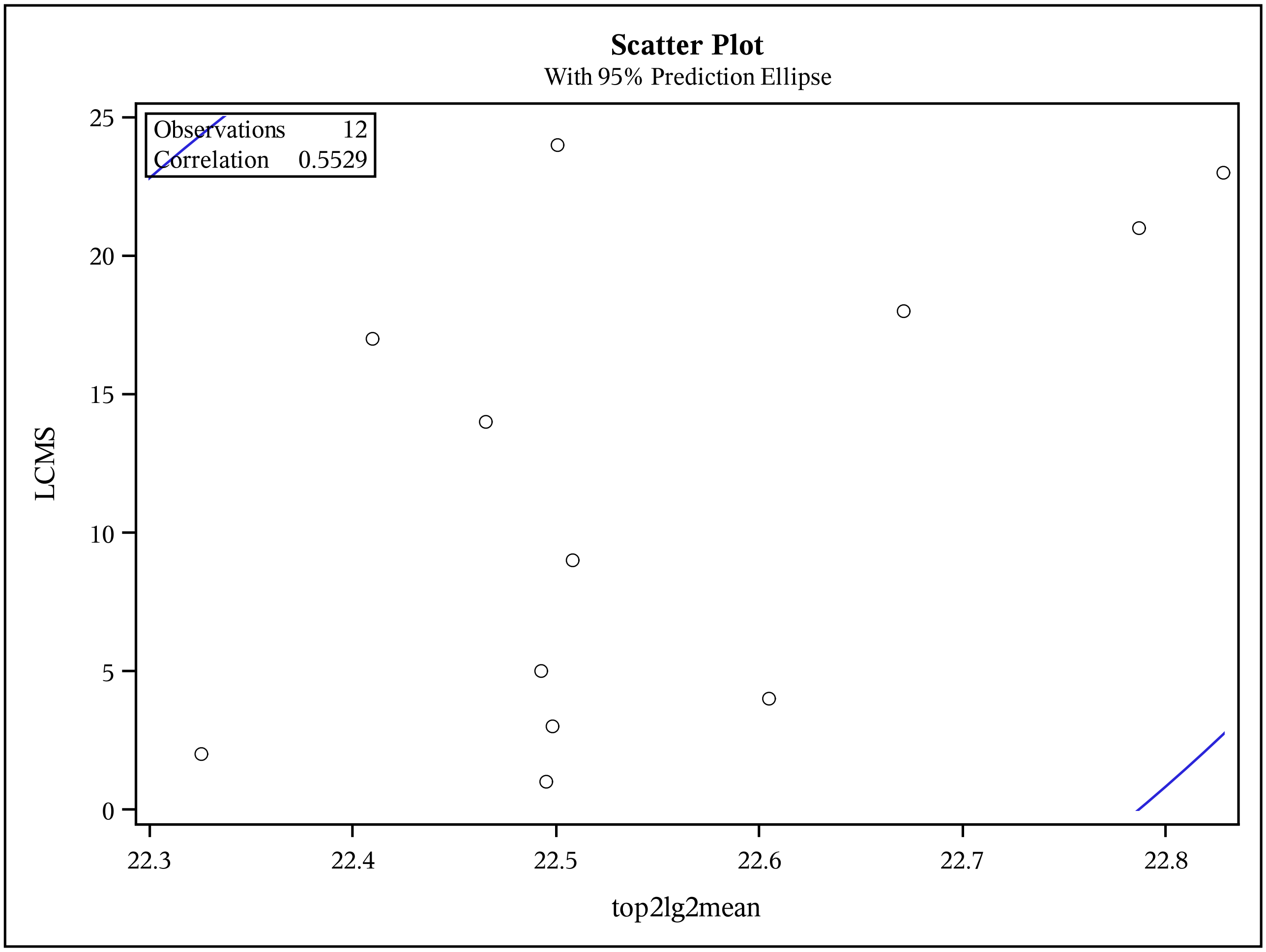

Correlation C_pooltop2lg2mean_tp2 symbol LCMS

The CORR Procedure

Gene_Symbol=APLP1

2 Variables:	top2lg2mean LCMS

Simple Statistics						
**Variable**	**N**	**Mean**	**Std Dev**	**Sum**	**Minimum**	**Maximum**
**top2lg2mean**	12	23.16222	0.29325	277.94665	22.65640	23.64982
**LCMS**	12	11.75000	8.70867	141.00000	1.00000	24.00000

Pearson Correlation Coefficients, N = 12<br>Prob > |r| under H0: Rho=0		
	**top2lg2mean**	**LCMS**
**top2lg2mean**	1.00000	0.30084<br>0.3420
**LCMS**	0.30084<br>0.3420	1.00000

Pearson Correlation Statistics (Fisher's z Transformation)									
**Variable**	**With Variable**	**N**	**Sample Correlation**	**Fisher's z**	**Bias Adjustment**	**Correlation Estimate**	**95% Confidence Limits**		**p Value for H0:Rho=0**
**top2lg2mean**	**LCMS**	12	0.30084	0.31045	0.01367	0.28836	-0.342171	0.739825	0.3517

Correlation C_pooltop2lg2mean_tp2 symbol LCMS

The CORR Procedure

Gene_Symbol=APLP1

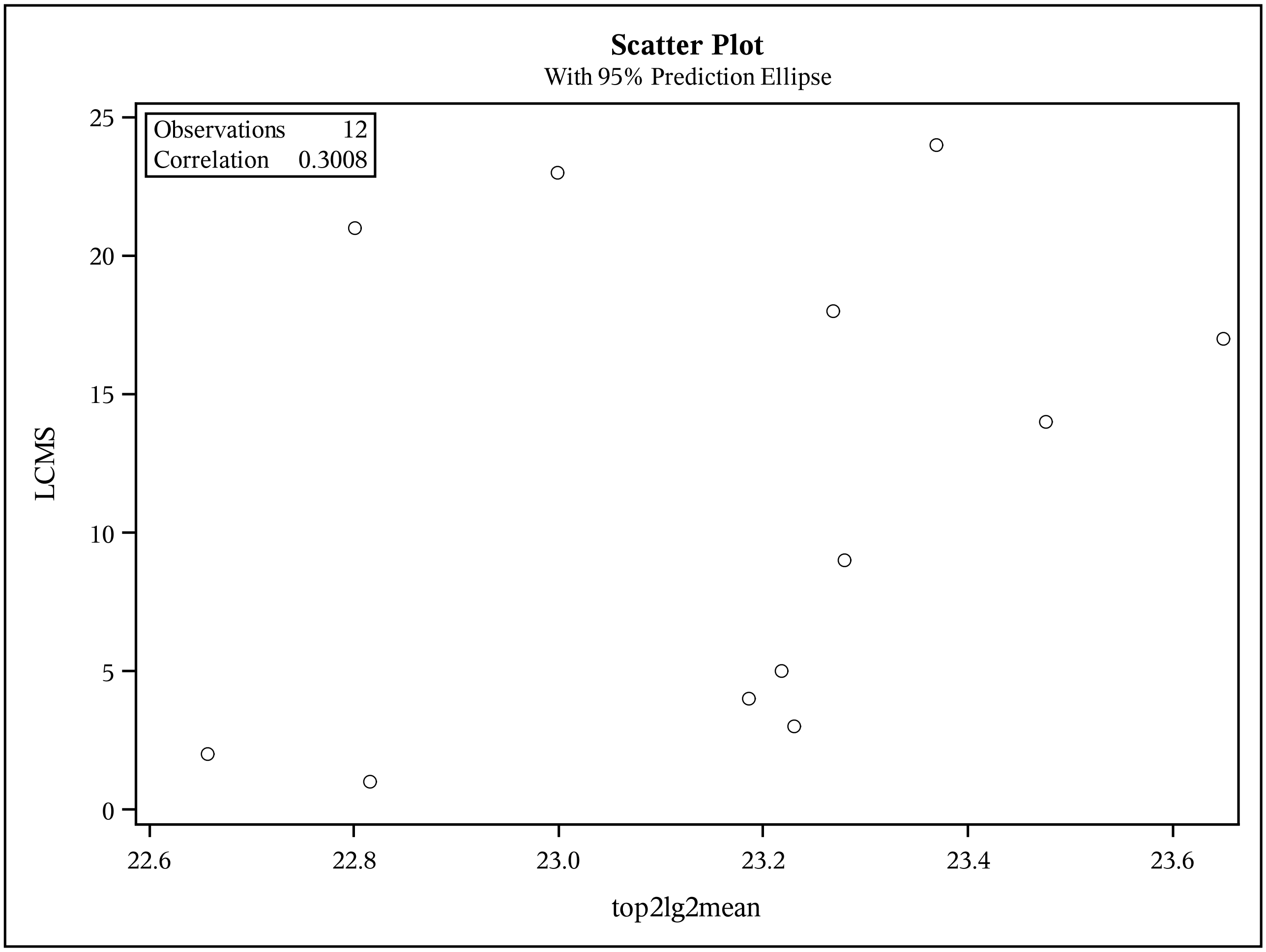

Correlation C_pooltop2lg2mean_tp2 symbol LCMS

The CORR Procedure

Gene_Symbol=APOA1

2 Variables:	top2lg2mean LCMS

Simple Statistics						
Variable	N	Mean	Std Dev	Sum	Minimum	Maximum
top2lg2mean	12	25.58229	0.22491	306.98745	25.28807	25.94263
LCMS	12	11.75000	8.70867	141.00000	1.00000	24.00000

Pearson Correlation Coefficients, N = 12<br>Prob > |r| under H0: Rho=0		
	top2lg2mean	LCMS
top2lg2mean	1.00000	0.83370<br>0.0008
LCMS	0.83370<br>0.0008	1.00000

Pearson Correlation Statistics (Fisher's z Transformation)									
Variable	With Variable	N	Sample Correlation	Fisher's z	Bias Adjustment	Correlation Estimate	95% Confidence Limits		p Value for H0:Rho=0
top2lg2mean	LCMS	12	0.83370	1.20013	0.03790	0.82177	0.469101	0.948394	0.0003

Correlation C_pooltop2lg2mean_tp2 symbol LCMS

The CORR Procedure

Gene_Symbol=APOA1

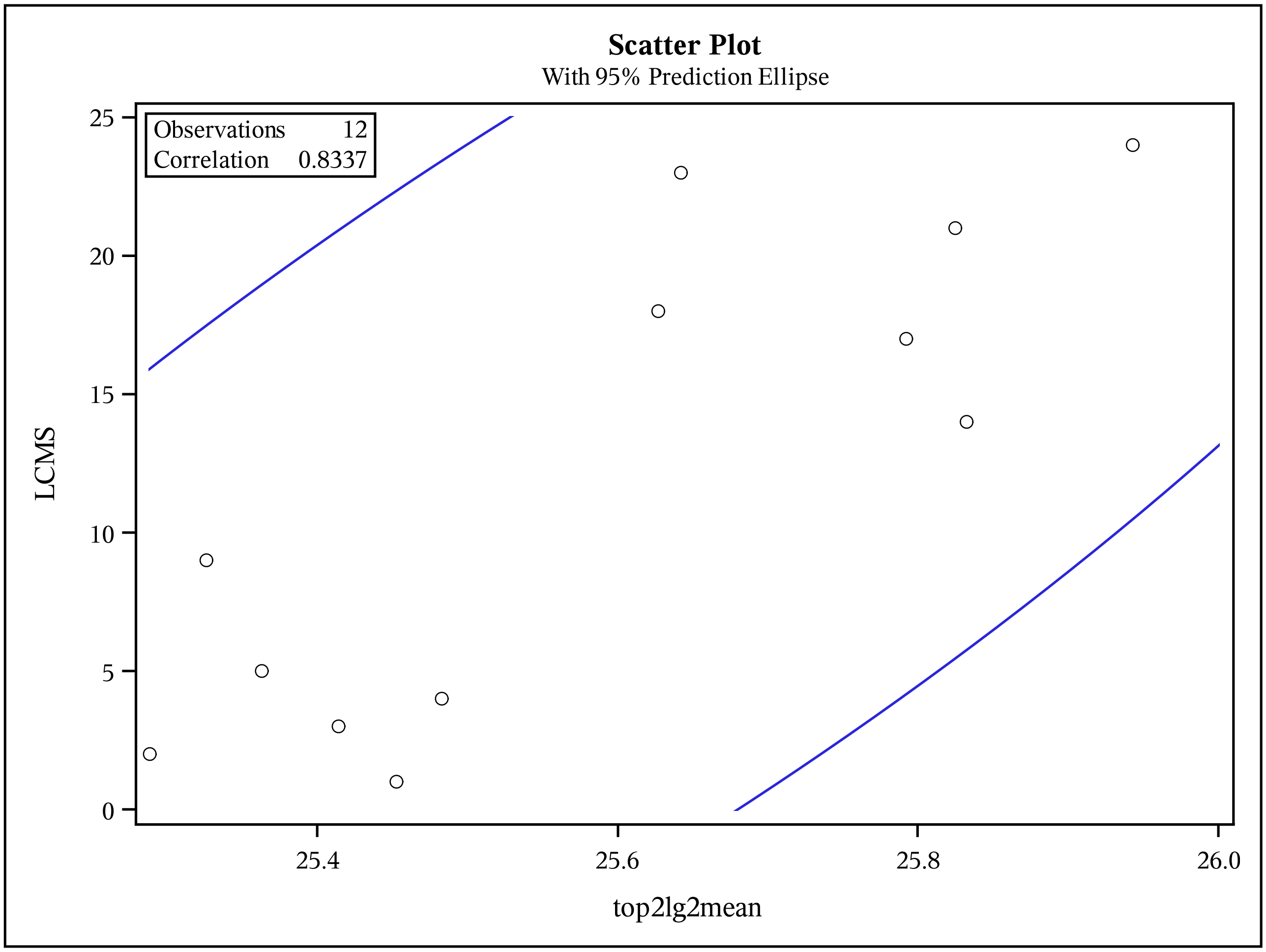

Correlation C_pooltop2lg2mean_tp2 symbol LCMS

The CORR Procedure

Gene_Symbol=APOA4

2 Variables:	top2lg2mean LCMS

Simple Statistics						
Variable	N	Mean	Std Dev	Sum	Minimum	Maximum
top2lg2mean	12	21.32405	0.37275	255.88859	20.64906	21.96869
LCMS	12	11.75000	8.70867	141.00000	1.00000	24.00000

Pearson Correlation Coefficients, N = 12<br>Prob > |r| under H0: Rho=0		
	top2lg2mean	LCMS
top2lg2mean	1.00000	0.51663<br>0.0855
LCMS	0.51663<br>0.0855	1.00000

Pearson Correlation Statistics (Fisher's z Transformation)									
Variable	With Variable	N	Sample Correlation	Fisher's z	Bias Adjustment	Correlation Estimate	95% Confidence Limits		p Value for H0:Rho=0
top2lg2mean	LCMS	12	0.51663	0.57174	0.02348	0.49921	-0.104683	0.834134	0.0863

Correlation C_pooltop2lg2mean_tp2 symbol LCMS

The CORR Procedure

Gene_Symbol=APOA4

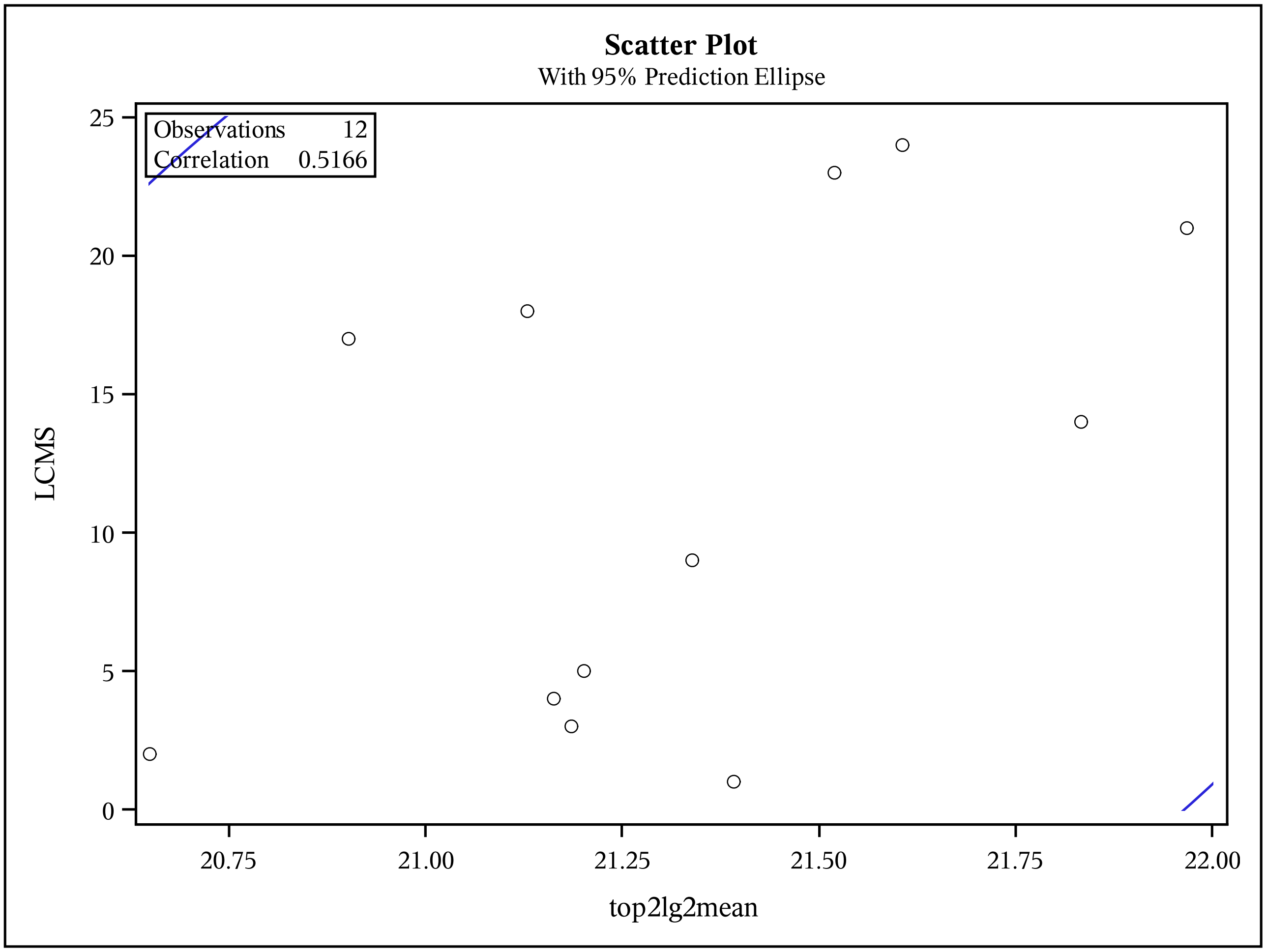

Correlation C_pooltop2lg2mean_tp2 symbol LCMS

The CORR Procedure

Gene_Symbol=APOD

2 Variables:	top2lg2mean LCMS

Simple Statistics						
Variable	N	Mean	Std Dev	Sum	Minimum	Maximum
top2lg2mean	12	21.87122	0.34024	262.45467	21.17123	22.36336
LCMS	12	11.75000	8.70867	141.00000	1.00000	24.00000

Pearson Correlation Coefficients, N = 12<br>Prob > |r| under H0: Rho=0		
	top2lg2mean	LCMS
top2lg2mean	1.00000	-0.32955<br>0.2955
LCMS	-0.32955<br>0.2955	1.00000

Pearson Correlation Statistics (Fisher's z Transformation)									
Variable	With Variable	N	Sample Correlation	Fisher's z	Bias Adjustment	Correlation Estimate	95% Confidence Limits		p Value for H0:Rho=0
top2lg2mean	LCMS	12	-0.32955	-0.34233	-0.01498	-0.31613	-0.753355	0.314899	0.3044

Correlation C_pooltop2lg2mean_tp2 symbol LCMS

The CORR Procedure

Gene_Symbol=APOD

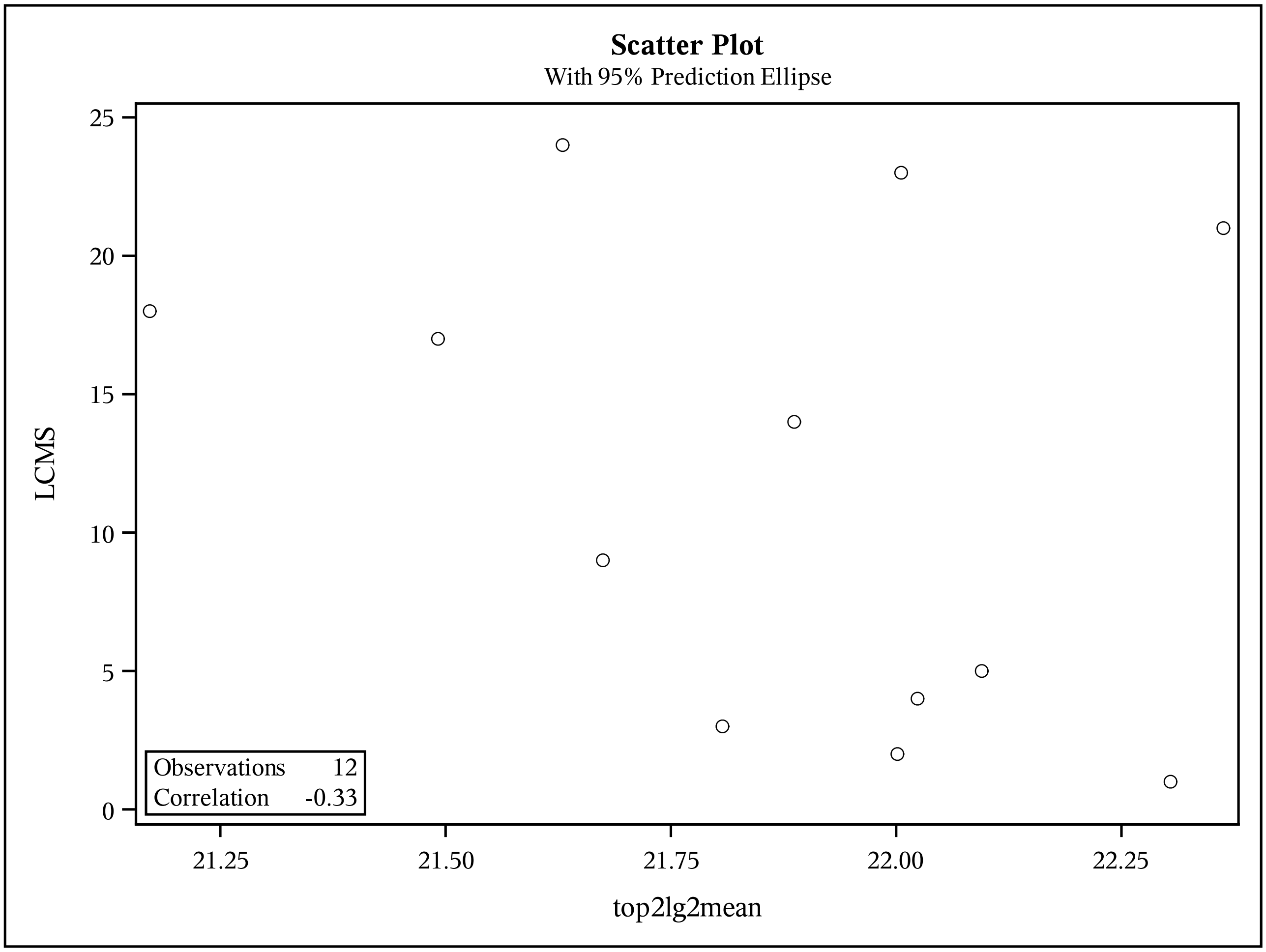

Correlation C_pooltop2lg2mean_tp2 symbol LCMS

The CORR Procedure

Gene_Symbol=APOE

2 Variables:	top2lg2mean LCMS

Simple Statistics						
Variable	N	Mean	Std Dev	Sum	Minimum	Maximum
top2lg2mean	12	23.90888	0.30086	286.90650	23.33682	24.38528
LCMS	12	11.75000	8.70867	141.00000	1.00000	24.00000

Pearson Correlation Coefficients, N = 12<br>Prob > |r| under H0: Rho=0		
	top2lg2mean	LCMS
top2lg2mean	1.00000	-0.07219<br>0.8236
LCMS	-0.07219<br>0.8236	1.00000

Pearson Correlation Statistics (Fisher's z Transformation)										
Variable	With Variable	N	Sample Correlation	Fisher's z	Bias Adjustment	Correlation Estimate	95% Confidence Limits		p Value for H0:Rho=0
top2lg2mean	LCMS	12	-0.07219	-0.07232	-0.00328	-0.06893	-0.618367	0.525774	0.8282

Correlation C_pooltop2lg2mean_tp2 symbol LCMS

The CORR Procedure

Gene_Symbol=APOE

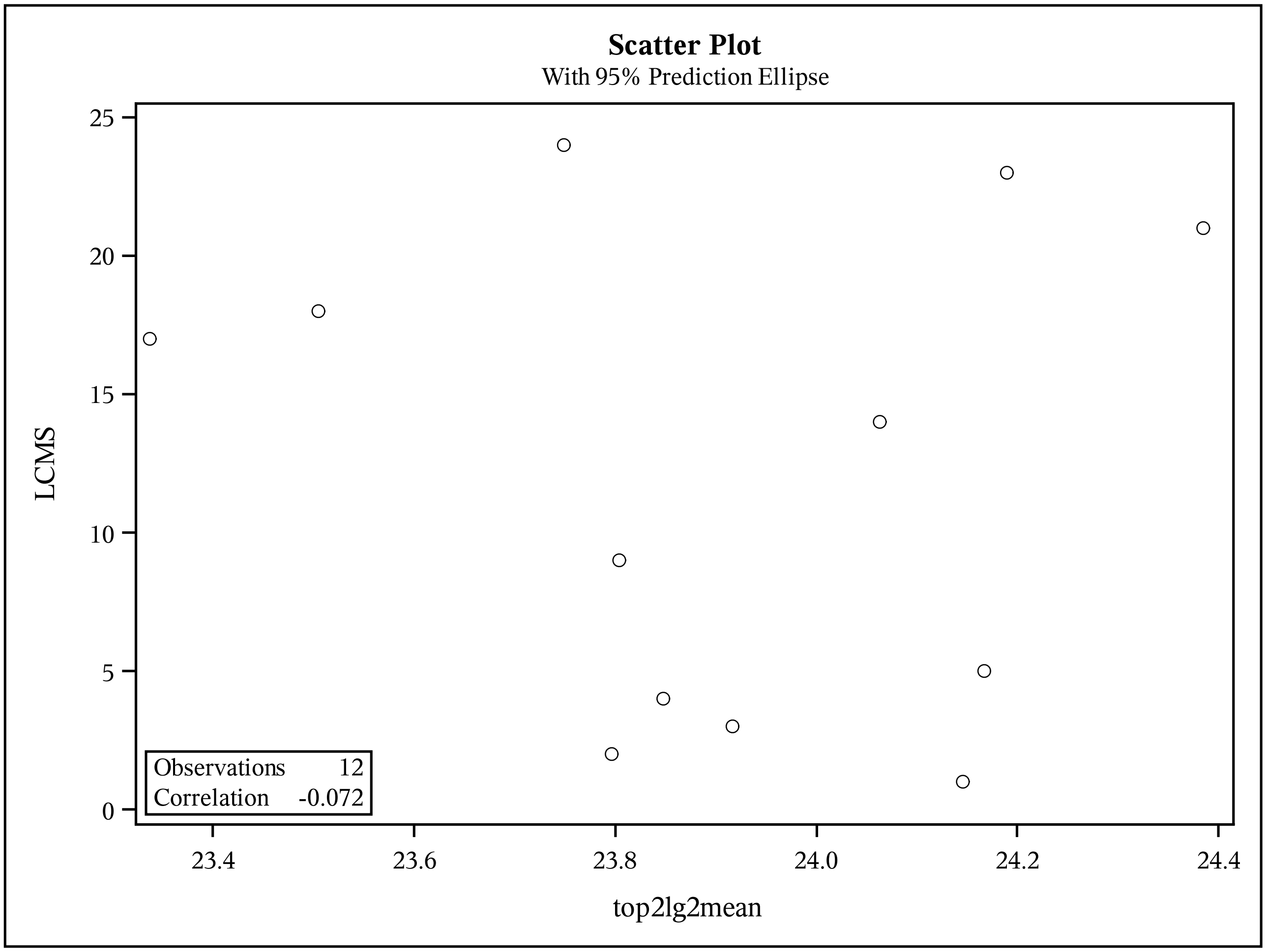

Correlation C_pooltop2lg2mean_tp2 symbol LCMS

The CORR Procedure

Gene_Symbol=APOH

2 Variables:	top2lg2mean LCMS

Simple Statistics						
Variable	N	Mean	Std Dev	Sum	Minimum	Maximum
top2lg2mean	12	22.29174	0.23615	267.50086	21.90547	22.59521
LCMS	12	11.75000	8.70867	141.00000	1.00000	24.00000

Pearson Correlation Coefficients, N = 12<br>Prob > |r| under H0: Rho=0		
	top2lg2mean	LCMS
top2lg2mean	1.00000	-0.40130<br>0.1960
LCMS	-0.40130<br>0.1960	1.00000

Pearson Correlation Statistics (Fisher's z Transformation)									
Variable	With Variable	N	Sample Correlation	Fisher's z	Bias Adjustment	Correlation Estimate	95% Confidence Limits		p Value for H0:Rho=0
top2lg2mean	LCMS	12	-0.40130	-0.42519	-0.01824	-0.38588	-0.785769	0.241501	0.2021

Correlation C_pooltop2lg2mean_tp2 symbol LCMS

The CORR Procedure

Gene_Symbol=APOH

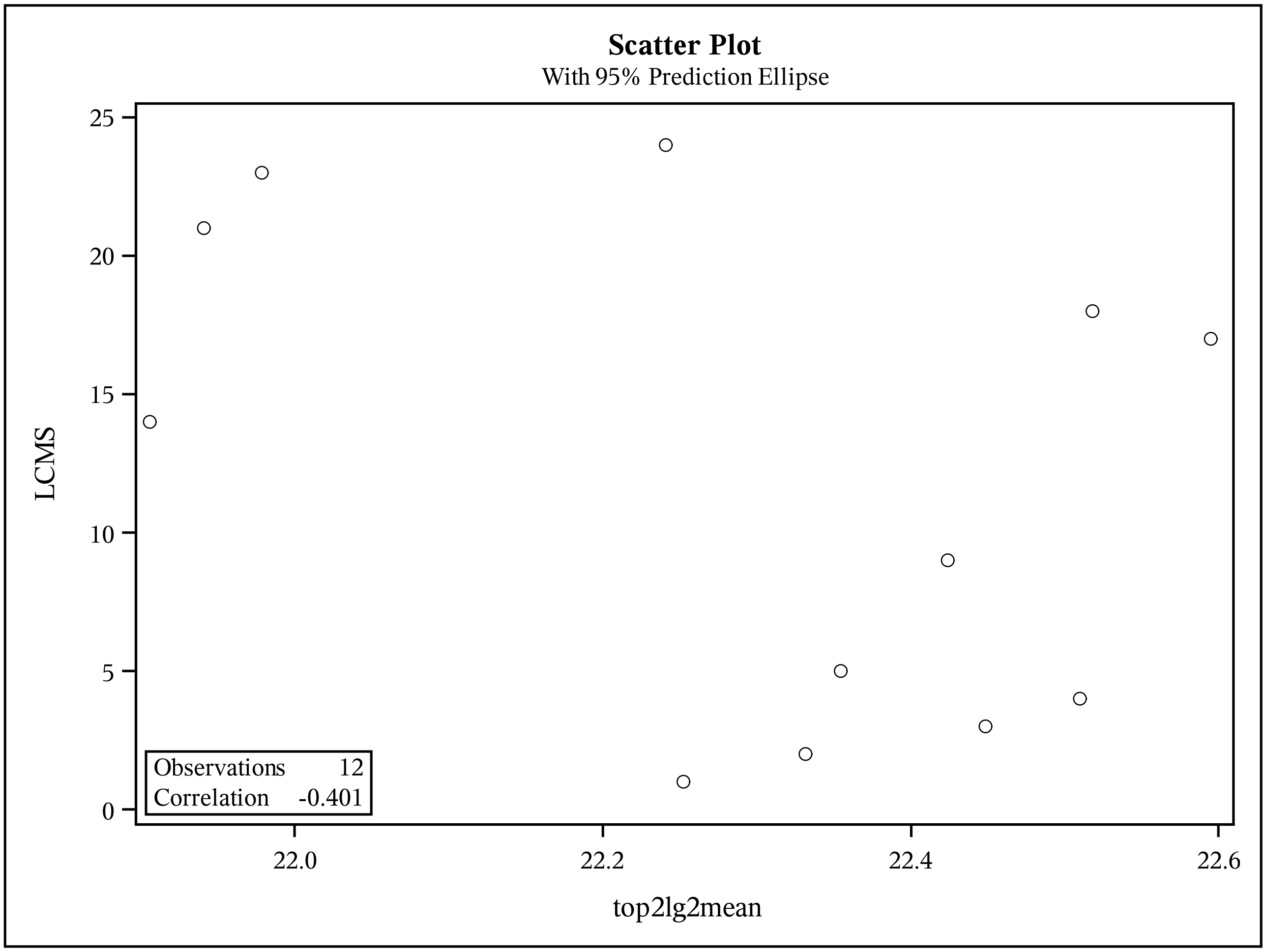

Correlation C_pooltop2lg2mean_tp2 symbol LCMS

The CORR Procedure

Gene_Symbol=APP

2 Variables:	top2lg2mean LCMS

Simple Statistics						
Variable	N	Mean	Std Dev	Sum	Minimum	Maximum
top2lg2mean	12	22.01715	0.31782	264.20575	21.17932	22.35052
LCMS	12	11.75000	8.70867	141.00000	1.00000	24.00000

Pearson Correlation Coefficients, N = 12<br>Prob > |r| under H0: Rho=0		
	top2lg2mean	LCMS
top2lg2mean	1.00000	0.60688<br>0.0364
LCMS	0.60688<br>0.0364	1.00000

Pearson Correlation Statistics (Fisher's z Transformation)									
Variable	With Variable	N	Sample Correlation	Fisher's z	Bias Adjustment	Correlation Estimate	95% Confidence Limits		p Value for H0:Rho=0
top2lg2mean	LCMS	12	0.60688	0.70396	0.02759	0.58916	0.023049	0.869175	0.0347

Correlation C_pooltop2lg2mean_tp2 symbol LCMS

The CORR Procedure

Gene_Symbol=APP

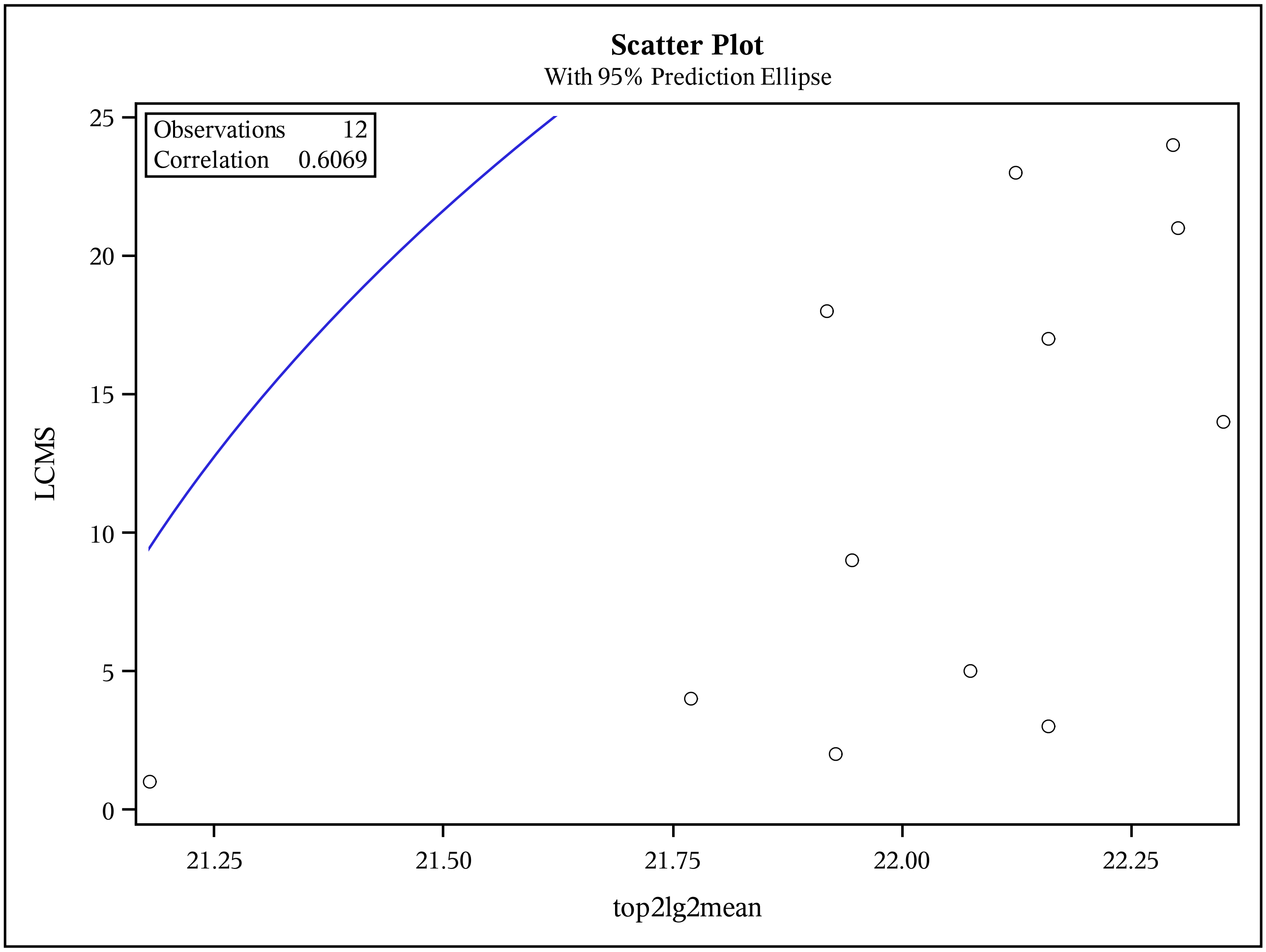

Correlation C_pooltop2lg2mean_tp2 symbol LCMS

The CORR Procedure

Gene_Symbol=AZGP1

2 Variables:	top2lg2mean LCMS

Simple Statistics						
Variable	N	Mean	Std Dev	Sum	Minimum	Maximum
top2lg2mean	12	21.82737	0.10820	261.92846	21.65513	22.02868
LCMS	12	11.75000	8.70867	141.00000	1.00000	24.00000

Pearson Correlation Coefficients, N = 12<br>Prob > |r| under H0: Rho=0		
	top2lg2mean	LCMS
top2lg2mean	1.00000	0.22790<br>0.4762
LCMS	0.22790<br>0.4762	1.00000

Pearson Correlation Statistics (Fisher's z Transformation)									
Variable	With Variable	N	Sample Correlation	Fisher's z	Bias Adjustment	Correlation Estimate	95% Confidence Limits		p Value for H0:Rho=0
top2lg2mean	LCMS	12	0.22790	0.23197	0.01036	0.21806	-0.406746	0.703874	0.4865

Correlation C_pooltop2lg2mean_tp2 symbol LCMS

The CORR Procedure

Gene_Symbol=AZGP1

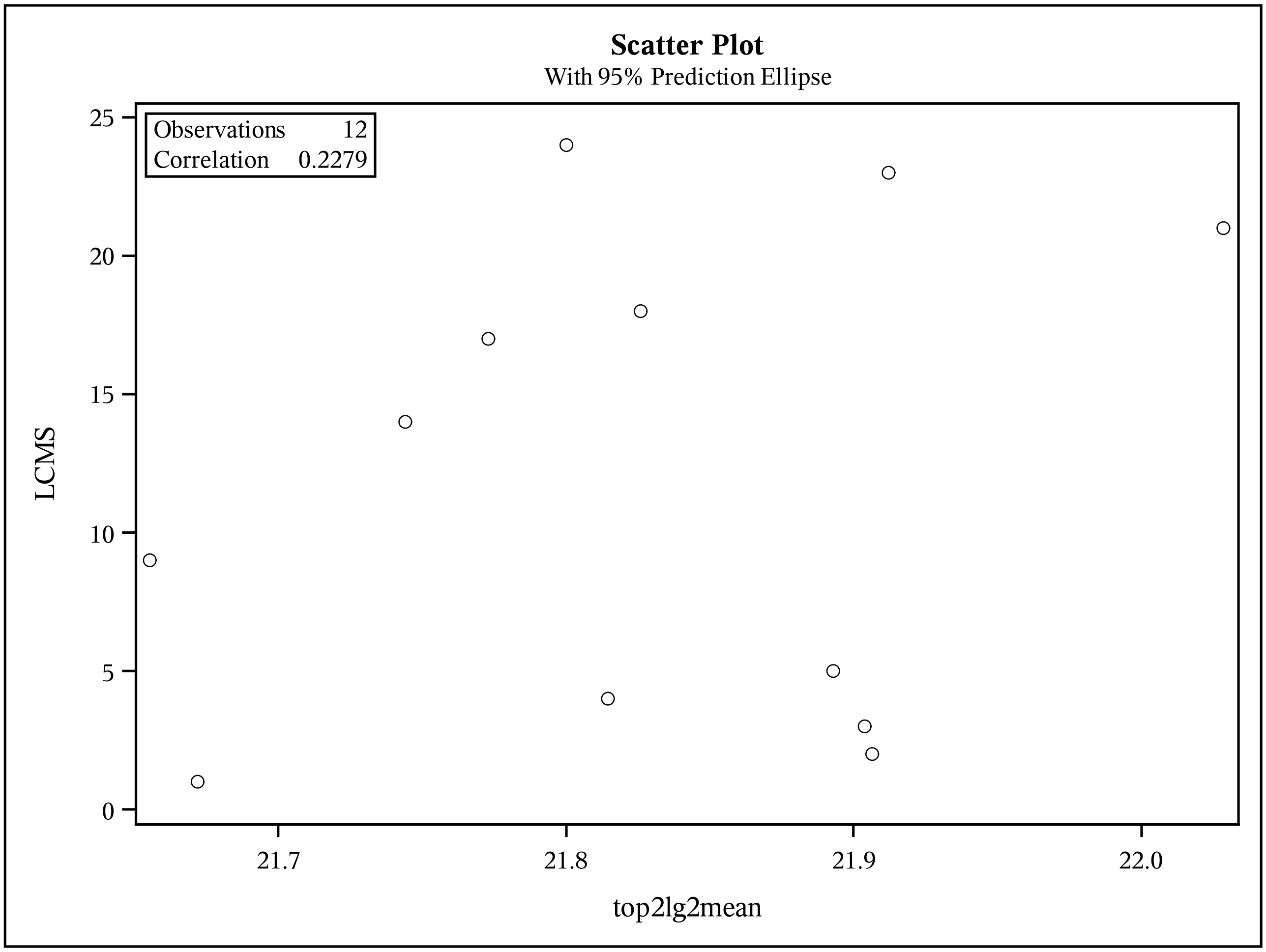

Correlation C_pooltop2lg2mean_tp2 symbol LCMS

The CORR Procedure

Gene_Symbol=B2M

**2 Variables:**	top2lg2mean LCMS

Simple Statistics						
**Variable**	**N**	**Mean**	**Std Dev**	**Sum**	**Minimum**	**Maximum**
**top2lg2mean**	12	23.15555	0.71637	277.86666	22.13271	24.50394
**LCMS**	12	11.75000	8.70867	141.00000	1.00000	24.00000

Pearson Correlation Coefficients, N = 12<br>Prob > |r| under H0: Rho=0		
	**top2lg2mean**	**LCMS**
**top2lg2mean**	1.00000	-0.83141<br>0.0008
**LCMS**	-0.83141<br>0.0008	1.00000

Pearson Correlation Statistics (Fisher's z Transformation)									
**Variable**	**With Variable**	**N**	**Sample Correlation**	**Fisher's z**	**Bias Adjustment**	**Correlation Estimate**	**95% Confidence Limits**		**p Value for H0:Rho=0**
**top2lg2mean**	**LCMS**	12	-0.83141	-1.19269	-0.03779	-0.81937	-0.947651	-0.463359	0.0003

Correlation C_pooltop2lg2mean_tp2 symbol LCMS

The CORR Procedure

Gene_Symbol=B2M

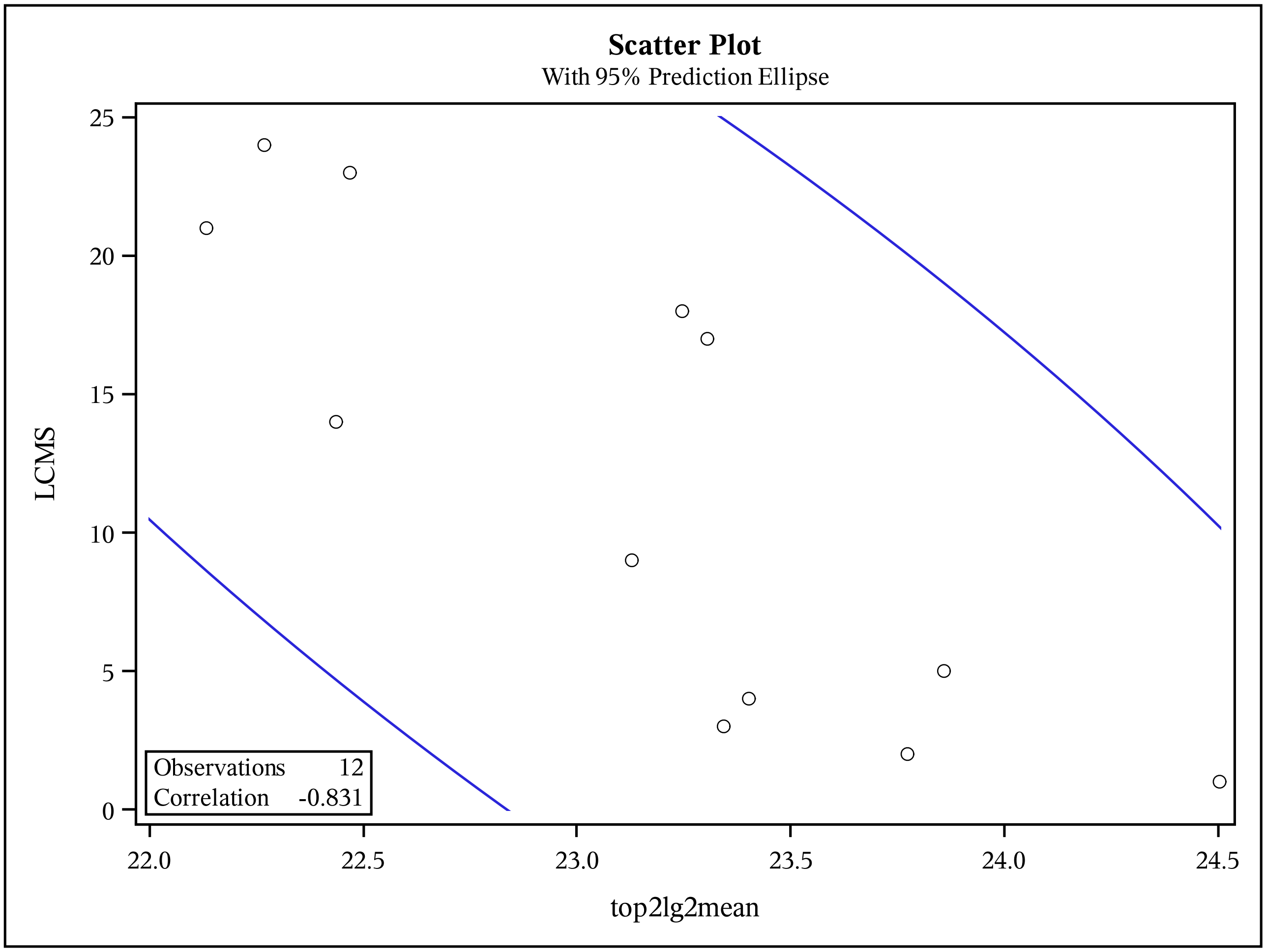

Correlation C_pooltop2lg2mean_tp2 symbol LCMS

The CORR Procedure

Gene_Symbol=B3GNT1

2 Variables:	top2lg2mean LCMS

Simple Statistics						
Variable	N	Mean	Std Dev	Sum	Minimum	Maximum
top2lg2mean	12	21.33297	0.12604	255.99567	21.16096	21.59144
LCMS	12	11.75000	8.70867	141.00000	1.00000	24.00000

Pearson Correlation Coefficients, N = 12<br>Prob > |r| under H0: Rho=0		
	top2lg2mean	LCMS
top2lg2mean	1.00000	0.42500<br>0.1684
LCMS	0.42500<br>0.1684	1.00000

Pearson Correlation Statistics (Fisher's z Transformation)									
Variable	With Variable	N	Sample Correlation	Fisher's z	Bias Adjustment	Correlation Estimate	95% Confidence Limits		p Value for H0:Rho=0
top2lg2mean	LCMS	12	0.42500	0.45378	0.01932	0.40904	-0.215429	0.796068	0.1734

Correlation C_pooltop2lg2mean_tp2 symbol LCMS

The CORR Procedure

Gene_Symbol=B3GNT1

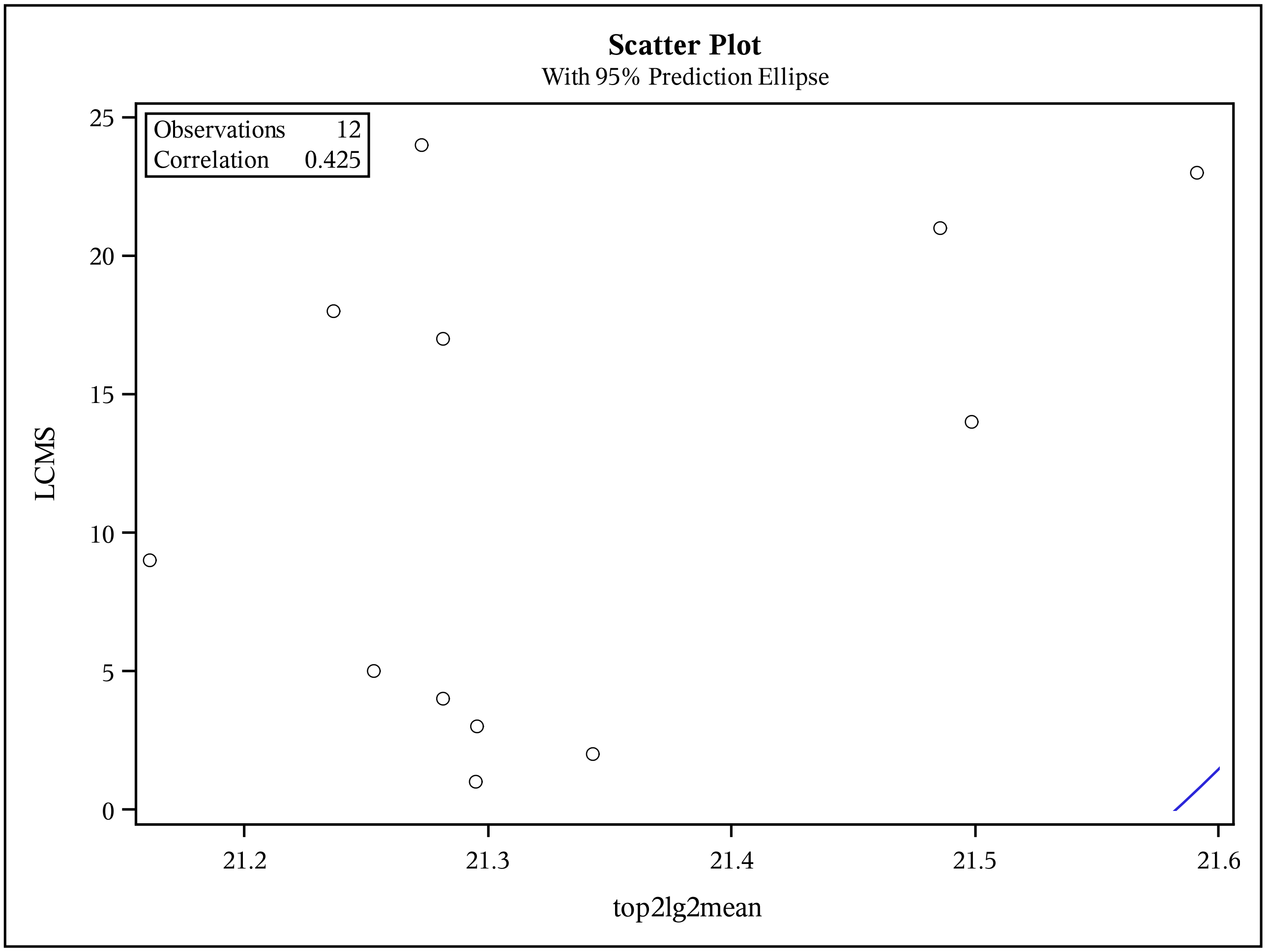

Correlation C_pooltop2lg2mean_tp2 symbol LCMS

The CORR Procedure

Gene_Symbol=BCAN

2 Variables:	top2lg2mean LCMS

Simple Statistics						
Variable	N	Mean	Std Dev	Sum	Minimum	Maximum
top2lg2mean	12	19.52838	0.34793	234.34050	18.70516	20.06111
LCMS	12	11.75000	8.70867	141.00000	1.00000	24.00000

Pearson Correlation Coefficients, N = 12<br>Prob > |r| under H0: Rho=0		
	top2lg2mean	LCMS
top2lg2mean	1.00000	0.48522<br>0.1098
LCMS	0.48522<br>0.1098	1.00000

Pearson Correlation Statistics (Fisher's z Transformation)									
Variable	With Variable	N	Sample Correlation	Fisher's z	Bias Adjustment	Correlation Estimate	95% Confidence Limits		p Value for H0:Rho=0
top2lg2mean	LCMS	12	0.48522	0.52979	0.02206	0.46818	-0.144564	0.821385	0.1120

Correlation C_pooltop2lg2mean_tp2 symbol LCMS

The CORR Procedure

Gene_Symbol=BCAN

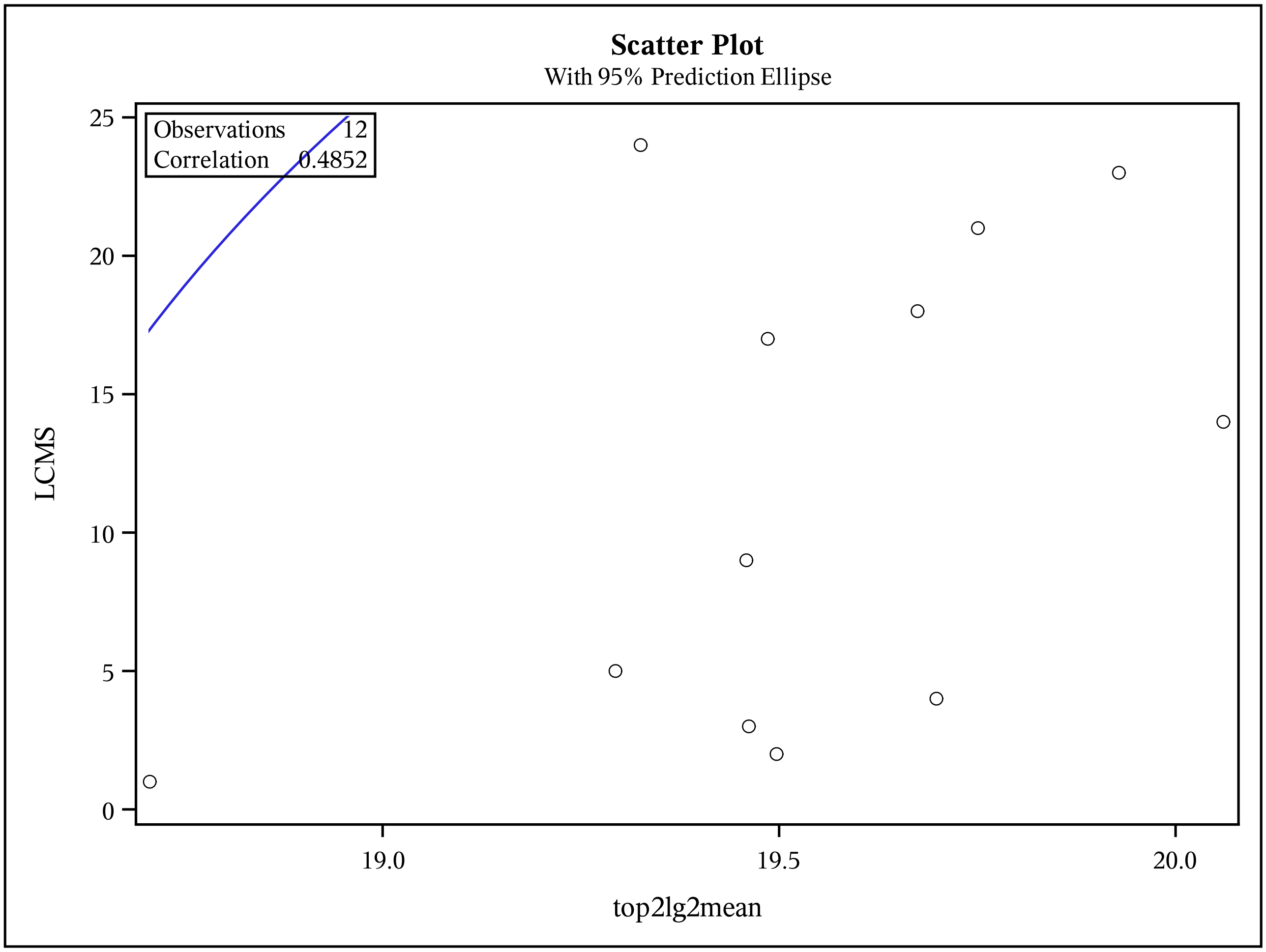

Correlation C_pooltop2lg2mean_tp2 symbol LCMS

The CORR Procedure

Gene_Symbol=C1S

2 Variables:	top2lg2mean LCMS

Simple Statistics						
Variable	N	Mean	Std Dev	Sum	Minimum	Maximum
top2lg2mean	12	20.39884	0.20738	244.78613	20.08916	20.75487
LCMS	12	11.75000	8.70867	141.00000	1.00000	24.00000

Pearson Correlation Coefficients, N = 12<br>Prob > |r| under H0: Rho=0		
	top2lg2mean	LCMS
top2lg2mean	1.00000	0.69720<br>0.0117
LCMS	0.69720<br>0.0117	1.00000

Pearson Correlation Statistics (Fisher's z Transformation)									
Variable	With Variable	N	Sample Correlation	Fisher's z	Bias Adjustment	Correlation Estimate	95% Confidence Limits		p Value for H0:Rho=0
top2lg2mean	LCMS	12	0.69720	0.86184	0.03169	0.68055	0.175003	0.902115	0.0097

Correlation C_pooltop2lg2mean_tp2 symbol LCMS

The CORR Procedure

Gene_Symbol=C1S

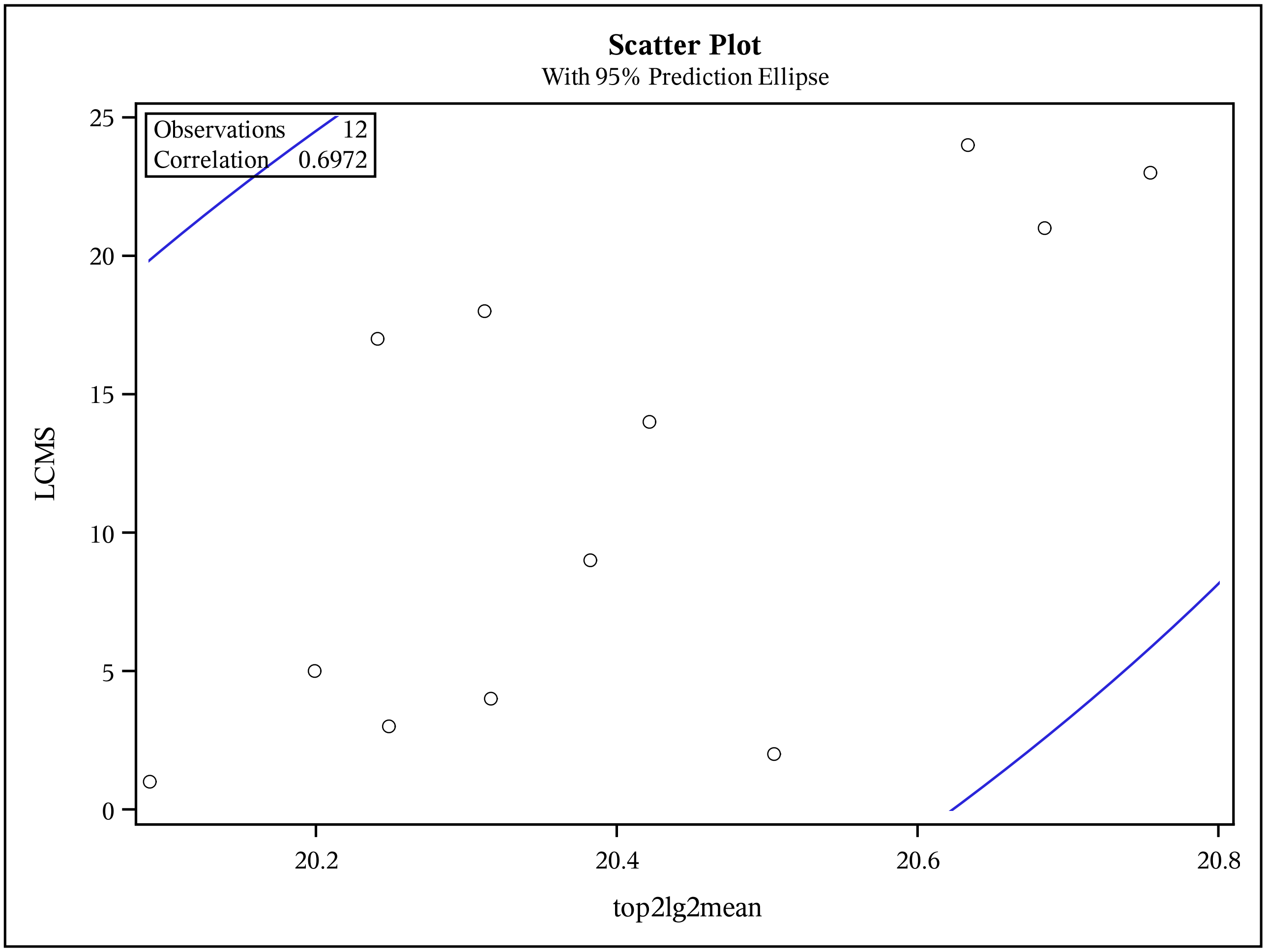

Correlation C_pooltop2lg2mean_tp2 symbol LCMS

The CORR Procedure

Gene_Symbol=C3

2 Variables:	top2lg2mean LCMS

Simple Statistics						
**Variable**	**N**	**Mean**	**Std Dev**	**Sum**	**Minimum**	**Maximum**
**top2lg2mean**	12	23.64879	0.24812	283.78550	23.13520	24.02660
**LCMS**	12	11.75000	8.70867	141.00000	1.00000	24.00000

Pearson Correlation Coefficients, N = 12<br>Prob > |r| under H0: Rho=0		
	**top2lg2mean**	**LCMS**
**top2lg2mean**	1.00000	0.45801<br>0.1343
**LCMS**	0.45801<br>0.1343	1.00000

Pearson Correlation Statistics (Fisher's z Transformation)									
**Variable**	**With Variable**	**N**	**Sample Correlation**	**Fisher's z**	**Bias Adjustment**	**Correlation Estimate**	**95% Confidence Limits**		**p Value for H0:Rho=0**
**top2lg2mean**	**LCMS**	12	0.45801	0.49479	0.02082	0.44140	-0.177450	0.810091	0.1377

Correlation C_pooltop2lg2mean_tp2 symbol LCMS

The CORR Procedure

Gene_Symbol=C3

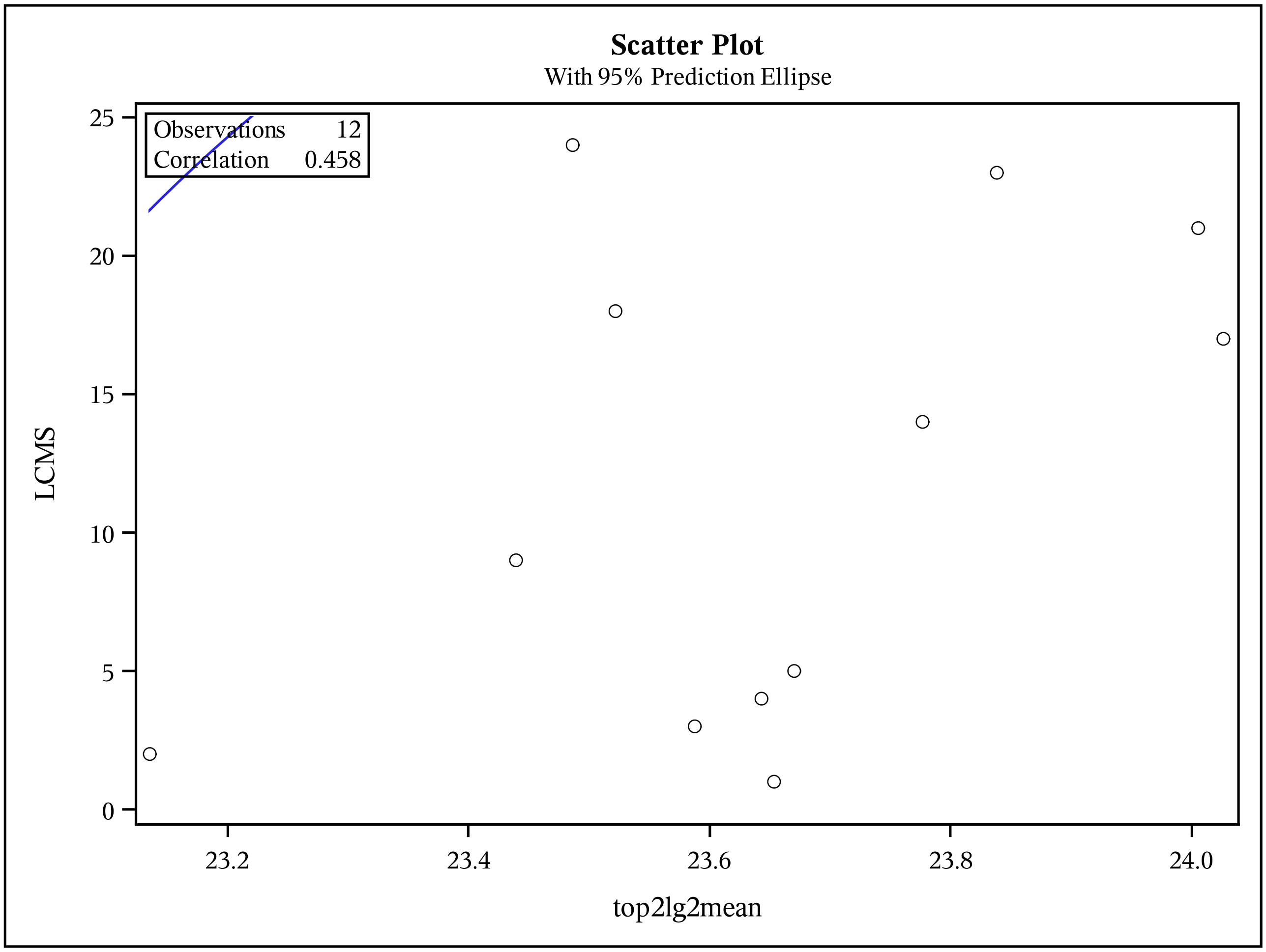

Correlation C_pooltop2lg2mean_tp2 symbol LCMS

The CORR Procedure

Gene_Symbol=C4A-C4B

2 Variables:	top2lg2mean LCMS

Simple Statistics						
**Variable**	**N**	**Mean**	**Std Dev**	**Sum**	**Minimum**	**Maximum**
**top2lg2mean**	12	23.25294	0.43429	279.03527	22.17556	23.98024
**LCMS**	12	11.75000	8.70867	141.00000	1.00000	24.00000

Pearson Correlation Coefficients, N = 12<br>Prob > |r| under H0: Rho=0		
	**top2lg2mean**	**LCMS**
**top2lg2mean**	1.00000	0.61056<br>0.0350
**LCMS**	0.61056<br>0.0350	1.00000

Pearson Correlation Statistics (Fisher's z Transformation)									
**Variable**	**With Variable**	**N**	**Sample Correlation**	**Fisher's z**	**Bias Adjustment**	**Correlation Estimate**	**95% Confidence Limits**		**p Value for H0:Rho=0**
**top2lg2mean**	**LCMS**	12	0.61056	0.70981	0.02775	0.59286	0.028732	0.870559	0.0332

Correlation C_pooltop2lg2mean_tp2 symbol LCMS

The CORR Procedure

Gene_Symbol=C4A-C4B

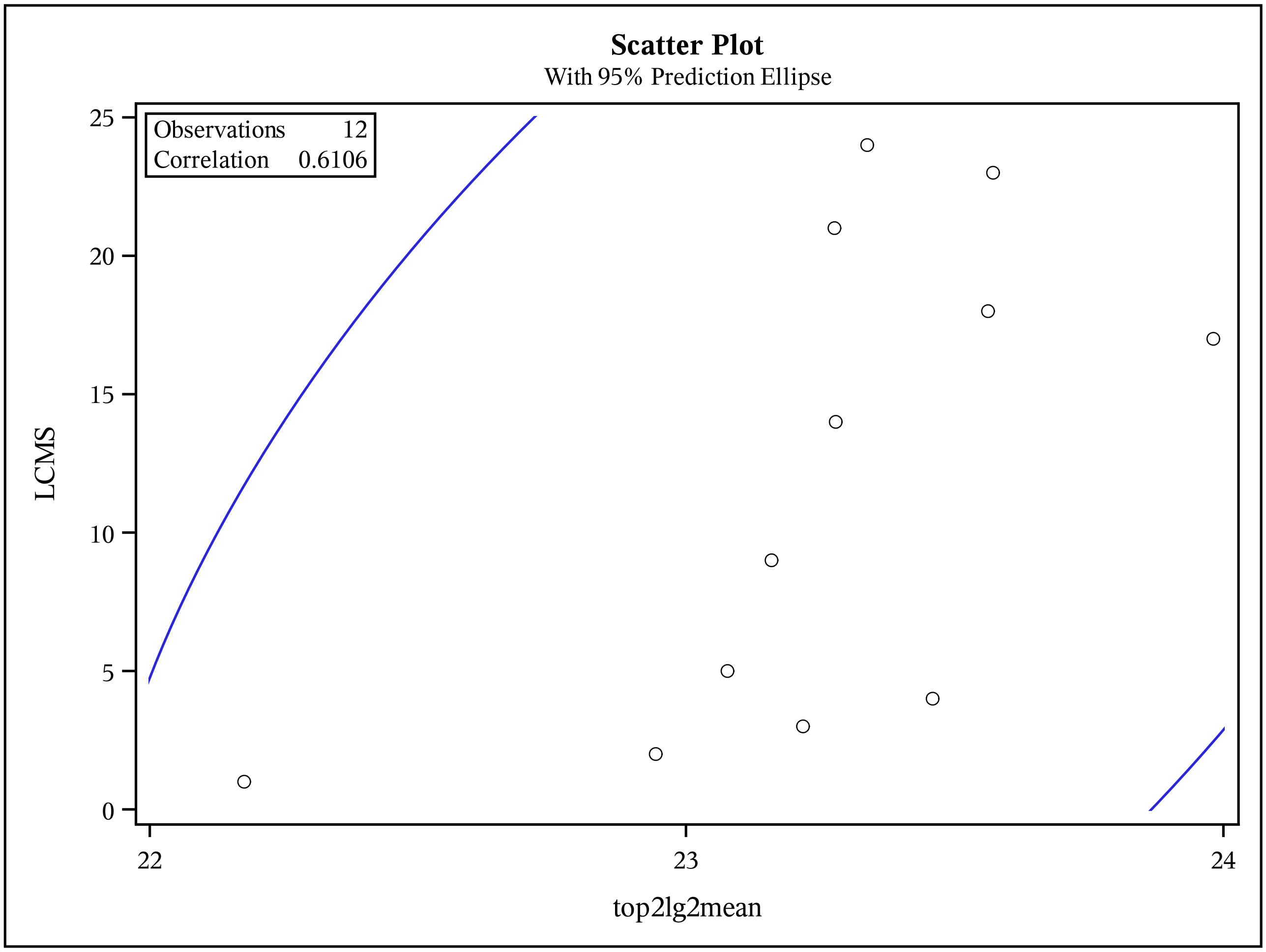

Correlation C_pooltop2lg2mean_tp2 symbol LCMS

The CORR Procedure

Gene_Symbol=C7

2 Variables:	top2lg2mean LCMS

Simple Statistics						
**Variable**	**N**	**Mean**	**Std Dev**	**Sum**	**Minimum**	**Maximum**
**top2lg2mean**	12	19.42969	0.32606	233.15628	18.96587	19.88187
**LCMS**	12	11.75000	8.70867	141.00000	1.00000	24.00000

Pearson Correlation Coefficients, N = 12<br>Prob > |r| under H0: Rho=0		
	**top2lg2mean**	**LCMS**
**top2lg2mean**	1.00000	0.50004<br>0.0978
**LCMS**	0.50004<br>0.0978	1.00000

Pearson Correlation Statistics (Fisher's z Transformation)										
**Variable**	**With Variable**	**N**	**Sample Correlation**	**Fisher's z**	**Bias Adjustment**	**Correlation Estimate**	**95% Confidence Limits**		**p Value for H0:Rho=0**
**top2lg2mean**	**LCMS**	12	0.50004	0.54936	0.02273	0.48280	-0.126015	0.827437	0.0993

Correlation C_pooltop2lg2mean_tp2 symbol LCMS

The CORR Procedure

Gene_Symbol=C7

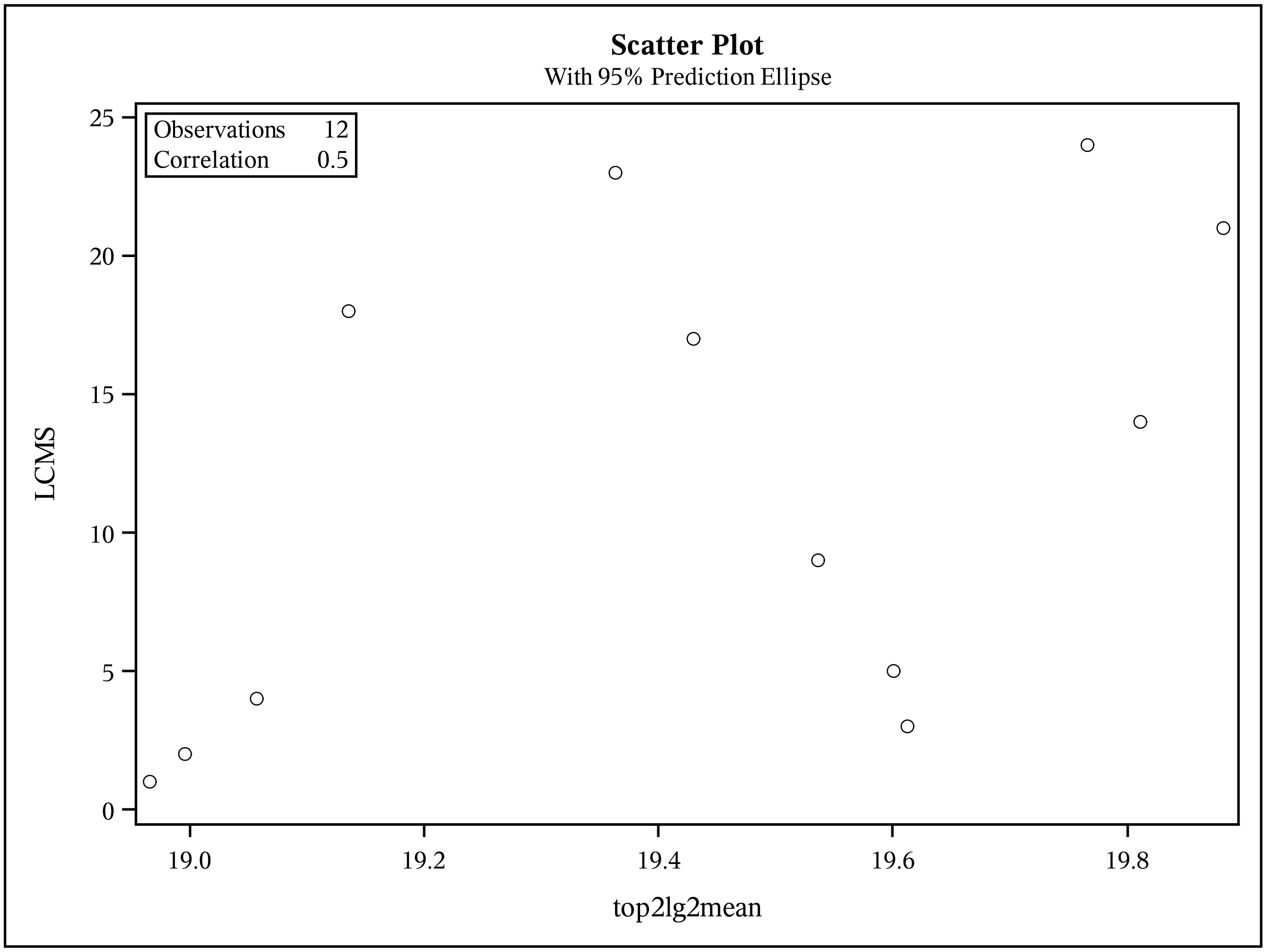

Correlation C_pooltop2lg2mean_tp2 symbol LCMS

The CORR Procedure

Gene_Symbol=CD14

2 Variables:	top2lg2mean LCMS

Simple Statistics						
Variable	N	Mean	Std Dev	Sum	Minimum	Maximum
top2lg2mean	12	21.00887	0.30714	252.10641	20.57639	21.50118
LCMS	12	11.75000	8.70867	141.00000	1.00000	24.00000

Pearson Correlation Coefficients, N = 12<br>Prob > |r| under H0: Rho=0		
	top2lg2mean	LCMS
top2lg2mean	1.00000	0.53670<br>0.0720
LCMS	0.53670<br>0.0720	1.00000

Pearson Correlation Statistics (Fisher's z Transformation)									
Variable	With Variable	N	Sample Correlation	Fisher's z	Bias Adjustment	Correlation Estimate	95% Confidence Limits		p Value for H0:Rho=0
top2lg2mean	LCMS	12	0.53670	0.59951	0.02440	0.51911	-0.078047	0.842125	0.0721

Correlation C_pooltop2lg2mean_tp2 symbol LCMS

The CORR Procedure

Gene_Symbol=CD14

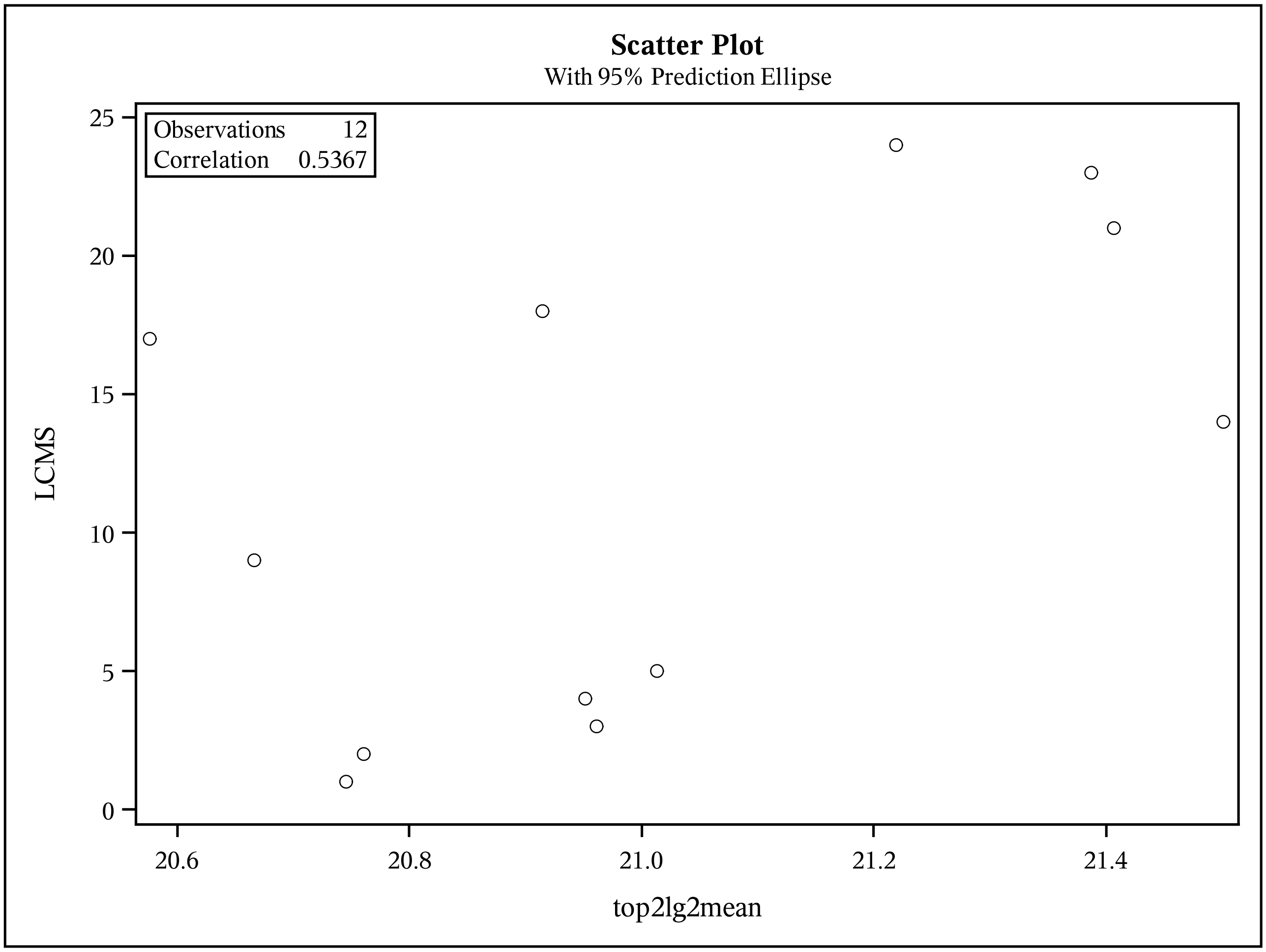

Correlation C_pooltop2lg2mean_tp2 symbol LCMS

The CORR Procedure

Gene_Symbol=CD59

2 Variables:	top2lg2mean LCMS

Simple Statistics						
Variable	N	Mean	Std Dev	Sum	Minimum	Maximum
top2lg2mean	12	20.70554	0.36595	248.46650	19.73407	21.12204
LCMS	12	11.75000	8.70867	141.00000	1.00000	24.00000

Pearson Correlation Coefficients, N = 12<br>Prob > |r| under H0: Rho=0		
	top2lg2mean	LCMS
top2lg2mean	1.00000	0.39848<br>0.1995
LCMS	0.39848<br>0.1995	1.00000

Pearson Correlation Statistics (Fisher's z Transformation)									
Variable	With Variable	N	Sample Correlation	Fisher's z	Bias Adjustment	Correlation Estimate	95% Confidence Limits		p Value for H0:Rho=0
top2lg2mean	LCMS	12	0.39848	0.42184	0.01811	0.38314	-0.244533	0.784533	0.2057

Correlation C_pooltop2lg2mean_tp2 symbol LCMS

The CORR Procedure

Gene_Symbol=CD59

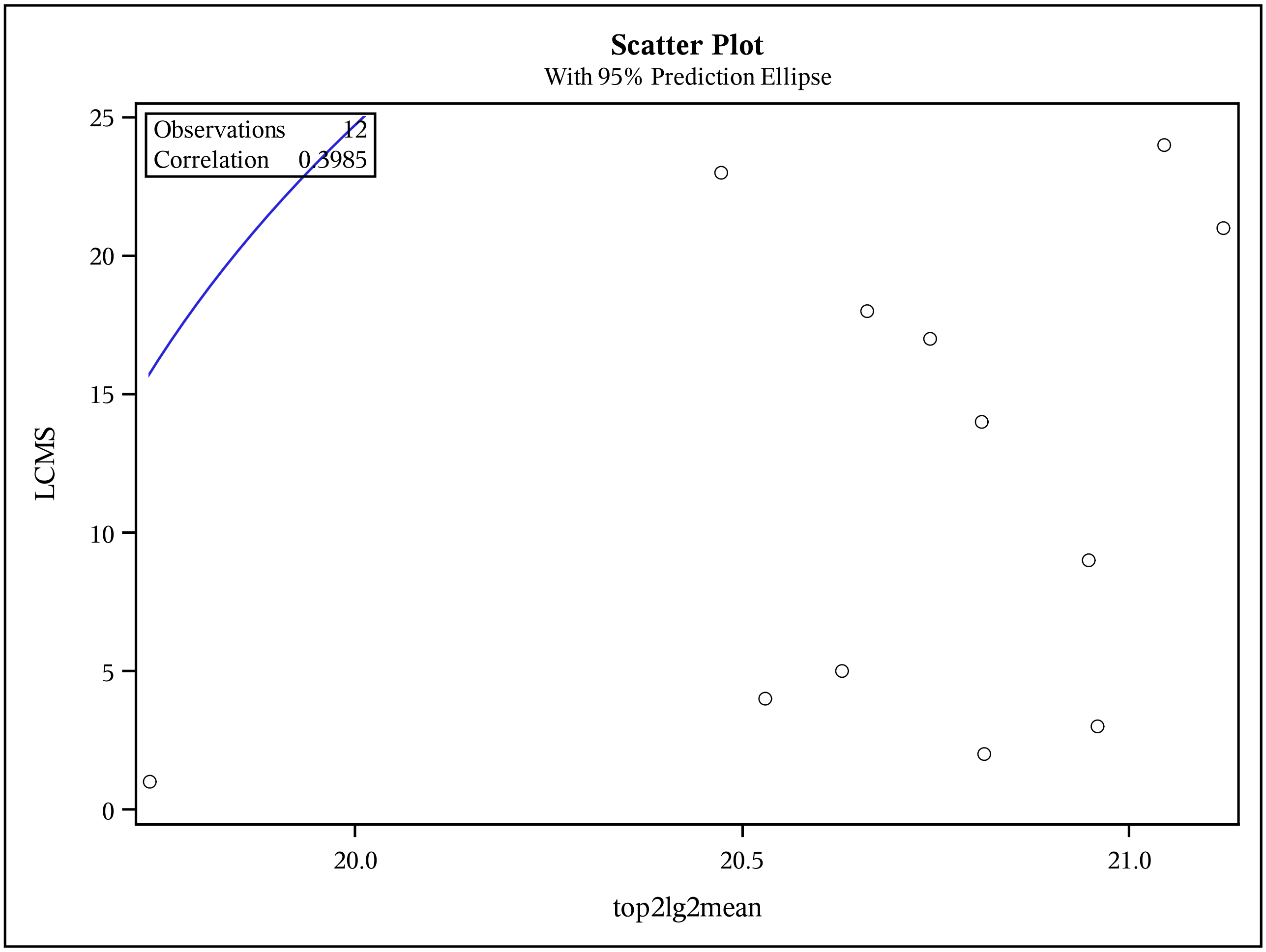

Correlation C_pooltop2lg2mean_tp2 symbol LCMS

The CORR Procedure

Gene_Symbol=CFB

2 Variables:	top2lg2mean LCMS

Simple Statistics						
**Variable**	**N**	**Mean**	**Std Dev**	**Sum**	**Minimum**	**Maximum**
**top2lg2mean**	12	20.10731	0.36953	241.28777	19.43929	20.88184
**LCMS**	12	11.75000	8.70867	141.00000	1.00000	24.00000

Pearson Correlation Coefficients, N = 12<br>Prob > |r| under H0: Rho=0		
	**top2lg2mean**	**LCMS**
**top2lg2mean**	1.00000	0.49220<br>0.1041
**LCMS**	0.49220<br>0.1041	1.00000

Pearson Correlation Statistics (Fisher's z Transformation)									
**Variable**	**With Variable**	**N**	**Sample Correlation**	**Fisher's z**	**Bias Adjustment**	**Correlation Estimate**	**95% Confidence Limits**		**p Value for H0:Rho=0**
**top2lg2mean**	**LCMS**	12	0.49220	0.53896	0.02237	0.47506	-0.135889	0.824243	0.1059

Correlation C_pooltop2lg2mean_tp2 symbol LCMS

The CORR Procedure

Gene_Symbol=CFB

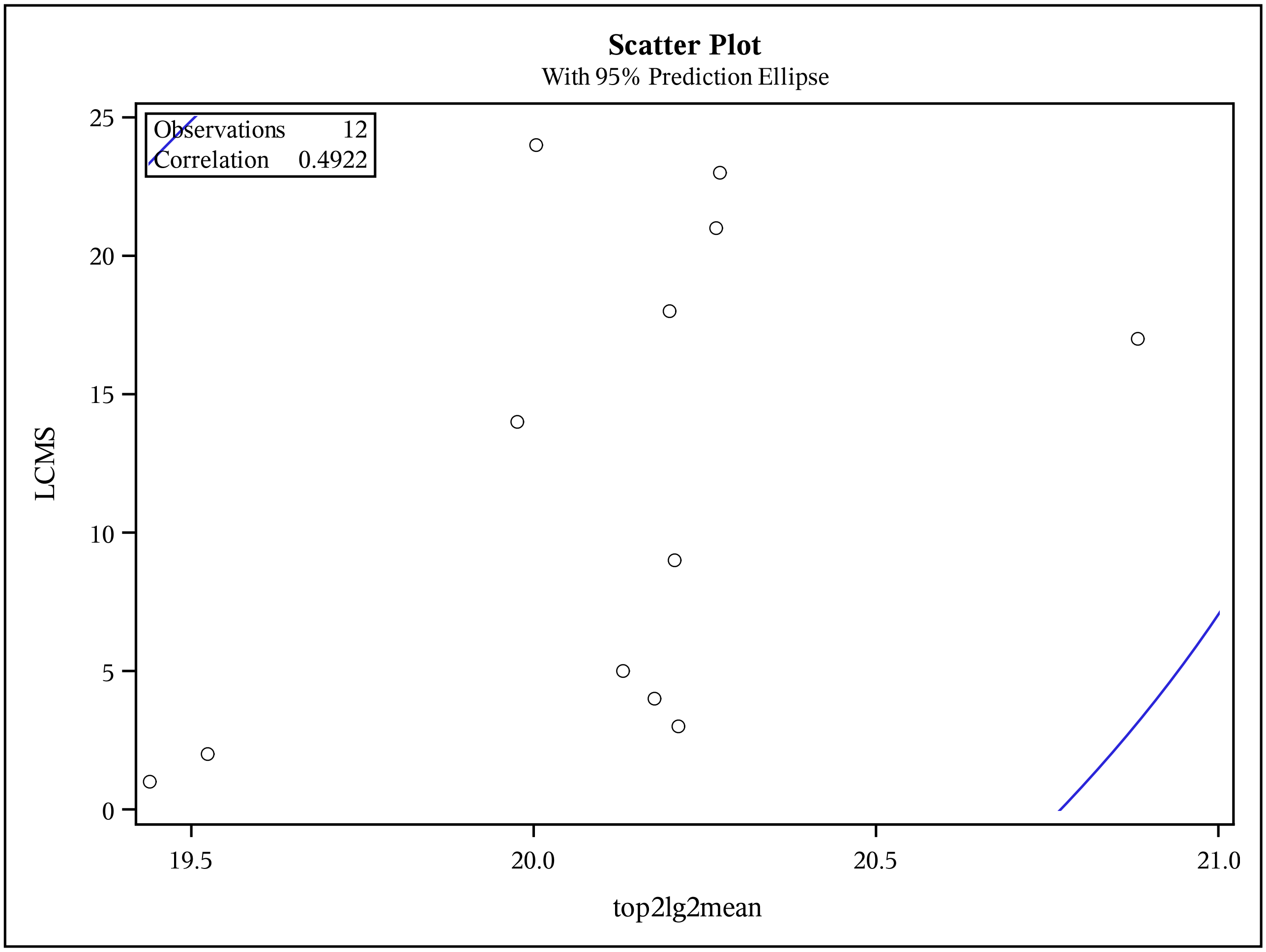

Correlation C_pooltop2lg2mean_tp2 symbol LCMS

The CORR Procedure

Gene_Symbol=CFH

**2 Variables:**	top2lg2mean LCMS

Simple Statistics						
**Variable**	**N**	**Mean**	**Std Dev**	**Sum**	**Minimum**	**Maximum**
**top2lg2mean**	12	20.28654	0.23510	243.43848	19.71553	20.55740
**LCMS**	12	11.75000	8.70867	141.00000	1.00000	24.00000

Pearson Correlation Coefficients, N = 12<br>Prob > |r| under H0: Rho=0		
	**top2lg2mean**	**LCMS**
**top2lg2mean**	1.00000	0.63336<br>0.0270
**LCMS**	0.63336<br>0.0270	1.00000

Pearson Correlation Statistics (Fisher's z Transformation)									
**Variable**	**With Variable**	**N**	**Sample Correlation**	**Fisher's z**	**Bias Adjustment**	**Correlation Estimate**	**95% Confidence Limits**		**p Value for H0:Rho=0**
**top2lg2mean**	**LCMS**	12	0.63336	0.74701	0.02879	0.61581	0.064809	0.879043	0.0250

Correlation C_pooltop2lg2mean_tp2 symbol LCMS

The CORR Procedure

Gene_Symbol=CFH

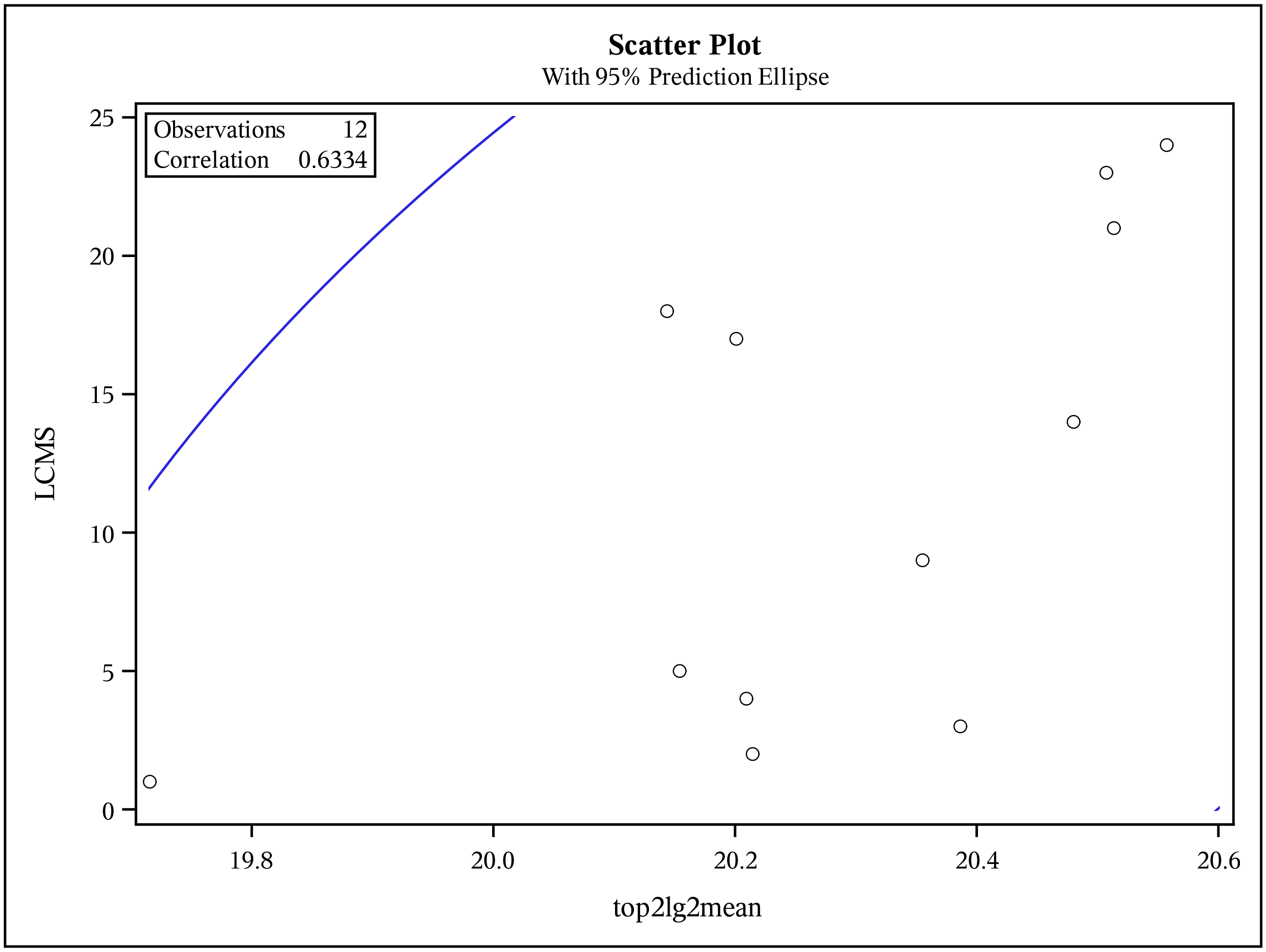

Correlation C_pooltop2lg2mean_tp2 symbol LCMS

The CORR Procedure

Gene_Symbol=CHGA

2 Variables:	top2lg2mean LCMS

Simple Statistics						
Variable	N	Mean	Std Dev	Sum	Minimum	Maximum
top2lg2mean	12	22.61559	0.18595	271.38709	22.31285	22.88093
LCMS	12	11.75000	8.70867	141.00000	1.00000	24.00000

Pearson Correlation Coefficients, N = 12<br>Prob > |r| under H0: Rho=0		
	top2lg2mean	LCMS
top2lg2mean	1.00000	0.14704<br>0.6484
LCMS	0.14704<br>0.6484	1.00000

Pearson Correlation Statistics (Fisher's z Transformation)									
Variable	With Variable	N	Sample Correlation	Fisher's z	Bias Adjustment	Correlation Estimate	95% Confidence Limits		p Value for H0:Rho=0
top2lg2mean	LCMS	12	0.14704	0.14812	0.00668	0.14050	-0.471416	0.661093	0.6568

Correlation C_pooltop2lg2mean_tp2 symbol LCMS

The CORR Procedure

Gene_Symbol=CHGA

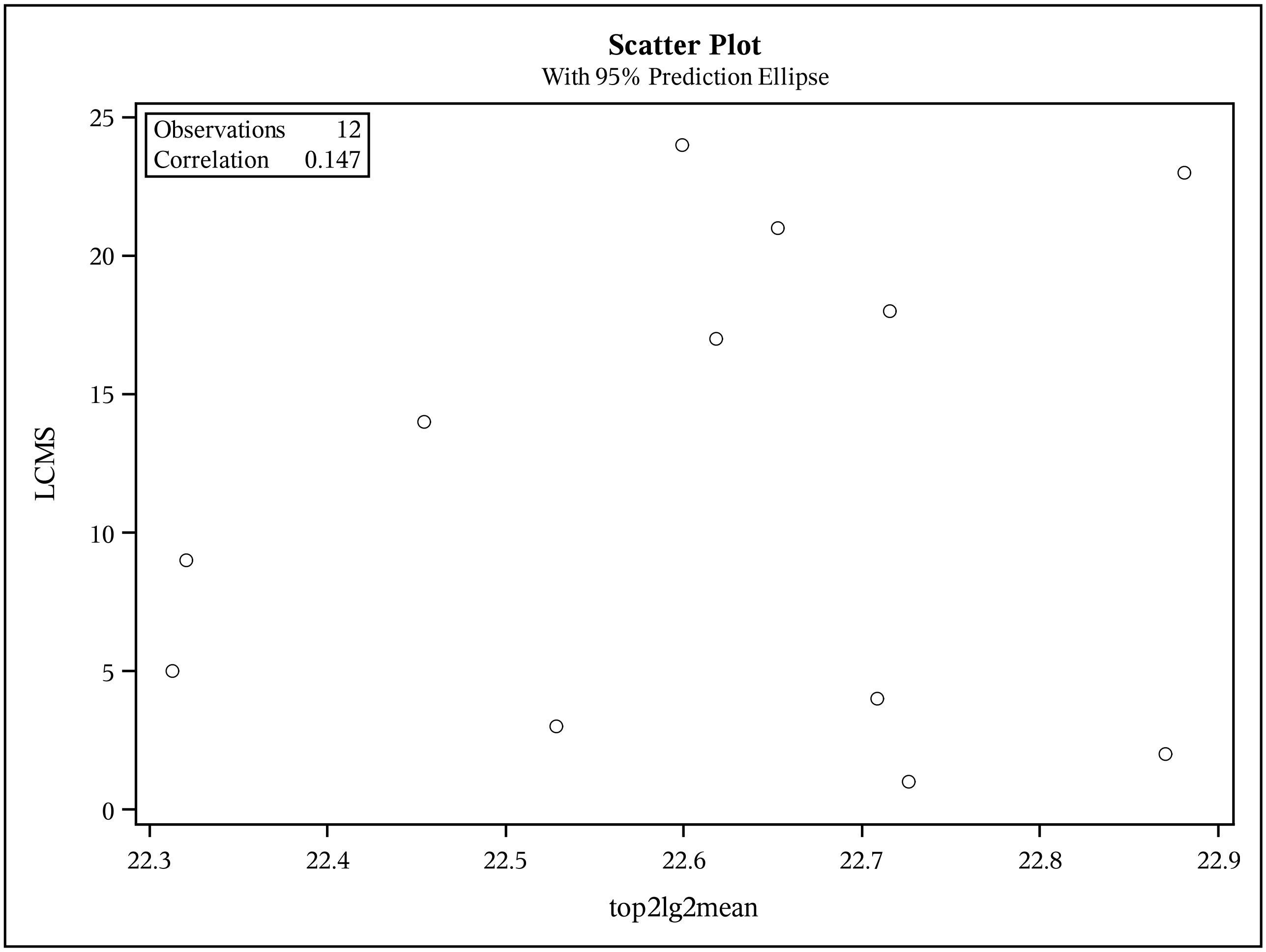

Correlation C_pooltop2lg2mean_tp2 symbol LCMS

The CORR Procedure

Gene_Symbol=CHGB

2 Variables:	top2lg2mean LCMS

Simple Statistics						
Variable	N	Mean	Std Dev	Sum	Minimum	Maximum
top2lg2mean	12	22.22482	0.18428	266.69786	21.95818	22.55311
LCMS	12	11.75000	8.70867	141.00000	1.00000	24.00000

Pearson Correlation Coefficients, N = 12<br>Prob > |r| under H0: Rho=0		
	top2lg2mean	LCMS
top2lg2mean	1.00000	0.10246<br>0.7513
LCMS	0.10246<br>0.7513	1.00000

Pearson Correlation Statistics (Fisher's z Transformation)									
Variable	With Variable	N	Sample Correlation	Fisher's z	Bias Adjustment	Correlation Estimate	95% Confidence Limits		p Value for H0:Rho=0
top2lg2mean	LCMS	12	0.10246	0.10282	0.00466	0.09785	-0.504374	0.636035	0.7577

Correlation C_pooltop2lg2mean_tp2 symbol LCMS

The CORR Procedure

Gene_Symbol=CHGB

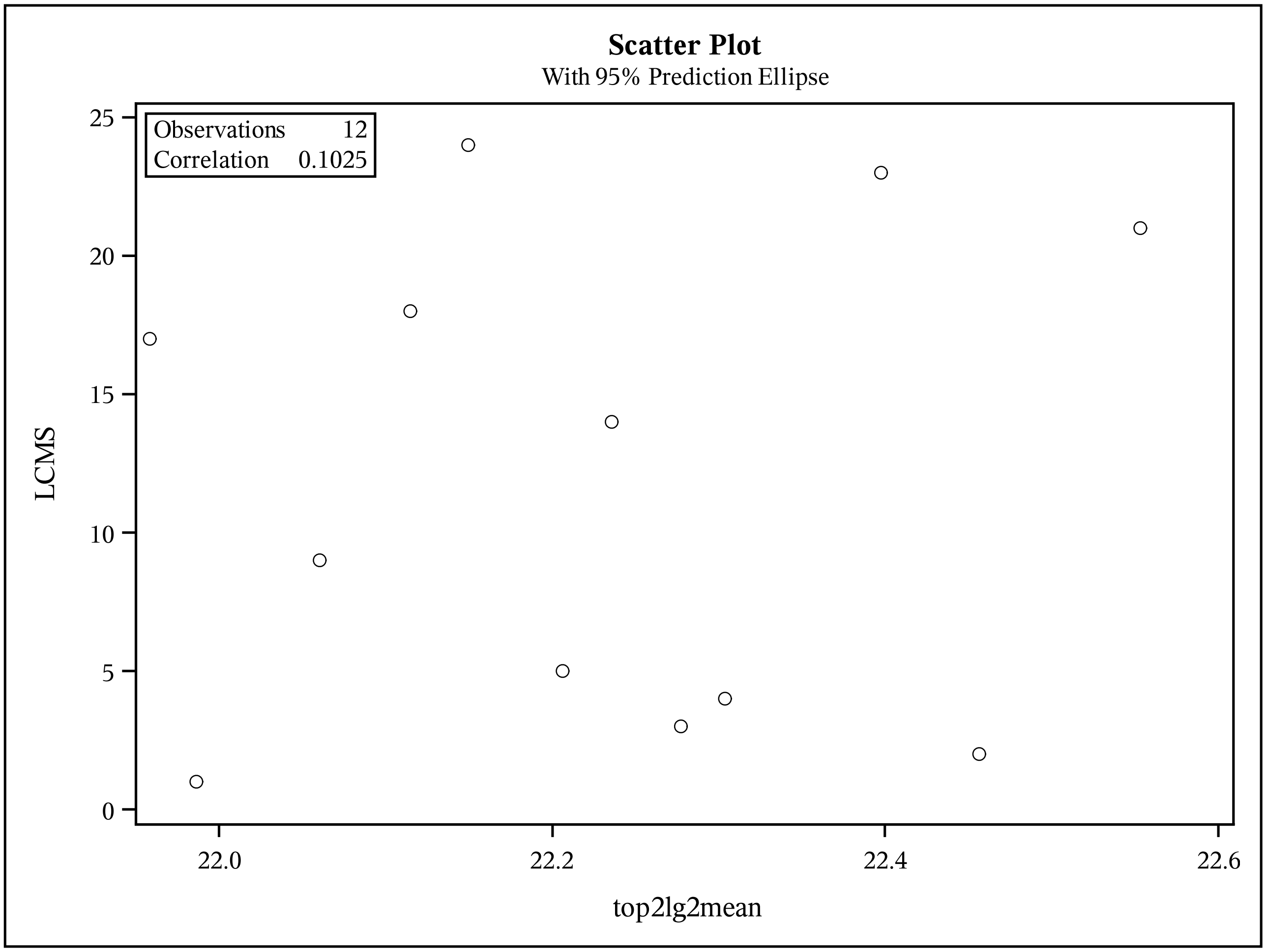

Correlation C_pooltop2lg2mean_tp2 symbol LCMS

The CORR Procedure

Gene_Symbol=CHL1

2 Variables:	top2lg2mean LCMS

Simple Statistics						
Variable	N	Mean	Std Dev	Sum	Minimum	Maximum
top2lg2mean	12	20.32975	0.64128	243.95697	19.43727	21.37758
LCMS	12	11.75000	8.70867	141.00000	1.00000	24.00000

Pearson Correlation Coefficients, N = 12<br>Prob > |r| under H0: Rho=0		
	top2lg2mean	LCMS
top2lg2mean	1.00000	0.38822<br>0.2124
LCMS	0.38822<br>0.2124	1.00000

Pearson Correlation Statistics (Fisher's z Transformation)									
Variable	With Variable	N	Sample Correlation	Fisher's z	Bias Adjustment	Correlation Estimate	95% Confidence Limits		p Value for H0:Rho=0
top2lg2mean	LCMS	12	0.38822	0.40971	0.01765	0.37313	-0.255475	0.780004	0.2190

Correlation C_pooltop2lg2mean_tp2 symbol LCMS

The CORR Procedure

Gene_Symbol=CHL1

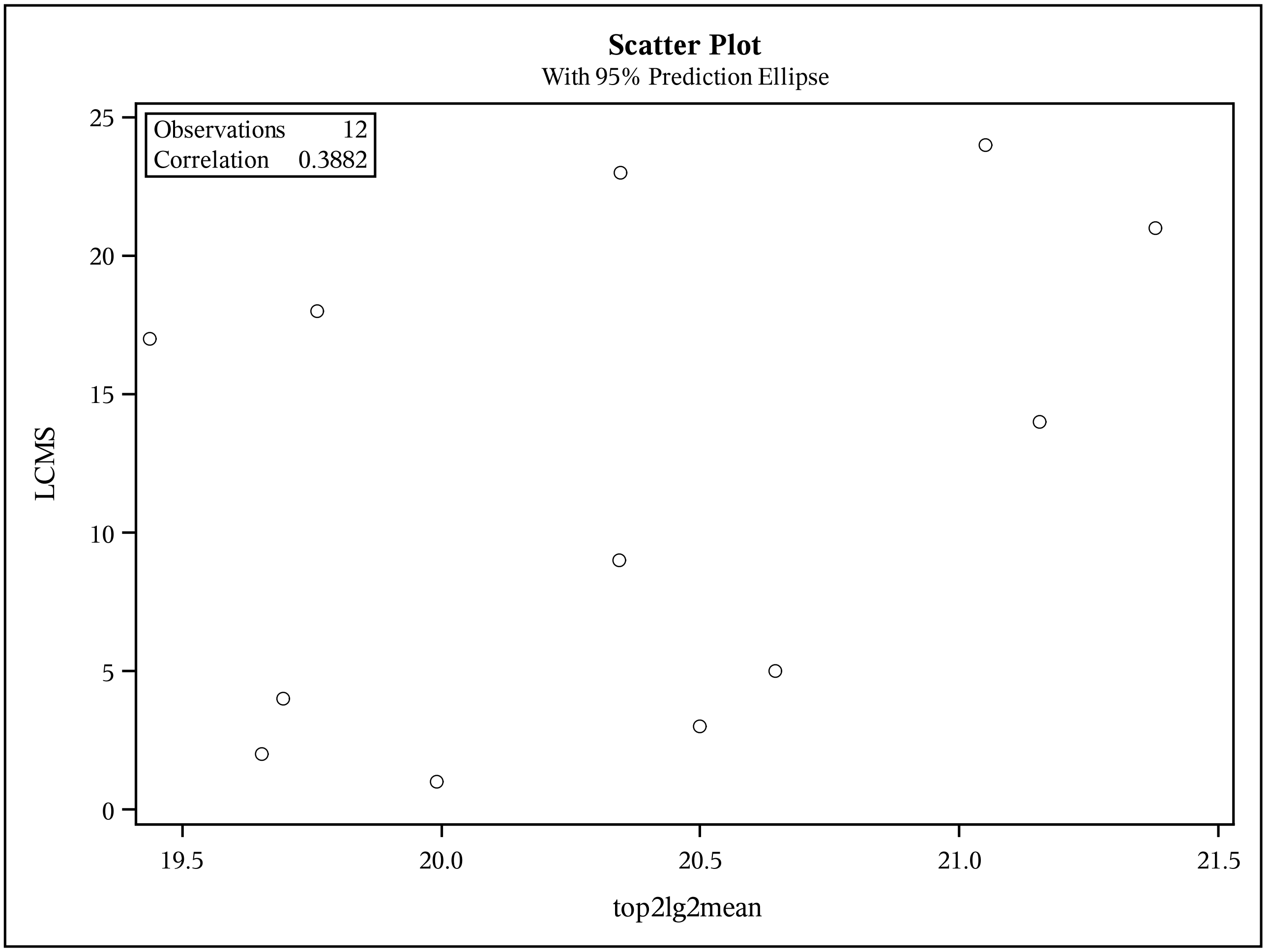

Correlation C_pooltop2lg2mean_tp2 symbol LCMS

The CORR Procedure

Gene_Symbol=CLU

2 Variables:	top2lg2mean LCMS

Simple Statistics						
Variable	N	Mean	Std Dev	Sum	Minimum	Maximum
top2lg2mean	12	24.56620	0.25894	294.79441	24.03016	24.87867
LCMS	12	11.75000	8.70867	141.00000	1.00000	24.00000

Pearson Correlation Coefficients, N = 12<br>Prob > |r| under H0: Rho=0		
	top2lg2mean	LCMS
top2lg2mean	1.00000	0.72523<br>0.0076
LCMS	0.72523<br>0.0076	1.00000

Pearson Correlation Statistics (Fisher's z Transformation)									
Variable	With Variable	N	Sample Correlation	Fisher's z	Bias Adjustment	Correlation Estimate	95% Confidence Limits		p Value for H0:Rho=0
top2lg2mean	LCMS	12	0.72523	0.91859	0.03296	0.70922	0.228211	0.911943	0.0059

Correlation C_pooltop2lg2mean_tp2 symbol LCMS

The CORR Procedure

Gene_Symbol=CLU

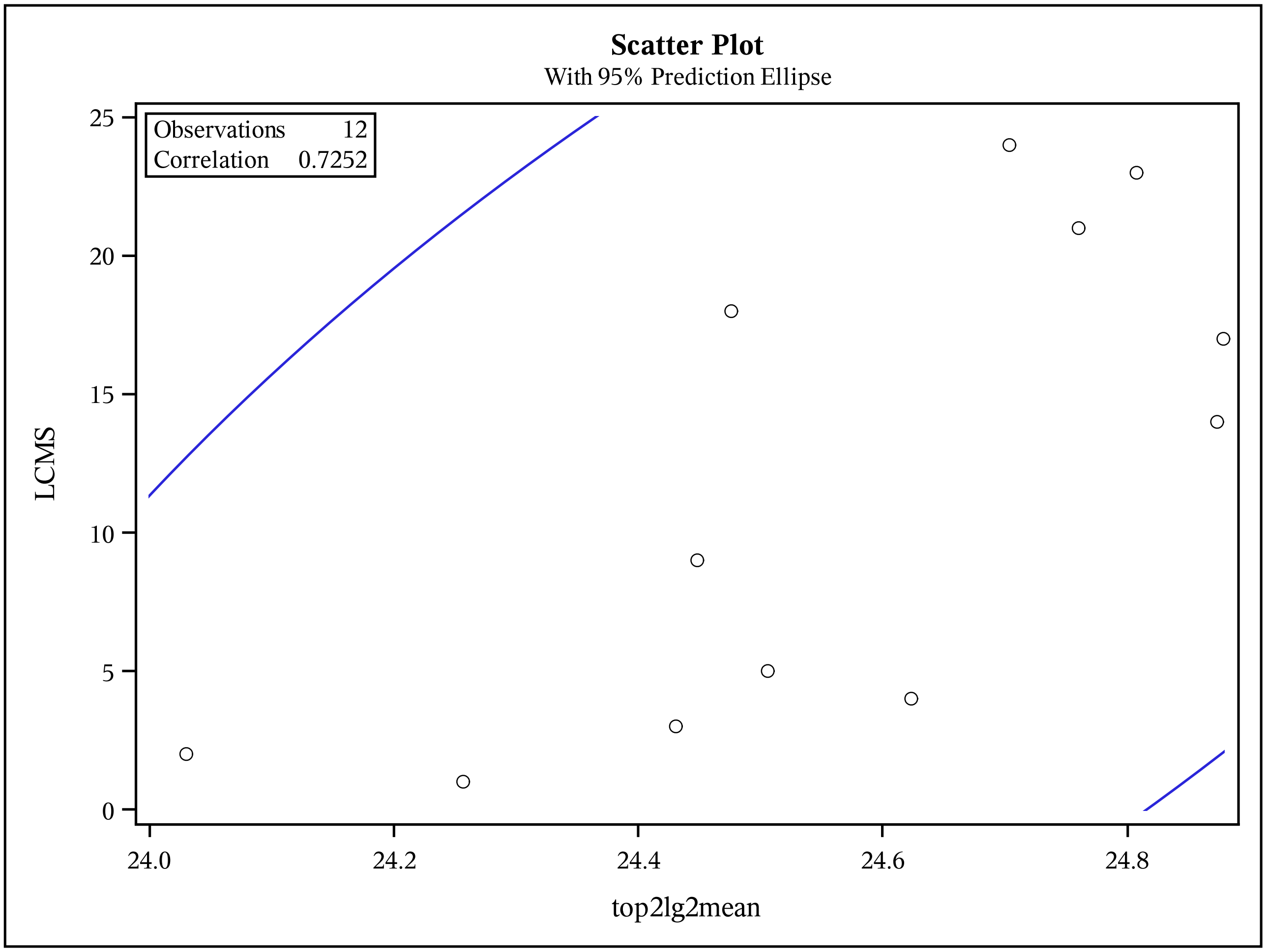

Correlation C_pooltop2lg2mean_tp2 symbol LCMS

The CORR Procedure

Gene_Symbol=CNDP1

2 Variables:	top2lg2mean LCMS

Simple Statistics						
**Variable**	**N**	**Mean**	**Std Dev**	**Sum**	**Minimum**	**Maximum**
**top2lg2mean**	12	22.80093	0.11438	273.61114	22.58143	22.93465
**LCMS**	12	11.75000	8.70867	141.00000	1.00000	24.00000

Pearson Correlation Coefficients, N = 12<br>Prob > |r| under H0: Rho=0		
	**top2lg2mean**	**LCMS**
**top2lg2mean**	1.00000	0.27949<br>0.3790
**LCMS**	0.27949<br>0.3790	1.00000

Pearson Correlation Statistics (Fisher's z Transformation)									
**Variable**	**With Variable**	**N**	**Sample Correlation**	**Fisher's z**	**Bias Adjustment**	**Correlation Estimate**	**95% Confidence Limits**		**p Value for H0:Rho=0**
**top2lg2mean**	**LCMS**	12	0.27949	0.28712	0.01270	0.26773	-0.361753	0.729539	0.3890

Correlation C_pooltop2lg2mean_tp2 symbol LCMS

The CORR Procedure

Gene_Symbol=CNDP1

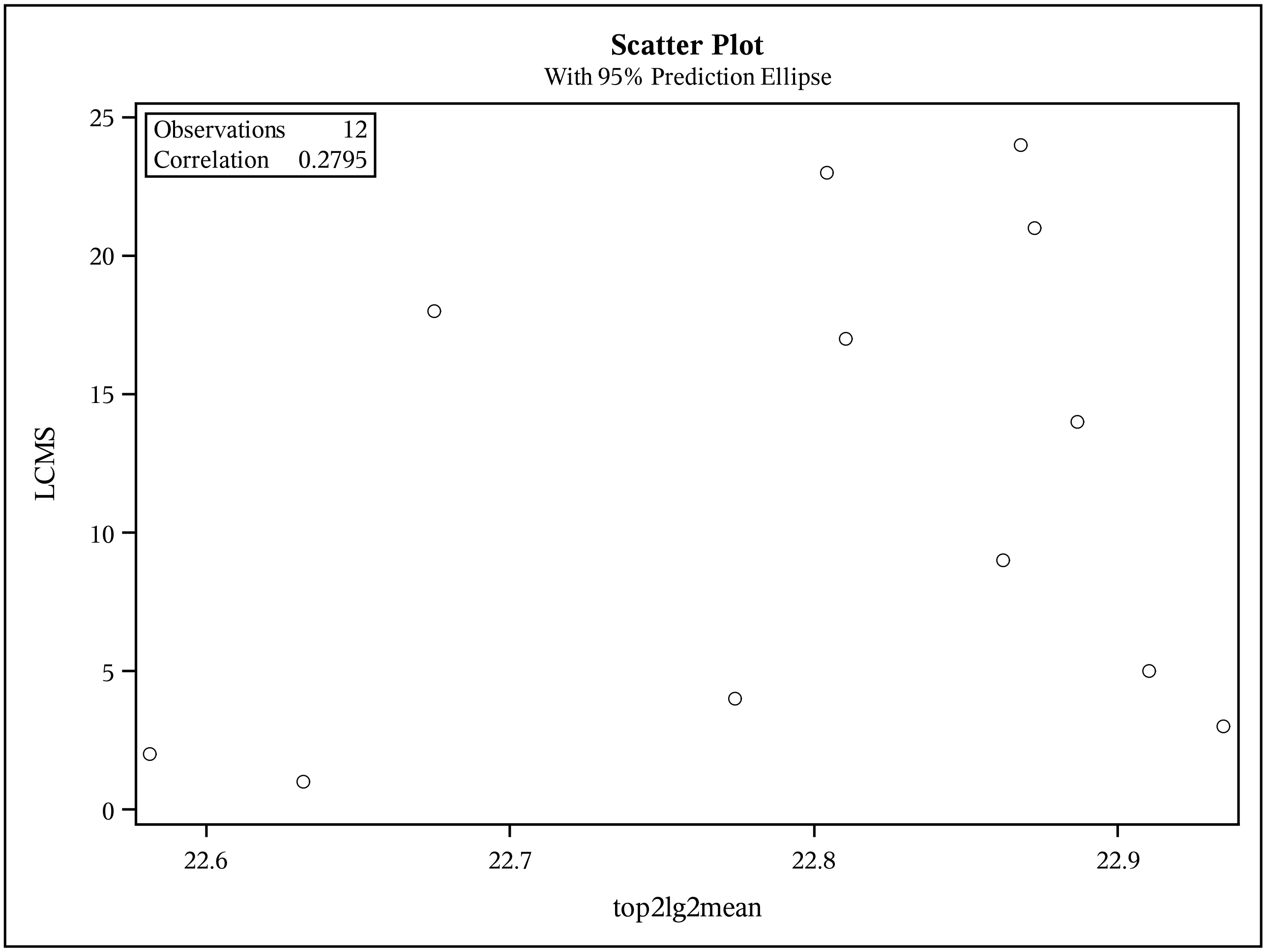

Correlation C_pooltop2lg2mean_tp2 symbol LCMS

The CORR Procedure

Gene_Symbol=CNTN1

2 Variables:	top2lg2mean LCMS

Simple Statistics						
Variable	N	Mean	Std Dev	Sum	Minimum	Maximum
top2lg2mean	12	20.00227	0.16488	240.02726	19.59967	20.23726
LCMS	12	11.75000	8.70867	141.00000	1.00000	24.00000

Pearson Correlation Coefficients, N = 12<br>Prob > |r| under H0: Rho=0		
	top2lg2mean	LCMS
top2lg2mean	1.00000	0.49263<br>0.1037
LCMS	0.49263<br>0.1037	1.00000

Pearson Correlation Statistics (Fisher's z Transformation)									
Variable	With Variable	N	Sample Correlation	Fisher's z	Bias Adjustment	Correlation Estimate	95% Confidence Limits		p Value for H0:Rho=0
top2lg2mean	LCMS	12	0.49263	0.53952	0.02239	0.47548	-0.135356	0.824417	0.1055

Correlation C_pooltop2lg2mean_tp2 symbol LCMS

The CORR Procedure

Gene_Symbol=CNTN1

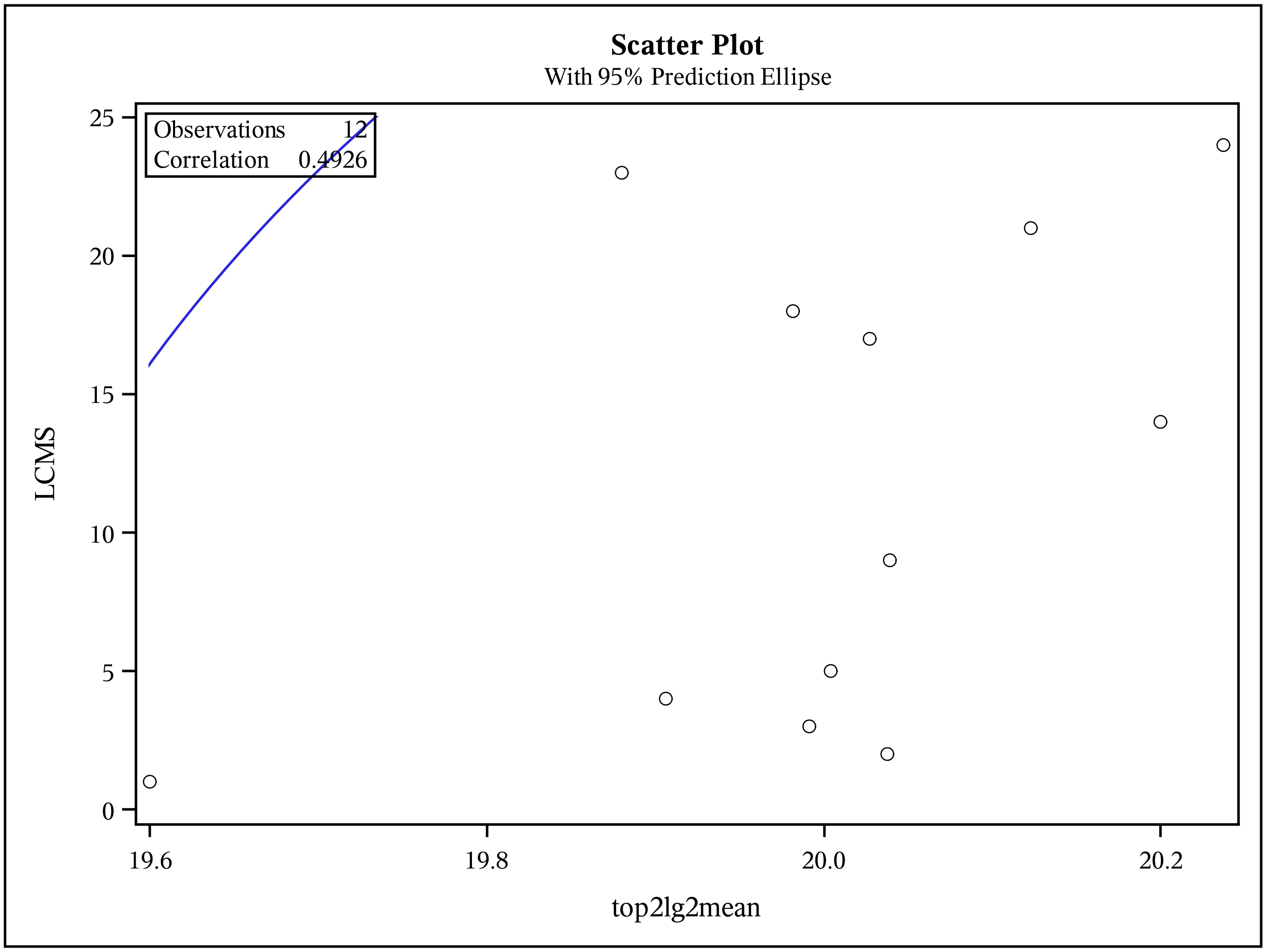

Correlation C_pooltop2lg2mean_tp2 symbol LCMS

The CORR Procedure

Gene_Symbol=CP

2 Variables:	top2lg2mean LCMS

Simple Statistics						
Variable	N	Mean	Std Dev	Sum	Minimum	Maximum
top2lg2mean	12	22.55661	0.31053	270.67936	22.02298	23.05525
LCMS	12	11.75000	8.70867	141.00000	1.00000	24.00000

Pearson Correlation Coefficients, N = 12<br>Prob > |r| under H0: Rho=0		
	top2lg2mean	LCMS
top2lg2mean	1.00000	-0.29225<br>0.3567
LCMS	-0.29225<br>0.3567	1.00000

Pearson Correlation Statistics (Fisher's z Transformation)									
Variable	With Variable	N	Sample Correlation	Fisher's z	Bias Adjustment	Correlation Estimate	95% Confidence Limits		p Value for H0:Rho=0
top2lg2mean	LCMS	12	-0.29225	-0.30102	-0.01328	-0.28005	-0.735709	0.350122	0.3665

Correlation C_pooltop2lg2mean_tp2 symbol LCMS

The CORR Procedure

Gene_Symbol=CP

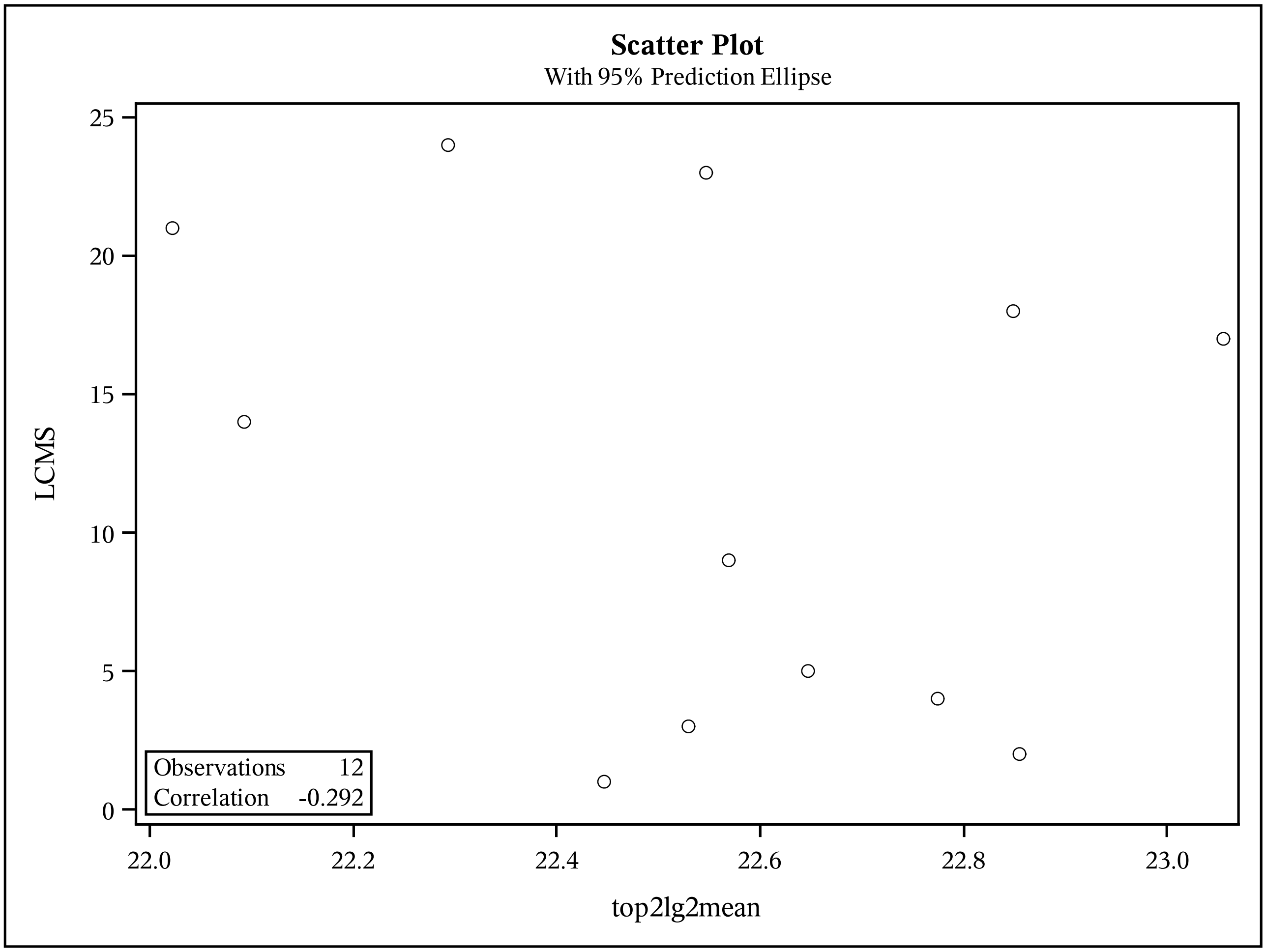

Correlation C_pooltop2lg2mean_tp2 symbol LCMS

The CORR Procedure

Gene_Symbol=CPA1

2 Variables:	top2lg2mean LCMS

Simple Statistics						
Variable	N	Mean	Std Dev	Sum	Minimum	Maximum
top2lg2mean	12	16.97277	0.37952	203.67329	16.44452	17.86625
LCMS	12	11.75000	8.70867	141.00000	1.00000	24.00000

Pearson Correlation Coefficients, N = 12<br>Prob > |r| under H0: Rho=0		
	top2lg2mean	LCMS
top2lg2mean	1.00000	-0.53262<br>0.0746
LCMS	-0.53262<br>0.0746	1.00000

Pearson Correlation Statistics (Fisher's z Transformation)									
Variable	With Variable	N	Sample Correlation	Fisher's z	Bias Adjustment	Correlation Estimate	95% Confidence Limits		p Value for H0:Rho=0
top2lg2mean	LCMS	12	-0.53262	-0.59380	-0.02421	-0.51506	-0.840510	0.083540	0.0748

Correlation C_pooltop2lg2mean_tp2 symbol LCMS

The CORR Procedure

Gene_Symbol=CPA1

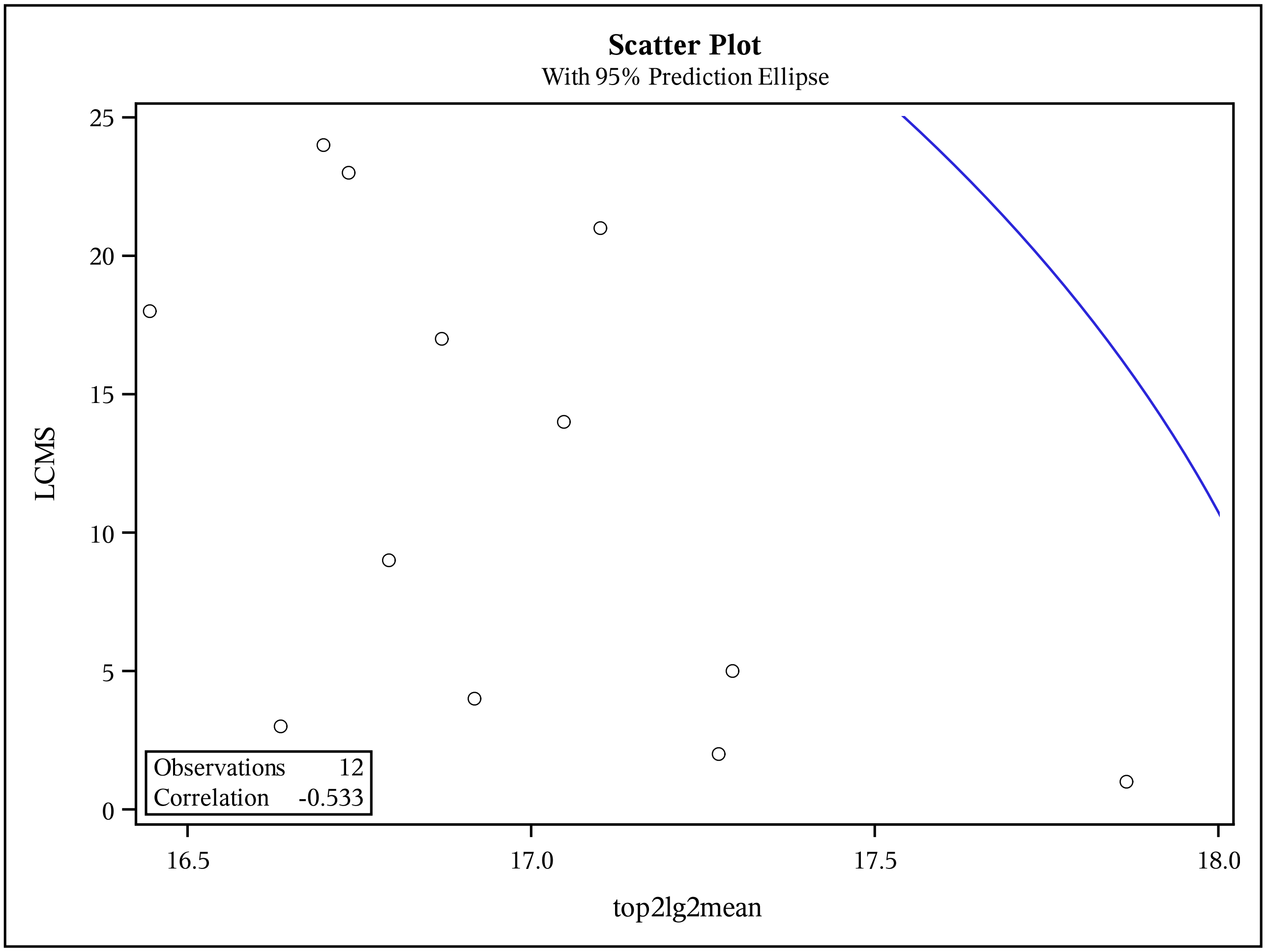

Correlation C_pooltop2lg2mean_tp2 symbol LCMS

The CORR Procedure

Gene_Symbol=CPE

2 Variables:	top2lg2mean LCMS

Simple Statistics						
Variable	N	Mean	Std Dev	Sum	Minimum	Maximum
top2lg2mean	12	17.11630	0.28200	205.39565	16.67208	17.43015
LCMS	12	11.75000	8.70867	141.00000	1.00000	24.00000

Pearson Correlation Coefficients, N = 12<br>Prob > |r| under H0: Rho=0		
	top2lg2mean	LCMS
top2lg2mean	1.00000	0.09411<br>0.7711
LCMS	0.09411<br>0.7711	1.00000

Pearson Correlation Statistics (Fisher's z Transformation)									
Variable	With Variable	N	Sample Correlation	Fisher's z	Bias Adjustment	Correlation Estimate	95% Confidence Limits		p Value for H0:Rho=0
top2lg2mean	LCMS	12	0.09411	0.09439	0.00428	0.08987	-0.510357	0.631214	0.7771

Correlation C_pooltop2lg2mean_tp2 symbol LCMS

The CORR Procedure

Gene_Symbol=CPE

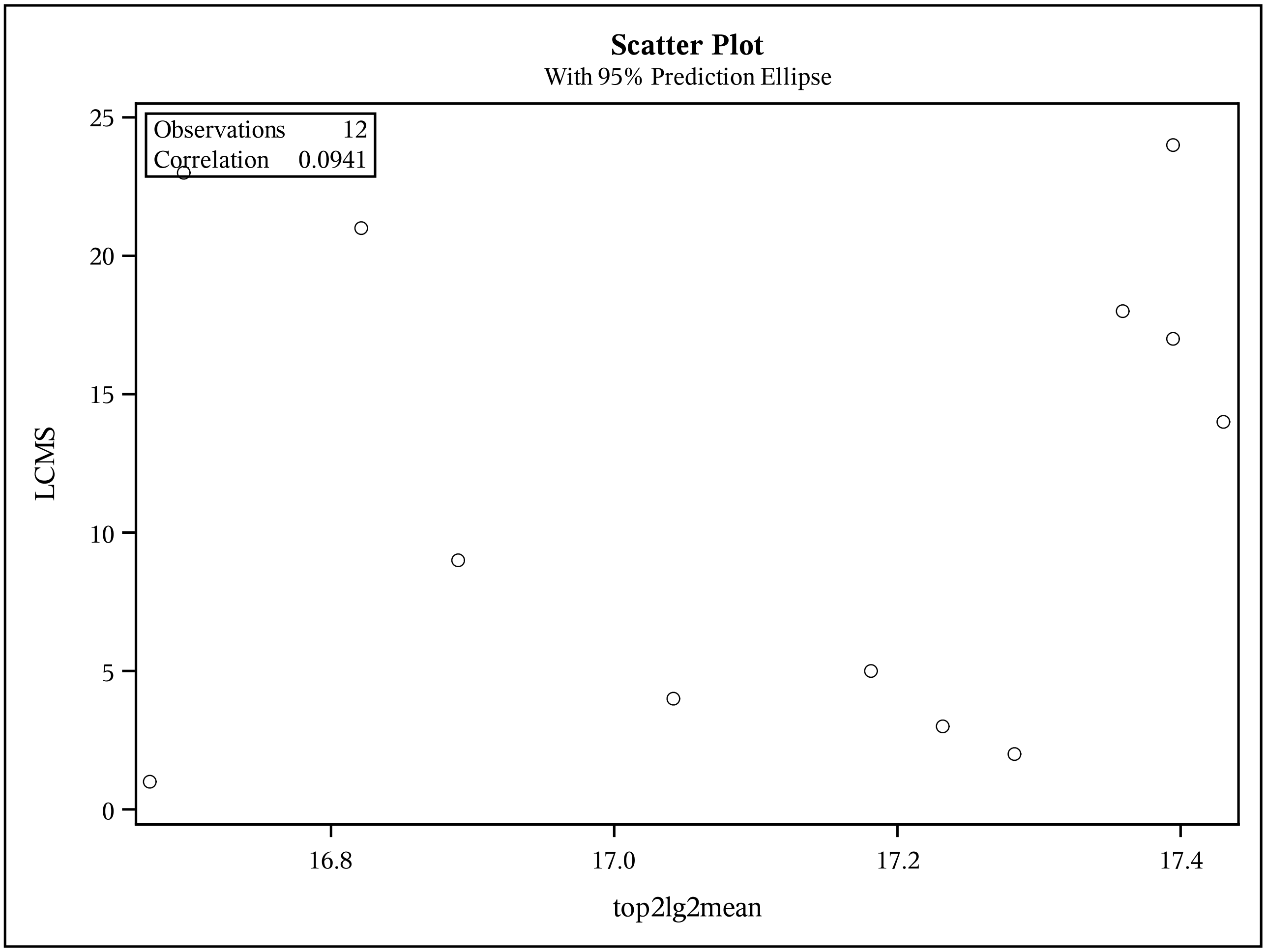

Correlation C_pooltop2lg2mean_tp2 symbol LCMS

The CORR Procedure

Gene_Symbol=CRTAC1

**2 Variables:**	top2lg2mean LCMS

Simple Statistics						
**Variable**	**N**	**Mean**	**Std Dev**	**Sum**	**Minimum**	**Maximum**
**top2lg2mean**	12	16.15360	1.94327	193.84315	10.01973	17.06571
**LCMS**	12	11.75000	8.70867	141.00000	1.00000	24.00000

Pearson Correlation Coefficients, N = 12<br>Prob > |r| under H0: Rho=0		
	**top2lg2mean**	**LCMS**
**top2lg2mean**	1.00000	0.28917<br>0.3620
**LCMS**	0.28917<br>0.3620	1.00000

Pearson Correlation Statistics (Fisher's z Transformation)									
**Variable**	**With Variable**	**N**	**Sample Correlation**	**Fisher's z**	**Bias Adjustment**	**Correlation Estimate**	**95% Confidence Limits**		**p Value for H0:Rho=0**
**top2lg2mean**	**LCMS**	12	0.28917	0.29766	0.01314	0.27708	-0.352950	0.734225	0.3719

Correlation C_pooltop2lg2mean_tp2 symbol LCMS

The CORR Procedure

Gene_Symbol=CRTAC1

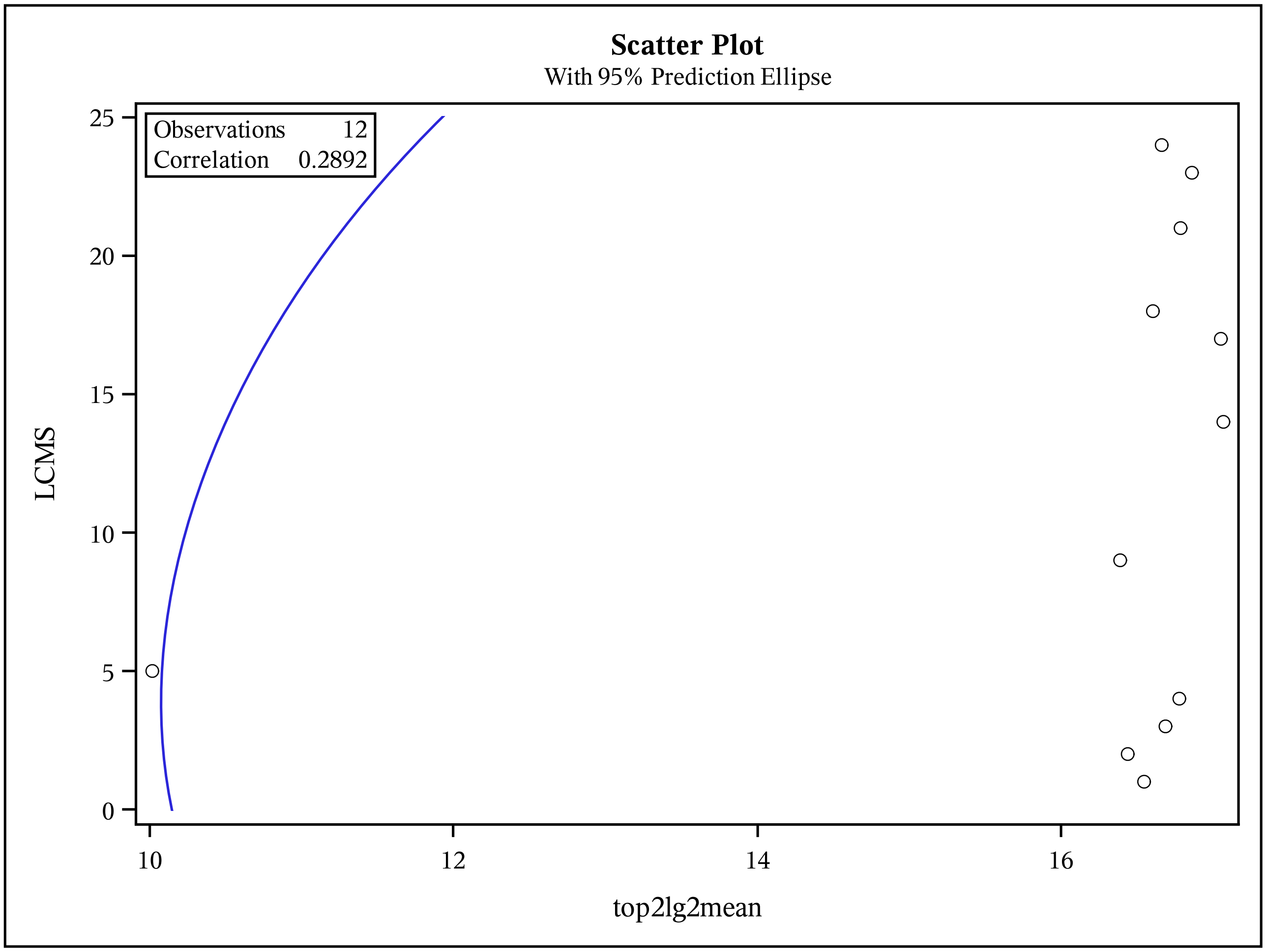

Correlation C_pooltop2lg2mean_tp2 symbol LCMS

The CORR Procedure

Gene_Symbol=CST3

**2 Variables:**	top2lg2mean LCMS

Simple Statistics						
**Variable**	**N**	**Mean**	**Std Dev**	**Sum**	**Minimum**	**Maximum**
**top2lg2mean**	12	25.23753	0.16033	302.85033	24.95270	25.56438
**LCMS**	12	11.75000	8.70867	141.00000	1.00000	24.00000

Pearson Correlation Coefficients, N = 12<br>Prob > |r| under H0: Rho=0		
	**top2lg2mean**	**LCMS**
**top2lg2mean**	1.00000	0.28532<br>0.3687
**LCMS**	0.28532<br>0.3687	1.00000

Pearson Correlation Statistics (Fisher's z Transformation)									
**Variable**	**With Variable**	**N**	**Sample Correlation**	**Fisher's z**	**Bias Adjustment**	**Correlation Estimate**	**95% Confidence Limits**		**p Value for H0:Rho=0**
**top2lg2mean**	**LCMS**	12	0.28532	0.29346	0.01297	0.27336	-0.356462	0.732368	0.3786

Correlation C_pooltop2lg2mean_tp2 symbol LCMS

The CORR Procedure

Gene_Symbol=CST3

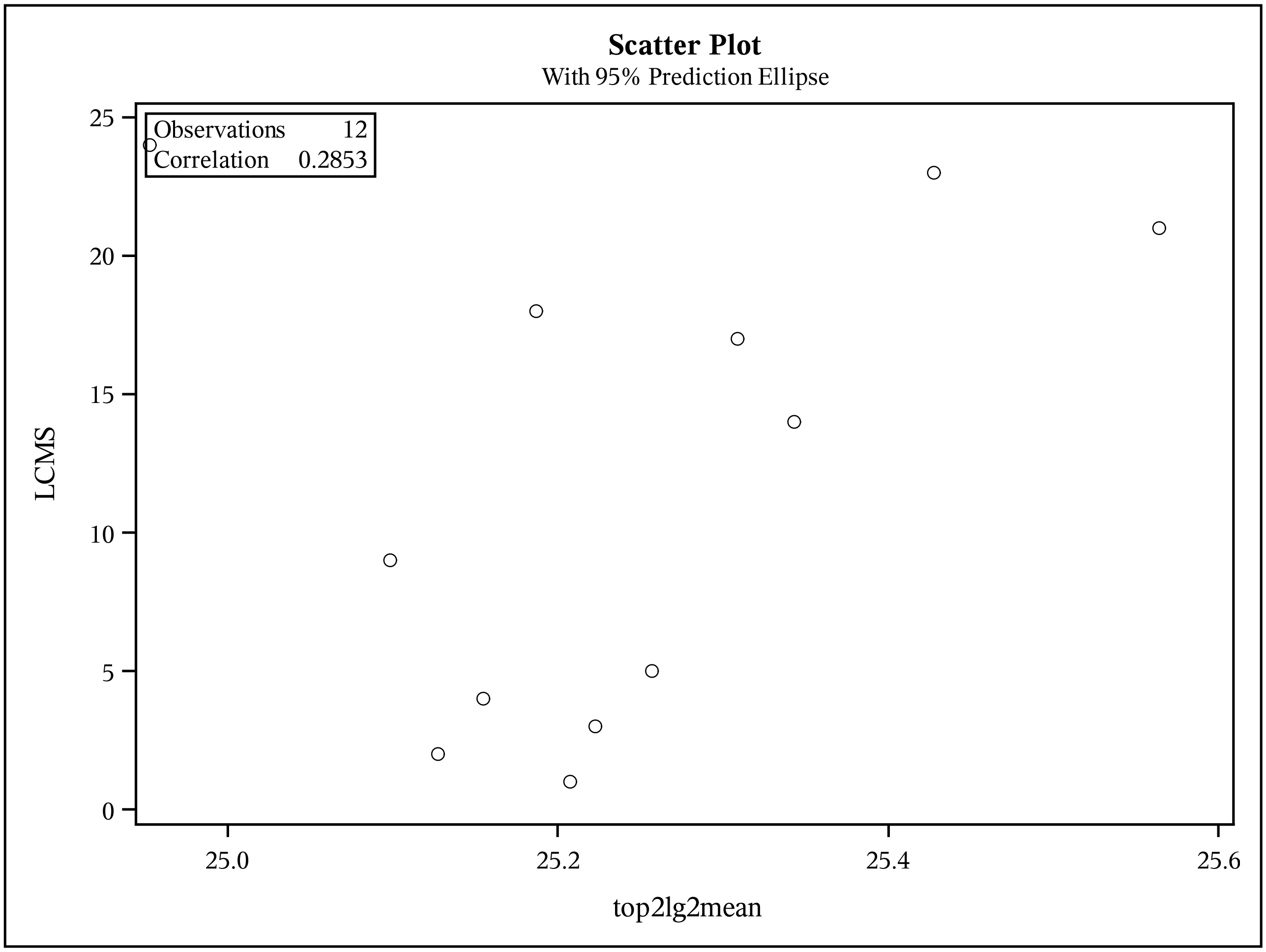

Correlation C_pooltop2lg2mean_tp2 symbol LCMS

The CORR Procedure

Gene_Symbol=DKK3

2 Variables:	top2lg2mean LCMS

Simple Statistics						
Variable	N	Mean	Std Dev	Sum	Minimum	Maximum
top2lg2mean	12	20.10947	0.79286	241.31363	19.13980	21.78206
LCMS	12	11.75000	8.70867	141.00000	1.00000	24.00000

Pearson Correlation Coefficients, N = 12<br>Prob > |r| under H0: Rho=0		
	top2lg2mean	LCMS
top2lg2mean	1.00000	-0.50936<br>0.0908
LCMS	-0.50936<br>0.0908	1.00000

Pearson Correlation Statistics (Fisher's z Transformation)										
Variable	With Variable	N	Sample Correlation	Fisher's z	Bias Adjustment	Correlation Estimate	95% Confidence Limits		p Value for H0:Rho=0
top2lg2mean	LCMS	12	-0.50936	-0.56186	-0.02315	-0.49201	-0.831207	0.114116	0.0919

Correlation C_pooltop2lg2mean_tp2 symbol LCMS

The CORR Procedure

Gene_Symbol=DKK3

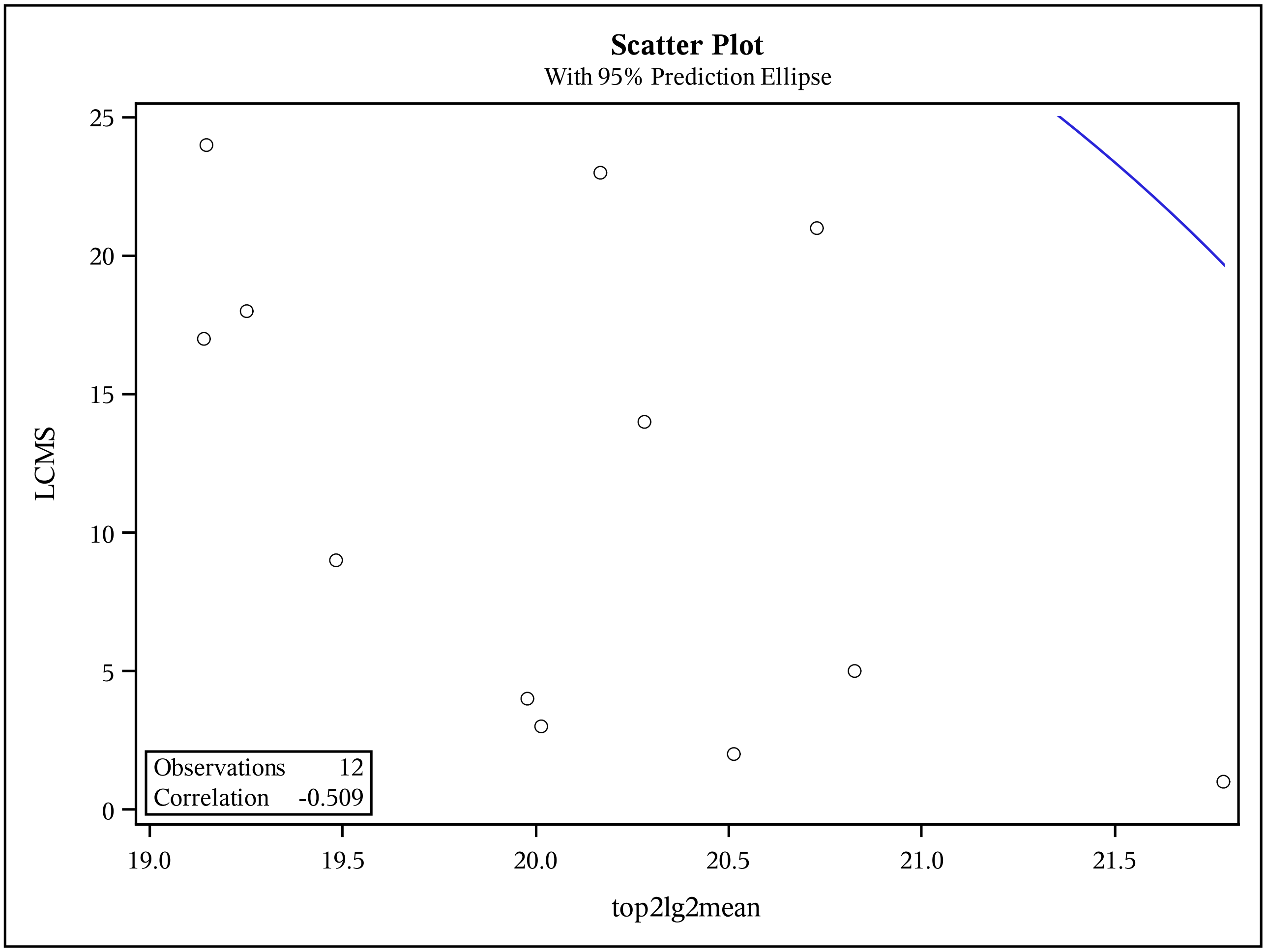

Correlation C_pooltop2lg2mean_tp2 symbol LCMS

The CORR Procedure

Gene_Symbol=EFEMP1

2 Variables:	top2lg2mean LCMS

Simple Statistics						
Variable	N	Mean	Std Dev	Sum	Minimum	Maximum
top2lg2mean	12	20.60120	0.23123	247.21445	20.22609	21.07743
LCMS	12	11.75000	8.70867	141.00000	1.00000	24.00000

Pearson Correlation Coefficients, N = 12<br>Prob > |r| under H0: Rho=0		
	top2lg2mean	LCMS
top2lg2mean	1.00000	-0.16093<br>0.6173
LCMS	-0.16093<br>0.6173	1.00000

Pearson Correlation Statistics (Fisher's z Transformation)									
Variable	With Variable	N	Sample Correlation	Fisher's z	Bias Adjustment	Correlation Estimate	95% Confidence Limits		p Value for H0:Rho=0
top2lg2mean	LCMS	12	-0.16093	-0.16235	-0.00732	-0.15380	-0.668680	0.460771	0.6262

Correlation C_pooltop2lg2mean_tp2 symbol LCMS

The CORR Procedure

Gene_Symbol=EFEMP1

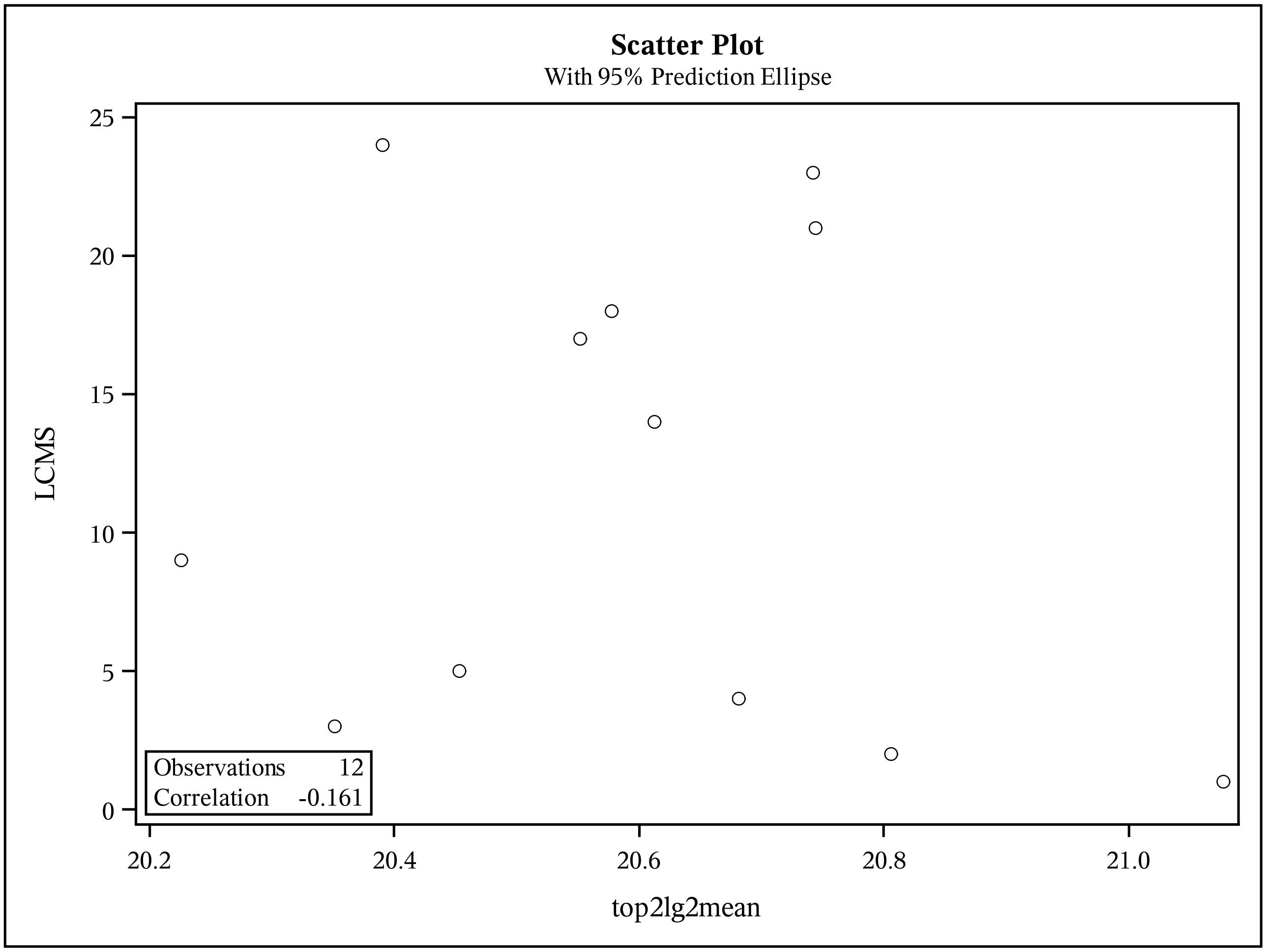

Correlation C_pooltop2lg2mean_tp2 symbol LCMS

The CORR Procedure

Gene_Symbol=ENPP2

2 Variables:	top2lg2mean LCMS

Simple Statistics						
Variable	N	Mean	Std Dev	Sum	Minimum	Maximum
top2lg2mean	12	20.07433	0.89659	240.89194	18.69225	21.72345
LCMS	12	11.75000	8.70867	141.00000	1.00000	24.00000

Pearson Correlation Coefficients, N = 12<br>Prob > |r| under H0: Rho=0		
	top2lg2mean	LCMS
top2lg2mean	1.00000	-0.22714<br>0.4777
LCMS	-0.22714<br>0.4777	1.00000

Pearson Correlation Statistics (Fisher's z Transformation)									
Variable	With Variable	N	Sample Correlation	Fisher's z	Bias Adjustment	Correlation Estimate	95% Confidence Limits		p Value for H0:Rho=0
top2lg2mean	LCMS	12	-0.22714	-0.23117	-0.01032	-0.21732	-0.703485	0.407389	0.4880

Correlation C_pooltop2lg2mean_tp2 symbol LCMS

The CORR Procedure

Gene_Symbol=ENPP2

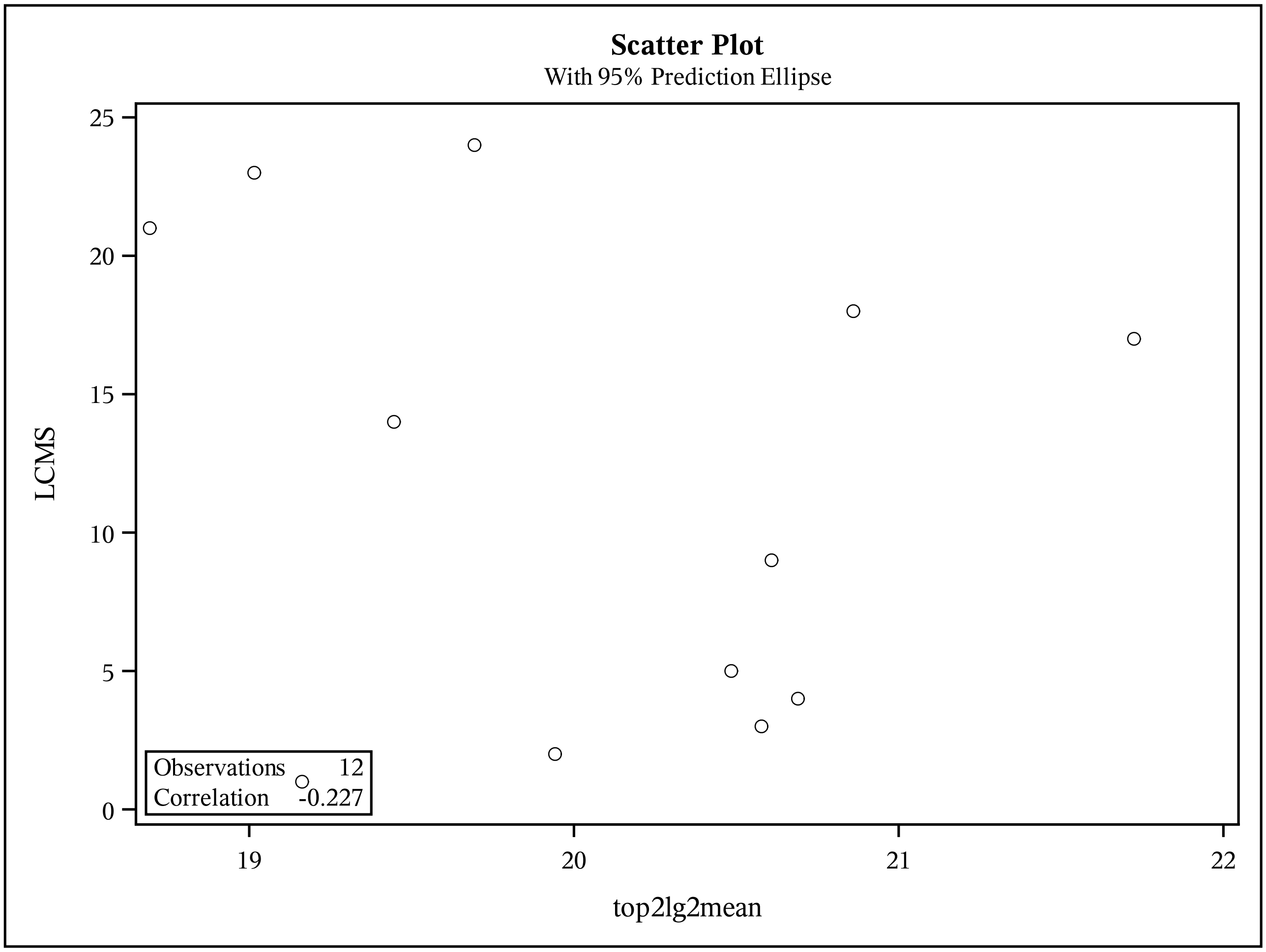

Correlation C_pooltop2lg2mean_tp2 symbol LCMS

The CORR Procedure

Gene_Symbol=F2

2 Variables:	top2lg2mean LCMS

Simple Statistics						
**Variable**	**N**	**Mean**	**Std Dev**	**Sum**	**Minimum**	**Maximum**
**top2lg2mean**	12	19.93872	0.47286	239.26468	19.14113	20.64381
**LCMS**	12	11.75000	8.70867	141.00000	1.00000	24.00000

Pearson Correlation Coefficients, N = 12<br>Prob > |r| under H0: Rho=0		
	**top2lg2mean**	**LCMS**
**top2lg2mean**	1.00000	0.37395<br>0.2311
**LCMS**	0.37395<br>0.2311	1.00000

Pearson Correlation Statistics (Fisher's z Transformation)									
**Variable**	**With Variable**	**N**	**Sample Correlation**	**Fisher's z**	**Bias Adjustment**	**Correlation Estimate**	**95% Confidence Limits**		**p Value for H0:Rho=0**
**top2lg2mean**	**LCMS**	12	0.37395	0.39301	0.01700	0.35924	-0.270412	0.773641	0.2384

Correlation C_pooltop2lg2mean_tp2 symbol LCMS

The CORR Procedure

Gene_Symbol=F2

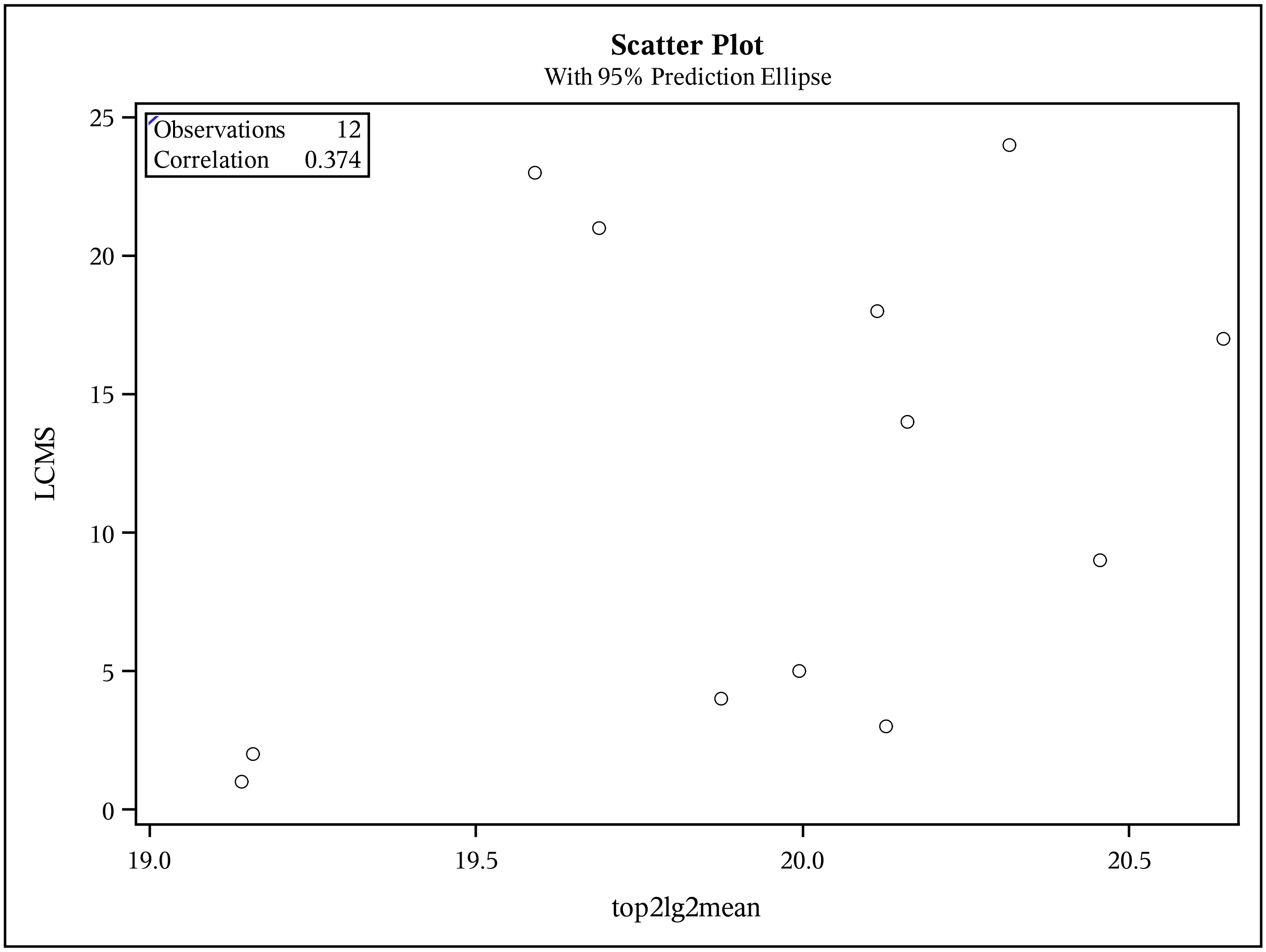

Correlation C_pooltop2lg2mean_tp2 symbol LCMS

The CORR Procedure

Gene_Symbol=FBLN1

2 Variables:	top2lg2mean LCMS

Simple Statistics						
**Variable**	**N**	**Mean**	**Std Dev**	**Sum**	**Minimum**	**Maximum**
**top2lg2mean**	12	21.28993	0.21031	255.47919	20.90053	21.60484
**LCMS**	12	11.75000	8.70867	141.00000	1.00000	24.00000

Pearson Correlation Coefficients, N = 12<br>Prob > |r| under H0: Rho=0		
	**top2lg2mean**	**LCMS**
**top2lg2mean**	1.00000	0.69212<br>0.0126
**LCMS**	0.69212<br>0.0126	1.00000

Pearson Correlation Statistics (Fisher's z Transformation)									
**Variable**	**With Variable**	**N**	**Sample Correlation**	**Fisher's z**	**Bias Adjustment**	**Correlation Estimate**	**95% Confidence Limits**		**p Value for H0:Rho=0**
**top2lg2mean**	**LCMS**	12	0.69212	0.85202	0.03146	0.67538	0.165699	0.900316	0.0106

Correlation C_pooltop2lg2mean_tp2 symbol LCMS

The CORR Procedure

Gene_Symbol=FBLN1

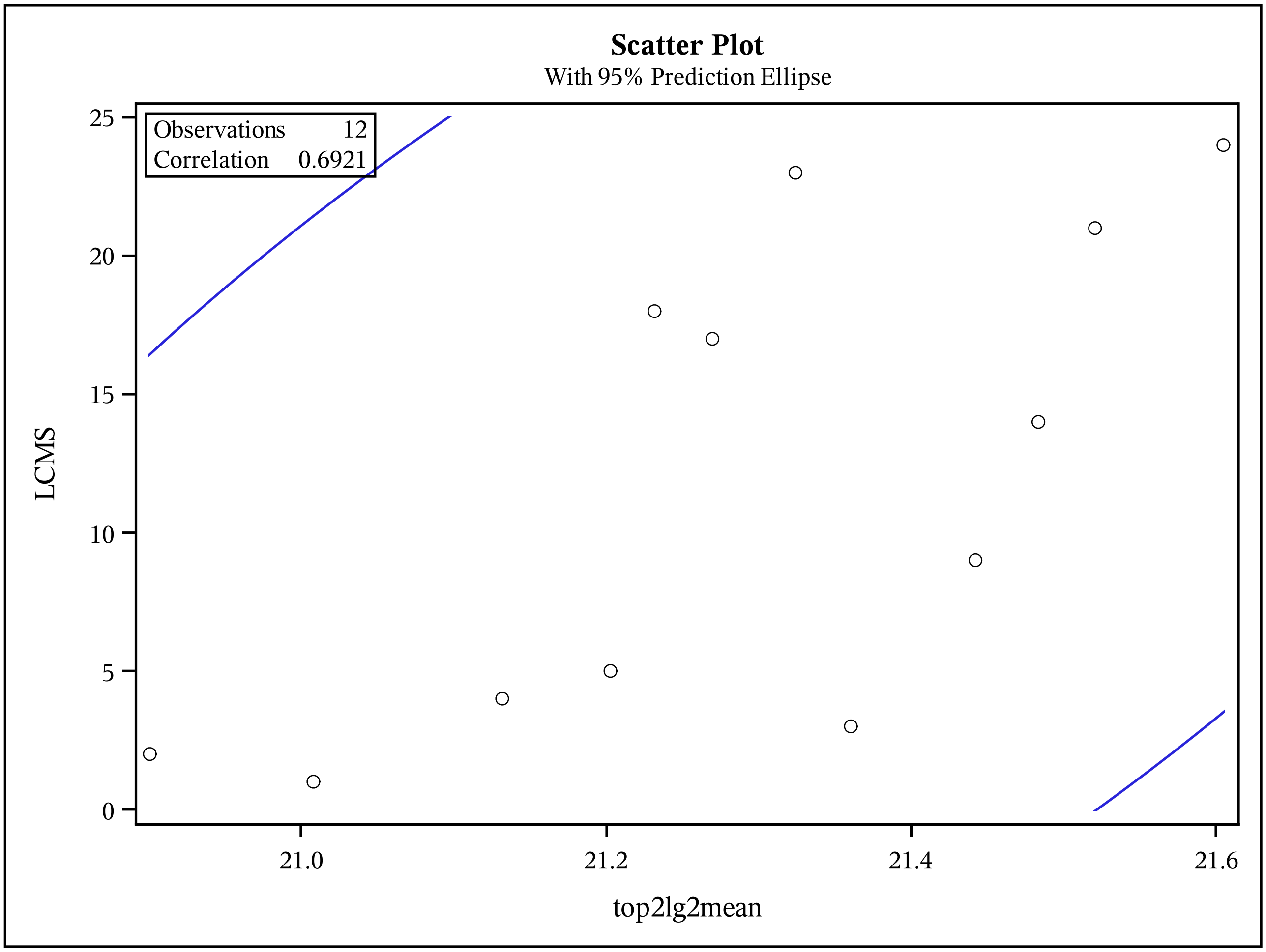

Correlation C_pooltop2lg2mean_tp2 symbol LCMS

The CORR Procedure

Gene_Symbol=FGG

2 Variables:	top2lg2mean LCMS

Simple Statistics						
Variable	N	Mean	Std Dev	Sum	Minimum	Maximum
top2lg2mean	12	17.97301	0.39551	215.67613	17.49951	19.09702
LCMS	12	11.75000	8.70867	141.00000	1.00000	24.00000

Pearson Correlation Coefficients, N = 12<br>Prob > |r| under H0: Rho=0		
	top2lg2mean	LCMS
top2lg2mean	1.00000	0.02584<br>0.9365
LCMS	0.02584<br>0.9365	1.00000

Pearson Correlation Statistics (Fisher's z Transformation)									
Variable	With Variable	N	Sample Correlation	Fisher's z	Bias Adjustment	Correlation Estimate	95% Confidence Limits		p Value for H0:Rho=0
top2lg2mean	LCMS	12	0.02584	0.02585	0.00117	0.02467	-0.557122	0.590213	0.9382

Correlation C_pooltop2lg2mean_tp2 symbol LCMS

The CORR Procedure

Gene_Symbol=FGG

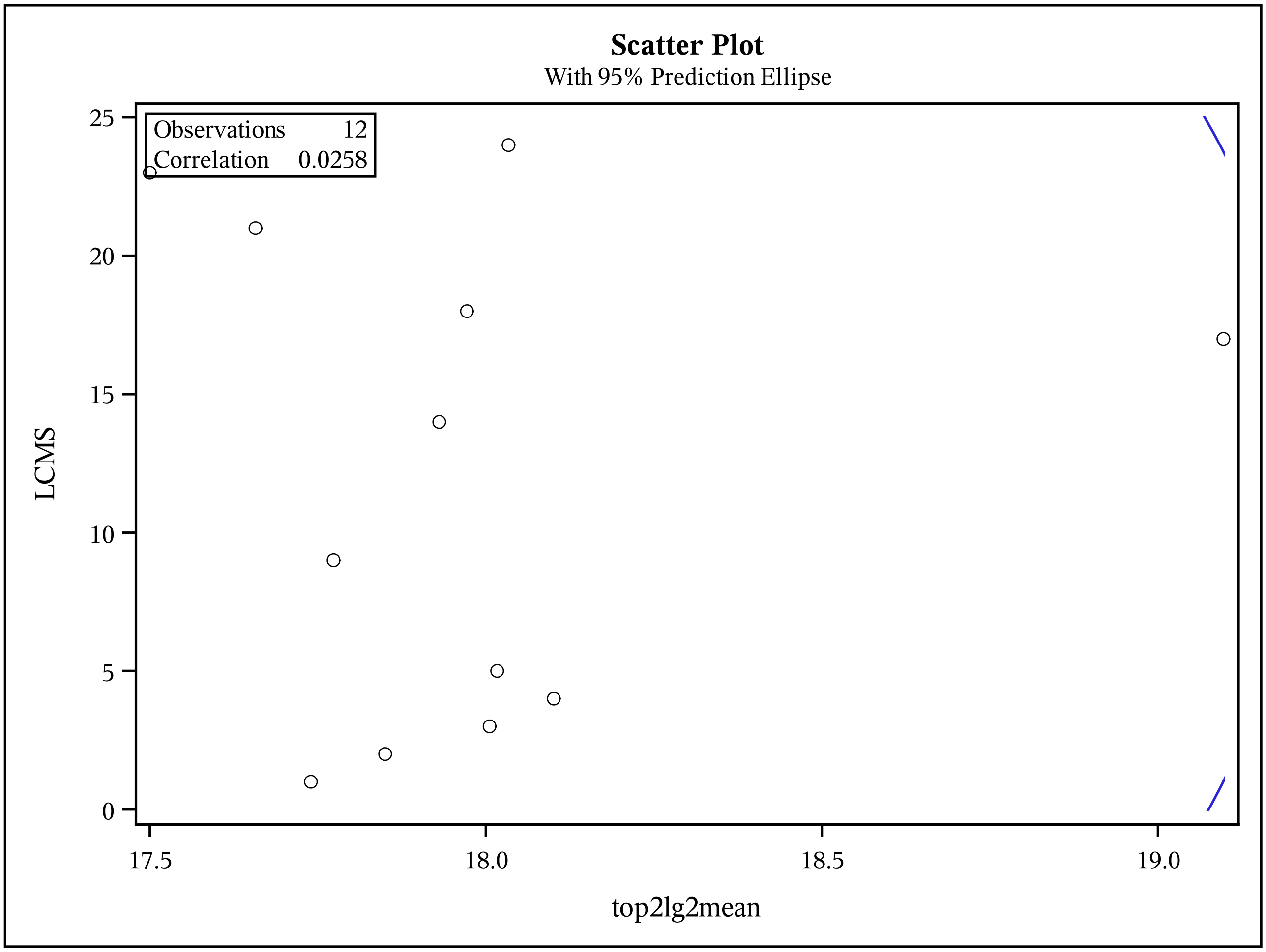

Correlation C_pooltop2lg2mean_tp2 symbol LCMS

The CORR Procedure

Gene_Symbol=FN1

**2 Variables:**	top2lg2mean LCMS

Simple Statistics						
**Variable**	**N**	**Mean**	**Std Dev**	**Sum**	**Minimum**	**Maximum**
**top2lg2mean**	12	20.22242	0.38957	242.66899	19.48613	20.90945
**LCMS**	12	11.75000	8.70867	141.00000	1.00000	24.00000

Pearson Correlation Coefficients, N = 12<br>Prob > |r| under H0: Rho=0		
	**top2lg2mean**	**LCMS**
**top2lg2mean**	1.00000	0.15107<br>0.6393
**LCMS**	0.15107<br>0.6393	1.00000

Pearson Correlation Statistics (Fisher's z Transformation)									
**Variable**	**With Variable**	**N**	**Sample Correlation**	**Fisher's z**	**Bias Adjustment**	**Correlation Estimate**	**95% Confidence Limits**		**p Value for H0:Rho=0**
**top2lg2mean**	**LCMS**	12	0.15107	0.15223	0.00687	0.14435	-0.468349	0.663303	0.6479

Correlation C_pooltop2lg2mean_tp2 symbol LCMS

The CORR Procedure

Gene_Symbol=FN1

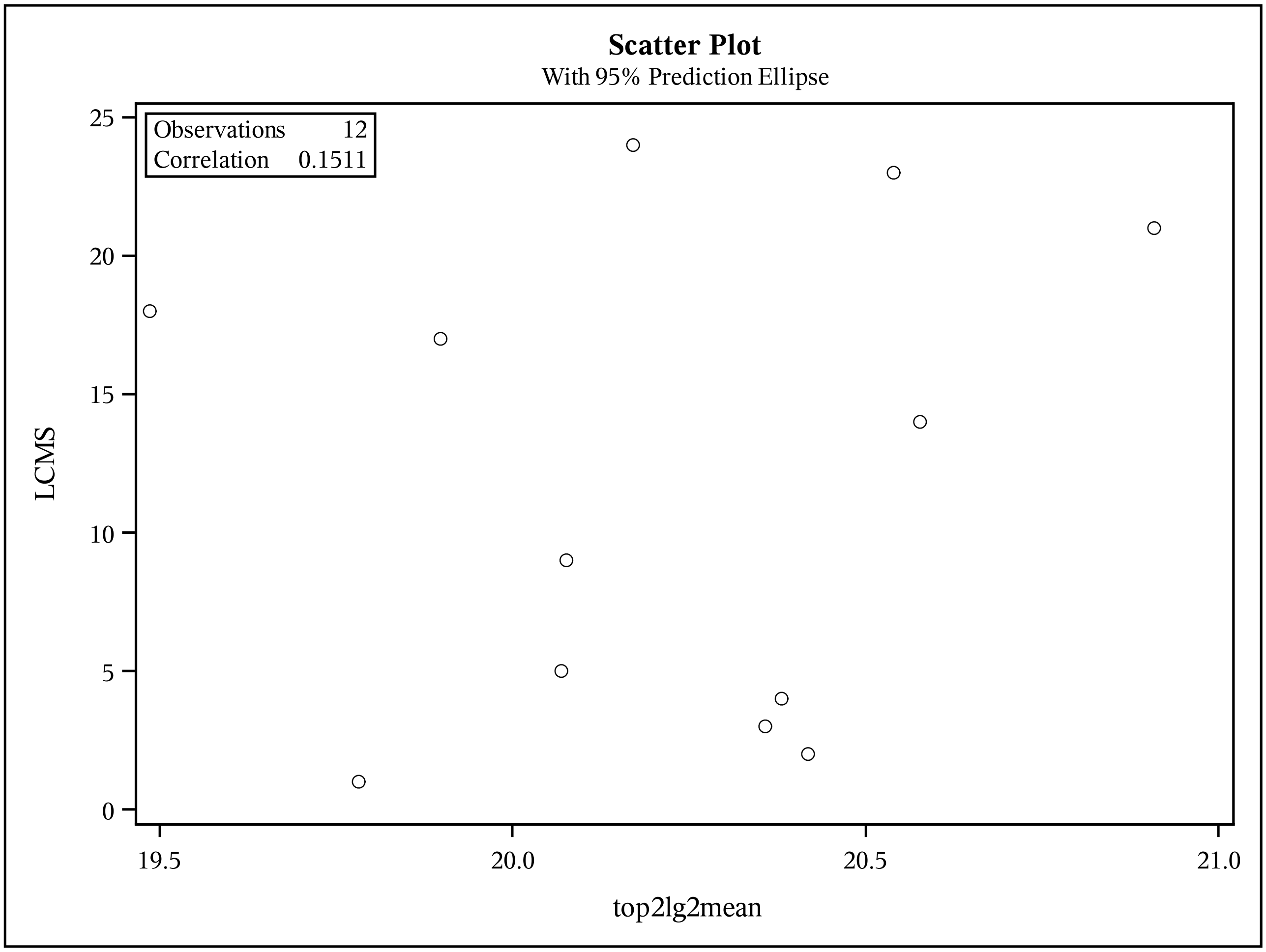

Correlation C_pooltop2lg2mean_tp2 symbol LCMS

The CORR Procedure

Gene_Symbol=GC

2 Variables:	top2lg2mean LCMS

Simple Statistics						
**Variable**	**N**	**Mean**	**Std Dev**	**Sum**	**Minimum**	**Maximum**
**top2lg2mean**	12	23.33209	0.20100	279.98510	22.82401	23.60913
**LCMS**	12	11.75000	8.70867	141.00000	1.00000	24.00000

Pearson Correlation Coefficients, N = 12<br>Prob > |r| under H0: Rho=0		
	**top2lg2mean**	**LCMS**
**top2lg2mean**	1.00000	0.68711<br>0.0136
**LCMS**	0.68711<br>0.0136	1.00000

Pearson Correlation Statistics (Fisher's z Transformation)									
**Variable**	**With Variable**	**N**	**Sample Correlation**	**Fisher's z**	**Bias Adjustment**	**Correlation Estimate**	**95% Confidence Limits**		**p Value for H0:Rho=0**
**top2lg2mean**	**LCMS**	12	0.68711	0.84246	0.03123	0.67027	0.156604	0.898532	0.0115

Correlation C_pooltop2lg2mean_tp2 symbol LCMS

The CORR Procedure

Gene_Symbol=GC

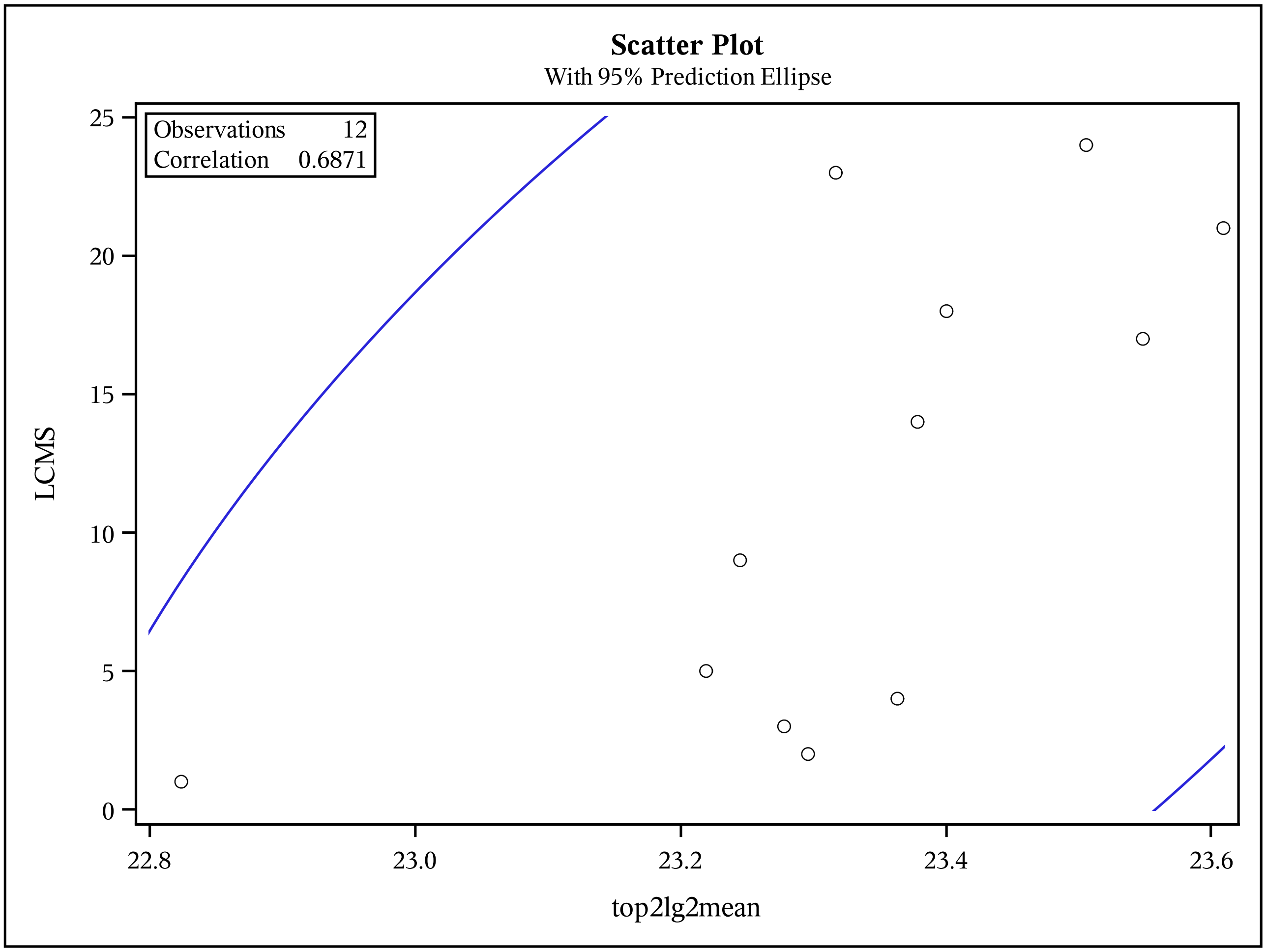

Correlation C_pooltop2lg2mean_tp2 symbol LCMS

The CORR Procedure

Gene_Symbol=GSN

2 Variables:	top2lg2mean LCMS

Simple Statistics						
**Variable**	**N**	**Mean**	**Std Dev**	**Sum**	**Minimum**	**Maximum**
**top2lg2mean**	12	21.67081	0.34197	260.04974	20.77562	21.98326
**LCMS**	12	11.75000	8.70867	141.00000	1.00000	24.00000

Pearson Correlation Coefficients, N = 12<br>Prob > |r| under H0: Rho=0		
	**top2lg2mean**	**LCMS**
**top2lg2mean**	1.00000	0.43011<br>0.1628
**LCMS**	0.43011<br>0.1628	1.00000

Pearson Correlation Statistics (Fisher's z Transformation)									
**Variable**	**With Variable**	**N**	**Sample Correlation**	**Fisher's z**	**Bias Adjustment**	**Correlation Estimate**	**95% Confidence Limits**		**p Value for H0:Rho=0**
**top2lg2mean**	**LCMS**	12	0.43011	0.46003	0.01955	0.41404	-0.209682	0.798263	0.1676

Correlation C_pooltop2lg2mean_tp2 symbol LCMS

The CORR Procedure

Gene_Symbol=GSN

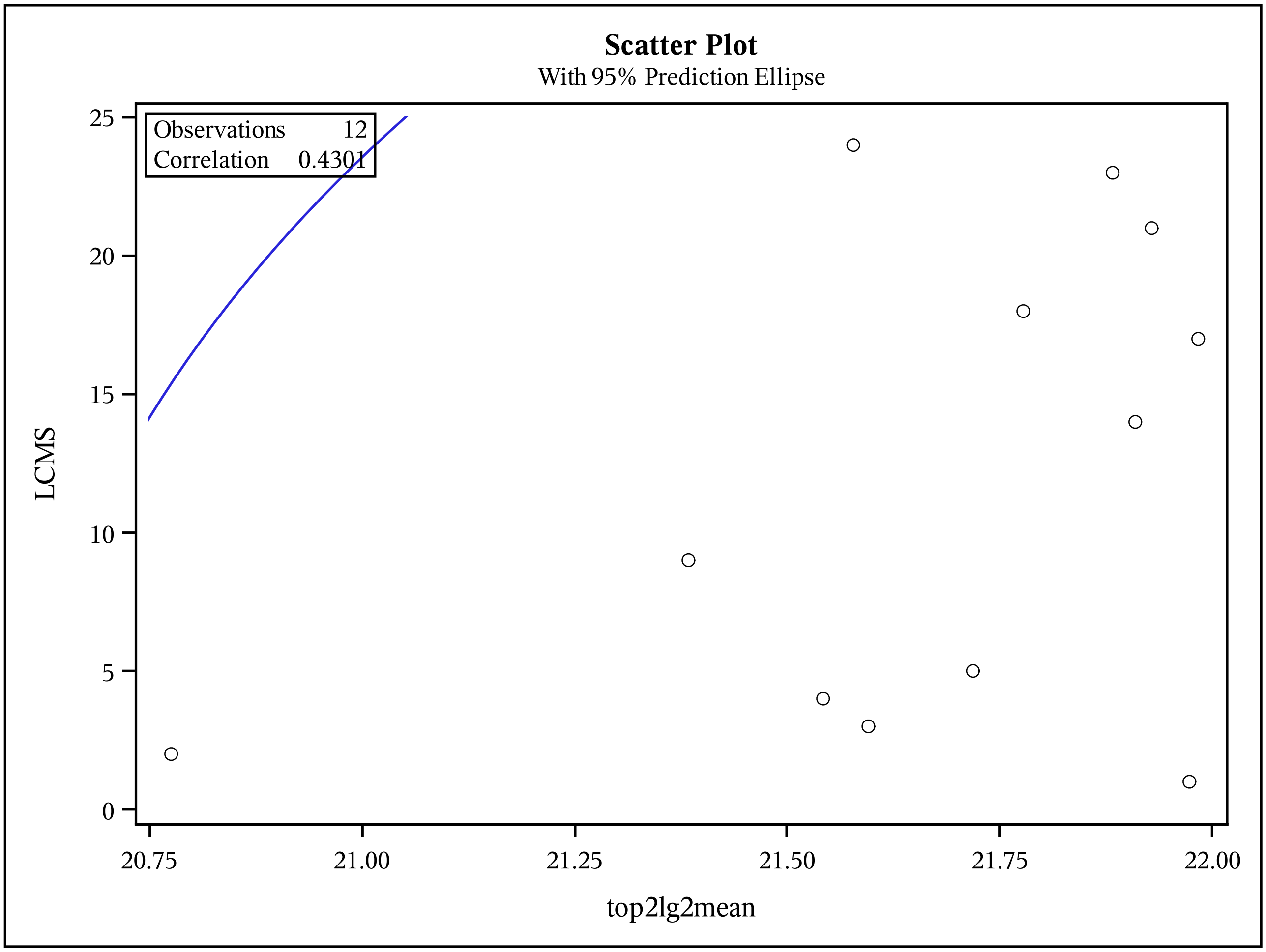

Correlation C_pooltop2lg2mean_tp2 symbol LCMS

The CORR Procedure

Gene_Symbol=HPX

2 Variables:	top2lg2mean LCMS

Simple Statistics						
Variable	N	Mean	Std Dev	Sum	Minimum	Maximum
top2lg2mean	12	25.81965	0.13873	309.83584	25.65299	26.07131
LCMS	12	11.75000	8.70867	141.00000	1.00000	24.00000

Pearson Correlation Coefficients, N = 12<br>Prob > |r| under H0: Rho=0		
	top2lg2mean	LCMS
top2lg2mean	1.00000	0.56920<br>0.0534
LCMS	0.56920<br>0.0534	1.00000

Pearson Correlation Statistics (Fisher's z Transformation)									
Variable	With Variable	N	Sample Correlation	Fisher's z	Bias Adjustment	Correlation Estimate	95% Confidence Limits		p Value for H0:Rho=0
top2lg2mean	LCMS	12	0.56920	0.64633	0.02587	0.55145	-0.032850	0.854819	0.0525

Correlation C_pooltop2lg2mean_tp2 symbol LCMS

The CORR Procedure

Gene_Symbol=HPX

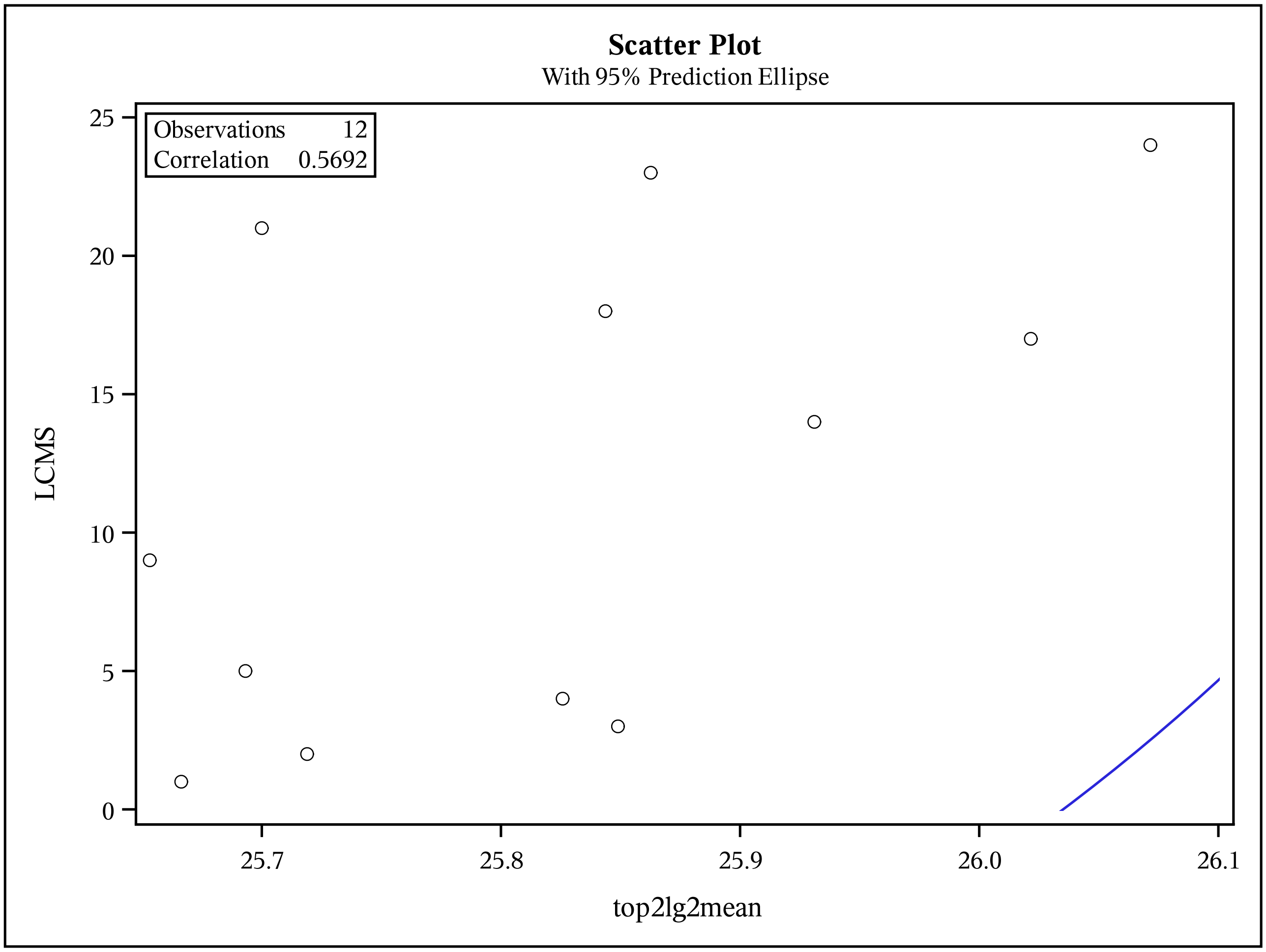

Correlation C_pooltop2lg2mean_tp2 symbol LCMS

The CORR Procedure

Gene_Symbol=IGFBP6

2 Variables:	top2lg2mean LCMS

Simple Statistics						
**Variable**	**N**	**Mean**	**Std Dev**	**Sum**	**Minimum**	**Maximum**
**top2lg2mean**	12	21.76537	0.24356	261.18448	21.06331	22.01586
**LCMS**	12	11.75000	8.70867	141.00000	1.00000	24.00000

Pearson Correlation Coefficients, N = 12<br>Prob > |r| under H0: Rho=0		
	**top2lg2mean**	**LCMS**
**top2lg2mean**	1.00000	0.64233<br>0.0243
**LCMS**	0.64233<br>0.0243	1.00000

Pearson Correlation Statistics (Fisher's z Transformation)									
**Variable**	**With Variable**	**N**	**Sample Correlation**	**Fisher's z**	**Bias Adjustment**	**Correlation Estimate**	**95% Confidence Limits**		**p Value for H0:Rho=0**
**top2lg2mean**	**LCMS**	12	0.64233	0.76214	0.02920	0.62486	0.079450	0.882346	0.0222

Correlation C_pooltop2lg2mean_tp2 symbol LCMS

The CORR Procedure

Gene_Symbol=IGFBP6

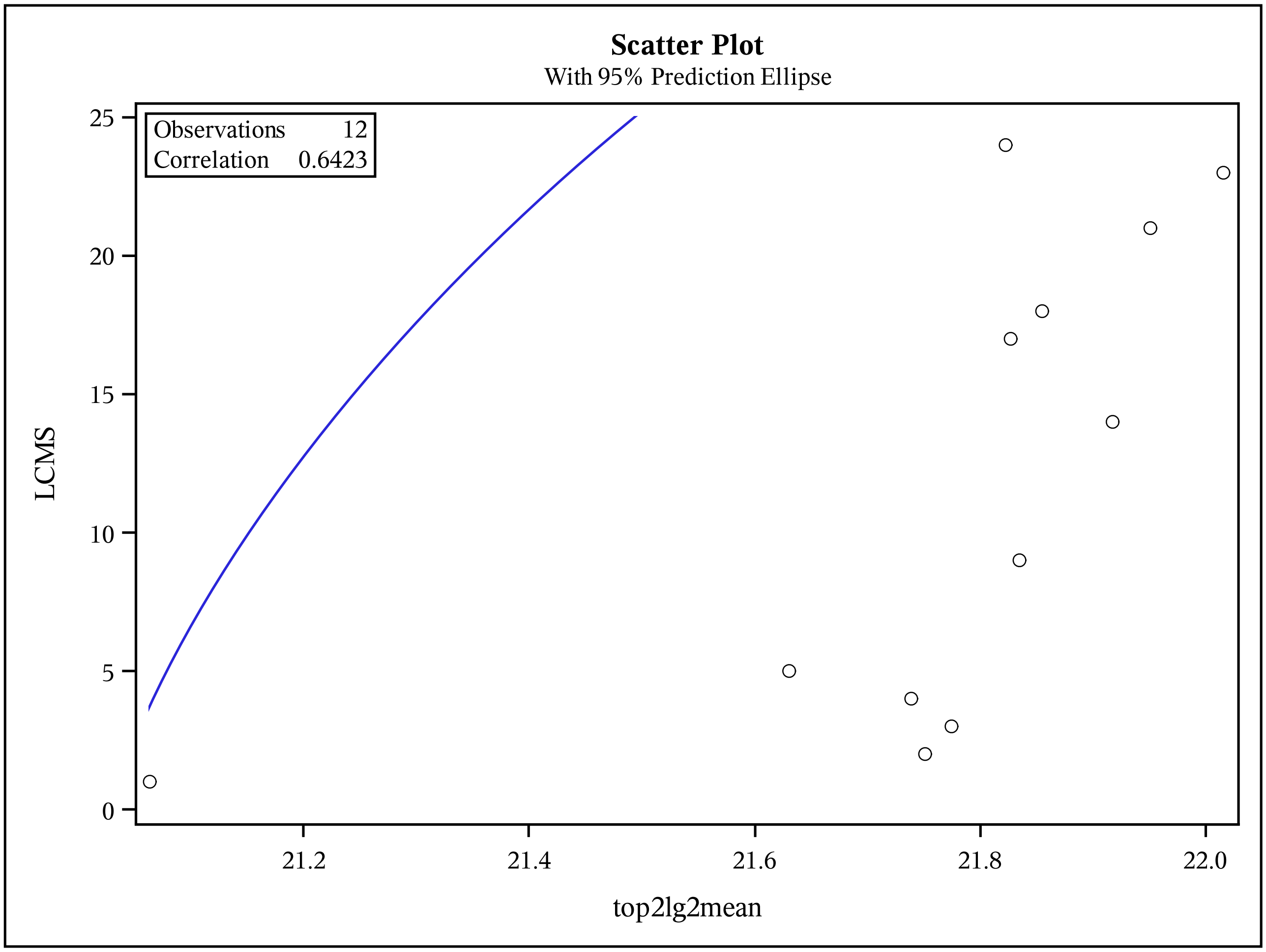

Correlation C_pooltop2lg2mean_tp2 symbol LCMS

The CORR Procedure

Gene_Symbol=KLK6

**2 Variables:**	top2lg2mean LCMS

Simple Statistics						
**Variable**	**N**	**Mean**	**Std Dev**	**Sum**	**Minimum**	**Maximum**
**top2lg2mean**	12	23.33664	0.20344	280.03972	23.06448	23.67569
**LCMS**	12	11.75000	8.70867	141.00000	1.00000	24.00000

Pearson Correlation Coefficients, N = 12<br>Prob > |r| under H0: Rho=0		
	**top2lg2mean**	**LCMS**
**top2lg2mean**	1.00000	0.34271<br>0.2755
**LCMS**	0.34271<br>0.2755	1.00000

Pearson Correlation Statistics (Fisher's z Transformation)									
**Variable**	**With Variable**	**N**	**Sample Correlation**	**Fisher's z**	**Bias Adjustment**	**Correlation Estimate**	**95% Confidence Limits**		**p Value for H0:Rho=0**
**top2lg2mean**	**LCMS**	12	0.34271	0.35716	0.01558	0.32889	-0.302019	0.759445	0.2840

Correlation C_pooltop2lg2mean_tp2 symbol LCMS

The CORR Procedure

Gene_Symbol=KLK6

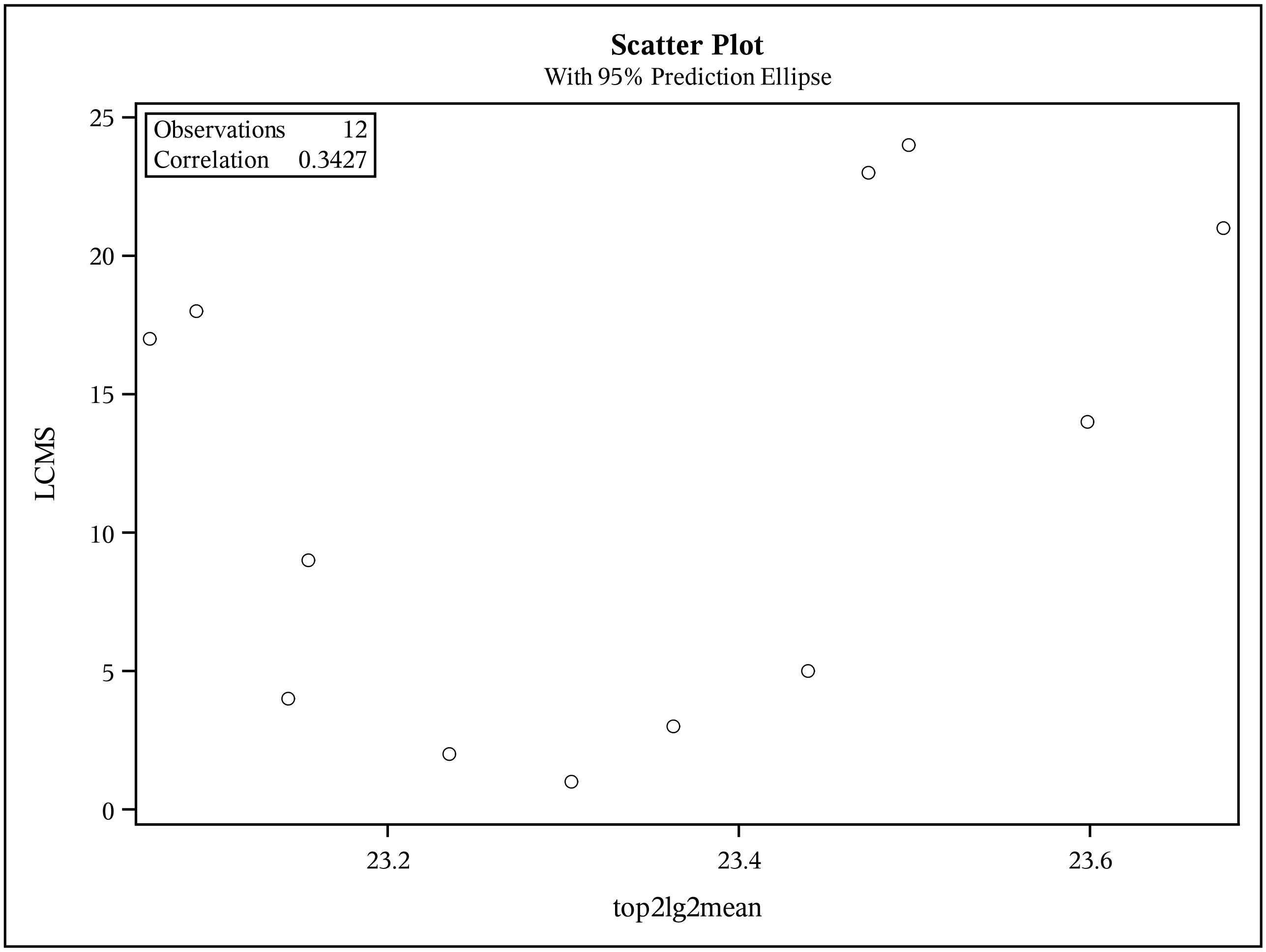

Correlation C_pooltop2lg2mean_tp2 symbol LCMS

The CORR Procedure

Gene_Symbol=KNG1

2 Variables:	top2lg2mean LCMS

Simple Statistics						
Variable	N	Mean	Std Dev	Sum	Minimum	Maximum
top2lg2mean	12	19.99606	0.33916	239.95268	19.18894	20.32536
LCMS	12	11.75000	8.70867	141.00000	1.00000	24.00000

Pearson Correlation Coefficients, N = 12<br>Prob > |r| under H0: Rho=0		
	top2lg2mean	LCMS
top2lg2mean	1.00000	0.55526<br>0.0609
LCMS	0.55526<br>0.0609	1.00000

Pearson Correlation Statistics (Fisher's z Transformation)									
Variable	With Variable	N	Sample Correlation	Fisher's z	Bias Adjustment	Correlation Estimate	95% Confidence Limits		p Value for H0:Rho=0
top2lg2mean	LCMS	12	0.55526	0.62596	0.02524	0.53756	-0.052555	0.849412	0.0604

Correlation C_pooltop2lg2mean_tp2 symbol LCMS

The CORR Procedure

Gene_Symbol=KNG1

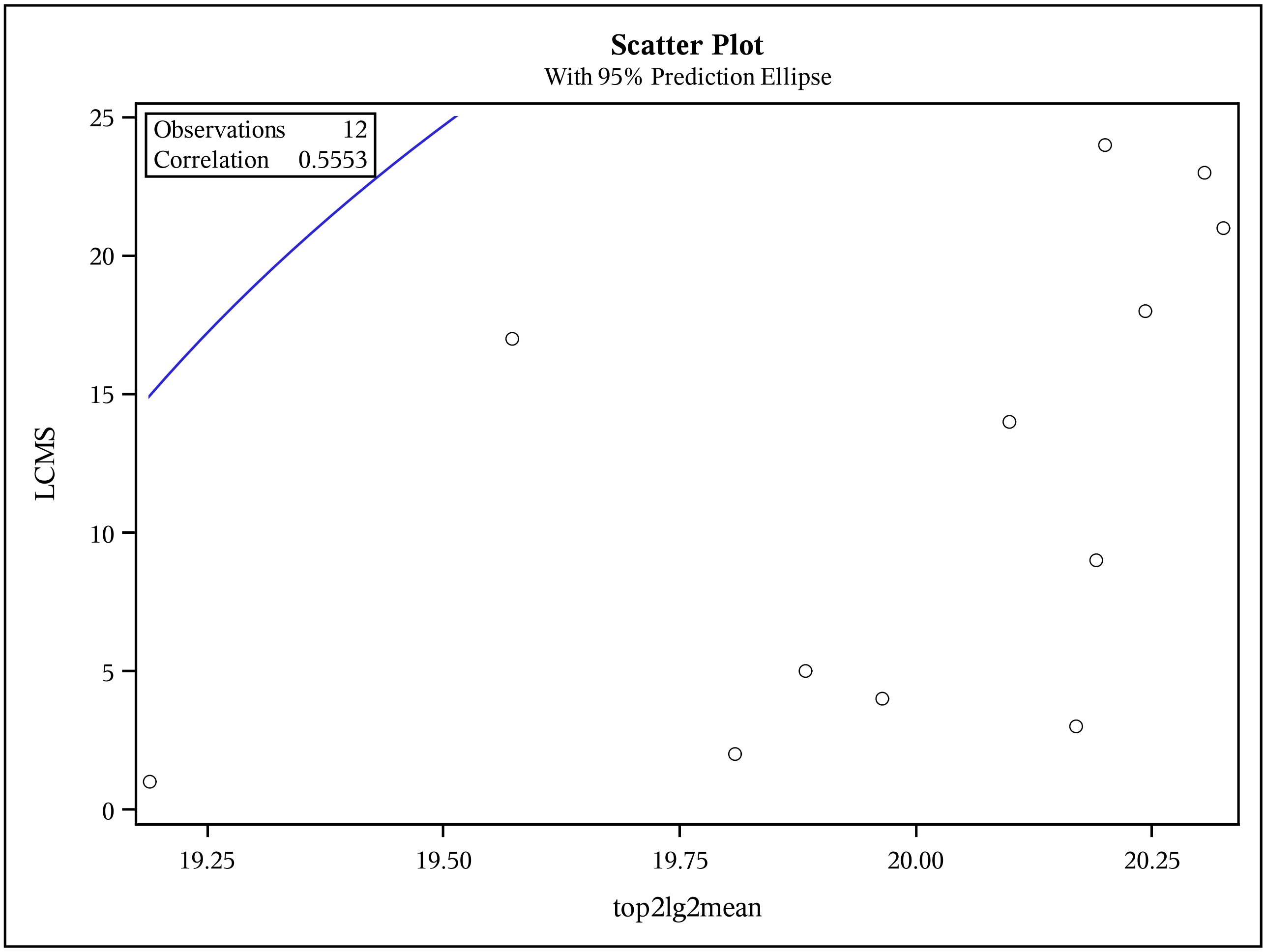

Correlation C_pooltop2lg2mean_tp2 symbol LCMS

The CORR Procedure

Gene_Symbol=LGALS3BP

2 Variables:	top2lg2mean LCMS

Simple Statistics						
Variable	N	Mean	Std Dev	Sum	Minimum	Maximum
top2lg2mean	12	19.05697	0.50398	228.68362	18.42866	19.85145
LCMS	12	11.75000	8.70867	141.00000	1.00000	24.00000

Pearson Correlation Coefficients, N = 12<br>Prob > |r| under H0: Rho=0		
	top2lg2mean	LCMS
top2lg2mean	1.00000	-0.04641<br>0.8861
LCMS	-0.04641<br>0.8861	1.00000

Pearson Correlation Statistics (Fisher's z Transformation)									
Variable	With Variable	N	Sample Correlation	Fisher's z	Bias Adjustment	Correlation Estimate	95% Confidence Limits		p Value for H0:Rho=0
top2lg2mean	LCMS	12	-0.04641	-0.04645	-0.00211	-0.04431	-0.602879	0.543412	0.8892

Correlation C_pooltop2lg2mean_tp2 symbol LCMS

The CORR Procedure

Gene_Symbol=LGALS3BP

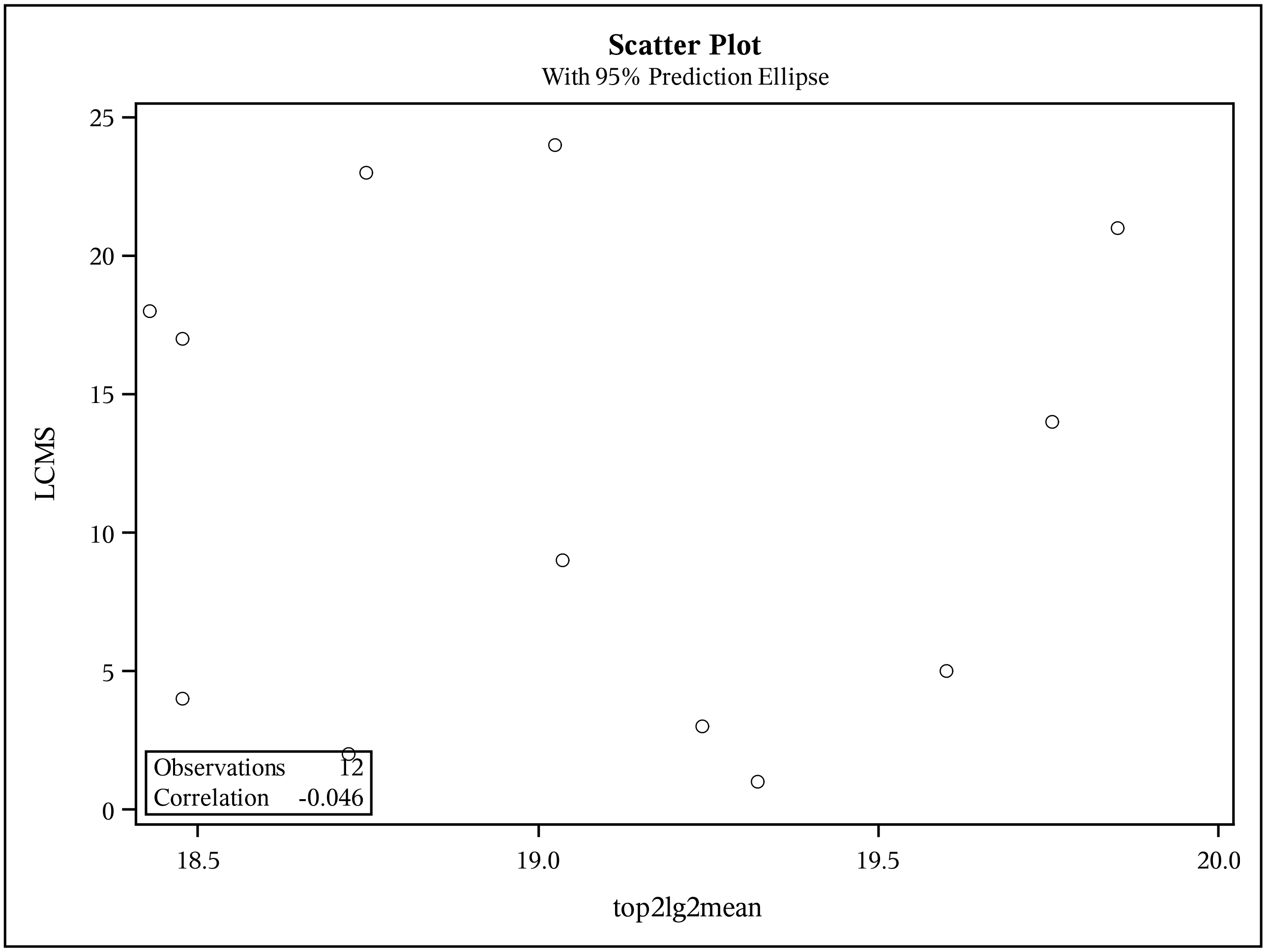

Correlation C_pooltop2lg2mean_tp2 symbol LCMS

The CORR Procedure

Gene_Symbol=LRG1

2 Variables:	top2lg2mean LCMS

Simple Statistics						
Variable	N	Mean	Std Dev	Sum	Minimum	Maximum
top2lg2mean	12	21.04480	0.32579	252.53765	20.54096	21.57320
LCMS	12	11.75000	8.70867	141.00000	1.00000	24.00000

Pearson Correlation Coefficients, N = 12<br>Prob > |r| under H0: Rho=0		
	top2lg2mean	LCMS
top2lg2mean	1.00000	0.20461<br>0.5235
LCMS	0.20461<br>0.5235	1.00000

Pearson Correlation Statistics (Fisher's z Transformation)									
Variable	With Variable	N	Sample Correlation	Fisher's z	Bias Adjustment	Correlation Estimate	95% Confidence Limits		p Value for H0:Rho=0
top2lg2mean	LCMS	12	0.20461	0.20754	0.00930	0.19568	-0.426069	0.691883	0.5335

Correlation C_pooltop2lg2mean_tp2 symbol LCMS

The CORR Procedure

Gene_Symbol=LRG1

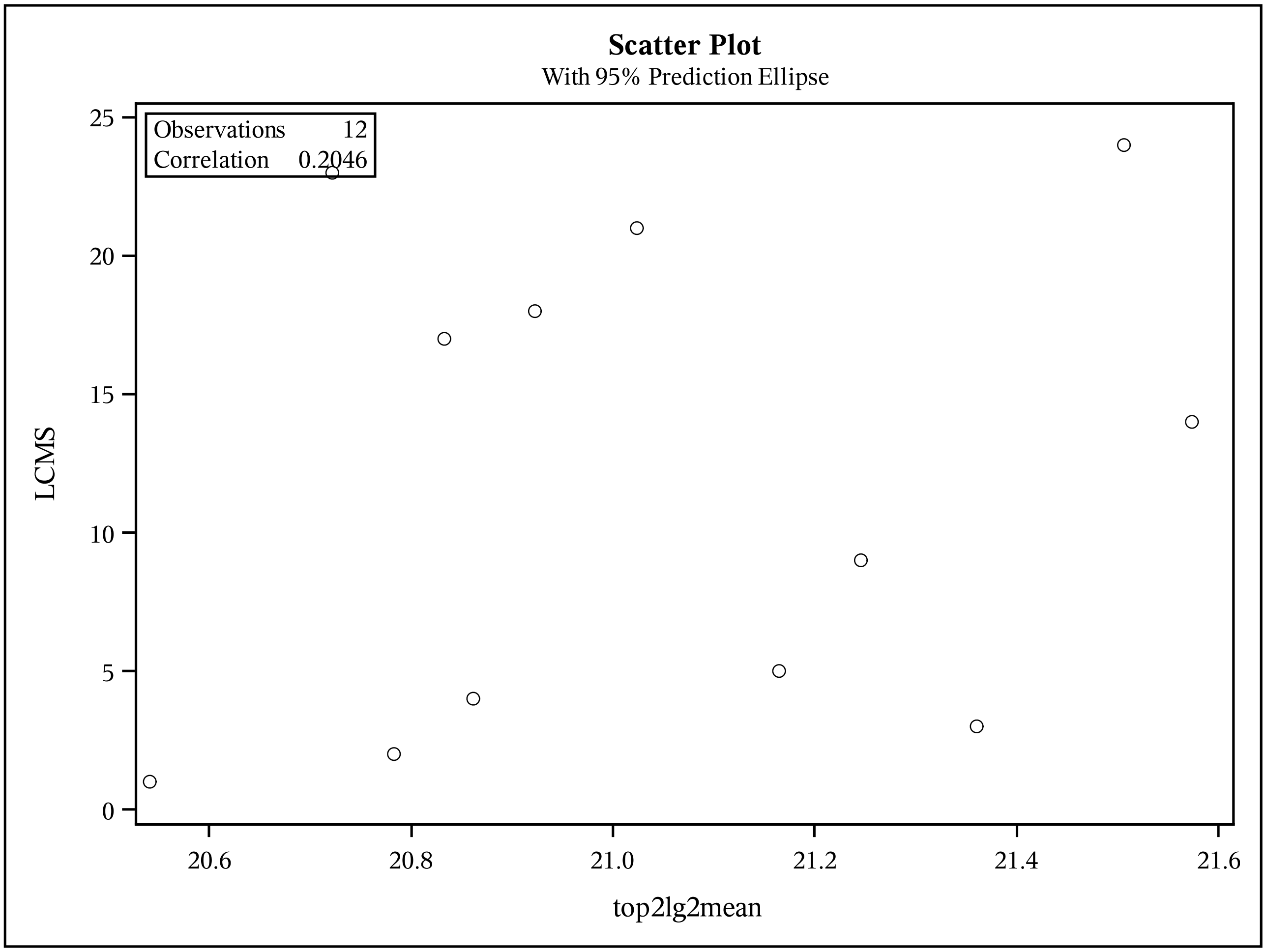

Correlation C_pooltop2lg2mean_tp2 symbol LCMS

The CORR Procedure

Gene_Symbol=NCAM1

**2 Variables:**	top2lg2mean LCMS

Simple Statistics						
**Variable**	**N**	**Mean**	**Std Dev**	**Sum**	**Minimum**	**Maximum**
**top2lg2mean**	12	20.08905	0.30789	241.06857	19.47076	20.43145
**LCMS**	12	11.75000	8.70867	141.00000	1.00000	24.00000

Pearson Correlation Coefficients, N = 12<br>Prob > |r| under H0: Rho=0		
	**top2lg2mean**	**LCMS**
**top2lg2mean**	1.00000	0.01436<br>0.9647
**LCMS**	0.01436<br>0.9647	1.00000

Pearson Correlation Statistics (Fisher's z Transformation)									
**Variable**	**With Variable**	**N**	**Sample Correlation**	**Fisher's z**	**Bias Adjustment**	**Correlation Estimate**	**95% Confidence Limits**		**p Value for H0:Rho=0**
**top2lg2mean**	**LCMS**	12	0.01436	0.01436	0.0006527	0.01371	-0.564637	0.583022	0.9656

Correlation C_pooltop2lg2mean_tp2 symbol LCMS

The CORR Procedure

Gene_Symbol=NCAM1

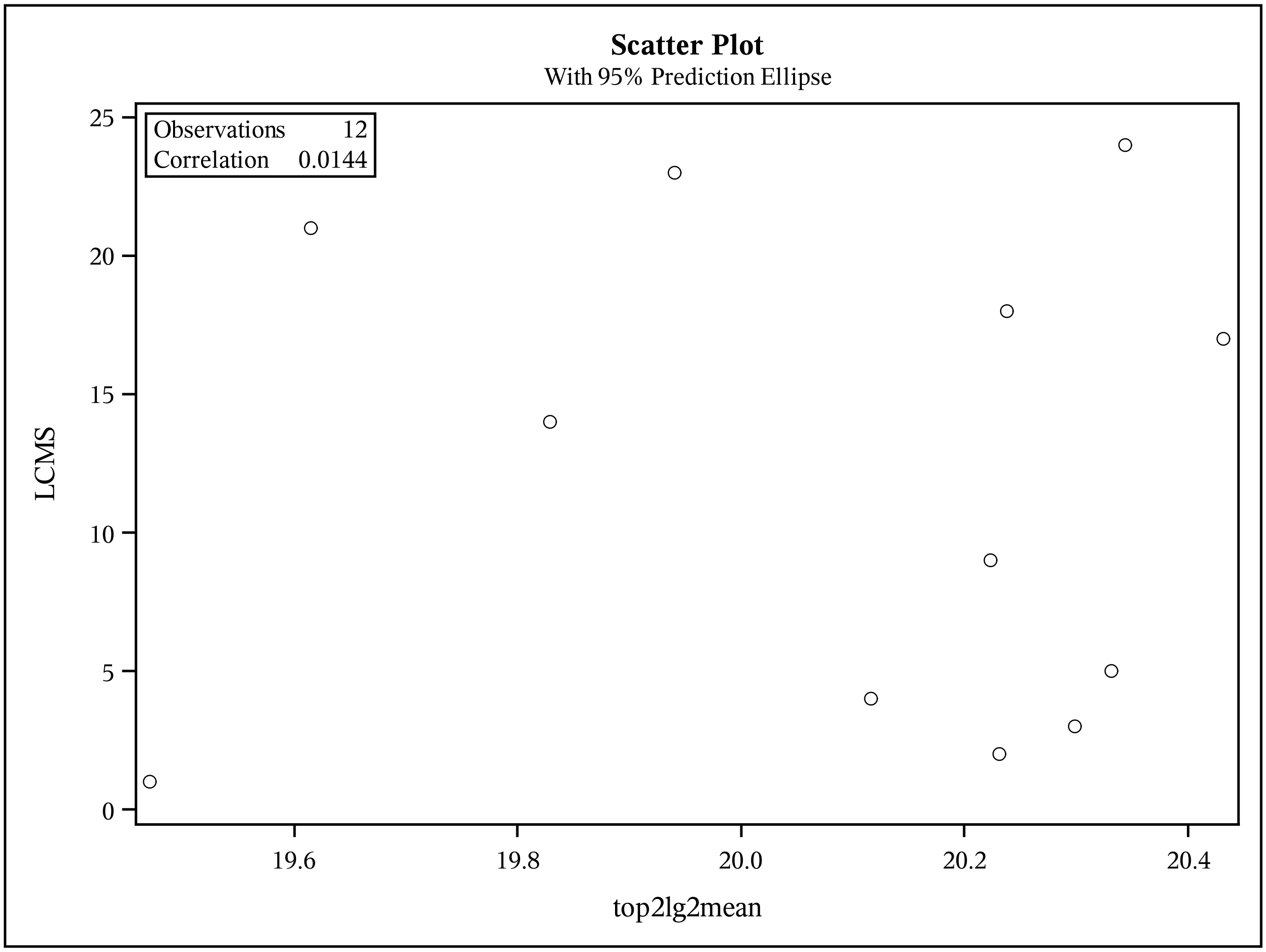

Correlation C_pooltop2lg2mean_tp2 symbol LCMS

The CORR Procedure

Gene_Symbol=NCAN

2 Variables:	top2lg2mean LCMS

Simple Statistics						
**Variable**	**N**	**Mean**	**Std Dev**	**Sum**	**Minimum**	**Maximum**
**top2lg2mean**	12	17.41564	0.26096	208.98771	16.95932	17.82961
**LCMS**	12	11.75000	8.70867	141.00000	1.00000	24.00000

Pearson Correlation Coefficients, N = 12<br>Prob > |r| under H0: Rho=0		
	**top2lg2mean**	**LCMS**
**top2lg2mean**	1.00000	-0.78699<br>0.0024
**LCMS**	-0.78699<br>0.0024	1.00000

Pearson Correlation Statistics (Fisher's z Transformation)									
**Variable**	**With Variable**	**N**	**Sample Correlation**	**Fisher's z**	**Bias Adjustment**	**Correlation Estimate**	**95% Confidence Limits**		**p Value for H0:Rho=0**
**top2lg2mean**	**LCMS**	12	-0.78699	-1.06347	-0.03577	-0.77298	-0.932994	-0.357816	0.0014

Correlation C_pooltop2lg2mean_tp2 symbol LCMS

The CORR Procedure

Gene_Symbol=NCAN

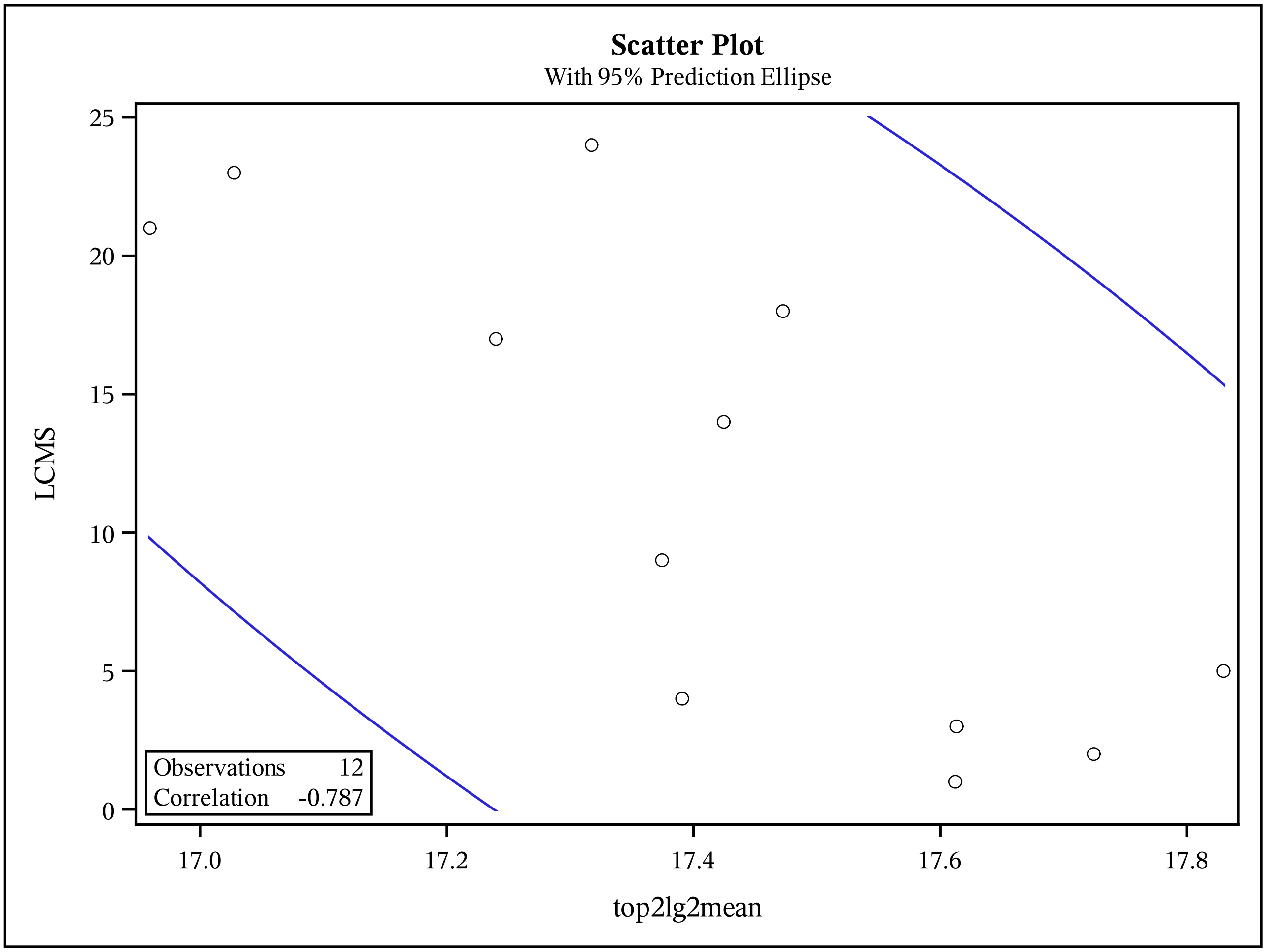

Correlation C_pooltop2lg2mean_tp2 symbol LCMS

The CORR Procedure

Gene_Symbol=NELL2

**2 Variables:**	top2lg2mean LCMS

Simple Statistics						
**Variable**	**N**	**Mean**	**Std Dev**	**Sum**	**Minimum**	**Maximum**
**top2lg2mean**	12	19.96714	0.43713	239.60562	19.30654	20.90915
**LCMS**	12	11.75000	8.70867	141.00000	1.00000	24.00000

Pearson Correlation Coefficients, N = 12<br>Prob > |r| under H0: Rho=0		
	**top2lg2mean**	**LCMS**
**top2lg2mean**	1.00000	-0.21615<br>0.4998
**LCMS**	-0.21615<br>0.4998	1.00000

Pearson Correlation Statistics (Fisher's z Transformation)									
**Variable**	**With Variable**	**N**	**Sample Correlation**	**Fisher's z**	**Bias Adjustment**	**Correlation Estimate**	**95% Confidence Limits**		**p Value for H0:Rho=0**
**top2lg2mean**	**LCMS**	12	-0.21615	-0.21962	-0.00983	-0.20677	-0.697858	0.416566	0.5100

Correlation C_pooltop2lg2mean_tp2 symbol LCMS

The CORR Procedure

Gene_Symbol=NELL2

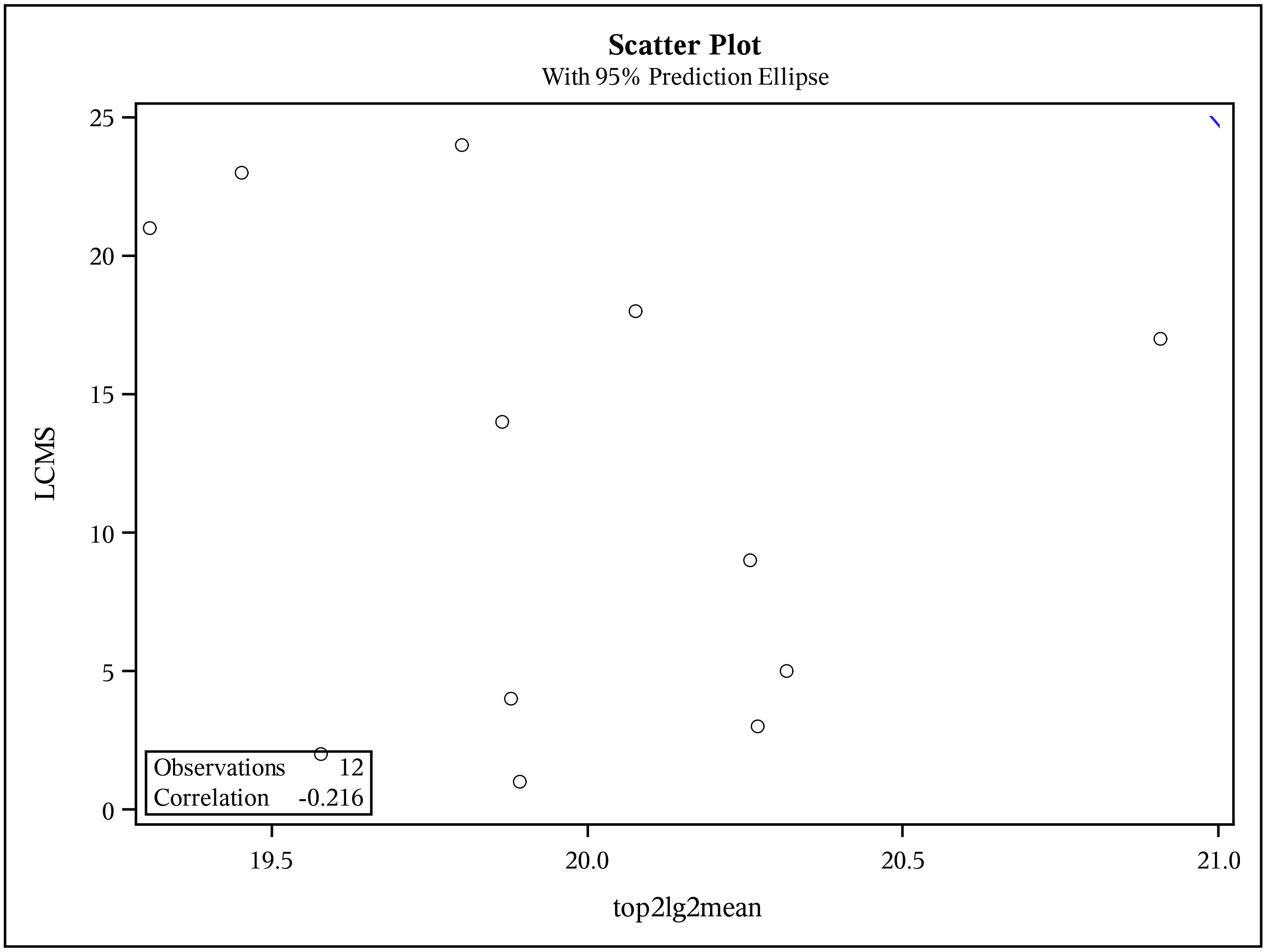

Correlation C_pooltop2lg2mean_tp2 symbol LCMS

The CORR Procedure

Gene_Symbol=NEO1

2 Variables:	top2lg2mean LCMS

Simple Statistics						
Variable	N	Mean	Std Dev	Sum	Minimum	Maximum
top2lg2mean	12	18.63564	0.41217	223.62765	17.85830	19.26165
LCMS	12	11.75000	8.70867	141.00000	1.00000	24.00000

Pearson Correlation Coefficients, N = 12<br>Prob > |r| under H0: Rho=0		
	top2lg2mean	LCMS
top2lg2mean	1.00000	0.69720<br>0.0117
LCMS	0.69720<br>0.0117	1.00000

Pearson Correlation Statistics (Fisher's z Transformation)									
Variable	With Variable	N	Sample Correlation	Fisher's z	Bias Adjustment	Correlation Estimate	95% Confidence Limits		p Value for H0:Rho=0
top2lg2mean	LCMS	12	0.69720	0.86184	0.03169	0.68056	0.175007	0.902116	0.0097

Correlation C_pooltop2lg2mean_tp2 symbol LCMS

The CORR Procedure

Gene_Symbol=NEO1

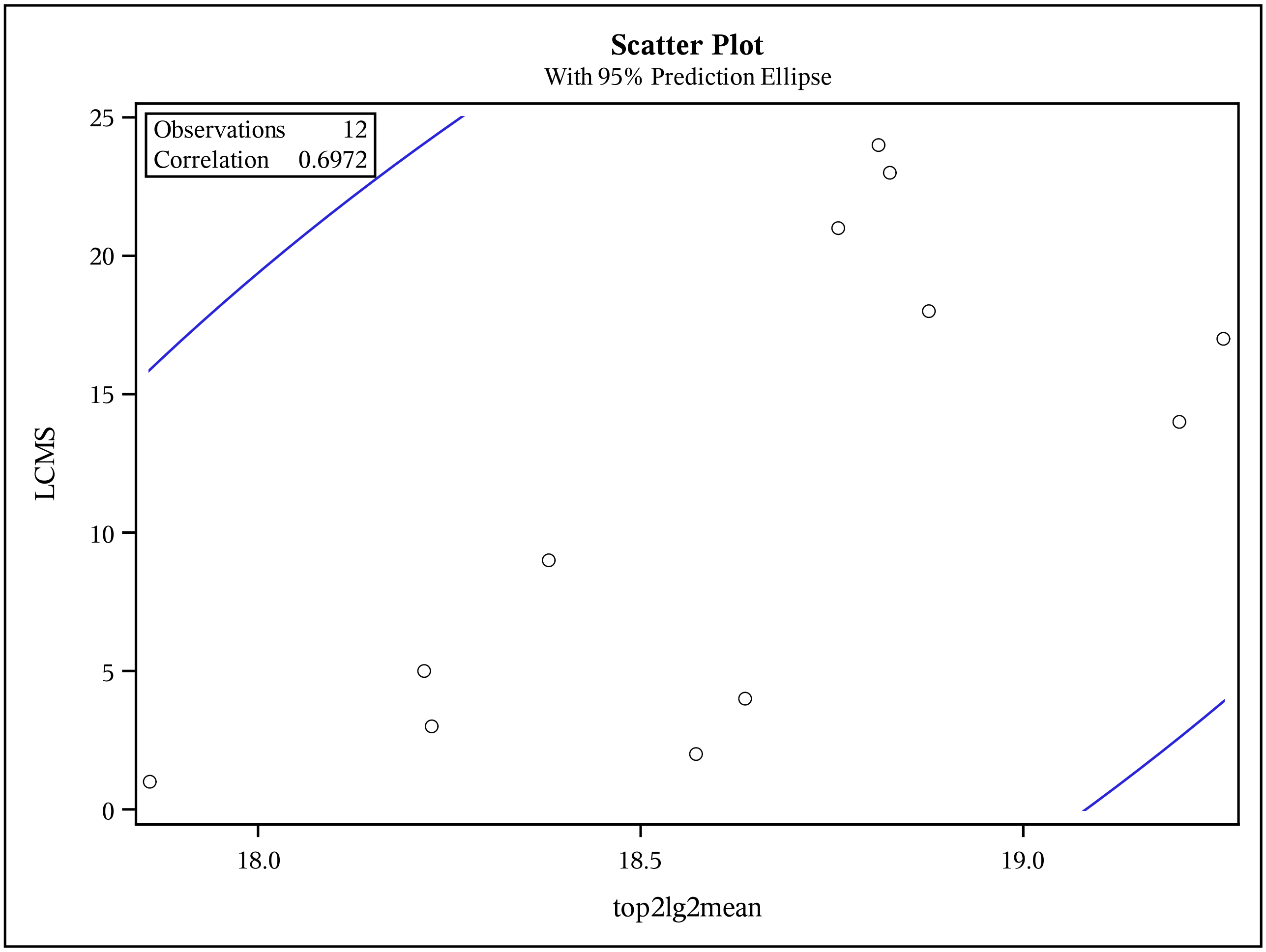

Correlation C_pooltop2lg2mean_tp2 symbol LCMS

The CORR Procedure

Gene_Symbol=NPC2

2 Variables:	top2lg2mean LCMS

Simple Statistics						
Variable	N	Mean	Std Dev	Sum	Minimum	Maximum
top2lg2mean	12	19.67006	0.50896	236.04070	18.82205	20.78194
LCMS	12	11.75000	8.70867	141.00000	1.00000	24.00000

Pearson Correlation Coefficients, N = 12<br>Prob > |r| under H0: Rho=0		
	top2lg2mean	LCMS
top2lg2mean	1.00000	-0.45667<br>0.1356
LCMS	-0.45667<br>0.1356	1.00000

Pearson Correlation Statistics (Fisher's z Transformation)									
Variable	With Variable	N	Sample Correlation	Fisher's z	Bias Adjustment	Correlation Estimate	95% Confidence Limits		p Value for H0:Rho=0
top2lg2mean	LCMS	12	-0.45667	-0.49309	-0.02076	-0.44008	-0.809527	0.179036	0.1391

Correlation C_pooltop2lg2mean_tp2 symbol LCMS

The CORR Procedure

Gene_Symbol=NPC2

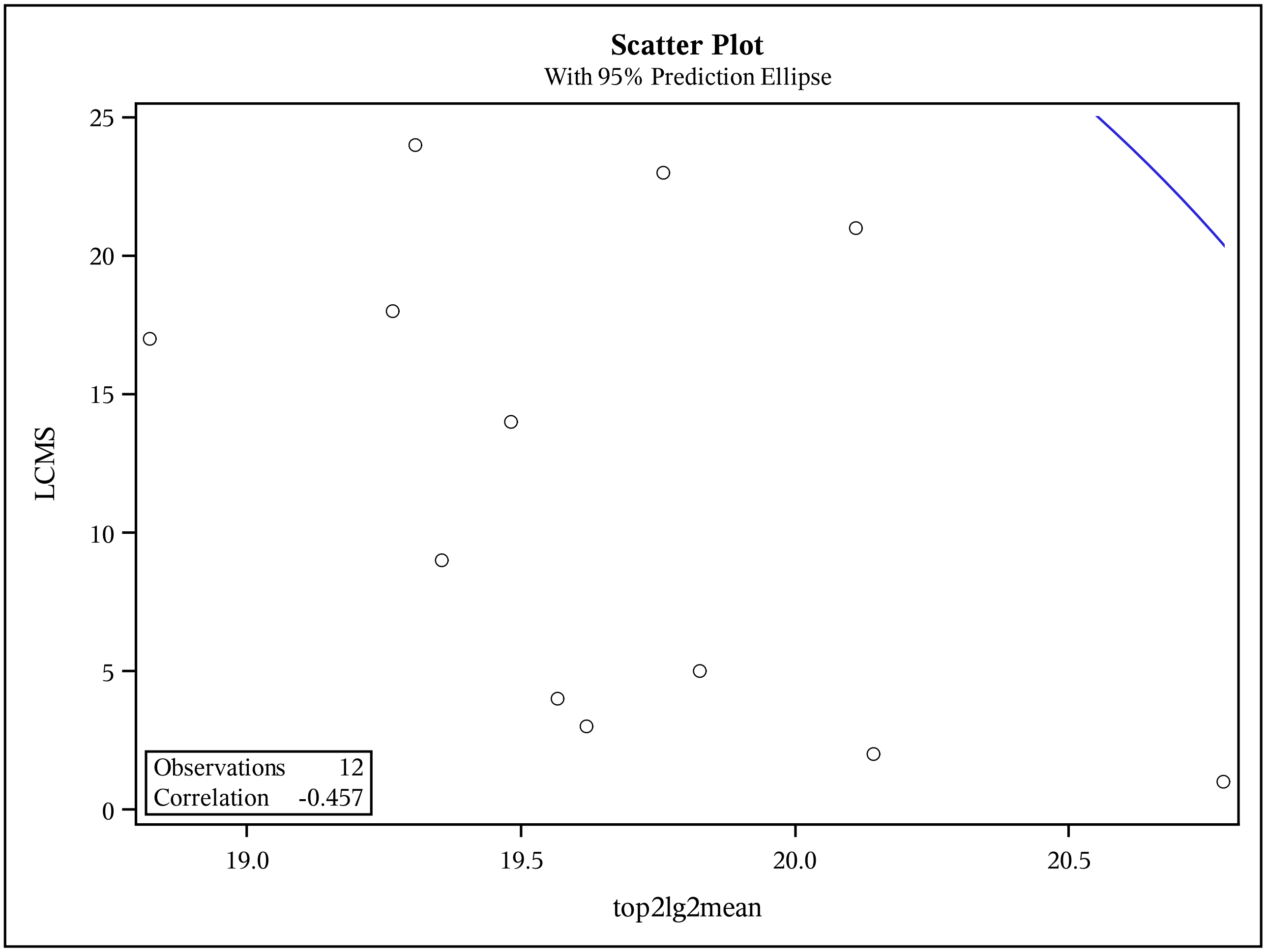

Correlation C_pooltop2lg2mean_tp2 symbol LCMS

The CORR Procedure

Gene_Symbol=NRCAM

2 Variables:	top2lg2mean LCMS

Simple Statistics						
**Variable**	**N**	**Mean**	**Std Dev**	**Sum**	**Minimum**	**Maximum**
**top2lg2mean**	12	20.82374	0.27138	249.88485	20.37181	21.20853
**LCMS**	12	11.75000	8.70867	141.00000	1.00000	24.00000

Pearson Correlation Coefficients, N = 12<br>Prob > |r| under H0: Rho=0		
	**top2lg2mean**	**LCMS**
**top2lg2mean**	1.00000	-0.07480<br>0.8173
**LCMS**	-0.07480<br>0.8173	1.00000

Pearson Correlation Statistics (Fisher's z Transformation)									
**Variable**	**With Variable**	**N**	**Sample Correlation**	**Fisher's z**	**Bias Adjustment**	**Correlation Estimate**	**95% Confidence Limits**		**p Value for H0:Rho=0**
**top2lg2mean**	**LCMS**	12	-0.07480	-0.07494	-0.00340	-0.07142	-0.619913	0.523957	0.8221

Correlation C_pooltop2lg2mean_tp2 symbol LCMS

The CORR Procedure

Gene_Symbol=NRCAM

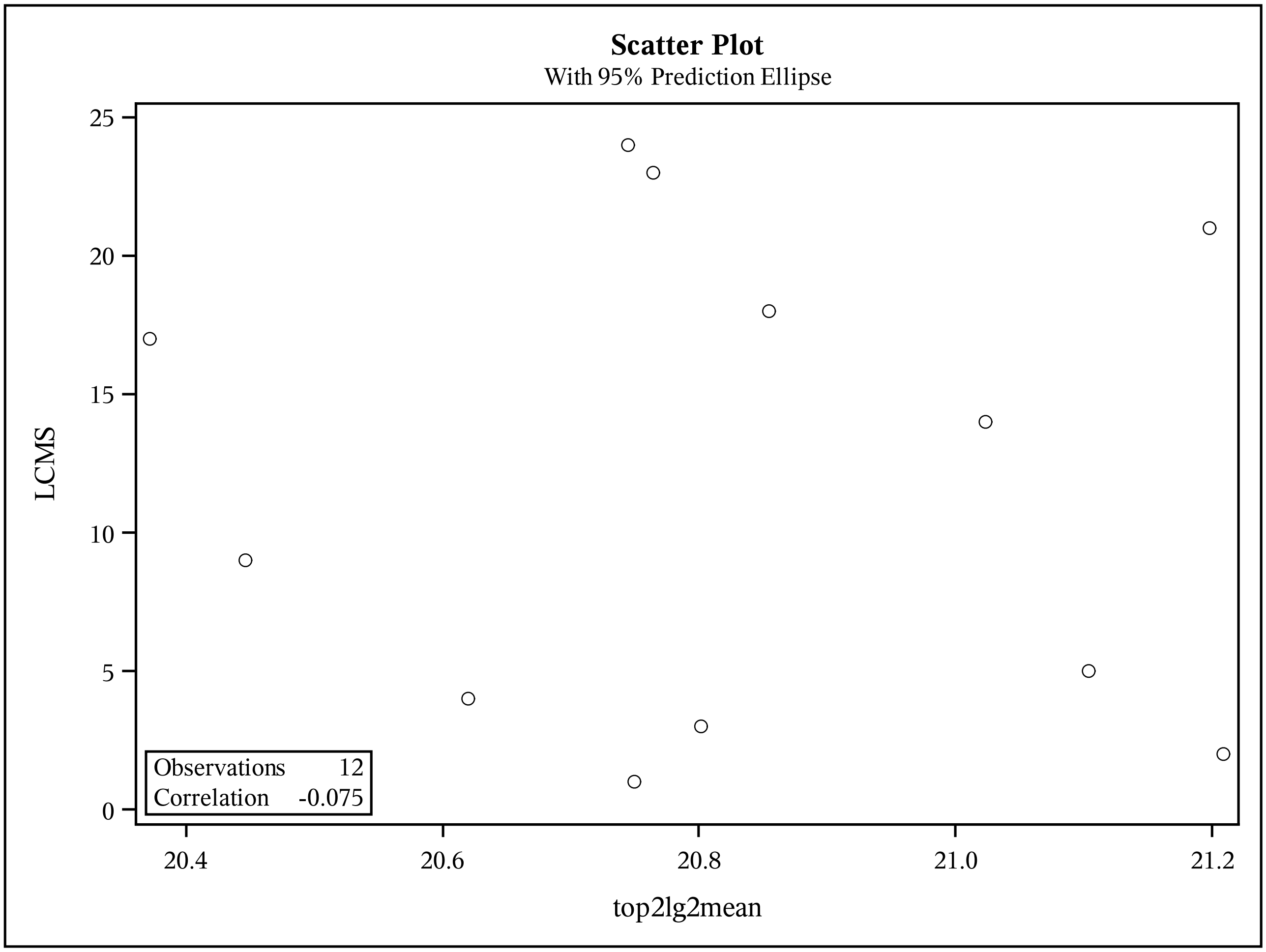

Correlation C_pooltop2lg2mean_tp2 symbol LCMS

The CORR Procedure

Gene_Symbol=NTM

2 Variables:	top2lg2mean LCMS

Simple Statistics						
Variable	N	Mean	Std Dev	Sum	Minimum	Maximum
top2lg2mean	12	19.04428	0.32215	228.53139	18.56987	19.55018
LCMS	12	11.75000	8.70867	141.00000	1.00000	24.00000

Pearson Correlation Coefficients, N = 12<br>Prob > |r| under H0: Rho=0		
	top2lg2mean	LCMS
top2lg2mean	1.00000	-0.36423<br>0.2444
LCMS	-0.36423<br>0.2444	1.00000

Pearson Correlation Statistics (Fisher's z Transformation)									
Variable	With Variable	N	Sample Correlation	Fisher's z	Bias Adjustment	Correlation Estimate	95% Confidence Limits		p Value for H0:Rho=0
top2lg2mean	LCMS	12	-0.36423	-0.38176	-0.01656	-0.34979	-0.769264	0.280404	0.2521

Correlation C_pooltop2lg2mean_tp2 symbol LCMS

The CORR Procedure

Gene_Symbol=NTM

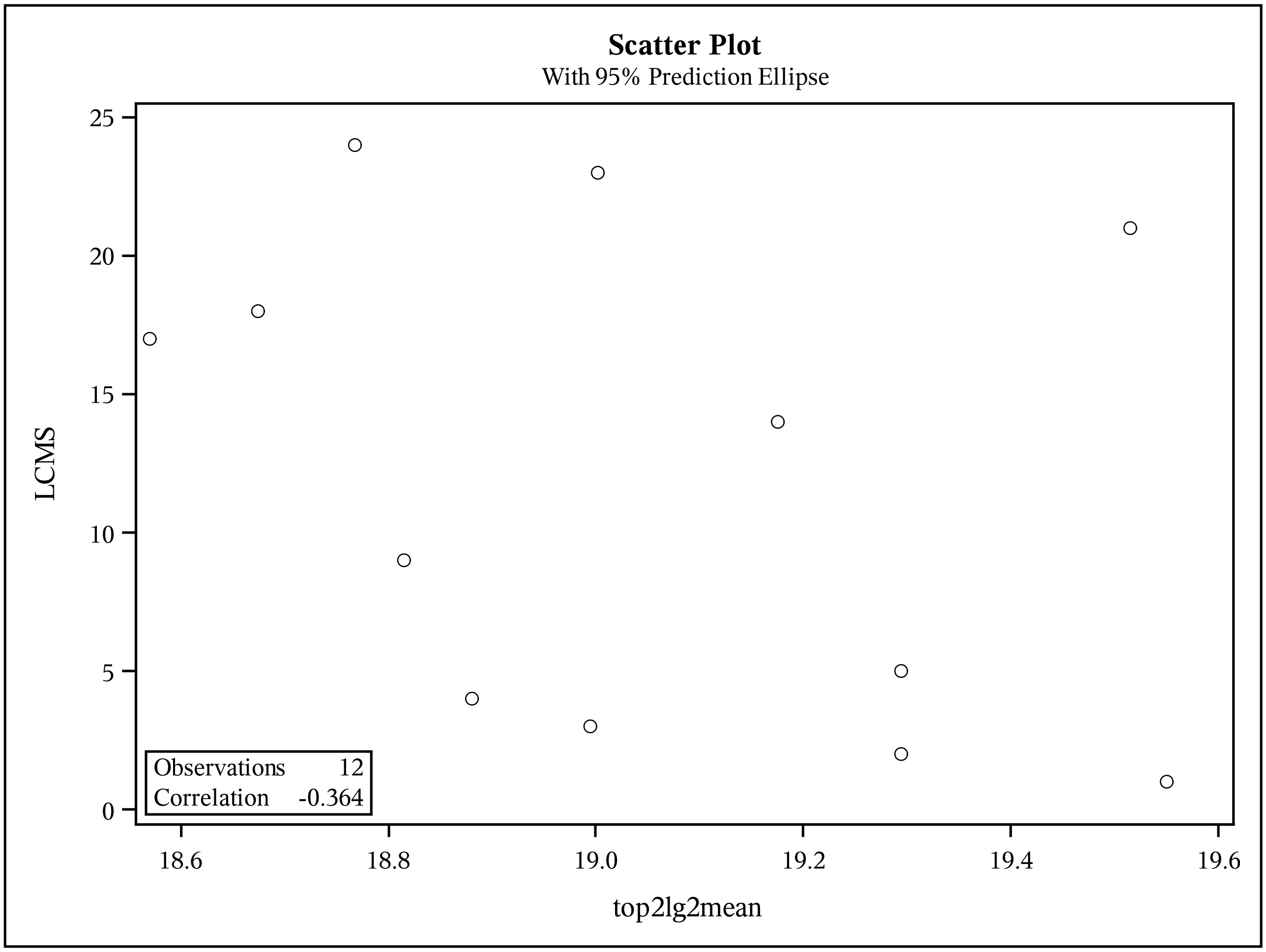

Correlation C_pooltop2lg2mean_tp2 symbol LCMS

The CORR Procedure

Gene_Symbol=OGN

**2 Variables:**	top2lg2mean LCMS

Simple Statistics						
**Variable**	**N**	**Mean**	**Std Dev**	**Sum**	**Minimum**	**Maximum**
**top2lg2mean**	12	20.80877	0.64074	249.70526	19.75513	22.16848
**LCMS**	12	11.75000	8.70867	141.00000	1.00000	24.00000

Pearson Correlation Coefficients, N = 12<br>Prob > |r| under H0: Rho=0		
	**top2lg2mean**	**LCMS**
**top2lg2mean**	1.00000	-0.04068<br>0.9001
**LCMS**	-0.04068<br>0.9001	1.00000

Pearson Correlation Statistics (Fisher's z Transformation)									
**Variable**	**With Variable**	**N**	**Sample Correlation**	**Fisher's z**	**Bias Adjustment**	**Correlation Estimate**	**95% Confidence Limits**		**p Value for H0:Rho=0**
**top2lg2mean**	**LCMS**	12	-0.04068	-0.04070	-0.00185	-0.03883	-0.599378	0.547264	0.9028

Correlation C_pooltop2lg2mean_tp2 symbol LCMS

The CORR Procedure

Gene_Symbol=OGN

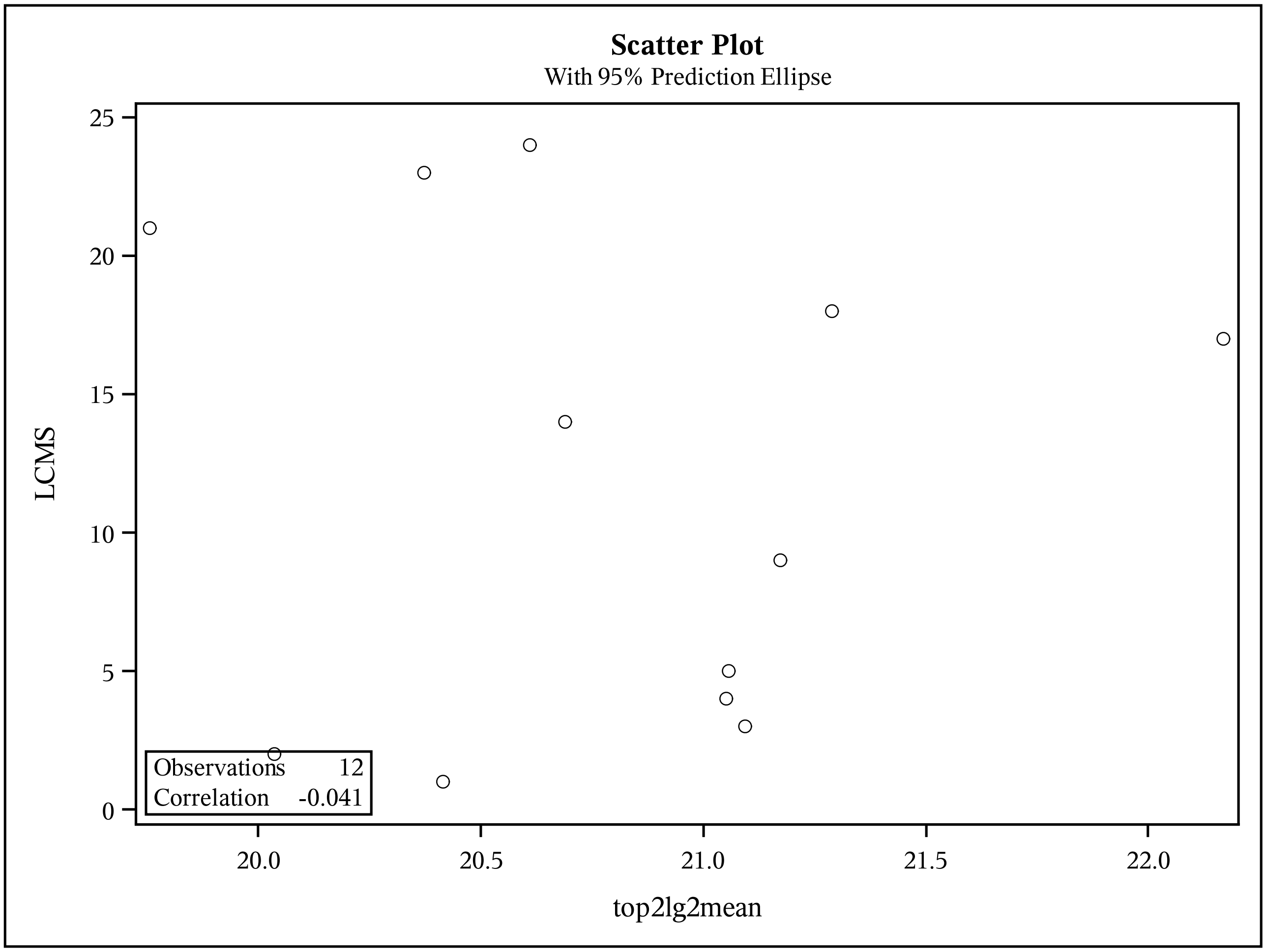

Correlation C_pooltop2lg2mean_tp2 symbol LCMS

The CORR Procedure

Gene_Symbol=ORM1

2 Variables:	top2lg2mean LCMS

Simple Statistics						
Variable	N	Mean	Std Dev	Sum	Minimum	Maximum
top2lg2mean	12	25.27837	0.18764	303.34041	24.93241	25.59154
LCMS	12	11.75000	8.70867	141.00000	1.00000	24.00000

Pearson Correlation Coefficients, N = 12<br>Prob > |r| under H0: Rho=0		
	top2lg2mean	LCMS
top2lg2mean	1.00000	0.48114<br>0.1133
LCMS	0.48114<br>0.1133	1.00000

Pearson Correlation Statistics (Fisher's z Transformation)									
Variable	With Variable	N	Sample Correlation	Fisher's z	Bias Adjustment	Correlation Estimate	95% Confidence Limits		p Value for H0:Rho=0
top2lg2mean	LCMS	12	0.48114	0.52446	0.02187	0.46415	-0.149597	0.819704	0.1156

Correlation C_pooltop2lg2mean_tp2 symbol LCMS

The CORR Procedure

Gene_Symbol=ORM1

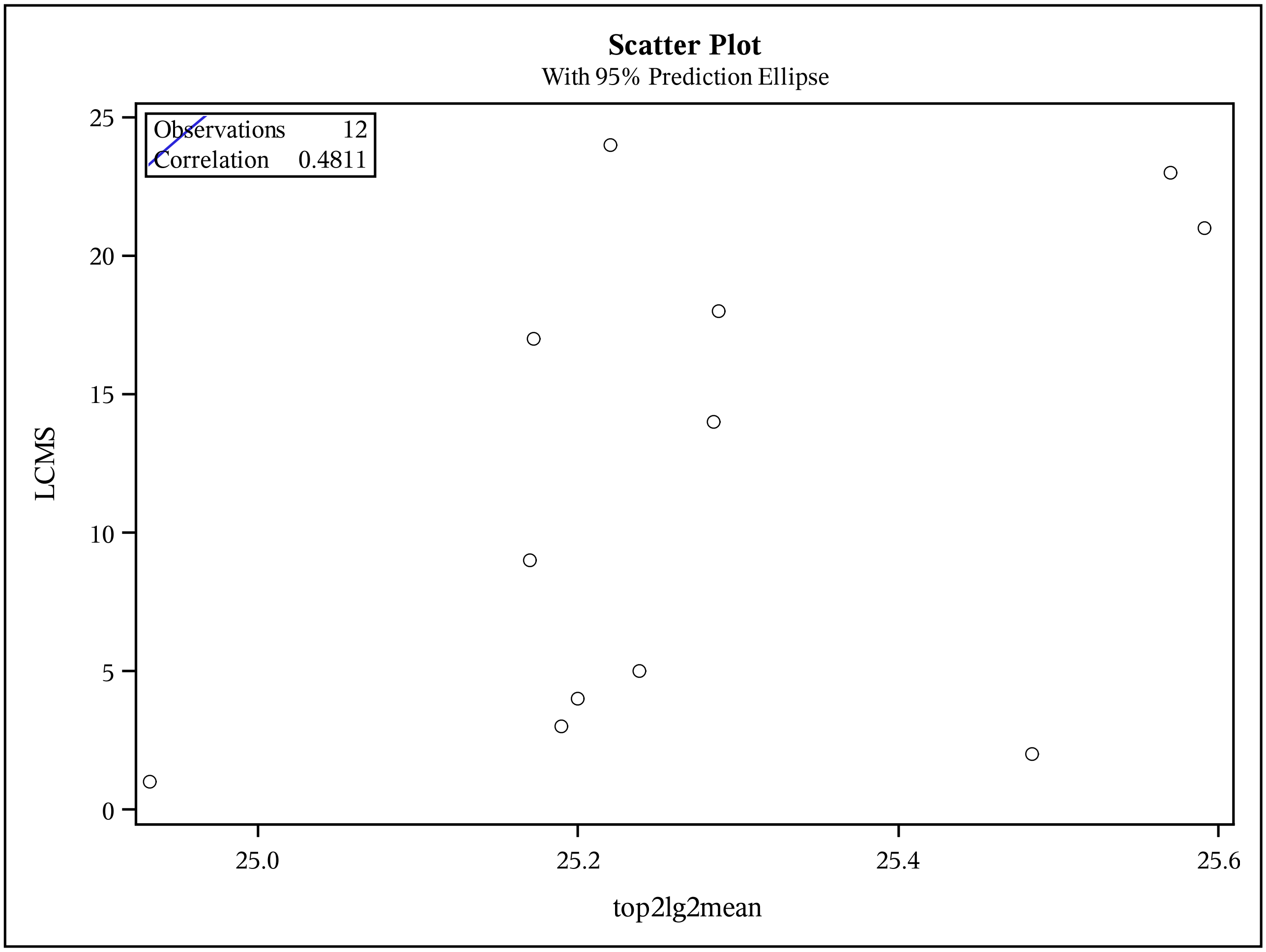

Correlation C_pooltop2lg2mean_tp2 symbol LCMS

The CORR Procedure

Gene_Symbol=PKM2

2 Variables:	top2lg2mean LCMS

Simple Statistics						
Variable	N	Mean	Std Dev	Sum	Minimum	Maximum
top2lg2mean	12	16.02777	0.55684	192.33324	15.22430	17.32496
LCMS	12	11.75000	8.70867	141.00000	1.00000	24.00000

Pearson Correlation Coefficients, N = 12<br>Prob > |r| under H0: Rho=0		
	top2lg2mean	LCMS
top2lg2mean	1.00000	0.35515<br>0.2573
LCMS	0.35515<br>0.2573	1.00000

Pearson Correlation Statistics (Fisher's z Transformation)									
Variable	With Variable	N	Sample Correlation	Fisher's z	Bias Adjustment	Correlation Estimate	95% Confidence Limits		p Value for H0:Rho=0
top2lg2mean	LCMS	12	0.35515	0.37133	0.01614	0.34096	-0.289608	0.765142	0.2653

Correlation C_pooltop2lg2mean_tp2 symbol LCMS

The CORR Procedure

Gene_Symbol=PKM2

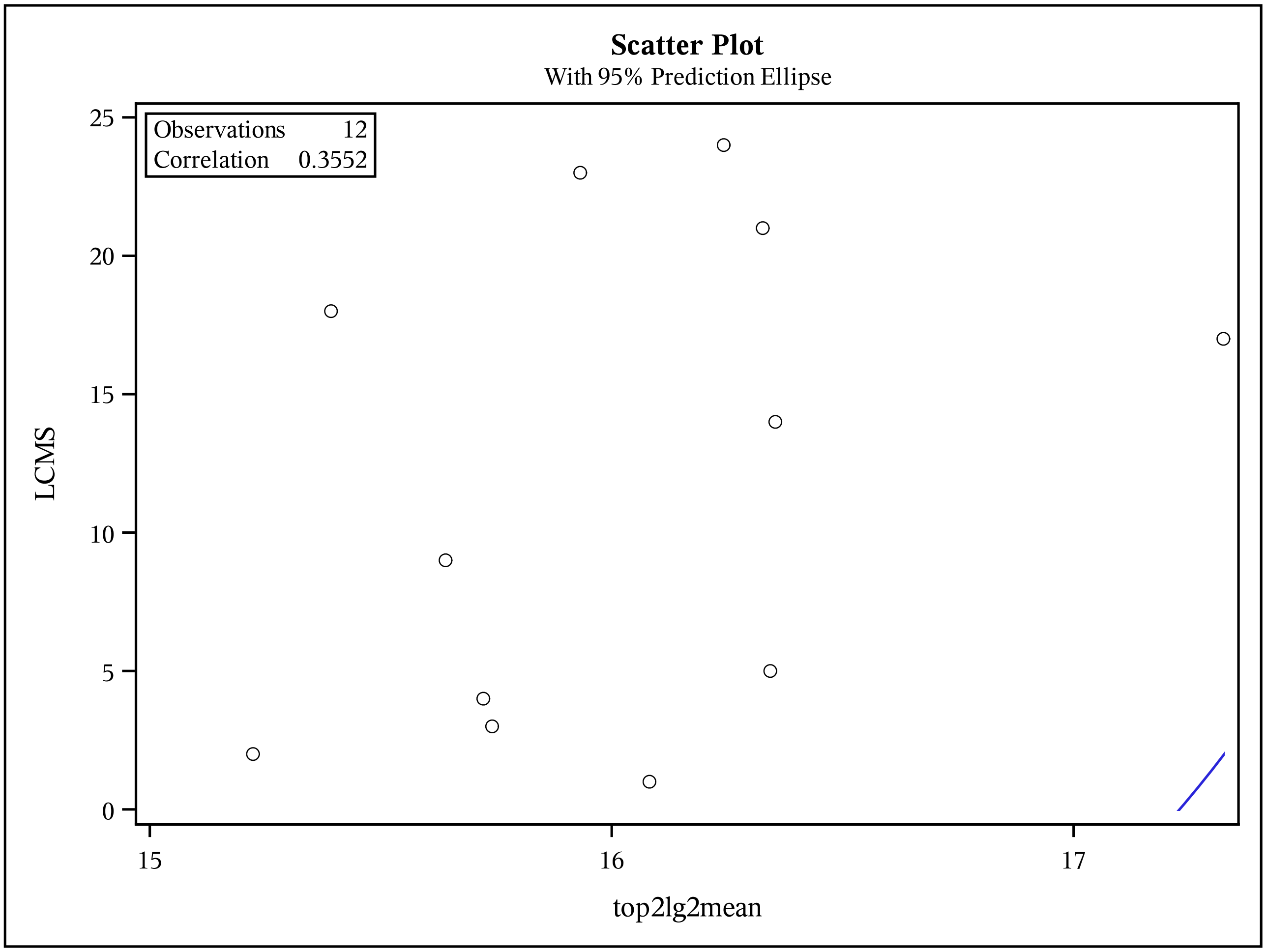

Correlation C_pooltop2lg2mean_tp2 symbol LCMS

The CORR Procedure

Gene_Symbol=PLG

**2 Variables:**	top2lg2mean LCMS

Simple Statistics						
**Variable**	**N**	**Mean**	**Std Dev**	**Sum**	**Minimum**	**Maximum**
**top2lg2mean**	12	20.94222	0.16342	251.30666	20.64623	21.19274
**LCMS**	12	11.75000	8.70867	141.00000	1.00000	24.00000

Pearson Correlation Coefficients, N = 12<br>Prob > |r| under H0: Rho=0		
	**top2lg2mean**	**LCMS**
**top2lg2mean**	1.00000	0.62345<br>0.0303
**LCMS**	0.62345<br>0.0303	1.00000

Pearson Correlation Statistics (Fisher's z Transformation)									
**Variable**	**With Variable**	**N**	**Sample Correlation**	**Fisher's z**	**Bias Adjustment**	**Correlation Estimate**	**95% Confidence Limits**		**p Value for H0:Rho=0**
**top2lg2mean**	**LCMS**	12	0.62345	0.73062	0.02834	0.60582	0.048924	0.875370	0.0284

Correlation C_pooltop2lg2mean_tp2 symbol LCMS

The CORR Procedure

Gene_Symbol=PLG

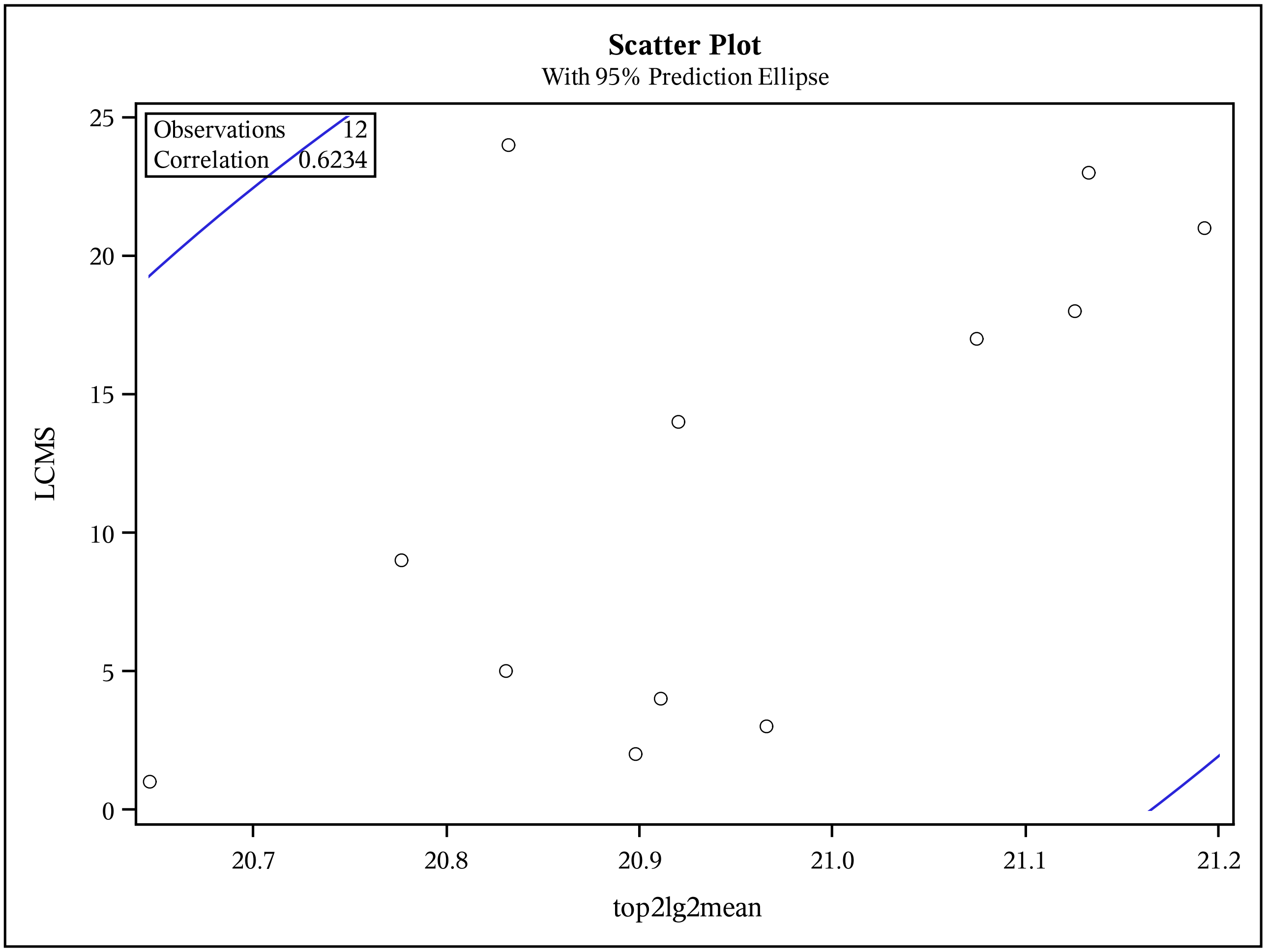

Correlation C_pooltop2lg2mean_tp2 symbol LCMS

The CORR Procedure

Gene_Symbol=PSAP

2 Variables:	top2lg2mean LCMS

Simple Statistics						
Variable	N	Mean	Std Dev	Sum	Minimum	Maximum
top2lg2mean	12	17.99037	0.31361	215.88439	17.41062	18.37198
LCMS	12	11.75000	8.70867	141.00000	1.00000	24.00000

Pearson Correlation Coefficients, N = 12<br>Prob > |r| under H0: Rho=0		
	top2lg2mean	LCMS
top2lg2mean	1.00000	-0.29472<br>0.3524
LCMS	-0.29472<br>0.3524	1.00000

Pearson Correlation Statistics (Fisher's z Transformation)									
Variable	With Variable	N	Sample Correlation	Fisher's z	Bias Adjustment	Correlation Estimate	95% Confidence Limits		p Value for H0:Rho=0
top2lg2mean	LCMS	12	-0.29472	-0.30372	-0.01340	-0.28243	-0.736893	0.347850	0.3622

Correlation C_pooltop2lg2mean_tp2 symbol LCMS

The CORR Procedure

Gene_Symbol=PSAP

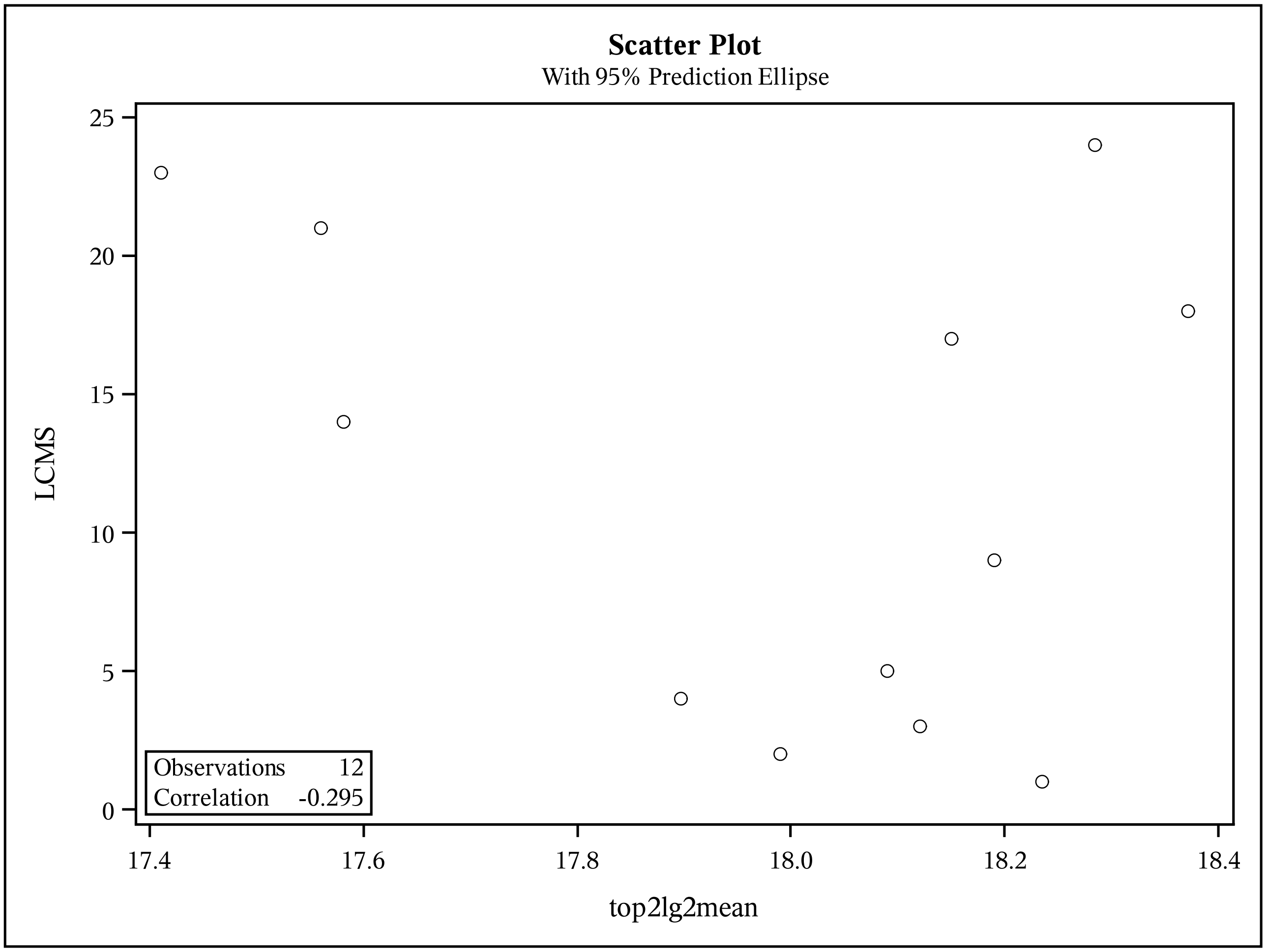

Correlation C_pooltop2lg2mean_tp2 symbol LCMS

The CORR Procedure

Gene_Symbol=PTGDS

2 Variables:	top2lg2mean LCMS

Simple Statistics						
**Variable**	**N**	**Mean**	**Std Dev**	**Sum**	**Minimum**	**Maximum**
**top2lg2mean**	12	23.74446	0.21616	284.93356	23.35282	24.11171
**LCMS**	12	11.75000	8.70867	141.00000	1.00000	24.00000

Pearson Correlation Coefficients, N = 12<br>Prob > |r| under H0: Rho=0		
	**top2lg2mean**	**LCMS**
**top2lg2mean**	1.00000	0.60027<br>0.0391
**LCMS**	0.60027<br>0.0391	1.00000

Pearson Correlation Statistics (Fisher's z Transformation)									
**Variable**	**With Variable**	**N**	**Sample Correlation**	**Fisher's z**	**Bias Adjustment**	**Correlation Estimate**	**95% Confidence Limits**		**p Value for H0:Rho=0**
**top2lg2mean**	**LCMS**	12	0.60027	0.69357	0.02729	0.58253	0.012963	0.866686	0.0375

Correlation C_pooltop2lg2mean_tp2 symbol LCMS

The CORR Procedure

Gene_Symbol=PTGDS

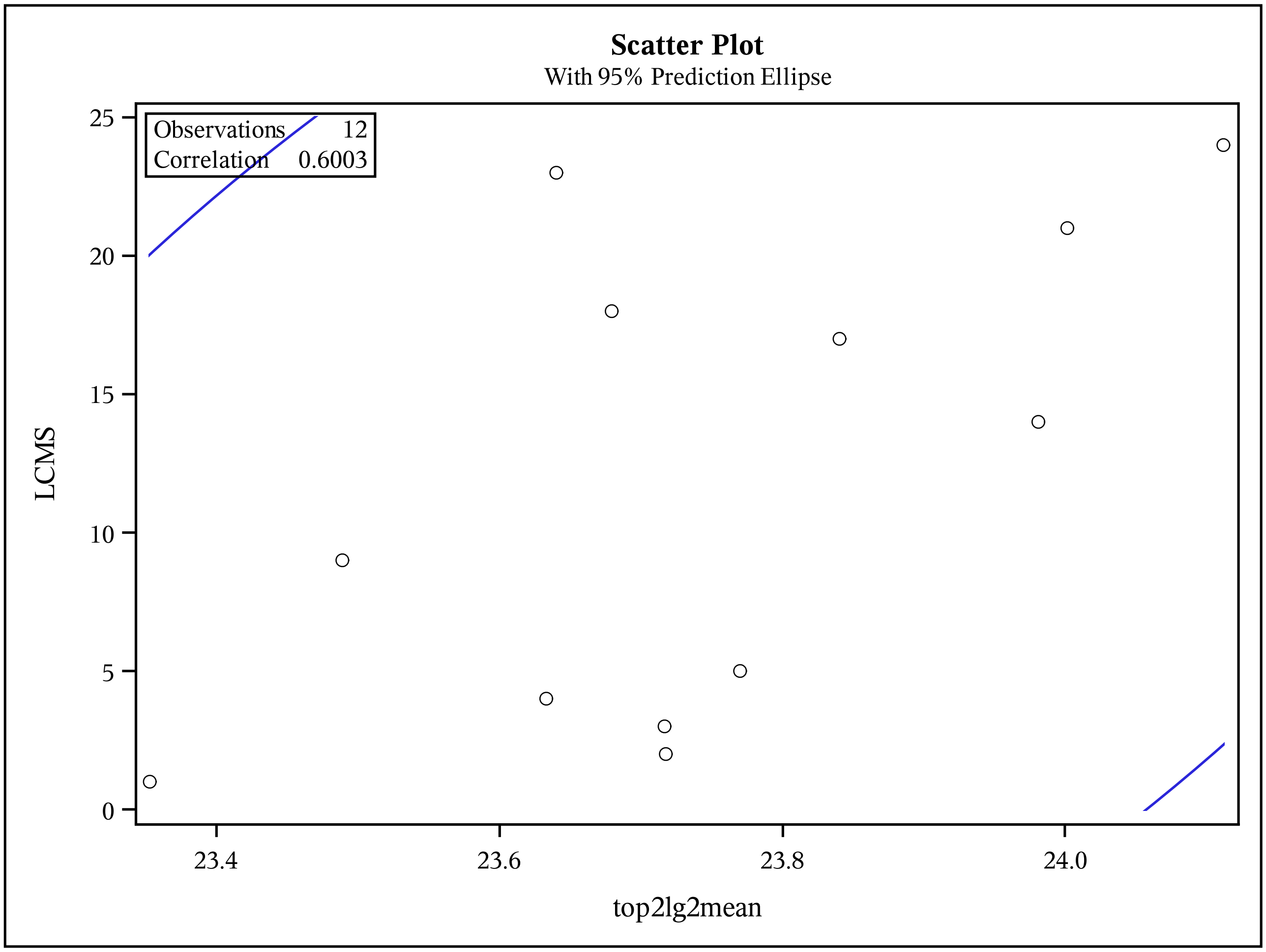

Correlation C_pooltop2lg2mean_tp2 symbol LCMS

The CORR Procedure

Gene_Symbol=RBP4

2 Variables:	top2lg2mean LCMS

Simple Statistics						
**Variable**	**N**	**Mean**	**Std Dev**	**Sum**	**Minimum**	**Maximum**
**top2lg2mean**	12	20.68553	0.17505	248.22639	20.30637	20.87414
**LCMS**	12	11.75000	8.70867	141.00000	1.00000	24.00000

Pearson Correlation Coefficients, N = 12<br>Prob > |r| under H0: Rho=0		
	**top2lg2mean**	**LCMS**
**top2lg2mean**	1.00000	-0.36116<br>0.2487
**LCMS**	-0.36116<br>0.2487	1.00000

Pearson Correlation Statistics (Fisher's z Transformation)									
**Variable**	**With Variable**	**N**	**Sample Correlation**	**Fisher's z**	**Bias Adjustment**	**Correlation Estimate**	**95% Confidence Limits**		**p Value for H0:Rho=0**
**top2lg2mean**	**LCMS**	12	-0.36116	-0.37822	-0.01642	-0.34680	-0.767872	0.283535	0.2565

Correlation C_pooltop2lg2mean_tp2 symbol LCMS

The CORR Procedure

Gene_Symbol=RBP4

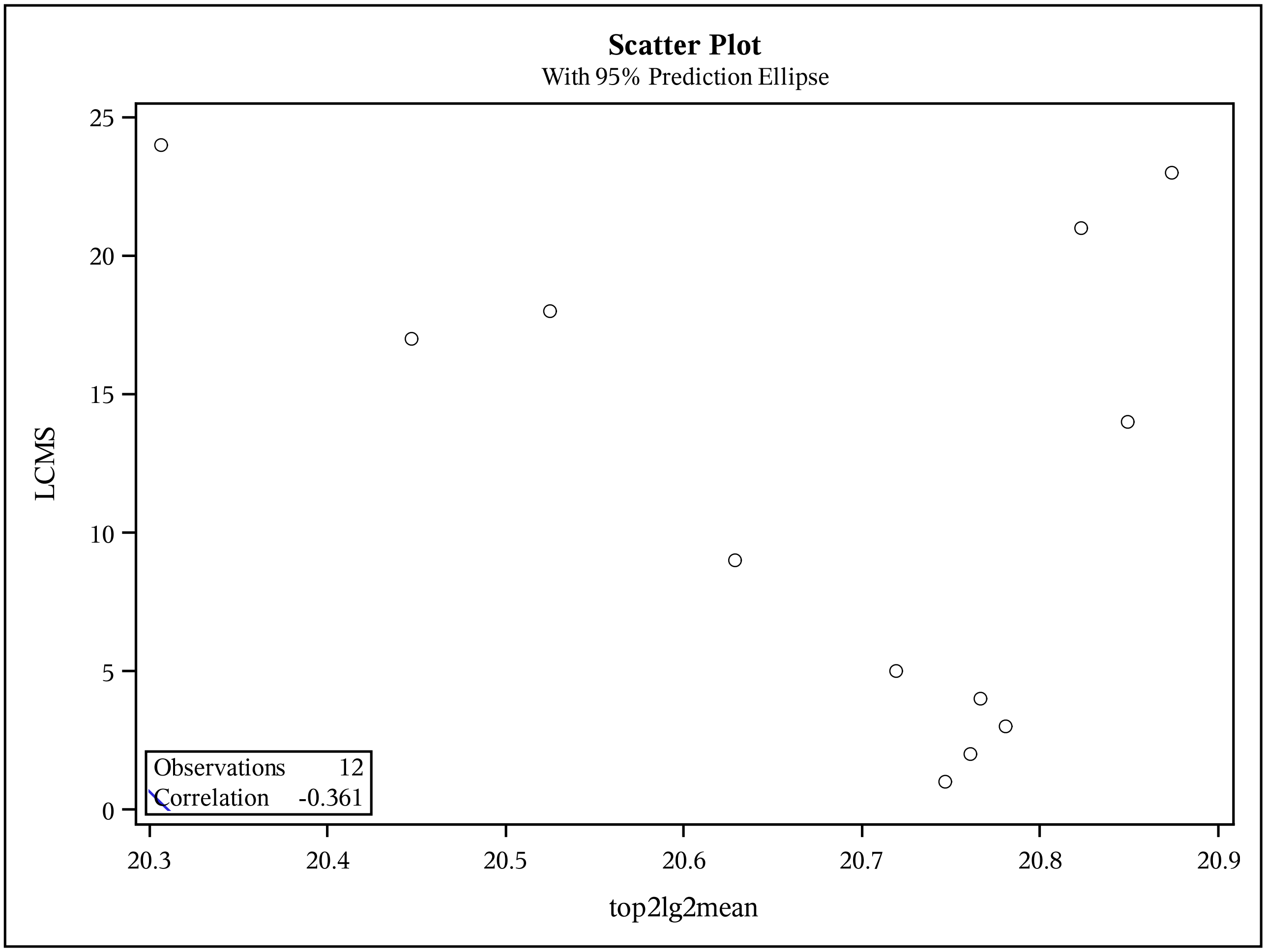

Correlation C_pooltop2lg2mean_tp2 symbol LCMS

The CORR Procedure

Gene_Symbol=RNASE1

**2 Variables:**	top2lg2mean LCMS

Simple Statistics						
**Variable**	**N**	**Mean**	**Std Dev**	**Sum**	**Minimum**	**Maximum**
**top2lg2mean**	12	22.32073	0.75803	267.84872	21.06036	23.40056
**LCMS**	12	11.75000	8.70867	141.00000	1.00000	24.00000

Pearson Correlation Coefficients, N = 12<br>Prob > |r| under H0: Rho=0		
	**top2lg2mean**	**LCMS**
**top2lg2mean**	1.00000	-0.69058<br>0.0129
**LCMS**	-0.69058<br>0.0129	1.00000

Pearson Correlation Statistics (Fisher's z Transformation)									
**Variable**	**With Variable**	**N**	**Sample Correlation**	**Fisher's z**	**Bias Adjustment**	**Correlation Estimate**	**95% Confidence Limits**		**p Value for H0:Rho=0**
**top2lg2mean**	**LCMS**	12	-0.69058	-0.84906	-0.03139	-0.67380	-0.899767	-0.162888	0.0109

Correlation C_pooltop2lg2mean_tp2 symbol LCMS

The CORR Procedure

Gene_Symbol=RNASE1

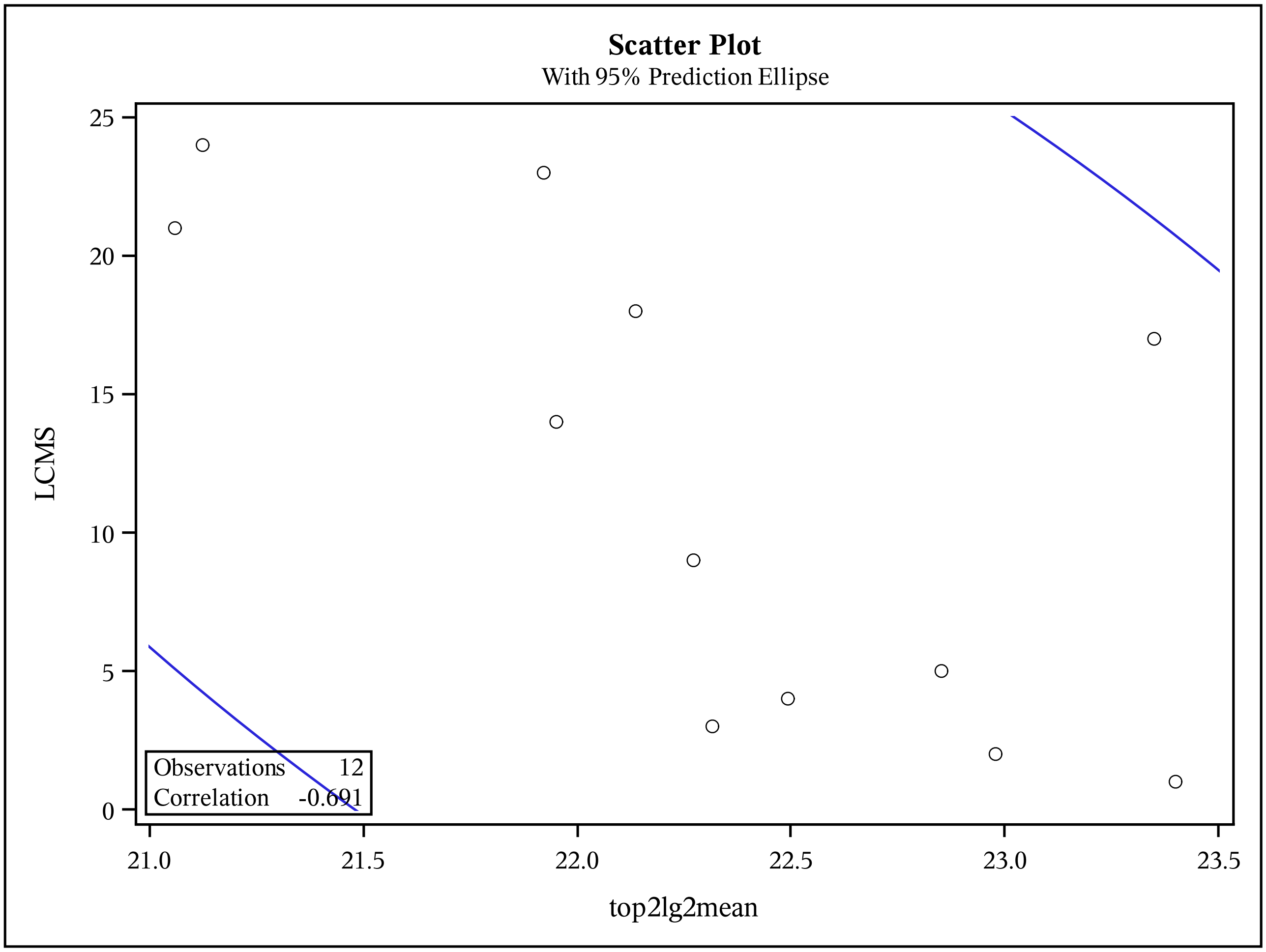

Correlation C_pooltop2lg2mean_tp2 symbol LCMS

The CORR Procedure

Gene_Symbol=SCG3

2 Variables:	top2lg2mean LCMS

Simple Statistics						
Variable	N	Mean	Std Dev	Sum	Minimum	Maximum
top2lg2mean	12	20.88501	0.26995	250.62014	20.48278	21.33450
LCMS	12	11.75000	8.70867	141.00000	1.00000	24.00000

Pearson Correlation Coefficients, N = 12<br>Prob > |r| under H0: Rho=0		
	top2lg2mean	LCMS
top2lg2mean	1.00000	0.13792<br>0.6691
LCMS	0.13792<br>0.6691	1.00000

Pearson Correlation Statistics (Fisher's z Transformation)									
Variable	With Variable	N	Sample Correlation	Fisher's z	Bias Adjustment	Correlation Estimate	95% Confidence Limits		p Value for H0:Rho=0
top2lg2mean	LCMS	12	0.13792	0.13880	0.00627	0.13176	-0.478308	0.656054	0.6771

Correlation C_pooltop2lg2mean_tp2 symbol LCMS

The CORR Procedure

Gene_Symbol=SCG3

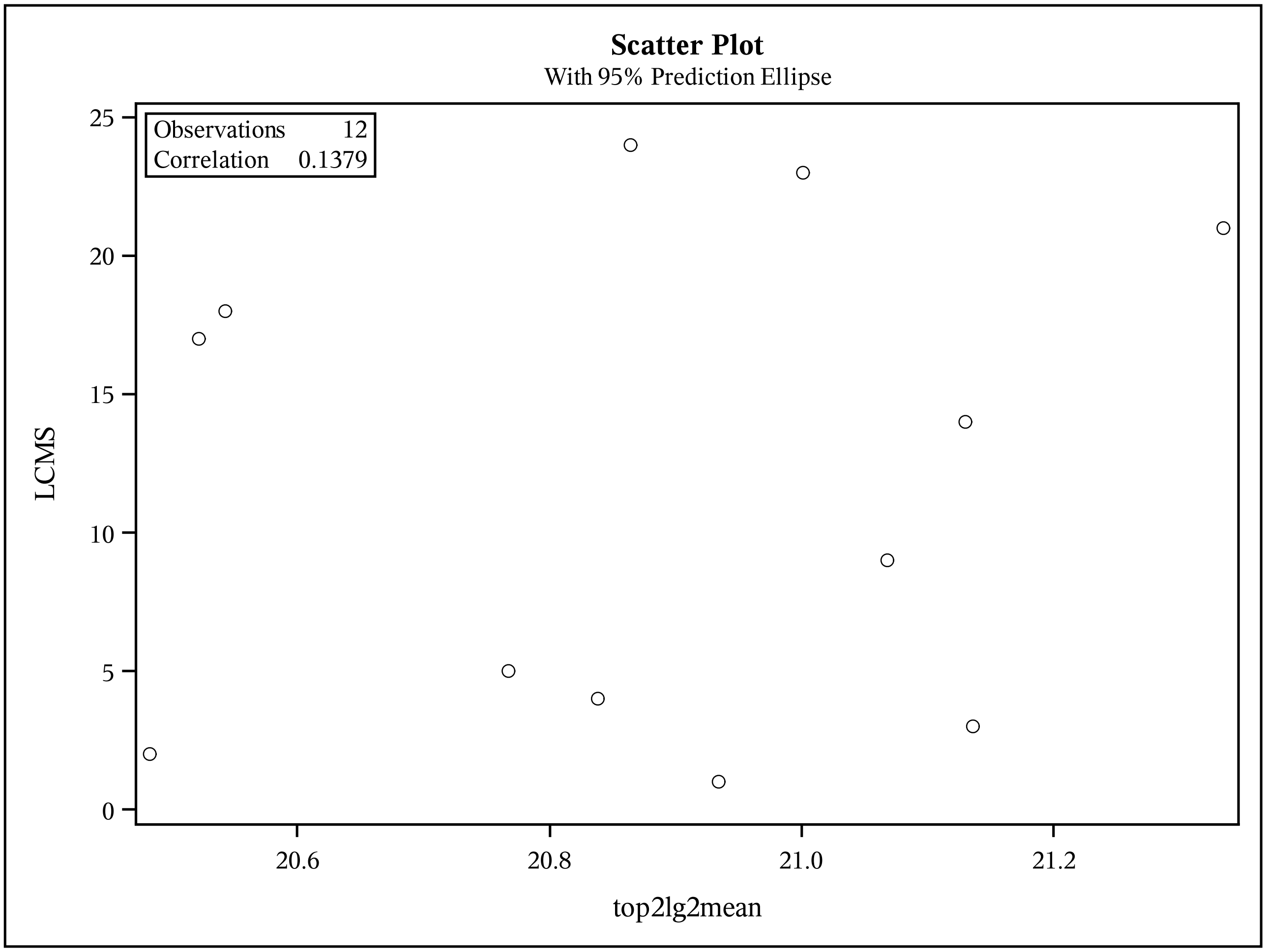

Correlation C_pooltop2lg2mean_tp2 symbol LCMS

The CORR Procedure

Gene_Symbol=SERPINA3

**2 Variables:**	top2lg2mean LCMS

Simple Statistics						
**Variable**	**N**	**Mean**	**Std Dev**	**Sum**	**Minimum**	**Maximum**
**top2lg2mean**	12	22.62516	0.32380	271.50187	22.10855	23.09064
**LCMS**	12	11.75000	8.70867	141.00000	1.00000	24.00000

Pearson Correlation Coefficients, N = 12<br>Prob > |r| under H0: Rho=0		
	**top2lg2mean**	**LCMS**
**top2lg2mean**	1.00000	0.59672<br>0.0405
**LCMS**	0.59672<br>0.0405	1.00000

Pearson Correlation Statistics (Fisher's z Transformation)									
**Variable**	**With Variable**	**N**	**Sample Correlation**	**Fisher's z**	**Bias Adjustment**	**Correlation Estimate**	**95% Confidence Limits**		**p Value for H0:Rho=0**
**top2lg2mean**	**LCMS**	12	0.59672	0.68803	0.02712	0.57897	0.007589	0.865342	0.0390

Correlation C_pooltop2lg2mean_tp2 symbol LCMS

The CORR Procedure

Gene_Symbol=SERPINA3

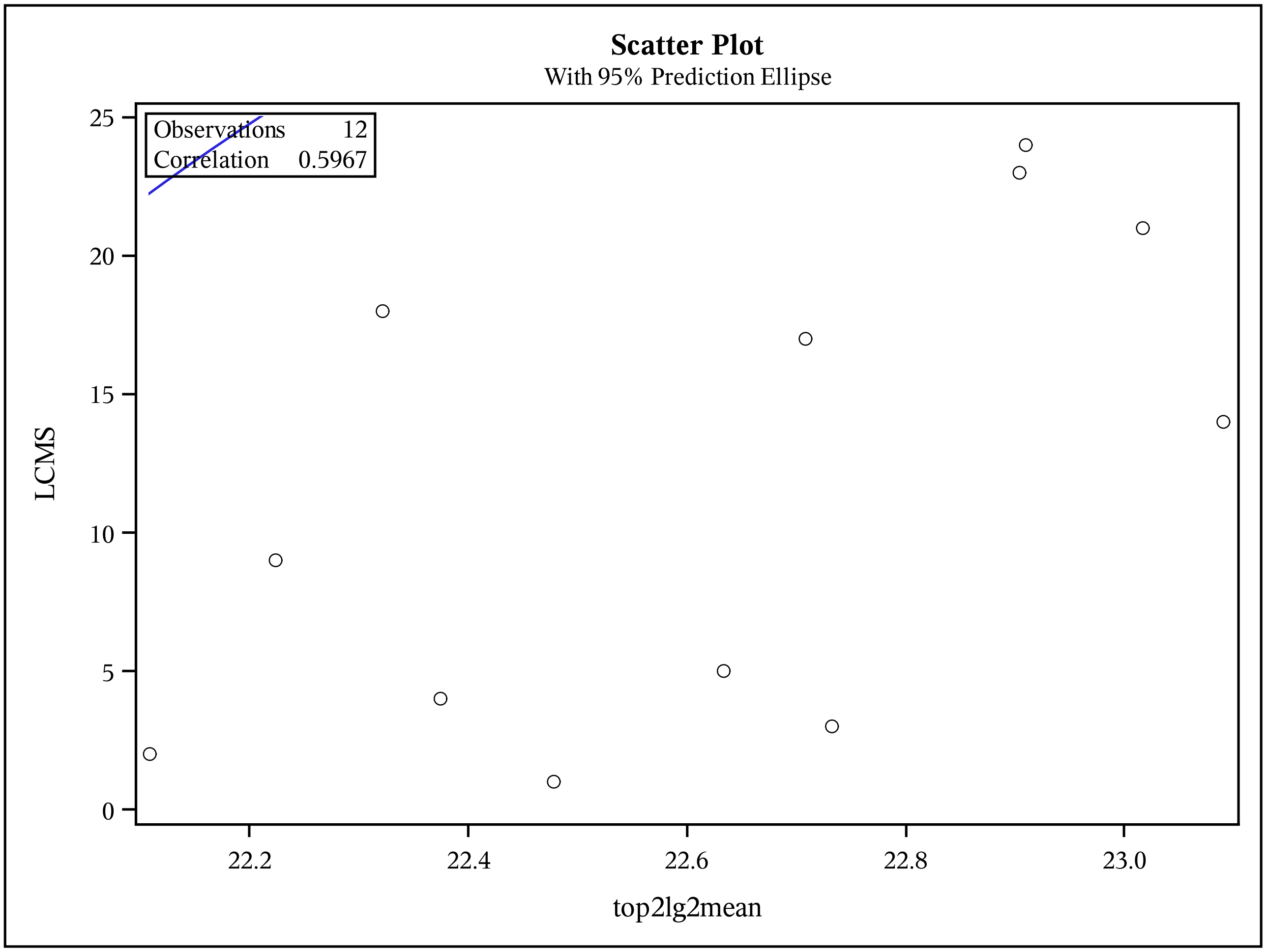

Correlation C_pooltop2lg2mean_tp2 symbol LCMS

The CORR Procedure

Gene_Symbol=SERPINC1

2 Variables:	top2lg2mean LCMS

Simple Statistics						
**Variable**	**N**	**Mean**	**Std Dev**	**Sum**	**Minimum**	**Maximum**
**top2lg2mean**	12	21.46794	0.16544	257.61532	21.17691	21.68697
**LCMS**	12	11.75000	8.70867	141.00000	1.00000	24.00000

Pearson Correlation Coefficients, N = 12<br>Prob > |r| under H0: Rho=0		
	**top2lg2mean**	**LCMS**
**top2lg2mean**	1.00000	0.55169<br>0.0629
**LCMS**	0.55169<br>0.0629	1.00000

Pearson Correlation Statistics (Fisher's z Transformation)									
**Variable**	**With Variable**	**N**	**Sample Correlation**	**Fisher's z**	**Bias Adjustment**	**Correlation Estimate**	**95% Confidence Limits**		**p Value for H0:Rho=0**
**top2lg2mean**	**LCMS**	12	0.55169	0.62081	0.02508	0.53401	-0.057525	0.848018	0.0625

Correlation C_pooltop2lg2mean_tp2 symbol LCMS

The CORR Procedure

Gene_Symbol=SERPINC1

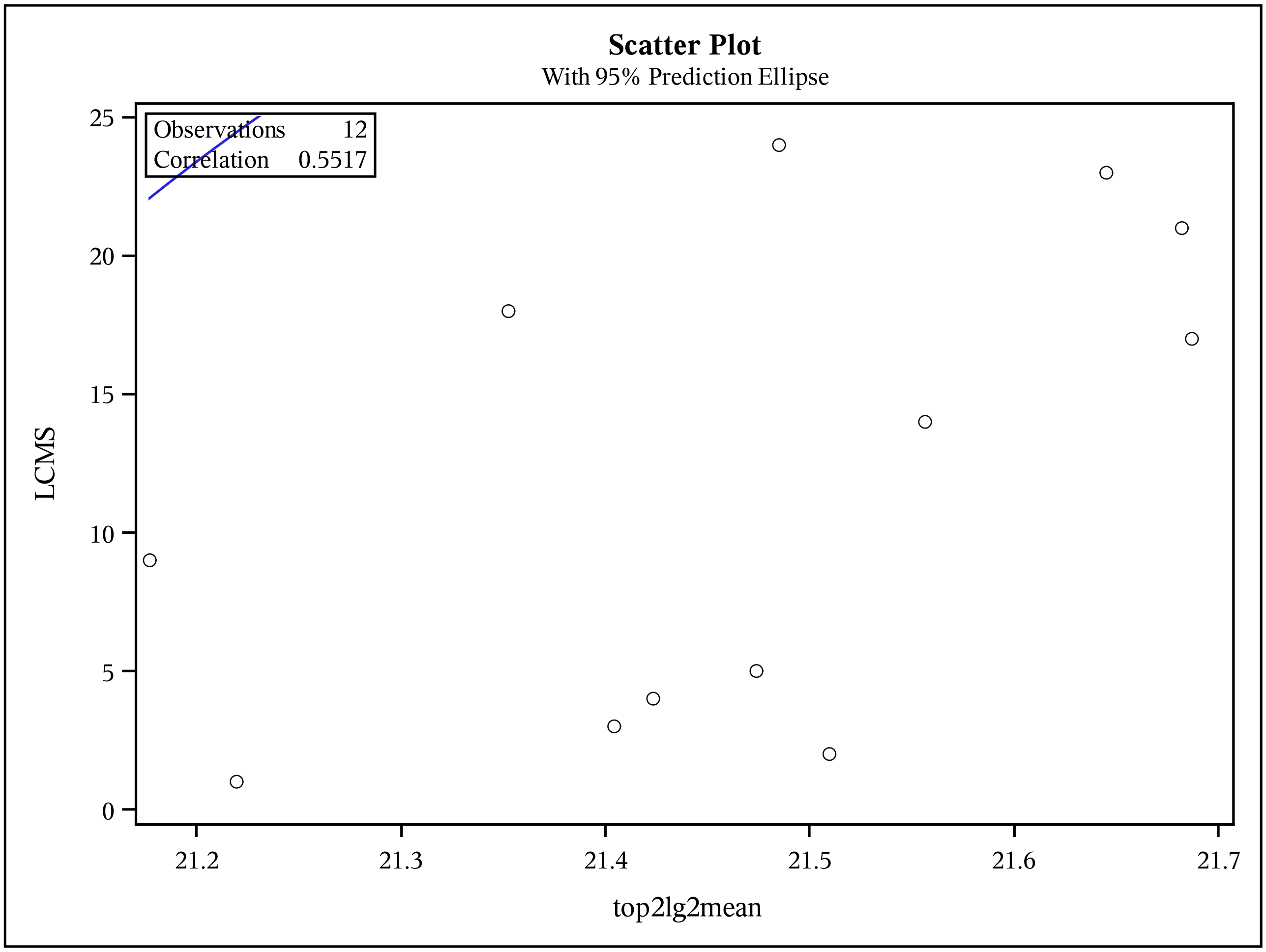

Correlation C_pooltop2lg2mean_tp2 symbol LCMS

The CORR Procedure

Gene_Symbol=SERPIND1

**2 Variables:**	top2lg2mean LCMS

Simple Statistics						
**Variable**	**N**	**Mean**	**Std Dev**	**Sum**	**Minimum**	**Maximum**
**top2lg2mean**	12	17.67289	0.55318	212.07465	16.75935	18.49608
**LCMS**	12	11.75000	8.70867	141.00000	1.00000	24.00000

Pearson Correlation Coefficients, N = 12<br>Prob > |r| under H0: Rho=0		
	**top2lg2mean**	**LCMS**
**top2lg2mean**	1.00000	0.46294<br>0.1296
**LCMS**	0.46294<br>0.1296	1.00000

Pearson Correlation Statistics (Fisher's z Transformation)									
**Variable**	**With Variable**	**N**	**Sample Correlation**	**Fisher's z**	**Bias Adjustment**	**Correlation Estimate**	**95% Confidence Limits**		**p Value for H0:Rho=0**
**top2lg2mean**	**LCMS**	12	0.46294	0.50105	0.02104	0.44625	-0.171603	0.812154	0.1328

Correlation C_pooltop2lg2mean_tp2 symbol LCMS

The CORR Procedure

Gene_Symbol=SERPIND1

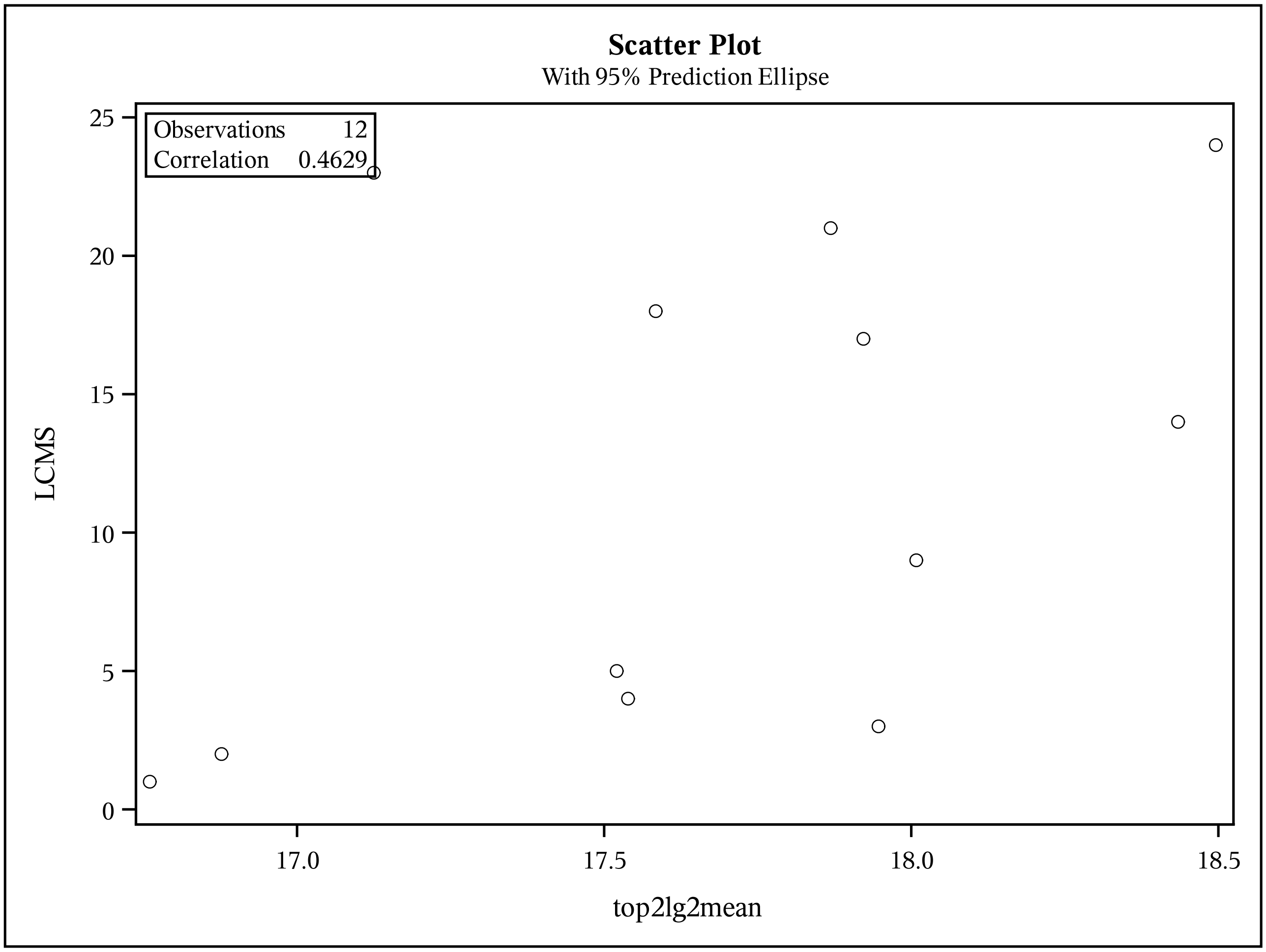

Correlation C_pooltop2lg2mean_tp2 symbol LCMS

The CORR Procedure

Gene_Symbol=SERPINF1

2 Variables:	top2lg2mean LCMS

Simple Statistics						
Variable	N	Mean	Std Dev	Sum	Minimum	Maximum
top2lg2mean	12	22.95120	0.25443	275.41434	22.55917	23.56111
LCMS	12	11.75000	8.70867	141.00000	1.00000	24.00000

Pearson Correlation Coefficients, N = 12<br>Prob > |r| under H0: Rho=0		
	top2lg2mean	LCMS
top2lg2mean	1.00000	0.36005<br>0.2503
LCMS	0.36005<br>0.2503	1.00000

Pearson Correlation Statistics (Fisher's z Transformation)										
Variable	With Variable	N	Sample Correlation	Fisher's z	Bias Adjustment	Correlation Estimate	95% Confidence Limits		p Value for H0:Rho=0
top2lg2mean	LCMS	12	0.36005	0.37694	0.01637	0.34572	-0.284663	0.767368	0.2581

Correlation C_pooltop2lg2mean_tp2 symbol LCMS

The CORR Procedure

Gene_Symbol=SERPINF1

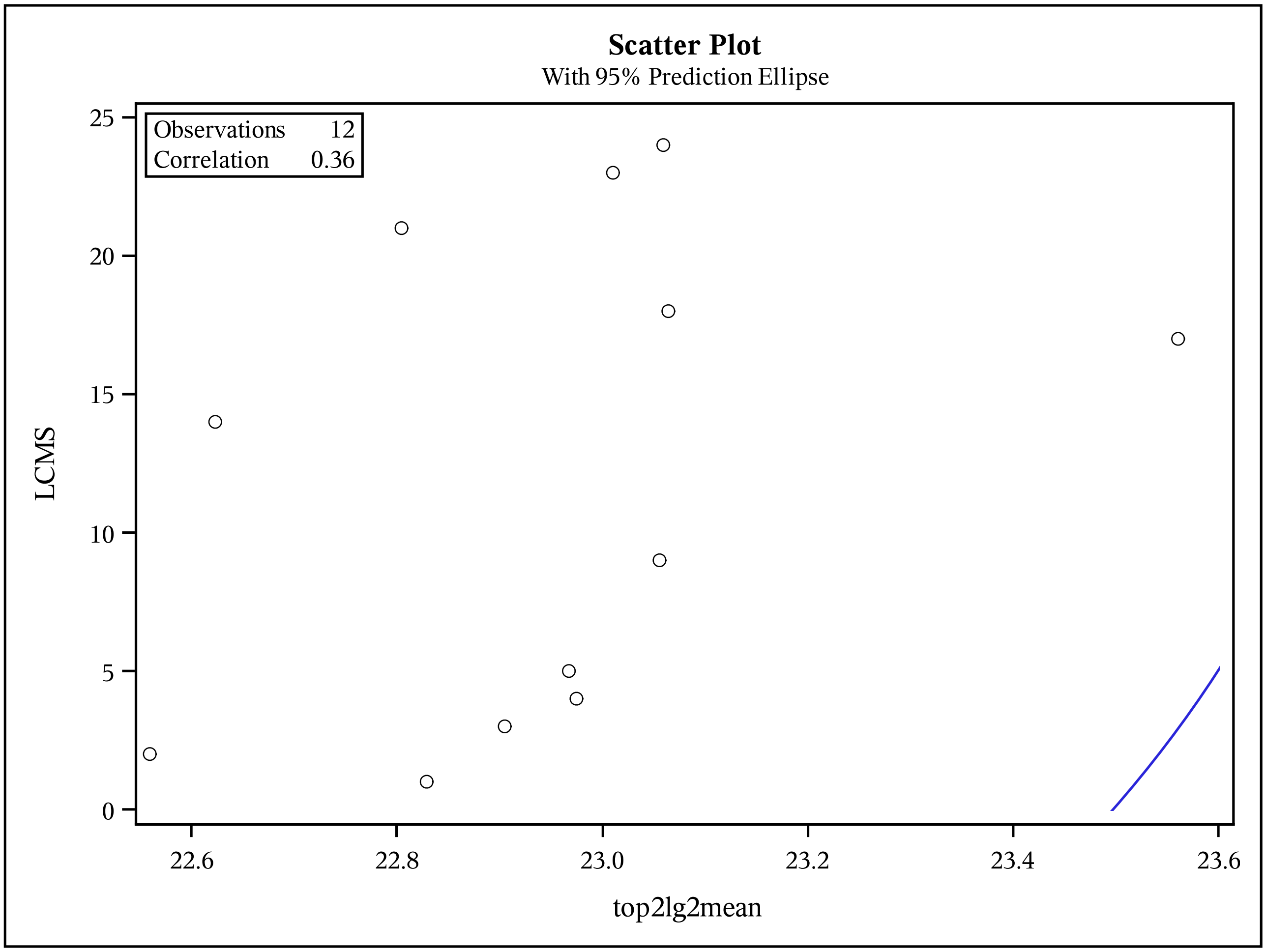

Correlation C_pooltop2lg2mean_tp2 symbol LCMS

The CORR Procedure

Gene_Symbol=SERPING1

2 Variables:	top2lg2mean LCMS

Simple Statistics						
Variable	N	Mean	Std Dev	Sum	Minimum	Maximum
top2lg2mean	12	21.81399	0.49183	261.76791	20.79293	22.57029
LCMS	12	11.75000	8.70867	141.00000	1.00000	24.00000

Pearson Correlation Coefficients, N = 12<br>Prob > |r| under H0: Rho=0		
	top2lg2mean	LCMS
top2lg2mean	1.00000	0.48380<br>0.1110
LCMS	0.48380<br>0.1110	1.00000

Pearson Correlation Statistics (Fisher's z Transformation)									
Variable	With Variable	N	Sample Correlation	Fisher's z	Bias Adjustment	Correlation Estimate	95% Confidence Limits		p Value for H0:Rho=0
top2lg2mean	LCMS	12	0.48380	0.52794	0.02199	0.46678	-0.146317	0.820801	0.1132

Correlation C_pooltop2lg2mean_tp2 symbol LCMS

The CORR Procedure

Gene_Symbol=SERPING1

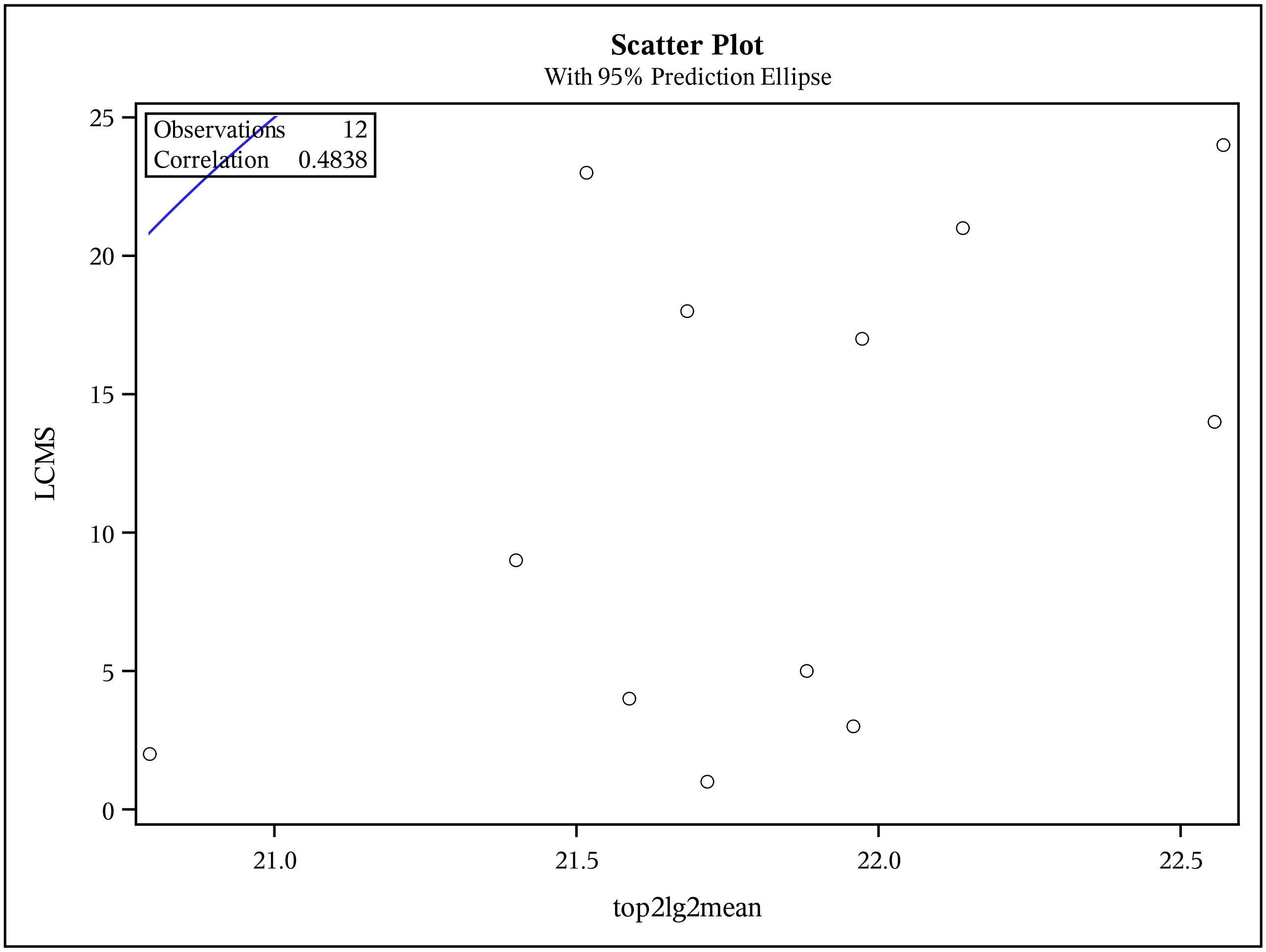

Correlation C_pooltop2lg2mean_tp2 symbol LCMS

The CORR Procedure

Gene_Symbol=SIRPA

2 Variables:	top2lg2mean LCMS

Simple Statistics						
**Variable**	**N**	**Mean**	**Std Dev**	**Sum**	**Minimum**	**Maximum**
**top2lg2mean**	12	18.70502	0.32043	224.46021	18.32002	19.31147
**LCMS**	12	11.75000	8.70867	141.00000	1.00000	24.00000

Pearson Correlation Coefficients, N = 12<br>Prob > |r| under H0: Rho=0		
	**top2lg2mean**	**LCMS**
**top2lg2mean**	1.00000	-0.14800<br>0.6462
**LCMS**	-0.14800<br>0.6462	1.00000

Pearson Correlation Statistics (Fisher's z Transformation)									
**Variable**	**With Variable**	**N**	**Sample Correlation**	**Fisher's z**	**Bias Adjustment**	**Correlation Estimate**	**95% Confidence Limits**		**p Value for H0:Rho=0**
**top2lg2mean**	**LCMS**	12	-0.14800	-0.14910	-0.00673	-0.14141	-0.661620	0.470687	0.6547

Correlation C_pooltop2lg2mean_tp2 symbol LCMS

The CORR Procedure

Gene_Symbol=SIRPA

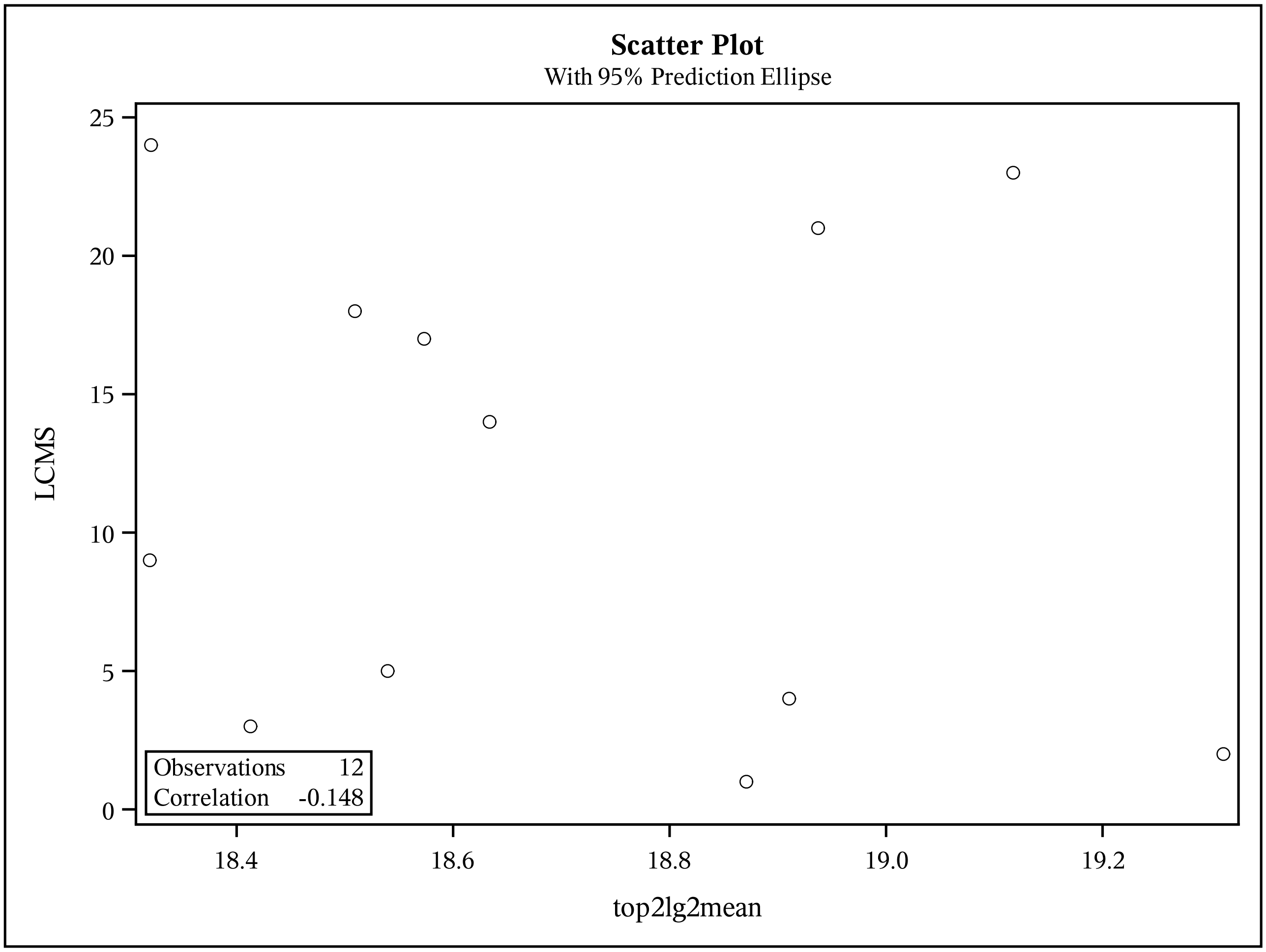

Correlation C_pooltop2lg2mean_tp2 symbol LCMS

The CORR Procedure

Gene_Symbol=SOD1

2 Variables:	top2lg2mean LCMS

Simple Statistics						
Variable	N	Mean	Std Dev	Sum	Minimum	Maximum
top2lg2mean	12	23.16349	0.19007	277.96185	22.94400	23.52580
LCMS	12	11.75000	8.70867	141.00000	1.00000	24.00000

Pearson Correlation Coefficients, N = 12<br>Prob > |r| under H0: Rho=0		
	top2lg2mean	LCMS
top2lg2mean	1.00000	-0.57125<br>0.0524
LCMS	-0.57125<br>0.0524	1.00000

Pearson Correlation Statistics (Fisher's z Transformation)									
Variable	With Variable	N	Sample Correlation	Fisher's z	Bias Adjustment	Correlation Estimate	95% Confidence Limits		p Value for H0:Rho=0
top2lg2mean	LCMS	12	-0.57125	-0.64938	-0.02597	-0.55350	-0.855613	0.029898	0.0514

Correlation C_pooltop2lg2mean_tp2 symbol LCMS

The CORR Procedure

Gene_Symbol=SOD1

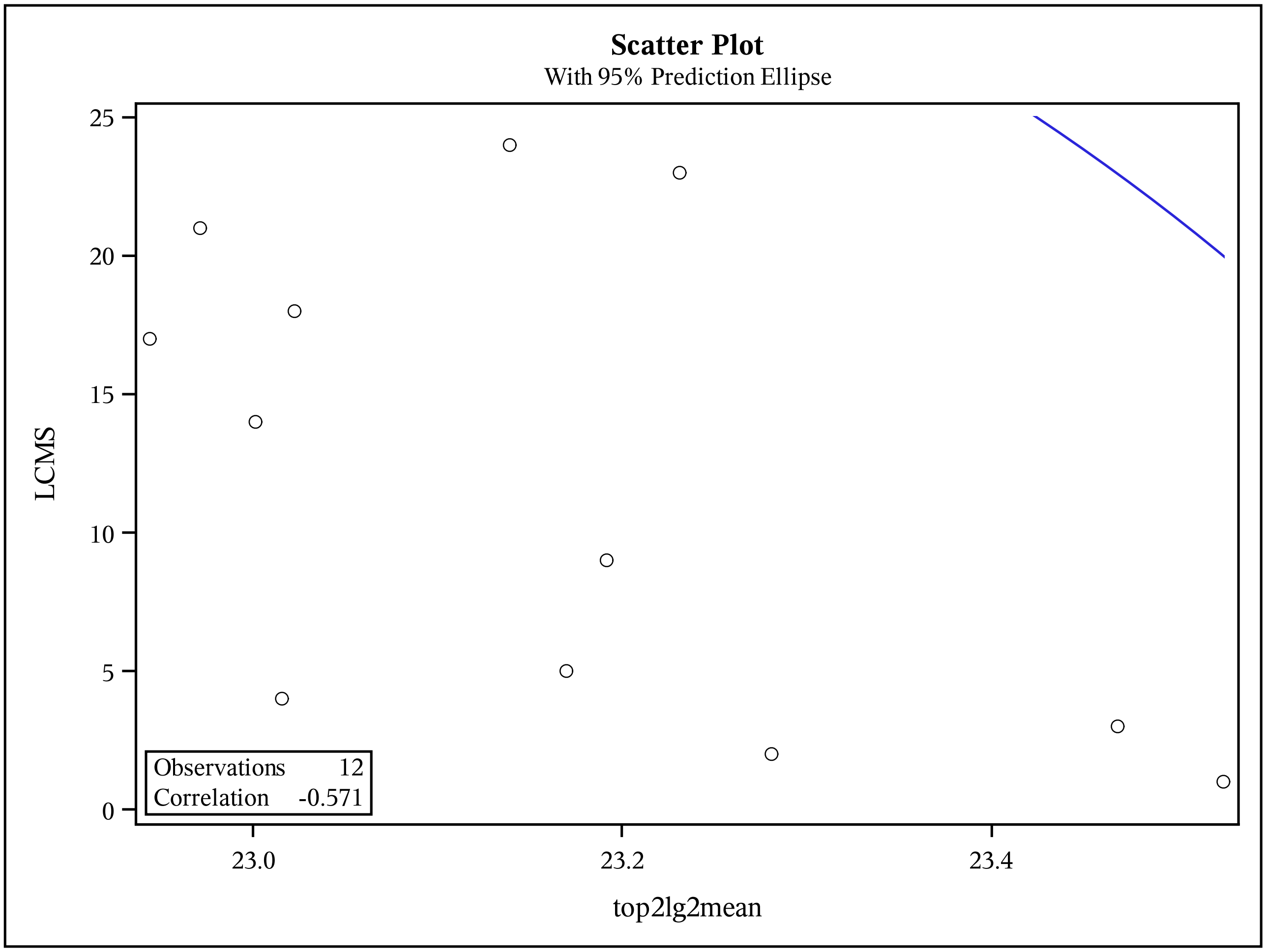

Correlation C_pooltop2lg2mean_tp2 symbol LCMS

The CORR Procedure

Gene_Symbol=SOD3

2 Variables:	top2lg2mean LCMS

Simple Statistics						
Variable	N	Mean	Std Dev	Sum	Minimum	Maximum
top2lg2mean	12	20.89297	0.47158	250.71558	19.97754	21.33323
LCMS	12	11.75000	8.70867	141.00000	1.00000	24.00000

Pearson Correlation Coefficients, N = 12<br>Prob > |r| under H0: Rho=0		
	top2lg2mean	LCMS
top2lg2mean	1.00000	-0.53066<br>0.0759
LCMS	-0.53066<br>0.0759	1.00000

Pearson Correlation Statistics (Fisher's z Transformation)									
Variable	With Variable	N	Sample Correlation	Fisher's z	Bias Adjustment	Correlation Estimate	95% Confidence Limits		p Value for H0:Rho=0
top2lg2mean	LCMS	12	-0.53066	-0.59106	-0.02412	-0.51311	-0.839731	0.086169	0.0762

Correlation C_pooltop2lg2mean_tp2 symbol LCMS

The CORR Procedure

Gene_Symbol=SOD3

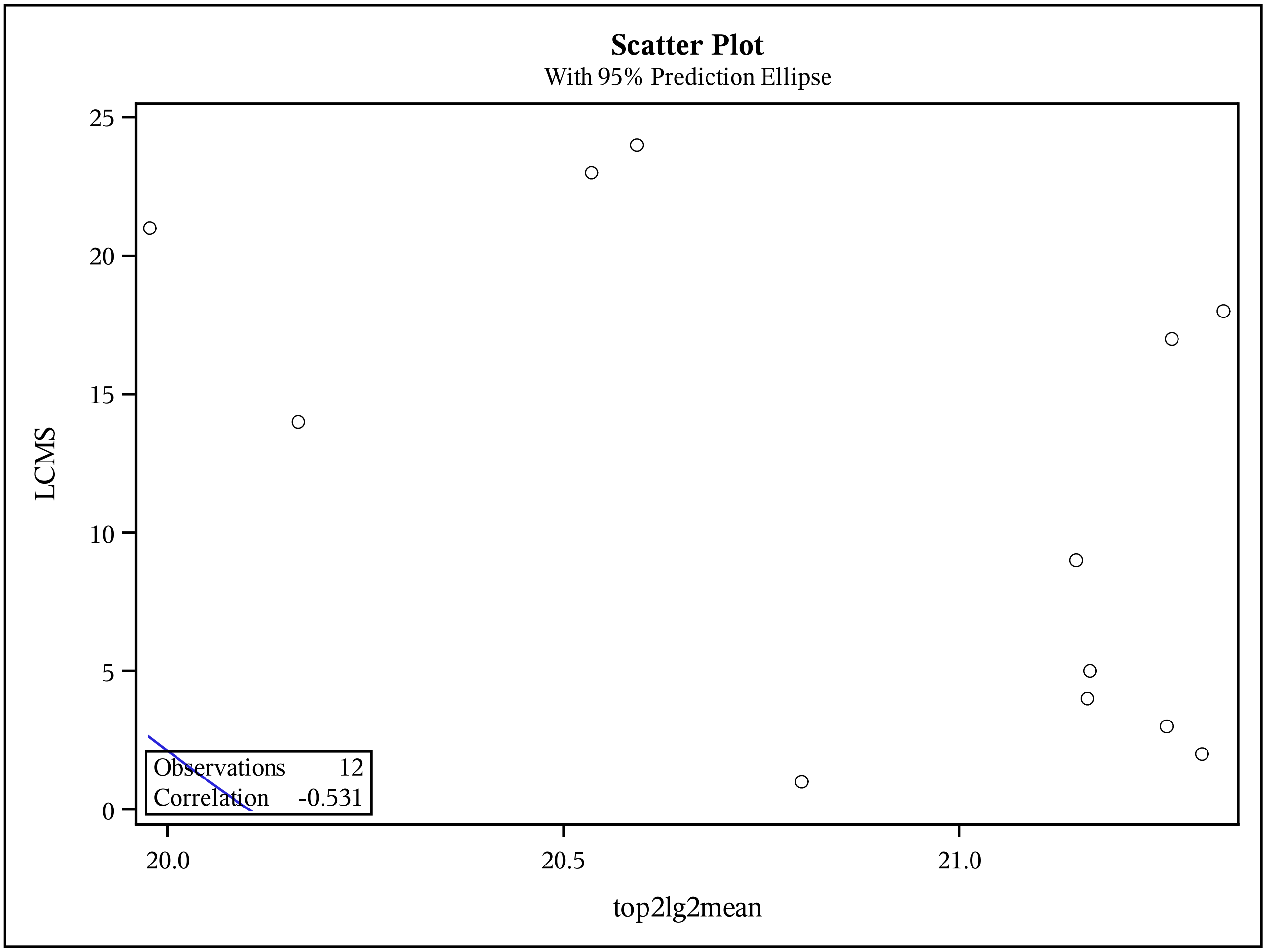

Correlation C_pooltop2lg2mean_tp2 symbol LCMS

The CORR Procedure

Gene_Symbol=SPARCL1

2 Variables:	top2lg2mean LCMS

Simple Statistics						
**Variable**	**N**	**Mean**	**Std Dev**	**Sum**	**Minimum**	**Maximum**
**top2lg2mean**	12	21.56743	0.29155	258.80916	20.82870	22.02751
**LCMS**	12	11.75000	8.70867	141.00000	1.00000	24.00000

Pearson Correlation Coefficients, N = 12<br>Prob > |r| under H0: Rho=0		
	**top2lg2mean**	**LCMS**
**top2lg2mean**	1.00000	0.50771<br>0.0920
**LCMS**	0.50771<br>0.0920	1.00000

Pearson Correlation Statistics (Fisher's z Transformation)									
**Variable**	**With Variable**	**N**	**Sample Correlation**	**Fisher's z**	**Bias Adjustment**	**Correlation Estimate**	**95% Confidence Limits**		**p Value for H0:Rho=0**
**top2lg2mean**	**LCMS**	12	0.50771	0.55965	0.02308	0.49039	-0.116226	0.830544	0.0932

Correlation C_pooltop2lg2mean_tp2 symbol LCMS

The CORR Procedure

Gene_Symbol=SPARCL1

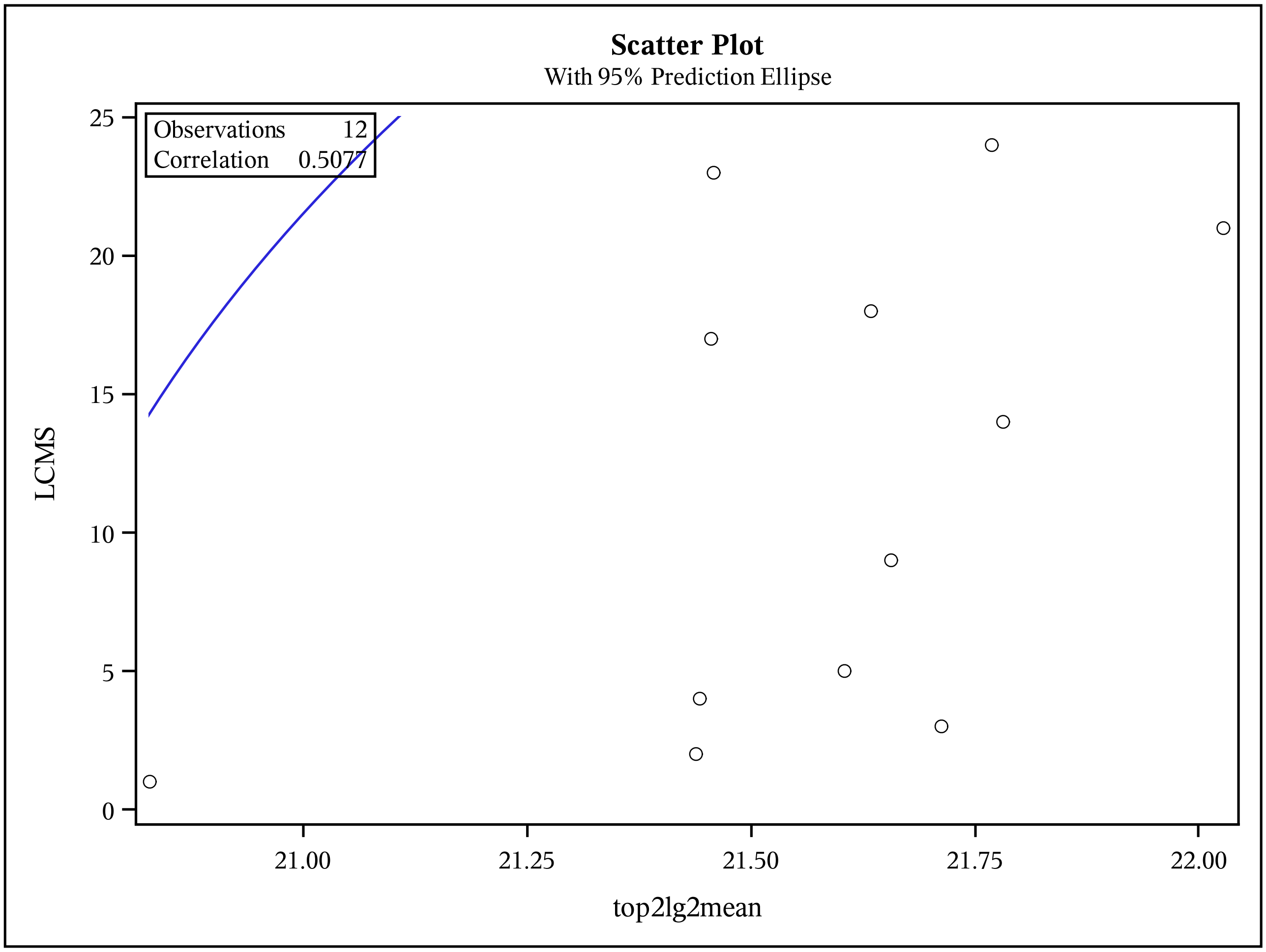

Correlation C_pooltop2lg2mean_tp2 symbol LCMS

The CORR Procedure

Gene_Symbol=SPP1

2 Variables:	top2lg2mean LCMS

Simple Statistics						
Variable	N	Mean	Std Dev	Sum	Minimum	Maximum
top2lg2mean	12	21.53026	0.31804	258.36306	20.94234	21.95977
LCMS	12	11.75000	8.70867	141.00000	1.00000	24.00000

Pearson Correlation Coefficients, N = 12<br>Prob > |r| under H0: Rho=0		
	top2lg2mean	LCMS
top2lg2mean	1.00000	0.27852<br>0.3807
LCMS	0.27852<br>0.3807	1.00000

Pearson Correlation Statistics (Fisher's z Transformation)									
Variable	With Variable	N	Sample Correlation	Fisher's z	Bias Adjustment	Correlation Estimate	95% Confidence Limits		p Value for H0:Rho=0
top2lg2mean	LCMS	12	0.27852	0.28608	0.01266	0.26681	-0.362621	0.729072	0.3908

Correlation C_pooltop2lg2mean_tp2 symbol LCMS

The CORR Procedure

Gene_Symbol=SPP1

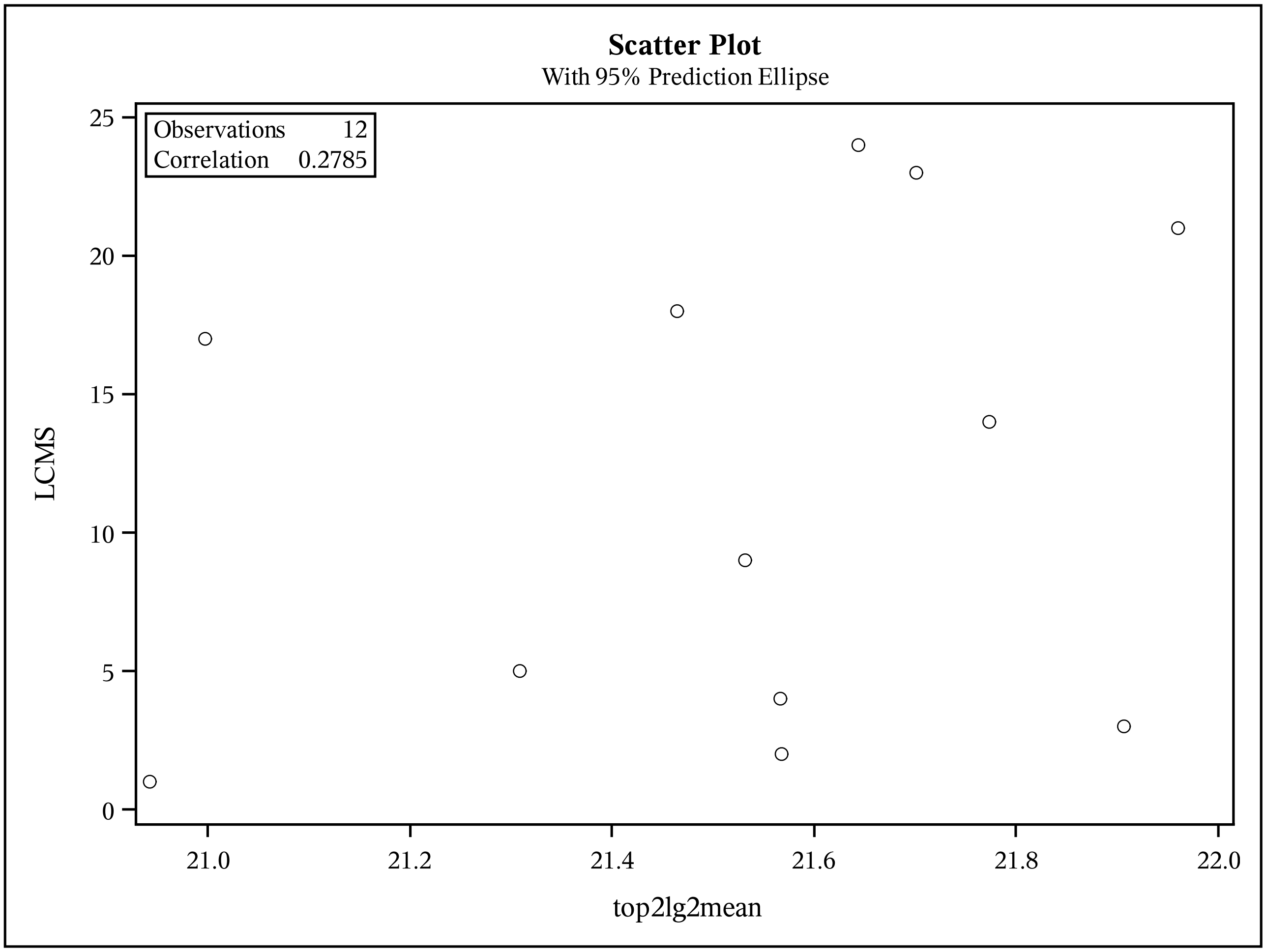

Correlation C_pooltop2lg2mean_tp2 symbol LCMS

The CORR Procedure

Gene_Symbol=THY1

2 Variables:	top2lg2mean LCMS

Simple Statistics						
**Variable**	**N**	**Mean**	**Std Dev**	**Sum**	**Minimum**	**Maximum**
**top2lg2mean**	12	21.42731	0.30687	257.12772	20.79614	21.87430
**LCMS**	12	11.75000	8.70867	141.00000	1.00000	24.00000

Pearson Correlation Coefficients, N = 12<br>Prob > |r| under H0: Rho=0		
	**top2lg2mean**	**LCMS**
**top2lg2mean**	1.00000	0.07268<br>0.8224
**LCMS**	0.07268<br>0.8224	1.00000

Pearson Correlation Statistics (Fisher's z Transformation)									
**Variable**	**With Variable**	**N**	**Sample Correlation**	**Fisher's z**	**Bias Adjustment**	**Correlation Estimate**	**95% Confidence Limits**		**p Value for H0:Rho=0**
**top2lg2mean**	**LCMS**	12	0.07268	0.07281	0.00330	0.06939	-0.525436	0.618655	0.8271

Correlation C_pooltop2lg2mean_tp2 symbol LCMS

The CORR Procedure

Gene_Symbol=THY1

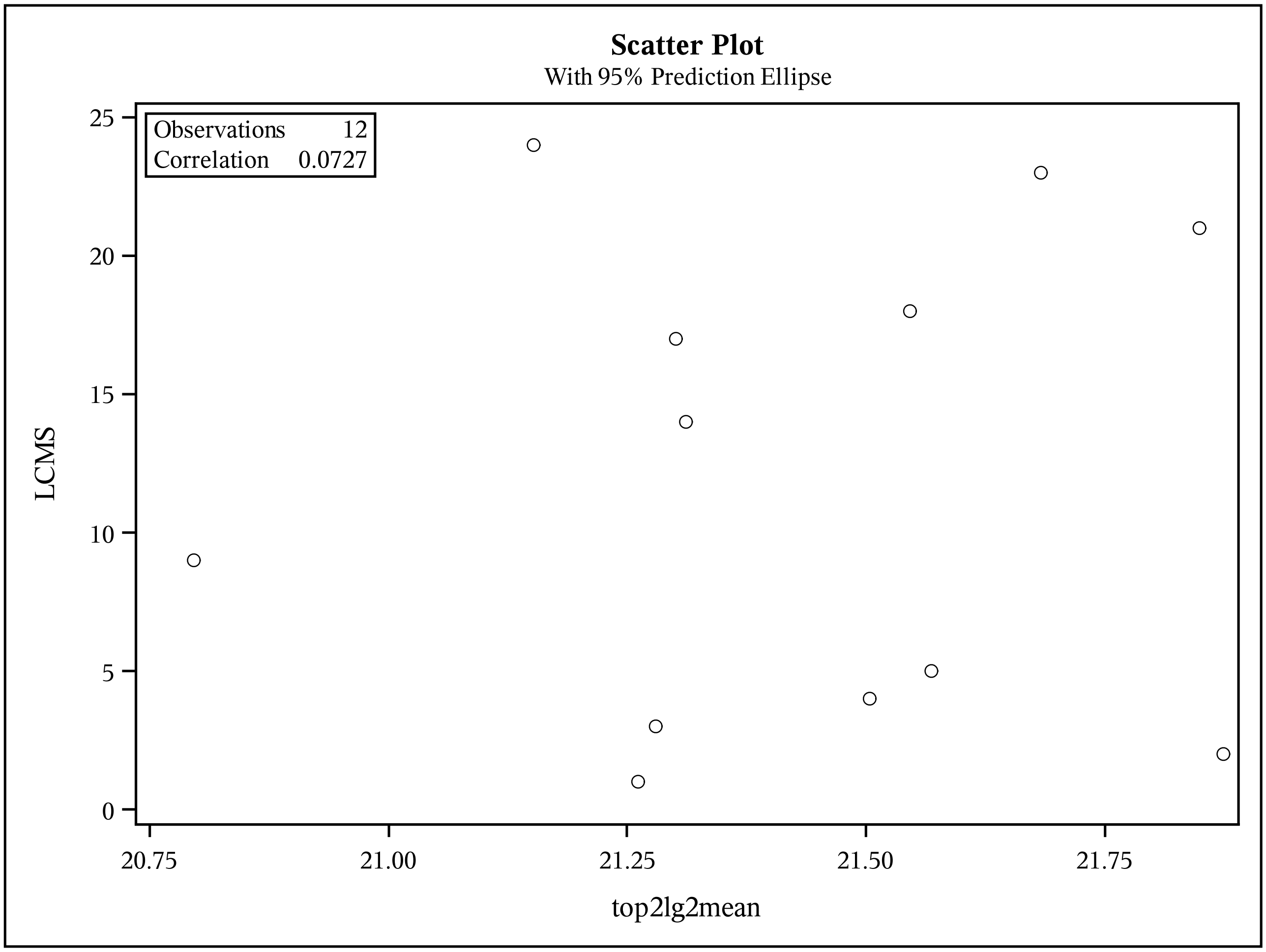

Correlation C_pooltop2lg2mean_tp2 symbol LCMS

The CORR Procedure

Gene_Symbol=TTR

**2 Variables:**	top2lg2mean LCMS

Simple Statistics						
**Variable**	**N**	**Mean**	**Std Dev**	**Sum**	**Minimum**	**Maximum**
**top2lg2mean**	12	27.71611	0.13881	332.59337	27.42233	27.91455
**LCMS**	12	11.75000	8.70867	141.00000	1.00000	24.00000

Pearson Correlation Coefficients, N = 12<br>Prob > |r| under H0: Rho=0		
	**top2lg2mean**	**LCMS**
**top2lg2mean**	1.00000	0.46726<br>0.1256
**LCMS**	0.46726<br>0.1256	1.00000

Pearson Correlation Statistics (Fisher's z Transformation)									
**Variable**	**With Variable**	**N**	**Sample Correlation**	**Fisher's z**	**Bias Adjustment**	**Correlation Estimate**	**95% Confidence Limits**		**p Value for H0:Rho=0**
**top2lg2mean**	**LCMS**	12	0.46726	0.50656	0.02124	0.45050	-0.166433	0.813958	0.1286

Correlation C_pooltop2lg2mean_tp2 symbol LCMS

The CORR Procedure

Gene_Symbol=TTR

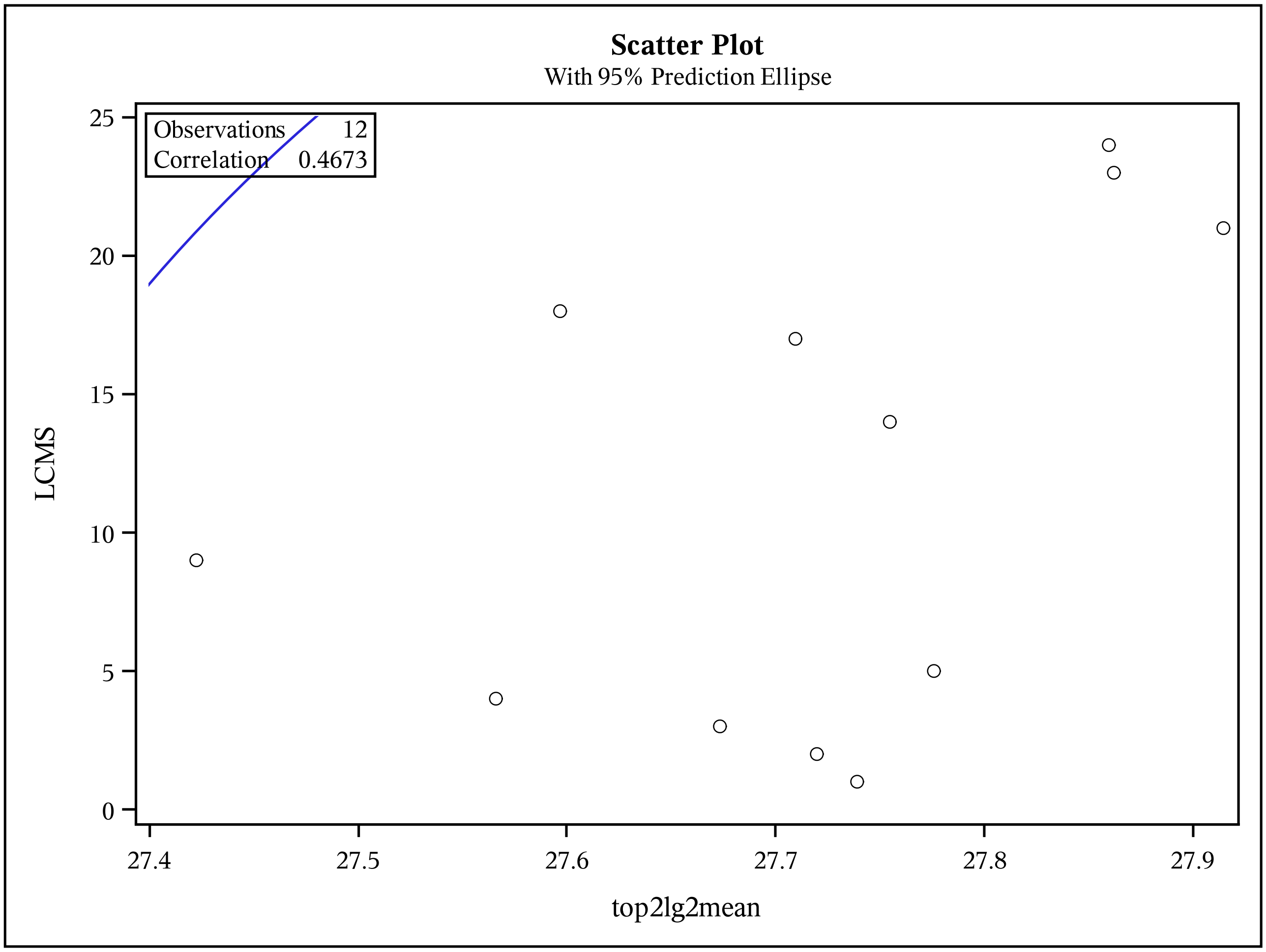

Correlation C_pooltop2lg2mean_tp2 symbol LCMS

The CORR Procedure

Gene_Symbol=UBA52

2 Variables:	top2lg2mean LCMS

Simple Statistics						
**Variable**	**N**	**Mean**	**Std Dev**	**Sum**	**Minimum**	**Maximum**
**top2lg2mean**	12	20.04327	0.66303	240.51925	18.84271	21.12546
**LCMS**	12	11.75000	8.70867	141.00000	1.00000	24.00000

Pearson Correlation Coefficients, N = 12<br>Prob > |r| under H0: Rho=0		
	**top2lg2mean**	**LCMS**
**top2lg2mean**	1.00000	0.05962<br>0.8540
**LCMS**	0.05962<br>0.8540	1.00000

Pearson Correlation Statistics (Fisher's z Transformation)									
**Variable**	**With Variable**	**N**	**Sample Correlation**	**Fisher's z**	**Bias Adjustment**	**Correlation Estimate**	**95% Confidence Limits**		**p Value for H0:Rho=0**
**top2lg2mean**	**LCMS**	12	0.05962	0.05969	0.00271	0.05692	-0.534439	0.610868	0.8579

Correlation C_pooltop2lg2mean_tp2 symbol LCMS

The CORR Procedure

Gene_Symbol=UBA52

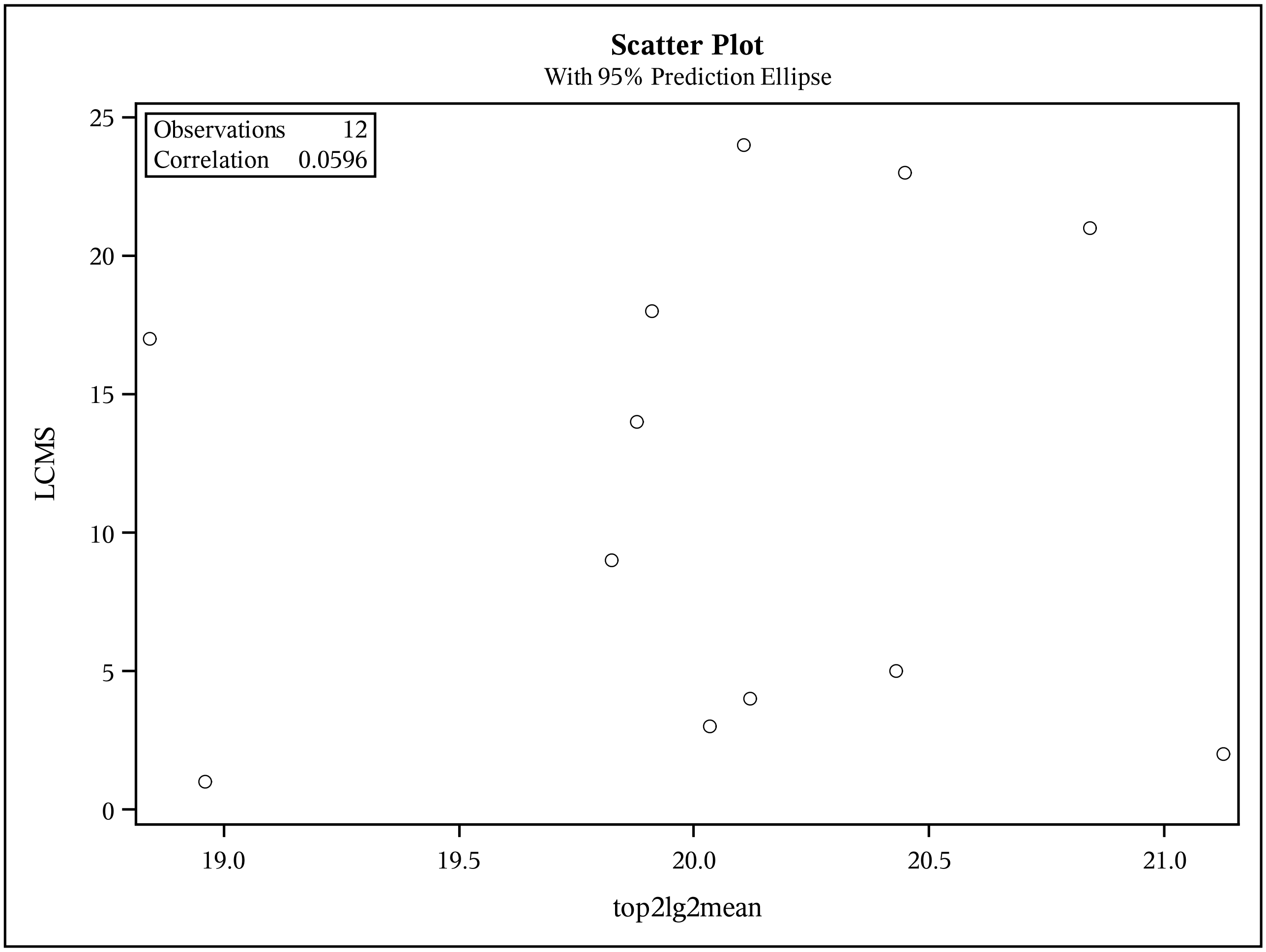

Correlation C_pooltop2lg2mean_tp2 symbol LCMS

The CORR Procedure

Gene_Symbol=VGF

2 Variables:	top2lg2mean LCMS

Simple Statistics						
Variable	N	Mean	Std Dev	Sum	Minimum	Maximum
top2lg2mean	12	17.78075	0.64762	213.36900	16.63359	18.78800
LCMS	12	11.75000	8.70867	141.00000	1.00000	24.00000

Pearson Correlation Coefficients, N = 12<br>Prob > |r| under H0: Rho=0		
	top2lg2mean	LCMS
top2lg2mean	1.00000	-0.27675<br>0.3839
LCMS	-0.27675<br>0.3839	1.00000

Pearson Correlation Statistics (Fisher's z Transformation)									
Variable	With Variable	N	Sample Correlation	Fisher's z	Bias Adjustment	Correlation Estimate	95% Confidence Limits		p Value for H0:Rho=0
top2lg2mean	LCMS	12	-0.27675	-0.28416	-0.01258	-0.26509	-0.728207	0.364221	0.3940

Correlation C_pooltop2lg2mean_tp2 symbol LCMS

The CORR Procedure

Gene_Symbol=VGF

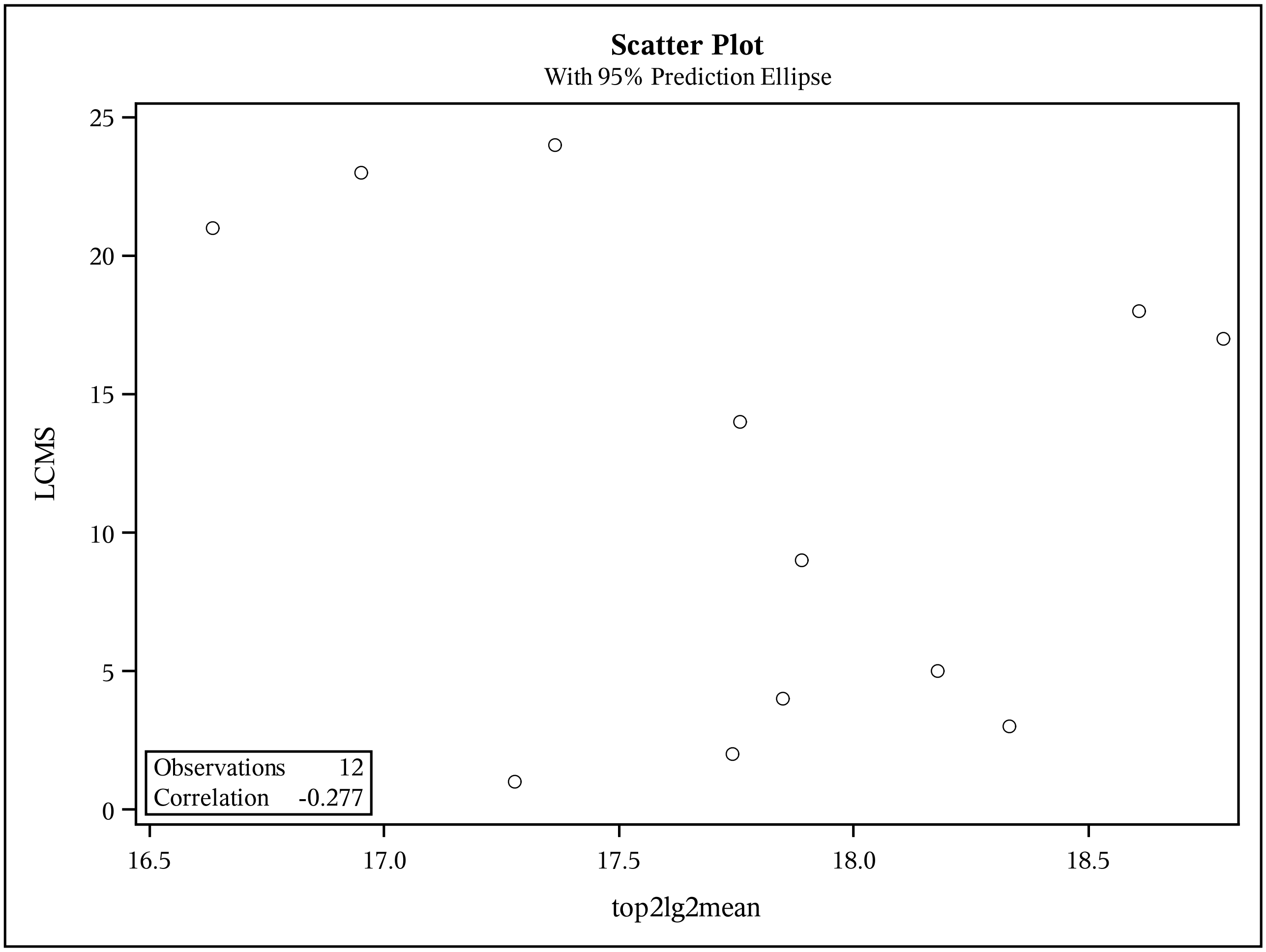

Correlation C_pooltop2lg2mean_tp2 symbol LCMS

The CORR Procedure

Gene_Symbol=VSTM2A

2 Variables:	top2lg2mean LCMS

Simple Statistics						
Variable	N	Mean	Std Dev	Sum	Minimum	Maximum
top2lg2mean	12	18.95094	0.20903	227.41134	18.48949	19.22659
LCMS	12	11.75000	8.70867	141.00000	1.00000	24.00000

Pearson Correlation Coefficients, N = 12<br>Prob > |r| under H0: Rho=0		
	top2lg2mean	LCMS
top2lg2mean	1.00000	0.58370<br>0.0463
LCMS	0.58370<br>0.0463	1.00000

Pearson Correlation Statistics (Fisher's z Transformation)									
Variable	With Variable	N	Sample Correlation	Fisher's z	Bias Adjustment	Correlation Estimate	95% Confidence Limits		p Value for H0:Rho=0
top2lg2mean	LCMS	12	0.58370	0.66806	0.02653	0.56594	-0.011792	0.860391	0.0451

Correlation C_pooltop2lg2mean_tp2 symbol LCMS

The CORR Procedure

Gene_Symbol=VSTM2A

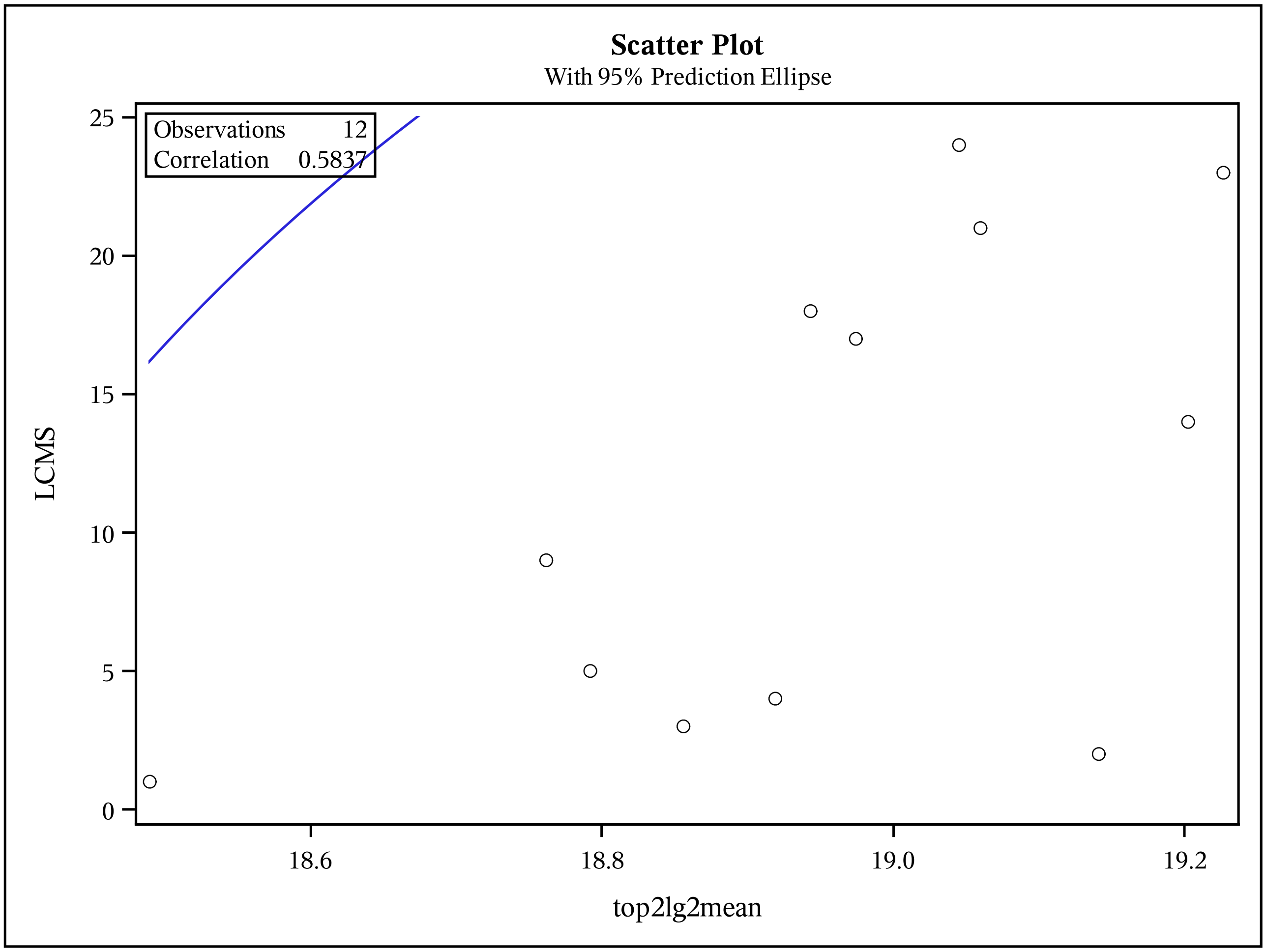

Correlation C_pooltop2lg2mean_tp2 symbol LCMS

The CORR Procedure

Gene_Symbol=VTN

**2 Variables:**	top2lg2mean LCMS

Simple Statistics						
**Variable**	**N**	**Mean**	**Std Dev**	**Sum**	**Minimum**	**Maximum**
**top2lg2mean**	12	16.88153	0.66936	202.57832	16.23902	18.58806
**LCMS**	12	11.75000	8.70867	141.00000	1.00000	24.00000

Pearson Correlation Coefficients, N = 12<br>Prob > |r| under H0: Rho=0		
	**top2lg2mean**	**LCMS**
**top2lg2mean**	1.00000	-0.08102<br>0.8024
**LCMS**	-0.08102<br>0.8024	1.00000

Pearson Correlation Statistics (Fisher's z Transformation)									
**Variable**	**With Variable**	**N**	**Sample Correlation**	**Fisher's z**	**Bias Adjustment**	**Correlation Estimate**	**95% Confidence Limits**		**p Value for H0:Rho=0**
**top2lg2mean**	**LCMS**	12	-0.08102	-0.08120	-0.00368	-0.07736	-0.623577	0.519611	0.8075

Correlation C_pooltop2lg2mean_tp2 symbol LCMS

The CORR Procedure

Gene_Symbol=VTN

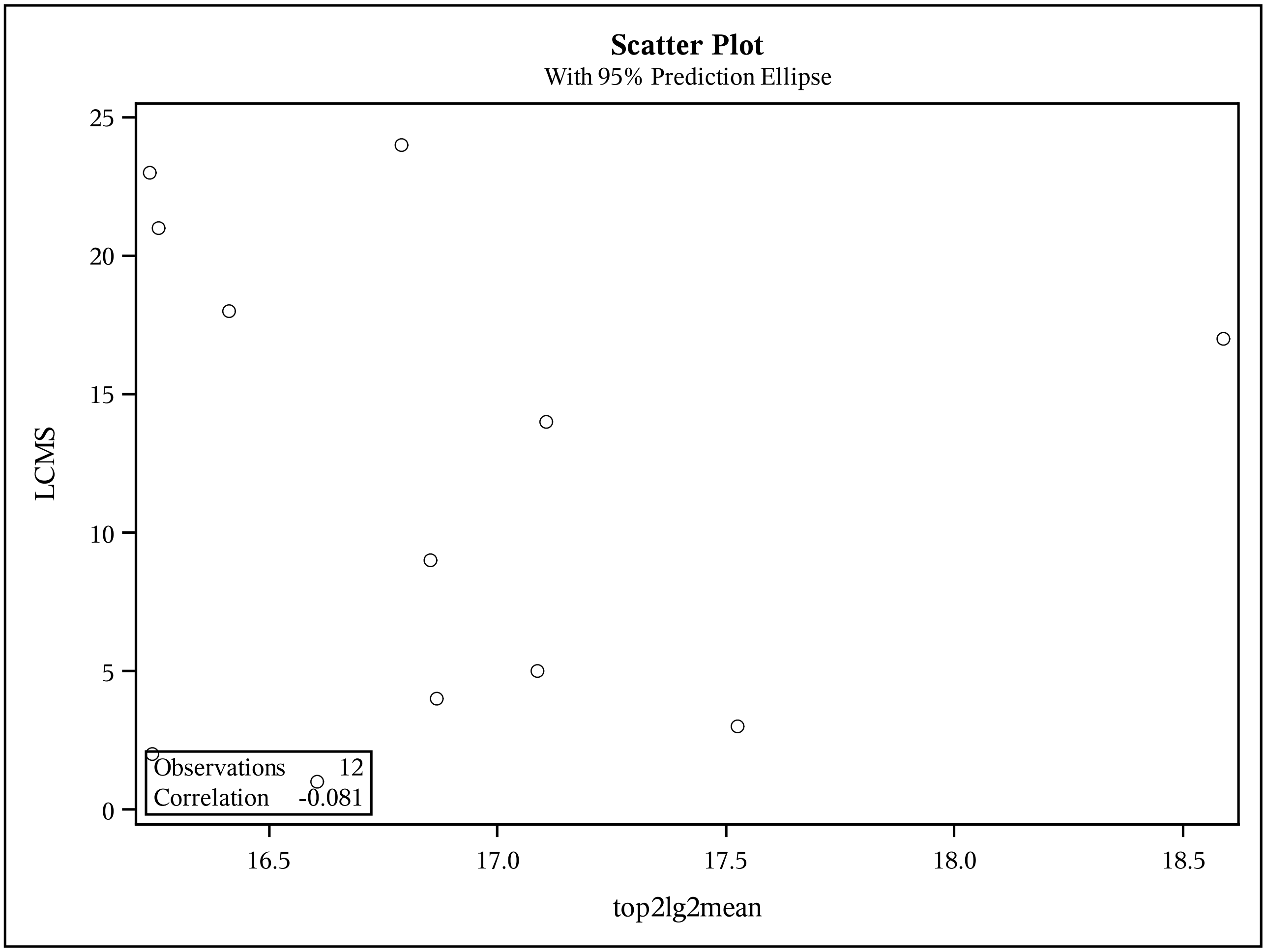

Supplement: Figure S13 — Influence of LC-MS run order on protein abundance measurements. For each of 81 proteins (organized alphabetically by gene symbol of origin), log2 transformed abundance values (calculated from the mean of the two most abundant peptides) for each of the pooled sample replicates are plotted versus LC-MS run order. Pearson correlation coefficients and statistics (Fisher's z transformation) are listed for each protein. (PDF) [file pone.0064314.s013.pdf]
